# Supplementary material for: The Healthy Food Environment Policy Index in Poland: Implementation Gaps and Actions for Improvement
Source: Foods. 2022 Jun 2;11(11):1648. doi: 10.3390/foods11111648 (PMC9180244; doi:10.3390/foods11111648)
Supplement: Supplementary file 1 [file foods-11-01648-s001.zip › File 1 Food-EPI indicators.pdf]

ANNEX 1

JPI
a healthy diet for a healthy life

PEN
Policy Evaluation Network

Effectiveness of existing policies for lifestyle interventions - Policy Evaluation Network (PEN)

Work package 1: Policy mapping and EPI development

Milestone 1.1.2: Food-EPI indicators have been set in each country

This document contains the finalised revised indicators which will be used in all PEN and STOP countries conducting a Food EPI.

FOOD EPI

DOMAIN 1 - FOOD COMPOSITION - Food composition targets/standards/restrictions for processed foods: There are government systems implemented to ensure that, where practicable, processed foods minimise the energy density and the nutrients of concern (salt, saturated fat, trans fat, added sugar)	
**Indicator definitions** - please take these into account in the rating process	**SCOPE (FOR INFORMATION ONLY)**<br>**This colum provides the type evidence collected for this indicator (as well as the evidence that will not be taken into account).**
**INDICATOR (COMP) 1: Food composition targets/standards/restrictions have been established by the government for the content of the nutrients of concern (trans fats, added sugars, salt, saturated fat) in industrially processed foods, in particular for those food groups that are major contributors to population intakes of those nutrients of concern**<br><br>*(Trans fat has been excluded at national level as it falls under EU regulation)*	➢ Includes packaged foods manufactured in country X or manufactured overseas and imported to country X for sale.<br>➢ Includes packaged, ready-to-eat meals sold in supermarkets.<br>➢ Includes mandatory or voluntary targets, standards (e.g., reduce by X%, maximum mg/g per 100g or per serving).<br>➢ Includes legislated ban on nutrients of concern.<br>➢ Excludes legislated restrictions related to other ingredients (e.g. additives).<br>➢ Excludes mandatory food composition regulation related to vitamins and micronutrients (e.g. folic acid or iodine fortification)<br>➢ Excludes food consumption standards/targets for fibre, healthy ingredients like fruits and vegetables<br>➢ Excludes food composition of ready-to-eat meals sold in food service outlets (see COMP2)<br>➢ Excludes general guidelines advising food companies to reduce nutrients of concern.<br>➢ Excludes the provision of resources or expertise to support individual food companies with reformulation.<br>➢ Industrially processed foods are the processed and ultra-processed foods according to the **NOVA classification** (please find the complete definitions here: https://world.openfoodfacts.org/nova):<br>➢ Processed foods, such as bottled vegetables, canned fish, fruits in syrup, cheeses and freshly made breads, are made essentially by adding salt, oil, sugar or other substances from Group 2 (processed culinary ingredients) to Group 1 (unprocessed or minimally processed) foods.<br>➢ Ultra-processed foods, such as soft drinks, sweet or savoury packaged snacks, reconstituted meat products and pre-prepared frozen dishes, are not modified foods but formulations made mostly or entirely from substances derived from foods and additives, with little if any intact Group 1 (unprocessed or minimally processed foods) food. The overall purpose of ultra-processing is to create

	branded, convenient (durable, ready to consume), attractive (hyper-palatable) and highly profitable (low-cost ingredients) food products designed to displace all other food groups.

**DOMAIN 1 - FOOD COMPOSITION - Food composition targets/standards/restrictions for processed foods: There are government systems implemented to ensure that, where practicable, processed foods minimise the energy density and the nutrients of concern (salt, saturated fat, trans fat, added sugar)**	
**Indicator definitions** - please take these into account in the rating process	**SCOPE (FOR INFORMATION ONLY)**<br>**This colum provides the type of evidence collected for this indicator (as well as the evidence that will not be taken into account).**
**INDICATOR (COMP) 2: Food composition targets/standards/restrictions have been established by the government for the content of the nutrients of concern (trans fats, added sugars, salt, saturated fat) in meals sold from food service outlets, in particular for those food groups that are major contributors to population intakes of those nutrients of concern.**<br><br>*(Trans fat has been excluded at national level as it falls under EU regulation)*	➢ Meals sold at food service outlets include foods sold at quick service restaurants, dine-in restaurants and take-away outlets, coffee, bakery and snack food outlets (both fixed outlets and mobile food vendors). This also includes foods from catering operations and delivery meals.<br>➢ Includes legislated bans on nutrients of concern<br>➢ Includes mandatory or voluntary targets, standards (i.e. reduce by X%, maximum mg/g per 100g or per serving)<br>➢ Excludes legislated restrictions related to other ingredients (e.g. additives)<br>➢ Excludes mandatory out-of-home meal composition regulations related to vitamins and micronutrients, e.g. folic acid or iodine fortification<br>➢ Excludes food consumption standards/targets for fibre, healthy ingredients like fruits and vegetables<br>➢ Excludes general guidelines advising food service outlets to reduce nutrients of concern<br>➢ Excludes the provision of resources or expertise to support food service outlets with reformulation

**DOMAIN 2 - FOOD LABELLING - There is a regulatory system implemented by the government for consumer-oriented labelling on food packaging and menu boards in restaurants to enable consumers to easily make informed food choices and to prevent misleading claims.**	
**Indicator definitions** - please take these into account in the rating process	**SCOPE (FOR INFORMATION ONLY)**<br>**This colum provides the type evidence collected for this indicator (as well as the evidence that will not be taken into account).**
**INDICATOR (LABEL) 1: Ingredient lists and nutrient declarations in line with Codex recommendations are present on the labels of all packaged foods.**<br><br>*(Label 1 will not be included for benchmarking at national level as it's being addressed at EU level)*	➢ Includes packaged foods manufactured in Country X or manufactured elsewhere and imported to Country X for sale.<br>➢ Nutrient declaration means a standardized statement or listing of the nutrient content of a food<br>➢ Excludes health and nutrition claims (see 'LABEL 2')<br>➢ Includes trans fats and added sugar which are not part of the standard seven elements generally part of mandatory nutrient declarations (energy, total fat, saturated fat, trans fat, carbohydrates, sugar, protein, sodium)
**INDICATOR (LABEL) 2: Evidence-based regulations are in place for approving and/or reviewing claims on foods, so that consumers are protected against unsubstantiated and misleading nutrition and health claims.**<br><br>*9Label 2 will not be included for benchmarking at national level as it's being addressed at EU level)*	➢ Nutrition claims include references to the nutritional content on food (e.g. low in fat).<br>➢ Health claims include function claims, such as 'calcium strengthens bones') and disease risk reduction and therapeutic claims, such as 'A healthy diet rich in a variety of vegetables and fruit may help reduce the risk of some types of cancer') claims that relate to the relationship between a diet, a food or a property of a food and a health effect.<br>➢ Includes the use of a nutrient profiling system to classify food products into permitted/not permitted to carry health claims and/or nutrition claims<br>➢ 'Evidence-based' refers to regulations that are based on an extensive review of up-to-date research and expert input or a validated nutrient profiling model to inform decision-making about nutrition or health claims
**INDICATOR (LABEL) 3: One or more interpretive, evidence-informed front-of-pack supplementary nutrition information system(s) endorsed by the Government, which readily allow consumers to assess a product's healthiness, is/are applied to all packaged foods (examples are the Nutriscore and traffic lights).**	➢ Nutrition information systems include traffic light labelling (overall or for specific nutrients); Warning labels; Nutriscore; star or points rating; percent daily intake.<br>➢ Keyhole and Finish heart symbol are not considered FOP labelling systems (but rather claims).<br>➢ 'Evidence-informed' refers to systems that utilise robust criteria (based on an extensive review of up-to-date research and expert input) or a validated nutrient profiling model to inform decision-making about the product's healthiness
**INDICATOR (LABEL) 4: A simple and clearly-visible system of labelling the menu boards of all quick service restaurants (i.e. fast food chains) is applied by the**	➢ Quick service restaurants: In the ' (Country name)' context this definition includes fast food chains as well as gas stations, kiosks, coffee, bakery and snack

**government, which allows consumers to interpret the nutrient quality and energy content of foods and meals on sale.**	food chains. It may also include supermarkets where ready-to-eat foods are sold.<br>➢ Definition Euromonitor: Fast food outlets offer limited menus that are prepared quickly. Customers order, pay and pick up their order from a counter. Outlets tend to specialize in one or two main entrees such as hamburgers, pizza, ice cream, or chicken, but they usually also provide salads, drinks, dessert etc. Food preparation is generally simple and involves one or two steps, allowing for kitchen staffs generally consisting of younger, unskilled workers. Other key characteristics include: • A standardised and restricted menu; • Food for immediate consumption; • Tight individual portion control on all ingredients and on the finished product; • Individual packaging of each item; • Counter service; • A seating area, or close access to a shared seating area, such as in a shopping centre food court • For chained fast food, chained and franchised operations which operate under a uniform fascia and corporate identity. • Take out is generally present, as is drive-through in some markets.<br>➢ Labelling systems: Includes any point-of-sale (POS) nutrition information such as total kilojoules; percent daily intake; traffic light labelling; star rating, or specific amounts of nutrients of concern, salt warning labels.<br>➢ Includes endorsement schemes (e.g., accredited healthy choice symbol) on approved menu items

DOMAIN 3 - FOOD PROMOTION - There is a comprehensive policy implemented by the government to reduce the impact (exposure and power) of promotion of unhealthy foods to children across all media.

- **Exposure of food marketing concerns the reach and frequency of a marketing message. This is dependent upon the media or channels which are used to market foods.**
- **The power of food marketing concerns the creative content of the marketing message. For example, using cartoons or celebrities enhances the power (or persuasiveness) of a marketing message because such strategies are attractive to children.**

**Indicator definitions** - please take these into account in the rating process	**SCOPE (FOR INFORMATION ONLY)**<br>This colum provides the type evidence collected for this indicator (as well as the evidence that will not be taken into account).
**INDICATOR (PROMO) 1: Effective policies are implemented by the government to restrict exposure and power of promotion of unhealthy foods to children including adolescents through broadcast media (TV, radio).**	➢ Includes mandatory policy (i.e. legislation or regulations) or voluntary standards, codes, guidelines set by government or by industry where the government plays a role in development, monitoring, enforcement or resolving complaints (i.e. co-regulation).<br>➢ Includes free-to-air and subscription television and radio only (see PROMO2, PROMO3 and PROMO5 for other forms of media).<br>➢ Effective means that the policies are likely to reduce overall exposure of children, including adolescents to unhealthy food advertising over the day.
**INDICATOR (PROMO) 2:**<br>**Effective policies are implemented by the government to restrict exposure and power of promotion of unhealthy foods to children including adolescents through online and social media.**	➢ Includes online media (e.g. social media, branded education websites, online games, competitions and apps)<br>➢ Where the promotion is specifically through other non-broadcast media than online and social media, this should be captured in 'PROMO3 and PROMO5'.<br>➢ Where the promotion is specifically in a children's setting, this should be captured in 'PROMO4'.<br>➢ Effective means that the policies are likely to reduce overall exposure of children, including adolescents to unhealthy food advertising over the day.
**INDICATOR (PROMO) 3: Effective policies are implemented by the government to restrict exposure and power of promotion of unhealthy foods to children including adolescents through non-broadcast media other than packaging and online/social media.**	➢ Non-broadcast media promotion includes: print (e.g. children's magazines), on/around public transport (e.g. signage, posters and billboards), cinema advertising, product placement and brand integration (e.g. in television shows and movies), direct marketing (e.g. provision of show bags, samples or flyers), or point-of-sale (POS) displays<br>➢ Non-broadcast media is excluding the media covered through other indicators like online and social media (PROMO2) and packaging (PROMO5)<br>➢ Where the promotion is specifically in a children's setting, this should be captured in 'PROMO4'

	➢ Effective means that the policies are likely to reduce overall exposure of children, including adolescents to unhealthy food advertising over the day.
**INDICATOR (PROMO) 4: Effective policies are implemented by the government to ensure that unhealthy foods are not commercially promoted to children including adolescents in settings where children gather (e.g. preschools, schools, sport and cultural events).**	➢ Children's settings include: areas in and around schools, preschools/ kindergartens, daycare centres, children's health services (including primary care, maternal and child health or tertiary settings), sport, recreation and play areas/ venues/ facilities and cultural/community events where children are commonly present.<br>➢ Includes restrictions on marketing in government-owned or managed facilities/venues (including within the service contracts where management is outsourced)<br>➢ Includes restriction on unhealthy food sponsorship in sport (e.g. junior sport, sporting events, venues)<br>➢ Effective means that the policies are likely to reduce overall exposure of children, including adolescents to unhealthy food advertising over the day.
**INDICATOR (PROMO) 5: Effective policies are implemented by the government to ensure that unhealthy foods are not commercially promoted to children, including adolescents on food packages**	➢ Includes product design and packaging (e.g. use of celebrities or cartoons, competitions and give-aways)<br>➢ Where the promotion is specifically in a children's setting, this should be captured in 'PROMO4'<br>➢ Effective means that the policies are likely to reduce overall exposure of children, including adolescents to unhealthy food advertising over the day.

DOMAIN 4- FOOD PRICES - Food pricing policies (e.g., taxes and subsidies) are aligned with health outcomes by helping to make the healthy eating choices the easier, cheaper choices	
**Indicator definitions** - please take these into account in the rating process	**SCOPE (FOR INFORMATION ONLY)**<br>This colum provides the type evidence collected for this indicator (as well as the evidence that will not be taken into account).
**INDICATOR (PRICES) 1: Taxes or levies on healthy foods are minimised to encourage healthy food choices (e.g. low or no sales tax, excise, value-added or import duties on fruit and vegetables)**	➢ Includes exemptions from excise tax, ad valorem tax or import duty.<br>➢ Includes differential application of excise tax, ad valorem tax or import duty.<br>➢ Excludes subsidies (see 'PRICES3') or food purchasing welfare support (see 'PRICES4')
**INDICATOR (PRICES) 2: Taxes or levies on unhealthy foods (e.g. sugar-sweetened beverages, foods high in nutrients of concern) are in place and increase the retail prices of these foods by at least 10% to discourage unhealthy food choices, and these taxes are reinvested to improve population health**	➢ Includes differential application of excise tax, ad valorem tax or import duty on high calorie foods or foods that are high in nutrients of concern
**INDICATOR (PRICES) 3: The intent of existing subsidies on foods, including infrastructure funding support (e.g. research and development, supporting markets or transport systems), is to favour healthy rather than unhealthy foods.**	➢ Includes agricultural input subsidies, such as free or subsidised costs for water, fertiliser, seeds, electricity or transport (e.g., freight) where those subsidies specifically target healthy foods<br>➢ Includes programs that ensure that farmers receive a certain price for their produce to encourage increased food production or business viability<br>➢ Includes grants or funding support for food producers (i.e. farmers, food manufacturers) to encourage innovation via research and development where that funding scheme specifically targets healthy food<br>➢ Includes funding support for wholesale market systems that support the supply of healthy foods<br>➢ Includes population level food subsidies at the consumer end (e.g. subsidising staples such as rice or bread)<br>➢ Excludes incentives for the establishment of, or ongoing support for, retail outlets (including greengrocers, farmers' markets, food co-ops, etc. See 'RETAIL2').<br>➢ Excludes subsidised training, courses or other forms of education for food producers - Excludes the redistribution of excess or second grade produce<br>➢ Excludes food subsidies related to welfare support (see 'PRICES4')<br>➢ Should be in line with population nutrition goals related to the prevention of obesity and diet-related NCDs (e.g., reducing intake of nutrients of concern, and should not related to micronutrient deficiencies)

**INDICATOR (PRICES) 4: The government ensures that food-related income support programs are for healthy foods**	➢ Includes programs such as 'food stamps' or other schemes where individuals can utilise government-administered subsidies, vouchers, tokens or discounts in retail settings for specific food purchasing.<br>➢ Excludes general programs that seek to address food insecurity such as government support for, or partnerships with, organisations that provide free or subsidised meals (including school breakfast programs) or food parcels or redistribute second grade produce for this purpose.<br>➢ Excludes food subsidies at the consumer end (e.g. subsidising staples at a population level – see 'PRICES3')

DOMAIN 5 - FOOD PROVISON - The government ensures that there are healthy food service policies implemented in government-funded settings to ensure that food provision encourages healthy food choices, and the government actively encourages and supports private companies to implement similar	
**Indicator definitions** - please take these into account in the rating process	**SCOPE (FOR INFORMATION ONLY)**<br>This colum provides the type evidence collected for this indicator (as well as the evidence that will not be taken into account).
**INDICATOR (PROV) 1: The government ensures that there are clear, consistent policies (including nutrition standards) implemented in schools and early childhood education services for food service activities (canteens, food at events, fundraising, promotions, vending machines etc.) to provide and promote healthy food choices.**	➢ Includes early childhood education and care services (0-5 years).<br>➢ Schools include government and non-government primary and secondary schools (up to age 18 years)<br>➢ Includes policies and nutrition standards to provide and promote healthy food choices or to limit or restrict the provision or promotion of unhealthy food choices<br>➢ Includes policies that relate to school meals programs, where the program is partly or fully funded, managed or overseen by the government<br>➢ Excludes programmes in schools that are targeted to children of low socioeconomic groups only (as these would be covered under PRICES4)
**INDICATOR (PROV) 2: The government ensures that there are clear, consistent policies in other public sector settings for food service activities (canteens, food at events, fundraising, promotions, vending machines, etc.) to provide and promote healthy food choices.**	➢ Public sector settings include: - Government-funded or managed services where the government is responsible for the provision of food, including public hospitals and other in-patient health services (acute and sub-acute, including mental health services), residential care homes, aged and disability care settings, custodial care facilities, prisons and home/community care services - Government-owned, funded or managed services where the general public purchase foods including health services, parks, sporting and leisure facilities, community events etc. - Public sector workplaces<br>➢ Includes private businesses that are under contract by the government to provide food<br>➢ Excludes 'public settings' such as train stations, venues, facilities or events that are not funded or managed by the government (see 'RETAIL4')<br>➢ Excludes school and early childhood settings (see 'PROV1')<br>➢ Includes policies and nutrition standards to provide and promote healthy food choices or to limit or restrict the provision or promotion of unhealthy food choices<br>➢ Includes the strategic placement of foods and beverages in cabinets, fridges, on shelves or near the cashier

	➢ Includes the use of signage to highlight healthy options or endorsements (such as traffic lights or a recognised healthy symbol)<br>➢ Includes modifying ingredients to make foods and drinks healthier, or changing the menu to offer healthier options<br>➢ Excludes public procurement standards (see 'PROV3')
**INDICATOR (PROV) 3: The government ensures that there are clear, consistent public procurement standards in public sector settings for food service activities to provide and promote healthy food choices.**	➢ Includes standards for the public sector which encourage the procurement of healthy foods<br>➢ Includes standards for the public sector which discourage the procurement of unhealthy foods<br>➢ Includes public sector settings as defined in PROV 1 and PROV 2
**INDICATOR (PROV) 4: The Government ensures that there are good support and training systems to help schools and other public sector organisations and their caterers meet the healthy food service policies and guidelines**	➢ Includes support for early childhood education services as defined in 'PROV1'<br>➢ Public sector organisations include settings defined in 'PROV2'<br>➢ Support and training systems include guidelines, toolkits, templates (e.g. policy/guidelines or contracts), recipes and menu planning tools, expert advice, menu and product assessments, online training modules, cook/caterer/other food service staff information and training workshops or courses
**INDICATOR (PROV) 5: The Government actively encourages and supports private companies to provide and promote healthy foods and meals in their workplaces**	➢ For the purpose of this indicator, 'private companies' includes for-profit companies and extends to non-government organisations (NGOs) including not-for-profit/charitable organisations, community-controlled organisations, etc.<br>➢ Includes healthy catering policies, fundraising, events - Includes support and training systems including guidelines, toolkits, templates (e.g. policy/guidelines or contracts), recipes and menu planning tools, expert advice, menu and product assessments, online training modules, cook/caterer/other food service staff information and training workshops or courses (where relevant to the provision of food in a workplace)<br>➢ Excludes the provision or promotion of food to people not employed by that organisation (e.g. visitors or customers)<br>➢ Excludes support for organisations to provide staff education on healthy foods

**DOMAIN 6 - FOOD IN RETAIL - The government has the power to implement policies and programs to support the availability of healthy foods and limit the availability of unhealthy foods in communities (outlet density and locations) and in-store (product placement)**

**Indicator definitions** - please take these into account in the rating process	**SCOPE (FOR INFORMATION ONLY)**<br>This colum provides the type evidence collected for this indicator (as well as the evidence that will not be taken into account).
**INDICATOR (RETAIL) 1: Zoning laws and policies are implemented to place limits on the density or placement of quick serve restaurants or other outlets selling mainly unhealthy foods in communities and/or access to these outlets (e.g. opening hours).**	➢ Includes the consideration of public health in State/Territory Planning Acts that guide the policies, priorities and objectives to be implemented at the local government level through their planning schemes<br>➢ Includes the consideration of public health in State/Territory subordinate planning instruments and policies<br>➢ Includes a State/Territory guideline that sets the policy objective of considering public health when reviewing and approving fast food planning applications.<br>➢ Includes limitations to access of unhealthy food outlets (i.e. opening hours)<br>➢ Excludes laws, policies or actions of local governments
**INDICATOR (RETAIL) 2: Zoning laws and policies are implemented to encourage the availability of outlets selling fresh fruit and vegetables and/or access to these outlets (e.g. opening hours, frequency i.e. for markets) .**	➢ Outlets include supermarkets, produce markets, farmers' markets, greengrocers, food cooperatives<br>➢ Includes fixed or mobile outlets<br>➢ Excludes community gardens, edible urban or backyard gardens (usually regulated by local governments)<br>➢ Includes policies that support local governments to reduce license or permit requirements or fees to encourage the establishment of such outlets<br>➢ Includes State/Territory policies to streamline and standardise planning approval processes or reduce regulatory burdens for these outlets<br>➢ Includes actions to improve access to healthy food outlets (i.e. opening hours; frequency i.e. for markets)<br>➢ Includes the provision of financial grants or subsidies to outlets
**INDICATOR 3 (RETAIL 3): The Government ensures existing support systems are in place to encourage food stores to promote the in-store availability of healthy foods and to limit the in-store availability of unhealthy foods**	➢ Food stores include supermarkets, convenience stores (including 'general stores' or 'milk bars'), greengrocers and other speciality food retail outlets<br>➢ Support systems include guidelines, resources or expert support<br>➢ Includes all settings with food retail stores, including but not exclusive to; train stations, venues, facilities or events frequented by the public etc.<br>➢ Excludes settings owned or managed by the government (see 'PROV2' and 'PROV4')

	- Includes the strategic placement of foods and beverages in cabinets, fridges, on shelves or near the cashier - Includes the use of signage to highlight healthy options or endorsements (such as traffic lights or a recognised healthy symbol)<br>- Includes offering healthier food and drink products, or changing the menu or store layout to offer more healthy options<br>- Includes decreasing the offer of unhealthy food and drink products<br>- Excludes reformulation and labelling in relation to nutrients of concern (COMP1; LABEL4)
**INDICATOR (RETAIL 4): The government ensures existing support systems are in place to encourage the promotion and availability of healthy foods in food service outlets and to discourage the promotion and availability of unhealthy foods in food service outlets**	- Food service outlets include for-profit quick service restaurants, eat-in or take-away restaurants, pubs, clubs<br>- Support systems include guidelines, resources or expert support<br>- Includes settings such as train stations, venues, facilities or events frequented by the public<br>- Excludes settings owned or managed by the government (see ‘PROV2’ and ‘PROV4’)<br>- Includes the strategic placement of foods and beverages in cabinets, fridges, on shelves or near the cashier - Includes the use of signage to highlight healthy options or endorsements (such as traffic lights or a recognised healthy symbol)<br>- Includes modifying ingredients to make foods and drinks more healthy, or changing the menu to offer more healthy options<br>- Excludes reformulation and labelling in relation to nutrients of concern (COMP2; LABEL4)

DOMAIN 7 - FOOD TRADE AND INVESTMENT - **The government ensures that trade and investment agreements protect food sovereignty, favour healthy food environments, are linked with domestic health and agricultural policies in ways that are consistent with health objectives, and do not promote unhealthy food environments.**	
**Indicator definitions** - please take these into account in the rating process	**SCOPE (FOR INFORMATION ONLY)**<br>This colum provides the type evidence collected for this indicator (as well as the evidence that will not be taken into account).
**INDICATOR (TRADE) 1: The Government undertakes risk impact assessments before and during the negotiation of trade and investment agreements, to identify, evaluate and minimize the direct and indirect negative impacts of such agreements on population nutrition and health**	➢ Includes policies or procedures that guide the undertaking of risk impact assessments before or during negotiation to assess risks and benefits in relation to public health and population nutrition<br>➢ Includes policies or procedures that guide the evaluation of trade and investment agreements after an agreement is finalised to monitor the impact for the purpose of informing future negotiations or reviews<br>➢ Includes policies or procedures that guide public consultation procedures before and during negotiations<br>➢ Any trade or economic agreements still considered active
**INDICATOR (TRADE) 2: The government adopts measures to manage investment and protect their regulatory capacity with respect to public health nutrition**	➢ Includes provisions in trade or economic agreements that protect the capacity of government to implement domestic policy in relation to food environments. This includes protections with respect to tariffs, non-tariff measures (such as quotas, regulations, standards, testing, certification, licensing procedures) and measures related to foreign direct investment<br>➢ Binding commitments made under Trade and Investment Agreements (TIA's) can constrain the way countries can regulate goods, services, and investments to promote public interests (including public health) in a way that is upstream from domestic policy processes.

DOMAIN 8 - LEADERSHIP - The political leadership ensures that there is strong support for the vision, planning, communication, implementation and evaluation of policies and actions to create healthy food environments, improve population nutrition, and reduce diet-related inequalities	
**Indicator definitions** - please take these into account in the rating process	**SCOPE (FOR INFORMATION ONLY)**<br>This colum provides the type evidence collected for this indicator (as well as the evidence that will not be taken into account).
**INDICATOR (LEAD 1): There is strong, visible, political support (at the head of government or state/ ministerial level) for improving food environments, population nutrition, diet related NCDs and their related inequalities"**	➢ Visible support includes statements of intent, election commitments, budget commitments, establishing priorities and targets, demonstration of support in the media, other actions that demonstrate support for new or strengthened policy<br>➢ Documents that contain evidence of strong political support include media releases, speeches, pre-election policy papers, introduction of a bill, State-level strategic plans with targets or key performance indicators
**INDICATOR (LEAD 2): Clear population intake targets have been established by the government for the nutrients of concern and / or relevant food groups to meet WHO and national recommended dietary intake levels**	Includes targets which specify population intakes according to average reductions in percentage or volume (e.g. mg/g) for salt/sodium, saturated fat, trans fats or added or free sugars*<br>➢ Typically requires the government to establish clear dietary guidelines on the maximum daily intake of nutrients of concern<br>➢ **Free sugar is defined as is the sugar no longer in its naturally-occurring state (i.e., no longer in whole fruits, vegetables, unsweetened dairy, and grains) and can be consumed as is or incorporated into other foods. Examples include table sugar, syrup, honey, fruit juice and nectars. Added sugar is defined as the free sugar that has been added to foods, however regulatory definitions vary widely under different jurisdictions, some of which are currently under review. These differ from naturally occurring sugars, defined as the sugar found naturally within whole foods (i.e., within whole fruits, vegetables, dairy, and some grains).
**INDICATOR (LEAD 3): Clear, interpretive, evidenced-informed food based dietary guidelines have been established and implemented.**	➢ Food-based dietary guidelines should be for both genders and key age groups including infants and pregnant women<br>➢ Evidence-informed includes extensive review of up-to-date research and mechanisms to seek expert input<br>➢ Evidence includes ways the FBDG have been used to develop/implement policies to improve diets
**INDICATOR (LEAD 4): There is a comprehensive, transparent, up-to-date implementation plan linked to national needs and priorities, to improve food environments , reduce the intake of the nutrients of**	➢ Includes documented plans with specific actions and interventions (i.e. policies, programs, partnerships)

**concern to meet WHO and national recommended dietary intake levels, and reduce diet-related NCDs**	➢ Plans should be current (i.e. maintain endorsement by the current government and/or are being reported against)<br>➢ Plans should refer to actions to improve food environments (as defined in the policy domains above) and should include both policy and program strategies<br>➢ Excludes overarching frameworks that provide general guidance and direction<br>➢ Includes priority policy and program strategies, social media marketing for public awareness and threat of legislation for voluntary approaches.
**INDICATOR (LEAD 5): Government priorities have been established to reduce inequalities or protect vulnerable populations in relation to diet, nutrition, obesity and NCDs**	➢ Frameworks, strategies or implementation plans specify aims, objectives or targets to reduce inequalities including taking a preventive approach that addresses the social and environmental determinants of health<br>➢ Frameworks, strategies or implementation plans identify vulnerable populations or priority groups<br>➢ Implementation plans specify policies or programs that aim to reduce inequalities for specific population groups<br>➢ Excludes priorities to reduce inequalities in secondary or tertiary prevention

DOMAIN 9 - GOVERNANCE: Governments have structures in place to ensure transparency and accountability, and encourage broad community participation and inclusion when formulating and implementing policies and actions to create healthy food environments, improve population nutrition, and reduce diet-related inequalities	
**Indicator definitions** - please take these into account in the rating process	**SCOPE (FOR INFORMATION ONLY)**<br>This colum provides the type evidence collected for this indicator (as well as the evidence that will not be taken into account).
**INDICATOR (GOVER)1: There are procedures in place to restrict commercial influences on the development of policies related to food environments where they have conflicts of interest with improving population nutrition. for example: restricting lobbying influences.**	➢ Includes government policies, guidelines, codes of conduct or other mechanisms to guide actions and decision-making by government employees, for example conflict of interest declaration procedures<br>➢ Includes procedures to manage partnerships with private companies or peak bodies representing industries that are consulted for the purpose of developing policy, for examples committee procedural guidelines or terms of reference.<br>➢ Includes publicly available, up to-to-date registers of lobbyist and/ or their activities
**INDICATOR (GOVER) 2: Policies and procedures are implemented for using evidence in the development of food and nutrition policies**	➢ Includes policies, procedures or guidelines to support government employees in the use of evidence for policy development including best practice evidence review methodology (including types and strength of evidence needed) and policy implementation in the absence of strong evidence (where the potential risk or harms of inaction are great).<br>➢ Includes policies, procedures or guidelines that stipulate the requirements for the establishment of a scientific or expert committee to inform policy development.<br>➢ Includes the use of evidence-based models, algorithms and tools to guide policy development or within policy to guide implementation (e.g. nutrient profiling model)<br>➢ Includes government resourcing of evidence and research by specific units, either within or across government departments
**INDICATOR (GOVER) 3: Policies and procedures are implemented for ensuring transparency in the development of food and nutrition policies**	➢ Includes policies or procedures that guide the use of consultation in the development of food policy<br>➢ Includes policies or procedures to guide the online publishing of private sector and civil society submissions to government around the development of policy and subsequent government response to these.<br>➢ Includes policies or procedures to guide the online publishing of scoping papers, draft and final policies<br>➢ Include policies or procedures to guide public communications around all policies put forward but not progressed

**INDICATOR (GOVER) 4: The government ensures public access to comprehensive nutrition information and key documents (e.g. budget documents, annual performance reviews and health indicators) for the public**	➢ Includes policies and procedures to guide the timely, online publishing of government budgets, performance reviews, audits, evaluation reports or the findings of other reviews or inquiries<br>➢ Includes 'freedom of information' legislation and related processes to enable the public access to government information on request, with minimal restrictions and exemptions<br>➢ Includes policies or procedures to guide the timely, online publishing of population health data captured / owned by government

DOMAIN 10 - MONITORING AND INTELLIGENCE - The government's monitoring and intelligence systems (surveillance, evaluation, research and reporting) are comprehensive and regular enough to assess the status of food environments, population nutrition and diet-related NCDs and their inequalities, and to measure progress on achieving the goals of nutrition and health plans	
**Indicator definitions** - please take these into account in the rating process	**SCOPE (FOR INFORMATION ONLY)**<br>This colum provides the type evidence collected for this indicator (as well as the evidence that will not be taken into account).
**INDICATOR (MONIT 1): Monitoring systems, implemented by the government, are in place to regularly monitor food environments(especially for food composition for nutrients of concern, food promotion to children, and nutritional quality of food in schools and other public sector settings), against codes / guidelines / standards / targets**	➢ Includes monitoring systems funded fully or in part by government that are managed by an academic institution or other organisation<br>➢ Includes regular monitoring and review of the impact of policies implemented by the government on food environments (as relevant to the individual State / Territory, and described in the policy domains above), in particular:<br>1. Monitoring of compliance with voluntary food composition standards related to nutrients of concern in out-of-home meals (as defined in the 'Food composition' domain)<br>2. Monitoring of compliance with food labelling regulations (as defined in the 'Food labelling' domain above)<br>3. Monitoring of unhealthy food promoted to children via broadcast and non-broadcast media and in children's settings (as defined in the 'Food promotion' domain above)<br>4. Monitoring of compliance with food provision policies in schools, early childhood services and public sector settings (as defined in the 'Food provision' domain above)
**INDICATOR (MONIT 2): There is regular monitoring of adult and childhood nutrition status and population intakes against specified intake targets or recommended daily intake levels**	➢ Includes monitoring of adult and child intake in line with X Countries Food Guide and dietary recommendations<br>➢ Includes monitoring of adult and child intake of nutrients of concern and noncore/discretionary foods including sugar-sweetened beverages (even if there are no clear intake targets for all of these)<br>➢ 'Regular' is considered to be every five years or more frequently
**INDICATOR (MONIT 3): There is regular monitoring of adult and childhood overweight and obesity prevalence using anthropometric measurements**	➢ Anthropometric measurements include height, weight and waist circumference<br>➢ 'Regular' is considered to be every five years or more frequently
**INDICATOR (MONIT4): There is regular monitoring of the prevalence of NCD metabolic risk factors and occurrence**	➢ Diet-related NCD risk factors and NCDs include, amongst others, hypertension, hypercholesterolemia, Type 2 Diabetes,

**rates (e.g. prevalence, incidence, mortality) for the main diet-related NCDs**	cardiovascular disease (including ischaemic heart disease, cerebrovascular disease and other diseases of the vessels), diet-related cancers<br>➢ disease and other diseases of the vessels), diet-related cancers<br>➢ May be collected through a variety of mechanisms such as population surveys or a notifiable diseases surveillance system
**INDICATOR (MONIT 5): Major programs and policies are regularly evaluated to assess their effectiveness and contributions to achieving the goals of the nutrition and health plans**	➢ Includes any policies, guidelines, frameworks or tools that are used to determine the depth and type (method and reporting) of evaluation required<br>➢ Includes a comprehensive evaluation framework and plan that aligns with the key preventive health or nutrition implementation plan<br>➢ The definition of a major programs and policies is to be defined by the relevant government department<br>➢ Evaluation should be in addition to routine monitoring of progress against a project plan<br>➢ or program logic
**INDICATOR (MONIT 6) Progress towards reducing health inequalities or health impacts in vulnerable populations and social and economic determinants of health are regularly monitored.**	➢ Monitoring of overweight and obesity and main diet-related NCDs includes stratification or analysis of population groups where there are the greatest health inequalities including Indigenous peoples and socio-economic strata<br>➢ Includes reporting against targets or key performance indicators related to health inequalities

**DOMAIN 11 - FUNDING AND RESOURCES - Sufficient funding is invested in 'Population Nutrition Promotion' (estimated from the investments in population promotion of healthy eating and healthy food environments for the prevention of obesity and diet-related NCDs, excluding all one-on-one promotion (primary-care, antenatal services, maternal and child nursing services etc.), food safety, micronutrient deficiencies (e.g. folate fortification and undernutrition) to create healthy food environments, improved population nutrition, reductions in obesity, diet-related NCDs and their related inequalities.**	
**Indicator definitions** - please take these into account in the rating process	**SCOPE (FOR INFORMATION ONLY)**<br>This colum provides the type evidence collected for this indicator (as well as the evidence that will not be taken into account).
**INDICATOR (FUND) 1: The 'population nutrition' budget, as a proportion of total health spending and/or in relation to the diet-related NCD burden sufficiently contributes to reducing diet-related NCD's**<br><br>**(This indicator isn't being included in the rating process)**	➢ 'Population nutrition' includes promotion of healthy eating, and policies and programs that support healthy food environments for the prevention of obesity and diet-related NCDs<br>➢ The definition excludes all one-on-one and group-based promotion (primary care, antenatal services, maternal and child nursing services etc.), food safety, micronutrient deficiencies (e.g. folic acid fortification) and undernutrition<br>➢ Includes estimates for the budget allocated to the unit within the Department of Health that has primary responsibility for population nutrition.<br>➢ The workforce comprises anyone whose primary role relates to population nutrition and who is employed full time, part time or casually by the Department of Health or contracted by the Department of Health to perform a population nutrition-related role (including consultants or funding of a position in another government or nongovernment agency). The number of full time equivalent persons in the workforce will be reported in 'FUND4<br>➢ Excludes budget items related to physical activity promotion.
**INDICATOR (FUND) 2: Government funded research is targeted for improving food environments, reducing obesity, NCDs and their related inequalities**	➢ Includes the clear identification of research priorities related to improving food environments, reducing obesity, NCDs and their related inequalities in health or medical research strategies or frameworks<br>➢ Includes identifying research projects conducted or commissioned by the government specifically targeting food environments, prevention of obesity or NCDs (excluding secondary or tertiary prevention)<br>➢ It is limited to research projects committed to or conducted within the last 12 months<br>➢ Excludes research grants administered by the government (including statutory agencies) to a research group where the allocation of a pool of

	funding was determined by an independent review panel<br>➢ Excludes evaluation of interventions (this is explored in 'MONIT5' and should be part of an overall program budget)
**INDICATOR (FUND) 3: There is a statutory health promotion agency in place that includes an objective to improve population nutrition with a secure funding stream**	➢ Agency was established through legislation<br>➢ Includes objective to improve population nutrition in relevant legislation, strategic plans or on agency website<br>➢ Secure funding stream involves the use of a hypothecated tax or other secure source

DOMAIN 12 - PLATFORMS AND INTERACTION: There are coordination platforms and opportunities for synergies across government departments, levels of government, and other sectors (NGOs, private sector, and academia) such that policies and actions in food and nutrition are coherent, efficient and effective in improving food environments, population nutrition, diet-related NCDs and their related inequalities	
**Indicator definitions** - please take these into account in the rating process	**SCOPE (FOR INFORMATION ONLY)**<br>This colum provides the type evidence collected for this indicator (as well as the evidence that will not be taken into account).
**INDICATOR (PLAT) 1: There are robust coordination mechanisms across departments and levels of government (national, state and local) to ensure policy coherence, alignment, and integration of food, obesity and diet-related NCD prevention policies across governments.**	➢ Includes cross-government or cross-departmental governance structures, committees or working groups (at multiple levels of seniority), agreements, memoranda of understanding, etc<br>➢ Includes cross-government or cross-departmental shared priorities, targets or objectives<br>➢ Includes strategic plans or frameworks that map the integration and alignment of multiple policies or programs across governments and across departments<br>➢ Includes cross-government or cross-departmental collaborative planning, implementation or reporting processes, consultation processes for the development of new policy or review of existing policy
**INDICATOR (PLAT) 2: There are formal platforms (with clearly defined mandates, roles and structures) for regular interactions between government and the commercial food sector on the implementation of healthy food policies and other related strategies.**	➢ The commercial food sector includes food production, food technology, manufacturing and processing, marketing, distribution, retail and food service, etc. For the purpose of this indicator, this extends to commercial non-food sectors (e.g. advertising and media, sports organisations, land/housing developers, private childcare, education and training institutes) that are indirectly related to food<br>➢ Includes established groups, forums or committees active within the last 12 months for the purpose of information sharing, collaboration, seeking advice on healthy food policies<br>➢ Includes platforms to support, manage or monitor private sector pledges, commitment or agreements<br>➢ Includes platforms for open consultation<br>➢ Includes platforms for the government to provide resources or expert support to the commercial food sector to implement policy<br>➢ Excludes joint partnerships on projects or co-funding schemes<br>➢ Excludes platforms to engage with industry in relation to development of policies.<br>➢ Excludes initiatives covered by 'RETAIL3' and 'RETAIL4'

**INDICATOR (PLAT) 3: There are formal platforms (with clearly defined mandates, roles and structures) for regular interactions between government and civil society on the development, implementation and evaluation of healthy food policies and other related strategies.**	➢ Civil society includes community groups and consumer representatives, NGOs, academia, professional associations, etc.<br>➢ Includes established groups, forums or committees active within the last 12 months for the purpose of information sharing, collaboration, seeking advice<br>➢ Includes platforms for consultation on proposed plans, policy or public inquiries<br>➢ Excludes policies or procedures that guide consultation in the development of food policy (see 'GOVER3')
**INDICATOR (PLAT) 4: The governments work with a system-based approach with (local and national) organisations/partners/groups to improve the healthiness of food environments at a national level.**	➢ Systems-based approaches may include policies within other domains of health<br>➢ May include a social-determinants of health approach<br>➢ May bring together multiple departments or ministries to approach health<br>➢ Includes multiple levels of government<br>➢ Aim of a systems-based approach is:<br>o resourcing and supporting a dedicated, reflective and skilled workforce at a state and/or local level to engage, activate and influence at multiple levels of the system to combat obesity and chronic disease<br>o building relationships with prevention partners across the system, and across sectors and industries, to strengthen positive health outcomes on multiple fronts<br>o capturing and feeding back knowledge and data on progress, impact and effectiveness and calling for new types of research, policy and practice collaborations<br>o allocating resources based on best possible investment to effect change and population need, seeding long term change by resourcing local governments to lead action towards public health<br>o building leadership for sustained prevention across the system to drive effective and long lasting change.

DOMAIN 13 - HEALTH IN ALL POLICIES - Processes are in place to ensure policy coherence and alignment, and that population health impacts are explicitly considered in the development of government policies	
**Indicator definitions** - please take these into account in the rating process	**SCOPE (FOR INFORMATION ONLY)**<br>This colum provides the type evidence collected for this indicator (as well as the evidence that will not be taken into account).
**INDICATOR (HIAP) 1: There are processes in place to ensure that population nutrition, health outcomes and reducing health<br>inequalities or health impacts in vulnerable populations are considered and prioritised in the development of all government policies relating to food**	➢ Includes policies, procedures, guidelines, tools and other resources that guide the consideration and assessment of nutrition, health outcomes and reducing health inequalities or health impacts in vulnerable populations prior to, during and following implementation of food-related policies<br>➢ Includes the establishment of cross-department governance and coordination structures while developing food-related policies
**INDICATOR (HIAP) 2: There are processes e.g. Health Impact Assessment's (HIAs) to assess and consider health impacts during the development of other non-food policies**	➢ Includes a government-wide HiAP strategy or plan with clear actions for non-health sectors<br>➢ Includes policies, guidelines, tools and other resources that guide the consideration and assessment of health impacts prior to, during and following implementation of non-food related policies (e.g. HIAs or health lens analysis)<br>➢ Includes the establishment of cross-department or cross-sector governance and coordination structures to implement a HiAP approach<br>➢ Includes workforce training and other capacity building activities in healthy public policy for non-health departments (e.g. agriculture, education, communications, trade)<br>➢ Includes monitoring or reporting requirements related to health impacts for non-health departments

Definitions

- **Food:** refers to food and non-alcoholic beverages. It excludes breastmilk or breastmilk substitutes.
- **Food environments:** the collective physical, economic, policy and socio-cultural surrounding, opportunities and conditions that influence people's food and beverage choices and nutritional status.
- **Government:** includes any government departments and, where appropriate, other agencies (i.e. statutory bodies such as offices, commissions, authorities, boards, councils, etc). Plans, strategies or actions by local government should not be included, although relevant information can be noted in the 'context/comments' sections.
- **Government implementation:** refers to the intentions and plans of the government and actions and policies implemented by the government as well as government funding for implementation of actions undertaken by non-governmental organisations, academic institutions, private companies (including consultants), etc.
- **Healthy/unhealthy food:** Categorisation of foods as healthy / unhealthy are in accordance with the WHO and EU guidelines). Where it is not clear which category to use, categorisation of foods should be informed by rigorous criteria or the use of a nutrient profiling model.
- **Nutrients of concern:** salt (sodium), saturated fat, trans fat, added sugar
- **Systems-based approaches**: This may include policies within other domains of health, a social-determinants of health approach, bringing together multiple departments or ministries to approach health and includes multiple levels of government.
- **Policy actions:** A broad view of "policy" is taken so as to include all government policies, plans, strategies and activities. Only current policy actions are considered, generally defined as policy activity of the previous 12 months (except where otherwise specified). Evidence of policy implementation takes consideration of the whole policy cycle, from agenda-setting, through to policy development, implementation and monitoring. A broad view of relevant evidence was taken, to include, inter alia:

    - Evidence of commitments from leadership to explore policy options
    - Allocation of responsibility to an individual/team (documented in a work plan, appointment of new position)
    - Establishment of a steering committee, working group, expert panel, etc.
    - Review, audit or scoping study undertaken
    - Consultation processes undertaken
    - Evidence of a policy brief/proposal that has been put forward for consideration
    - Preparation of a regulatory or economic impact assessment, health impact assessment, etc.
    - Regulations / legislation / other published policy details
    - Monitoring data
    - Policy evaluation report

ANNEX 2: BEST PRACTICE EXEMPLARS (2019)

The Best practice Exemplars are based on the version of the INFORMAS benchmarks from August 2017

DOMAIN 1 - FOOD COMPOSITION

**COMP1**	
**Food composition targets/standards/restrictions have been established by the government for the content of the nutrients of concern (added sugars, salt, saturated fat) in industrially processed foods, in particular for those food groups that are major contributors to population intakes of those nutrients of concern**<br><br>**(Trans fat has been excluded as it falls under EU regulation)**	
**International best practice examples (benchmarks)**	**UK:** In 2016**,** a key commitment of the 'Childhood obesity: a plan for action' was to launch a broad, structured sugar reduction programme to remove sugar from everyday products. All sectors of the food and drinks industry were challenged to reduce overall sugar across a range of products that contribute most to children's sugar intakes by at least 20% by 2020, including a 5% reduction in the first year of the programme (August 2016 to August 2017). The overall reduction between 2015 -2018 (in total sugar per 100g) was -2.9%(Public Health England, 2019). Only three food groups of the eight measured have managed at least a 5% reduction in the first year: sweet spreads and sauces, yoghurts and fromage frais, and breakfast cereals. There has been no sugar reduction in biscuits and chocolate bars(Public Health England, 2018). In contrast to this co-regulation, for products where the sugar tax applies over the same period a reduction in sugar of about 30% was found.<br><br>**South Africa:** In 2013, the South African Department of Health adopted mandatory targets for salt reduction in 13 food categories by means of regulation (Foodstuffs, Cosmetics and Disinfectants Act). There is a stepped approach with food manufacturers given until June 2016 to meet one set of category-based targets and another three years until June 2019 to meet the next (World Cancer Research Fund, 2016b, Hofman and Tollman, 2013). Overall, 67% of targeted foods had a sodium level at or below the legislated limit. About half (49%) of targeted foods not meeting the legislated limits were less than 25% above the maximum sodium level (Peters et al., 2017).<br><br>**The Netherlands:** On January 2014, the Dutch Ministry of Health, Welfare and Sport signed an agreement with trade organizations representing food manufacturers, supermarkets, hotels, restaurants, caterers and the hospitality industry to lower the levels of salt, saturated fat and calories in food products. The agreement includes voluntary ambitions for the period up to 2020 and aims to increase the healthiness of the food supply (World Cancer Research Fund, 2016b, National Agreement to improve Product Composition, 2017). Voluntary agreements have been made for

	the reduction of salt, saturated fat and energy/sugars in a variety of product groups and soft drinks.

**COMP2**	
**Food composition targets/standards/restrictions have been established by the government for the content of the nutrients of concern (added sugars, salt, saturated fat) in meals sold from food service outlets, in particular for those food groups that are major contributors to population intakes of those nutrients of concern.**<br><br>**(Trans fat has been excluded as it falls under EU regulation)**	
**International best practice examples (benchmarks)**	**New Zealand:** In New Zealand, The Chip group, funded 50% by the Ministry of Health and 50% by industry, aims to improve the nutritional quality of deep-fried chips served by food service outlets by setting an industry standard for deep frying oils. The standard for deep frying oil is maximum 28% of saturated fat, 3% linoleic acid and 1% of trans-fat. The Chip group oil logo for use on approved oil packaging was developed in 2010 (The Chip Group, 2016).<br><br>**New York City (US):** In 2009, New York City established voluntary salt guidelines for restaurant and store-bought foods. In 2010, this evolved into the National Salt Reduction Initiative that encouraged nationwide partnerships among food manufacturers and restaurants involving more than 100 city and state health authorities to reduce excess sodium by 25% in packaged and restaurant foods. In 2012, 26% of the categories met the targets, and 3% met the targets by the end of 2014. Between 2009 and 2014, there was nearly a 7% reduction in sodium levels in the U.S. food supply (New York City Health, 2017). There are 28 companies, including packaged food corporations and restaurants, who are committed to the salt reduction targets (Department of Health, 2014). In July 25, 2019, the Voluntary Sugar Reduction Targets from the National Salt and Sugar Reduction Initiative were revised. There is an open technical comment period until September 30, 2019 (NYC Health Department, 2019).

DOMAIN 2 – FOOD LABELLING

LABEL1
**Ingredient lists and nutrient declarations in line with Codex recommendations are present on the labels of all packaged foods**
Label 1 will not be included for benchmarking at national level as it's being addressed at EU level

LABEL2
**Evidence-based regulations are in place for approving and/or reviewing claims on foods, so that consumers are protected against unsubstantiated and misleading nutrition and health claims.**
Label 2 will not be included for benchmarking at national level as it's being addressed at EU level

LABEL3	
**One or more interpretive, evidence-informed front-of-pack supplementary nutrition information system(s) endorsed by the Government, which readily allow consumers to assess a product's healthiness, is/are applied to all packaged foods (examples are the Nutri-Score and traffic lights).**	
**International best practice examples (benchmarks)**	**UK:** In 2013, the Government published national guidance for voluntary 'traffic light' labelling for use on the front of pre-packaged food products. The label uses green, amber and red to identify whether products contain low, medium or high levels of energy, fat, saturated fat, salt and sugar. A combination of colour coding and nutritional information is used to show how much fat, salt and sugar and how many calories are in each product. The voluntary scheme is used by all the major retailers and some manufacturers (Deparment of Health, 2013).

Australia/New Zealand: The government-approved, voluntary 'Health Star Rating' (HSR) scheme applies a stare rating system where ratings range from ½ star (least healthy) to 5 stars (most healthy). The rating is based on the content of energy, saturated fat, sodium and total sugars content, along with certain 'positive' aspects of a food such as fruit and vegetable content, and in some instances, dietary fibre and protein content. Implementation of the system began in June 2014 and is overseen by a number of governmental instances, one of which evaluates progress. As of 2016, about 900 products had stars on them (Commonwealth of Australia, 2016).

France/ Belgium/Germany/Spain: Since October 2017, the five-colour NutriScore, the official voluntary label for France has been implemented as the official, voluntary FOP scheme in four European countries. It aims to limit the consumption of foods high in energy, saturated fats, sugar or salt, in the context of an overall improvement in the nutritional quality of diets (World Health Organisation, 2017). Based on a scientific algorithm, each product is given a score based on the content of the nutrients of concern (energy value and the amount of sugars, saturated fats and salt) and positive ones (the amount of fibre, protein, fruit, vegetables and nuts) (Colruyt Group, 2018). The system was developed by the Nutritional Epidemiology research Team at the University of Paris (Chantal, 2017).

Table1: The Nutri-Score (Colruyt Group, 2018)

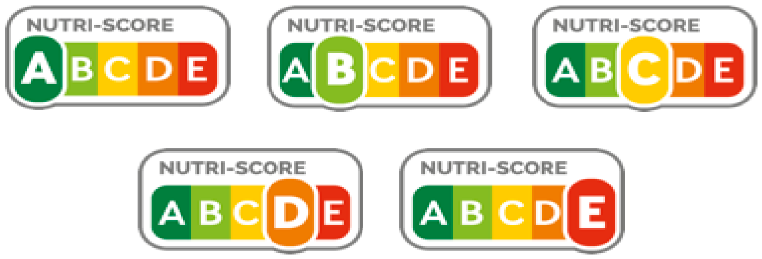

**LABEL4**	
**A simple and clearly-visible system of labelling the menu boards of all quick service restaurants (i.e. fast food chains) is applied by the government, which allows consumers to interpret the nutrient quality and energy content of foods and meals on sale.**	
**International best practice examples (benchmarks)**	**South Korea**: Since 2010, the Special Act on Safety Control of Children's Dietary Life has required all chain restaurants with 100 or more establishments to display nutrient information on menus including

	energy, total sugars, protein, saturated fat and sodium (World Cancer Research Fund, 2016c).<br><br>**Canada:** In effect since 1 January 2017, Ontario's Healthy Menu Choices Act 2015, requires food service premises that are part of a chain of 20 or more food service premises in Ontario (as well as certain cafeteria-style food service premises) to display calories for "standard food items" on menus, labels and display tags. The Act's regulations specify where caloric information is to be displayed on the menus, as well as the size, format and prominence of the display (Nutrition Resource Centre, 2017). Food service premises must also display information on daily caloric requirements: "*Adults and youth (ages 13 and older) need an average of 2,000 calories a day, and children (ages 4 to 12) need an average of 1,500 calories a day. However, individual needs vary.*"<br>Ontario's 36 public health units are responsible for implementation of the Act (Nutrition Resource Centre, 2017)<br><br>**Saudi Arabia**: In 2018, the Saudi Food and Drug Authority (SFDA) introduced mandatory measures on calorie labels on menus. These measures apply to all food facilities including restaurants, ice cream parlours, juice and fresh fruit vendors, bakeries, sweets shops, cafeterias, supermarkets, recreation facilities, colleges, universities and government agencies. Calories will be displayed at cashier desks, menu boards, table menus, drive-through menus, phone and web applications (Saudi Food & Druf Authority, 2018)<br><br>**USA:** In the US, the Patient Protection and Affordable Care Act (2010) (Office of the Federal Register, 2013) requires that all chain restaurants with 20 or more establishments to display energy information on menus. The menu labelling rule was implemented in May 2018 (Administration, 2019). The regulations will be pre-empted by the national law once implemented; local governments will still be able to enact menu labelling regulations for establishments not covered by national law. The regulations require vending machine operators of more than 20 vending machines to post calories for foods where the on-pack label is not visible to consumers by 26 July 2018 (World Cancer Research Fund, 2016c).<br><br>**Australia:** Legislation in Australian Capital Territory (Food Regulation 2002) and the States of New South Wales (Food Regulation 2010) and South Australia (Food Regulation 2002) requires restaurant chains (e.g. fast food chains, ice cream bars) with ≥20 outlets in the state (or seven in the case of ACT), or 50 or more across Australia, to display the kilojoule content of food products on their menu boards. Average adult daily energy intake of 8700kJ must also be prominently featured. Other chains/food outlets are allowed to provide this information on a voluntary

	basis but must follow the provisions of the legislation (World Cancer Research Fund, 2016c).<br><br>**New York City, USA:** Chain restaurants are required to put a warning label on menus and menu boards, in the form of a salt-shaker symbol (saltshaker inside a triangle), when dishes contain 2,300 mg of sodium or more. It applies to food service establishments with 15 or more locations nationwide. In addition, a warning statement is required to be posted at the point of purchase: This came into effect 1 December 2015 (World Cancer Research Fund, 2016c) (Department of Health and Mental Hygiene) and the Health Department started issuing violations June 6, 2016. Findings showed that directly following the sodium warning label regulation coming into effect, about 21% of restaurants had implemented the labels. By the end of February 2015, almost 70% of restaurants (from six of the ten chains) had implemented labels at one location or more. Overall, the findings suggested that the majority of restaurants were complying with the sodium warning label policy, despite issues with visibility, but that the labels may not be influencing consumer purchasing decisions (Downs, 2017).

DOMAIN 3 - FOOD PROMOTION

PROMO1	
**Effective policies are implemented by the government to restrict exposure and power of promotion of unhealthy foods to children including adolescents through broadcast media (TV, radio).**	
**International best practice examples (benchmarks)**	**Quebec:** In Québec, the Consumer Protection Act prohibits commercial advertising (including food and non-food) directed at children less than 13 years of age through television, radio and other media. Account must be taken of the context of its presentation, and in particular of: a) the nature and intended purpose of the goods advertised; b) the manner of presenting such advertisement; and c) the time and place it is shown. A cut-off of 15% share of child audience is used to protect children from TV advertising (Kent et al., 2011). Per indictment, a person is liable to: a fine ranging from $600 to $15,000 (in the case of a natural person); a fine ranging from $2,000 to $100,000 (in the case of a legal person).<br><br>**Norway/Sweden:** Under the Broadcasting Act, advertisements (food and non-food) may not be broadcast on television directed to children or in connection with children's programs. (World Cancer Research Fund, 2016e). Norway has implemented a self-regulation scheme approved and evaluated by Government. The scheme prohibits child-targeted unhealthy food marketing before 21:00 (9 PM) (MFU, 2016)<br><br>**Ireland:** Advertising, sponsorship, teleshopping and product placement of foods high in fats, sugars and salt, as defined by a nutrient profiling model, are prohibited during children's TV and radio programmes where over 50% of the audience are under 18 years old (Broadcasting Authority of Ireland, 2013). In addition, there is an overall limit on advertising of foods high in fats, sugars and salt adverts at any time of day to no more than 25% of sold advertising time and to only one in four advertisements. Remaining advertising targeted at children under the age of 18 must not include nutrient or health claims or

	include licensed characters (World Cancer Research Fund, 2016). As provided under the Broadcasting Act 2009, the BAI is required to undertake a statutory review of the effectiveness of the Children's Code. It is expected that review will commence in the second half of 2018 with revision and finalisation of the Code in 2019 (Broadcasting Authority of Ireland, 2019).<br><br>**Chile:** In June 2016, The Law of Nutritional Composition of Food and Advertising was enforced and restricts advertising directed to children under 14 years (for foods exceeding limits for calories, sugar, saturated fat and/or sodium in food and beverages). The regulatory norms define advertising targeted to children as programmes with an audience of greater than 20% children. Promotional strategies and incentives, such as cartoons, animations, and toys that could attract the attention of children are included in the ban. Monitoring and enforcement of the law are carried out by both regional and national public health authorities. Inspections are conducted on-site and online. After more than 2000 inspections, compliance with the law is improving, rising from under 40% to over 60% (Global Agricultural Information Network, 2018). A qualitative study carried out in 2017, found that the regulation has made mothers more aware of the importance of eating healthy, made it easier to choose healthy foods, and also made children actors in their own food choices (Correa et al., 2019).

**PROMO2**	
**Effective policies are implemented by the government to restrict exposure and power of promotion of unhealthy foods to children including adolescents through online and social media.**	
**International best practice examples (benchmarks)**	**Chile:** In June 2015, the Chilean authority approved the regulatory norms required for the law of Nutritional Composition of Food and Advertising implementation. The regulatory norms define limits for calories, saturated fat, sugar and sodium content considered "high" in foods and beverages. The law restricts advertising directed to children under the age of 14 years of foods in the "high in" category. The regulatory norms define advertising targeted to children as websites directed to children or with an audience of greater than 20% children, and according to the design of the advertisement. Promotional strategies and incentives, such as cartoons, animations, and toys

	are included in the ban. The regulation took effect 1 July 2016 and applies to all advertising media (World Cancer Research Fund, 2016).<br><br>**Quebec:** In Québec, the Consumer Protection Act prohibits commercial advertising directed at children less than 13 years of age through all media. Account must be taken of the context of its presentation, and in particular of: a) the nature and intended purpose of the goods advertised; b) the manner of presenting such advertisement; and c) the time and place it is shown (Kent et al., 2011). Any stakeholder involved in a commercial process (from the request to create an advertisement to its distribution, including its design) may be accused of not complying with the legislation in force. Per indictment, that person is liable to: a fine ranging from $600 to $15,000 (in the case of a natural person); a fine ranging from $2,000 to $100,000 (in the case of a legal person).<br><br>**Norway**: A government-approved and evaluated self-regulation scheme prohibits online food-marketing which is targeted at children under 13 years. Specifically, interactive games "specifically aimed at children and where a product's trademark, or other elements of the marketing of the product, form an integral part" will always be defined as child-targeted and a violation of the code (36). The scheme also applies to social media. In 2019, the code was revised. The age limit is still 13 years but in order to exercise caution in marketing to young people, it is advised to not conduct contests with age limit less than 16 years; to buy age groups less than 16 years in digital media; to use role models appealing to youth in media which is directly targeted to youth; and to encourage engagement (share, like, send in material) so that youth become marketing actors. Violations of the code results in no other sanctioning than "naming and shaming" of offenders (Matbransjens Faglige Utvalg, 2019).<br><br>**UK:** UK CAP rules have been reviewed so that online marketing targeted to under-16s is prohibited. This means that HFSS product ads are not permitted to appear in media that is specifically targeted at under-16s (for example, a children's magazine or on a website aimed at children); or where under-16s make up a significant proportion (more than 25%) of the audience (for example, advertorial content with an influencer that might have broad appeal but also a significant child audience) (Advertising Standards Authority, 2018).

PROMO4
**Effective policies are implemented by the government to ensure that unhealthy foods are not commercially promoted to children including adolescents in settings where children gather (e.g. preschools, schools, sport and cultural events).**

**International best practice examples (benchmarks)**	**Chile:** In June 2015, the Chilean authority approved the regulatory norms required for the Law of Nutritional Composition of Food and advertisings implementation. The regulatory norms define limits for calories, saturated fat, sugar and sodium content considered "high" in foods and beverages. The law restricts advertising directed to children under the age of 14 of foods in the "high in" category on school grounds, including preschools, primary and secondary schools. Chile has also restricted outdoor advertising, with ten municipalities adopting legislations banning outdoor marketing one block around schools (The Organisation for Economic Co-operation and Development, 2019). The law is scheduled to take effect in July 2016 (New York City Health, 2017). has also restricted outdoor advertising, with ten municipalities adopting legislations banning outdoor marketing one block around schools. The law is scheduled to take effect in July 2016 (New York City Health, 2017).<br><br>**Uruguay:** In September 2013, the government of Uruguay adopted Law No 19.140 (Healthy foods in schools) (Morley et al., 2013). The law prohibits the advertising and marketing of foods and drinks that don't meet the nutrition standards. Advertising in all forms is prohibited, including posters, billboards, and use of logos/brands on school supplies, sponsorship, and distribution of prizes, free samples on school premises and the display and visibility of food. The implementation of the law started in 2015 (World Cancer Research Fund, 2016).<br><br>**Hungary:** Based on Section 8 of Act XLVIII on Basic Requirements and Certain Restrictions of Commercial Advertising Activities (2008), Hungary prohibits all advertising directed at children under 18 in child welfare and child protection institutes, kindergartens, elementary schools and their dormitories. Health promotion and prevention activities in schools may only involve external organizations and consultants who are recommended by the National Institute for Health Development (World Cancer Research Fund, 2016e).<br><br>**Spain:** In 2011 the Spanish Parliament approved a Law on Nutrition and Food Safety, which stated that kindergartens and schools should be free from all advertising. Criteria for the authorisation of food promotion campaigns, nutritional education and promotion of sports or physical activity campaigns were developed jointly by the Spanish Agency for Consumer Affairs, Food Safety and Nutrition (AECOSAN) and the Regional Health Authorities and implemented in July 2015. AECOSAN and the Spanish Regional Education and Health Administrations monitor the enforcement of the Law (World Cancer Research Fund, 2016)

**PROMO3**	
**Effective policies are implemented by the government to restrict exposure and power of promotion of unhealthy foods to children including adolescents through non-broadcast media other than packaging and online/social media.**	
**International best practice examples (benchmarks)**	**Chile:** In May 2018, a new regulation launched, extended marketing restrictions of regulated foods in cinema and TV to a 6 AM to 10 PM time frame, expanding the scope of the original law. Additionally, starting in June 2018, any marketing done for "High in" foods or beverages must also show the following statement: "Choose foods with less warning labels" and then "Ministry of Health," which needs to be placed next to the MoH logo. This applies to marketing done in billboard, cinema, and other vehicles but food packages (Corvalán C, 2018).<br><br>**London UK:** On 25 February 2019, the Mayor of London, introduced restrictions on the advertising of unhealthy food across the entire Transport for London (TfL) public transport network, as part of his work to help tackle childhood obesity in London. The policy specifies that food and non-alcoholic drinks high in fat, salt and sugar (according to the UK Nutrient Profiling Model, are not permitted to be advertised on TfL-controlled buses, underground and over ground train networks, taxis, river services, trams and other transport systems. Food and drink brands, restaurants, takeaways and ordering services are required to promote their healthier food and drink instead of just advertising their brand. Advertisements for food and non-alcoholic drink products that are considered to be high in fat, salt, sugar may be considered for an exception by TfL if the advertiser can demonstrate, with appropriate

	evidence, that the product does not contribute to child obesity (Greater London Authority, 2019)

PROMO5	
**Effective policies are implemented by the government to ensure that unhealthy foods are not commercially promoted to children including adolescents, on food packages.**	
**International best practice examples (benchmarks)**	To be checked with the legal expert Amandine Garde)

DOMAIN 4 - FOOD PRICES

PRICES1
**Taxes or levies on healthy foods are minimised to encourage healthy food choices (e.g. low or no sales tax, excise, value-added or import duties on fruit and vegetables).**

**International best practice examples (benchmarks)**	**Australia:** Goods and services tax (GST) exemption exists for basic foods (including fresh fruits and vegetables)(Veerman, 2013).<br><br>**Tonga:** In 2013, as part of a broader package of fiscal measures, import duties were lowered from 20% to 5% for imported fresh, tinned or frozen fish in order to increase affordability and promote healthier diets (World Cancer Research Fund, 2016f).

**PRICES2**	
**Taxes or levies on unhealthy foods (e.g. sugar-sweetened beverages, foods high in nutrients of concern) are in place and increase the retail prices of these foods by at least 10% to discourage unhealthy food choices, and these taxes are reinvested to improve population health.**	
**International best practice examples (benchmarks)**	**Ireland:** On 1 May 2018, the Republic of Ireland's Sugar Sweetened Drinks Tax came into force under the Finance Act 2017 (No. 41 of 2017). The tax applies to non-alcoholic, water-based and juice based drinks which have added sugar content of 5g per 100mL and above. Drinks with over 8g of sugar per 100mL are taxed at 30 cents per litre, and drinks with between 5g and 8g of sugar per 100mL are taxed at 20 cents per litre. Fruit juices and dairy products are excluded from the tax (World Cancer Research Fund, 2018)<br><br>**UK:** In April 2018 the UK government's Soft Drinks Industry Levy came into force (as outlined in the Finance Act 2017). The Soft Drink Industry Levy applies to any pre-packaged soft drink with added sugar, containing at least 5g of total sugars per 100mL of prepared drink. Soft drinks that have a total sugar content of more than 5g and less than 8g per 100mL are taxed 0.18 British pounds ($0.25) per litre and drinks that have a total sugar content of 8g or more per 100mL are taxed 0.24 British pounds ($0.34) per litre. Milk-based drinks, milk substitute drinks, pure fruit juices, or any other drinks with no added sugar, alcohol substitute drinks, and soft drinks of a specified description which are for use for medicinal or other specified purposes are exempt from the levy. The levy applies to soft drinks produced and packaged in the UK and soft drinks imported into the UK (World Cancer Research Fund,

	2018). Manufacturers had two years to prepare ahead of this tax coming into effect and over 50% of them took action to cut sugar in their products during that period (Rathbone Greenbank Investments, 2019). It was forecasted that, the tax would bring in £520 million in its first year of operation, but this was revised down to £275 million as a result of company efforts to remove sugar from their products. Data from the first full year of the tax is not yet available, but receipts from April to October 2018 totalled £154 million. It was confirmed that the Department for Education would receive the full £1 billion funding that had originally been expected from the sugar tax in this Parliament (Parliment UK, 2017).<br><br>**Hungary:** A "public health tax" adopted in 2012 is applied on the salt, sugar and caffeine content of various categories of ready-to-eat foods, including soft drinks, energy drinks, and pre-packaged sugar-sweetened products. The tax is applied at varying rates. Soft drinks, for example, are taxed at $0.24 per litre and other sweetened products at $0.47 per litre. The tax also applies to products high in salt, including salty snacks with >1g salt per 100g, condiments with >5g salt per 100g and flavourings >15g salt per 100g (World Cancer Research Fund, 2016f, Biro, 2015).<br><br>**Mexico:** In December 2013, the Mexican legislature passed two new taxes as part of the national strategy for the prevention of overweight, obesity and diabetes. An excise duty of 1 peso ($0.80) per litre applies to sugary drinks. This is expected to increase the price of sugary drinks by around 10%. An ad valorem excise duty of 8% applies to foods with high caloric density, defined as equal to or more than 275 calories per 100 grams. The taxes entered into force on 1 January 2014. The aim is for the revenue of taxes to be reinvested in population health, namely providing safe drinking water in schools, but there is no evidence (yet) that this is the case as the taxes are not earmarked (World Cancer Research Fund, 2016f, Colchero, 2016). In 2019, a study was conducted to estimate changes in taxed and untaxed beverages by volume of beverage purchased after the sugar-sweetened beverage (SSB) tax was introduced in 2014 (Ng et al., 2019). Results found that, The HTLU-unhealthier and HTHU groups had the largest absolute and relative reductions in taxed beverages and increased their purchases of untaxed beverages. Households with lower purchases of untaxed beverages (HTLU unhealthier and LTLU) had the largest absolute and relative increases in untaxed beverages. Furthermore, among households with higher purchases of taxed beverages, the group with lowest socio-economic status had the greatest reduction in purchases of taxed beverages (Ng et al., 2019).<br><br>**Morocco**: On 1 January 2019, Article 5 of the Finance Act 2019 came into effect increasing Morocco's value-added tax on manufactured or imported

	soft and non-carbonated drinks with added sugars by 50%. Carbonated or non-carbonated water, mineral water, table water or others containing <10% of edible fruit juice or juice concentrates are taxed Moroccan Dirham (MAD) 0.45 (about $0.04) per litre; or those with >10% fruit juice or juice concentrates taxed at MAD 0.15 (about $0.016) per litre. Lemonades containing sugar with <6% lemon juice or concentrate equivalent were taxed MAD 0.45 per litre; or those containing >6% lemon juice or concentrate equivalent taxed at MAD 0.15 per litre. Unfermented carbonated or non-carbonated beverages were taxed MAD 1.24 (about $0.13) per litre. Energy drinks containing at least two stimulant ingredients such as caffeine, taurine and glucuronolactone were taxed MAD 6.00 (about $0.62) per litre (World Cancer Research Fund, 2018).<br><br>**Qatar:** In 2018, the Government of Qatar introduced Law No. (25) the 'Qatar Excise Tax Law' that came into effect on 1 January 2019. The Qatar Excise Tax Law introduced a 50% ad valorem tax on carbonated waters with added sugar, sweeteners or flavours, as well as concentrates, powders, gels or extracts intended to be made into a carbonated beverage. A tax rate of 100% is applied to beverages sold as energy drinks that contain stimulant substances (e.g. caffeine, taurine, ginseng, guarana). Carbonated non-flavoured waters, coffee and tea are excluded from the excise tax. The excise tax applies to all imported, produced or stockpiled aerated beverages (except unflavoured aerated water) and energy drinks (World Cancer Research Fund, 2018).

**PRICES3**	
**The intent of existing subsidies on foods, including infrastructure funding support (e.g. research and development, supporting markets or transport systems), is to favour healthy rather than unhealthy foods.**	
**International best practice examples (benchmarks)**	**Singapore:** The government, through the Health Promotion Board (HPB) increases the availability and use of healthier ingredients through the "Healthier Ingredient Scheme" (formerly part of the "Healthier Hawker" programme, launched in 2011), which provides in the first instance transitional support to oil manufacturers and importers to help them increase the sale of healthier oils to the food service industry (World Cancer Research Fund, 2016a). The Healthier Ingredient Subsidy Scheme offers a subsidy to suppliers stocking healthier items. Cooking oil is the first ingredient under the

	scheme, which subsidises oils with a saturated fat level of 35 per cent or lower.

**PRICES4**	
**The government ensures that food-related income support programs are for healthy foods**	
**International best practice examples (benchmarks)**	**USA:** In 2009, the U.S. Department of Agriculture's implemented revisions to the Special Supplemental Nutrition Program for Women, Infants, and Children (WIC) to improve the composition and quantities of WIC-provided foods from a health perspective. The revisions include: Increase the dollar amount for purchases of fruits and vegetables, expand whole-grain options, allow for yoghurt as a partial milk substitute, allow parents of older infants to buy fresh produce instead of jarred infant food and give states and local WIC agencies more flexibility in meeting the nutritional and cultural needs of WIC participants (World Cancer Research Fund, 2016f).<br><br>**USA:** In 2012, the USDA piloted a "Healthy Incentives Pilot" as part of the Supplemental Nutrition Assistance Program (SNAP, formerly "food stamps"). Participants received an incentive of 30 cents per US$ spent on targeted fruit and vegetables (transferred back onto their SNAP card). The Pilot included 7500 individuals (World Cancer Research Fund, 2016f). In New York City and Philadelphia, “Health Bucks” are distributed to farmer’s markets. When customers use income support (e.g. Food Stamps) to purchase food at farmer’s markets, they receive one Health Buck worth 2USD for each 5USD spent, which can then be used to purchase fresh fruit and vegetable products at a farmer’s market (World Cancer Research Fund, 2016f). In Philadelphia, the programme has been expanded to other retail settings like supermarkets and corner store.<br><br>**UK:** The British Healthy Start programme provides pregnant women and/or families with children under the age of four with weekly vouchers to spend on foods including milk, plain yoghurt, and fresh and frozen fruit and vegetables. Participants or their family must be receiving income support/jobseekers’ allowance or child tax credits. Pregnant women under the age of 18 can also apply. Full national implementation of the programme began in 2006 (World Cancer Research Fund, 2016f).

DOMAIN 5 - FOOD PROVISON

PROV1	
**The government ensures that there are clear, consistent policies (including nutrition standards) implemented in schools and early childhood education services for food service activities (canteens, food at events, fundraising, promotions, vending machines etc.) to provide and promote healthy food choices.**	
**International best practice examples (benchmarks)**	**Ireland:** The School Meals (Local Projects) Scheme, is an administrative scheme, operated directly by the Department of Employment Affairs and Social Protection (Healthy Ireland, 2017). The Scheme provides funding to primary and post-primary schools, local groups, voluntary organisations and community-based not-for-profit preschools operating their own school meals projects. The 'Nutrition Standards for School Meals', are being implemented under this scheme and aim to ensure that children and young people in schools participating in the scheme are provided with healthy balanced meals that follow the Healthy Eating Guidelines. These Nutrition Standards are food-based, and are provided for each meal type funded by the Scheme, that is: Breakfast or snack Lunch or after-school meal Dinner, only healthy food choices that meet the standards will be funded.The Standards will also be used by those administering the Scheme in the schools, commencing in January 2018, to ensure that food purchased complies with the Nutrition Standards when food contracts are being specified in the procurement process, and should also be applied when planning menus (Healthy Ireland, 2017).<br><br>**Jamaica:** In November 2018, the Ministry of Health published mandatory nutrient guidelines for beverages sold/served within all public educational institutions for children (i.e. early childhood, primary level and secondary level). The guidelines prohibit sweetened beverages that exceed a maximum sugar concentration of: 6g/100ml (effective 1 January 2019); 5g/100ml (effective 1 January 2020); 4g/100ml (effective 1 January 2021); and 2.5g/100ml (effective 1 January 2023). All unsweetened beverages are permitted The guidelines also caution against beverages containing >10mg/serve of caffeine, discourage the use of artificial sweeteners and recommend beverage portions sold/served of <12 ounces (not including water).<br><br>**Chile:** In 2012, the Chilean government approved a Law of Nutritional Composition of Food and Advertising [51]. In June 2015, the Chilean authority approved the regulatory norms required for the law's implementation. The

	regulatory norms define limits for calories, saturated fat, sugar and sodium content considered 'high' in foods and beverages. The law prohibits the sale of foods in the 'high in' category in schools. The law came into effect on 27 June, 2016. In 2019, a study conducted on the impact of this law funds that, foods exceeding any cut-offs decreased from 90.4% in 2014 to 15.0% in 2016. Solid products had a substantial reduction in calories, sugar, saturated fat, and sodium. Liquid products had a reduction in calories, total sugar, and saturated fat, whereas sodium increased. This was a result of changes in product mix (Massri et al., 2019).<br><br>**Finland**: In 2008, the National Nutrition Council approved nutrition recommendations for school meals. These include food and nutrient recommendations for salt, fibre, fat, starch, fat and salt maximums for meat and processed meat, and drinks. There are also criteria for snacks provided in schools. New recommendations on Eating and learning together - recommendations for school meals have been published in 2017 (National Nutrition Council, 2017). In 2018, the early childhood education: Health and joy from food - meal recommendations for early childhood education and care, were published (National Nutrition Council, 2018). The 2018 published recommendations for families with children: Eating together - food recommendations for families with children, were updated in 2019 (National Institute for Health and Welfare, 2019). Additionally, Finland published its first nutrition recommendations for upper secondary schools and vocational schools.<br><br>**UK:** England, Scotland, Wales and Northern Ireland have mandatory nutritional standards for school food, which also apply to food provided in schools other than school lunches. These standards apply to most state schools (with the exception of around 4,000 academies established between September 2010 and June 2014, which are exempt) and restrict foods high in fat, salt and sugar, as well as low quality reformed or reconstituted foods .<br><br>**Brazil:** The national school feeding programme (Fundo Nacional de Desinvolvimento da Educacao, 2016) mandates a weekly minimum of fruits and vegetables, regulates sodium content and restricts the availability of sweets in school meals. A school food procurement law (Ministry of Education, 2016), approved in 2001, limits the amount of processed foods purchased by schools to 30%, and bans the procurement of drinks with low nutritional value, such as sugary drinks. The law requires schools to buy locally grown or manufactured products, supporting small farmers and stimulating the local economy. Resolution no 38 (16 July 2009) sets food- and nutrition-based standards for the foods available in the national school meal programme (Law 11.947/2009). Article 17 prohibits drinks of low nutritional value (e.g. soda), canned meats, confectionary and processed foods with a sodium and saturated fat content higher than a specified threshold.

	**Costa Rica:** Executive Decree No 36910-MEP-S (2012) of the Costa Rican Ministries of Health and Education sets restrictions on products sold to students in elementary and high schools, including food with high levels of fats, sugars and salt, such as chips, cookies, candy and carbonated sodas. Schools are only permitted to sell food and beverages that meet specific nutritional criteria. The restrictions were upheld by the Constitutional Court in 2012 following a challenge by the food industry (World Cancer Research Fund, 2016d).<br><br>**Hungary**: Since 2012, food and beverages subject to the public health product tax may not be sold on school premises or at events organized for school children, including out of school events based on the Ministerial Decree 20/2012 (VIII.31) on the Operation of Public Education Institutions and the of Names of Public Education Institutions. Section 130(2) of the Decree requires the head of the educational institution to consult the school health service prior to entering into agreements with vending machine operators or food vending businesses. The school health service verifies whether the products to be sold meet the nutritional guidelines set by the National Institute of Pharmacy and Nutrition. Products that do not comply are prohibited (World Cancer Research Fund, 2016d).<br><br>**Uruguay**: In September 2013, the government of Uruguay adopted Law No 19.140 on 'healthy eating in schools. It mandated the Ministry of Health to develop standards for food available in canteens and kiosks in schools, prohibited advertising for these same foods and restricted the availability of saltshakers. The school food standards were elaborated in March 2014 and aimed to promote foods with natural nutritional value with a minimum degree of processing and to limit the intake of free sugars, saturated fat, trans fat and sodium. Limits are set per 100g of food, 100ml for drinks and also per 50g portion. This was implemented in public schools in 2015 (World Cancer Research Fund, 2016d, Fundo Nacional de Desinvolvimento da Educacao, 2016). This was implemented in public schools in 2015 (World Cancer Research Fund, 2016d, Fundo Nacional de Desinvolvimento da Educacao, 2016).

PROV2
**The government ensures that there are clear, consistent policies in other public sector settings for food service activities (canteens, food at events, fundraising, promotions, vending machines, public procurement standards etc.) to provide and promote healthy food choices**.

**International best practice examples (benchmarks)**	**Latvia:** In 2012, the government set salt levels for all foods served in hospitals and long-term social care institutions. Levels may not exceed 1.25g of salt/100g; fish products may contain up to 1.5g salt/100g (World Cancer Research Fund, 2016d).<br><br>**Ireland:** The HSE Vending Policy 2019 replaces the 2014 Healthier Vending Policy and applies to all vending machines that stock cold soft drinks, confectionery and snacks on HSE premises & premises funded by the HSE. Sugar sweetened beverages will not be stocked in vending machines, 50% of beverages stocked will be still water and the remaining beverages stocked will include non-sugar sweetened beverages e.g. diet drinks, juices, flavoured and sparkling water. Snacks containing more than 200 calories per packet will not be stocked in machines. An exception to this is 3 packets of dried fruits, nuts or seeds (plain and unsalted). Products will be clearly labelled with the number of calories per product related fields (Health Service Executive, 2019).<br><br>**Bermuda:** In 2008, the Government Vending Machine Policy was implemented in government offices and facilities to ensure access to healthy snacks and beverages for staff. The policy requires that all food and beverages in vending machines on government premises meet specific criteria based on levels of total fat, saturated fat, trans fat, sodium and sugar. Criteria exclude nuts and 100% fruit juices (World Cancer Research Fund, 2016d).<br><br>**New York**: New York City's Food Standards (enacted with Executive Order 122 of 2008) set nutritional standards for all food purchased or served by city agencies, which applies to prisons, hospitals and senior care centres. The Standards include: maximum and minimum levels of nutrients per serving; standards on specific food items (e.g. only no-fat or 1% fat milk); portion size requirements; the requirement that water be offered with food; a prohibition on the deep-frying of foods; and daily calorie and nutrient targets, including population-specific guidelines (e.g. children, seniors) (World Cancer Research Fund, 2016d, Lederer, 2014). As of 2015, 11 city agencies are subject to the NYC Food Standards, serving and selling almost 250 million meals a year. The Food Policy Coordinator has the responsibility of ensuring adherence with the Food Standards. Self-reported compliance with the standard is 96%.<br><br>**Wales:** Vending machines are prohibited in National Health Service Hospitals. The government issued a guidance defining what is allowed and not and has liaised with major vending providers to find ways to introduce healthier food options (Health Promoting Hospital Vending Directions and Guide 2008).<br><br>**The Netherlands:** The Netherlands Nutrition Centre introduced the 'Guidelines for Healthier Canteens', which can be applied in canteens at schools, sports clubs and workplaces to make them more healthy. The Guidelines for Healthier Canteens

	cover canteens at product level and at the level of the full range of food and drink being offered, together with the canteen's general display layout. The framework of the Guidelines for Healthier Canteens defines three different levels: bronze, silver and gold (Netherlands Nutrition Centre, 2017).

PROV3	
**The government ensures that there are clear, consistent public procurement standards in public sector settings for food service activities to provide and promote healthy food choices.**	
**International best practice examples (benchmarks)**	**Brazil:** A school food procurement law (Ministry of Education, 2016), approved in 2001, limits the amount of processed foods purchased by schools to 30%, and bans the procurement of drinks with low nutritional value, such as sugary drinks. The law requires schools to buy locally grown or manufactured products, supporting small farmers and stimulating the local economy<br><br>**UK:** The UK Government Buying Standard for Food and Catering Services (GBSF of 2014, updated March 2015, sets out standards for the public sector when buying food and catering services. It is supported by the Plan for Public Procurement: Food and Catering Services (2014). The nutrition requirements must be followed by schools, hospitals, care homes, communities and the armed forces. To improve diets, the GBSF sets maximum levels for sugar in cereals and generally for saturated fat and salt, in addition to minimum content of fibre in cereals and fruit in desserts. (World Cancer Research Fund, 2016e).

**PROV4**	
**The Government ensures that there are good support and training systems to help schools and other public sector organisations and their caterers meet the healthy food service policies and guidelines**	
**International best practice examples (benchmarks)**	**Australia:** The Healthy Eating Advisory Service supports settings such as childcare centers, schools, workplaces, health services, food outlets, parks and sporting centres to provide healthy foods and drinks to the public in line with Victorian Government policies and guidelines. The Healthy Eating Advisory Service is delivered by experienced nutritionists and dieticians at Nutrition Australia Victorian Division. The support includes training cooks, chefs, foods service and other key staff, discovering healthier recipes, food ideas and other helpful resources to provide healthier menus and products (Healthy Eating Advisory Service, 2017).

	**Singapore:** The National Workplace Health Promotion Programme, launched in Singapore in 2000, is run by the Health Promotion Board. Both private and public institutions are encouraged to improve the workplace environment by providing tools and grants. Grants are awarded to help companies start and sustain health promotion programmes. Tools include a sample Healthy Workplace Nutrition Policy, a sample Healthy Workplace Catering Policy, and a detailed Essential Guide to Workplace Health, setting out ways to transform the workplace into a health-supporting work environment (World Cancer Research Fund, 2016d).<br><br>**Netherlands:** The Healthy School Canteen Brigade (https://gezondeschoolkantine.voedingscentrum.nl/nl.aspx) is a team consisting of dieticians and health scientists of the Dutch Nutrition Center to help school realize healthy canteens. They visit schools in the Netherlands and give them advice. The Dutch Nutrition Center also developed the canteen scan (https://gezondeschoolkantine.voedingscentrum.nl/nl/stap-voor-stap/kantinescan.aspx), a tool to check the level of healthiness of canteens and which gives practical advises.

**PROV5**	
**The Government actively encourages and supports private companies to provide and promote healthy foods and meals in their workplaces**	
**International best practice examples (benchmarks)**	• **Ireland:** A Healthy Workplaces Framework has been developed as part of the governments public health Framework, Healthy Ireland - A Framework for improved health and wellbeing: 2013 – 2025, by the Department of Health and the Department of Business, Enterprise and Innovation (Department of Health, 2013) (McAvoy, 2018). It was developed following a public consultation with interested stakeholders and aims to enhance existing initiatives to, facilitate the sharing of experience and learning, and also provide the necessary supports and tools for organisations or companies who haven't yet developed their own resources. The Healthy Workplaces Framework is due to be implemented in 2020.

DOMAIN 6 - FOOD IN RETAIL

**RETAIL1**	
**Zoning laws and policies are implemented to place limits on the density or placement of quick serve restaurants or other outlets selling mainly unhealthy foods in communities and/or access to these outlets (e.g. opening hours).**	
**International best practice examples (benchmarks)**	**South Korea**: In 2010 the Special Act on Children's Dietary Life Safety Management established the creation of 'Green Food Zones' around schools, banning the sale of foods (fast food and soda) deemed unhealthy by the Food and Drug Administration of Korea within 200 metres of

	schools (Ministry of Food and Drug Safety, 2017, Bae, 2012) In 2016, Green Food Zones existed at over 10000 schools.<br><br>**UK:** Around 15 local authorities have developed "supplementary planning documents" on the development of hot food takeaways. The policies typically exclude hot food takeaways from a 400m zone around the target location. All policies include secondary schools, some policies also include primary schools, parks and youth centres(World Cancer Research Fund, 2016).<br><br>**Detroit USA:** In Detroit, the zoning code prohibits the building of fast food restaurants within 500 ft. of all elementary, junior and senior high schools (World Cancer Research Fund, 2016).

**RETAIL2**	
**Zoning laws and policies are implemented to encourage the availability of outlets selling fresh fruit and vegetables and/or access to these outlets (e.g. opening hours, frequency i.e. for markets)-.**	
**International best practice examples (benchmarks)**	**USA:** February 2014 the US Congress formally established the Healthy Food Financing Initiative (following a three-year pilot) which provides grants to states to provide financial and/or other types of assistance to attract healthier retail outlets to underserved areas. The pilot distributed over 140 million USD in grants to states to provide financial and other types of assistance to attract healthier retail outlets in underserved areas. To date, 23 US states have implemented financing initiatives (World Cancer Research Fund, 2016).<br><br>**New York City (USA):** The 'Green Cart Permit' was developed with reduced restrictions on zoning requirements to increase the availability of fresh fruits and vegetables in designated, underserved neighbourhoods (World Cancer Research Fund, 2016). In 2008 New York City made 1000 licenses for green carts available to street vendors who exclusively sell fresh fruit and vegetables in neighbourhoods with limited access to healthy foods (World Cancer Research Fund, 2016). In addition, in 2009, New York City established the food retail expansion to support the health program of New York City (FRESH). Under the programme, financial and zoning incentives are offered to promote neighbourhood grocery stores offering fresh meat, fruit and vegetables in under-served communities. The

	financial benefits consist of an exemption or reduction of certain taxes. The zoning incentives consist of providing additional floor area in mixed buildings, reducing the amount of required parking, and permitting larger grocery stores in light manufacturing districts.

**RETAIL3**	
**The Government ensures existing support systems are in place to encourage food stores to promote the in-store availability of healthy foods and to limit the in-store availability of unhealthy foods**	
**International best practice examples (benchmarks)**	**USA:** The Special Supplemental Nutrition Program for Women, Infants, and Children (WIC) requires WIC authorised stores to stock certain healthier products (e.g. wholegrain bread) (World Cancer Research Fund, 2016f).<br><br>**The Netherlands:** The National Action plan for vegetables and Fruit is a cooperation of government, industry and civil society organisations. The Goal is to increase the consumption of vegetables and fruits in 3 years (2018-2020) by linking and strengthening existing initiatives**.** The National Action Plan vegetables and fruit stimulates consumers to eat more vegetables and fruit using the motto 'Go for Colour'. As part of 'Go for Colour' an in-store experiment has taken place promoting the in-store availability of vegetables and fruit.

**RETAIL 4**	
**The government ensures existing support systems are in place to encourage the promotion and availability of healthy foods in food service outlets and to discourage the promotion and availability of unhealthy foods in food service outlets**	

International best practice examples (benchmarks)	**USA:** In December 2011, San Francisco implemented the Health Food Incentives Ordinance which bans restaurants, including takeaway restaurants, to give away toys and other free incentive items with children's meals unless the meals meet nutritional standards as set out in the Ordinance: meals must not contain more than 600 calories and include a min amount of fruits and vegetables. It also applies to drinks with excessive calories, fat, excessive sugars ,added non-nutritive sweeteners or caffeine (World Cancer Research Fund, 2016e).<br><br>**France:** Since January 2017 France has banned unlimited offers of sweetened beverages for free or at a fixed price in public restaurants and other facilities accommodating or receiving children under the age of 18. Sweetened beverages are defined as any drink sweetened with sugar or artificial (caloric and non-caloric) sweeteners, including flavoured carbonated and still beverages, fruit syrups, sport and energy drinks, fruit and vegetable nectars, fruit- and vegetable-based drinks, as well as water- milk- or cereal based beverages (World Cancer Research Fund, 2016).<br><br>**Los Angles, USA:** In September 2013, the Los Angeles County Department of Public Health launched Choose Health LA Restaurants in partnership with local restaurants to promote healthier meal choices. Restaurants must apply to become a partner. Participating restaurants offer customers smaller portion size options (in addition to existing items on the menu), healthier meals for children that include vegetables and fruit, healthy beverages, non-fried food and free chilled water. Participating restaurants are recognised as Public Health partners in promoting healthier communities.

DOMAIN 8 – LEADERSHIP

**LEAD1**

There is strong, visible, political support (at the head of government or state/ ministerial level) for improving food environments, population nutrition, diet related NCDs and their related inequalities"	
**International best practice examples (benchmarks)**	**New York City (USA):** As Mayor of New York City, Michael Bloomberg prioritised food policy and introduced a number of ground breaking policy initiatives including 'Health Bucks', a restriction on trans fats, establishment of an obesity taskforce, a portion size restriction on sugar-sweetened beverages, public awareness campaigns, etc. He showed strong and consistent leadership and a commitment to innovative approaches and cross-sectoral collaboration (Kelly, 2016).<br><br>**Brazil:** The Minister of Health showed leadership in developing new dietary guidelines that are drastically different from the majority of dietary guidelines created by any nation to date, and align with some of the most commonly cited recommendations for healthy eating (World Public Health Nutrition Association Update team, 2014).<br><br>**Some Caribbean Countries:** Active NCD commissions exist in six of the 20 CARICOM member states (Bahamas, Barbados, Bermuda, British Virgin Islands, Dominica, Grenada) which are all housed in their Ministries of Health, with members recommended by the Minister of Health and appointed by the Cabinet of Government for a fixed duration; all include government agencies and to a varying degree, civil society and the private sector<br><br>**Ireland:** Healthy Ireland "A Framework for Improved Health and Wellbeing 2013-2025", was launched in 2013 and aims to increase the proportion of people who are healthy at all stages of life, reduce health inequalities, protect the public from threats to health and wellbeing and create an environment where every individual and sector of society can play their part in achieving a healthy Ireland. In 2016, the Government approved the creation of a Healthy Ireland Fund with an initial allocation of €5 million approved in Budget 2017 to establish and support the implementation of Healthy Ireland programmes and projects in a variety of settings. The primary aim of the fund is to support innovative, cross-sectoral, evidence-based projects and initiatives that support the implementation of key national policies in areas such as obesity, smoking, alcohol, physical activity and sexual health(Pobal, 2016). The Department of Health has approved a third round of funding, which aims to support local and national organisations to deliver actions that will improve health and wellbeing in line with Healthy Ireland, A Framework for Improved Health and Wellbeing 2013-2025 (Department of Health, 2019a).

	**The Netherlands:** In 2018, the Ministry of Health, together with more than 70 organizations signed the National Prevention Agreement. It aims to reduce smoking, overweight and problematic alcohol consumption. The agreement includes voluntary ambitions, objectives and actions on these three subjects for the period (2018-2040). The National prevention agreement acknowledges that peoples' contexts is important, and that, among other factors, a healthy environment is needed for those that need it in order to prevent overweight, obesity and NCD's. The agreement formulates that inhabitants of the Netherlands need a healthy social, economic and physical environment, that supports healthy living, including schools, care facilities, restaurants, cafes, caterers and supermarkets. Specific voluntary targets with respect to the food environment are:<br>• In 2020, 2,500 sports clubs will be working on providing a healthier range of food products in their sports canteens.<br>• No later than 2025, 50% of hospitals will offer a healthy diet; no later than 2030, all of them will.<br>• In 2020, there will be 950 healthy school canteens. This means that 50% of all school canteens will be healthy.<br>• Businesses will develop healthier products (e.g. reformulation by limiting sugar content)<br>• The central government wants to introduce, no later than 2020, a new, broadly supported food-choice logo based on thorough, independent consumer research.<br>An example of a voluntary actions formulated is: Supermarkets will entice consumers to buy more products from the Wheel of Five, among other things by telling them which products are suitable (Netherlands, 2019).

**LEAD2**

Clear population intake targets have been established by the government for the nutrients of concern and / or relevant food groups to meet WHO and national recommended dietary intake levels	
**International best practice examples (benchmarks)**	**Brazil:** The "Strategic Action Plan for Confronting NCDs in Brazil, 2011-2022 specifies a target of increasing adequate consumption of fruits and vegetables, from 18.2% to 24.3 % between 2010 and 2022 and reduction of the average salt intake of 12 g to 5 g, between 2010 and 2022 (Ministry of Health Brazil, 2011).<br><br>**South Africa:** The South African plan for the prevention and control of non-communicable diseases includes a target on reducing mean population intake of salt to <5 grams per day by 2020 (Ministry of Health South Africa, 2013).<br><br>**UK:** In August 2016, government set out its approach to reduce the prevalence of childhood obesity in 'Childhood obesity: a plan for action'. A key commitment in the plan was to launch a broad, structured sugar reduction programme to remove sugar from everyday products. All sectors of the food and drinks industry are challenged to reduce overall sugar across a range of products that contribute most to children's sugar intakes by at least 20% by 2020, including a 5% reduction in the first year of the programme (Public Health England, 2017).<br><br>**The Netherlands:** On January 2014, the Dutch Ministry for Health, Welfare and Sport, signed an agreement with trade organisations representing food manufactures, supermarkets, hotels, restaurants, caterers and the hospitality industry. The agreement included intake targets for example; a maximum of 6 grams of salt consumption per day in 2020 and consuming a maximum of 10% energy from saturated fat per day in 2020 (The Central Government for the Netherlands, 2014)<br><br>**Norway:** The National Action Plan for a Better Diet (2017-2021) contains quantitative intake targets for nutrient of concern and specific food groups in the population. By 2021, the plan sets out a reduction of the following nutrients: Added sugar from 13 to 11E%; saturated fat from 14 to 12E%; and a 22% reduction in salt intake from 10 g/day. There are specific targets to halve the proportion of youth that consumes sugar-sweetened beverages or sweets more than 5 times per week; to double the proportion of youth that eats fruit and vegetables daily; and to increase by 20% the proportion of youth that eats fish at least once a week. There are also targets to increase the intake of fruit, vegetables, whole grain products and fish with 20% in the general population (Røynstrand, 2017).

LEAD3	
**Clear, interpretive, evidenced-informed food based dietary guidelines have been established and implemented.**	
**International best practice examples (benchmarks)**	**Brazil:** The national dietary guidelines of Brazil address healthy eating from a cultural, ethical and environmental perspective, rather than based on number of servings per food group. The main recommendations are: 'Make natural or minimally processed foods the basis of your diet'; 'use oils, fats, salt, and sugar in small amounts for seasoning and cooking foods'; 'use processed foods in small amounts'; 'avoid ultra-processed foods'. They also provide advice on planning, shopping and sharing meals, as well as warning people to be wary of food marketing and advertising (Monteiro, 2015, Ministry of Health Brazil, 2014).<br><br>**Ireland:** A Healthy Weight for Ireland, Obesity Policy and Action Plan - 2016–2025 called for the development of a suite of Healthy Eating Guidelines for the general population. These revised Healthy Eating Guidelines and Food Pyramid resources, are an early action under the Plan (Action 5.3.) (Department of Health, 2016a). The revised Healthy Eating Guidelines and Food Pyramid are based on the FSAI Scientific Recommendations for Healthy Eating Guidelines in Ireland from 2011 (Food Safety Authority of Ireland, 2011). They describe how to build a healthy diet, for different age groups (from 5 years of age), depending also on gender and activity levels. The Guidelines, Food Pyramid and supporting resources have been published, disseminated and communicated in 2017, including dissemination of the new Guidelines to all primary and post-primary schools. The revised Healthy Eating Guidelines and Food Pyramid toolkit has been developed by the Department of Health and the Health Service Executive with key stakeholders and aims to help reduce the intake of high fat, salt and sugar (HFSS) foods and drinks from the Top Shelf of the Food Pyramid (Healthy Ireland, 2016)<br><br>**The Netherlands:** The Dutch Health Council published the 'Guidelines Good Food' 2015. These guidelines advise to eat more plant-based and less animal-based food and include advice on the intake of different food

	products. The Dutch Nutrition Center published the 'Wheel of Five' Guidelines, based on the 'Guidelines Good Food' of the Dutch Health Council. The 'Wheel of Five' includes advice on the ingredients of a healthy diet, making a distinction between five sections: (1) Vegetables and fruit (2) spread and cooking fats (3) Fish, legumes, meat, eggs, nuts and dairy products (4) Bread, cereal products and potatoes and (5) Drinks (Health Council of the Netherlands, 2015)

**LEAD4**	
**There is a comprehensive, transparent, up-to-date implementation plan linked to national needs and priorities, to improve food environments , reduce the intake of the nutrients of concern to meet WHO and national recommended dietary intake levels, and reduce diet-related NCDs**	
**International best practice examples (benchmarks)**	**Ireland:** A Healthy Weight for Ireland', the Obesity Policy and Action Plan 2016-2025 (OPAP), was launched in September 2016 under the auspices of the Healthy Ireland agenda. The OPAP covers a ten-year period up to 2025, which prescribed 'Ten Steps Forward' that would be taken to reverse obesity trends, prevent health complications and reduce the overall burden for individuals, families, the health system, and the wider society and economy (Department of Health, 2016a). A new Obesity Policy Implementation Oversight Group (OPIOG) was established in October 2017 and a progress report on each recommendation in the OPAP is currently being finalised under the aegis of the OPIOG (Oireachtas, 2019). As set out in Healthy Ireland, integrated health and social impact assessments will be conducted on relevant policy areas to support other government departments in contributing towards the prevention of overweight and obesity. (Healthy Ireland, 2013-2025)

**LEAD5**	
**Government priorities have been established to reduce inequalities or protect vulnerable populations in relation to diet, nutrition, obesity and NCDs**	
**International best practice examples (benchmarks)**	**New Zealand:** The Ministry of Health reports the estimates derived from health surveys and nutrition surveys by four subpopulation groups including age group, gender, ethnic group and an area level deprivation

	index. Similarly, estimates derived from other data types (e.g. mortality) are presented by these subpopulation groups. The contracts between MoH and NGOs or other institutions include a section on Maori Health and state: *"An overarching aim of the health and disability sector is the improvement of Maori health outcomes and the reduction of Maori health inequalities".* In the specific contract between the Ministry of Health and Agencies for Nutrition Action the first clause is on Maori Health relating to compliance with any Maori specific service requirements, quality requirements and specific monitoring requirements contained in the Service specifications to this agreement.<br><br>**Australia:** The National Indigenous Reform Agreement (Closing the Gap) is an agreement between the Commonwealth of Australia and the States and Territories. The objective of this agreement is to work together with Indigenous Australians to Close the Gap in Indigenous disadvantage. The targets agreed to by COAG relate to health or social determinants of health. For the target 'Closing the life expectancy gap within a generation (by 2031)', one of the performance indicators is the prevalence of overweight and obesity**.**<br><br>**Ireland**: **Step 9** of the Healthy Weight for Ireland: Obesity Policy and Action Plan 2016-2025 aims to, allocate resources to those population groups most in need of support in the prevention and management of obesity, with particular emphasis on families and children during the first 1,000 days of life. The priority actions to commence in first year were to, assess the needs of vulnerable groups as the basis of allocation of resources for preventative and treatment services for children and adults (Department of Health, 2016b). The Healthy Ireland fund was established in 2017 with an allocation of €5 million and with additional allocations of €5 million in 2018 and 2019. The first round of the Fund was distributed through Local Community Development Committees, Children and Young Person's Services Committees and statutory organisations. The Fund has been effective at targeting population groups that experience health inequalities. In Round 1 (2017/18), there was a focus on specific groups experiencing health inequalities, including people living in areas of social disadvantage (71% of actions), people with disabilities (45%), people from new communities including refugees and asylum seekers (39%) and members of the Traveller community (36%). Furthermore, of the local actions that were implemented in Round 1, 61% related to physical activity and 32% were related to food, nutrition and weight management. Round 2 of the Fund is currently being implemented (Oireachtas, 2019) The Healthy Ireland 2019 communications and citizen engagement campaign has continued on from 2018, launching on the 8th of April 2019, with an

	announced funding of €1 million to boost community engagement on health and wellbeing in every county (Department of Health, 2019b).

DOMAIN 9 - GOVERNANCE

**GOVER1**	
**There are procedures in place to restrict commercial influences on the development of policies related to food environments where they have conflicts of interest with improving population nutrition. for example: restricting lobbying influences.**	
**International best practice examples (benchmarks)**	**USA:** Mandatory and publicly accessible lobby registers exist at the federal level, as well as in nearly every state. Financial information must be disclosed, and the register is enforced through significant sanctions. A number of pieces of legislation uphold compliance with the register including Lobbying Disclosure Act of 1995 and the Honest Leadership and Open Government Act 2007.<br><br>**New Zealand:** The State Services Commission has published Best Practice Guidelines for Departments Responsible for Regulatory Processes with Significant Commercial Implications. They cover the development and operation of a regulatory process and include specific references to principles around stakeholder relationship management (State Services Commission).<br><br>**Australia:** The Australian Public Service Commission's Values and Code of Conduct includes a number of relevant sections such as the Conflict of Interest, Working with the Private Sector and other Stakeholders and the Lobbying Code of Conduct.<br><br>**Ireland:** The Regulation of Lobbying Act 2015 was signed into law in March 2015. The purpose of the Act is to, provide for a web-based Register of Lobbying to make information available to the public on the identity of those communicating with designated public officials on specific policy, legislative matters or prospective decisions. In support of the Act's objectives to foster transparency and the proper conduct of lobbying activities, the Code of Conduct for persons carrying on lobbying activities was established. Its purpose is to govern the behaviour of persons carrying on lobbying activities. The provisions of the Act can apply to employers; to

	representative or advocacy bodies; to professional lobbyists or third parties who are being paid to communicate on behalf of a client or other person; and, significantly, to any person communicating about the development or zoning of land (Standards in Public Office Commission, 2019).

**GOVER2**	
**Policies and procedures are implemented for using evidence in the development of food and nutrition policies**	
**International best practice examples (benchmarks)**	**Australia:** The National Health and Medical Research Council Act 1992 (NHMRC Act) requires NHMRC to develop evidence-based guidelines. These national guidelines are developed by teams of specialists following a rigorous nine-step development process (Government of Canada, 2016).

**GOVER3**	
**Policies and procedures are implemented for ensuring transparency in the development of food and nutrition policies**	
**International best practice examples (benchmarks)**	**New Zealand:** Food Standards Australia New Zealand (FSANZ) is required by the Food Standards Australia New Zealand Act 1991 to engage stakeholders in the development of new standards. This process is open to everyone in the community including consumers, public health professionals, and industry and government representatives. FSANZ has developed a Stakeholder Engagement Strategy 2013-16 that outlines the scope and processes for engagement. Under the Stakeholder Engagement Priorities 2013-16, it outlined "maintain our open and transparent approach" as one of the first priorities (Food Standards Australia New Zealand, 2013).

	**Norway:** The Public Administration Act provides general procedural rules for public administration. The Act regulates the administrative procedures when decisions are made, especially the rights of parties during the procedures. The Central Government Communication Policy contains the central goals and principles of the central government's communication with citizens, businesses, organizations and other public sector activities. The goals of the central government communication policy state that the citizens shall: receive accurate and clear information about their rights, duties and opportunities; have access to information about central government activities; and be invited to participate in the formulation of policy, schemes and services (Norwegian Ministry of Local Government and Modernisation, 2019).

**GOVER4**	
**The government ensures public access to comprehensive nutrition information and key documents (e.g. budget documents, annual performance reviews and health indicators) for the public**	
**International best practice examples (benchmarks)**	**New Zealand/Australia:** The freedom of information Act provides a legally enforceable right of the public to assess documents of government departments and most agencies.<br><br>**Ireland:** The Freedom of Information Act 2014 came into effect in October 2014 and repealed the 1997 and 2003 Acts. The 2014 Act now applies to all public bodies, unless they are specifically exempt. It also allows for the Government to prescribe (or designate) other bodies receiving significant public funds, so that the FOI legislation applies to them also. The old legislation continues to apply to any FOI request that was made before the 2014 Act came into effect. It also applies to any subsequent review or appeal. It provides the following statutory rights, (1) A legal right for each person to access information held by a body to which FOI legislation applies known as an *FOI body.* (2) A legal right for each person to have official information relating to himself/herself amended where it is incomplete, incorrect or misleading. (3) A legal right for each person to obtain reasons for decisions affecting himself/herself (Citizens Information, 2014).<br><br>**Norway:** The Freedom of Information Act grants everyone the right of access to case documents, journals and similar registers for any agencies encompassed by the Act. The Act applies to all government agencies,

	municipalities and county authorities. The general rule is that access shall be granted, and exceptions to this rule require legal authority prescribed by or pursuant to law. The Act also contains rules for handling right of access claims and the opportunity to appeal decisions in access matters (Norwegian Ministry of Local Government and Modernisation, 2019).

DOMAIN 10 - MONITORING AND INTELLIGENCE

**MONIT1**	
**Monitoring systems, implemented by the government, are in place to regularly monitor food environments(especially for food composition for nutrients of concern, food promotion to children, and nutritional quality of food in schools and other public sector settings), against codes / guidelines / standards / targets**	
**International best practice examples (benchmarks)**	**Many countries:** have food composition databases available. For example, the New Zealand Institute for Plant & Food Research Limited and the Ministry of Health jointly own the New Zealand Food Composition Database (NZFCD) which is a comprehensive collection of nutrient data in New Zealand containing nutrient information on more than 2600 foods.<br><br>**New Zealand:** A national School and Early Childhood Education Services (ECES) Food and Nutrition Environment Survey was organised in all Schools and ECES across New Zealand in 2007 and 2009 by the Ministry of Health to measure the food environments in schools and ECEs in New Zealand.<br><br>**UK:** In October 2005, the School Food Trust ('the Trust'; now called the Children's Food Trust) was established to provide independent support and advice to schools, caterers, manufacturers and others on improving the standard of school meals. They perform annual surveys, including the latest information on how many children are having school meals in England, how much they cost and how they're being provided.<br><br>**Germany:** The German Nutrition Report published by the DGE on behalf of the Federal Republic of Germany. The Nutrition Reports for the Federal Republic of Germany have been published by the DGE since 1969. Since 1972 it is provided every 4 years by directive of the Federal Ministry of Consumer Protection, Food and Agriculture. The subjects follow topics of current interest (German Nutrition Society).(German Nutrition Society). The Robert-Koch-Institute (RKI), the government's scientific institution in the field of public health, started in 2015 the project 'AdiMon' that collects regular data on influencing (environmental) factors and prevalence of

	childhood obesity, as well as interventions for prevention and health promotion (Robert Koch Institut).<br><br>**The Netherlands:** the progress in product improvement of salt, saturated fat and calories (sugar and/or (saturated) fat) is monitored by the Dutch Insititute of Public Health and Environment (Rijksinstituut voor Volksgezondheid en Milieu (RIVM)) at product level. RIVM uses the product databank (levensmiddelendatabank (LEDA) as basis for which companies have to provide information about product contents (Ministry of Public Health).

**MONIT2**	
**There is regular monitoring of adult and childhood nutrition status and population intakes against specified intake targets or recommended daily intake levels**	
**International best practice examples (benchmarks)**	**USA:** The National Health and Nutrition Examination Survey (NHANES) is a program of studies designed to assess the health and nutritional status of adults and children in the United States. The survey is unique in that it combines interviews and physical examinations (Centres for Disease Control, 2016). (Centres for Disease Control, 2016). The NHANES program began in the early 1960s and has been conducted as a series of surveys focusing on different population groups or health topics. In 1999, the survey became a continuous program that has a changing focus on a variety of health and nutrition measurements to meet emerging needs. The survey examines a nationally representative sample of about 5,000 persons each year. These persons are located in counties across the country, 15 of which are visited each year.<br><br>**The Netherlands:** The Dutch Institute of Public Health and Environment (Rijksinstituut voor Volksgezondheid en Milieu (RIVM)) is appointed by the Ministry of Health to periodically collect data about the food consumption and food condition of the Dutch population in general and of separate population groups via the Food Consumption Survey. Currently, a Food Consumption Survey (Dutch population 1-79 years) is being conducted for the years 2019-2021. Prior Food Consumption Surveys have been conducted for the years 2012-2016 (Dutch population 1-79 years), 2010-2012 (elderly 70+), 2007-2010 (7-69 years), 2005-2006 (2-6 years), 2003 (9-16 years) (National Institute for Public Health and the Environment Ministry of Health, 2011)

MONIT3	
**There is regular monitoring of adult and childhood overweight and obesity prevalence using anthropometric measurements**	
**International best practice examples (benchmarks)**	**UK:** England's National Child Measurement Programme was established in 2006 and aims to measure all children in England in the first (4-5) years and last (10-11 years) of primary school. In 2011-2012, 565 662 children (4-5 years) and 491118 children (10-11 years) were measured (Health and Social Care Information Centre, 2016).<br><br>**Ireland:** The 4th Childhood Obesity Surveillance Initiative report was launched in 2017. COSI collects data from children in primary schools in the Republic of Ireland. The survey is carried out periodically. Data was first collected from children in 2008 in first class and again in 2010 from first class and third class, in 2012 from first, third and fifth classes and in 2015 from first, fourth and sixth class. Trained researchers collected weight, height and waist circumference measurements. These figures were used to examine prevalence of normal weight, overweight, obesity and mean BMI (National nutrition Surveillance Centre).

MONIT4	
**There is regular monitoring of the prevalence of NCD metabolic risk factors and occurrence rates (e.g. prevalence, incidence, mortality) for the main diet-related NCDs**	

**International best practice examples (benchmarks)**	

**MONIT5**	
**Major programs and policies are regularly evaluated to assess their effectiveness and contributions to achieving the goals of the nutrition and health plans**	
**International best practice examples (benchmarks)**	**USA:** The National Institutes for Health (NIH) provide funding for rapid assessments of natural experiments. The funding establishes an accelerated review/award process to support time-sensitive research to evaluate a new policy or program expected to influence obesity related behaviours (e.g., dietary intake, physical activity, or sedentary behaviour) and/or weight outcomes in an effort to prevent or reduce obesity (US National Institutes of Health, 2016).<br><br>**The Netherlands:** The Dutch Insititute of Public Health and Environment (Rijksinstituut voor Volksgezondheid en Milieu (RIVM)) conducted in 2017 a midterm evaluation to calculate the effect of the agreed maximum norms for salt and sugar in the Agreement on Product Improvement (The Dutch Insititute of Public Health and Environment 2017). A midterm evaluation has been performed to calculate the effect of the agreed maximum norms for salt and sugar reduction, and four scenarios have been calculated with the Food Consumption Survey.<br><br>**Ireland:** Under the 'Healthy Weight for Ireland', Obesity Policy and Action Plan 2016-2025, a new Obesity Policy Implementation Oversight Group (OPIOG) was established in October 2017 and a progress report on each recommendation in the OPAP is currently being finalised under the aegis of the OPIOG (Oireachtas, 2019). As set out in Healthy Ireland, integrated health and social impact assessments will be conducted on relevant policy

	areas to support other government departments in contributing towards the prevention of overweight and obesity.

**MONIT6**	
**Progress towards reducing health inequalities or health impacts in vulnerable populations and social and economic determinants of health are regularly monitored.**	
**International best practice examples (benchmarks)**	**New Zealand:** All annual Ministry of Health Surveys report estimates by subpopulations in particular by ethnicity (including Maori and Pacific peoples), by age, by gender, and by New Zealand area deprivation.

DOMAIN 11 - FUNDING AND RESOURCES:

**FUND1**	
**The 'population nutrition' budget, as a proportion of total health spending and/or in relation to the diet-related NCD burden sufficiently contributes to reducing diet-related NCD's.** <br>**(This indicator isn't being included in the online rating process)**	
**International best practice examples (benchmarks)**	**New Zealand:** The total funding for population nutrition was estimated at about $67 million or 0.6% of the health budget during 2008/09 Healthy Eating Healthy Action period. Dietary risk factors account for 11.4% of health loss in New Zealand. <br>**Thailand:** According to the most recent report on health expenditure in 2012 the government greatly increased budget spent on policies and actions related to nutrition (excluding food, hygiene and drinking water control). Total expenditure on health related to nutrition specifically from local governments was 29,434.5 million Baht (7.57% of total health expenditure from public funding agencies), which was ten times over the budget spending on nutrition in 2011. Dietary risk factors account for more than 10% of health loss in Thailand

**FUND2**	
**Government funded research is targeted for improving food environments, reducing obesity, NCDs and their related inequalities**	

**International best practice examples (benchmarks)**	**Australia:** The National Health and Medical Research Council (NHMRC) Act requires the CEO to identify major national health issues likely to arise. The National Health Priority Areas (NHPAs) articulate priorities for research and investment and have been designated by Australian governments as key targets because of their contribution to the burden of disease in Australia. For the 2015-16 Corporate Plan, obesity, diabetes and cardiovascular health are three of these NHPAs.<br><br>**Thailand:** The National Research Council funded more research projects on obesity and diet-related chronic diseases (such as diabetes, cardiovascular diseases and hypertension) in 2014, accountable for almost six times over the research funding in 2013 (from 6,875,028 baht in 2013 to 37,872,416 baht in 2014)<br><br>**Ireland:** The Food Institutional Research Measure (FIRM) is funded by the Department of Agriculture, Food and the Marine and is the primary national funding mechanism for food research in higher education institutions and other public research institutes. Beneficiaries are required to widely disseminate the results of their research. FIRM aims to develop public good technologies that will underpin a competitive, innovative and sustainable food manufacturing and marketing sector. The programme is creating a base of knowledge and expertise in generic technologies that will support a modern, consumer-focused industry and build Ireland's capacity for R&D (Marine, 2017).(Marine, 2017). The Health Research Board (HRB) is a statutory agency under the aegis of the Department of Health. It's the lead agency in Ireland responsible for supporting and funding health research, information and evidence, which aims to improve people's health and to enhance healthcare delivery (Health Research Board, 2016).

**FUND3**	
**There is a statutory health promotion agency in place that includes an objective to improve population nutrition with a secure funding stream**	

**International best practice examples (benchmarks)**	**Australia:** The Victorian Health Promotion Foundation (VicHealth) was the world's first health promotion foundation, established by the Victorian Parliament as part of the Tobacco Act of 1987 (for the first 10 years through a hypothecated tobacco tax) through which the objectives of VicHealth are stipulated. VicHealth continues to maintain bipartisan support.<br><br>**Germany:** The **Federal Centre for Health Education** and the Federal Center for Nutrition disseminate guidelines and health promotion strategies to the general public and stakeholders and multipliers<br><br>**The Netherlands:** The Dutch Nutrition Center (https://www.voedingscentrum.nl/nl/service/over-ons.aspx) is 100% funded by the government and offers consumers and professionals scientific and independent information about a healthy, safe and sustainable food choice. The famous 'Wheel of Five' Guidelines is one of their products.

DOMAIN 12 - PLATFORMS FOR INTERACTION

**PLAT1**	
**There are robust coordination mechanisms across departments and levels of government (national, state and local) to ensure policy coherence, alignment, and integration of food, obesity and diet-related NCD prevention policies across governments.**	
**International best practice examples (benchmarks)**	**Finland:** The Finnish National Nutrition Council is an inter-governmental expert body under the Ministry of Agriculture and Forestry with advisory, coordinating and monitoring functions. It is composed of representatives elected for three-year terms from government authorities dealing with nutrition, food safety, health promotion, catering, food industry, trade and agriculture (World Cancer Research Fund, 2016a).<br><br>**Malta:** Based on the Healthy Lifestyle Promotion and Care of NCDs Act (2016), Malta established an inter-ministerial Advisory Council on Healthy Lifestyles in August 2016 to advise the Minister of Health on any matter related to healthy lifestyles. In particular, the Advisory Council advises on a life course approach to physical activity and nutrition, and on policies,

	action plans and regulations intended to reduce the occurrence of NCDs. The prime minister appoints the chair and the secretary of the Advisory Council, while the ministers of education, health, finance, social policy, sports, local government, and home affairs appoint one member each (World Cancer Research Fund, 2016a).<br><br>**Australia:** There are several forums and committees for the purpose of strengthening food regulation with representation from New Zealand and Health Ministers from Australian States and Territories, the Australian Government, as well as other Ministers from related portfolios (e.g. Primary Industries). Where relevant, there is also representation from the Australian Local Government Association.<br><br>**Ireland:** The Department of Health, through 'A Healthy weight for Ireland', Obesity Policy and Action Plan 2016 - 2025, will provide leadership, engage and co-ordinate multi-sectorial action and implement best practice in the governance of the OPAP. The department of health and safefood are taking action to establish a multi stakeholder partnership to share knowledge and initiative on healthy weight initiatives (Department of Health, 2016a)

**PLAT2**	
**There are formal platforms (with clearly defined mandates, roles and structures) for regular interactions between government and the commercial food sector on the implementation of healthy food policies and other related strategies**	
**International best practice examples (benchmarks)**	**UK:** The UK 'Responsibility Deal' was a UK government initiative to bring together food companies and NGOs to take steps (through voluntary pledges) to address NCDs. It was chaired by the Secretary of State for Health and included senior representatives from the business community (as well as NGOs, public health organisations and local government). A number of other subgroups were responsible for driving specific programs relevant to the commercial food sector.<br><br>**Norway:** The letter of intent (Memorandum of Understanding, MoU) for facilitating a healthier diet in the population is a signed agreement between the Norwegian health authorities and food industry (food and trade organizations, food and beverage manufacturers, food retailers and food service industry) in a *Partnership for a healthier diet.* The MoU was signed in 2016 and lasts until 2021. The agreement contains specific

	quantitative goals related to reducing the intake of salt, added sugar and saturated fat, and increasing the intake of fruits and berries, vegetables, whole grain foods, fish and seafood in the population. The Partnership is organized in a Coordination group with representatives from the main partners including the health authorities. The Coordination group reports to the Minister's food industry group (lead by the Minister for the Elderly and Public Health) that ensures dialogue and political focus on the areas of action. The Coordination group is assisted by a Secretariat organized by the Directorate of Health. A Reference group of scientists within nutrition, food technology, consumer behaviour, psychology and marketing provide expert advice to the coordination group (Helsedirektoratet, 2016).

**PLAT3**	
**There are formal platforms (with clearly defined mandates, roles and structures) for regular interactions between government and civil society on the development, implementation and evaluation of healthy food policies and other related strategies.**	
**International best practice examples (benchmarks)**	**Brazil:** The National Council of Food and Nutrition Security (CONSEA) is a formal advisory platform made up of civil society (2/3) and government reps (1/3). It is a participatory instrument for designing, suggesting, implementing and evaluating food and nutritional security policy (CONSEA). Through CONSEA, civil society has been able to influence policy directions more directly. CONSEA supported Congress to pass a bill obliging local governments to buy at least 30% of the food destined for school meals from small-scale farmers.

**PLAT4**

The governments work with a system-based approach with (local and national) organisations/partners/groups to improve the healthiness of food environments at a national level.	
**International best practice examples (benchmarks)**	**New Zealand:** Healthy Families NZ is a large-scale initiative that brings community leadership together in a united effort for better health. It aims to improve people's health where they live, learn, work and play, in order to prevent chronic disease. Led by the Ministry of Health, the initiative will focus on ten locations in New Zealand in the first instance. It has the potential to impact the lives of over a million New Zealanders. The Government has allocated $40 million over four years to support Healthy Families NZ (Ministry of Health New Zealand, 2016).<br><br>**Australia:** Healthy together Victoria in Australia focuses on addressing the underlying causes of poor health in children's settings, workplaces and communities by encouraging healthy eating and physical activity and reducing smoking and harmful alcohol use. Healthy Together Victoria incorporates policies and strategies to support good health across Victoria, as well as locally-led Healthy Together Communities. The initiative was originally jointly funded by the State Government of Victoria and the Australian Government through the National Partnership Agreement on Preventive Health (Government of South Australia, 2016). It is unclear at this stage whether funding for Healthy Together Victoria will continue or not.

DOMAIN 13 - HEALTH IN ALL POLICIES

HIAP1	
**There are processes in place to ensure that population nutrition, health outcomes and reducing health inequalities or health impacts in vulnerable populations are considered and prioritised in the development of all government policies relating to food**	
**International best practice examples (benchmarks)**	**Slovenia:** A Health Impact Assessment was undertaken in Slovenia to assess the health effects of agricultural policy at national level policy analysis, rapid appraisal workshops with stakeholders from a range of backgrounds, review of research evidence relevant to the agricultural policy, analysis of Slovenian data for key health-related indicators, a report on the findings to a key cross-government group and evaluation (Lock, 2003).

	**Ireland: Step 9** of the ‘ A Healthy Weight for Ireland’÷¿ Obesity Policy and Action Plan 2016-2025 aims to, allocate resources according to need, in particular to those population groups most in need of support in the prevention and management of obesity, with particular emphasis on families and children during the first 1,000 days of life. The priority actions to commence in first year were to, assess the needs of vulnerable groups as the basis of allocation of resources for preventative and treatment services for children and adults (Department of Health, 2016b). The Healthy Ireland fund was established in 2017 with an allocation of €5 million and with additional allocations of €5 million in 2018 and 2019. The first round of the Fund was distributed through Local Community Development Committees, Children and Young Person’s Services Committees and statutory organisations (Oireachtas, 2019).

**HIAP2**	
**There are processes e.g. Health Impact Assessment’s (HIAs) to assess and consider health impacts during the development of other non-food policies.**	
**International best practice examples (benchmarks)**	**South Australia:** Established in 2007, the implementation of Health in All Policies (HiAP) in South Australia has been supported by a high-level mandate from central government, an overarching framework which is supportive of a diverse program of work, a commitment to work collaboratively and in partnership across agencies, and a strong evaluation process. The government has established a dedicated HiAP team within South Australia Health to build workforce capacity and support Health Lens Analysis projects. Since 2007, the South Australian HiAP approach has evolved to remain relevant in a changing context. However, the purpose and core principles of the approach remain unchanged. There

	have been five phases to the work of HiAP in South Australia between 2007 and 2016: 1) Prove concept and practice emerges (2007-2008), 2) Establish and apply methodology (2008-2009), 3) Consolidate and grow (2009-2013), 4) Adapt and review (2014) and 5) Strengthen and systematize (2015-2016).

Bibliography

ADMINISTRATION, F. A. D. 2019. FDA Nutrition Innovation Strategy. .

ADVERTISING STANDARDS AUTHORITY. 2018. *Food: HFSS Media Placement* [Online]. Available: https://www.asa.org.uk/advice-online/food-hfss-media-placement.html [Accessed].

BAE, S. G., J.Y. KIM, K.Y. KIM, S.W. PARK, J. BAE, AND W.K. LEE, 2012. Changes in dietary behavior among adolescents and their association with government nutrition policies in Korea, 2005-2009. J Prev Med Public Health, 45(1): p. 47-59.

BIRO, A. 2015. Did the junk food tax make the Hungarians eat healthier? Food Policy. 54: p. 107-115.

BROADCASTING AUTHORITY OF IRELAND 2013. BAI Children's Commercial Communications Code.

BROADCASTING AUTHORITY OF IRELAND. 2019. *Codes and Standards* [Online]. Available: https://www.bai.ie/en/codes-standards/#al-block-5 [Accessed].

CENTRES FOR DISEASE CONTROL 2016. National Health and Nutrition Examination Survey. Available from: http://www.cdc.gov/nchs/nhanes.htm.

CHANTAL, J. A. H., S., 2017. Development of a new front-of-pack nutrition label in France: the five colour Nutri-Score.

CITIZENS INFORMATION 2014. Freedom of information. [online] available: https://www.citizensinformation.ie/en/government_in_ireland/national_government/standards_and_accountability/freedom_of_information.html.

COLCHERO, M. A., B.M. POPKIN, J.A. RIVERA, AND S.W. NG, 2016. Beverage purchases from stores in Mexico under the excise tax on sugar sweetened beverages: observational study. BMJ 352: p. h6704.

COLRUYT GROUP. 2018. *What is the Nutri-Score? [online] available: https://nutriscore.colruytgroup.com/colruytgroup/en/about-nutri-score/* [Online]. [Accessed].

COMMONWEALTH OF AUSTRALIA 2016. Health Star Rating System. Available from: http://healthstarrating.gov.au/internet/healthstarrating/publishing.nsf/content/home.

CONSEA Building up the National Policy and System for Food and Nutrition Security: the Brazilian experience. Available from: https://www.fao.org.br/download/Seguranca_Alimentar_Ingles.pdf.

CORREA, T., FIERRO, C., REYES, M., DILLMAN CARPENTIER, F. R., TAILLIE, L. S. & CORVALAN, C. 2019. "Responses to the Chilean law of food labeling and advertising: exploring knowledge, perceptions and behaviors of mothers of young children". *International Journal of Behavioral Nutrition and Physical Activity,* 16**,** 21.

CORVALÁN C, R. M., GARMENDIA ML, UAUY R, 2018. Structural responses to the obesity and non-communicable diseases epidemic: Update on the Chilean law of food labelling and advertising. Obesity Reviews. 2018;1–8. https://

doi.org/10.1111/obr.12802.

DEPARMENT OF HEALTH 2013. Guide to creating a front of pack (FoP) nutrition label for pre-packed products sold through retail outlets. Available from: https://www.gov.uk/government/uploads/system/uploads/attachment_data/file/300886/2902158_FoP_Nutrition_2014.pdf.

DEPARTMENT OF HEALTH 2013. Healthy Ireland - a framework for improved health and wellbeing 2013 - 2025. Dublin: Department of Health.

DEPARTMENT OF HEALTH. 2014. *National salth reduction initiative corporate achievements 2009-2014.* [Online]. Available: https://www1.nyc.gov/assets/doh/downloads/pdf/cardio/nsri-corporate-commitments.pdf [Accessed].
DEPARTMENT OF HEALTH 2016a. A Healthy Weight for Ireland. Obesity Policy and Action Plan: 2016-2025.
DEPARTMENT OF HEALTH 2016b. A Healthy Weight for Ireland: Obesity Policy and Action Plan 2016 – 2025.
DEPARTMENT OF HEALTH 2019a. Healthy Ireland Fund 2019- 2021. Expression of Interest Guidelines. [online] Accessed on https://drive.google.com/file/d/1rqVbr_YILdi12GqeGNgPnILPaEbrCBKB/view.
DEPARTMENT OF HEALTH 2019b. Taoiseach and Ministers launch Healthy Ireland campaign. [online] Accessed on https://www.gov.ie/en/news/20cff4-taoiseach-and-health-ministers-to-launch-2019-healthy-ireland-campai/.
DEPARTMENT OF HEALTH AND MENTAL HYGIENE Food Preparation and Food Establishments (Article 81, Section 49) - Sodium Warning. Available from: http://rules.cityofnewyork.us/content/food-preparation-and-food-establishments-article-81-section-49-sodium-warning-0.
DOWNS, S., BLOEM, M AND GRAZIOSE, MM., 2017. Salt and the city: a preliminary examination of New York City's sodium warning labels. .
FOOD SAFETY AUTHORITY OF IRELAND 2011. Scientific Recommendations for Healthy Eating Guidelines in Ireland [Online]. Dublin: Food Safety Authority of Ireland.
FOOD STANDARDS AUSTRALIA NEW ZEALAND 2013. Stakeholder Engagement Strategy 2013-2016. FSANZ New Zealand: Wellington.
FUNDO NACIONAL DE DESINVOLVIMENTO DA EDUCACAO 2016. Sobre o PNAE. Available from: http://www.fnde.gov.br/programas/alimentacao-escolar.
GERMAN NUTRITION SOCIETY. *Nutrition Reports. [online] available: https://www.dge.de/en/nutrition-reports/* [Online]. [Accessed].
GLOBAL AGRICULTURAL INFORMATION NETWORK 2018. Update on Chilean Advertising of Packaged Foods and Beverages. [online] available: https://apps.fas.usda.gov/newgainapi/api/report/downloadreportbyfilename?filename=Update%20on%20Chilean%20Advertising%20of%20Packaged%20Foods%20and%20Beverages%20_Santiago_Chile_2-14-2018.pdf.
GOVERNMENT OF CANADA 2016. Symposium on Sodium Reduction in Foods Meeting Report. Available from: https://www.canada.ca/en/services/health/symposium-sodium-reduction-foods-meeting-report.html.
GOVERNMENT OF SOUTH AUSTRALIA 2016. Health in All Policies: The South Australian Approach. Available from: http://www.sahealth.sa.gov.au/wps/wcm/connect/public+content/sa+health+internet/health+reform/health+in+all+policies.
GREATER LONDON AUTHORITY 2019. TfL junk food ads ban will tackle child obesity. [online] available: https://www.london.gov.uk/what-we-do/business-and-economy/food/tfl-junk-food-ads-ban-will-tackle-child-obesity.
HEALTH AND SOCIAL CARE INFORMATION CENTRE 2016. National Child Measurement Programme. Available from: http://www.hscic.gov.uk/ncmp.
HEALTH COUNCIL OF THE NETHERLANDS 2015. Dutch dietary guidelines 2015. [online] available: file:///C:/Users/charlottegriffin/Downloads/advisory-report-dutch-dietary-guidelines-2015.pdf.
HEALTH RESEARCH BOARD 2016. Research Evidence Action. HRB Strategy 2016-2020.
HEALTH SERVICE EXECUTIVE 2019. HSE Vending Policy
HEALTHY EATING ADVISORY SERVICE 2017. Improving wellbeing through healthy eating.
HEALTHY IRELAND 2013-2025. A framework for improved health and wellbeing 2013-2025.

HEALTHY IRELAND 2016. Healthy Food for Life. Revised healthy eating guidelines and Food Pyramid rationale. .

HEALTHY IRELAND 2017. Nutrition Standards for School Meals

HELSEDIREKTORATET 2016. Partnership for a healthier diet. [online] available: https://www.helsedirektoratet.no/english/partnership-for-a-healthier-diet.

HOFMAN, K. J. & TOLLMAN, S. M. 2013. Population health in South Africa: a view from the salt mines. *The Lancet Global Health,* 1**,** e66-e67.

KELLY, P. M., A. DAVIES, A.J. GREIG, AND K.K. LEE, 2016. Obesity Prevention in a City State: Lessons from New York City during the Bloomberg Administration. Front Public Health, 4: p. 60.

KENT, M. P., DUBOIS, L. & WANLESS, A. J. I. J. O. P. O. 2011. Food marketing on children's television in two different policy environments. 6**,** e433-441.

LEDERER, A., C.J. CURTIS, L.D. SILVER, AND S.Y. ANGELL, 2014. Toward a healthier city: nutrition standards for New York City government. Am J Prev Med 46(4): p. 423-8.

LOCK, K., M. GABRIJELCIC-BLENKUS, M. MARTUZZI, P. OTOREPEC, P. WALLACE, C. DORA, J.M. ZAKOTNIC, 2003. Health impact assessment of agriculture and food policies: lessons learnt from the Republic of Slovenia. Bull World Health Organ, 81(6): p. 391-8.

MARINE, D. O. A. F. A. 2017. *Food Institutional Research Measure* [Online]. Available: https://www.agriculture.gov.ie/research/competitivenationalprogrammes/foodinstitutionalresearchmeasurefirm/ [Accessed].

MASSRI, C., SUTHERLAND, S., KÄLLESTÅL, C. & PEÑA, S. J. A. J. O. P. H. 2019. Impact of the Food-Labeling and Advertising Law Banning Competitive Food and Beverages in Chilean Public Schools, 2014–2016. 109**,** 1249-1254.

MATBRANSJENS FAGLIGE UTVALG 2019. Guidelines for marketing food and drink aimed at children. [online] available: http://www.mfu.as/85197-Retningslinjer-for-markedsforing-oppdatert-1-10-2019.

MCAVOY, H., BERGIN, D., KIERNAN, R., KEATING, T., 2018. Consultation on the development of a Healthy Workplaces Framework for Ireland: A report prepared for the Department of Health. Dublin: Institute of Public Health in Ireland.

MFU. 2016. *Code for marketing of food and drink aimed at children updated 1/9-2016* [Online]. Available: http://www.mfu.as/44552-Code-for-marketing-of-food-and-drink-aimed-at-children-updated-1-9-2016 [Accessed].

MINISTRY OF EDUCATION, N. F. F. E. D. 2016. Resolution No 26 of 17 June 2013 (Ministério da Educação, Fundo Nacional de Desenvolvimento da Educação, Resolução No 26 de 17 de junho de 2013). Ministry of Education: Brazil.

MINISTRY OF FOOD AND DRUG SAFETY 2017. The Special Act on the Safety Management of Children's Dietary Life. Available from: https://www.mfds.go.kr/eng/index.do?nMenuCode=66.

MINISTRY OF HEALTH BRAZIL 2011. Health Surveillance Secretariat: Health situation analysis department. Strategic action plan to tackle noncommunicable diseases in Brazil 2011-2022. Ministry of Health: Brazil.

MINISTRY OF HEALTH BRAZIL 2014. Dietary guidelines for the Brazilian population. Available from: http://www.foodpolitics.com/wp-content/uploads/Brazilian-Dietary-Guidelines-2014.pdf.

MINISTRY OF HEALTH NEW ZEALAND 2016. Healthy Families NZ. Available from: http://www.health.govt.nz/our-work/preventative-health-wellness/healthy-families-nz.

MINISTRY OF HEALTH SOUTH AFRICA 2013. Strategic Plan for the Prevention and Control of Non-Communicable Diseases 2013-17. Available from: http://www.hsrc.ac.za/uploads/pageContent/3893/NCDs%20STRAT%20PLAN%20%20CONTENT%208%20april%20proof.pdf.

MINISTRY OF PUBLIC HEALTH, W. A. S. Food Database. [online] available: https://www.levensmiddelendatabank.nl/Security/Login.aspx?ReturnUrl=%2f.

MONTEIRO, C. A., G. CANNON, J.C. MOUBARAC, A.P. MARTINS, C.A. MARTINS, J. GARZILLO, P.C. JAIME, 2015. Dietary guidelines to nourish humanity and the planet in the twenty-first century. A blueprint from Brazil. Public Health Nutr, 18(13): p. 2311-22.

MORLEY, B., SCULLY, M., MARTIN, J., NIVEN, P., DIXON, H. & WAKEFIELD, M. 2013. What types of nutrition menu labelling lead consumers to select less energy-dense fast food? An experimental study. *Appetite,* 67**,** 8-15.

NATIONAL AGREEMENT TO IMPROVE PRODUCT COMPOSITION 2017. Available from: http://www.akkoordverbeteringproductsamenstelling.nl/dsresource?type=pdf&disposition=inline&objectid=rivmp:303682&versionid=&subobjectname=.

NATIONAL INSTITUTE FOR HEALTH AND WELFARE 2019. EATING TOGETHER -food recommendations for families with children.

NATIONAL INSTITUTE FOR PUBLIC HEALTH AND THE ENVIRONMENT MINISTRY OF HEALTH, W. A. S. 2011. Overview of food consumption surveys. [online] available: https://www.rivm.nl/voedselconsumptiepeiling/overzicht-voedselconsumptiepeilingen.

NATIONAL NUTRITION COUNCIL 2017. Eating and learning together – recommendations for school meals.

NATIONAL NUTRITION COUNCIL 2018. HEALTH AND JOY FROM FOOD - meal recommendations for early childhood education and care.

NATIONAL NUTRITION SURVEILLANCE CENTRE Childhood Obesity Surveillance Initiative (COSI). [online] available: http://www.ucd.ie/nnsc/research/ourresearch/childhoodobesitysurveillanceinitiativecosi/.

NETHERLANDS, G. O. T. 2019. The National Prevention Agreement.

NETHERLANDS NUTRITION CENTRE 2017. Guidelines for Healthier Canteens Fact sheet. [online] available: https://mobiel.voedingscentrum.nl/Assets/Uploads/voedingscentrum/Documents/Professionals/Pers/Factsheets/English/Fact%20sheet%20Guidelines%20for%20Healthier%20Canteens.pdf.

NEW YORK CITY HEALTH 2017 National Salt Reduction Initiative (NSRI). Available from: https://www1.nyc.gov/site/doh/health/health-topics/national-salt-reduction-initiative.page.

NG, S. W., RIVERA, J. A., POPKIN, B. M. & COLCHERO, M. A. J. P. H. N. 2019. Did high sugar-sweetened beverage purchasers respond differently to the excise tax on sugar-sweetened beverages in Mexico? 22**,** 750-756.

NORWEGIAN MINISTRY OF LOCAL GOVERNMENT AND MODERNISATION 2019. Fourth Norwegian Action Plan Open Government Partnership (OGP) 2019 - 2021.

NUTRITION RESOURCE CENTRE 2017. Ontario's Healthy Menu Choices Act In Effect. [online] available https://opha.on.ca/Nutrition-Resource-Centre/News/NRC-News/2017/Ontario%E2%80%99s-Healthy-Menu-Choices-Act-In-Effect.aspx.

NYC HEALTH DEPARTMENT 2019. National Salt and Sugar Reduction Initiative (NSSRI). .

OFFICE OF THE FEDERAL REGISTER 2013. Food Labeling: Nutrition Labeling of Standard Menu Items in Restaurants and Similar Retail Food Establishments. Available from: https://www.federalregister.gov/regulations/0910-AG57/food-labeling-nutrition-labeling-of-standard-menu-items-in-restaurants-and-similar-retail-food-estab.

OIREACHTAS 2019. Obesity Strategy Dáil Éireann Debate, Tuesday - 12 March 2019. [online] Accessed https://www.oireachtas.ie/en/debates/question/2019-03-12/275/.

PARLIMENT UK 2017. Financial Statement. [online] available on https://hansard.parliament.uk/Commons/2017-03-08/debates/9128238B-4A1C-4EEA-A6F0-6FEB0B5024D8/FinancialStatement#contribution-7B30FFC0-4B7A-4D63-8075-3C7F83EDC5.

PETERS, S., DUNFORD, E., WARE, L., HARRIS, T., WALKER, A., WICKS, M., VAN ZYL, T., SWANEPOEL, B., CHARLTON, K. & WOODWARD, M. J. N. 2017. The sodium content of processed foods in South Africa during the introduction of mandatory sodium limits. 9**,** 404.

POBAL 2016. Healthy Ireland Fund. [online] Accessed on https://www.pobal.ie/programmes/healthy-ireland-fund/.

PUBLIC HEALTH ENGLAND 2017. Sugar Reduction: Achieving the 20% A technical report outlining progress to date, guidelines for industry, 2015 baseline levels in key foods and next steps. .

PUBLIC HEALTH ENGLAND 2018. Sugar Reduction Programme. Progress made by industry in the first year.

PUBLIC HEALTH ENGLAND 2019. Sugar reduction: Report on progress between 2015 and 2018. September 2019.

RATHBONE GREENBANK INVESTMENTS 2019. The UK sugar tax: one year on. [online] available: https://www.rathbonegreenbank.com/insight/uk-sugar-tax-one-year.

ROBERT KOCH INSTITUT. *The AdiMon Indicator System. [online] available: https://www.rki.de/EN/Content/Health_Monitoring/HealthSurveys/AdiMon/AdiMon_inhalt.html* [Online]. [Accessed].

RØYNSTRAND, S. 2017. *Spiser mosjonister og supermosjonister sunt?: En kartlegging av kostholdet til mannlige utholdenhetsmosjonister og supermosjonister.* Universitetet i Agder; University of Agder.

SAUDI FOOD & DRUF AUTHORITY 2018. SFDA launches Healthy Food Regulation Strategy. [online] available: https://www.sfda.gov.sa/en/food/news/Pages/f11-9-2018a1.aspx.

STANDARDS IN PUBLIC OFFICE COMMISSION 2019. 2018 Code of Conduct for persons carrying on lobbying acitivities. [online] Accessed on https://www.lobbying.ie/media/6119/code-of-conduct-english-final-version-for-web.pdf.

STATE SERVICES COMMISSION Best Practice Guidelines for Departments Responsible for Regulatory Processes with Significant Commercial Implications. Available from: http://www.ssc.govt.nz/resources/2814/all-pages.

THE CENTRAL GOVERNMENT FOR THE NETHERLANDS 2014. Agreed improvement product composition salt, saturated fat, sugar. [online] available: https://www.rijksoverheid.nl/documenten/convenanten/2014/01/23/akkoord-verbetering-productsamenstelling-zout-verzadigd-vet-suiker.

THE CHIP GROUP 2016. The Chip Group: Helping you make better chips. Available from: http://blog.thechipgroup.co.nz/.

THE DUTCH INSITITUTE OF PUBLIC HEALTH AND ENVIRONMENT 2017. Agreed improvement product composition salt, saturated fat, sugar. [online] available: https://www.rijksoverheid.nl/documenten/convenanten/2014/01/23/akkoord-verbetering-productsamenstelling-zout-verzadigd-vet-suiker.

THE ORGANISATION FOR ECONOMIC CO-OPERATION AND DEVELOPMENT 2019. OECD Reviews of Public Health: Chile: A Healthier Tomorrow, OECD Publishing, Paris.

https://doi.org/10.1787/9789264309593-en.

US NATIONAL INSTITUTES OF HEALTH 2016. Time-Sensitive Obesity Policy and Program Evaluation (R01). Available from: http://grants.nih.gov/grants/guide/pa-files/PAR-12-257.html.

VEERMAN, J. L. A. L. J. C. 2013 Removing the GST exemption for fresh fruits and vegetables could cost lives. Med J Aust, 199(8): p. 534-5.

WORLD CANCER RESEARCH FUND 2016a. NOURISHING Framework - Harness food supply chain. Available from: http://www.wcrf.org/int/policy/nourishing-framework/harness-food-supply-chain.

WORLD CANCER RESEARCH FUND 2016b. NOURISHING Framework - Improving the food supply. Available from: http://www.wcrf.org/int/policy/nourishing-framework/improve-food-supply.

WORLD CANCER RESEARCH FUND 2016c. NOURISHING Framework - Nutrition labels. Available from: http://www.wcrf.org/int/policy/nourishing-framework/nutrition-labels.

WORLD CANCER RESEARCH FUND 2016d. NOURISHING Framework - Offer healthy foods. Available from: http://www.wcrf.org/int/policy/nourishing-framework/offer-healthy-foods.

WORLD CANCER RESEARCH FUND 2016e. NOURISHING Framework - Restrict food marketing. Available from: http://www.wcrf.org/int/policy/nourishing-framework/restrict-food-marketing.
WORLD CANCER RESEARCH FUND 2016f. NOURISHING Framework - Use economic tools. Available from: http://www.wcrf.org/int/policy/nourishing-framework/use-economic-tools.
WORLD CANCER RESEARCH FUND 2016 NOURISHING Framework - Set retail environment incentives. Available from: http://www.wcrf.org/int/policy/nourishing-framework/set-retail-environment-incentives.
WORLD CANCER RESEARCH FUND 2018. NOURISHING framework. Use economic tools to address food affordability and purchase incentives. [online] available on https://www.wcrf.org/sites/default/files/Use-economic-tools.pdf.
WORLD HEALTH ORGANISATION 2017. France becomes one of the first countries in Region to recommend colour-coded front-of-pack nutrition labelling system.
WORLD PUBLIC HEALTH NUTRITION ASSOCIATION UPDATE TEAM 2014. Brazilian dietary guidelines. At last! Guidelines based on food and meals!. Available from: http://wphna.org/wp-content/uploads/2015/01/WN-2014-05-12-1050-1051-Update-Guia.pdf.

The Healthy Food Environment Policy Index (FOOD-EPI)

Introduction

Overweight, obesity and nutrition-related non-communicable diseases have risen dramatically in the past decennia, caused by changes in dietary and physical activity patterns. The current food environment (e.g. easy availability of energy-dense, fat- and sugar-rich, and ultra-processed foods) is one of the key factors contributing to this public health problem. Governmental policies have the potential to improve these food environments, making a healthy choice easier. Our research will therefore answer the question:

'What EU-level policies do exist that have a (potential) positive influence on the food environment?'

Aim

The aim of our research is:

(1) To provide an overview of EU public policies (i.e. policies developed by the European Commission and that apply to all EU member states) with a direct or indirect (potential) influence on food environments and;

(2) To identify implementation gaps and policy priorities. We use the Food Environment Policy Index (Food-EPI) developed by the International Network for Food and Obesity / Non-communicable Diseases Research, Monitoring and Action Support (INFORMAS) for our analysis (https://www.informas.org/modules/public-sector/). This is a tool to assess the extent of implementation of government policies and actions for creating healthy food environments.

Scope of our research

Globally, the Food-EPI has already been applied in more than twenty countries. For instance, reports can be found about Australia (EPI: https://preventioncentre.org.au/wp-content/uploads/2015/10/Food-Policy-Index-Progress-Update-2019-Victoria-FINAL-002.pdf) and New-Zealand (https://figshare.com/articles/Executive_Summary_Benchmarking_Food_Environments_Progress_by_the_New_Zealand_Government_on_Implementing_Recommended_Food_Environment_Policies_and_Prioritised_Recommendations_2017_/5673481).

This research will apply the Food-EPI to evaluate policies influencing food environments as developed by the European Commission. Similar studies will be conducted at national level in several European countries in the upcoming years as part of the PEN-project, such as Ireland, Germany, the Netherlands, Norway and Poland.

Collection of relevant policy documents and evidence of implementation at EU level

The Food-EPI consists of 13 domains, including 47 indicators, which reflect the extent to which actions and policies for creating healthy food environments have been developed and implemented. **For each of the 47 Food-EPI indicators, evidence for the existence and degree of implementation of policies has been collected by us as researchers, by searching for and reading EU policy documents. All policies at the EU level with a potential influence on the food environment that we came across in our search have been summarized in this "evidence report" under the heading of the Food-EPI indicators.**

We used several main sources to search for the relevant policy documents, like the European Commission's websites:

- European Commission> Live, work, travel in the EU> Public Health> Nutrition and physical activity overview: https://ec.europa.eu/health/nutrition_physical_activity/overview_en

- JRC Health knowledge Gateway: https://ec.europa.eu/jrc/en/health-knowledge-gateway/promotion-prevention/nutrition
- EUR-Lex: https://eur-lex.europa.eu/homepage.html
- Organisation and governance of the European Commission: https://ec.europa.eu/info/about-european-commission_en

Via these websites we found information and links to additional useful documents. Examples of these documents are the:

- The EU Action Plan on Childhood Obesity 2014-2020
- Initiatives on Nutrition and Physical Activity 2019 (https://ec.europa.eu/health/sites/health/files/nutrition_physical_activity/docs/2019_initiatives_npa_en.pdf)
- DG Sante's Strategic Plan 2016-2020

The Strategic Plan of DG Health and Food Safety refers to the Treaty on the Functioning of the European Union, which shapes the EU's degree of influence in health and food policies, saying that Member States are responsible for the definition of their health policy and for the organization and delivery of health services and medical care. DG Health and Food Safety therefore states that EU action is mainly linked to incentive measures, e.g. raising awareness to prevent chronic disease and promote good health and cooperation measures. However, one of its missions is to 'improve and protect human health'.

EU LEGAL INSTRUMENTS[1]

The term European legal instruments refers to the instruments available to the European institutions to carry out their tasks. The instruments listed in Article 288 of the Treaty on the Functioning of the European Union (TFEU) are:

	**Binding in their entirety**	**Binding to the results to be achieved**	**Non-binding**
**Directly applicable in all countries**	Regulations		
**Have to be transposed into the national legal framework**		Directives	
**Directly applicable to whom they are addressed**	Decisions		
**No legal obligation on those to whom it is addressed**			Opinions<br><br>Recommendations

- **Regulations:** these are binding in their entirety and directly applicable in all EU countries;
- **Directives:** these bind the EU countries as to the results to be achieved; they have to be transposed into the national legal framework and thus leave margin for manoeuvre as to the form and means of implementation;
- **Decisions:** these are fully binding on those to whom they are addressed and are directly applicable.
- **Recommendations:** these are non-binding, declaratory instruments. A recommendation allows the institutions to make their views known and to suggest a line of action without imposing any legal obligation on those to whom it is addressed.
- **Opinions:** these are non-binding, declaratory instruments. An "opinion" is an instrument that allows the institutions to make a statement in a non-binding fashion, in other words without imposing any legal obligation on those to whom it is addressed.

Furthermore, Article 290 of the TFEU introduces the possibility for the European legislator to delegate to the Commission the power to adopt **non-legislative acts of general scope** which supplement or amend non-essential elements of legislative acts.

In addition to the instruments listed in Article 288 of the TFEU, practice has led to the development of a whole series of other documents: **interinstitutional agreements, resolutions, conclusions, communications, green papers and white papers.**

[1] https://eur-lex.europa.eu/summary/glossary/community_legal_instruments.html And https://europa.eu/european-union/eu-law/legal-acts_en

Under the Common Foreign and Security Policy, specific legal instruments are used, such as EU **actions and positions.**

- **Interinstitutional agreements** regulate certain aspects of consultation and cooperation between the EU institutions and are the product of a consensus between them — i.e. they constitute a form of **joint rules of procedure**.[2]
- **Council conclusions:** are adopted after a debate during a Council meeting. They can contain a political position on a specific topic. It is important to distinguish between **Council conclusions** and **presidency conclusions**. Council conclusions are issued by the Council while presidency conclusions only express the position of the presidency and do not engage the Council.[3]
- **Council resolutions**: usually set out future work foreseen in a specific policy area. They have no legal effect but they can invite the Commission to make a proposal or take further action. If the resolution covers an area that is not entirely an area of EU competency, it takes the form of a **'resolution of the Council and the representatives of the governments of the member states'.**[4]
- **A Communication is a policy document with no mandatory authority.** [5]The Commission takes the initiative of publishing a Communication when it wishes to set out its own thinking on a topical issue. A Communication has no legal effect.
- **WHITE PAPER:** documents containing proposals for European Union (EU) action in a specific area. In some cases, they follow on from a Green Paper published to launch a consultation process at EU level. The purpose of a White Paper is to launch a debate with the public, stakeholders, the European Parliament and the Council in order to arrive at a political consensus.
- **GREEN PAPER:** documents published by the European Commission to stimulate discussion on given topics at European level. They invite the relevant parties (bodies or individuals) to participate in a consultation process and debate on the basis of the proposals they put forward. Green Papers may give rise to legislative developments that are then outlined in White Papers.

---

[2] https://eur-lex.europa.eu/legal-content/EN/TXT/?uri=LEGISSUM:010302_1
[3] https://www.consilium.europa.eu/en/council-eu/conclusions-resolutions/
[4] https://www.consilium.europa.eu/en/council-eu/conclusions-resolutions/
[5] https://ec.europa.eu/civiljustice/glossary/glossary_en.htm

DOMAIN 1 - FOOD COMPOSITION

Food composition targets/standards/restrictions for processed foods: This domain concerns the extent to which the EU stimulated/proposed/developed/implemented systems to ensure that, where practicable, processed foods minimise the energy density and the nutrients of concern (salt, saturated fat, trans fat, added sugar)

COMP1: Food composition targets/standards/restrictions have been established by the EU for the content of the nutrients of concern (trans fats, added sugars, salt, saturated fat) in industrially processed foods, in particular for those food groups that are major contributors to population intakes of those nutrients of concern

Definitions and scope of COMP 1

- Includes packaged foods manufactured within the EU countries or manufactured overseas and imported to the EU countries for sale.
- Includes packaged, ready-to-eat meals sold in supermarkets.
- Includes mandatory or voluntary targets, standards (e.g., reduce by X%, maximum mg/g per 100g or per serving).
- Includes legislated ban on nutrients of concern.
- Excludes legislated restrictions related to other ingredients (e.g. additives).
- Excludes mandatory food composition regulation related to micronutrients e.g. vitamins, minerals (e.g. folic acid or iodine fortification)
- Excludes food consumption standards/targets for fibre, healthy ingredients like fruits and vegetables
- Excludes food composition of ready-to-eat meals sold in food service outlets (see COMP2)
- Excludes general guidelines advising food companies to reduce nutrients of concern.
- Excludes the provision of resources or expertise to support individual food companies with reformulation.
- Industrially processed foods are the processed and ultra-processed foods according to the **NOVA classification** (please find the complete definitions here: https://world.openfoodfacts.org/nova):
- Processed foods, such as bottled vegetables, canned fish, fruits in syrup, cheeses and freshly made breads, are made essentially by adding salt, oil, sugar or other substances from Group 2 (processed culinary ingredients) to Group 1 (unprocessed or minimally processed) foods.

  Ultra-processed foods, such as soft drinks, sweet or savoury packaged snacks, reconstituted meat products and pre-prepared frozen dishes, are not modified foods but formulations made mostly or entirely from substances derived from foods and additives, with little if any intact Group 1 (unprocessed or minimally processed foods) food. The overall purpose of ultra-processing is to create branded, convenient (durable, ready to consume), attractive (hyper-palatable) and highly profitable (low-cost ingredients) food products designed to displace all other food groups.

COMP 2 Food composition targets/standards/restrictions have been established by the EU for the content of the nutrients of concern (trans fats, added sugars, salt, saturated fat) in meals sold from food service outlets, in particular for those food groups that are major contributors to population intakes of those nutrients of concern.

Definitions and scope

- Meals sold at food service outlets include foods sold at quick service restaurants, dine-in restaurants and take-away outlets, coffee, bakery and snack food outlets (both fixed

outlets and mobile food vendors). This also includes foods from catering operations and delivery meals.

- Includes legislated bans on nutrients of concern
- Includes mandatory or voluntary targets, standards (i.e. reduce by X%, maximum mg/g per 100g or per serving)
- Excludes legislated restrictions related to other ingredients (e.g. additives)
- Excludes mandatory out-of-home meal composition regulations related to micronutrients, e.g. vitamins, minerals (e.g. folic acid or iodine fortification)
- Excludes food consumption standards/targets for fibre, healthy ingredients like fruits and vegetables
- Excludes general guidelines advising food service outlets to reduce nutrients of concern

Excludes the provision of resources or expertise to support food service outlets with reformulation

Policy Evidence Summary

Food composition targets/standards/restrictions for the content of nutrients of concern in industrially processed foods (COMP 1) and in meals sold from food service outlets (COMP 2)

The EU has not made a distinction in their policy documents between nutrients of concern in industrially processed foods and in meals sold from food service outlets. Therefore the same policy documents apply for both indicators. Some policy documents propose certain food categories for establishing food composition targets, including restaurant meals, catering meals, school food offer and ready meals.

1. *Mandatory food composition targets/standard/restrictions*

Policy documents which contain mandatory food composition restrictions at EU level are the Directive for the prohibition of added sugars in fruit juices and the Regulation on trans fats.

1.1 Directive for the prohibition of added sugars in fruit juices[6]

Since 2012, added sugars in fruit juices is no longer allowed under the Directive 2012/12/EU of the European Parliament and the Council.

1.2 Regulation on trans fats[7]

In April 2019, the Commission adopted an EU-wide legal limit for industrially produced trans fat (amending Annex III to regulation (EC) No 1925/2006). The adopted Regulation (No 2019/649 of 24 April 2019 as regards trans fat*)* prescribes a maximum limit of trans fat, other than trans fat naturally occurring in fat of animal origin, in food which is intended for the final consumer and food intended for supply to retail, of 2 grams per 100 grams of fat.

2. *Voluntary food composition policies*

The EU Framework for National Salt Initiatives and the Framework for National Initiatives on selected nutrients, with the Annexes on Saturated Fat and Added Sugars set voluntary targets/goals to establish a benchmark for overall reduction of the nutrients of concern. Participation by Member States in these frameworks is voluntary.

[6] Directive 2012/12/EU of the European Parliament and the Council of 19 April 2012 amending Council Directive 2001/112/EC relating to fruit juices and certain similar products intended for human consumption. Official Journal of the European Union L 115, 27.4.2012, p. 1–11. EUR-Lex: https://eur-lex.europa.eu/legal-content/EN/TXT/PDF/?uri=CELEX:32012L0012&from=EN

[7] Commission Regulation (EU) 2019/649 of 24 April 2019 amending Annex III to Regulation (EC) No 1925/2006 of the European Parliament and of the Council as regards trans fat, other than trans fat naturally occurring in fat of animal origin (Text with EEA relevance.). OJ L 110, 25.4.2019, p. 17–20. EUR-Lex: https://eur-lex.europa.eu/legal-content/EN/TXT/PDF/?uri=CELEX:32019R0649&from=EN

2.1 EU Framework for National Salt Initiatives[8]
In 2008, the High Level Group on Nutrition and Physical Activity developed the EU Framework for National Salt initiatives. The framework sets the goal to establish a benchmark for overall salt reduction of a minimum of 16% in 4 years against the individual baseline level in 2008, applicable to all food products as well as to food consumed in restaurants and catering facilities such as canteens (exceeding the 16% target is encouraged).
In order to effectively reduce salt intake, the framework proposes 12 food categories to concentrate activities on and member states are encouraged select at least 5 categories in their national plans. At least in four food categories (bread, meat products, cheeses and ready meals) the lowest possible salt levels ('best in class' levels) are identified at EU level but member states may also identify 'best in class' products within further food categories themselves.

The Commission published a report on the Implementation of the EU Salt Reduction Framework in 2012. [9] Concluded was that during the first two years of the implementation of the framework, 29 European countries (EU Member States, Norway and Switzerland) have salt reduction initiatives in place, but that the reduction of salt intake in populations a slow process is (e.g., concerning technological barriers, food safety concerns, consumer acceptance).

2.2 EU Framework for National Initiatives on selected nutrients[10]
At the beginning of 2011 the High Level Group on Nutrition and Physical Activity agreed on the EU Framework for National Initiatives on selected nutrients (by selected nutrients the framework refers to a complex set of target variables that may vary nationally, such as saturated fat, trans fat, energy, total fat content, added sugars, portion sizes and consumption frequency. Member States initiatives may cover one or a combination of these elements.). This sets out a framework for cooperation between EU Member States who want to work on reformulation issues, including set goals to establish a benchmark for overall reductions of the nutrients of concern.

2.3 Annex on Saturated Fat[11]
Complementing this Framework, an Annex on Saturated Fat was published in 2012. This annex proposes to set a general benchmark for saturated fat reduction of a minimum of 5% in 4 years and a minimum of an additional 5% reduction by 2020 against the individual baseline levels at the end of 2012. The annex suggests that priority is given to food categories that commonly represent major sources of saturated fat in European diets. In order to maximise the impact of reformulation, food business operators would prioritise the products with the largest market share. Furthermore, the annex emphasizes that care should be taken that reductions are delivered across the full range of food products (premium to economy) so that all population groups can benefit.

For dairy and meat products, the annex proposes special arrangements, like a fat content for dairy products at or below 1,5% and the provision that lower fat options of meat should at least not be more salted that higher-fat options. The annex further indicated that for ready meals, fats, oils and margarines, food items served in modern restaurants, breakfast cereals and meat products 'best in

[8] European Commission, High Level Group on Nutrition and Physical Activity, 2008. EU FRAMEWORK FOR NATIONAL SALT INITIATIVES. https://ec.europa.eu/health/archive/ph_determinants/life_style/nutrition/documents/salt_initiative.pdf
[9] European Commission, 2012. Implementation of the EU Salt Reduction Framework. Results of Member States Survey. https://ec.europa.eu/health/sites/health/files/nutrition_physical_activity/docs/salt_report_en.pdf
[10] European Commission, High Level Group on Nutrition and Physical Activity, 2011. EU FRAMEWORK FOR NATIONAL INITIATIVES ON SELECTED NUTRIENTS. https://ec.europa.eu/health/sites/health/files/nutrition_physical_activity/docs/euframework_national_nutrients_en.pdf
[11] European Commission, 2012. ANNEX I: SATURATED FAT. EU FRAMEWORK FOR NATIONAL INITIATIVES ON SELECTED NUTRIENTS. Ref. Ares(2012)699700 - 12/06/2012. https://ec.europa.eu/health/sites/health/files/nutrition_physical_activity/docs/satured_fat_eufnisn_en.pdf

class' saturated-fat levels would be identified at EU level, but member states may also identify 'best in class' products within other food categories.

2.4 Annex on Added Sugars[12]
Following the EU framework for national initiatives on selected nutrients, the High Level Group on Nutrition and Physical Activity agreed in December 2015 to an Added Sugars Annex. This Annex promoted a voluntary reduction of 10% in added sugars in processed foods by 2020, against the Member State baseline levels at the end of 2015 or to move towards 'best in class' levels. In general, the Annex mentions that the reduction of added sugars should not lead to an increase of the absolute amount or caloric content, saturated fat, trans fat or salt, but should lead to a decreased energy content. The annex prioritises food categories like Sugars Sweetened Beverages, sugars sweetened dairy, breakfast cereals, bread, confectionary, bakery products, ready meals, savoury snacks, sauces, sugars sweetened desserts, canned fruit and vegetables, school food offer and catering meals.

2.5 Other developments on Product Improvement
In 2016, a Roadmap for Action on Food Product Improvement was endorsed by the EU Member States and EFTA countries, food business operators and NGO's. They endorsed the urgency to develop more concerted action towards a healthier product offer, by lowering levels of salt, saturated fat and added sugars. [13]

A pilot database on the nutritional characteristics of food products in the EU was commissioned in 2017 to help monitor whether food products have increasingly less (or increasingly more) salt, fat or sugars.[14] It will inform authorities, consumers and industry about the scope for improvements in food products. Since «what gets measured gets done», this can strengthen national reformulation initiatives and support consumer choice, innovation and a level playing field for industry.

Also, a joint initiative of all the Member States and the Commission (a Joint Action) will adapt and implement practices that have already proven to work in the three areas: reformulation, marketing and public procurement.[15] Starting in 2020, it will promote the monitoring of food reformulation (namely the monitoring of reformulation initiatives) but also, the reduction of aggressive marketing to children of foods high in fat, salt and sugar, and the improvement of public procurement of food.

Comments/notes

[12] European Commission, High Level Group on Nutrition and Physical Activity, 2015. Annex II, Added Sugars. EU FRAMEWORK FOR NATIONAL INITIATIVES ON SELECTED NUTRIENTS. https://ec.europa.eu/health/sites/health/files/nutrition_physical_activity/docs/added_sugars_en.pdf
[13] Dutch Presidency EU Conference Food Product Improvement, 22 February 2016. Roadmap for Action on Food Product Improvement. http://www.agripress.be/_STUDIOEMMA_UPLOADS/downloads/roadmap.pdf
[14] European Commission, 2019. Initiatives on Nutrition and Physical acitivity. https://ec.europe.eu/health/sites/health/files/nutrition_physical_activity/docs/2019_initiatives_npa_en.pdf
[15]European Commission, 2019. Initiatives on Nutrition and Physical acitivity. https://ec.europe.eu/health/sites/health/files/nutrition_physical_activity/docs/2019_initiatives_npa_en.pdf

DOMAIN 2 - FOOD LABELLING - This domain concerns the extent to which the EU proposed/developed a regulatory system for consumer-oriented labelling on food packaging and menu boards in restaurants to enable consumers to easily make informed food choices and to prevent misleading claims.

LABEL 1 Ingredient lists and nutrient declarations in line with Codex recommendations are present on the labels of all packaged foods.

Definitions and scope

- Includes packaged foods manufactured within the EU countries or manufactured elsewhere and imported to the EU countries for sale.
- Nutrient declaration means a standardized statement or listing of the nutrient content of a food
- Excludes health and nutrition claims (see 'LABEL 2')
- Includes trans fats and added sugar which are not part of the standard seven elements generally part of mandatory nutrient declarations (energy, total fat, saturated fat, trans fat, carbohydrates, sugar, protein, sodium)

LABEL 2 Evidence-based regulations are in place for approving and/or reviewing claims on foods, so that consumers are protected against unsubstantiated and misleading nutrition and health claims.

- Nutrition claims include references to the nutritional content on food (e.g. low in fat).
- Health claims are claims that state, suggest or imply that a relationship exists between a food category, a food or one of its constituents and health. These include function claims, such as 'calcium strengthens bones' and disease risk reduction claims, such as 'A healthy diet rich in a variety of vegetables and fruit may help reduce the risk of some types of cancer'.
- Includes the use of a nutrient profiling system to classify food products into permitted/not permitted to carry health claims and/or nutrition claims
- 'Evidence-based' refers to regulations that are based on an extensive review of up-to-date research and expert input or a validated nutrient profiling model to inform decision-making about nutrition or health claims

LABEL 3 One or more interpretive, evidence-informed front-of-pack supplementary nutrition information system(s) proposed/required by the EU, which readily allow consumers to assess a product's healthiness, is/are applied to all packaged foods (examples are the Nutriscore and traffic lights).

Definitions and scope

- Nutrition information systems include traffic light labelling (overall or for specific nutrients); Warning labels; Nutriscore; star or points rating; percent daily intake.
- Keyhole and Finish heart symbol are not considered FOP labelling systems (but rather claims).
- 'Evidence-informed' refers to systems that utilise robust criteria (based on an extensive review of up-to-date research and expert input) or a validated nutrient profiling model to inform decision-making about the product's healthiness

LABEL 4 A simple and clearly-visible system of labelling the menu boards of all quick service restaurants (i.e. fast food chains) is set/proposed by the EU to be implemented by the Member States, which allows consumers to interpret the nutrient quality and energy content of foods and meals on sale.

Definitions and scope

- Quick service restaurants: In the EU' context this definition includes fast food chains as well as gas stations, kiosks, coffee, bakery and snack food chains. It may also include supermarkets where ready-to-eat foods are sold.
- Definition Euromonitor: Fast food outlets offer limited menus that are prepared quickly. Customers order, pay and pick up their order from a counter. Outlets tend to specialize in one or two main entrees such as hamburgers, pizza, ice cream, or chicken, but they usually also provide salads, drinks, dessert etc. Food preparation is generally simple and involves one or two steps, allowing for kitchen staffs generally consisting of younger, unskilled workers. Other key characteristics include: • A standardised and restricted menu; • Food for immediate consumption; • Tight individual portion control on all ingredients and on the finished product; • Individual packaging of each item; • Counter service; • A seating area, or close access to a shared seating area, such as in a shopping centre food court • For chained fast food, chained and franchised operations which operate under a uniform fascia and corporate identity. • Take out is generally present, as is drive-through in some markets.
- Labelling systems: Includes any point-of-sale (POS) nutrition information such as total kilojoules; percent daily intake; traffic light labelling; star rating, or specific amounts of nutrients of concern, salt warning labels.

Includes endorsement schemes (e.g., accredited healthy choice symbol) on approved menu items

Policy evidence summary

Ingredient lists and nutrient declarations in line with Codex recommendations (LABEL 1)

Mandatory policy instruments

The Regulation (EU) No 1169/2011 on the provision of food information to consumers[16]

The Regulation (EU) No 1169/2011 on the provision of food information to consumers entered into application on 13 December 2014. The obligation to provide nutrition information applied from 13 December 2016. [17] Article 6 of the Regulation prescribes as a basic requirement that 'any food intended for supply to the final consumer or to mass caterers shall be accompanied by food information in accordance with this Regulation'.

Chapter IV, section I, article 9 of this Regulation contains mandatory food information regarding the content and presentation of food. It contains a list of mandatory particulars including rules for a list of ingredients and a nutrition declaration. This is in line with Codex recommendations. [18]

---

[16] Regulation (EU) No 1169/2011 of the European Parliament and of the Council of 25 October 2011 on the provision of food information to consumers, amending Regulations (EC) No 1924/2006 and (EC) No 1925/2006 of the European Parliament and of the Council, and repealing Commission Directive 87/250/EEC, Council Directive 90/496/EEC, Commission Directive 1999/10/EC, Directive 2000/13/EC of the European Parliament and of the Council, Commission Directives 2002/67/EC and 2008/5/EC and Commission Regulation (EC) No 608/2004. OJ L 304, 22.11.2011, p. 18–63. EUR-Lex : https://eur-lex.europa.eu/legal-content/EN/TXT/PDF/?uri=CELEX:32011R1169&from=EN.

[17] https://ec.europa.eu/food/safety/labelling_nutrition/labelling_legislation_en

[18] Codex Alimentarius, 2018. GENERAL STANDARD FOR THE LABELLING OF PREPACKAGED FOODS CXS 1-1985. Adopted in 1985. Amended in 1991, 1999, 2001, 2003, 2005, 2008 and 2010. Revised in 2018. http://www.fao.org/fao-who-codexalimentarius/sh-

The list of ingredients shall include all the ingredients of the food, in descending order of weight. Some foods are exempted from bearing a list of ingredients, like fresh fruits and vegetables, carbonated water, foods consisting of a single ingredient, etc.

With regard to the nutrient declaration, declaration of the energy value and the amounts of fat, saturates, carbohydrate, sugars, protein and salt is mandatory, which is in line with Codex recommendations.[19] (This may voluntary be supplemented with other declarations, e.g. mono-unsaturated fats, fiber, vitamins). The Regulation No 1169/2011 does not allow for declaration of added sugars or trans fat in the nutrition declaration.

Nutrition declarations for foods listed in Annex V of the Regulation are not mandatory, for example unprocessed products that comprise a single ingredient or category of ingredients.
The Regulation contains an annex (XIV) with conversion factors to be used for calculating the energy value, which are in line with Codex recommendations.[20] The energy value and the amounts of nutrients shall be expressed per 100 g or per 100 ml, using the measurement units (kilojoule, kcal, grams, milligrams or micrograms) listed in an annex (XV) of the Regulation.

Commission Notice on the application of the principle of quantitative ingredients declaration (QUID) [21]
On 20 November 2017, the Commission adopted a Commission Notice on the application of the principle of quantitative ingredients declaration (QUID). T**he purpose of this Notice is to provide guidelines on the application of the principle of quantitative ingredients declaration in the context of Regulation** (EU) No 1169/2011.

Article 22(1) of the Regulation provides that 'The indication of the quantity of an ingredient or category of ingredients used in the manufacture or preparation of a food shall be required where the ingredient or category of ingredients concerned:

(a) Appears in the name of the food or is usually associated with that name by the consumer;
(b) Is emphasised on the labelling in words, pictures or graphics; or
(c) Is essential to characterise a food and to distinguish it from products with which it might be confused because of its name or appearance.'

With regard to article 22 point a of this Regulation, QUID is mandatory where the **ingredient** ('ham and mushroom pizza', 'strawberry yoghurt', 'salmon mousse', 'chocolate ice cream') or the **food category of the ingredients** ('vegetable pasty', 'fish fingers', 'nut loaf', 'fruit pie') appears in the name of the food. In these cases QUID should refer to the total vegetable, fish, nut or fruit content of the food.

When compound ingredients (for example cream filling, containing eggs) appear in the name of the food the QUID of these ingredients should also be given. There are again some exemptions to

proxy/en/?lnk=1&url=https%253A%252F%252Fworkspace.fao.org%252Fsites%252Fcodex%252FStandards%252FCXS%2B1-1985%252FCXS_001e.pdf

[19] Codex Alimentarius, 2017. GUIDELINES ON NUTRITION LABELLING CAC/GL 2-1985. Adopted in 1985. Revised in 1993 and 2011. Amended in 2003, 2006, 2009, 2010, 2012, 2013, 2015, 2016 and 2017. ANNEX adopted in 2011. Revised in 2013, 2015, 2016 and 2017. http://www.fao.org/fao-who-codexalimentarius/sh-proxy/en/?lnk=1&url=https%253A%252F%252Fworkspace.fao.org%252Fsites%252Fcodex%252FStandards%252FCXG%2B2-1985%252FCXG_002e.pdf

[20] Idem.

[21] European Commission, 2017. Commission Notice on the application of the principle of quantitative ingredients declaration (QUID). OJ C 393, 21.11.2017, p. 5–12. EUR-Lex : https://eur-lex.europa.eu/legal-content/EN/TXT/PDF/?uri=CELEX:52017XC1121(01)&from=EN.

these rules, for example for foods consisting of a single ingredient, or naturally occurring constituents in foods.

Evidence-based regulations for approving and/or reviewing claims on foods (LABEL 2)

Mandatory policy instruments

Regulation (EC) No 1924/2006 on Nutrition and Health Claims[22]
European Union rules on nutrition and health claims have been established by Regulation (EC) No 1924/2006. The definition provided for claims in the Regulation is:
"any message or representation which is not mandatory under Community or national legislation…". It includes foods placed on the market or supplied in bulk and foods intended for supply to restaurants, hospitals, schools, canteens and similar mass caterers.

The definitions provided for nutrition and health claims in the Regulation are:

"nutrition claim' means any claim which states, suggests or implies that a food has particular beneficial nutritional properties due to (a) the energy (calorific value) it provides (at a reduced or increased rate) or does not provide; (b) the nutrients or other substances it contains (in reduced or increased proportions or does not contain".

"health claim' means any claim that states, suggests or implies that a relationship exists between a food category, a food or one of its constituents and health".

General principles for all claims included in the regulation are that nutrition and health claims shall not:

- Be ambiguous, false or misleading;
- Give rise to doubt about the safety and/or the nutritional adequacy of other foods;
- Encourage or condone excess consumption of a food;
- State, suggest or imply that a balanced and varied diet cannot provide appropriate quantities of nutrients in general;
- Refer to changes in bodily functions, which could give rise to, or exploit fear in the consumer.

Article 4 of the Regulation contains the conditions for the use of nutrition and health claims, including the use of nutrient profiles, which are thresholds of nutrients such as fat, salt and sugars above which nutrition and health claims are restricted. Although the Regulation (Article 4) prescribes that the Commission by 19 January 2009 shall establish specific nutrient profiles, the Roadmap for the Evaluation of the Regulation published in 2015 indicates that the Commission did not establish nutrient profiles yet, due to the complexity of the subsequent discussions in relation to scientific issues and potential economic impacts.

Article 5 of the Regulation consists of general conditions which much be fulfilled to use nutrition and health claims. An example is that the presence, absence or reduced content of a nutrient in respect of which a claim is made has been shown to have a beneficial nutritional or physiological effect as established by generally accepted scientific data.

[22] REGULATION (EC) No 1924/2006 OF THE EUROPEAN PARLIAMENT AND OF THE COUNCIL of 20 December 2006 on nutrition and health claims made on foods. OJC 404, 30.12.2006, p. 9-25. EUR-Lex: https://eur-lex.europa.eu/LexUriServ/LexUriServ.do?uri=OJ:L:2006:404:0009:0025:EN:PDF

The Regulation further contains rules for authorization procedures for nutrition and health claims. Nutrition claims shall only be permitted if they are listed in the Annex of Regulation (EU) No 1047/2012[23] and are in conformity with the conditions set out in the Regulation (EC) No 1924/2006. Unlike nutrition claims, an application for authorization shall be submitted for health claims, in which the European Food and Safety Authority (EFSA) gives an opinion. After receiving the opinion of EFSA, the Commission shall submit to the Standing Committee on the Food Chain and Animal Health a draft decision on the list of permitted health claims. A public EU register of Nutrition and Health Claims lists all permitted nutrition claims and all authorized and non-authorized health claims.

The Directive on Fruit Juices (2012/12/EU)[24] includes specific rules for claims on fruit nectar products. According to the Directive, sugars and/or honey up to 20% of the total weight of the finished fruit nectar products and/or sweeteners are allowed. A claim stating that sugars have not been added to fruit nectar, and any claim likely to have the same meaning for the consumer, may only be made where the product does not contain any added mono- or disaccharides or any other food used for its sweetening properties.

Front-of-pack supplementary nutrition information system(s) (LABEL 3)
Mandatory EU policy instruments

Under the current EU rules, the indication of nutrition information on the front-of-pack is voluntary under certain conditions:

Voluntary EU policies

The Regulation EU 1169/2011[25] allows Member States to recommend or food business operators to use, on a voluntary basis, additional forms of expression and presentation of the nutrition declaration (on the front-of-pack) or other voluntary nutrition information provided that specific requirements are met. The EU does not allow Member States to implement mandatory front-of-pack labels.
Member States shall provide the Commission with the details of such additional forms of expression and presentation. These additional forms of expressions and presentation are usually provided on the front of the pack. The Regulation specifies that such expression/presentation of nutrition declarations or information has to be presented in the 'principle field of vision' (commonly known as the 'front of pack' as mentioned in recital 41 of the Regulation).

Article 35 of the Regulation (EU) required the Commission to submit by December 2017 a report to the European Parliament and Council on the use of front-of-pack nutrition labelling schemes, on their effect on the internal market and on the advisability of further harmonisation in the area. Considering the limited experience with front-of-pack labelling schemes in the EU, the adoption of the report was postponed with a view to including the experience with recently introduced schemes.

The Working Group of the Standing Committee on Plants, Animals, Food and Feed – Regulation (EU) No 1169/2011 on the provision of food information to consumers and the Advisory Group on the Food Chain, Animal and Plant Health held meetings in 2018 on the front-of-pack labelling. JRC

[23] COMMISSION REGULATION (EU) No 1047/2012 of 8 November 2012 amending Regulation (EC) No 1924/2006 with regard to the list of nutrition claims. EUR-Lex: https://eur-lex.europa.eu/LexUriServ/LexUriServ.do?uri=OJ:L:2012:310:0036:0037:EN:PDF
[24] Directive 2012/12/EU of the European Parliament and the Council of 19 April 2012 amending Council Directive 2001/112/EC relating to fruit juices and certain similar products intended for human consumption. Official Journal of the European Union L 115, 27.4.2012, p. 1–11. EUR-Lex: https://eur-lex.europa.eu/legal-content/EN/TXT/PDF/?uri=CELEX:32012L0012&from=EN
[25] https://eur-lex.europa.eu/legal-content/EN/TXT/PDF/?uri=CELEX:32011R1169&from=EN

conducted a study on FOP labelling schemes, which will be published together with the Commission report. At the moment of writing this evidence document (November 2019), the report has not yet been adopted and therefore, it is not yet available.

There are currently six front-of-pack schemes developed or endorsed by the public sector: the Keyhole logo (used in Sweden, Denmark, Lithuania), the Nutri-Score (used in France and Belgium and future implementation announced by Spain and Germany), the Multiple Traffic Light combined with Reference Intakes (UK), the Finnish Heart Symbol, the Slovenian 'Little Heart' logo and the Croatian 'Healthy Living' logo. The Mid Term Evaluation of the Action Plan on Childhood Obesity 2014-2020[26] showed that front of pack labelling was seen as one of the most difficult activities to work on, due to difficulties with placing foods in certain categories with respect to their nutritional value and resistance from the industry.

Labelling system of the menu boards of quick service restaurants (LABEL 4)
There is no system at EU level which prescribes the labelling of menu boards at quick service restaurants, which allows consumers to interpret the nutrient quality and energy content of foods and meals on sale. As regulated via Regulation (EU) No 1169/2011[27] only the allergen information is mandatory for non-prepacked food in restaurants and cafes (article 44). Information of other particulars is voluntary unless Member States adopt national measures. In the EU Action Plan on Childhood Obesity 2014-2020[28] is ‘implementing a clear signposting scheme for foods and meals in supermarkets and restaurants’ included as a voluntary objective for Member States. However, the Mid Term Evaluation of the Action Plan on Childhood Obesity 2014-2020[29] showed that menu labelling was seen as one of the most difficult activities to work on, due to difficulties with placing foods in certain categories with respect to their nutritional value and resistance from the industry.

Comments/notes

[26] Directorate-General for Health and Food Safety (European Commission) , European Public Health Alliance (EPHA) , National Institute for Public Health and the Environment (RIVM) , Netherlands Institute for Health Services Research (NIVEL), 2018. Supporting the mid-term evaluation of the EU action plan on childhood obesity. Luxembourg: Publications Office of the European Union, 2019 https://publications.europa.eu/en/publication-detail/-/publication/7e0320dc-ee18-11e8-b690-01aa75ed71a1/language-en

[27] Regulation (EU) No 1169/2011 of the European Parliament and of the Council of 25 October 2011 on the provision of food information to consumers, amending Regulations (EC) No 1924/2006 and (EC) No 1925/2006 of the European Parliament and of the Council, and repealing Commission Directive 87/250/EEC, Council Directive 90/496/EEC, Commission Directive 1999/10/EC, Directive 2000/13/EC of the European Parliament and of the Council, Commission Directives 2002/67/EC and 2008/5/EC and Commission Regulation (EC) No 608/2004. OJ L 304, 22.11.2011, p. 18–63. EUR-Lex : https://eur-lex.europa.eu/legal-content/EN/TXT/PDF/?uri=CELEX:32011R1169&from=EN

[28] European Commission, 2014. EU Action Plan on Childhood Obesity 2014-2020. https://ec.europa.eu/health/sites/health/files/nutrition_physical_activity/docs/childhoodobesity_actionplan_2014_2020_en.pdf

[29] Directorate-General for Health and Food Safety (European Commission) , European Public Health Alliance (EPHA) , National Institute for Public Health and the Environment (RIVM) , Netherlands Institute for Health Services Research (NIVEL), 2018. Supporting the mid-term evaluation of the EU action plan on childhood obesity. Luxembourg: Publications Office of the European Union, 2019 https://publications.europa.eu/en/publication-detail/-/publication/7e0320dc-ee18-11e8-b690-01aa75ed71a1/language-en

DOMAIN 3 - FOOD PROMOTION - This domain concerns the extent to which the EU has set/proposed policies to reduce the impact (exposure and power) of promotion of unhealthy foods to children including adolescents across all media.

- **Exposure of food marketing concerns the reach and frequency of a marketing message. This is dependent upon the media or channels, which are used to market foods.**
- **The power of food marketing concerns the creative content of the marketing message. For example, using cartoons or celebrities enhances the power (or persuasiveness) of a marketing message because such strategies are attractive to children.**

PROMO1 Effective policies are set/proposed by the EU to be implemented by the Member States to restrict exposure and power of promotion of unhealthy foods to children including adolescents through broadcast media (TV, radio).
**Definitions and scope**<br>➢ Includes mandatory policy (i.e. legislation or regulations) or voluntary standards, codes, guidelines set by the EU or by industry where the EU plays a role in development, monitoring, enforcement or resolving complaints (i.e. co-regulation).<br>➢ Includes free-to-air and subscription television and radio only (see PROMO2, PROMO3 and PROMO5 for other forms of media).<br>➢ Effective means that the policies are likely to reduce overall exposure of children, including adolescents to unhealthy food advertising over the day.

PROMO2 Effective policies are set/proposed by the EU to be implemented by the Member States to restrict exposure and power of promotion of unhealthy foods to children including adolescents through online and social media.
**Definitions and scope**<br>• Includes online media (e.g. social media, branded education websites, online games, competitions and apps)<br>• Where the promotion is specifically through other non-broadcast media than online and social media, this should be captured in 'PROMO3 and PROMO5'.<br>• Where the promotion is specifically in a children's setting, this should be captured in 'PROMO4'.<br>• Effective means that the policies are likely to reduce overall exposure of children, including adolescents to unhealthy food advertising over the day.

PROMO3 Effective policies are set/proposed by the EU to be implemented by the Member States to restrict exposure and power of promotion of unhealthy foods to children including adolescents through non-broadcast media other than packaging and online/social media.
**Definitions and scope**<br>• Non-broadcast media promotion includes: print (e.g. children's magazines), on/around public transport (e.g. signage, posters and billboards), cinema advertising, product placement and brand integration (e.g. in television shows and movies), direct marketing (e.g. provision of show bags, samples or flyers), or point-of-sale (POS) displays

- Non-broadcast media is excluding the media covered through other indicators like online and social media (PROMO2) and packaging (PROMO5)
- Where the promotion is specifically in a children's setting, this should be captured in 'PROMO4'
- Effective means that the policies are likely to reduce overall exposure of children, including adolescents to unhealthy food advertising over the day.

PROMO4 Effective policies are set/proposed by the EU to be implemented by the Member States to ensure that unhealthy foods are not commercially promoted to children including adolescents in settings where children gather (e.g. preschools, schools, sport and cultural events).

Definitions and scope

- Children's settings include: areas in and around schools, preschools/ kindergartens, daycare centres, children's health services (including primary care, maternal and child health or tertiary settings), sport, recreation and play areas/ venues/ facilities and cultural/community events where children are commonly present.
- Includes restrictions on marketing in government-owned or managed facilities/venues (including within the service contracts where management is outsourced)
- Includes restriction on unhealthy food sponsorship in sport (e.g. junior sport, sporting events, venues)
- Effective means that the policies are likely to reduce overall exposure of children, including adolescents to unhealthy food advertising over the day.

PROMO5 Effective policies are set/proposed by the EU to be implemented by the Member States to ensure that unhealthy foods are not commercially promoted to children (including adolescents) on food packages.

Definitions and scope

- Includes product design and packaging (e.g. use of celebrities or cartoons, competitions and give-aways)
- Where the promotion is specifically in a children's setting, this should be captured in 'PROMO4'
- Effective means that the policies are likely to reduce overall exposure of children (including adolescents) to unhealthy food advertising over the day.

Policy Evidence Summary

Policies to restrict exposure and power of unhealthy foods to children through broadcast, online and social media, non-broad cast media, in settings where children gather and on packages. (PROMO 1 -5)

In the EU, there are no strict regulations that prohibit Member States to market unhealthy foods to children through broadcast, online and social media, non-broadcast media, in settings where children gather and on packages. However the EU recognizes the influence of marketing and advertising to children and encourages Member States to take action.

EU Action Plan on Childhood Obesity 2014-2020[30]

The EU Action Plan on Childhood Obesity 2014-2020 developed by the EU Member States recognizes that efforts to restrict marketing and advertising to children and young people should include not only TV, but all marketing elements, including in store environments, promotional actions, internet presence and social media.

In the EU Action Plan on Childhood Obesity 2014-2020 several objectives are related to restrict the exposure and power of promotion of unhealthy foods to children through different kinds of media and settings. However non-broad cast media is not mentioned specifically.
These objectives fall under Action area 4 of the Plan: 'Restrict Marketing and advertising to children.' Main priority of this action area is 'to limit the exposure of children to advertisement of food/drinks high in fats, sugars and salt.
The objectives are:

- Defining nutrition criteria to use in a framework for marketing of foods to children. Target: consolidated nutrition criteria for restricting marketing of less healthy food options to children by 2016 at latest. Responsible: Member States and Stakeholders.
- Setting recommendations for marketing foods via TV, internet, sport events etc. Target: 30% of Member States with recommendations. Responsible: Member States.
- Encouraging media service providers to set up stricter codes of conduct on audiovisual communications to children regarding foods which are less healthy options. Actions to strengthen the implementation of Article 9.2 of the Directive on Audiovisual Media Services. Target: 80% of Member States with fully implemented Directive on Audiovisual Media Services. Responsible: Commission and Member States.
- Ensure that schools are free from marketing of less healthy food and drink options. Target: less than 5% of schools reporting violation, annually per Member State. Responsible: Member States and Stakeholders.

There are no objectives specifically related to the restriction of marketing to children on food packages. However the EU recognizes in the Plan the possible impact of marketing on food packages to children.

Supporting the mid-term Evaluation of the Action Plan on Childhood Obesity 2014-2020

The mid-term evaluation of the Action Plan[31] showed that almost 90% of the countries have initiatives to restrict marketing and advertising of foods and beverages that are high in salt, sugar or fat or that otherwise do not fit national or international nutritional guidelines to children or have plans in this area (6% of the countries). Two thirds of the initiatives being (voluntary) codes issued by the private sector. About half of the countries use nutrient criteria to restrict marketing of foods to children or have plans for it.

Directive 2018/1808 on Audiovisual Media Services[32]

The EU's Audiovisual Media Services Directive governs EU-wide coordination of national legislation on all audiovisual media, both traditional TV broadcasts and on-demand services.[33] The provisions included in the Directive to restrict the exposure and power of unhealthy food marketing to

[30] European Commission, 2014. EU Action Plan on Childhood Obesity 2014-2020. https://ec.europa.eu/health/sites/health/files/nutrition_physical_activity/docs/childhoodobesity_actionplan_2014_2020_en.pdf

[31] Directorate-General for Health and Food Safety (European Commission) , European Public Health Alliance (EPHA) , National Institute for Public Health and the Environment (RIVM) , Netherlands Institute for Health Services Research (NIVEL), 2018. Supporting the mid-term evaluation of the EU action plan on childhood obesity. Luxembourg: Publications Office of the European Union, 2019 https://publications.europa.eu/en/publication-detail/-/publication/7e0320dc-ee18-11e8-b690-01aa75ed71a1/language-en

[32] DIRECTIVE (EU) 2018/1808 OF THE EUROPEAN PARLIAMENT AND OF THE COUNCIL of 14 November 2018 amending Directive 2010/13/EU on the coordination of certain provisions laid down by law, regulation or administrative action in Member States concerning the provision of audiovisual media services (Audiovisual Media Services Directive) in view of changing market realities. EUR-Lex: https://eur-lex.europa.eu/legal-content/EN/TXT/PDF/?uri=CELEX:32018L1808&from=HR

[33] https://ec.europa.eu/digital-single-market/en/policies/audiovisual-media-services

children are not mandatory, but they encourage the Member States to ensure that self- and coregulation, including through codes of conduct, is used to effectively reduce the exposure of children to audiovisual commercial communications regarding foods and beverages that are high in salt, sugars, fat or that otherwise do not fit national or international nutritional guidelines (Article 9). This leaves a lot of space for Member States and media service providers to do or not do anything with the restriction of unhealthy foods marketing to children. However, giving only encouragements to Member States on the commercial communications, the Directive prohibits product placement in children's programmes.

The Directive doesn't give any definition of the notion of a child.[34] Under the EU Pledge, signatory companies have committed (1) not to advertise food on mass media where children under 12 make up 35% or more of the audience, except for products that meet common EU Pledge Nutrition Criteria (e.g. on sodium, saturated fat and salt), or (2) not to advertise their products at all to children under the age of 12 years.[35] WHO therefore recommends to extend the scope of the rules to protect all children. The Action Plan on Childhood Obesity 2014-2020 has already done this, by setting the goal to contribute to halting the rise in overweight and obesity in children and young people from 0 to 18 years by 2020.

Furthermore the Directive on Audiovisual Media Services is designed to limit unhealthy food marketing that is 'on children's programmes'. According to the WHO this means that existing regulations are not very successful as children watch mixed audience programmes as well.

Studies on the impact of marketing on children's behavior and the exposure of minors to TV and online marketing of unhealthy foods
March 2016, A study has been published about a EU-funded research project on the impact of marketing through social media, online games and mobile applications on children's behaviour'.[36] The study recommended to make marketing and advertisements more transparent to consumers and enhancing protection of children, Introduce protective measures targeting children directly and to update the Regulatory framework. In 2017, DG SANTE and DG CNECT launched a study on the exposure of minors to TV and online marketing of foods high in fat, salt or sugar.[37] Results will be available in May 2020. December 2019, the Joint Research Centre released a toolkit to support Member States in reducing the exposure of children and adolescents (up to 18 years old) to marketing of unhealthy food, non-alcoholic and alcoholic beverages.[38] The toolkit supports Member States in developing and updating codes of conducts in this area.
The 2017 study and toolkit include tools for the Member States to use the full potential of the Audio Visual Media Services Directive.

Joint Action
Also, a joint initiative of all the Member States and the Commission (a Joint Action) will adapt and implement practices that have already proven to work in the areas: reformulation, marketing and public procurement. Starting in 2020, it will promote the monitoring of food reformulation (namely the monitoring of reformulation initiatives) but also, the reduction of aggressive

[34] WHO Regional Office for Europe, 2018. Evaluating implementation of the WHO Set of Recommendations on the marketing of foods and non-alcoholic beverages to children. Progress, challenges and guidance for next steps in the WHO European Region. http://www.euro.who.int/__data/assets/pdf_file/0003/384015/food-marketing-kids-eng.pdf
[35] https://eu-pledge.eu/our-commitment/
[36] Consumers, Health, Agriculture and Food Executive Agency (Chafea) on behalf of Directorate-General for Justice and Consumer (European Commission), 2016. Study on the impact of marketing through social media, online games and and mobile applications on children's behaviour. Final report: https://ec.europa.eu/info/sites/info/files/online_marketing_children_final_report_en.pdf
[37] European Commission, 2019. Initiatives on Nutrition and Physical activity. https://ec.europa.eu/health/sites/health/files/nutrition_physical_activity/docs/2019_initiatives_npa_en.pdf and https://etendering.ted.europa.eu/cft/cft-display.html?cftId=2733
[38] https://ec.europa.eu/jrc/en/news/toolkit-limit-marketing-food-non-alcoholic-and-alcoholic-beverages-minors

marketing to children of foods high in fat, salt and sugar, and the improvement of public procurement of food.[39]

Strategy for a Better Internet for Children[40]
In 2012, the Commission published a European Strategy for a Better Internet for Children. One of the pillars in this Strategy is 'creating a safe environment for children online'. However, the Strategy does not say anything about restricting (unhealthy food) marketing to children.

Comments/notes

The EU and EU countries must respect, protect and promote children's rights. All EU policies that have an impact on children must be designed in line with the best interests of the child.[41]

[39] European Commission, 2019. Initiatives on Nutrition and Physical activity. https://ec.europa.eu/health/sites/health/files/nutrition_physical_activity/docs/2019_initiatives_npa_en.pdf
[40] European Commission, 2012. COMMUNICATION FROM THE COMMISSION TO THE EUROPEAN PARLIAMENT, THE COUNCIL, THE EUROPEAN ECONOMIC AND SOCIAL COMMITTEE AND THE COMMITTEE OF THE REGIONS. European Strategy for a Better Internet for Children. EUR-Lex: https://eur-lex.europa.eu/legal-content/EN/TXT/PDF/?uri=CELEX:52012DC0196&from=EN
[41] https://ec.europa.eu/info/policies/justice-and-fundamental-rights/rights-child_en

DOMAIN 4 - FOOD PRICES – This domain concerns the extent to which food pricing policies (e.g., taxes and subsidies) are aligned with health outcomes by helping to make the healthy eating choices the easier, cheaper choices

**PRICES1 Taxes or levies on healthy foods are minimised to encourage healthy food choices (e.g. low or no sales tax, excise, value-added or import duties on fruit and vegetables).**
**Definitions and scope**<br>• Includes exemptions from excise tax, ad valorem tax or import duty.<br>• Includes differential application of excise tax, ad valorem tax or import duty.<br>• Excludes subsidies (see 'PRICES3') or food purchasing welfare support (see 'PRICES4')

**PRICES2 Taxes or levies on unhealthy foods (e.g. sugar-sweetened beverages, foods high in nutrients of concern) are in place and increase the retail prices of these foods by at least 10% to discourage unhealthy food choices, and these taxes are reinvested to improve population health**
**Definitions and scope**<br>• Includes differential application of excise tax, ad valorem tax or import duty on high calorie foods or foods that are high in nutrients of concern

**PRICES 3 The intent of existing subsidies on foods, including infrastructure funding support (e.g. research and development, supporting markets or transport systems), is to favour healthy rather than unhealthy foods.**
**Definitions and scope**<br>• Includes agricultural input subsidies, such as free or subsidised costs for water, fertiliser, seeds, electricity or transport (e.g., freight) where those subsidies specifically target healthy foods.<br>• Includes programs that ensure that farmers receive a certain price for their produce to encourage increased food production or business viability.<br>• Includes grants or funding support for food producers (i.e. farmers, food manufacturers) to encourage innovation via research and development where that funding scheme specifically targets healthy food.<br>• Includes funding support for wholesale market systems that support the supply of healthy foods.<br>• Includes population level food subsidies at the consumer end (e.g. subsidising staples such as rice or bread).<br>• Excludes incentives for the establishment of, or ongoing support for, retail outlets (including greengrocers, farmers' markets, food co-ops, etc. See 'RETAIL2').<br>• Excludes subsidised training, courses or other forms of education for food producers - Excludes the redistribution of excess or second grade produce.<br>• Excludes food subsidies related to welfare support (see 'PRICES4')<br>• Should be in line with population nutrition goals related to the prevention of obesity and diet-related NCDs (e.g., reducing intake of nutrients of concern, and should not related to micronutrient deficiencies).

PRICES 4 The EU ensures that food-related income support programs are for healthy foods within EU countries.

Definitions and scope

- Includes programs such as ‘food stamps’ or other schemes where individuals can utilise government-administered subsidies, vouchers, tokens or discounts in retail settings for specific food purchasing.
- Excludes general programs that seek to address food insecurity such as government support for, or partnerships with, organisations that provide free or subsidised meals (including school breakfast programs) or food parcels or redistribute second grade produce for this purpose.
- Excludes food subsidies at the consumer end (e.g. subsidising staples at a population level – see ‘PRICES3’).

Policy evidence summary

Taxes or levies on healthy and unhealthy foods (PRICES 1 and PRICES 2)

At EU level there are no specific rules to minimize taxes or levies for encouraging healthy food choices or increase taxes to discourage unhealthy food choices. The Council Directive (2006/112/EC of 28 November 2006)[42] on the common system of value added tax has laid down rules that Member States shall apply a minimum of 5% VAT on all foods. This includes fruit and vegetables but also unhealthy foods. However, largely for historical reasons and under certain conditions, some countries (like the UK and Ireland) have been allowed to continue a zero VAT rate on certain products, like fruit and vegetables.[43]

Subsidies to favour healthy foods (PRICES 3)

An EU subsidy scheme to promote healthy foods is the School Fruit and Vegetable Scheme- a scheme to provide free fruit and vegetables to children in schools. This Scheme is part of the Market Measures of the Common Agricultural Policy of the EU and has been included in the Regulation on Common Market Organisation.[44] The Scheme began in the 2009/2010 school year and had an initial budget of 90 million euros with cofinancing by national or private funds required in each country.[45] Next to the Fruit and Vegetable Scheme a School Milk Scheme has been set up, which promotes the consumption of milk as an alternative to sugar-sweetened beverages. Both Schemes are part of the objectives in action area 2 ‘promote healthier environments, especially at schools and pre-schools’ of the EU Action Plan on Childhood Obesity.[46] The School Fruit and Vegetable Scheme and School Milk Scheme have been merged into a single School Food Scheme since the school year 2017/2018.[47] A budget of €250 million has been allocated to the scheme for the 2017/18 school year, of which €100 million has been set aside for milk and €150 million for

[42] COUNCIL DIRECTIVE 2006/112/EC of 28 November 2006 on the common system of value added tax. OJ L347, pp. 1-118. EUR-Lex: https://eur-lex.europa.eu/legal-content/EN/TXT/PDF/?uri=CELEX:32006L0112&from=EN

[43] https://ec.europa.eu/taxation_customs/business/vat/eu-vat-rules-topic/vat-rates_en and https://en.wikipedia.org/wiki/European_Union_value_added_tax

[44] REGULATION (EU) No 1308/2013 OF THE EUROPEAN PARLIAMENT AND OF THE COUNCIL of 17 December 2013 establishing a common organisation of the markets in agricultural products and repealing Council Regulations (EEC) No 922/72, (EEC) No 234/79, (EC) No 1037/2001 and (EC) No 1234/2007. OJ L347, pp. 671-854. EUR-Lex: https://eur-lex.europa.eu/legal-content/EN/TXT/PDF/?uri=CELEX:32013R1308&from=EN

[45] WHO Regional Office for Europe, 2015. Report: using price policies to promote healthier diets. http://www.euro.who.int/__data/assets/pdf_file/0008/273662/Using-price-policies-to-promote-healthier-diets.pdf

[46] European Commission, 2014. EU Action Plan on Childhood Obesity 2014-2020. https://ec.europa.eu/health/sites/health/files/nutrition_physical_activity/docs/childhoodobesity_actionplan_2014_2020_en.pdf

[47] https://ec.europa.eu/info/news/eu-school-fruits-vegetables-and-milk-scheme-launched-coming-school-year_en

fruit and vegetables. The mid-term evaluation of the Action Plan on Childhood Obesity[48] mentioned that none of the respondents saw the provision of fruit and vegetables in schools as one of the most successful activities. Nonetheless, the Schemes reached over 20 million children in 28 countries across the EU during the school year 2017/2018. During that time, a total of 255,500 tonnes of fresh fruit and vegetables and 178 million litres of milk were distributed due to more than 182 million euros from the EU budget.[49]

Liberalization of the sugar market

On 30 September 2017, after nearly 50 years, the sugar quota system has been ended.[50] This decision was agreed between the European Parliament and Member States in the 2013 reform of the Common Agricultural Policy.[51] The end of the quota system and other measures such as the minimum purchase price per tonne of sugar beet, has removed limitations on how much EU producers can put on the market[52] and gives producers the possibility to adjust their production to real commercial opportunities.[53] However despite this liberalization, the sugar sector still can fall back on various measures from the Common Agricultural Policy to deal with unexpected disturbances on the market.

This liberalization has raised concerns among various stakeholders (e.g. the European Public Health Alliance and the Centre for Diet and Activity Research) for the negative impact on health.[54] They fear that sugar will become cheaper potentially increasing sugar contents in existing products and the diversity of products containing sugar. A study carried out by JRC would have supported the expected price drop. The sugar market observatory provides the EU sugar sector with more transparency by means of disseminating market data and short-term analysis in a timely manner.[55] However, no data are available on whether higher amounts of sugar were added to foods sold to EU consumers.

Food-related income support programs (PRICES 4)

In 2014, the Fund for European Aid to the most Deprived (FEAD) was adopted as the successor to the most deprived persons (MDP) programme.[56] This programme offers material assistance and social inclusion measures, in addition to food aid. 83% of the total funding of this programme is devoted to food support according to the European Court of Auditors.[57] Management of the programme moved from the DG for Agriculture and Rural Development to the DG of Employment and Social Affairs. The programme provides 3,8 billion euros of EU funding for the period 2014-

[48] Directorate-General for Health and Food Safety (European Commission) , European Public Health Alliance (EPHA) , National Institute for Public Health and the Environment (RIVM) , Netherlands Institute for Health Services Research (NIVEL), 2018. Supporting the mid-term evaluation of the EU action plan on childhood obesity. Luxembourg: Publications Office of the European Union, 2019 https://publications.europa.eu/en/publication-detail/-/publication/7e0320dc-ee18-11e8-b690-01aa75ed71a1/language-en

[49] https://ec.europa.eu/commission/news/eu-school-fruit-vegetables-and-milk-scheme-2019-mar-27_en

[50] http://europa.eu/rapid/press-release_IP-17-3487_en.htm

[51] Regulation (EU) No 1308/2013 of the European Parliament and of the Council of 17 December 2013 establishing a common organisation of the markets in agricultural products and repealing Council Regulations (EEC) No 922/72, (EEC) No 234/79, (EC) No 1037/2001 and (EC) No 1234/2007, OJ L 347, pp. 671-854. EUR-Lex: https://eur-lex.europa.eu/legal-content/EN/TXT/?uri=celex:32013R1308

[52] European Union, 2018. The sugar sector in the EU. http://www.europarl.europa.eu/RegData/etudes/ATAG/2018/620224/EPRS_ATA(2018)620224_EN.pdf

[53] http://europa.eu/rapid/press-release_IP-17-3487_en.htm

[54] European Public Health Alliance (EPHA), AISBL, 2016. A CAP for a Healthy Living. Mainstreaming Health into the Common Agricultural Policy. https://ec.europa.eu/agriculture/sites/agriculture/files/future-of-cap/foc-fb-ha_en.pdf
Centre for Diet and Activity Research, 2015. EU Common Agricultural Policy Sugar Reforms Implications for Public Health https://www.cedar.iph.cam.ac.uk/wp-content/uploads/2015/10/Evidence-Brief-9-CAP-and-Sugar-v.1.3-27.10.15.pdf.

[55] https://ec.europa.eu/info/food-farming-fisheries/farming/facts-and-figures/markets/overviews/market-observatories/sugar_en

[56] European Court of Auditors, 2019. FEAD-Fund for European Aid to the Most Deprived: Valuable support but its contribution to reducing poverty is not yet established. Special Report, No.5. https://www.eca.europa.eu/lists/ecadocuments/sr19_05/sr_fead_en.pdf

[57] Idem.

2020 and is implemented at national level through operational programmes, which are approved by the European Commission. [58] Although we can say FEAD is a food-related income support programme, healthy foods are not really a subject in the FEAD regulation. [59] It does mention however that 'where appropriate the choice of food products to be distributed shall be made having considered their contribution to the balanced diet of the most deprived persons.'

In May 2018, the Commission adopted a legislative proposal for a new European Social Fund Plus (ESF+) Programme, based on the Multiannual Financial Framework for the period 2012-2027, which merges the FEAD programme with other programmes (e.g. the Health Programme, Employment and Social Innovation programme, European Social Fund and Youth Employment Initiative programme). [60] Although this ESF+ Programme includes a strong health dimension with a budget of 413 million euros, the provision about food support has not been changed a lot compared to the FEAD provision. The same reference (Article 17) has been made that ''where appropriate the choice of food products to be distributed shall be made having considered their contribution to the balanced diet of the most deprived persons.' [61]

Comments/notes

DOMAIN 5 - FOOD PROVISON – This domain concerns the extent to which the EU ensures that there are healthy food service policies to be implemented by Member States in government-funded settings to ensure that food provision encourages healthy food choices, and the extent to which the EU actively encourages and supports private companies to implement similar

PROV1 The EU ensures that there are clear, consistent policies (including nutrition standards) to be implemented by Member States in schools and early childhood education services for food service activities (canteens, food at events, fundraising, promotions, vending machines etc.) to provide and promote healthy food choices.

Definitions and scope

- Includes early childhood education and care services (0-5 years).
- Schools include government and non-government primary and secondary schools (up to age 18 years)
- Includes policies and nutrition standards to provide and promote healthy food choices or to limit or restrict the provision or promotion of unhealthy food choices
- Includes policies that relate to school meals programs, where the program is partly or fully funded, managed or overseen by the government
- Excludes programmes in schools that are targeted to children of low socioeconomic groups only (as these would be covered under PRICES4)

[58] https://ec.europa.eu/social/main.jsp?catId=1089
[59] Regulation (EU) No 223/2014 of the European Parliament and of the Council of 11 March 2014 on the Fund for European Aid to the Most Deprived. OJ L 72, pp. 1-41. EUR-Lex: https://eur-lex.europa.eu/LexUriServ/LexUriServ.do?uri=OJ:L:2014:072:0001:0041:EN:PDF
[60] European Commission. https://ec.europa.eu/health/funding/future_health_budget_en
[61] European Commission, 2018. Proposal for a REGULATION OF THE EUROPEAN PARLIAMENT AND OF THE COUNCIL on the European Social Fund Plus (ESF+). https://ec.europa.eu/commission/sites/beta-political/files/budget-may2018-european-social-fund-plus-regulation_en.pdf

PROV2 The EU ensures that there are clear, consistent policies to be implemented by Member States in other public sector settings for food service activities (canteens, food at events, fundraising, promotions, vending machines, etc.) to provide and promote healthy food choices.

Definitions and scope

- Public sector settings include: - Government-funded or managed services where the government is responsible for the provision of food, including public hospitals and other in-patient health services (acute and sub-acute, including mental health services), residential care homes, aged and disability care settings, custodial care facilities, prisons and home/community care services - Government-owned, funded or managed services where the general public purchase foods including health services, parks, sporting and leisure facilities, community events etc. - Public sector workplaces
- Includes private businesses that are under contract by the government to provide food
- Excludes 'public settings' such as train stations, venues, facilities or events that are not funded or managed by the government (see 'RETAIL4')
- Excludes school and early childhood settings (see 'PROV1')
- Includes policies and nutrition standards to provide and promote healthy food choices or to limit or restrict the provision or promotion of unhealthy food choices
- Includes the strategic placement of foods and beverages in cabinets, fridges, on shelves or near the cashier
- Includes the use of signage to highlight healthy options or endorsements (such as traffic lights or a recognised healthy symbol)
- Includes modifying ingredients to make foods and drinks healthier, or changing the menu to offer healthier options
- Excludes public procurement standards (see 'PROV3')

PROV3 The EU ensures that there are clear, consistent public procurement standards to be implemented by Member States in public sector settings for food service activities to provide and promote healthy food choices.

Definitions and scope

- Includes standards for the public sector which encourage the procurement of healthy foods.
- Includes standards for the public sector which discourage the procurement of unhealthy foods.
- Includes public sector settings as defined in PROV 1 and PROV 2.

PROV4 The EU ensures that there are good support and training systems to be implemented by Member States to help schools and other public sector organisations and their caterers meet the healthy food service policies and guidelines

Definitions and scope

- Includes support for early childhood education services as defined in 'PROV1'
- Public sector organisations include settings defined in 'PROV2'
- Support and training systems include guidelines, toolkits, templates (e.g. policy/guidelines or contracts), recipes and menu planning tools, expert advice, menu and product

assessments, online training modules, cook/caterer/other food service staff information and training workshops or courses

PROV5 The EU actively encourages and supports private companies to provide and promote healthy foods and meals in their workplaces

Definitions and scope

- For the purpose of this indicator, 'private companies' includes for-profit companies and extends to non-government organisations (NGOs) including not-for-profit/charitable organisations, community-controlled organisations, etc.
- Includes healthy catering policies, fundraising, events - Includes support and training systems including guidelines, toolkits, templates (e.g. policy/guidelines or contracts), recipes and menu planning tools, expert advice, menu and product assessments, online training modules, cook/caterer/other food service staff information and training workshops or courses (where relevant to the provision of food in a workplace)
- Excludes the provision or promotion of food to people not employed by that organisation (e.g. visitors or customers)
- Excludes support for organisations to provide staff education on healthy foods

Policy evidence summary

Policies to be implemented by Member States in schools and early childhood education services (PROV 1) and other public sector settings to provide and promote healthy food choices (PROV 2)

EU Action Plan on Childhood Obesity 2014-2020[62]
The EU Action Plan on Childhood Obesity 2014-2020, developed by Member States' delegates, proposes voluntary policies to be implemented by Member States to provide and promote healthy food choices in schools and pre-schools under Action area 2: 'promote healthier environments, especially at schools and pre-schools', Action area 3 'make the healthy option, the easier option' and Action area 4 'restrict marketing and advertising to children' .

An objective in Action area 2 is to provide the healthy option and increase daily consumption of fresh fruit and vegetables, healthy food and water in schools (with a targeted focus on schools in underprivileged districts). Focus should also be on making the school environment attractive to eat in. Actions named for this objective are the (extension of national implementation of the) EU School Fruit Scheme and the EU School Milk Scheme[63], the promotion of the intake of of tap water whilst reducing the intake of sweetened beverages, and the implementation of pilot projects on the distribution of healthy foods to vulnerable groups, including children, in the populations of EU NUTS2 regions in Romania, Bulgaria and Slovakia as well as in Poland and Hungary.
Action area 3 ('make the healthy option, the easier option'), also includes objectives and actions for schools. Examples are the development of a sign posting scheme promoting healthy options in schools and preschools, providing quality standards for the foods included in school meals and free supply of fresh drinking water. Action area 4 has the objective to ensure that schools are free from marketing of less healthy food and drink options.

[62] European Commission, 2014. EU Action Plan on Childhood Obesity 2014-2020. https://ec.europa.eu/health/sites/health/files/nutrition_physical_activity/docs/childhoodobesity_actionplan_2014_2020_en.pdf
[63] These Schemes have now been merged into one EU school fruit, vegetables and milk Scheme.

The High Level Group on Nutrition and Physical Activity wrote an opinion in 2017 in which they called the Member States' national health authorities to take another step in creating a healthier school environment for children by convincing agriculture authorities to only fund the distribution of products with no added sugars under the School Fruit, Vegetables and Milk Scheme. [64]

The Action Plan on Childhood Obesity 2014-2020 proposes voluntary policies to be implemented by Member States to provide and promote healthy food choices in other public sector settings under Action area 1: 'support a healthy start in life' and Action area 3 'make the healthy option, the easier option'. The Action Plan mentions as objectives/activities in these areas: create a healthy environment in hospitals and primary health care facilities, continue to encourage all food producers to enhance their reformulation actions especially those providing food and drinks in sport halls and venues and community activity centres, and promote free water in public areas like administrations and hospitals.

Supporting the Mid term evaluation of the EU Action Plan on Childhood Obesity[65]
From the Mid term evaluation of the EU Action Plan on Childhood Obesity, can be concluded that Action area 2 '*Promote healthier environments, especially in schools' is* one of the areas for action that is addressed by most countries. According to the evaluation, policies to improve the school environment are in place or planned in all countries, whereas policies on supplying easily accessible free drinking water in schools are available in 64% of the countries. In another 21% of the countries tap water is safe, so free drinking water is considered to be available in schools also. Most school food policies include policies on vending machines and energy drinks. All but three EU Member States participated in the EU School Fruit and Vegetable Scheme in the 2015/2016 school year. All EU Member States participate in the new School Fruit, Vegetable and Milk Scheme, that applies since the 2017/2018 school year.

JRC Toolkits to promote healthy food and drink choices in school-aged children[66]
In 2016, JRC published two toolkits to promote healthy food and drink choices in school-aged children. The first toolkit is about 'how to promote fruit and vegetable consumption in schools' [67]and the second about 'how to promote water intake in schools'.[68]

Public Procurement standards to be implemented by Member States in public sector settings to provide and promote healthy food choices (PROV 3)
Regarding to the procurement of food services, every year, around €82 billion is spent in the EU on the purchase of food services.[69] The Commission acknowledges that public procurement on food represents an opportunity to help steer both demand and supply of healthier food options. [70]

---

[64]https://ec.europa.eu/health/sites/health/files/nutrition_physical_activity/docs/hlg_opinion_addedsugars_school_fruitvegetablesmmilkscheme_en.pdf
[65] Directorate-General for Health and Food Safety (European Commission) , European Public Health Alliance (EPHA) , National Institute for Public Health and the Environment (RIVM) , Netherlands Institute for Health Services Research (NIVEL), 2018. Supporting the mid-term evaluation of the EU action plan on childhood obesity. Luxembourg: Publications Office of the European Union, 2019. https://publications.europa.eu/en/publication-detail/-/publication/7e0320dc-ee18-11e8-b690-01aa75ed71a1/language-en
[66] https://ec.europa.eu/jrc/en/science-update/promoting-healthy-food-and-drink-choices-school-age-children
[67] Tsz Ning Mak, Stefan Storcksdieck genannt Bonsmann, Sandra Caldeira and Jan Wollgast; How to promote fruit and vegetable consumption in schools: a toolkit; EUR 27946 EN; doi:10.2788/678338

[68] JRC, Stefan Storcksdieck genannt Bonsmann, Tsz Ning Mak , Sandra Caldeira, Jan Wollgast, 2016. How to promote water intake in schools: a toolkit. EUR 27945 EN ; DOI : 10.2788 /95048
[69] JRC. Maltese EU Presidency team. DG SANTE. Public Procurement of Food for Health. Technical report on the school setting. 2017.
[70] European Commission, 2019. Initiatives on Nutrition and Physical Activity. https://ec.europa.eu/health/sites/health/files/nutrition_physical_activity/docs/2019_initiatives_npa_en.pdf

The EU rules for public procurement have been laid down in Directive 2014/24/EU.[71] This Directive aims to introduce a minimum body of the public procurement rules for the award of public contracts into national law. There are no specific provisions in this Directive that relate to the provision and promotion of healthy food choices. However, the Directive states that "nothing in the Directive should prevent the imposition or enforcement of measures necessary to protect public policy, public morality, public security, health etc."

Next to this Directive, JRC has written a technical report (2017) on public procurement of food for health in school settings to help schools draft better food catering contracts. [72] Mentioned in this report are the guide 'buying social'[73] which is a very concrete tool to help public authorities to buy goods and services in a socially responsible way in line with EU rules and the EU Handbook 'buying green'[74], which is including food and catering services and focuses mostly on sustainable and organic foods. Regarding fruits and vegetables the handbook recommends 'specify minimum percentages and/or award points for the use of fruit and vegetables that are in season'.

A joint initiative of all the Member States and the Commission (a Joint Action) will adapt and implement practices that have already proven to work in the three areas: reformulation, marketing and public procurement. Starting in 2020, it will promote the monitoring of food reformulation, the reduction of aggressive marketing to children of foods high in fat, salt and sugar, and the improvement of public procurement of food.[75]

Support and training systems to be implemented by Member States to help schools and other public sector organisations and their caterers meet the healthy food service policies and guidelines (PROV 4)

JRC Toolkits to promote healthy food and drink choices in school-aged children[76]
As we have seen above, JRC published two toolkits in 2016 to promote healthy food and drink choices in school-aged children. The fruit and vegetable consumption toolkit provides policymakers with successful measures to promote fruit and vegetable consumption in schools and to support in implementing and evaluating these measures in schools. The water intake toolkit aims to provide policymakers with successful measures to promote water consumption and reduce SSBs in schools and to support in implementing and evaluating these measures in schools.

EU Action Plan on Childhood Obesity 2014-2020[77]
In the EU Action Plan on Childhood Obesity 2014-2020 there are objectives including training, supporting of schools, health care staff, catering/restaurant staff with dedicated responsibility for these objectives and activities to Member States and Stakeholders (but not to the European Commission). Examples are cooperation between teachers, catering staff school managers and health care providers to create a healthy school environment, providing nutritional training to school kitchen staff and restaurant staff, providing quality standards for the foods in school meals, providing guidelines on portion sizes and providing nutrition guidelines for health experts working

[71] DIRECTIVE 2014/24/EU OF THE EUROPEAN PARLIAMENT AND OF THE COUNCIL of 26 February 2014 on public procurement and repealing Directive 2004/18/EC. EUR-Lex:https://eur-lex.europa.eu/legal-content/EN/TXT/PDF/?uri=CELEX:32014L0024&from=en
[72] JRC, 2017. Maltese EU Presidency team. DG SANTE. Public Procurement of Food for Health. Technical report on the school setting. https://ec.europa.eu/jrc/sites/jrcsh/files/public-procurement-food-health-technical-report.pdf
[73] http://europa.eu/rapid/press-release_IP-11-105_en.htm
[74] European Union, 2016. Buying green! A handbook on green public procurement 3rd Edition. https://ec.europa.eu/environment/gpp/pdf/Buying-Green-Handbook-3rd-Edition.pdf
[75] European Commission, 2019. Initiatives on Nutrition and Physical Activity. https://ec.europa.eu/health/sites/health/files/nutrition_physical_activity/docs/2019_initiatives_npa_en.pdf
[76] https://ec.europa.eu/jrc/en/science-update/promoting-healthy-food-and-drink-choices-school-age-children
[77] European Commission, 2014. EU Action Plan on Childhood Obesity 2014-2020. https://ec.europa.eu/health/sites/health/files/nutrition_physical_activity/docs/childhoodobesity_actionplan_2014_2020_en.pdf

with socially disadvantages communities and children. However, the EU did not develop guidelines and/or trainings themselves.

Encouraging and supporting private companies to provide and promote healthy foods and meals in their workplaces (PROV 5)

European FOOD Programme[78]
The European Fighting Obesity through Offer and Demand (FOOD) Programme is very much focusing on improving the nutritional quality of the food offered in restaurants and workplaces. Two main objectives of the programme are: (1) educating employees to help them improve their nutritional choices and (2) improving the nutritional quality of nutrition on offer by liasing with food providers. FOOD developed a communication campaign (guides, leaflets, posters, video's, training sessions, websites etc.) on nutrition that reached out to 352,000 restaurants and four million employees in Belgium, the Czech Republic, France, Italy, Spain and Sweden. FOOD succeeded from a co-funded project in 2009 to a selfsustained programme. It took on board four additional countries: Austria, Romania, the Slovak Republic and Portugal.

Health and Safety at Work
The EU Strategic Framework on Health and Safety at Work 2014-2020[79] does not say anything about nutrition/diet in relation to health. However the Communication from the Commission on Safer and Healthier Work for All (2017)[80] does mention unhealthy diets as a threat to workers health. One key action identified is helping businesses to comply with occupational and health rules. As a result of this the Commission wrote the Guidance 'Health and Safety at work is everybody's business-practical guidance for employers'. [81] However nutrition/diet-related health is not mentioned in this guidance.

Comments/notes

DOMAIN 6 - FOOD IN RETAIL – This domain concerns the extent to which the EU has the power to set/propose policies and programs to be implemented by Member States to support the availability of healthy foods and limit the availability of unhealthy foods in communities (outlet density and locations) and in-store (product placement)

RETAIL1 Zoning laws and policies are proposed by the EU to be implemented by the Member States to place limits on the density or placement of quick serve restaurants or other outlets selling mainly unhealthy foods in communities and/or access to these outlets (e.g. opening hours).

Definitions and scope

[78] http://www.food-programme.eu/en/about/food-from-2009-to-today/article/fighting-obesity-through-offer-and-demand
[79] European Commission, 2014. COMMUNICATION FROM THE COMMISSION TO THE EUROPEAN PARLIAMENT, THE COUNCIL, THE EUROPEAN ECONOMIC AND SOCIAL COMMITTEE AND THE COMMITTEE OF THE REGIONS on an EU Strategic Framework on Health and Safety at Work 2014-2020. Brussels, 6.6.2014 COM(2014) 332 final. EUR-Lex: https://eur-lex.europa.eu/legal-content/EN/TXT/PDF/?uri=CELEX:52014DC0332&from=GA
[80] European Commission, 2017. COMMUNICATION FROM THE COMMISSION TO THE EUROPEAN PARLIAMENT, THE COUNCIL, THE EUROPEAN ECONOMIC AND SOCIAL COMMITTEE AND THE COMMITTEE OF THE REGIONS Safer and Healthier Work for All - Modernisation of the EU Occupational Safety and Health Legislation and Policy. Brussels, 10.1.2017 COM(2017) 12 final. EUR-Lex : https://eur-lex.europa.eu/legal-content/EN/TXT/?uri=COM:2017:012:FIN
[81] European Commission, 2016. 'Health and Safety at work is everybody's business'- practical guidance for employers. Luxembourg: Publications Office of the European Union. doi:10.2767/721956 https://publications.europa.eu/en/publication-detail/-/publication/cbe4dbb7-ffdc-11e6-8a35-01aa75ed71a1

- Includes the consideration of public health in EU programs and policies that guide national policies and the policies, priorities and objectives to be implemented at the local government level through their planning schemes
- Includes the consideration of public health in subordinate planning instruments and policies
- Includes an EU guideline that sets the policy objective of considering public health when reviewing and approving fast food planning applications.
- Includes limitations to access of unhealthy food outlets (i.e. opening hours)
- Excludes laws, policies or actions of national and local governments

RETAIL2 Zoning laws and policies are proposed by the EU to be implemented by the Member States to encourage the availability of outlets selling fresh fruit and vegetables and/or access to these outlets (e.g. opening hours, frequency i.e. for markets).

Definitions and scope

- Outlets include supermarkets, produce markets, farmers' markets, greengrocers, food cooperatives
- Includes fixed or mobile outlets
- Excludes community gardens, edible urban or backyard gardens (usually regulated by local governments)
- Includes policies that support local governments to reduce license or permit requirements or fees to encourage the establishment of such outlets
- Includes policies that guide streamlining and standardising planning approval processes or reducing regulatory burdens for these outlets
- Includes actions to improve access to healthy food outlets (i.e. opening hours; frequency i.e. for markets)
- Includes the provision of financial grants or subsidies to outlets

RETAIL3 The EU ensures existing support systems are in place to be implemented by the Member States to encourage food stores to promote the in-store availability of healthy foods and to limit the in-store availability of unhealthy foods.

Definitions and scope

- Food stores include supermarkets, convenience stores (including 'general stores' or 'milk bars'), greengrocers and other specialty food retail outlets
- Support systems include guidelines, resources or expert support
- Includes all settings with food retail stores, including but not exclusive to; train stations, venues, facilities or events frequented by the public etc.
- Excludes settings owned or managed by the government (see 'PROV2' and 'PROV4')
- Includes the strategic placement of foods and beverages in cabinets, fridges, on shelves or near the cashier - Includes the use of signage to highlight healthy options or endorsements (such as traffic lights or a recognised healthy symbol)
- Includes offering healthier food and drink products, or changing the menu or store layout to offer more healthy options
- Includes decreasing the offer of unhealthy food and drink products
- Excludes reformulation and labelling in relation to nutrients of concern (COMP1; LABEL4)

RETAIL4 The EU ensures existing support systems are in place to be implemented by the Member States to encourage the promotion and availability of healthy foods in food service outlets and to discourage the promotion and availability of unhealthy foods in food service outlets

Definitions and scope

- Food service outlets include for-profit quick service restaurants, eat-in or take-away restaurants, pubs, clubs
- Support systems include guidelines, resources or expert support
- Includes settings such as train stations, venues, facilities or events frequented by the public
- Excludes settings owned or managed by the government (see 'PROV2' and 'PROV4')
- Includes the strategic placement of foods and beverages in cabinets, fridges, on shelves or near the cashier - Includes the use of signage to highlight healthy options or endorsements (such as traffic lights or a recognised healthy symbol)
- Includes modifying ingredients to make foods and drinks more healthy, or changing the menu to offer more healthy options
- Excludes reformulation and labelling in relation to nutrients of concern (COMP2; LABEL4)

Policy evidence summary

Zoning laws and policies to place limits on the density or placement of quick service restaurants or other outlets selling mainly unhealthy foods (RETAIL 1) or to encourage the availability of outlets selling fruits and vegetables (RETAIL 2)

The EU does not have competence in the field of spatial planning, so nor on implementing zoning laws and policies to place limits on the density or placement of quick service restaurants selling mainly unhealthy foods or to encourage the availability of outlets selling fruits and vegetables.

However, the EU has some legal instruments on spatial planning that point out to human health, but all lack explicit assessment or declaration with respect to healthy food environments:

Environmental Impact Assessments

One example is the Strategic Environmental Assessment (SEA, Directive 2001/42/EC)[82] which requires an impact assessment to be conducted for land use and other spatial programmes prepared or adopted by national, regional or local authorities. The SEA assesses these plans according to the environmental effects including "human health" in general.

Another legal instrument of the EU is the Environmental Impact Assessment (EIA, Directive 2011/92/EU)[83]. The EIA requires and impact assessment for certain types of large scale projects, e.g long distance railway lines, motorways, airports). For urban development projects national authorities have to decide whether an EIA is needed. The assessment must include information on

[82] DIRECTIVE 2001/42/EC OF THE EUROPEAN PARLIAMENT AND OF THE COUNCIL of 27 June 2001 on the assessment of the effects of certain plans and programmes on the environment. OJ L 197, pp. 30-37. 27.1.2001. EUR-Lex: https://eur-lex.europa.eu/legal-content/EN/TXT/PDF/?uri=CELEX:32001L0042&from=EN

[83] DIRECTIVE 2011/92/EU OF THE EUROPEAN PARLIAMENT AND OF THE COUNCIL of 13 December 2011 on the assessment of the effects of certain public and private projects on the environment. OJ L26, pp.1-21, 28.1.2012. EUR-Lex : https://eur-lex.europa.eu/LexUriServ/LexUriServ.do?uri=OJ:L:2012:026:0001:0021:EN:PDF

all relevant environmental effects including human health, but not specifically includes food environmental indicators.

Agenda and discourse setting

If we look at the reports of the EU to set agenda and discourse around spatial planning, healthy food environments do not play a role. Some reports like the 'Urban Europe report- Statistics on Cities, Towns and Suburbs'[84] do address sustainable consumption and production, public health or healthy living environments (but neither specifically address the food environment).

The most recent EU policy documents within the environmental domain are based on the 7th Environment Action Programme (2014-2020)[85]. One objective of this programme is to safeguard the quality of life and well-being of its habitants. Although this objective refers to human health and well-being there is no reference made to healthy food environments, but mostly to improve implementation and legislation of air quality, noise regulation, drinking water and hazardous chemicals, as part of a broader, strategic approach for a non-toxic environment.

Spatial planning policies

Spatial planning policies like the Territorial Agenda of the European Union 2020[86] do not contain anything about health or healthy food environments. The objective of the Territorial Agenda 2020 is to provide strategic orientations for territorial development, fostering integration of territorial dimension within different policies at all governance levels and to ensure implementation of the Europe 2020 Strategy according to territorial cohesion principles.

The European Spatial Development Perspective mentions health and food a few times, but this relates to health of the population in general and food production rather than healthy food environments. [87]

European Environment Agency (EEA)

The EU has also an European Environment Agency (EEA) but although 'environment and health' is a theme of this Agency, it focuses on topics like chemicals, human biomonitoring and noise.

Support systems for food stores and food service outlets (RETAIL 3 and RETAIL 4)

There are no support systems at EU level to encourage food stores to promote the in-store availability of healthy foods and to limit the in-store availability of unhealthy foods. Neither are there support systems at EU level to encourage the promotion and availability of healthy foods in food service outlets and to discourage the promotion and availability of unhealthy foods in food service outlets.

Comments/notes

[84] Eurostat, 2016. Urban Europe : Statistics on cities, towns and suburbs. Luxembourg: Publications office of the European Union. doi: 10.2785/91120. https://ec.europa.eu/eurostat/documents/3217494/7596823/KS-01-16-691-EN-N.pdf

[85] DECISION No 1386/2013/EU OF THE EUROPEAN PARLIAMENT AND OF THE COUNCIL of 20 November 2013 on a General Union Environment Action Programme to 2020 'Living well, within the limits of our planet'. OJ L 354, pp. 171-200. 28.12.2013. EUR-Lex : https://eur-lex.europa.eu/legal-content/EN/TXT/PDF/?uri=CELEX:32013D1386&from=EN

[86] Territorial Agenda of the European Union 2020. Towards an Inclusive, Smart and Sustainable Europe of Diverse Regions; agreed at the Informal Ministerial Meeting of Ministers responsible for Spatial Planning and Territorial Development on 19th May 2011 Gödöllő, Hungary. https://www.nweurope.eu/media/1216/territorial_agenda_2020.pdf

[87] European Commission, 1999. ESDP European Spatial Development Perspective
Towards Balanced and Sustainable Development of the Territory of the European Union. Agreed at the Informal Council of Ministers responsible for Spatial Planning in Potsdam, May 1999.
https://ec.europa.eu/regional_policy/sources/docoffic/official/reports/pdf/sum_en.pdf

DOMAIN 7 - FOOD TRADE AND INVESTMENT – This domain concerns the extent to which the EU ensures that trade and investment agreements protect food sovereignty, favour healthy food environments, are linked with domestic health and agricultural policies in ways that are consistent with health objectives, and do not promote unhealthy food environments.
** The evidence description in this domain has not been checked yet by relevant EU governmental officials/experts*

TRADE1 The EU undertakes risk impact assessments before and during the negotiation of trade and investment agreements, to identify, evaluate and minimize the direct and indirect negative impacts of such agreements on population nutrition and health

Definitions and scope

- Includes policies or procedures that guide the undertaking of risk impact assessments before or during negotiation to assess risks and benefits in relation to public health and population nutrition
- Includes policies or procedures that guide the evaluation of trade and investment agreements after an agreement is finalised to monitor the impact for the purpose of informing future negotiations or reviews
- Includes policies or procedures that guide public consultation procedures before and during negotiations
- Any trade or economic agreements still considered active

TRADE2 The EU adopts measures to manage investment and protect their regulatory capacity with respect to public health nutrition

Definitions and scope

- Includes provisions in trade or economic agreements that protect the capacity of government to implement domestic policy in relation to food environments. This includes protections with respect to tariffs, non-tariff measures (such as quotas, regulations, standards, testing, certification, licensing procedures) and measures related to foreign direct investment
- Binding commitments made under Trade and Investment Agreements (TIA's) can constrain the way countries can regulate goods, services, and investments to promote public interests (including public health) in a way that is upstream from domestic policy processes.

Policy evidence summary

Risk impact assessments before and during the negotiation of trade and investment agreements to identify, evaluate, and minimize the direct and indirect negative impacts of such agreements on population nutrition and health (TRADE 1)

The European Commission conducts analysis on various aspects of EU trade policy in order to assess the impact of trade on the EU and the global economy. [88] The economic assessment of the negotiated outcome after the conclusion of the negotiation and before the signature of the agreement is mandatory and undertaken by the Chief Economist team in DG Trade and

[88] http://ec.europa.eu/trade/policy/policy-making/analysis/

independent consultants with funding from the Commission. Additionally, three other major types of assessments and evaluations can be conducted during the life of a proposed new trade agreement: an impact assessment at the initial design stage, a Sustainability Impact Assessment (SIA) during the negotiations, and finally, an ex post evaluation, after implementation. [89]

Regarding the impact assessments, the European Commission has published guidelines (Better Regulation guidelines- impact assessment) [90]. Possible identified impacts in these guidelines are the impacts on health. However, it is not further specified if this also refers to population nutrition and health. The European Commission wrote a handbook for trade sustainability impact assessment[91], which contains a detailed description of the environmental and social analysis included in the SIA. There are no clear references made to health and nutrition in this handbook. The handbook mentions only that the environmental analysis 'should try to identify how the agreement under negotiation could contribute to promote sustainable consumption and production' and for the social analysis that 'potential impacts on the health and safety of individuals or populations should also be considered' (health related employment).

As can be concluded, impacts on population nutrition and health are very marginal present in the assessment procedure of new trade agreements and health impact assessments are not mandatory for new trade agreements.

Measures to manage investment and protect regulatory capacity with respect to public health nutrition (TRADE 2)

The EU has adopted trade rules that protect the capacity of the EU to implement EU policy. However these rules are not specifically related to public health nutrition or healthy food environments. Nevertheless we will shortly describe the EU trade policy and the way it is related to or touches upon subjects as health and food environments.

The EU manages its trade and investment relation with non-EU countries through its trade and investment policy. [92] The Treaty on the Functioning of the European Union sets out rules on EU trade policy.[93] In Article 207 it mentions 'The Council shall act unanimously for the negotiation and conclusion of agreements: in the field of trade in social, education and health services, where these agreements risk seriously disturbing the national organization of such services and prejudicing the responsibility of Member States to deliver them'. Specific rules to protect its regulatory capacity with respect to agricultural policy have been included in Article 39 and 40 of the Treaty, aiming to stabilize imports and exports as means to address market volatility, and deliver on the objectives of the CAP. Sustainability is also an important aspect in EU trade policy. The EU's Trade for All Strategy[94] sets out the EU's priorities for trade policy, to make trade policy-making more effective, transparent, and ethical. In this Strategy health is mentioned several times like 'The Commission will continue promoting an ambitious global health agenda', 'EU health, safety, consumer protection, labour and environmental rules are amongst the most protective and effective in the world' and 'one of the aims of the EU is to ensure that economic growth goes hand in hand with social justice, respect for human rights, high labour and environmental standards,

[89] European Commission, 2016. Handbook for trade sustainability impact assessment. 2nd edition. Luxembourg: Publications Office of the European Union. doi:10.2781/999660. http://trade.ec.europa.eu/doclib/docs/2016/april/tradoc_154464.PDF

[90] European Commission. Chapter III, Guidelines on Impact Assessment. https://ec.europa.eu/info/sites/info/files/better-regulation-guidelines-impact-assessment.pdf

[91] European Commission, 2016. Handbook for trade sustainability impact assessment. 2nd edition. Luxembourg: Publications Office of the European Union. doi:10.2781/999660. http://trade.ec.europa.eu/doclib/docs/2016/april/tradoc_154464.PDF

[92] http://ec.europa.eu/trade/policy/policy-making/

[93] European Union, 2012. Consolidated version of the Treaty on the Functioning of the European Union. OJ C 326, pp. 47-390. 26. 10.2012. EUR-Lex: https://eur-lex.europa.eu/legal-content/EN/TXT/PDF/?uri=CELEX:12012E/TXT&from=EN

[94] European Union, 2015. Trade for All. Towards a more responsible trade and investment policy. Luxembourg: Publications Office of the European Union, 2014. DOI: 10.2781/472505. https://trade.ec.europa.eu/doclib/docs/2015/october/tradoc_153846.pdf

and health and safety protection.' The report on the implementation of the Trade for All Strategy published in 2017[95] does not mention anything relevant in relation to population nutrition and health.
Comments/notes

DOMAIN 8 - LEADERSHIP - This domain concerns the extent to which political leadership ensures that there is strong support for the vision, planning, communication, implementation and evaluation of policies and actions to create healthy food environments, improve population nutrition, and reduce diet-related inequalities.

LEAD1 **There is strong, visible, political support (at the head of European Commission/Parliament level) expressed at European, supra national as well as national level for improving food environments, population nutrition, diet related NCDs and their related inequalities"**
**Definitions and scope**<br>• Visible support includes statements of intent, election commitments, budget commitments, establishing priorities and targets, demonstration of support in the media, other actions that demonstrate support for new or strengthened policy<br>• Documents that contain evidence of strong political support include media releases, speeches, pre-election policy papers, introduction of a bill, strategic plans with targets or key performance indicators

LEAD2 **Clear population intake targets have been proposed by the EU for the nutrients of concern and / or relevant food groups to meet WHO and European recommended dietary intake levels**
**Definitions and scope**<br>• Includes targets which specify population intakes according to average reductions in percentage or volume (e.g. mg/g) for salt/sodium, saturated fat, trans fats or added or free sugars*. Typically requires the government to establish clear dietary guidelines on the maximum daily intake of nutrients of concern<br>• *Free sugar is defined as is sugar no longer in its naturally-occurring state (i.e., no longer in whole fruits, vegetables, unsweetened dairy, and grains) and can be consumed as is or incorporated into other foods. Examples include table sugar, syrup, honey, fruit juice and nectars. Added sugar is defined as the free sugar that has been added to foods, however regulatory definitions vary widely under different jurisdictions, some of which are currently under review. These differ from naturally occurring sugars, defined as the sugar found naturally within whole foods (i.e., within whole fruits, vegetables, dairy, and some grains).

[95] European Commission, 2017. REPORT FROM THE COMMISSION TO THE EUROPEAN PARLIAMENT, THE COUNCIL, THE EUROPEAN ECONOMIC AND SOCIAL COMMITTEE AND THE COMMITTEE OF THE REGIONS. Report on the Implementation of the Trade Policy Strategy Trade for All Delivering a Progressive Trade Policy to Harness Globalisation. Brussels, 13.9.2017. COM(2017) 491 final. https://trade.ec.europa.eu/doclib/docs/2017/september/tradoc_156037.pdf

LEAD3 Clear, interpretive, evidenced-informed food based dietary guidelines have been established and conveyed to EU countries

Definitions and scope

- Food-based dietary guidelines should be for both genders and key age groups including infants and pregnant women
- Evidence-informed includes extensive review of up-to-date research and mechanisms to seek expert input
- Evidence includes ways the FBDG have been used to develop/implement policies to improve diets

LEAD4 There is a comprehensive, transparent, up-to-date implementation plan linked to EU countries' needs and priorities, to improve food environments , reduce the intake of the nutrients of concern to meet WHO and European recommended dietary intake levels, and reduce diet-related NCDS.

Definitions and scope

- Includes documented plans with specific actions and interventions (i.e. policies, programs, partnerships)
- Plans should be current (i.e. maintain endorsement by the current government and/or are being reported against)
- Plans should refer to actions to improve food environments (as defined in the policy domains above) and should include both policy and program strategies
- Excludes overarching frameworks that provide general guidance and direction
- Includes priority policy and program strategies, social media marketing for public awareness and threat of legislation for voluntary approaches.

LEAD5 EU priorities have been established to reduce inequalities or protect vulnerable populations in relation to diet, nutrition, obesity and NCDs

Definitions and scope

- Frameworks, strategies or implementation plans specify aims, objectives or targets to reduce inequalities including taking a preventive approach that addresses the social and environmental determinants of health
- Frameworks, strategies or implementation plans identify vulnerable populations or priority groups
- Implementation plans specify policies or programs that aim to reduce inequalities for specific population groups
- Excludes priorities to reduce inequalities in secondary or tertiary prevention

Policy evidence summary

Strong, visible, political support (at the head of European Commission/Parliament level) for improving food environments, population nutrition, diet related NCDs and their related Inequalities (LEAD 1)

EU political support for improving food environments, population nutrition, diet related NCDs and their related inequalities, is mainly linked to voluntary incentive measures e.g. raising awareness to prevent chronic disease and promote good health and cooperation measures.

White Paper 'A Strategy on Nutrition, Overweight and Obesity-related health issues' (2007)[96]
The White Paper 'A Strategy on Nutrition, Overweight and Obesity-related health issues' (2007) expresses the political support of the European Commission for improving food environments and population nutrition, and their related inequalities. The purpose of this White Paper was to set out an integrated EU approach to contribute to reducing ill health due to poor nutrition, overweight and obesity. The European Commission states that it will aim to complement and optimize actions undertaken at national and regional level and recognizes its responsibility regarding actions directly relating to the functioning of the internal market (e.g. labelling requirements, health claims authorisations), specific frameworks (e.g. Common Agricultural Policy and the fruit and vegetables Scheme), regional policy (structural funds) and audiovisual and media policy. 'Better informed consumers', 'making the healthy option available', 'priority groups and settings', 'developing the evidence-base to support policy making' and 'developing monitoring systems' are subjects addressed in the White Paper with regard to the European Commission's actions.

In 2010, a resolution of the European Parliament on this White Paper was published.[97] The European Parliament calls for example: to recognize obesity as a chronic disease, for more tangible measures especially targeted at children and at risk groups, for improved labelling, to develop guidelines on nutrition policies at schools, to cut VAT on fruit and vegetables and to reconsider the voluntary approach in the Audio visual Media Services Directive. It further approves setting up the High Level Group on Nutrition and Physical Activity and European health survey systems, urges the Commission to take a more holistic approach to nutrition, and urges the European Union to take a leading role in formulating a common approach and the coordination between Member States.

Council Conclusions[98]
The Council Conclusions on nutrition and physical activity of 2014[99], on food product improvement of 2016, on Childhood Obesity of 2017 and on healthy nutrition for children of 2018 show further political support. These Conclusions recognize that healthy diets and physical activity significantly reduce the risk of non-communicable diseases and contribute significantly to the healthy grow of children, healthy life years, and quality of life of children, adolescents and adults. The council urges further to address inequalities in relation to nutrition and physical activity between and within Member States. The council conclusions on healthy nutrition for children of 2018[100] invite the Member States for example to actively fight an obesogenic environment, especially in settings where children gather, invites the Commission and the Member States to monitor the compliance of national and EU voluntary initiatives aimed at reducing the impact on children of marketing of food with a high content of fat, salt and sugars and invites the Commission to continue prioritising

---

[96] Commission of the European Communities, 2007. White Paper on Nutrition, Overweight and Obesity-related health issues. Brussels, 30.5.2007 COM(2007) 279 final. https://ec.europa.eu/health/archive/ph_determinants/life_style/nutrition/documents/nutrition_wp_en.pdf

[97] European Parliament, 2010. White Paper on Nutrition, Overweight and Obesity-related health issues. P6_TA(2008)0461. European Parliament resolution of 25 September 2008 on the White Paper on nutrition, overweight and obesity-related health issues (2007/2285(INI)). OJ C8 E, pp. 97-105. 14.1.2010. EUR-Lex : https://eur-lex.europa.eu/LexUriServ/LexUriServ.do?uri=OJ:C:2010:008E:0097:0105:EN:PDF

[98] https://ec.europa.eu/health/nutrition_physical_activity/key_documents_en#anchor3

[99] Council of the European Union, 2014. Council conclusions on nutrition and physical activity. EMPLOYMENT, SOCIAL POLICY, HEALTH and COSUMER AFFAIRS. Council meeting Luxembourg, 20 June 2014. Brussels, press office, consilium. https://www.consilium.europa.eu/media/28049/143285.pdf

[100] Council of the European Union, 2018. Healthy nutrition for children: The healthy future of Europe - Council conclusions (22 June 2018). Outcome of proceedings. 10355/18. http://data.consilium.europa.eu/doc/document/ST-10355-2018-INIT/en/pdf

public health, in particular by addressing issues of cross-border importance, such as marketing of food to children, food product improvement and labelling.

Strategic Plan DG Health and Food Safety 2016-2020[101]
The Strategic Plan of DG Health and Food Safety refers to the Treaty on the Functioning of the European Union, which shapes the EU's degree of influence in health and food policies, saying that Member States are responsible for the definition of their health policy and for the organization and delivery of health services and medical care. DG Health and Food Safety therefore states that EU action is mainly linked to incentive measures, e.g. raising awareness to prevent chronic disease and promote good health and cooperation measures. However, one of its missions is to 'improve and protect human health'.

Paper 'Initiatives on Nutrition and Physical Activity'[102]
The Paper 'Initiatives on Nutrition and Physical Activity' makes clear that the Commission offers practical tools to help Member States and EU citizens to make real progress in the areas of nutrition and physical activity.

European Commission's Reflection Paper towards a sustainable Europe by 2030 (2019)[103]
The Commission recognizes that overweight and obesity are the EU's central nutrition issues. Achieving safe and healthy diets and ensuring productive and sustainable agricultural systems, fisheries and aquaculture are a priority in the EU.

European action supporting the 2030 Agenda and the Sustainable Development Goals[104]
In her Commission Staff Working document on European Action supporting the 2030 Agenda and the Sustainable Development Goals, the European Commission gives an overview of key existing European initiatives related to the achievement of the 2030 Agenda for Sustainable Development. Related to SDG 2 'End hunger, achieve food security and improved nutrition, and promote sustainable agriculture' the Common Agricultural Policy is mentioned including the promotion of healthy diets among schoolchildren through the EU School Fruit and Vegetables and Milk Scheme. Under this SDG is also FOOD 2030 mentioned, which will explore at EU level what is needed to transform and future-proof our food systems to be sustainable, resilient, competitive, diverse, responsible and performant in their provision of accessible, healthy and sustainable food and diets for all. Related to SDG 3 'Ensure healthy lives and promote well-being for all at all ages' it is stressed that the EU supports, coordinates and complements the Member States in their health policy. In relation to chronic, non-communicable diseases and mental health and well-being the Commission co-funds joint actions with Member States on chronic diseases and ageing, mental health, cancer, dementia, physical activity, nutrition and obesity and concluded a joint action on alcohol under the Health Programme.

Tartu Call for a Healthy Lifestyle[105]

[101] European Commission. Strategic Plan 2016-2020, DG Health and Food Safety. Ref. Ares(2017)6260978 - 20/12/2017 https://ec.europa.eu/info/sites/info/files/strategic-plan-2016-2020-dg-sante_en_0.pdf
[102] European Commission. DG Health and Food Safety, 2019. Initiatives on Nutrition and Physical Activity'. https://ec.europa.eu/health/sites/health/files/nutrition_physical_activity/docs/2019_initiatives_npa_en.pdf
[103] European Commission, 2019. Reflection paper Towards a sustainable Europe by 2030. Brussels, COM(2019)22 of 30 January 2019. https://ec.europa.eu/commission/sites/beta-political/files/rp_sustainable_europe_30-01_en_web.pdf
[104] European Commission, 2016. Key European action supporting the 2030 Agenda and the Sustainable Development Goals. Commission Staff Working Document SWD(2016) 390 final. Strasbourg, 22.11.2016. https://ec.europa.eu/europeaid/sites/devco/files/swd-key-european-actions-2030-agenda-sdgs-390-20161122_en.pdf

[105] https://ec.europa.eu/commission/presscorner/detail/en/ip_19_3028

In the Tartu Call for a Healthy Lifestyle[106] launched in 2017, Tibor Navracsics, Commissioner for Education, Culture, Youth and Sport, Vytenis Andriukaitis, Commissioner for Health and Food Safety, and Phil Hogan, Commissioner for Agriculture and Rural Development, made 15 commitments to promote healthy lifestyles through sport, food, innovation or research.

Steering Group on Health Promotion, Disease Prevention and Management of non-communicable diseases
Members of the Steering Group had identified nutrition and physical activity as their main priority for implementation in 2019.[107]

Establishment of clear population intake targets (LEAD 2)

There are no clear population intake targets established at EU level. EFSA publishes intake recommendations in Scientific Opinions. JRC collates intake recommendations from authoritative public health bodies in the Health Promotion and Knowledge Gateway.

EFSA
EFSA's Panel on Nutrition, Dietetics and Allergies (NDA) advises on request of the European Commission on recommended intake value for macro and micro nutrients, by writing Scientific Opinions.[108] Since 2004, the Panel has published Opinions on Dietary Reference Values for macro and micronutrients (e.g. water, energy, carbohydrates and dietary fibre, fats, protein, molybdenum, fluoride, vitamin C, iron, selenium, calcium and manganese). The most recent Opinions are on the dietary reference values for sodium and chloride, published in September 2019.[109]

EFSA was asked to provide scientific advice on the daily intake of added sugars in 2017. A scientific protocol was developed. A draft is expected to be ready for public consultation in 2020.[110]

European Commission-JRC
The Joint Research Centre seeks to support Member States in setting nutrition policies based on authoritative recommendations. JRC therefore collates dietary recommendations of nutrient of concern and policy options/actions in the Health Promotion and Knowledge Gateway.[111] It refers to different sources for their recommendations, like the EFSA, the WHO, WCRF/AICR, Food Based Dietary Guidelines in EU Countries, and the FAO.

Clear, interpretative, evidence-informed food based dietary guidelines (LEAD 3)
The EU has not established food-based dietary guidelines for Member States. The Commission requested EFSA in 2010 to provide guidance on the translation of nutrient based dietary advice into food-based dietary guidelines.[112] In reply to this, the EFSA Panel on Nutrition, Dietetic Products, and Allergies (NDA) published a Scientific Opinion on establishing Food-based dietary guidelines.[113] This Opinion states that the differences in EU countries regarding dietary habits, the prevalence of nutrient imbalances and diet-related public health issues require that food-based dietary guidelines (FBDGs) are established by the country or region. All countries in the EU plus

[106] https://ec.europa.eu/sport/sites/sport/files/ewos-tartu-call_en.pdf
[107]https://ec.europa.eu/health/sites/health/files/major_chronic_diseases/docs/ev_20180411_flash_en.pdf
[108] https://www.efsa.europa.eu/en/panels/nda
[109] https://www.efsa.europa.eu/en/efsajournal/pub/5778 and https://www.efsa.europa.eu/en/efsajournal/pub/5779
[110] https://www.efsa.europa.eu/en/press/news/190719
[111] https://ec.europa.eu/jrc/en/health-knowledge-gateway/promotion-prevention/nutrition
[112] European Food Safety Authority, 2010. Scientific Opinion on establishing Food-Based Dietary Guidelines. EFSA Panel on Dietetic Products, Nutrition, and Allergies (NDA). Parma, Italy. EFSA Journal 2010; 8(3):1460, pp.1-42. https://efsa.onlinelibrary.wiley.com/doi/epdf/10.2903/j.efsa.2010.1460
[113] Idem.

Switzerland, Norway and Iceland have FBDGs, which are published on the website of the European Commission.[114]

Comprehensive, transparent, up-to-date implementation plan (LEAD 4)
At EU level, there is one implementation plan to improve food environments, reduce the intake of the nutrients of concern and reduce diet-related NCDs, which is the EU Action Plan on Childhood Obesity 2014-2020.[115] This plan contains eight areas for action, the following five of which directly relate to the food and nutrition environment:

1. Support a healthy start in life
2. Promote healthier environments, especially at schools and pre-schools
3. Make the healthy option, the easier option
4. Restrict marketing and advertising to children
5. Inform and empower families

Per area operational objectives and responsible parties (EC, Member States, stakeholders) are identified.

EU priorities to reduce inequalities or protect vulnerable populations in relation to diet, nutrition, obesity and NCDs (LEAD 5)

The EU recognizes that obesity has a higher prevalence among people in lower socio-economic groups, that these groups need particular attention and that reducing health inequalities is important to mitigate the vicious cycle of ill health and poverty, as appears from documents like the White Paper of the European Commission in 2007 on the Strategy for Europe on Nutrition, Overweight and Obesity related health issues[116], and the Strategic Plan 2016-2020 of DG Sante[117]The EU Action Plan on Childhood Obesity 2014-2020[118] also states that it is important to tackle health inequalities. Furthermore, the EU Cohesion Policy which is the EU's main investment policy and supports investments in health,[119] aims to achieve economic, social and territorial cohesion, by reducing economic and social inequalities between EU regions. It has a thematic objective dedicated to promoting social inclusion and tackle poverty and discrimination by for example investing in health and social infrastructure, reducing inequalities in terms of health status and promoting social inclusion through improved access to social, cultural and recreational services. [120]

Comments/notes

[114] https://ec.europa.eu/jrc/en/health-knowledge-gateway/promotion-prevention/nutrition/food-based-dietary-guidelines
[115] European Commission, 2014. EU Action Plan on Childhood Obesity 2014-2020. https://ec.europa.eu/health/sites/health/files/nutrition_physical_activity/docs/childhoodobesity_actionplan_2014_2020_en.pdf.
[116] Commission of the European Communities, 2007. White Paper on Nutrition, Overweight and Obesity-related health issues. Brussels, 30.5.2007 COM(2007) 279 final. https://ec.europa.eu/health/archive/ph_determinants/life_style/nutrition/documents/nutrition_wp_en.pdf
[117] European Commission. Strategic Plan 2016-2020 DG Health and Food Safety. Ref. Ares(2017)6260978 - 20/12/2017. https://ec.europa.eu/info/sites/info/files/strategic-plan-2016-2020-dg-sante_en_0.pdf
[118] European Commission, 2014. EU Action Plan on Childhood Obesity 2014-2020. https://ec.europa.eu/health/sites/health/files/nutrition_physical_activity/docs/childhoodobesity_actionplan_2014_2020_en.pdf.
[119] https://ec.europa.eu/health/funding/structural_funds_en and European Commission, 2016. COMMISSION STAFF WORKING DOCUMENT Key European action supporting the 2030 Agenda and the Sustainable Development Goals. Strasbourg, 22.11.2016 SWD(2016) 390 final. https://ec.europa.eu/europeaid/sites/devco/files/swd-key-european-actions-2030-agenda-sdgs-390-20161122_en.pdf

[120] European Commission, 2016. COMMISSION STAFF WORKING DOCUMENT Key European action supporting the 2030 Agenda and the Sustainable Development Goals. Strasbourg, 22.11.2016 SWD(2016) 390 final. https://ec.europa.eu/europeaid/sites/devco/files/swd-key-european-actions-2030-agenda-sdgs-390-20161122_en.pdf

DOMAIN 9 - GOVERNANCE: This domain concerns the extent to which the EU has structures in place to ensure transparency and accountability, and encourage broad community participation and inclusion when formulating and implementing policies and actions to create healthy food environments, improve population nutrition, and reduce diet-related inequalities.

GOVER1 There are procedures in place to restrict commercial influences on the development of policies related to food environments where they have conflicts of interest with improving population nutrition. for example: restricting lobbying influences.

Definitions and scope

- Includes government policies, guidelines, codes of conduct or other mechanisms to guide actions and decision-making by government employees, for example conflict of interest declaration procedures
- Includes procedures to manage partnerships with private companies or trade associations representing industries that are consulted for the purpose of developing policy, for examples committee procedural guidelines or terms of reference.
- Includes publicly available, up to-to-date registers of lobbyist and/ or their activities

GOVER2 Policies and procedures are implemented for using evidence in the development of food and nutrition policies

Definitions and scope

- Includes policies, procedures or guidelines to support government employees in the use of evidence for policy development including best practice evidence review methodology (including types and strength of evidence needed) and policy implementation in the absence of strong evidence (where the potential risk or harms of inaction are great).
- Includes policies, procedures or guidelines that stipulate the requirements for the establishment of a scientific or expert committee to inform policy development.
- Includes the use of evidence-based models, algorithms and tools to guide policy development or within policy to guide implementation (e.g. nutrient profiling model)
- Includes government resourcing of evidence and research by specific units, either within or across government departments

GOVER3 Policies and procedures are implemented for ensuring transparency in the development of food and nutrition policies

Definitions and scope

- Includes policies or procedures that guide the use of consultation in the development of food policy
- Includes policies or procedures to guide the online publishing of private sector and civil society submissions to government around the development of policy and subsequent government response to these.
- Includes policies or procedures to guide the online publishing of scoping papers, draft and final policies

- Include policies or procedures to guide public communications around all policies put forward but not progressed

GOVER4 The EU ensures public access to comprehensive nutrition information and key documents (e.g. budget documents, annual performance reviews and health indicators) for the public.

Definitions and scope

- Includes policies and procedures to guide the timely, online publishing of government budgets, performance reviews, audits, evaluation reports or the findings of other reviews or inquiries
- Includes 'freedom of information' legislation and related processes to enable the public access to government information on request, with minimal restrictions and exemptions
- Includes policies or procedures to guide the timely, online publishing of population health data captured / owned by government

Policy evidence summary

Procedures to restrict commercial influences on the development of policies related to food environments where they have conflict of interests with improving population nutrition (GOVER 1)

There are procedures in place to restrict commercial influences on the development of policies where they have conflict of interests. These procedures are not specifically related to policies related to the food environment, but more generally to 1) commissioners and 2) commission staff.

Commissioners

The Treaty on the Union[121] and the Treaty on the Functioning of the European Union[122] provide rules with regard to the independence of Commissioners and to make sure they behave with integrity and discretion. Commissioners also have to sign a Code of Conduct, which states that Members shall avoid any situation which may give rise to a conflict of interest or which may be perceived as such.[123] Any financial or other interest or assets which create a conflict of interest must be declared. These declarations must be made public. Gifts with a value of more than 150 euros shall not be accepted. The Commission's Protocol Department shall keep a public register of gifts. After ceasing to hold office, former Members shall be bound to their duty of integrity and discretion. The Commission further established an Independent Ethical Committee which advises the Commission on ethical questions related to the Code of Conduct. The Commission publishes annually a report on the application of the Code of Conduct including the work of the independent Ethical Committee.

Commission staff

[121] European Union, 2016. Consolidated version of the Treaty on European Union. TITLE III - PROVISIONS ON THE INSTITUTIONS Article 17 *OJ C 202, 7.6.2016, p. 25–26.* EUR-Lex*:* https://eur-lex.europa.eu/eli/treaty/teu_2016/art_17/oj

[122] European Union, 2012. Consolidated version of the Treaty on the Functioning of the European Union. OJ C326, 26.10.2012, pp. 47-390. EUR-Lex : https://eur-lex.europa.eu/legal-content/EN/TXT/PDF/?uri=CELEX:12012E/TXT&from=EN

[123] European Commission, 2018. Commission Decision of 31 January 2018 on a Code of Conduct for the Members of the European Commission. OJC 65, 21.2.2018, pp. 7-20. EUR-Lex: https://eur-lex.europa.eu/legal-content/EN/TXT/?uri=CELEX:32018D0221(02)

The rules for EU staff has been laid down in the Staff Regulations of Officials (No 31 (EEC), 11 (EAEC).[124] This Regulation prescribes that an official shall carry out his duties and conduct himself solely with the interests of the Union in mind. He shall neither seek nor take instructions from any government, authority, organization or person outside his institution. He shall not accept without the permission of the appointing authority any honour, decoration, favour, gift or payment of any kind, from any government or from any source outside the organization. The appointing authority, shall before recruiting an official, examine whether the candidate has any personal interest which impairs his independence or any other conflict of interest. The candidate has to fill a form by which he informs the authority about any potential conflict of interest. After leaving the office, officials continue to be bound to their duty of integrity and discretion. During the 12 months after leaving, officials are prohibited to engage in lobbying or advocacy against staff of their former institution on matters for which they were responsible during the last three years of employment.

Next to these Staff Regulations, there is a Code for Good Administrative Behaviour[125], which emphasizes the importance of objectivity and impartiality of staff and Guidelines on Gifts and Hospitality for staff members[126].

EU Transparency Register[127]
The European Commission has set up a database of special interest groups whose goal is to influence policy and law-making at the European institutions. Registrants are bound by a Code of Conduct (Annex 3 of the Interinstitutional Agreement)[128], which set out rules for all registrants and establishes the underlying principles for standards of behavior in all relations with the EU institutions.

Commissioners, their cabinet members and Directors-General publish information on meetings held with organizations or self-employed individuals. [129] Meetings related to policy-making and implementation in the EU can only take place if the interest representatives are registered in the EU Transparency Register.

Policies and procedures for using evidence in the development of food and nutrition policies (GOVER 2)

Pilot Projects
From February 2016 till February 2018, the European Parliament funded pilot projects for more effective and efficient action to tackle challenges related to nutrition and physical activity and to help prevent non-communicable diseases among vulnerable people and disadvantaged people in particular. [130] Scientific evidence and policies were reviewed to create a comprehensive evidence-base for the benefit of possible future nutrition and physical activity initiatives.

[124] Regulation No 31 (EEC), 11 (EAEC), laying down the Staff Regulations of Officials and the Conditions of Employment of Other Servants of the European Economic Community and the European Atomic Energy Community, OJ P 045 14.6.1962, p. 1385. EUR-Lex: https://eur-lex.europa.eu/legal-content/EN/TXT/?uri=CELEX%3A01962R0031-20140501

[125] European Commission, 2000. Rules of Procedure of the Commission. OJ L308, 8.12.2000, pp. 26-34. EUR-Lex: https://eur-lex.europa.eu/legal-content/EN/TXT/PDF/?uri=CELEX:32000Q3614&from=EN

[126] Šefčovič , 2012. COMMUNICATION TO THE COMMISSION - Communication from Vice-President Šefčovič to the Commission on Guidelines on Gifts and Hospitality for the staff members. Brussels, 7.3.2012 SEC(2012) 167 final https://ec.europa.eu/info/sites/info/files/communication-to-the-commission-guidelines-on-gifts-and-hospitality_2012_en.pdf

[127] https://ec.europa.eu/info/about-european-commission/service-standards-and-principles/transparency/transparency-register_en
[128] http://ec.europa.eu/transparencyregister/public/staticPage/displayStaticPage.do?locale=en&reference=CODE_OF_CONDUCT
[129] https://ec.europa.eu/info/about-european-commission/service-standards-and-principles/transparency/transparency-register_en
[130] https://ec.europa.eu/health/nutrition_physical_activity/projects/ep_funded_projects_en#fragment4

Expert Group on Health Information

The Expert Group on Health Information (EGHI) is an advisory group for evidence-based policy made up of representatives from EU countries, European Economic Area Countries, possible future EU members and international organisations. [131]

Joint Research Centre

The Joint Research Centre supports EU policies with independent scientific evidence throughout the whole policy cycle, more specifically JRC provides scientific and technical support to EU policies on food, consumer products, chemicals and public health. [132] JRC supports the Strategy on nutrition, overweight and obesity related issues and the EU Action Plan on Childhood Obesity 2014-2020. The Knowledge Gateway on Health Promotion and Disease Prevention and healthy and sustainable diets are major areas of focus of JRC.

JRC produced for example a collection of targeted briefs for policy makers, which evolved into a Health Promotion and Disease Prevention Knowledge Gateway.[133] The Joint Research Centre was also asked to collect information on the national nutritional guidelines. The Food Based Dietary Guidelines have been published on the Knowledge Gateway[134] and presentations on the content have been given both at a Fund for European Aid to the most Deprived (FEAD) event on food aid and a couple of DG AGRI meetings on the School Fruit, Vegetables and Milk scheme.

European Food and Safety Authority

The mission of the European Food and Safety Authority includes providing scientific advice and scientific and technical support on human nutrition in relation to Community legislation and, at the request of the Commission, assistance concerning communication on nutritional issues within the Framework of the Community Health Programme. [135]

OECD

The Commission makes use of scientific evidence coming from the OECD, to provide additional argumentation for Member States in the development of food and nutrition policies. [136] In October 2019 the OECD published the report 'the Heavy Burden of Obesity'. The report makes the urgent economic case to scale up investments in policies to promote healthy lifestyles and tackle this growing global public health problem. The book evaluates a number of policies which could significantly improve health outcomes while being an excellent investment for countries.[137]

DG Health and Food Safety- Directorate Health and food audits and analysis

The Directorate Health and food audits and analysis, also contributed to the development of evidence-based policies, in particular, through the increased country knowledge available. The

[131] https://ec.europa.eu/health/indicators_data/eghi_en

[132] https://ec.europa.eu/jrc/en/about/jrc-in-brief

[133] European Commission, DG Health and Food Safety, 2019. Initiatives on nutrition and physical activity. https://ec.europa.eu/health/sites/health/files/nutrition_physical_activity/docs/2019_initiatives_npa_en.pdf. https://ec.europa.eu/jrc/en/health-knowledge-gateway/promotion-prevention/nutrition/food-based-dietary-guidelines

[134] https://ec.europa.eu/jrc/en/health-knowledge-gateway/promotion-prevention/nutrition/food-based-dietary-guidelines

[135] REGULATION (EC) No 178/2002 OF THE EUROPEAN PARLIAMENT AND OF THE COUNCIL of 28 January 2002 laying down the general principles and requirements of food law, establishing the European Food Safety Authority and laying down procedures in matters of food safety. OJ L31, 1.2.2002, pp. 1-24. EUR-Lex: https://eur-lex.europa.eu/legal-content/EN/TXT/PDF/?uri=CELEX:32002R0178&from=EN.

[136] European Commission, DG Health and Food Safety, 2019. Initiatives on nutrition and physical activity. https://ec.europa.eu/health/sites/health/files/nutrition_physical_activity/docs/2019_initiatives_npa_en.pdf.

[137] OECD, 2019. The Heavy Burden of Obesity: The Economics of Prevention. Published on October 10, 2019. https://www.oecd.org/health/the-heavy-burden-of-obesity-67450d67-en.htm

knowledge is presented in 'country profiles' which outline the performance of Member States and provide country specific knowledge. [138]

Other initiatives[139]

Further initiatives at EU level to use evidence in the development of food and nutrition policies are:

(1) The collection of validated best practices from Member States through a Best Practice portal to support implementation since 2017.[140]
(2) Multiple workshops on reformulation have been organized as well as one workshop on food taxation, presenting the latest scientific evidence on impact (OECD and the WHO), legal advice from other services and real life experiences from Member States

Policies and procedures for ensuring transparency in the development of food and nutrition policies (GOVER 3)

The general principle of transparency has been laid down in the Treaty on the Functioning of the European Union.[141] Article 15 states that in order to promote good governance and ensure the participation of civil society, the Union's institutions, bodies, offices and agencies shall conduct their work as openly as possible.

The Commission's Better Regulation Agenda was set up in 2015 to achieve better results, by opening up policy and law-making and listen more to people it affects. [142] Transparency in the preparation and reviewing of policies, in the legislative process and in public consultations is a very important theme of this Agenda.

Regulations related to food and nutrition policies also include text phrases related to transparency. The Regulation on the provision of food information to consumers[143] and the General Food Law[144] prescribe that during the preparation, evaluation and revision of food (information) law an open

[138] European Commission, DG Health and Food Safety, 2018. Health and Food Audits and Analysis Programme 2019. Luxembourg: Publications Office of the European Union. doi:10.2875/469690 https://ec.europa.eu/food/sites/food/files/hfaa_progr_en_2019.pdf

[139] European Commission, DG Health and Food Safety, 2019. Initiatives on nutrition and physical activity. https://ec.europa.eu/health/sites/health/files/nutrition_physical_activity/docs/2019_initiatives_npa_en.pdf.

[140] https://webgate.ec.europa.eu/dyna/bp-portal/index.cfm

[141] European Union, 2012. Consolidated version of the Treaty on the Functioning of the European Union. OJ C326, 26.10.2012, pp. 47-390. EUR-Lex : https://eur-lex.europa.eu/legal-content/EN/TXT/PDF/?uri=CELEX:12012E/TXT&from=EN

[142] European Commission, 2015. COMMUNICATION FROM THE COMMISSION TO THE EUROPEAN PARLIAMENT, THE COUNCIL, THE EUROPEAN ECONOMIC AND SOCIAL COMMITTEE AND THE COMMITTEE OF THE REGIONS Better regulation for better results - An EU agenda. COM/2015/0215 final. EUR-Lex: https://eur-lex.europa.eu/legal-content/EN/TXT/?qid=1451989021436&uri=CELEX:52015DC0215

[143] REGULATION (EU) No 1169/2011 OF THE EUROPEAN PARLIAMENT AND OF THE COUNCIL of 25 October 2011 on the provision of food information to consumers, amending Regulations (EC) No 1924/2006 and (EC) No 1925/2006 of the European Parliament and of the Council, and repealing Commission Directive 87/250/EEC, Council Directive 90/496/EEC, Commission Directive 1999/10/EC, Directive 2000/13/EC of the European Parliament and of the Council, Commission Directives 2002/67/EC and 2008/5/EC and Commission Regulation (EC) No 608/2004. OJ L 304, 22.11.2011, pp. 18-63. EUR-Lex : https://eur-lex.europa.eu/LexUriServ/LexUriServ.do?uri=OJ:L:2011:304:0018:0063:EN:PDF

[144] REGULATION (EC) No 178/2002 OF THE EUROPEAN PARLIAMENT AND OF THE COUNCIL of 28 January 2002 laying down the general principles and requirements of food law, establishing the European Food Safety Authority and laying down procedures in matters of food safety. OJ L 31, 1.2.2002, pp. 1-24. EUR-Lex: https://eur-lex.europa.eu/legal-content/EN/TXT/PDF/?uri=CELEX:32002R0178&from=EN

and transparent public consultation shall be conducted. The Regulation on nutrition and health claims[145] stipulates the establishment of a public register of claims[146] for the sake of transparency.

The evaluation of the General Food Law[147] concludes that EFSA has been highly transparent since it's operation. However, with regard to risk assessment, EFSA is bound to strict confidentiality rules laid down in the General Food Law and some sectorial acts, which creates a perception of a certain lack of transparency, which is further reinforced by civil society's concern over EFSA's independence from industrial interests, as studies conducted by the industry form a big part (but are not the only part) of the basis of EFSA's risk assessment. Following these conclusion the Commission has put forward a proposal for revising the General Food Law and eight other sectorial acts to increase transparency and sustainability of the risk assessments. This new Regulation has been published the 6th of September 2019 and will start applying as of 27 March 2021.[148]

Following negotiations between the EU institutions[149]
The European Parliament provides a "Legislative Observatory" (online at http://www.europarl.europa.eu/oeil/home/home.do) to enable the process of a particular legislative proposal to be followed in detail.

Public access to comprehensive nutrition information and key documents for the public (GOVER 4)
The general rights of individuals to access documents of EU bodies are laid down in Article 15 of the Treaty on the Functioning of the European Union[150] and Article 42 of the Charter of Fundamental Rights of the EU[151] and implemented through the Regulation (No 1049/2001) regarding public access[152] to European Parliament, Council and Commission documents. Article 2 of that Regulation prescribes that any citizen of the Union and any person residing in a Member State, has a right to access documents of the European Union institutions. There are some exceptions, which are for example documents where disclosure would undermine the protection of public interests, privacy and integrity, commercial interests, court proceedings and the purpose of audits/inspections/investigations. Different types of documents that are available online are official documents and publications (e.g. agenda's, meeting minutes, reports), legislative documents, responses to petitions sent to Commissioners. Registers and databases that are accessible are the financial transparency system, the transparency register and the documents on EU institutional issues.

[145] REGULATION (EC) No 1924/2006 OF THE EUROPEAN PARLIAMENT AND OF THE COUNCIL of 20 December 2006 on nutrition and health claims made on foods. OJ L 404. 30.12.2006, pp. 9-25. EUR-Lex: https://eur-lex.europa.eu/legal-content/EN/TXT/PDF/?uri=CELEX:32006R1924&from=en

[146] http://ec.europa.eu/food/safety/labelling_nutrition/claims/register/public/?event=register.home

[147] COMMISSION STAFF WORKING DOCUMENT THE REFIT EVALUATION of the General Food Law (Regulation (EC) No 178/2002) { 15.1.2018 SWD(2018) 38 final PART 1/2. https://ec.europa.eu/food/sites/food/files/gfl_fitc_comm_staff_work_doc_2018_part1_en.pdf

[148] European Union, 2019. REGULATION (EU) 2019/1381 OF THE EUROPEAN PARLIAMENT AND OF THE COUNCIL of 20 June 2019 on the transparency and sustainability of the EU risk assessment in the food chain and amending Regulations (EC) No 178/2002, (EC) No 1829/2003, (EC) No 1831/2003, (EC) No 2065/2003, (EC) No 1935/2004, (EC) No 1331/2008, (EC) No 1107/2009, (EU) 2015/2283 and Directive 2001/18/EC. OJL 231, 6.9.2019, pp.1-28. https://eur-lex.europa.eu/legal-content/EN/TXT/PDF/?uri=CELEX:32019R1381

[149] Scott L. Greer, Nick Fahy, Heather A. Elliott, Matthias Wismar, Holly Jarman, Willy Palm, World Health Organization 2014, European Observatory on Health Systems and Policies. Everything you always wanted to know about European Health policies but were afraid to ask. United Kingdom: London.

[150] European Union, 2012. Consolidated version of the Treaty on the Functioning of the European Union. OJ C326, 26.10.2012, pp. 47-390. EUR-Lex: https://eur-lex.europa.eu/legal-content/EN/TXT/PDF/?uri=CELEX:12012E/TXT&from=EN

[151] European Union, 2000. CHARTER OF FUNDAMENTAL RIGHTS OF THE EUROPEAN UNION. OJ C 364, 18.12.2000, pp. 1-22. EUR-Lex: https://www.europarl.europa.eu/charter/pdf/text_en.pdf

[152] REGULATION (EC) No 1049/2001 OF THE EUROPEAN PARLIAMENT AND OF THE COUNCIL of 30 May 2001 regarding public access to European Parliament, Council and Commission documents. OJ L 145, 31.5.2001, pp. 43-48. EUR-Lex : https://eur-lex.europa.eu/legal-content/EN/TXT/PDF/?uri=CELEX:32001R1049&from=EN

Regarding Public access to nutrition and health data, provisions have been included in several regulations. The Regulation on nutrition and health claims[153] prescribes for example the establishment and maintenance of a Register on nutrition and health claims[154], which is available online. The General Food Law[155] contains an article which prescribes that EFSA shall ensure wide access to the documents it possesses. With the revision of the General Food Law, citizens will have automatic access to all studies and information submitted by the industry in the risk assessment process.

EUROSTAT is the main source of EU Health data. Health data is based on the Regulation on Community Statistics on public health and health and safety at work (No1338/2008)[156]. This Regulation prescribes that the Commission (Eurostat) shall take the necessary steps to improve the dissemination, accessibility and documentation of statistical information. Eurostat's dissemination policy is characterized by free access to European statistics for all users, which is together with other principles laid down in Regulation on European statistics (No 223/2009)[157] and in the European Statistics Code of Practice. [158]

Joint Action on Health Information

The Joint Action on Health Information has been established in 2018 to facilitate the development of a sustainable EU health information infrastructure by improving the availability of comparable, robust and policy-relevant health data and health system performance information. [159]

Comments/notes

DOMAIN 10 - MONITORING AND INTELLIGENCE – This domain concerns the extent to which the EU's monitoring and intelligence systems (surveillance, evaluation, research and reporting) are comprehensive and regular enough to assess the status of food environments, population nutrition and diet-related NCDs and their inequalities, and to measure progress on achieving the goals of nutrition and health plans.

MONIT1 Monitoring systems, implemented by the EU, are in place to regularly monitor food environments(especially for food composition for nutrients of concern, food promotion to children, and nutritional quality of food in schools and other public sector settings), against codes / guidelines / standards / targets

[153] REGULATION (EC) No 1924/2006 OF THE EUROPEAN PARLIAMENT AND OF THE COUNCIL of 20 December 2006 on nutrition and health claims made on foods. OJ L 404, 30.12.2006, pp. 9-25. EUR-Lex: https://eur-lex.europa.eu/legal-content/EN/TXT/PDF/?uri=CELEX:32006R1924&from=en

[154] http://ec.europa.eu/food/safety/labelling_nutrition/claims/register/public/?event=register.home

[155] European Commission, 2018. COMMISSION STAFF WORKING DOCUMENT THE REFIT EVALUATION of the General Food Law (Regulation (EC) No 178/2002) {SWD(2018) 37 final}. Brussels, 15.1.2018 SWD(2018) 38 final PART 1/2 . https://ec.europa.eu/food/sites/food/files/gfl_fitc_comm_staff_work_doc_2018_part1_en.pdf

[156] REGULATION (EC) No 1338/2008 of the EUROPEAN PARLIAMENT and of the COUNCIL of 16 December 2008 on Community statistics on public health and health and safety at work. OJ L 354, 31.12.2008, pp. 70-81. EUR-Lex: https://eur-lex.europa.eu/legal-content/EN/TXT/PDF/?uri=CELEX:32008R1338&from=EN

[157] REGULATION (EC) No 223/2009 OF THE EUROPEAN PARLIAMENT AND OF THE COUNCIL of 11 March 2009 on European statistics and repealing Regulation (EC, Euratom) No 1101/2008 of the European Parliament and of the Council on the transmission of data subject to statistical confidentiality to the Statistical Office of the European Communities, Council Regulation (EC) No 322/97 on Community Statistics, and Council Decision 89/382/EEC, Euratom establishing a Committee on the Statistical Programmes of the European Communities. OJ L 87, 31.3.2009, pp. 164-173. EUR-Lex: https://eur-lex.europa.eu/legal-content/EN/TXT/PDF/?uri=CELEX:32009R0223&from=EN.

[158] EUROSTAT, 2017. European Statistics Code of Practice, Revised Edition 2017. Publication Office of the European Union 2018. doi:10.2785/798269. https://ec.europa.eu/eurostat/web/products-catalogues/-/KS-02-18-142

[159] https://webgate.ec.europa.eu/chafea_pdb/health/projects/801553/summary

Definitions and scope

- ➢ Includes monitoring systems funded fully or in part by government that are managed by an academic institution or other organisation
- ➢ Includes regular monitoring and review of the impact of policies implemented by the EU on food environments and described in the policy domains above), in particular:
    - Monitoring of compliance with voluntary food composition standards related to nutrients of concern in out-of-home meals (as defined in the 'Food composition' domain)
    - Monitoring of compliance with food labelling regulations (as defined in the 'Food labelling' domain above)
    - Monitoring of unhealthy food promoted to children via broadcast and non-broadcast media and in children's settings (as defined in the 'Food promotion' domain above)
    - Monitoring of compliance with food provision policies in schools, early childhood services and public sector settings (as defined in the 'Food provision' domain above)

MONIT2 There is regular monitoring of adult and childhood nutrition status and population intakes against specified intake targets or recommended daily intake levels

Definitions and scope

- Includes monitoring of adult and child intake in line with X Countries Food Guide and dietary recommendations
- Includes monitoring of adult and child intake of nutrients of concern and noncore/discretionary foods including sugar-sweetened beverages (even if there are no clear intake targets for all of these)
- 'Regular' is considered to be every five years or more frequently

MONIT3 There is regular monitoring of adult and childhood overweight and obesity prevalence using anthropometric measurements

Definitions and scope

- Anthropometric measurements include height, weight and waist circumference
- 'Regular' is considered to be every five years or more frequently

MONIT4 There is regular monitoring of the prevalence of NCD metabolic risk factors and occurrence rates (e.g. prevalence, incidence, mortality) for the main diet-related NCDs

Definitions and scope

- Diet-related NCD risk factors and NCDs include, amongst others, hypertension, hypercholesterolemia, Type 2 Diabetes, cardiovascular disease (including ischaemic heart disease, cerebrovascular disease and other diseases of the vessels), diet-related cancers.
- May be collected through a variety of mechanisms such as population surveys or a notifiable diseases surveillance system.

MONIT5 Major programs and policies are regularly evaluated to assess their effectiveness and contributions to achieving the goals of the nutrition and health plans

Definitions and scope

- Includes any policies, guidelines, frameworks or tools that are used to determine the depth and type (method and reporting) of evaluation required
- Includes a comprehensive evaluation framework and plan that aligns with the key preventive health or nutrition implementation plan
- The definition of a major programs and policies is to be defined by the relevant EU department
- Evaluation should be in addition to routine monitoring of progress against a project plan or program logic

MONIT6 Progress towards reducing health inequalities or health impacts in vulnerable populations and social and economic determinants of health are regularly monitored.

Definitions and scope

- Monitoring of overweight and obesity and main diet-related NCDs includes stratification or analysis of population groups where there are the greatest health inequalities including Indigenous peoples and socio-economic strata
- Includes reporting against targets or key performance indicators related to health inequalities

Policy evidence summary

Monitoring systems are in place to regularly monitor food environments against codes/guidelines/standards/targets (MONIT 1)

According to the EU Action Plan on Childhood Obesity 2014-2020[160], Member States are responsible for implementing monitoring mechanisms like national food composition databases. The WHO is, according to the plan, responsible for collecting from the Member States on the monitored initiatives, e.g. via the WHO Nutrition, Obesity and Physical Activity (NOPA) database and the WHO Health Behaviour among Schoolchildren (HBSC) and for Health-Promoting Schools surveys.

Monitoring of compliance with food composition standards

EFSA has a food composition database,[161] which gives information on the amount of vitamins and minerals contained in foods but not on the nutrients of concern. However, there have been some developments at EU level.

The Joint Action on Nutrition and Physical Activity (JANPA) project which ran from September 2015 till November 2017 conducted a pilot study on food composition monitoring, which highlighted the importance of a European harmonized monitoring tool (referring to the French “Oqali” tool as a good example) and setting up a monitoring network. [162] Currently, a new Joint Action is being

[160] European Union, 2014. EU Action Plan on Childhood Obesity 2014-2020. 24 February 2014. https://ec.europa.eu/health/sites/health/files/nutrition_physical_activity/docs/childhoodobesity_actionplan_2014_2020_en.pdf

[161] https://www.efsa.europa.eu/en/data/food-composition and https://www.efsa.europa.eu/en/microstrategy/food-composition-data

[162] http://www.janpa.eu/outcomes/Deliverables/4_Scheda%20inform_Facsheet%205.2_V9_2p_210x297_JANPA.pdf

prepared to start in 2020, which will adapt and implement practices that have already proven to work in the three areas: reformulation, marketing and public procurement.[163] The Joint Action will implement a European Standardised Monitoring system for the reformulation of processed foods.

In 2017, the European Commission (the Consumers, Health, Agriculture and Food Executive Agency) commissioned a pilot database on the nutritional characteristics of food products in the EU to help monitor whether food products have increasingly less (or increasingly more) salt, fat or sugars.[164] ICF is leading in a partnership which will tackle this challenge by collecting data on the ingredients and nutritional content of more than 50,000 processed food products in 16 European countries.[165] The database will inform authorities, consumers and industry about the scope for improvements in food products. Since «what gets measured gets done», this can strengthen national reformulation initiatives and support consumer choice, innovation and a level playing field for industry.

Monitoring of compliance with food labelling regulations

** The evidence description is this paragraph has not been checked yet by relevant EU governmental officials/experts*

Compliance with the food labelling regulations on food information to consumers and health and nutrition claims is mainly regulated by the Official Controls Regulation (EU) 2017/625[166] and Regulation (EC) No 882/2004[167] (As this report is currently being drafted both regulations are still in force). The Regulation stipulates that it is the responsibility of Member States to designate the competent authority or authorities on which they confer the responsibility to organize or perform official controls and other activities (article 4). Regarding the monitoring of nutrition and health claims, Article 26 of the Regulation (EC) No 1924/2006 on nutrition and health claims[168] states further that Member States may require the manufacturer or person placing foods on the market to notify the competent authority of that placing on the market by forwarding to it a model of the label used for the product. Regarding additional forms of expression and presentation of nutrition labelling, Article 35 of the Regulation on the provision of food information to consumers[169], states that Member States may require food business operators to notify the competent authority of the use of an additional form of expression or presentation and to provide them with the relevant justifications.

The Commission produces reports on the overall operation of official controls in EU countries, in which she incorporates information based on the annual reports submitted by the national

[163]European Commission, 2019. Initiatives on Nutrition and Physical acitivity. https://ec.europe.eu/health/sites/health/files/nutrition_physical_activity/docs/2019_initiatives_npa_en.pdf

[164] European Commission, 2019. Initiatives on Nutrition and Physical Activity. https://ec.europa.eu/health/sites/health/files/nutrition_physical_activity/docs/2019_initiatives_npa_en.pdf and https://etendering.ted.europa.eu/cft/cft-display.html?cftId=3068

[165] https://www.linkedin.com/pulse/eu-investing-data-learning-needed-understand-increase-andrew-jarvis/

[166] REGULATION (EU) 2017/625 OF THE EUROPEAN PARLIAMENT AND OF THE COUNCIL of 15 March 2017 on official controls and other official activities performed to ensure the application of food and feed law, rules on animal health and welfare, plant health and plant protection products. OJ L 95, 7.4.2017, pp.1-142. EUR-Lex: https://eur-lex.europa.eu/legal-content/EN/TXT/PDF/?uri=CELEX:32017R0625&from=EN

[167] REGULATION (EC) No 882/2004 OF THE EUROPEAN PARLIAMENT AND OF THE COUNCIL of 29 April 2004 on official controls performed to ensure the verification of compliance with feed and food law, animal health and animal welfare rules. OJ L 191, 28.5.2004, p. 1-59. EUR-Lex: https://eur-lex.europa.eu/LexUriServ/LexUriServ.do?uri=CONSLEG:2004R0882:20060525:EN:PDF

[168] REGULATION (EC) No 1924/2006 OF THE EUROPEAN PARLIAMENT AND OF THE COUNCIL of 20 December 2006 on nutrition and health claims made on foods. OJ L 404, 30.12.2006, pp.9-25. EUR-Lex: https://eur-lex.europa.eu/legal-content/EN/TXT/PDF/?uri=CELEX:32006R1924&from=en

[169] REGULATION (EU) No 1169/2011 OF THE EUROPEAN PARLIAMENT AND OF THE COUNCIL of 25 October 2011 on the provision of food information to consumers

authorities on their control activities and the outcome of Commission controls carried out in the Member States. [170] The most recent report covers the years 2014-2016.
Furthermore, DG Health and Food Safety (Directorate on Health and Food Audits and Analysis) contributes to the effective implementation of food legislation by conducting Audits and Analysis.[171] [172]

Monitoring of compliance with food provision policies in schools, early childhood services and public sector settings
Establishing a harmonized monitoring of school nutrition in the EU (primary and secondary schools) is an objective in the EU Action Plan on Childhood Obesity 2014-2020[173], for which Member States are according to the plan responsible. Euridyce, which is a source of information on education systems and policies in 38 countries (established by the European Commission and Member states in 1980) is mentioned as a possible monitoring tool for this. School nutrition indicators could be defined and implemented to the Eurydice.

In addition to this the EU Action Plan on Childhood Obesity, refers to the WHO Health Behaviour among Schoolchildren and for Health-Promoting Schools (HBSC) surveys for monitoring. These surveys, which collect data every four years on 11-, 13- and 15-years old in 49 countries and regions, include questions around the school environment.

The new Joint Action project, which will start in 2020, will also cover the monitoring of the improvement of public procurement of food.[174] A prototype catalogue of food will be tested in the public procurement procedure to contribute to the higher quality of menus by assuring transparent quality of the procured foods within public institutions.

We have not found any information on the monitoring of compliance with food provision policies in other public sector settings.

Monitoring of unhealthy food promoted to children
Article 9 of the Audiovisual Media Services Directive[175] prescribes that Member States adopt code of conduct, which aim to reduce the exposure of children to unhealthy food promotion and which shall provide for regular, transparent and independent monitoring and evaluation of the objectives aimed at. In addition, these code of conducts shall also provide for effective enforcement including effective and proportionate sanctions.

In addition to this, there are some monitoring initiatives at EU level: (1) DG Sante and DG CNECT launched in 2017 a study on the exposure of minors to TV and online marketing of unhealthy foods (results will be available in May 2020) and (2) JRC produced (released 18 December 2019) a toolkit

[170] https://ec.europa.eu/food/audits_analysis/annual_reports_en
[171] https://ec.europa.eu/food/sites/food/files/hfaa_progr_en_2019.pdf
[172] DG Sante Strategic Plan 2016-2020
[173] European Union, 2014. EU Action Plan on Childhood Obesity 2014-2020. 24 February 2014. https://ec.europa.eu/health/sites/health/files/nutrition_physical_activity/docs/childhoodobesity_actionplan_2014_2020_en.pdf
[174] European Commission, 2019. Initiatives on Nutrition and Physical Activity. https://ec.europa.eu/health/sites/health/files/nutrition_physical_activity/docs/2019_initiatives_npa_en.pdf

[175] DIRECTIVE (EU) 2018/1808 OF THE EUROPEAN PARLIAMENT AND OF THE COUNCIL of 14 November 2018 amending Directive 2010/13/EU on the coordination of certain provisions laid down by law, regulation or administrative action in Member States concerning the provision of audiovisual media services (Audiovisual Media Services Directive) in view of changing market realities. OJ L 303, 28.11.2018, p. 69–92. EUR-Lex: https://eur-lex.europa.eu/eli/dir/2018/1808/oj

to support Member States in developing and updating codes of conduct on marketing of food, non-alcoholic and alcoholic beverages.[176] [177]

The new Joint Action, starting in 2020, will also cover monitoring activities on the reduction of aggressive marketing to children of foods high in fat, salt and sugar.[178] The Joint Action aims to deliver a harmonised EU approach to reducing unhealthy (digital) food marketing to children and adolescents and to use already developed tools for harmonised monitoring of (digital) marketing.

Monitoring of adult and childhood nutrition status and population intakes (MONIT 2), overweight and obesity prevalence (MONIT 3) and the prevalence of NCD metabolic risk factors and occurrence rates for the main diet-related NCDs (MONIT 4)
There are different initiatives at EU level, regarding monitoring of adult and childhood nutrition status and population intakes, overweight and obesity prevalence and the prevalence of NCD metabolic risk factors and occurrence rates for the main diet-related NCDs.

Regarding the monitoring of adult and childhood intakes, the European Commission notes that this is clearly the responsibility of the Member States.

EFSA
EFSA has a database, 'the Comprehensive European Food Consumption Database'[179] which contains data on food consumption habits and patterns across the EU.
Member States used different methods to collect food consumption data. However, as can be concluded from information on the European Commission's website it is still difficult to draw conclusions and make comparisons on the intake of for example sugars, salt and fat across the EU, due to differences in methodology. Therefore, in 2011, EFSA launched the EU Menu project, which aims to provide standardized, harmonized information on what people eat in all countries and regions across the EU.[180] Under this project, EFSA funded 32 surveys for different age groups across Europe. Data from all surveys are expected to become available by 2023.

Eurostat- European Health Interview Survey (EHIS)[181]
The European Health Interview Survey, coordinated by Eurostat, aims at measuring on a harmonized basis with a high degree of comparability, the health status, health determinants and access to health care services of EU citizens. EHIS is to be conducted every five years [182] and includes health status (e.g. self-perceived health, chronic diseases, limitation in activities, mental health, pain, accidents, etc.) and health determinants like height and weight, the consumption of fruits, vegetables and juice.

Eurostat- Health in the European Union- facts and figures[183]

[176] European Commission, DG Health and Food Safety, 2019. Initiatives on Nutrition and Physical Activity. https://ec.europa.eu/health/sites/health/files/nutrition_physical_activity/docs/2019_initiatives_npa_en.pdf
[177] https://ec.europa.eu/jrc/en/news/toolkit-limit-marketing-food-non-alcoholic-and-alcoholic-beverages-minors
[178] European Commission, 2019. Initiatives on Nutrition and Physical Activity. https://ec.europa.eu/health/sites/health/files/nutrition_physical_activity/docs/2019_initiatives_npa_en.pdf
[179] https://www.efsa.europa.eu/en/data/food-consumption-data
[180] https://www.efsa.europa.eu/en/data/food-consumption-data
[181] European Commission, Eurostat. European Health Interview Survey. https://ec.europa.eu/eurostat/cache/metadata/en/hlth_det_esms.htm
[182] This periodicity will change to 6 years for future EHIS waves that will be conducted under the new Regulation (EU) 2019/1700 of the European Parliament and of the Council establishing a common framework for European statistics relating to persons and households, based on data at individual level collected from samples.
[183] European Commission, Eurostat. Health in the European Union- facts and figures. https://ec.europa.eu/eurostat/statistics-explained/index.php?title=Health_in_the_European_Union_%E2%80%93_facts_and_figures

Eurostat publishes the data on NCD's on their website and reports on it in the 'Health in the European Union- facts and figures'. Indicators reported on are for example: cardiovascular diseases, cancers, and respiratory diseases.

Eurostat- Sustainable development indicators
Eurostat monitors yearly policies contributing to the UN Sustainable Development Goals, based on the EU SDG indicator set. [184] Indicators selected for Goal 3 on health and well-being and are for example: Life expectancy at birth, Self-perceived health *and* death rate due to chronic diseases (included in the indicator are malignant neoplasms, diabetes mellitus, ischaemic heart diseases, cerebrovascular diseases, chronic lower respiratory diseases and chronic liver diseases).

European Core Health Indicators (ECHI) [185]
The ECHI data tool, contains 88 indicators, resulted from the European Community Health Indicators Monitoring (ECHIM) project. These include health determinant indicators on Body Mass Index and the consumption of fruit and vegetables and indicators on health status like diabetes (self-reported and register-based prevalence) and cancer incidence.

State of Health
The State of Health is a two-year initiative undertaken by the European Commission, that provides policy makers, interest groups, and health practitioners with factual, comparative data and insights into health and health systems in EU countries. [186] Country Health Profiles are drafted that may report on health status like the prevalence of overweigh and obesity (based on self-reported data), cancer, cardiovascular diseases and diabetes.

JRC- European Cancer Information System (ECIS)[187]
The European Cancer Information System (ECIS) by JRC provides the latest information on indicators that quantify cancer burden across Europe. It permits the exploration of geographical patterns and temporal trends of incidence, mortality and survival data across Europe for the major cancer entities. [188]

WHO COSI, NOPA database and WHO HBSC surveys
In the EU Action Plan on Childhood Obesity 2014-2020[189] is referred to the WHO, as being responsible for collecting from the Member States on the monitored initiatives, e.g. via the WHO European Childhood Obesity Surveillance Initiative (COSI), WHO Nutrition, Obesity and Physical Activity (NOPA) and the WHO Health Behaviour in School Aged Children (HBSC) surveys.

The WHO COSI [190] objectively measures trends in overweight and obesity among primary school aged children (6-9 years). It's a survey based on nationally representative samples and takes standardized weight and height measurements and collects information on school environments and dietary habits.

---

[184] European Commission, Eurostat, 2017. EU SDG INDICATOR SET Final version of 28 April 2017 as agreed with Commission Services, which received a favourable opinion by the European Statistical System Committee at its meeting of 17-18 May 2017. https://ec.europa.eu/eurostat/documents/276524/7736915/EU-SDG-indicator-set-with-cover-note-170531.pdf
[185] https://ec.europa.eu/health/indicators/echi/list_en
[186] https://ec.europa.eu/health/state/summary_en
[187] https://ecis.jrc.ec.europa.eu/
[188] https://ecis.jrc.ec.europa.eu/
[189] European Union, 2014. EU Action Plan on Childhood Obesity 2014-2020. 24 February 2014. https://ec.europa.eu/health/sites/health/files/nutrition_physical_activity/docs/childhoodobesity_actionplan_2014_2020_en.pdf
[190] http://www.euro.who.int/en/health-topics/disease-prevention/nutrition/activities/who-european-childhood-obesity-surveillance-initiative-cosi/about-cosi

The WHO NOPA database[191] compiles information to monitor progress on nutrition, diet, physical activity and obesity. The Country information contains surveillance data, policy documents, action to implement policy and examples of good practice in programmes and interventions.

These WHO HBSC surveys, which collect self-reported data every four years on 11-, 13- and 15-years old in 49 countries and regions, include questions around areas like eating behaviours, obesity, the socio- economic environment and the school environment. [192]

Integrated surveillance on NCD's (iNCD)
The WHO has started the Integrated surveillance on NCD's project in 2013 which was co-financed by the EU. The project aimed to describe how Member States of the European Union can optimize their use of the ECHI indicators to report on progress towards reaching the nine global targets of the Global Monitoring Framework on NCDs[193], including the targets:

- A 25% relative reduction in the overall mortality from cardiovascular diseases, cancer, diabetes, or chronic respiratory diseases
- A 30% relative reduction in mean population intake of salt/sodium
- A 25% relative reduction in the prevalence of raised blood pressure or contain the prevalence of raised blood pressure, according to national circumstances
- Halt the rise in diabetes & obesity

Evaluation of major programs and policies to assess their effectiveness and contributions to achieving the goals of the nutrition and health plans (MONIT 5)
In the Strategic Plan of DG Sante 2016-2020 it is emphasized that Better Regulation is a key horizontal priority for the Commission. [194] Impact assessments, public consultations, ex-post evaluations and Fitness Checks are instruments for the Commission to ensure the regulatory cycle is transparent and of good quality. The Commission published Guidelines on evaluation and Fitness Checks.[195] A Fitness Check is a type of evaluation that assesses several related actions like the interaction of laws, policies and programmes.

The Guidelines state about the periodicity of evaluations:
"*In general, it is recommended to evaluate only once sufficient time has elapsed from the implementation of the intervention to allow at least 3 years of reasonably full data to be collected. See Tool #45 on How to undertake a proportionate evaluation."*

Evaluations or fitness checks that have been completed or are in progress, are:

- Monitoring the activities of the EU Platform for Action on Diet, Physical Activity and Health (annual monitoring reports and an assessment report of the EU Platform (planned to be published in December 2019))
- Evaluation of the implementation of the Strategy for Europe on Nutrition, Overweight and Obesity related issues (covering 2007-2011, report published in 2013)
- Mid-term evaluation of the third Health Programme 2014-2020 (covering 2016-2017)
- REFIT Evaluation of the General Food Law (published in 2018)
- Supporting the mid-term evaluation of the EU Action Plan on Childhood Obesity 2014-2020 (covering 2014-2017, published in 2018)

---

[191] http://data.euro.who.int/nopa/
[192] http://www.hbsc.org/about/index.html
[193] WHO, Regional Office for Europe. Integrated surveillance of Noncommunicable Diseases (iNCD) A project co-financed with the European Commission. http://www.euro.who.int/__data/assets/pdf_file/0018/253422/WHO-iNCD-June-2014-WEBnew-3.pdf?ua=1
[194] European Commission. Strategic Plan 2016-2020 DG Health and Food Safety. Ref. Ares(2017)6260978 - 20/12/2017. https://ec.europa.eu/info/sites/info/files/strategic-plan-2016-2020-dg-sante_en_0.pdf
[195] European Commission. Better Regulation guidelines. Chapter VI Guidelines on evaluation (including fitness checks). https://ec.europa.eu/info/sites/info/files/better-regulation-guidelines-evaluation-fitness-checks.pdf

- REFIT: Evaluation of the Regulation on nutrition and health claims (started in 2016, in progress)
- Monitoring of the EU Fruit and Vegetable Scheme (countries provide annual monitoring reports, Evaluation reports cover five school years and will be available from 1 March 2023)

Progress towards reducing health inequalities or health impacts in vulnerable populations and monitoring of social and economic determinants of health (MONIT 6)

Different monitoring tools at EU level include background variables on demography and socio-economic status and/or report on health or socio-economic inequalities.

EU Menu project- harmonising collection of food consumption data

EFSA's EU Menu project (2011-2023), aims to provide standardised information on what people eat in all countries and regions across the EU. The guidance on the EU menu methodology[196] provides recommendations on how to collect harmonised and high quality data (in age groups ranging from three months to 74 years) on food consumption information, weight, height, physical activity levels and background information of the participants. The guidance recommends to stratify the sample at least by age group and sex and where possible by region of residence, urban and rural areas, seaside and countryside area, ethnic groups and household size. The guidance further recommends to consider and clearly describe variability in dietary patterns, because of regional, socio-economic, ethnic or other differences.

European Health Interview Survey (EHIS) **[197]**

The European Health Interview Survey (EHIS) includes background variables on demography and socio-economic status. All indicators are expressed as percentages within the population and statistics are broken down by age groups and sex and one other dimension such as educational attainment level, income quintile group or degree of urbanization .

State of Health [198]

Countries report in their Country Health Profile on the socio-economic inequalities (people with different education or income levels) in their country (based on self-reported data). The Companion report published along with the Country Health Profiles reports on health inequalities, like life expectancy in the different countries.

European Core Health Indicators (ECHI) [199]

The ECHI data tool, includes indicators related to demography and socio-economic situation, e.g. population by education, population by occupation, total unemployment, population below poverty line and income inequality. It further contains health status indicators, like life expectancy by educational attainment and healthy life expectancy.

Integrated surveillance on NCD's (iNCD) [200]

iNCD reviewed key international databases, assessing the completeness and quality of indicators. Furthermore the iNCD project assessed the comparability and availability of data broken down by age, sex and/or socio-economic risk factors in order to address inequalities.

---

[196] https://www.efsa.europa.eu/en/efsajournal/pub/3944
[197] Eurostat. European Health Interview Survey. https://ec.europa.eu/eurostat/cache/metadata/en/hlth_det_esms.htm
[198] https://ec.europa.eu/health/state/summary_en
[199] https://ec.europa.eu/health/indicators/echi/list_en
[200] WHO, Regional Office for Europe. Integrated surveillance of Noncommunicable Diseases (iNCD) A project co-financed with the European Commission. http://www.euro.who.int/__data/assets/pdf_file/0018/253422/WHO-iNCD-June-2014-WEBnew-3.pdf?ua=1

Evaluations of major nutrition and health programs and policies
Evaluations of major nutrition and health programs and policies at EU level have addressed health and/or socio-economic inequalities.

The Evaluation of the implementation of the Strategy for Europe on nutrition, overweight and obesity related issues, [201] states that EU-level initiatives in the area of social inequalities in obesity have mainly focused on strengthening the evidence base, EU added value in addressing the social dimension in overweight and obesity have been limited. The report recommends the EU to take a careful consideration of effects on lower socio-economic groups to ensure that initiatives do not further exacerbate health inequalities. The evaluation mentions a few key policy interventions which are particularly effective in reducing social inequalities in obesity, like school fruit and vegetables schemes, fat taxes and policies relevant to the life-course approach. Less effective interventions are information campaigns and food labelling interventions.

The mid-term evaluation of the EU Action Plan on Childhood Obesity 2014-2020[202] used interviews to obtain relevant policy developments, of which one of the questions was about how health inequalities are addressed in policies that are relevant to childhood obesity.

The annual report of 2016 on the Monitoring of the activities of the EU Platform for Action on Diet, Physical Activity and Health reports on commitments of the Platform which address reducing health inequalities or focused on lower socioeconomic groups in their objectives. The report recommends to further reinforce the theme of reducing health inequalities. [203]

The EU Health Programme has a main goal to in improve the health of Europeans and reduce inequalities by promoting health, encouraging innovation, boosting the sustainability of health systems and protecting Europeans from serious cross-border health threats. Joint Actions like Equity Action[204] and JANPA addressed health and socio-economic inequality, identifying tools such as Health Impact Assessments with an equity focus, Health Equity Audits and developing models of good practice with special attention to social inequality aspects. The mid-term evaluation of the EU Health Programme mentions inequalities, but doesn't really report on this. [205]

Comments/notes

[201] Public Health Evaluation and Impact Assessment Consortium (PHEIAC), 2013. Evaluation of the implementation of the Strategy for Europe on Nutrition, Overweight and Obesity related health issues FINAL REPORT. 29 April 2013. https://ec.europa.eu/health/sites/health/files/nutrition_physical_activity/docs/pheiac_nutrition_strategy_evaluation_en.pdf

[202] European Commission, 2017. REPORT FROM THE COMMISSION TO THE EUROPEAN PARLIAMENT, THE COUNCIL, THE EUROPEAN ECONOMIC AND SOCIAL COMMITTEE AND THE COMMITTEE OF THE REGIONS. Mid-term evaluation of the 3rd Health programme 2014-2020 under Regulation (EU) No 282/2014 on the establishment of a third programme of Union action in the field of health (2014-2020) {SWD(2017) 331 final} {SWD(2017) 333 final}. Brussels, 11.10.2017 COM(2017) 586 final. https://ec.europa.eu/health/funding/programme/2014-2020/midterm_evaluation_nl. https://ec.europa.eu/health/sites/health/files/programme/docs/2014-2020_evaluation_midtermreport_en.pdf

[203] European Commission, 2016. Monitoring the activities of the EU Platform for Action on Diet, Physical Activity and Health. Annual Report 2016. https://ec.europa.eu/health/sites/health/files/nutrition_physical_activity/docs/2016_report_en.pdf

[204] https://eurohealthnet.eu/hpe/equity-action-joint-action

[205] European Commission, 2017. REPORT FROM THE COMMISSION TO THE EUROPEAN PARLIAMENT, THE COUNCIL, THE EUROPEAN ECONOMIC AND SOCIAL COMMITTEE AND THE COMMITTEE OF THE REGIONS. Mid-term evaluation of the 3rd Health programme 2014-2020 under Regulation (EU) No 282/2014 on the establishment of a third programme of Union action in the field of health (2014-2020) {SWD(2017) 331 final} {SWD(2017) 333 final}. Brussels, 11.10.2017 COM(2017) 586 final. https://ec.europa.eu/health/funding/programme/2014-2020/midterm_evaluation_nl. https://ec.europa.eu/health/sites/health/files/programme/docs/2014-2020_evaluation_midtermreport_en.pdf

DOMAIN 11 - FUNDING AND RESOURCES - This domain concerns the extent to which the EU has sufficient funding invested in 'Population Nutrition Promotion' (estimated from the investments in population promotion of healthy eating and healthy food environments for the prevention of obesity and diet-related NCDs, excluding all one-on-one promotion (primary-care, antenatal services, maternal and child nursing services etc.), food safety, micronutrient deficiencies (e.g. folate fortification and undernutrition) to create healthy food environments, improved population nutrition, reductions in obesity, diet-related NCDs and their related inequalities.

FUND1 The 'population nutrition' budget, as a proportion of total health spending and/or in relation to the diet-related NCD burden sufficiently contributes to reducing diet-related NCD's.

Definitions and scope

- 'Population nutrition' includes promotion of healthy eating, and policies and programs that support healthy food environments for the prevention of obesity and diet-related NCDs
- The definition excludes all one-on-one and group-based promotion (primary care, antenatal services, maternal and child nursing services etc.), food safety, micronutrient deficiencies (e.g. folic acid fortification) and undernutrition
- Includes estimates for the budget allocated to health related DG's within the EU (e.g. DG Health and Food Safety (SANTE), that has primary responsibility for population nutrition.
- The workforce comprises anyone whose primary role relates to population nutrition and who is employed full time, part time or casually by the Department of Health or contracted by the Department of Health to perform a population nutrition-related role (including consultants or funding of a position in another government or nongovernment agency). The number of full time equivalent persons in the workforce will be reported in 'FUND4
- Excludes budget items related to physical activity promotion

FUND2 EU funded research is targeted for improving food environments, reducing obesity, NCDs and their related inequalities

Definitions and scope

- Includes the clear identification of research priorities related to improving food environments, reducing obesity, NCDs and their related inequalities in health or medical research strategies or frameworks
- Includes identifying research projects conducted or commissioned by the government specifically targeting food environments, prevention of obesity or NCDs (excluding secondary or tertiary prevention)
- It is limited to research projects committed to or conducted within the last 12 months
- Excludes research grants administered by the government (including statutory agencies) to a research group where the allocation of a pool of funding was determined by an independent review panel
- Excludes evaluation of interventions (this is explored in 'MONIT5' and should be part of an overall program budget)

FUND3 There is a statutory health promotion agency in place that includes an objective to improve population nutrition with a secure funding stream

Definitions and scope

- Agency was established through legislation
- Includes objective to improve population nutrition in relevant legislation, strategic plans or on agency website
- Secure funding stream involves the use of a hypothecated tax or other secure source

Policy evidence summary

The 'population nutrition' budget, as a proportion of total health spending and/or in relation to the diet-related NCD burden (FUND 1)

Funding for DG SANTE's activities is fixed within the EU's 2014-2020 Multiannual Financial Framework.[206] Expenditure related to 'Food and Feed' and 'Public Health' falls under the Heading 3: Security and Citizenship of the Multiannual Financial Framework[207]. The budget for activities linked 'Food and Feed' (animal and plant health programme) is 1891 million euros and for activities linked to 'Public Health' (EU Health Programme) 449 million euros.[208][209]
Expenditure of the Health Programme is directly managed by DG SANTE and the Consumer, Health and Food Executive Agency (CHAF-EA).[210] The Commission also receives additional funding from the European Parliament for pilot projects to improve future policy action in the area of nutrition and physical activity and to identify good practices. Under the Third Health Programme (2014-2020) the Commission has contributed (till 22.10.2019) a total of € 16.611.981,00 on different projects related to population nutrition and to the promotion of healthy diets (the amount is also including projects related to the promotion of energy-balance related lifestyles in general, e.g. physical activity).[211] Examples of projects are the Schools for Health in Europe network (SHE), Mapping Member States' fiscal measures and pricing policies applied to food, non-alcoholic drinks and alcoholic beverages and the feasibility study for a monitoring system on reformulation initiatives for salt, sugars and fat.

Funding for the Common Agricultural Policy (CAP) falls under Heading 2: Sustainable growth: natural resources of the Multiannual Financial Framework.[212] For the EU Fruit and Vegetable Scheme which falls into the first pillar of the CAP (direct payments and market measures, 312,735

---

[206] European Commission. Strategic Plan 2016-2020 DG Health and Food Safety. Ref. Ares(2017)6260978 - 20/12/2017. https://ec.europa.eu/info/sites/info/files/strategic-plan-2016-2020-dg-sante_en_0.pdf.

[207] Council of the European Union, 2017. ANNEX to the COMMUNICATION FROM THE COMMISSION TO THE COUNCIL AND THE EUROPEAN PARLIAMENT Technical adjustment of the financial framework for 2019 in line with movements in GNI (ESA 2010) (Article 6 of Council Regulation No 1311/2013 laying down the multiannual financial framework for the years 2014-2020) https://data.consilium.europa.eu/doc/document/ST-9147-2018-ADD-1/en/pdfhttps://ec.europa.eu/info/sites/info/files/about_the_european_commission/eu_budget/com_2017_220_annex_en.pdf

[208] European Commission. Strategic Plan 2016-2020 DG Health and Food Safety. Ref. Ares(2017)6260978 - 20/12/2017. https://ec.europa.eu/info/sites/info/files/strategic-plan-2016-2020-dg-sante_en_0.pdf. and https://ec.europa.eu/info/about-european-commission/eu-budget/spending/topic/eu-funding-programmes-2014-2020/heading-3-security-and-citizenship_en

[209] https://ec.europa.eu/info/about-european-commission/eu-budget/spending/topic/eu-funding-programmes-2014-2020/heading-3-security-and-citizenship_en

[210] European Commission. Strategic Plan 2016-2020 DG Health and Food Safety. Ref. Ares(2017)6260978 - 20/12/2017. https://ec.europa.eu/info/sites/info/files/strategic-plan-2016-2020-dg-sante_en_0.pdf.

[211] European Commission, 2019. Nutrition projects Health Programme. Excel overview, 22.10.2019.

[212] https://ec.europa.eu/info/about-european-commission/eu-budget/spending/topic/eu-funding-programmes-2014-2020/heading-2-sustainable-growth-natural-resources_en#common-agricultural-policy-pillar-i

million euros)[213] is a maximum of 150 million euros per school year available for requesting Member States, taking into account their number of six-to ten-year-old children and the degree of development of their regions.[214] All Member States except Sweden and the UK have received EU Fruit and Vegetable Scheme funds for the 2017-2018 and 2018-2019 school years. The European Public Health Alliance (EPHA) states in a report[215] that the School fruit and vegetable Scheme only represents 0.25% of the overall CAP budget. EPHA therefore recommends to gradually increase the budget for this scheme, in line with increased take-up, and enhance co-financing rates for schools in economically deprived areas where vegetable and fruit intake is especially low.

Next to the Public Health Programme, the EU provides other funds to promote investing in health as a broader means of achieving smart and inclusive growth. These funds are the European and Structural Investment Funds (ESIF)[216] and the European Fund for Strategic Investments (EFSI).[217] The health sector receives ESIF for investments through the European Regional Development Fund and the European Social Fund. For 2014-2020 more than 9 billion euros was foreseen in all Member States for health-related investments.[218] EFSI is a partnership of the Commission and the EIB, providing a financial guarantee to the value of 315 billion euros over a three year period and with an extension up to 500 billion euros until 2020.[219]

For the next Multiannual Financial Framework 2021-2027, the European Social Fund Plus Programme (ESF +) will be the main investment fund for investing in Health.[220] This programme merges existing funds like the Health Programme, The European Social Fund (ESF) and the Youth Employment Initiative (YEI), The Fund for European Aid to the Most Deprived (FEAD) and The Employment and Social Innovation (EaSI) programme. The ESF + Programme opens up potential for a stronger financial translation of health in all policies. For the Health strand an amount of 413 billion euros will be available. How much specifically is related to nutrition and promoting healthy diets is unknown in advance as there is no earmarking with such level of detail.

EU funded research targeted for improving food environments, reducing obesity, NCDs and their related inequalities (FUND 2)
The European Union's strategy 2020 for smart, sustainable and inclusive growth, sets the strengthening of research and innovation as one of its main objectives. The implementation of the Innovation Union (to create jobs and growth by improving conditions and access to finance for research and innovation)[221] including the European Research Area[222] are part of this Strategy. Horizon 2020 and the Joint Programming Initiative 'A Healthy Diet for a Healthy Life' are research programmes contributing to a fully operational European Research Area on the prevention of diet-related diseases '.

[213] https://ec.europa.eu/info/about-european-commission/eu-budget/spending/topic/eu-funding-programmes-2014-2020/heading-2-sustainable-growth-natural-resources_en#common-agricultural-policy-pillar-i
[214] European Union, 2019. Briefing: The EU Fruit and Vegetable Sector. EPRS | European Parliamentary Research Service Author: Rachele Rossi Members' Research Service PE 635.563 – March 2019. http://www.europarl.europa.eu/RegData/etudes/BRIE/2019/635563/EPRS_BRI(2019)635563_EN.pdf
[215] European Public Health Alliance (EPHA), 2016. A CAP for a Healthy Living. Mainstreaming Health into the EU Common Agricultural Policy. https://epha.org/wp-content/uploads/2016/03/A-CAP-for-Healthy-Living_EPHA_2016.pdf
[216] https://ec.europa.eu/health/funding/structural_funds_en
[217] https://ec.europa.eu/health/funding/investment_plan_en
[218] European Commission. HEALTH INVESTMENTS BY EUROPEAN STRUCTURAL AND INVESTMENT FUNDS (ESIF) 2014-2020 https://ec.europa.eu/health/sites/health/files/health_structural_funds/docs/esif_factsheet_en.pdf
[219] https://ec.europa.eu/health/funding/investment_plan_en
[220] https://ec.europa.eu/health/funding/future_health_budget_en
[221] https://www.mariecuriealumni.eu/newsletter/all-you-need-know-about-innovation-union-iu
[222] https://ec.europa.eu/info/research-and-innovation/strategy/era_en

Horizon 2020 invests in innovative solutions that help tackle the adverse effects on human health[223], with funding of nearly 80 billion euros available (2014-2020). [224] 'Food and Healthy Diet' is marked as an area of Horizon 2020, with issues as 'promoting informed consumer choices' and delivering strategic solutions for healthy and safe foods and diets for all'. [225] Example of projects which has received funding from Horizon 2020 are 'FOOD 2030' which addresses Nutrition for sustainable and healthy diets[226], STOP[227] and CO-CREATE[228] which address overweight and obesity in children and adolescents.

The Joint Programming Initiative 'A Healthy Diet for a Healthy Life' started in 2014 and is a voluntary partnership between Member States and Associated Countries of the European Union, to address societal challenges as Health and Nutrition. [229] This includes research to increase the understanding of health-impacting behavior with respect to making food choices, to create insight in how the environment influences this behavior and to raise consumer understanding of healthy foods and diets. One of the projects financed by this JPI is the 'Policy-Evaluation Network (PEN)- Public policies addressing health- related behaviours in Europe'.

The Consumers, Health, Agriculture and Food Executive Agency (Chafea) has reserved funding for monitoring activities and behavioural studies, like the database on the nutritional characteristics of food products, to help monitor whether food products have increasingly less (or increasingly more) salt, fat or sugars[230] and the study on the impact of marketing on children's behavior.[231]

Statutory health promotion agency (FUND 3)
At EU level there is a Steering Group on Health Promotion, Disease Prevention and Management of Non-Communicable Diseases, [232] which was set up in July 2018. [233] This Steering Group assists and advises the Commission on for example the coordination between Member States in addressing challenges caused by NCD's, the selection of best practices regarding health promotion, disease prevention and management of NCDs and monitoring progress towards reducing mortality due to NCD's. The Steering Committee is composed of one member per Member State and is chaired by DG SANTE. [234] Interested parties in the field can discuss with the Steering Group via the

---

[223] European Commission, 2016. COMMISSION STAFF WORKING DOCUMENT Key European action supporting the 2030 Agenda and the Sustainable Development Goals Accompanying the document Communication from the Commission to the European Parliament, the Council, the European Economic and Social Committee and the Committee of the Regions Next steps for a sustainable European future: European Union action for sustainability. Strasbourg, 22.11.2016 SWD(2016) 390 final. EUR-Lex: https://eur-lex.europa.eu/legal-content/EN/TXT/PDF/?uri=CELEX:52016SC0390&from=en
[224] https://ec.europa.eu/programmes/horizon2020/en/what-horizon-2020
[225] https://ec.europa.eu/programmes/horizon2020/en/area/food-healthy-diet
[226] https://ec/europa.eu/research/bioeconomy/index.cfm?pg=policy&lib=food2030
[227] http://www.stopchildobesity.eu/what-is-stop/
[228] https://www.fhi.no/en/studies/co-create/
[229] https://www.healthydietforhealthylife.eu/index.php/about/vision

[230] https://etendering.ted.europa.eu/cft/cft-display.html?cftId=3068.
https://ec.europa.eu/health/sites/health/files/nutrition_physical_activity/docs/2019_initiatives_npa_en.pdf
[231] https://ec.europa.eu/info/publications/study-impact-marketing-through-social-media-online-games-and-mobile-applications-childrens-behaviour_en
[232] http://ec.europa.eu/transparency/regexpert/index.cfm?do=groupDetail.groupDetail&groupID=3622

[233] COMMISSION DECISION of 17.7.2018 setting up a Commission expert group "Steering Group on Health Promotion, Disease Prevention and Management of Non-Communicable Diseases" and repealing the Decision setting up a Commission expert group on rare diseases and the Decision establishing a Commission expert group on Cancer Control. C(2018) 4492 final. EUR-Lex: https://eur-lex.europa.eu/legal-content/EN/TXT/?uri=CELEX%3A32018D0718%2802%29

[234] COMMISSION DECISION of 17.7.2018 setting up a Commission expert group "Steering Group on Health Promotion, Disease Prevention and Management of Non-Communicable Diseases" and repealing the Decision setting up a Commission expert group on rare diseases and the Decision establishing a Commission expert group on Cancer Control. C(2018) 4492 final. EUR-Lex : https://eur-lex.europa.eu/legal-content/EN/TXT/?uri=CELEX%3A32018D0718%2802%29

Health Policy Platform.[235] The Steering Group has been set up by a Commission Decision.[236] The costs of running the Steering group (secretariat, travel expenses) are born by the administrative budget of the Commission. The support to projects in areas identified as priority by the group can originate from – and follow the rules of – any of the financial support envelopes of the Commission.

Comments/notes

DOMAIN 12 - PLATFORMS AND INTERACTION: This domain concerns the extent to which there are coordination platforms and opportunities for synergies across EU departments, levels of government, and other sectors (NGOs, private sector, and academia) such that policies and actions in food and nutrition are coherent, efficient and effective in improving food environments, population nutrition, diet-related NCDs and their related inequalities.

PLAT1 There are robust coordination mechanisms across departments and levels of government (European, national, state and local) to ensure policy coherence, alignment, and integration of food, obesity and diet-related NCD prevention policies across governments.

Definitions and scope

- Includes cross-government or cross-departmental governance structures, committees or working groups (at multiple levels of seniority), agreements, memoranda of understanding, etc.
- Includes cross-government or cross-departmental shared priorities, targets or objectives
- Includes strategic plans or frameworks that map the integration and alignment of multiple policies or programs across governments and across departments
- Includes cross-government or cross-departmental collaborative planning, implementation or reporting processes, consultation processes for the development of new policy or review of existing policy

PLAT2 There are formal platforms (with clearly defined mandates, roles and structures) for regular interactions between the EU and the commercial food sector on the implementation of healthy food policies and other related strategies.

Definitions and scope

- The commercial food sector includes food production, food technology, manufacturing and processing, marketing, distribution, retail and food service, etc. For the purpose of this indicator, this extends to commercial non-food sectors (e.g. advertising and media, sports organisations, land/housing developers, private childcare, education and training institutes) that are indirectly related to food

[235] https://ec.europa.eu/health/non_communicable_diseases/steeringgroup_promotionprevention_en

[236] European Commission, 2018. COMMISSION DECISION of 17.7.2018 setting up a Commission expert group "Steering Group on Health Promotion, Disease Prevention and Management of Non-Communicable Diseases" and repealing the Decision setting up a Commission expert group on rare diseases and the Decision establishing a Commission expert group on Cancer Control. Brussels, 17.7.2018 C(2018) 4492 final. https://ec.europa.eu/health/sites/health/files/major_chronic_diseases/docs/c2018_4492_en.pdf

- Includes established groups, forums or committees active within the last 12 months for the purpose of information sharing, collaboration, seeking advice on healthy food policies
- Includes platforms to support, manage or monitor private sector pledges, commitment or agreements
- Includes platforms for open consultation
- Includes platforms for the government to provide resources or expert support to the commercial food sector to implement policy
- Excludes joint partnerships on projects or co-funding schemes
- Excludes platforms to engage with industry in relation to development of policies.
- Excludes initiatives covered by 'RETAIL3' and 'RETAIL4'

PLAT3 There are formal platforms (with clearly defined mandates, roles and structures) for regular interactions between the EU and civil society on the development, implementation and evaluation of healthy food policies and other related strategies.

Definitions and scope

- Civil society includes community groups and consumer representatives, NGOs, academia, professional associations, etc.
- Includes established groups, forums or committees active within the last 12 months for the purpose of information sharing, collaboration, seeking advice
- Includes platforms for consultation on proposed plans, policy or public inquiries
- Excludes policies or procedures that guide consultation in the development of food policy (see 'GOVER3')

PLAT4 The governments work with a system-based approach with (local, national and European) organisations/partners/groups to improve the healthiness of food environments in EU countries

Definitions and scope

- Systems-based approaches may include policies within other domains of health
- May include a social-determinants of health approach
- May bring together multiple departments or ministries to approach health
- Includes multiple levels of government
- Aim of a systems-based approach is:
    - resourcing and supporting a dedicated, reflective and skilled workforce at a EU, state and/or local level to engage, activate and influence at multiple levels of the system to combat obesity and chronic disease
    - building relationships with prevention partners across the system, and across sectors and industries, to strengthen positive health outcomes on multiple fronts
    - capturing and feeding back knowledge and data on progress, impact and effectiveness and calling for new types of research, policy and practice collaborations
    - allocating resources based on best possible investment to effect change and population need, seeding long term change by resourcing local governments to lead action towards public health

- building leadership for sustained prevention across the system to drive effective and long lasting change

Policy evidence summary

Article 168 of the Treaty on the Functioning of the European Union includes an integration clause requiring that human health protection is ensured in all EU policies and activities. [237] The European Union shall encourage cooperation between the Member States, but the Member States themselves shall coordinate their policies and programmes in the area of improving public health and preventing physical and mental illness and diseases.

Coordination mechanisms to ensure policy coherence, alignment and integration of food, obesity, and diet-related NCD prevention policies (PLAT 1)

Nevertheless there are mechanisms at EU level, which aim to ensure cooperation and coordination at the level of the European Commission, the European Parliament, the Council, between the EU and the Member States, between Member States, and with subnational governments and social partners.

Coordination at the European Commission

At the commission level there are rules of procedures for the cooperation and coordination between departments. [238] These rules prescribe that the Secretary-General ensures that the necessary coordination between departments in the preparatory stages takes place. Before submitting a document to the Commission, the responsible department shall consult other departments which are associated or concerned by virtue of their powers or responsibilities or by nature of the subject. In case, this did not happen, the responsible department shall inform the Secretary General.

Legislative and other proposal documents should be introduced by a consultative document or roadmap followed by a public consultation and a Commission impact assessment focusing on economic, environmental and social aspects (including a voluntary impact assessment on public health). [239][240][241] Any important proposal needs to pass the Regulatory Scrutiny board, composed of three high-level Commission officials and three experts recruited from outside the Commission, before it can be agreed internally.[242]

[237] European Union, 2012. Consolidated version of the Treaty on the Functioning of the European Union. OJ C 326, pp. 47-390. 26. 10.2012. EUR-Lex: https://eur-lex.europa.eu/legal-content/EN/TXT/PDF/?uri=CELEX:12012E/TXT&from=EN

[238] European Commission, 2000. RULES OF PROCEDURE OF THE COMMISSION (C(2000) 3614). OJ L 308. 8.12.2000, pp. 26-34. EUR-Lex: https://eur-lex.europa.eu/legal-content/EN/TXT/PDF/?uri=CELEX:32000Q3614&from=EN

[239]Scott L. Greer, Nick Fahy, Heather A. Elliott, Matthias Wismar, Holly Jarman, Willy Palm, World Health Organization 2014, European Observatory on Health Systems and Policies. Everything you always wanted to know about European Health policies but were afraid to ask. United Kingdom: London. And: European Commission. 2013 roadmaps. Luxembourg, Publications Office of the European Union, 2013. http://ec.europa.eu/governance/impact/planned_ia/roadmaps_2013_en.htm#SANCO.

[240] European Commission, 2017. COMMISSION STAFF WORKING DOCUMENT Better Regulation Guidelines. Chapter III Guidelines on impact assessment. Brussels, 7 July 2017 SWD (2017) 350 http://www.emcdda.europa.eu/system/files/attachments/7906/better-regulation-guidelines.pdf

[241] https://ec.europa.eu/info/law/law-making-process/evaluating-and-improving-existing-laws/refit-making-eu-law-simpler-and-less-costly_en

[242] https://ec.europa.eu/info/law/law-making-process/regulatory-scrutiny-board_en

Coordination at the European Parliament and Council

Legislative proposals prepared by the Commission have to be adopted by the European Parliament and the Council of Ministers. [243]

Members of the European Parliament are divided up among a number of specialized standing committees, to do preparatory work for the Parliament's plenary sittings. [244] The lead committee for health is the Environment, Public Health and Food Safety Committee, but other committees also play a significant role in relation to health such as the Employment and Social Affairs Committee or the Industry, Research and Energy Committee. [245] When a member of the European Parliament within a certain committee prepares a report, this report is then considered and revised by the committee as a whole, and then by parliament as a whole in one of the monthly plenary sessions. Furthermore, if several committees have interest in a file, they have an opportunity to be consulted and put forward amendments for their areas of responsibility. Where disagreements remain, these can be taken to the full plenary session of parliament. [246]

The Council of Ministers is made up of the relevant ministers from one Member State. [247] There is a Council for Employment, Social Policy , health and Consumers Affairs and a Council for Agriculture and Fisheries. [248] In practice these councils are made up of different representatives. Coordination in the Council is in the hands of the Council Presidency (agenda setting, chairing and brokering compromises), a role which is shared among the EU countries. The General Affairs Council also has a coordination role and is responsible for institutional, administrative and horizontal matters. [249] However, this approach does not allow a Council with one thematic focus, to comment of engage with decisions taken by another Council. Therefore this relies on effective coordination of Member States at national level to ensure that positions expressed in one Council take account of the full range of views domestically and represent an integrated opinion. [250]

The European Council, is made up of the heads of state and government of the Member States, which cannot adopt legislation, but sets the overall direction of the EU and brokering solutions to its most intractable problems. [251]

The Council is supported by the Committee of Permanent Representatives of the governments of the Member States to the European Union (Coreper) and more than 10 specialized working parties and committees. [252] The Working Party on Public Health is such a preparatory body. There is also a Working Party on Public Health at Senior Level (top officials) which aim is to better connect the EU agenda with the Member States's agenda's and preferences. [253]

Other treaty bodies

[243]Scott L. Greer, Nick Fahy, Heather A. Elliott, Matthias Wismar, Holly Jarman, Willy Palm, World Health Organization 2014, European Observatory on Health Systems and Policies. Everything you always wanted to know about European Health policies but were afraid to ask. United Kingdom: London.

[244] http://www.europarl.europa.eu/about-parliament/en/organisation-and-rules/organisation/committees

[245]Scott L. Greer, Nick Fahy, Heather A. Elliott, Matthias Wismar, Holly Jarman, Willy Palm, World Health Organization 2014, European Observatory on Health Systems and Policies. Everything you always wanted to know about European Health policies but were afraid to ask. United Kingdom: London.

[246] Idem.

[247] Idem.

[248] https://www.consilium.europa.eu/en/council-eu/configurations/

[249] Idem.

[250] Scott L. Greer, Nick Fahy, Heather A. Elliott, Matthias Wismar, Holly Jarman, Willy Palm, World Health Organization 2014, European Observatory on Health Systems and Policies. Everything you always wanted to know about European Health policies but were afraid to ask. United Kingdom: London.

[251] Idem.

[252] Idem.

[253] Idem.

Other treaty bodies which may or have to be consulted during the legislative process are the Economic and Social Committee which represents social partners (Employers and workers) and the Committee of the Regions which agglomerates the opinions of subnational governments. [254]

High Level group on Nutrition and Physical Activity

The High Level group on Nutrition and Physical Activity led by the European Commission and composed of EU government representatives has a coordination role in the sense that it enables governments to share policy ideas and best practices and enhances contact between governments and the EU Platform for action on diet, physical activity and health. [255] The High level Group did not meet since October 2018.

Open Method of Coordination

The Open Method of Coordination is an EU policy making process or regulatory instrument initiated by the Lisbon European Council in 2000. [256][257] The OMC does not result in EU legislation but is a method of soft governance which aims to spread best practice and achieve convergence towards EU goals in those policy areas which fall under the partial or full competence of Member States. OMC has been mostly applied in the social policy area and not widely used in the health area. There is also some debate about the impact of the OMC process. [258][259]

Platforms on the implementation of healthy food policies and other related strategies (PLAT 2 and PLAT 3)

The EU Platform for action on diet, physical activity and health

The EU Platform for action on diet, physical activity and health is a forum for European-level organisations which was founded in 2005 and includes food business operators, consumer organisations, public health NGO's and scientific and professional associations. [260] The Platform receives guidance from the High Level Group on Nutrition and Physical Activity and holds joint meetings with the High Level Group. Platform members share their action plans with each other. [261] All actions of the platform are available in the Platform database. [262] On 3 July 2019, seven civil society organisations (BEUC, EHN, EPHA, CPME, COFACE, World Obesity, ERWCPT and IBFAN) announced their resignation because they did not find the Platform fit for purpose. [263] The Commission announced an internal review of the Platform based on an external assessment of the Platform activities (due in December 2019). The Platform did not meet since October 2018.

The EU Pledge

[254] Scott L. Greer, Nick Fahy, Heather A. Elliott, Matthias Wismar, Holly Jarman, Willy Palm, World Health Organization 2014, European Observatory on Health Systems and Policies. Everything you always wanted to know about European Health policies but were afraid to ask. United Kingdom: London.

[255] https://ec.europa.eu/health/nutrition_physical_activity/high_level_group_en

[256] Scott L. Greer, Nick Fahy, Heather A. Elliott, Matthias Wismar, Holly Jarman, Willy Palm, World Health Organization 2014, European Observatory on Health Systems and Policies. Everything you always wanted to know about European Health policies but were afraid to ask. United Kingdom: London.

[257] European Parliament, 2014. The Open Method of Coordination. At a glance, October 2014. http://www.europarl.europa.eu/EPRS/EPRS-AaG-542142-Open-Method-of-Coordination-FINAL.pdf

[258] Scott L. Greer, Nick Fahy, Heather A. Elliott, Matthias Wismar, Holly Jarman, Willy Palm, World Health Organization 2014, European Observatory on Health Systems and Policies. Everything you always wanted to know about European Health policies but were afraid to ask. United Kingdom: London.

[259] European Parliament, 2014. The Open Method of Coordination. At a glance, October 2014. http://www.europarl.europa.eu/EPRS/EPRS-AaG-542142-Open-Method-of-Coordination-FINAL.pdf

[260] https://ec.europa.eu/health/nutrition_physical_activity/platform_en
https://ec.europa.eu/health/nutrition_physical_activity/events_en#anchor0

[261] https://ec.europa.eu/health/nutrition_physical_activity/platform_nl

[262] https://ec.europa.eu/health/nutrition_physical_activity/platform/platform_db_en

[263] https://www.foodnavigator.com/Article/2019/07/03/BEUC-EPHA-and-others-leave-EU-Diet-Platform-claiming-structure-not-fit-for-purpose and https://www.babymilkaction.org/archives/22161

The EU Pledge was launched in 2007 as part of a commitment to the Platform for action on diet, physical activity and health.[264] The EU Pledge is a voluntary initiative by food and beverage companies to change advertising to children under 12 years in the European Union.

European Health Policy Platform
The European Health Policy Platform is a collaborative initiative under the 3rd Health Programme to ease communication among health stakeholders and with the European Commission.[265] DG SANTE act's as the secretary of the Platform.

European Economic and Social Committee
The EESC enables civil society organisations (350 members) from the Member States to express their views at European level.[266] It's opinions are addressed to the European Commission, Parliament and Council. Consultation of the EESC is mandatory for public health policies (Article 168 of the TFEU).[267] The EESC may also adopt opinions on its own initiative.[268]

System-based approach to improve the healthiness of food environments in EU countries (PLAT 4)
We have found some evidence that the EU is aiming towards a system-based approach to improve the healthiness of food environments in EU countries.

In 2016, JRC published a foresight study 'Delivering on EU food safety and nutrition in 2050 - future challenges and policy preparedness'[269] which aims to aid policy makers in their assessment of the resilience of the current food policy and regulatory framework with a time horizon to 2050, contributing to ensuring that EU citizens continue to enjoy high standards of safe, nutritious and affordable food. The REFIT of the General Food Law was led by JRC employees involved in the foresight study. JRC and DG Health and Food Safety published also a viewpoint around win-wins and trade-offs building on the foresight study.[270] The publication states that future food policies need to be more sensitive to impacts on food safety and nutrition and health aspects. '*A holistic food systems approach must be taken to identify and discuss in advance possible tensions and trade-offs and to address them upfront in a systematic and transparent manner.*'

Earlier in 2014, JRC conducted a foresight study around research priorities for foods and diets towards a healthy society using a systems-based approach.[271] The study aimed at informing DG Research and Innovation for the development of research calls during HORIZON 2020. This has for example led to the development of a multidisciplinary research program SUSFANs (Metric, Models and Foresight for European Sustainable Food and Nutrition Security)[272], which overall objective is to build the conceptual framework, the evidence base and analytical tools for

264 https://eu-pledge.eu/about-the-eu-pledge/
265 https://webgate.ec.europa.eu/hpf/
266 https://www.eesc.europa.eu/en/about
267 European Union, 2012. Consolidated version of the Treaty on the Functioning of the European Union. OJ C 326, pp. 47-390. 26. 10.2012. https://eur-lex.europa.eu/legal-content/EN/TXT/PDF/?uri=CELEX:12012E/TXT&from=EN
268 Eric Davies, Cardiff University, 2013. European Economic and Social Committee. Information Guide. A guide to information sources on the European Economic and Social Committee (EESC), with hyperlinks to further sources of information within European Sources Online and on external websites http://aei.pitt.edu/75338/1/European_Economic_Social_Committee.pdf
269 Mylona, K., Maragkoudakis, P., Bock, A.-K., Wollgast, J., Caldeira, S. and Ulberth, F., Delivering on EU Food Safety and Nutrition in 2050 – Future challenges and policy preparedness, EUR27957 EN, Publications Office of the European Union, Luxembourg, 2016, ISBN 978-92-79-58916-4, doi:10.2787/625130. https://ec.europa.eu/jrc/en/publication/eur-scientific-and-technical-research-reports/delivering-eu-food-safety-and-nutrition-2050-future-challenges-and-policy-preparedness
270 Mylona K, Maragkoudakisa P, Mikob L, Bocka AK, Wollgast J, Caldeiraa S, Ulbertha F. (2018) Viewpoint: Future of food safety and nutrition - Seeking win-wins, coping with trade-offs, Food Policy 74 (2018) 143–146.
271 Bock AK, Maragkoudakis P, Wollgast J, Caldeira S, Czimbalmos A, Rzychon M, Atzel B, Ulberth F, 2014. JRC FORESIGHT STUDY Tomorrow's Healthy Society Research Priorities for Foods and Diets. https://ec.europa.eu/jrc/en/publication/eur-scientific-and-technical-research-reports/tomorrows-healthy-society-research-priorities-foods-and-diets
272 https://www.susfans.eu/

underpinning EU-wide food policies with respect to their impact on consumer diet and their implications for nutrition and public health in the EU, the environment, the competitiveness of the EU agri-food sectors, and global food and nutrition security.

The current work programme 2019-2020 of JRC is including projects using food system approaches, including work towards integrating assessments of food production, food waste and nutrition and health (Project 9500 – INTEGRATE).[273]

Furthermore, the political guidelines for the new Commission introduced the "Farm to Fork Strategy" on sustainable food [274]. If the new college is confirmed, one Commission Vice-President has 'foresight' in his title[275].

The FAO published in the frame of the Strategic Partnership with the Directorate for International Cooperation and Development of the European Commission a guideline (Food Systems for healthy Diets) to support the use of a comprehensive food system approach for policy makers in countries. [276]

However, a lot of different organisations still have recommended a system-based approach for the EU, like the EESC (opinion on civil society's contribution to the development of a comprehensive food policy in the EU) [277], the WHO (Connecting Food Systems for co-benefits Policy Brief) [278] and IPES (Towards a Common Food Policy for the European Union report). [279]

Comments/notes

---

[273] JRC 2019-2020 Detailed Work Plan. https://ec.europa.eu/jrc/sites/jrcsh/files/adopted_jrc_2019-20_wp_europa_v2.pdf

[274] By candidate for President of the European Commission Ursula von der Leyen. A Union that strives for more My agenda for Europe. Political Guidelines for the next European Commission 2019-2024. https://ec.europa.eu/commission/sites/beta-political/files/political-guidelines-next-commission_en.pdf

[275] Von der Leyen, U, President elect oft he European Commission, 2019. Mission Letter, Brussels 10 September 2019. https://ec.europa.eu/commission/sites/beta-political/files/mission-letter-maros-sefcovic-2019_en.pdf

[276] FAO, 2018. Food Systems for healthy diets. STRENGTHENING SECTOR POLICIES FOR BETTER FOOD SECURITY AND NUTRITION RESULTS. http://www.fao.org/3/CA2797EN/ca2797en.pdf

[277] Schmidt, P, European Economic and Social Committee (EESC), 2019. Promoting healthy and sustainable diets in the EU (own-initiative opinion). Reference: NAT/755-EESC-2018-04568. Adopted on 20/02/2019 - Bureau decision date: 12/07/201. https://www.eesc.europa.eu/en/our-work/opinions-information-reports/opinions/promoting-healthy-and-sustainable-diets-eu-own-initiative-opinion

[278] WHO Regional Office for Europe, 2018. Hawkes, Parsons. Policy Brief 31: Connecting food systems for co-benefits: How can food systems combine diet-related health with environmental and economic policy goals? http://www.euro.who.int/__data/assets/pdf_file/0007/387070/policy-brief-31-austria-eng.pdf?ua=1

[279] IPES Food Panel, 2019. TOWARDS A COMMON FOOD POLICY FOR THE EUROPEAN UNION THE POLICY REFORM AND REALIGNMENT THAT IS REQUIRED TO BUILD SUSTAINABLE FOOD SYSTEMS IN EUROPE. http://www.ipes-food.org/_img/upload/files/CFP_FullReport.pdf

DOMAIN 13 - HEALTH IN ALL POLICIES - This domain concerns the processes that are in place to ensure policy coherence and alignment, and that population health impacts are explicitly considered in the development of EU policies.

HIAP1 There are processes in place to ensure that population nutrition, health outcomes and reducing health inequalities or health impacts in vulnerable populations are considered and prioritised in the development of all EU policies relating to food

Definitions and scope

- Includes policies, procedures, guidelines, tools and other resources that guide the consideration and assessment of nutrition, health outcomes and reducing health inequalities or health impacts in vulnerable populations prior to, during and following implementation of food-related policies.
- Includes the establishment of cross-department governance and coordination structures while developing food-related policies.

HIAP2 There are processes e.g. Health Impact Assessment's (HIAs) to assess and consider health impacts during the development of other non-food policies.

Definitions and scope

- Includes a government-wide HiAP strategy or plan with clear actions for non-health sectors.
- Includes policies, guidelines, tools and other resources that guide the consideration and assessment of health impacts prior to, during and following implementation of non-food related policies (e.g. HIAs or health lens analysis).
- Includes the establishment of cross-department or cross-sector governance and coordination structures to implement a HiAP approach.
- Includes workforce training and other capacity building activities in healthy public policy for non-health departments (e.g. agriculture, education, communications, trade).
- Includes monitoring or reporting requirements related to health impacts for non-health departments.

Policy evidence summary

Health in All Policies at the EU (HIAP 1 and HIAP 2)

The importance of the 'Health in all Policies' principle is highlighted in several EU policy documents and objectives/tasks of EU institutions.

Article 168 of the Treaty on the Functioning of the European Union incorporates health in all policies stating 'A high level of human health protection shall be ensured in the definition and implementation of all Union policies and activities'.[280]

[280] European Union, 2012. Consolidated version of the Treaty on the Functioning of the European Union. OJ C 326, 26.10.2012, p. 47–390. EUR-Lex: https://eur-lex.europa.eu/legal-content/EN/TXT/?uri=celex%3A12012E%2FTXT.

The Regulation (EU) No 282/2014 of the European Parliament and the Council on the establishment of the third Health Programme (2014-2020)[281] also refers to health in all policies in the Annex about Thematic Priorities: 'Promote health, prevent diseases and foster supportive environments for healthy lifestyles taking into account the ' health in all policies' principle'. This principle is also guiding in one of the objectives of the European Health Policy Platform, which is an initiative under the 3rd Health Programme, i.e. 'provide information on other policy areas related to health following the 'Health in All Policies' approach.[282]

Furthermore, supporting Health in All Policies is an assigned task of the High Level Group on Nutrition and Physical Activity.[283] Health in All Policies is also mentioned in the 'Initiatives on Nutrition and Physical Activity' document of DG Health and Food Safety.[284] Examples mentioned are DG SANTE collecting best practices and providing input to the discussions of the Fruit, Vegetables and Milk Scheme and the modernisation of the Common Agriculture Policy.

However, other policy documents like the Europe 2020 Strategy do not mention anything about Health in All Policies.[285] Also in practice, the health in all policies principle is not really implemented. Examples here are the Impact Assessments, where the Health Impact Assessment part remains voluntary. Every Directorate-General starts a political validation process to decide if an Impact Assessment is required for a certain proposal. When an Impact Assessment starts, DG's have the freedom to choose which impacts to describe, although the quality of each report is checked by an independent Regulatory Scrutiny Board. For the Strategic Environmental Assessments, health is included,[286] but SEA's are only applied to plans and programmes and not to policies.

In 2018, the European Public Health Alliance (EPHA) and several other health organisations made a joint statement to call on the European Union to do more for health.[287] They stated that population health is a precondition for economic prosperity. One of the key asks they made included a call on the EU to develop and routinely deploy a robust Health In All methodology to respect article 168 of the Treaty on the Functioning of the EU.

Impact Assessment of the CAP (HIAP 1)

The Impact Assessment of the CAP (report of June 2018), which accompanied the legislative proposals for the CAP Post 2020 in the context of the next Multi Annual Financial Framework (MFF), refers several times to health.[288] The Assessment states, for example, that 'a strong CAP is needed to address societal expectations on food and health' and that 'the CAP is expected to respond better to consumer demands on food and health'. One workshop to collect evidence for the impact assessment had the theme 'Food and related issues' which confirmed that the CAP can help in providing a mix of interventions which influence food consumption because the CAP is well aligned with food safety requirements and already includes schemes that promote healthy diets. The workshop further stated that 'the governance of food systems requires a coordinated approach across policy domains'.

---

[281] REGULATION (EU) No 282/2014 OF THE EUROPEAN PARLIAMENT AND OF THE COUNCIL of 11 March 2014 on the establishment of a third Programme for the Union's action in the field of health (2014-2020) and repealing Decision No 1350/2007/EC. OJ L 86, 21.3.2014. pp. 1-13. EUR-Lex: https://eur-lex.europa.eu/legal-content/EN/TXT/PDF/?uri=CELEX:32014R0282

[282] https://webgate.ec.europa.eu/hpf/

[283] https://ec.europa.eu/health/nutrition_physical_activity/high_level_group_en

[284] European Commission, 2019. Initiatives on nutrition and physical activity. https://ec.europa.eu/health/sites/health/files/nutrition_physical_activity/docs/2019_initiatives_npa_en.pdf

[285] European Commission, 2010. COMMUNICATION FROM THE COMMISSION EUROPE 2020 A strategy for smart, sustainable and inclusive growth. Brussels, 3.3.2010 COM(2010) 2020. https://ec.europa.eu/eu2020/pdf/COMPLET%20EN%20BARROSO%20%20%20007%20-%20Europe%202020%20-%20EN%20version.pdf

[286] https://ec.europa.eu/environment/eia/sea-legalcontext.htm

[287] EPHA, et.al, 2018. Joint Statement Europe let's do more for health. Our vision for health in the European Union. https://epha.org/wp-content/uploads/2018/04/EU4health-joint-statement.pdf

[288] European Commission, 2018. COMMISSION STAFF WORKING DOCUMENT IMPACT ASSESSMENT. Brussels,1.6.2018 SWD(2018) 301 final. EUR-Lex: https://eur-lex.europa.eu/legal-content/EN/TXT/?uri=SWD%3A2018%3A301%3AFIN

Nevertheless health organisations also have their remarks on this IA, saying that ‘’the assessment however is not built on a systematic exposition of the main links between agriculture and public health.’[289]
**Comments/notes**

[289] EPHA, 2018. A healthy future needs good impact assessments, today. https://epha.org/a-healthy-future-needs-good-impact-assessments-today/

ANNEX 4A
THE FOOD- ENVIRONMENT POLICY INDEX (FOOD-EPI):IRELAND

Table of Contents

Introduction 4
.... 6
Instructions for rating 7
Introduction to the socio economic inequalities aspect of Food-EPI 9
Definitions 10
Abbreviations 12
Healthy Food Environment Policy Index: Policy domains 14
DOMAIN 1 - FOOD COMPOSITION 14
COMP1 14
COMP2 21
DOMAIN 2 - FOOD LABELLING 24
LABEL1 24
LABEL2 24
LABEL3 25
LABEL4 30
DOMAIN 3 - FOOD PROMOTION: 36
PROMO1 36
PROMO2 40
PROMO3 44
PROMO4 48
PROMO5 51
DOMAIN 4 - FOOD PRICES 52
PRICES1 52
PRICES2 54
PRICES3 60
PRICES4 64
DOMAIN 5 - FOOD PROVISON 67
PROV1 67
PROV2 77

PROV3 ... 83
PROV4 ... 85
PROV5 ... 90
DOMAIN 6 - FOOD IN RETAIL ... 94
RETAIL1 ... 94
RETAIL2 ... 97
RETAIL3 ... 99
RETAIL4 ... 101
Healthy Food Environment Policy Index: Infrastructure domains ... 103
DOMAIN 8 – LEADERSHIP ... 103
LEAD1 ... 103
LEAD2 ... 110
LEAD3 ... 113
LEAD4 ... 118
LEAD5 ... 122
DOMAIN 9 - GOVERNANCE ... 128
GOVER1 ... 128
GOVER2 ... 132
GOVER3 ... 134
GOVER4 ... 137
DOMAIN 10 - MONITORING AND INTELLIGENCE ... 140
MONIT1 ... 140
MONIT2 ... 145
MONIT3 ... 148
MONIT4 ... 152
MONIT5 ... 153
MONIT6 ... 156
DOMAIN 11 - FUNDING AND RESOURCES: ... 158
FUND1 ... 158
FUND2 ... 161

FUND3 ... 164
DOMAIN 12 - PLATFORMS FOR INTERACTION ... 166
PLAT1 ... 166
PLAT2 ... 169
PLAT3 ... 172
PLAT4 ... 174
DOMAIN 13 - HEALTH IN ALL POLICIES ... 177
HIAP1 ... 177
HIAP2 ... 179
References ... 181

Introduction

The World Health Organization (WHO) predicts that Ireland will be one of Europe's most overweight countries by 2030 (Breda et al., 2015, Healthy Ireland, 2016a). With one out of four children and two out of three adults carrying excess weight (Layte and McCrory, 2011), obesity is at an unacceptably high level (Keane et al., 2014). The direct and indirect costs associated with adult overweight and obesity are estimated at €1.13 billion per year (Dee et al., 2015), while the annual direct healthcare costs amongst children attributable to childhood overweight and obesity are estimated at €1.7 million (Ivan J. Perry, 2017).

Effective government policies are essential to increase the healthiness of food environments and to reduce obesity, NCDs, and their related inequalities (INFORMAS, 2019). Food environments are defined as the collective physical, economic, policy and socio-cultural surroundings, opportunities and conditions that influence people's food and beverage choices and nutritional status. Unhealthy food environments lead to unhealthy diets and excess energy intake, which have consequences on levels of morbidity and mortality. It is critical that Governments implement preventive policies and actions to match the magnitude of the burden that unhealthy diets are creating (INFORMAS, 2019).

The Food Environment Policy Index (Food-EPI) has been developed by the International Network for Food and Obesity/NCDs Research, Monitoring and Action Support (INFORMAS) and assesses a government's level of implementation of policies and infrastructure support against a set of good practice statements (Vandevijvere and Swinburn, 2015). The Food-EPI aims to answer the overarching question: how much progress have governments made towards good practice in improving food environments and implementing obesity and non-communicable disease (NCD) prevention policies and actions? Thus the goal of the Food EPI Ireland is;

1. To assess and compare the extent of implementation of national government policies and actions in Ireland, for creating healthy food environments against international best practice, and to identify the major implementation gaps.
2. To propose concrete actions to close the implementation gaps identified.
3. To prioritize actions needed to address critical gaps in government policies and infrastructure support for implementation according to their importance and achievability.

Globally, it has already been applied in over twenty countries and will now be used to assess the level of implementation of policies within European countries such as; Ireland, the Netherlands, Poland, Germany and Norway. Furthermore, the role of EU policies will also be taken into account within this approach.

The Food-EPI index consists of two components (Policies and Infrastructure Support), 13 domains and 47 good practice indicators. The policy component includes seven domains to address the key aspects of food environments that can be influenced by governments to create readily accessible, available and affordable healthy food choices. The infrastructure support component includes six domains that facilitate policy development and implementation to prevent obesity and NCDs. Good practice statements are proposed within each domain, that describe the 'best practices' (policies and infrastructure support) that governments put in place to contribute towards a healthier food environment (see figure 1 below).

Researchers based at the School of Public Health in University College Cork have compiled the Food- EPI Evidence Paper for Ireland. The researchers have highlighted if there is new evidence of implementation since 2016, and if the benchmark has substantially improved since then.

FIGURE 1 FOOD ENVIRONMENT POLICY INDEX (FOOD-EPI)

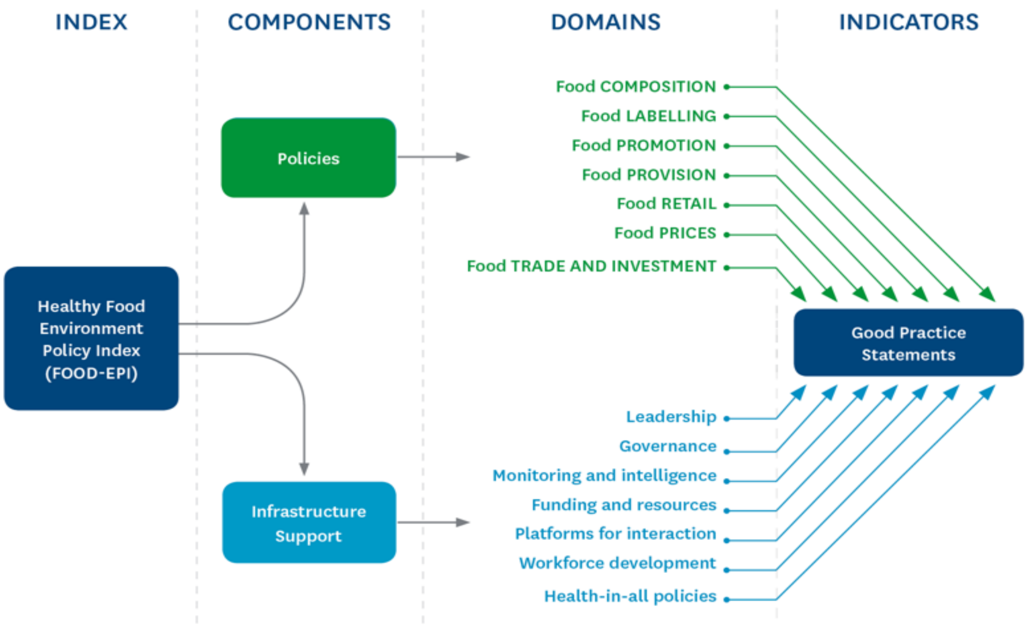

Instructions for rating

You are invited to participate in the Irish Food-EPI 2020 expert panel. This will involve rating the current level of Government action on policies impacting on the Irish food environment for 47 good practice indicators, against international best practice, on a Likert scale from 1 to 5.

The meaning of the Likert scale is:

1: <20% implemented compared to international best practice
2: 20-40% implemented compared to international best practice
3: 40-60% implemented compared to international best practice
4: 60-80% implemented compared to international best practice
5: 80-100% implemented compared to international best practice

There is also a 'cannot rate' option, but please only use this if really needed and provide comments in the comment box on why you cannot rate for a particular good practice indicator.

The ratings require expert judgment, taking multiple considerations into account:

1. The Quality of government policies/actions compared to international best practice. For example, a voluntary scheme will often be considered weaker than an implemented regulation.

2. The Extent of implementation of government policies/actions compared with international best practice, considering all aspects of the 'policy cycle':

- Agenda setting and initiation
- Policy development
- Implementation
- Evaluation

3. You should not take into account health outcomes such as overweight and obesity in the population.

4. You should consider the status of policy as it is now, and not how it may change over time.

5. Some indicators have shared national/ EU jurisdiction. For example: there is shared jurisdiction only for COMP1 and COMP2 on trans-fat and for LABEL 1-3. Therefore, this needs to be taken into account during the rating process.

The ratings thus need to take into account the intentions and plans of the Government, government funding for implementation of actions undertaken by NGOs and establishment of working or advisory groups, etc., in addition to the policies and actions that have been implemented.

The Food - EPI evidence document gives you the full details of the current evidence of implementation by the Irish Government for each good practice indicator and includes international best practice examples (benchmarks) for each good practice indicator to support you in the rating process and give you confidence to make those judgements. The evidence and the benchmarks are also available within the online questionnaire used for the rating process. It is important to read the evidence of implementation and international best practice exemplars (benchmarks) before putting in your rating for each good practice indicator

Introduction to the socio economic inequalities aspect of Food-EPI

Large socio economic inequalities in diet exist, which in turn affects nutrition and health outcomes. People with a lower socio-economic status ("SES", that is those with a lower educational level and/or lower income level) consume on average more unhealthier foods (energy-dense high-processed foods) than people with a high SES. It is preferable that the implementation of food policies leads to a reduction of SES inequalities in diet (and not to a widening of SES inequalities) and that, policies have a larger positive effect on the diets of lower SES groups than high SES groups. Therefore, during the rating of each Food-EPI indicator (for the 6 policy domains only), we invite you to indicate how and to which degree you think that the implementation of each Food-EPI indicator can impact on socioeconomic inequalities in diet in Ireland, and in which direction (reduction or widening of inequalities).

NOTE: You do not need to consider the extent of implementation in your country but only consider the ideal good practice description.

For example:

COMP1 - Food composition targets/standards have been established for processed foods by the government for the content of the nutrients of concern in certain foods or food groups if they are major contributors to population intakes of these nutrients of concern (added sugars in processed foods, salt in bread, saturated fat in commercial frying fats).

ANSWER OPTIONS:

- Could lead to a considerable reduction of SES inequalities.
- Could lead to a small reduction of SES inequalities
- No impact on SES inequalities.
- Could lead to a small widening of SES inequalities
- Could lead to a considerable widening of SES inequalities.
- I don't know

Definitions

- **Food:** refers to food and non-alcoholic beverages. It excludes breastmilk or breastmilk substitutes.

- **Food environments:** the collective physical, economic, policy and socio-cultural surrounding, opportunities and conditions that influence people's food and beverage choices and nutritional status.

- **Government:** includes any government departments and, where appropriate, other agencies (i.e. statutory bodies such as offices, commissions, authorities, boards, councils, etc). Plans, strategies or actions by local government should not be included, although relevant information can be noted in the 'context/comments' sections.

- **Government implementation:** refers to the intentions and plans of the government and actions and policies implemented by the government as well as government funding for implementation of actions undertaken by non-governmental organisations, academic institutions, private companies (including consultants), etc.

- **Healthy/unhealthy food:** Categorisation of foods as healthy / unhealthy are in accordance with the WHO and EU guidelines). Where it is not clear which category to use, categorisation of foods should be informed by rigorous criteria or the use of a nutrient profiling model.

- **Nutrients of concern:** salt (sodium), saturated fat, trans fat, added sugar.

- **Systems-based approaches**: This may include policies within other domains of health, a social-determinants of health approach, bringing together multiple departments or ministries to approach health and includes multiple levels of government.

- **Policy actions:** A broad view of "policy" is taken to include all government policies, plans, strategies and activities. Only current policy actions are considered, generally defined as policy activity of the previous 12 months (except where otherwise specified). Evidence of policy implementation takes consideration of the whole policy cycle, from agenda-setting, through to policy development, implementation and monitoring. A broad view of relevant evidence was taken, to include, inter alia:
    - Evidence of commitments from leadership to explore policy options
    - Allocation of responsibility to an individual/team (documented in a work plan, appointment of new position)

- Establishment of a steering committee, working group, expert panel, etc.
- Review, audit or scoping study undertaken
- Consultation processes undertaken
- Evidence of a policy brief/proposal that has been put forward for consideration
- Preparation of a regulatory or economic impact assessment, health impact assessment, etc.
- Regulations / legislation / other published policy details
- Monitoring data
- Policy evaluation report

Abbreviations

BAI: Broadcasting Authority of Ireland

CAP: Common Agricultural Policy

CFP: Common Fisheries Policy

CN: Combined Nomenclature

COSI: Childhood Obesity Surveillance Initiative

CVD: Cardiovascular Disease

EC: European Commission

EFSA: European Food Safety Authority

EPHA: European Public Health Alliance

EU: European Union

FDI: Food Drink Ireland

Food EPI: Food Environment Policy Index

FIC: Food Information to the Consumer

FOP: Front of Pack

FSAI: Food Safety Authority of Ireland

GDA: Guideline Daily Amount

GUF: General University Funds

HEA: Higher Education Authority

HFFA: Healthy Food for All

HFSS: High in Fat, Sugar and/or Salt

HiAP: Health in All Policies

HIA: Health Impact Assessment

HIQA: Health Information and Quality Authority

HSE: Health Service Executive

IFR: Institute of Food Research

INFORMAS: International Network for Food and Obesity/ NCDs Research, Monitoring and Action Support

NCD: Non-Communicable Disease

NGO: Non-Government Organisation

NNSC: National Nutrition Surveillance Centre

OPIOG: Obesity Policy Implementation Oversight Group

PRTLI: Programme for Research in Third Level Institutions

SFI: Science Foundation Ireland

SID: Social Inclusion Division

SSDT: Sugar Sweetened Drinks Tax

TTIP: Transatlantic Trade and Investment Partnership

UCC: University College Cork

WHO: World Health Organization

Healthy Food Environment Policy Index: Policy domains

DOMAIN 1 - FOOD COMPOSITION: There are government systems implemented to ensure that, where practicable, processed foods minimise the energy density and the nutrients of concern (salt, fat, saturated fat, *trans* fat, added sugar).

**COMP1**<br>**Food composition targets/standards have been established for processed foods by the government for the content of the nutrients of concern in certain foods or food groups if they are major contributors to population intakes of these nutrients of concern (added sugars in processed foods, salt in bread, saturated fat in commercial frying fats).**<br>**(Trans fat has been excluded as it falls under EU regulation)**	
**Definitions and scope**	• Includes packaged foods manufactured in country X or manufactured overseas and imported to country X for sale.<br>• Includes packaged, ready-to-eat meals sold in supermarkets.<br>• Includes mandatory or voluntary targets, standards (e.g., reduce by X%, maximum mg/g per 100g or per serving).<br>• Includes legislated ban on nutrients of concern.<br>• Excludes legislated restrictions related to other ingredients (e.g. additives).<br>• Excludes mandatory food composition regulation related to vitamins and micronutrients (e.g. folic acid or iodine fortification)<br>• Excludes food consumption standards/targets for fibre, healthy ingredients like fruits and vegetables<br>• Excludes food composition of ready-to-eat meals sold in food service outlets (see COMP2)<br>• Excludes general guidelines advising food companies to reduce nutrients of concern.<br>• Excludes the provision of resources or expertise to support individual food companies with reformulation.<br>• Industrially processed foods are the processed and ultra-processed foods according to the **NOVA classification** (please find the complete definitions here: https://world.openfoodfacts.org/nova):<br>• Processed foods, such as bottled vegetables, canned fish, fruits in syrup, cheeses and freshly made breads, are made essentially by adding salt, oil, sugar or other substances from Group 2 (processed culinary ingredients) to Group 1 (unprocessed or minimally processed) foods.

	Ultra-processed foods, such as soft drinks, sweet or savoury packaged snacks, reconstituted meat products and pre-prepared frozen dishes, are not modified foods but formulations made mostly or entirely from substances derived from foods and additives, with little if any intact Group 1 (unprocessed or minimally processed foods) foods. The overall purpose of ultra-processing is to create branded, convenient (durable, ready to consume), attractive (hyper-palatable) and highly profitable (low-cost ingredients) food products designed to displace all other food groups.
**International best practice examples (benchmarks)**	**UK:** In 2016**,** a key commitment of the 'Childhood obesity: a plan for action' was to launch a broad, structured sugar reduction programme to remove sugar from everyday products. All sectors of the food and drinks industry were challenged to reduce overall sugar across a range of products that contribute most to children's sugar intakes by at least 20% by 2020, including a 5% reduction in the first year of the programme (August 2016 to August 2017). The overall reduction between 2015 -2018 (in total sugar per 100g) was -2.9%(Public Health England, 2019). Only three food groups of the eight measured have managed at least a 5% reduction in the first year: sweet spreads and sauces, yoghurts and fromage frais, and breakfast cereals. There has been no sugar reduction in biscuits and chocolate bars(Public Health England, 2018). In contrast to this co-regulation, for products where the sugar tax applies over the same period a reduction in sugar of about 30% was found.<br><br>**South Africa:** In 2013, the South African Department of Health adopted mandatory targets for salt reduction in 13 food categories by means of regulation (Foodstuffs, Cosmetics and Disinfectants Act). There is a stepped approach with food manufacturers given until June 2016 to meet one set of category-based targets and another three years until June 2019 to meet the next (World Cancer Research Fund, 2016b, Hofman and Tollman, 2013). Overall, 67% of targeted foods had a sodium level at or below the legislated limit. About half (49%) of targeted foods not meeting the legislated limits were less than 25% above the maximum sodium level (Peters et al., 2017).<br><br>**The Netherlands:** On January 2014, the Dutch Ministry of Health, Welfare and Sport signed an agreement with trade organizations representing food manufacturers, supermarkets, hotels, restaurants, caterers and the hospitality industry to lower the levels of salt, saturated fat and calories in food products. The agreement includes voluntary ambitions for the period up to 2020 and aims to increase the healthiness of the food supply (World Cancer Research Fund, 2016b, National Agreement to improve Product Composition, 2017). Voluntary agreements have been made for the

	reduction of salt, saturated fat and energy/sugars in a variety of product groups and soft drinks.
**Context** e.g. EU action/ regulation / food industry action etc.	**Commission Directive 2006/125/EC (OJ L339, p16, 06/12/2006)**<br><br>• Commission Directive 2006/125/EC (OJ L339, p16, 06/12/2006) of 5 December 2006 on processed cereal-based foods and baby foods for infants and young children.<br>- European Communities (Processed Cereal-based foods and baby foods for Infants and Young Children) Regulations, 2007 (S.I. No 776 of 2007) Commission Directive 2006/125/EC codifies and replaces the previous Directive (Commission Directive 96/5/EC).<br>- Commission Directive 2006/125/EC is transposed into Irish legislation by S.I. No 776 of 2007 and it sets the rules on the composition and labelling of processed cereal-based foods for nutritional use for infants and young children in good health and are intended for use by infants when they are being weaned and as a supplement to the diet of young children (The commission of the European communities, 2006).<br><br>**Food industry action**<br><br>• In 2016, Food Drink Ireland (FDI) published a report investigating the impact of reformulation efforts by 14 key FDI members, which identified a reduction in energy, total fat, saturated fat, sodium and sugar, of selected studied products in a 7-year period. It found that in the seven years between 2005 and 2012, the energy, total fat, saturated fat, sodium and sugar content of the products analysed had been reduced by 12%, 10%, 12%, 37% and 14% respectively (Food Drink Ireland, 2016)<br><br>**The FDI 'National Reformulation Programme'**<br><br>• Following the 2016 report, the FDI launched a *'National Reformulation Programme',* with the FSAI providing oversight, to continue their reformulation efforts in meeting consumers' changing requirements. FDI aims to expand the initiative and recruit more companies across the food industry to document the impact of their reformulation efforts on the population of Ireland. The FSAI believes that the reformulation of foods

must be driven by the industry, with the FSAI continuing its independent monitoring of efforts over the coming years.

- The FDI published a further report in 2019 entitled "The Evolution of Food and Drink in Ireland, 2005 – 2017" that, shows the decreases in sugar and saturated fat in Irish diets between 2005 and 2017 as a result of voluntary undertakings by food and drink companies. The main findings of the report are:

**Direct reformulation of products on the market in both 2005 and 2017**	**Reductions**
Sodium	28%
Saturated fat	10.1%
Sugar	8%
Energy	1.6%
Total fat	0.3%
**Reductions in sugar intake between 2005 and 2017**	**Reductions**
Adult sugar intake	0.8g/day
Teen sugar intake	2.7g/day
Child sugar intake	3.2g/day
Pre-schooler sugar intake	2.0g/day
**Reductions in saturated fat intake between 2005 and 2017:**	**Reductions**
Adult saturated fat intake	0.5g/day
Teen saturated fat intake	0.2g/day
Child saturated fat intake	0.2g/day
Pre-schooler saturated fat intake	remained constant

	Results for the other nutrients were more modest, with sodium, total fat and energy intake remaining relatively stable over the period (Food and Drink Ireland, 2019).<br>**Lidl Ireland**<br>• **Lidl Ireland** – On the 27th of March 2019 Lidl announced that, over 850 own-brand products (over 30% of the product range) are being assessed, as the retailer commits to a 20% reduction in added sugar content as well as reducing salt levels in accordance with best practice standards by the end of 2020. This comes as a response to the Irish Government-led initiative, Healthy Ireland, in particular the Obesity Action Plan and as a direct response to consumer's demand for healthier food choices.<br>• **Sugar:** Lidl Ireland will reduce the added sugar content in own brand products by 20% by the end of 2020 focusing primarily on foods that are popular with and consumed by children. Food categories such as breakfast cereals, spreads, sauces, and sweet confectionery will be a major focus. Lidl Ireland will be reviewing approximately 350 own brand products in order to reach this sugar target.<br>• **Salt:** Lidl Ireland will reduce the salt content of own-brand products in line with the UK's Food Standards Agency (FSA) 2017 salt targets by the end of 2020. The reduction of salt first focuses on food categories that are consumed on a regular basis and generally make up a large share of the daily salt intake including; ready meals, soups, pizzas, crisps, cakes and meat products. Over 500 of Lidl Ireland's own brand products will be reviewed to reach this salt target (Lidl, 2019).
**Evidence of implementation**	**The Obesity Policy Implementation Oversight Group (OPIOG)**<br>• A subgroup of the Obesity Policy Implementation Oversight Group (OPIOG) was established with a term of reference to recommend guidelines for food reformulation and a mechanism for engagement with the food industry. This food reformulation subgroup was established in January 2018, is chaired by Prof Ivan Perry, School of Public Health in UCC. The OPIOG will report to the Department of Health by 2020.<br>• In Step three of the ' *A Healthy Weight for Ireland: Obesity Policy and Action Plan 2016 -2025* , the Irish Government planned to agree food reformulation targets with the food industry (Department of Health., 2016). Leads were identified and deemed responsible for this step including, the Department

JPI a healthy diet for a healthy life
PEN Policy Evaluation Network
FOOD EPI
INFORMAS Benchmarking food environments

	of Health, Irish Business and Employers' Confederation, Food Drink Ireland (FDI) and the Food Safety Authority of Ireland (FSAI), Department of Agriculture, Food and the Marine as a potential partner.<br><br>**Voluntary reformulation / composition targets**<br><br>**The FSAI salt reduction programme**<br><br>• In January 2016, The FDI issued a first report estimating the impact of reformulation by some of its food industry members on the Irish population (Food Drink Ireland, 2016).<br>• The FDI published a further report in 2019 entitled "The Evolution of Food and Drink in Ireland, 2005 – 2017" that showed the decreases in sugar and saturated fat in Irish diets between 2005 and 2017 because of voluntary undertakings by food and drink companies.<br><br>*(Details of the findings from both reports are detailed in the 'context' section above).*<br><br>• Coupled with this industry-led reformulation programme will be a continuation of the FSAI's independent surveillance and commentary on the commitments and achievements of the industry in relation to salt reduction.<br>• The FSAI published an update of its annual salt monitoring programme in November 2016. An examination of 530 samples of processed food was conducted in 2015, across four food product categories including: processed meats, breads, breakfast cereals and spreadable fats. Significant reductions in salt were found across a variety of products, most notably in processed meats such as rashers, cooked ham and sausages. However, while levels of salt have decreased in processed foods, the average dietary salt intake in Irish adults is currently 11.1 g salt per day in men and 8.5 g salt per day in women (Food Safety Authority of Ireland, 2016d).<br><br>**Through the salt reduction programme the FSAI established 7 objectives:**<br><br>• Raise awareness in the food industry of the salt and health issue, the role of processed food in salt intake and the health gains to the Irish population of reducing salt in processed food.<br>• Focus on the manufacturers of food in the food groups that contribute most salt to the diet, and secure gradual and sustained reductions in the salt content of their food working on a united front across each sector.

	• Bring on board the manufacturers of food in other food groups that contribute to salt intake and secure gradual and sustained reductions in the salt content of their food working on a united front across each sector.<br>• Work with the food industry to bring about the universal labelling of salt in packaged foodstuffs.<br>• Target the retailers of food who set specifications for own brand processed food and have strong influence on manufacturers through their buying power. Secure gradual and sustained salt reductions in own brand processed food and start to focus on stocking low salt options of branded processed food.<br>• Target catering representative bodies and companies to secure a reduction in the use of salt in prepared food eaten outside the home.<br>• Work with other State bodies whose role it is to increase consumer understanding of the salt and health issue and bring about behavioural change in consumers (Food Safety Authority of Ireland, 2014b).<br><br>**Guidelines for use of potassium**<br><br>• The FSAI in 2018 has begun work on developing guidelines for the food industry on the use of potassium and other mineral-based salt replacement ingredients. These guidelines, when issued will consider the following:<br>• Possible effects of the use of these ingredients on vulnerable groups<br>• Types of replacement ingredients required by the food industry<br>• Types of foods in which these ingredients would be used and at what levels<br>• Likely reductions in salt levels in these foods<br>• Impact on actual sodium reduction in foods<br>• Impact on potassium intakes in the population (Food Safety Authority of Ireland, 2016c, Food Safety Authority of Ireland, 2018a)
**Comments/notes**	

**COMP2**<br>**Food composition targets/standards/restrictions have been established by the government for the content of the nutrients of concern (added sugars, salt, saturated fat) in meals sold from food service outlets, in particular for those food groups that are major contributors to population intakes of those nutrients of concern.**<br>**(Trans fat has been excluded as it falls under EU regulation)**	
**Definitions**<br><br>**and scope**	• Meals sold at food service outlets include foods sold at quick service restaurants, dine-in restaurants and take-away outlets, coffee, bakery and snack food outlets (both fixed outlets and mobile food vendors). This also includes foods from catering operations and delivery meals.<br>• Includes legislated bans on nutrients of concern<br>• Includes mandatory or voluntary targets (i.e. reduce by X%, maximum mg/g per 100g or per serving)<br>• Excludes legislated restrictions related to other ingredients (e.g. additives)<br>• Excludes mandatory out-of-home meal composition regulations related to vitamins and micronutrients, e.g. folic acid or iodine fortification<br>• Excludes food consumption standards/targets for fibre, healthy ingredients like fruits and vegetables<br>• Excludes general guidelines advising food service outlets to reduce nutrients of concern<br>• Excludes the provision of resources or expertise to support food service outlets with reformulation
**International best practice examples (benchmarks)**	**New Zealand:** In New Zealand, The Chip group, funded 50% by the Ministry of Health and 50% by industry, aims to improve the nutritional quality of deep-fried chips served by food service outlets by setting an industry standard for deep frying oils. The standard for deep frying oil is maximum 28% of saturated fat, 3% linoleic acid and 1% of trans-fat. The Chip group oil logo for use on approved oil packaging was developed in 2010 (The Chip Group, 2016).<br><br>**New York City (US):** In 2009, New York City established voluntary salt guidelines for restaurant and store-bought foods. In 2010, this evolved into the National Salt Reduction Initiative that encouraged nationwide partnerships among food manufacturers and restaurants involving more than 100 city and state health authorities to reduce excess sodium by 25% in packaged and restaurant foods. In 2012, 26% of the categories met the targets, and 3% met the targets by the end

	of 2014. Between 2009 and 2014, there was nearly a 7% reduction in sodium levels in the U.S. food supply (New York City Health, 2017). There are 28 companies, including packaged food corporations and restaurants, who are committed to the salt reduction targets (Department of Health, 2014). In July 25, 2019, the Voluntary Sugar Reduction Targets from the National Salt and Sugar Reduction Initiative were revised. There is an open technical comment period until September 30, 2019 (NYC Health Department, 2019).
**Context** e.g. EU action/ regulation / food industry action etc.	**EU Action**<br><br>The EU has not made a distinction in their policy documents between the nutrients of concern in industrially processed foods and in meals sold from food service outlets.<br><br>**The EU Framework for National Salt Initiatives**<br><br>• Restaurant meals, catering meals and ready meals are identified as 3 of the 12 food categories in the framework. The framework prescribes that at least in four food categories including 'ready meals' the lowest possible salt levels ('best in class' levels) are identified at EU level.<br><br>**Annex on Saturated Fat**<br><br>• The Annex on Saturated Fat indicated 5 food categories for which the lowest possible levels (best in class levels) would be identified at EU level. Ready meals (saturated fat) and food items served in modern (saturated fat) restaurants are part of these 5 categories.<br><br>**Annex on Added Sugars**<br><br>• The annex on Added Sugars prioritises 13 food categories including ready meals, school food offers and catering meals.<br><br>**Regulation on trans fats**<br><br>• The regulation on trans fats sets a maximum limit of trans fat (other than trans fat naturally occurring in fat of animal origin) in food which is intended for the final consumer and food intended for supply to retail. So this is also including meals sold from food service outlets.<br><br>**EU regulation**

	On **4 October 2018** the Commission published a draft Commission Regulation, amending Annex III to Regulation (EC) No 1925/2006 of the European Parliament and of the Council as regards trans-fat, other than trans-fat naturally occurring in animal fat, in foods intended for the final consumer. On 24 April 2019, the Commission adopted this regulation (European Comission).<br><br>**Main elements of the regulation**:<br><br>• A maximum limit of trans fat, other than trans fat naturally occurring in fat of animal origin, in food which is intended for the final consumer and food intended for supply to retail, of 2 grams per 100 grams of fat<br>• Definitions of "fat" and of "'trans fat" in line with the definitions in Annex I to Regulation (EU) No 1169/2011<br>• Definition of "retail" in line with Article3(7) of Regulation (EC) No 178/2002<br>• An obligation for business to business transmission of information on the amount of trans fat in foods when it exceeds the limit of 2% of fat<br>• Food which does not comply may continue to be placed on the market until 1 April 2021
**Evidence of implementation**	• **No evidence found from 2016 onwards**
**Comments/notes**	

DOMAIN 2 - FOOD LABELLING: There is a regulatory system implemented by the government for consumer-oriented labelling on food packaging and menu boards in restaurants to enable consumers to easily make informed food choices and to prevent misleading claims.

**LABEL1**<br>**Ingredient lists and nutrient declarations in line with Codex recommendations are present on the labels of all packaged foods**
Label 1 will not be including for benchmarking at national level as it's being addressed at EU level

**LABEL2**<br>**Evidence-based regulations are in place for approving and/or reviewing claims on foods, so that consumers are protected against unsubstantiated and misleading nutrition and health claims.**
Label 2 will not be including for benchmarking at national level as it's being addressed at EU level

**LABEL3**<br>**One or more interpretive, evidence-informed front-of-pack supplementary nutrition information system(s) endorsed by the Government, which readily allow consumers to assess a product's healthiness, is/are applied to all packaged foods (examples are the NutriScore and traffic lights).**	
**Definitions**<br><br>**and scope**	• Nutrition information systems include traffic light labelling (overall or for specific nutrients); Warning labels; Nutriscore; star or points rating; percent daily intake.<br>• Keyhole and Finish heart symbol are not considered FOP labelling systems (but rather claims).<br>• 'Evidence-informed' refers to systems that utilise robust criteria (based on an extensive review of up-to-date research and expert input) or a validated nutrient profiling model to inform decision-making about the product's healthiness.
**International best practice examples (benchmarks)**	**UK:** In 2013, the Government published national guidance for voluntary 'traffic light' labelling for use on the front of pre-packaged food products. The label uses green, amber and red to identify whether products contain low, medium or high levels of energy, fat, saturated fat, salt and sugar. A combination of colour coding and nutritional information is used to show how much fat, salt and sugar and how many calories are in each product. The voluntary scheme is used by all the major retailers and some manufacturers (Deparment of Health, 2013).<br><br>**Australia/New Zealand:** The government-approved, voluntary 'Health Star Rating' (HSR) scheme applies a stare rating system where ratings range from ½ star (least healthy) to 5 stars (most healthy). The rating is based on the content of energy, saturated fat, sodium and total sugars content, along with certain 'positive' aspects of a food such as fruit and vegetable content, and in some instances, dietary fibre and protein content. Implementation of the system began in June 2014 and is overseen by a number of governmental instances, one of which evaluates progress. As of 2016, about 900 products had stars on them (Commonwealth of Australia, 2016).<br><br>**France/ Belgium/Germany/Spain:** Since October 2017, the five-colour NutriScore, the official voluntary label for France has been implemented as the official, voluntary FOP scheme in four European countries. It aims to limit the consumption of foods high in energy, saturated fats, sugar or salt, in the context of an overall improvement in the nutritional quality of diets (World

	Health Organisation, 2017). Based on a scientific algorithm, each product is given a score based on the content of the nutrients of concern (energy value and the amount of sugars, saturated fats and salt) and positive ones (the amount of fibre, protein, fruit, vegetables and nuts) (Colruyt Group, 2018). The system was developed by the Nutritional Epidemiology research Team at the University of Paris (Chantal, 2017).<br><br>**<u>Table1: The Nutri-Score (Colruyt Group, 2018)</u>**<br><br>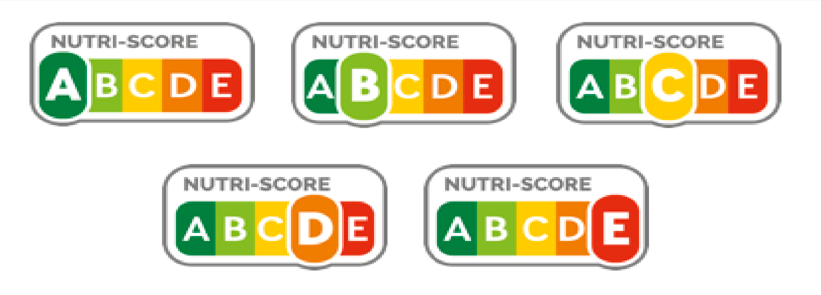
**Context** e.g. EU action/ regulation / food industry action etc.	**EU Action**<br>• There are currently no mandatory interpretive, evidence-informed front-of-pack supplementary nutrition information system(s) set /proposed by the European Union.<br><br>**Regulation (EU) 1169/2011 on food information to consumers**<br><br>• The Regulation (EU) 1169/2011 on food information to consumers contains rules for <u>mandatory nutrition declaration (as was also described in LABEL 1)</u>: (a) energy value (in both kilojoules (kJ) and kilocalories (kcal)); and (b) the amounts (in grams (g)) of fat, saturates, carbohydrate, sugars, protein and salt. This nutrition information (energy value and amounts of nutrients) must be expressed per 100g or per 100ml of the food. Nutrition information per portion can be given in addition.<br><br>• There have been developments in the expression of the nutrition declaration, other than per 100 g, per 100 ml or per portion (which is mandatory under Regulation (EU) 1169/2011 on food information to consumers), or in its presentation, through the use of graphical forms or symbols, by some Member States and organisations in the food

	sector. Such additional forms of expression and presentation may help consumers to better understand the nutrition declaration.<br><br>• The initial legislative proposal of the Commission included mandatory indication of energy, fat, saturates, carbohydrates, sugars and salt on the front of the pack, but this was rejected by the Committee on the Environment, Public Health and Food Safety (Nikolaas Tilkin-Franssens).<br><br>• An EU wide traffic light system was not part of the proposal, but had been discussed during the consultation process (Corporate Europe Observatory, 2010). While health and consumer organisations opted for a traffic light system giving consumers a visual warning for high fat, sugar or salt content of a product, The Confederation of the Food and Drink Industry of the EU (CIAA) has opposed proposals for front-of-pack 'traffic light' labels (Euractive, 2013, Euractive, 2014).<br><br>**NutriScore**<br><br>• A NutriScore initiative has been implemented in Belgium, France and Spain. Several other countries are considering its implementation. A NutriScore is a logo that shows the nutritional quality of food products using five colour-coded boxes with letters in them – A in dark green to E red. The grade is determined by the amount of healthy and unhealthy nutrients, taking into account salt, sugar and fat content as well as positive nutrients such as vitamins.<br><br>**Food Industry**<br><br>**Tesco**<br><br>• A labeling scheme called Guideline Daily Amounts (GDA) was introduced by Tesco in 2006 and also adopted by some other manufacturers (Tesco, 2008). Furthermore, in August Tesco announced plans to add traffic-light labels to the nutritional

	information it puts on products and indicated that its roll out of the system in Ireland would mirror that of the UK (Michelle Russell, 2012).<br><br>**Lidl**<br><br>• Implementation of the traffic-light system in Ireland in 2014 (Michelle Russell, 2012).
**Evidence of implementation**	• Currently there is not any work being done to consider the implementation of an interpretive, evidence informed front of pack supplementary nutrition information system in Ireland.<br><br>**Food Safety Authority of Ireland (FSAI)**<br><br>• According to FSAI, Front of Pack (FoP) labelling is a voluntary initiative that gives the consumer a quick glimpse of 5 main nutrients in the product, namely calories, sugar, fats, saturates (saturated fat), and salt. Where the mandatory nutrition information is declared on the label, certain nutrients may be repeated in the 'principle field of vision', i.e. the front of pack. This repeated information is a voluntary measure but where a food business chooses to provide this additional declaration, only the following information can be provided:<br>- Energy only or energy along with fat, saturates, sugar and salt<br><br>**This repeated information may be provided:**<br>- Per 100g/ml only<br>- Per 100g/ml and per portion or<br>- On a per portion basis only<br>- When providing this 'Front of Pack' information Energy must always be indicated per 100g/ml as a minimum.<br>**(Food Safety Authority of Ireland, 2016a)**<br><br>***Table 6: Repeated information for front of pack***<br>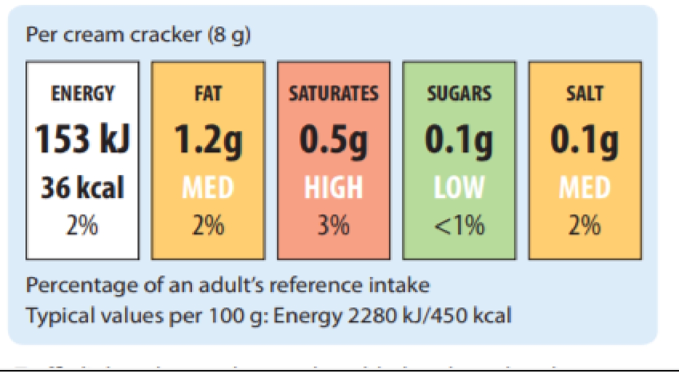

Comments/notes	

LABEL4<br>**A simple and clearly-visible system of labelling the menu boards of all quick service restaurants (i.e. fast food chains) is applied by the government, which allows consumers to interpret the nutrient quality and energy content of foods and meals on sale.**	
**Definitions and scope**	• Quick service restaurants: In the context of Ireland, this definition includes fast food chains as well as gas stations, kiosks, coffee, bakery and snack food chains. It may also include supermarkets where ready-to-eat foods are sold.<br>• Definition Euro monitor: Fast food outlets offer limited menus that are prepared quickly. Customers order, pay and pick up their order from a counter. Outlets tend to specialize in one or two main entrees such as hamburgers, pizza, ice cream, or chicken, but they usually also provide salads, drinks, dessert etc. Food preparation is generally simple and involves one or two steps, allowing for kitchen staffs generally consisting of younger, unskilled workers. Other key characteristics include:<br>- A standardised and restricted menu<br>- Food for immediate consumption<br>- Tight individual portion control on all ingredients and on the finished product;<br>- Individual packaging of each item<br>- Counter service<br>- A seating area, or close access to a shared seating area, such as in a shopping centre food court<br>- For chained fast food, chained and franchised operations which operate under a uniform fascia and corporate identity.<br>- Take out is generally present, as is drive-through in some markets.<br>• Labelling systems: Includes any point-of-sale (POS) nutrition information such as total kilojoules; percent daily intake; traffic light labelling; star rating, or specific amounts of nutrients of concern, salt warning labels.<br>• Includes endorsement schemes (e.g., accredited healthy choice symbol) on approved menu items
**International best practice examples (benchmarks)**	**South Korea**: Since 2010, the Special Act on Safety Control of Children's Dietary Life has required all chain restaurants with 100 or more establishments to display nutrient information on menus including energy, total sugars, protein, saturated fat and sodium (World Cancer Research Fund, 2016c).

	**Canada:** In effect since 1 January 2017, Ontario's Healthy Menu Choices Act 2015, requires food service premises that are part of a chain of 20 or more food service premises in Ontario (as well as certain cafeteria-style food service premises) to display calories for "standard food items" on menus, labels and display tags. The Act's regulations specify where caloric information is to be displayed on the menus, as well as the size, format and prominence of the display (Nutrition Resource Centre, 2017). Food service premises must also display information on daily caloric requirements: "*Adults and youth (ages 13 and older) need an average of 2,000 calories a day, and children (ages 4 to 12) need an average of 1,500 calories a day. However, individual needs vary.*"<br><br>Ontario's 36 public health units are responsible for implementation of the Act (Nutrition Resource Centre, 2017)<br><br>**Saudi Arabia**: In 2018, the Saudi Food and Drug Authority (SFDA) introduced mandatory measures on calorie labels on menus. These measures apply to all food facilities including restaurants, ice cream parlours, juice and fresh fruit vendors, bakeries, sweets shops, cafeterias, supermarkets, recreation facilities, colleges, universities and government agencies. Calories will be displayed at cashier desks, menu boards, table menus, drive-through menus, phone and web applications (Saudi Food & Druf Authority, 2018)<br><br>**USA:** In the US, the Patient Protection and Affordable Care Act (2010) (Office of the Federal Register, 2013) requires that all chain restaurants with 20 or more establishments to display energy information on menus. The menu labelling rule was implemented in May 2018 (Administration, 2019). The regulations will be pre-empted by the national law once implemented; local governments will still be able to enact menu labelling regulations for establishments not covered by national law. The regulations require vending machine operators of more than 20 vending machines to post calories for foods where the on-pack label is not visible to consumers by 26 July 2018 (World Cancer Research Fund, 2016c).<br><br>**Australia:** Legislation in Australian Capital Territory (Food Regulation 2002) and the States of New South Wales (Food Regulation 2010) and South Australia (Food Regulation 2002) requires restaurant chains (e.g. fast food chains, ice cream bars) with ≥20 outlets in the state (or seven in the case of ACT), or 50 or more across Australia, to display the kilojoule content of food products on their menu boards. Average adult daily energy intake of 8700kJ must also be prominently featured. Other chains/food outlets are allowed to

	provide this information on a voluntary basis but must follow the provisions of the legislation (World Cancer Research Fund, 2016c).<br><br>**New York City, USA:** Chain restaurants are required to put a warning label on menus and menu boards, in the form of a salt-shaker symbol (saltshaker inside a triangle), when dishes contain 2,300 mg of sodium or more. It applies to food service establishments with 15 or more locations nationwide. In addition, a warning statement is required to be posted at the point of purchase: This came into effect 1 December 2015 (World Cancer Research Fund, 2016c) (Department of Health and Mental Hygiene) and the Health Department started issuing violations June 6, 2016. Findings showed that directly following the sodium warning label regulation coming into effect, about 21% of restaurants had implemented the labels. By the end of February 2015, almost 70% of restaurants (from six of the ten chains) had implemented labels at one location or more. Overall, the findings suggested that the majority of restaurants were complying with the sodium warning label policy, despite issues with visibility, but that the labels may not be influencing consumer purchasing decisions (Downs, 2017).
**Context** e.g. EU action/ regulation / food industry action etc.	**EU Action**<br><br>• There is no system at European level which prescribes the labelling of menu boards at quick service restaurants, which allows consumers to interpret the nutrient quality and energy content of foods and meals on sale.<br><br>**Regulation (EU) No 1169/2011 on food information to consumers**<br><br>• Only the allergen information for non-prepacked food, including in restaurants and cafes is mandatory with the Regulation (EU) No 1169/2011:<br><br>**University College Cork**<br><br>• In 2018, UCC published an evaluation and exploration of Irish food-service businesses' uptake of and attitudes towards a voluntary government-led menu energy (calorie) labelling initiative. The results found that, in the telephone survey, 7 % (n 42) of food businesses reported displaying calories and the observation visits revealed that of these businesses, 10 % (n 4) were not displaying calorie information. Three major themes emerged from the semi-structured interviews: uncertainty, impact on business and consumer nutrition knowledge. Participants expressed concerns regarding inaccuracies in the calorie information, cost and time implications, mistrust in the

	food-service industry and poor nutritional knowledge among consumers. These concerns impeded the implementing of calorie menu labelling.<br><br>• The findings concluded that, calorie labelling should be implemented as part of a large-scale systemic programme of multiple obesity prevention strategies. To facilitate this and improve levels of compliance with calorie menu labelling, a collaborative approach between policy makers, academics and food-service business owners is needed to facilitate implementation. This approach should incorporate providing food-service business owners with guidance, support and practical assistance, along with a reasonable legislative structure and a standardised monitoring system (Fitzgerald et al., 2018)
**Evidence of implementation**	• In 2012, the Minister for Health in Ireland called on all standard food service businesses to voluntarily display calories on food and drinks served in Ireland under the calorie menu labelling scheme. This came as result of a national consultation which found that a vast majority of consumers in Ireland (over 95%) want calorie information on menus (Food Safety Authority of Ireland., 2012). The main reasons given for wanting calorie menu labelling in all outlets were 'informed decision making' (46%), 'fairness and equality amongst all food outlets' (10%), and 'to encourage healthier eating' (3%). Those wanting calorie labelling in some outlets want it in; fast food outlets (95%), followed by coffee shops and delis (63%), cinemas (58%), vending machines (57%), pubs (26%), and fine-dining restaurants (18%). Significantly more submissions from food businesses, compared to submissions involving those with any other background, did not want calorie menu labelling in any food outlet. (FSAI, 2012).<br><br>• In Step two of the 'A *Healthy Weight for Ireland: Obesity Policy and Action Plan 2016-2025*, the Irish Government plan to *"regulate for a healthier environment"* by developing, implementing and evaluating calorie posting legislation. This

	will be led by the Department of Health with input from public sector organisations, with the view of implementing evidence informed regulation. The timeline is 2016-2025 (Department of Health., 2016).<br><br>• At present, calorie posting legislation is currently in preparation (The Irish Times, 2019).<br><br>• Food service businesses have been provided with initial support to help them implement calorie menu labelling. MenuCal, the menu calculator has been developed to assist food businesses in Ireland comply with their legal requirements to display allergen information on foods. It also enables food businesses to calculate calories for their menus. It has been developed with the input of chefs, caterers and small business owners (Food Safety Authority of Ireland, 2019).<br><br>• The FSAI have developed a best practice calorie menu labelling system with appropriate guidance to be adopted, centred on the four principles and based on the technical guidance for provided consultation (Food Safety Authority of Ireland, 2012)<br><br>**Principle 1.** Calorie information is provided for ALL standard food and drink items sold A 'standard' food or drink item is a product that: 1) Is on sale for at least 30 days a year; and 2) Remains the same each time it is made. In this way, calorie information calculated on these food and drink items remains accurate.<br>**Principle 2.** Calorie information is displayed clearly and prominently at the 'point of choice' for the consumer. Calorie information must be given clearly and prominently beside the price. Individual businesses must decide how to best arrange this according to their situation.<br>**Principle 3.** Calorie information is provided per portion or per meal Calorie information should be provided based on the food

	and drink served to an individual consumer. Therefore, calorie information must be provided per portion or per meal served.<br>**Principle 4.** Information on how many calories an average person needs in a day is given to help consumers 'make sense' of calories on menus. the average amount of calories women, men and children need every day will be displayed to help consumers 'make sense' of calorie information on foods and drinks on sale.
**Comments/notes**	

DOMAIN 3 - FOOD PROMOTION: There is a comprehensive policy implemented by the government to reduce the impact (exposure and power) of promotion of unhealthy foods to children across all media.

- Exposure of food marketing concerns the reach and frequency of a marketing message. This is dependent upon the media or channels which are used to market foods.
- The power of food marketing concerns the creative content of the marketing message. For example, using cartoons or celebrities enhances the power (or persuasiveness) of a marketing message because such strategies are attractive to children.

**PROMO1**<br>**Effective policies are implemented by the government to restrict exposure and power of promotion of unhealthy foods to children including adolescents through broadcast media (TV, radio).**	
**Definitions and scope**	• Includes mandatory policy (i.e. legislation or regulations) or voluntary standards, codes, guidelines set by government or by industry where the government plays a role in development, monitoring, enforcement or resolving complaints (i.e. co-regulation).<br>• Includes free-to-air and subscription television and radio only (see PROMO2, PROMO3 and PROMO5 for other forms of media).<br>• Effective means that the policies are likely to reduce overall exposure of children, including adolescents to unhealthy food advertising over the day.
**International best practice examples (benchmarks)**	**Quebec:** In Québec, the Consumer Protection Act prohibits commercial advertising (including food and non-food) directed at children less than 13 years of age through television, radio and other media. Account must be taken of the context of its presentation, and in particular of: a) the nature and intended purpose of the goods advertised; b) the manner of presenting such advertisement; and c) the time and place it is shown. A cut-off of 15% share of child audience is used to protect children from TV advertising (Kent et al., 2011). Per indictment, a person is liable to: a fine ranging from $600 to $15,000 (in the case of a natural person); a fine ranging from $2,000 to $100,000 (in the case of a legal person).<br><br>**Norway/Sweden:** Under the Broadcasting Act, advertisements (food and non-food) may not be broadcast on television directed to children or in connection with children's programs. (World Cancer Research Fund, 2016e). Norway has implemented a self-regulation scheme approved and evaluated

	by Government. The scheme prohibits child-targeted unhealthy food marketing before 21:00 (9 PM) (MFU, 2016)<br><br>**Ireland:** Advertising, sponsorship, teleshopping and product placement of foods high in fats, sugars and salt, as defined by a nutrient profiling model, are prohibited during children's TV and radio programmes where over 50% of the audience are under 18 years old (Broadcasting Authority of Ireland, 2013). In addition, there is an overall limit on advertising of foods high in fats, sugars and salt adverts at any time of day to no more than 25% of sold advertising time and to only one in four advertisements. Remaining advertising targeted at children under the age of 18 must not include nutrient or health claims or include licensed characters (World Cancer Research Fund, 2016). As provided under the Broadcasting Act 2009, the BAI is required to undertake a statutory review of the effectiveness of the Children's Code. It is expected that review will commence in the second half of 2018 with revision and finalisation of the Code in 2019 (Broadcasting Authority of Ireland, 2019).<br><br>**Chile:** In June 2016, The Law of Nutritional Composition of Food and Advertising was enforced and restricts advertising directed to children under 14 years (for foods exceeding limits for calories, sugar, saturated fat and/or sodium in food and beverages). The regulatory norms define advertising targeted to children as programmes with an audience of greater than 20% children. Promotional strategies and incentives, such as cartoons, animations, and toys that could attract the attention of children are included in the ban. Monitoring and enforcement of the law are carried out by both regional and national public health authorities. Inspections are conducted on-site and online. After more than 2000 inspections, compliance with the law is improving, rising from under 40% to over 60% (Global Agricultural Information Network, 2018). A qualitative study carried out in 2017, found that the regulation has made mothers more aware of the importance of eating healthy, made it easier to choose healthy foods, and also made children actors in their own food choices (Correa et al., 2019).
**Context** e.g. EU action/ regulation / food industry action etc.	**Broadcasting Authority of Ireland**<br><br>• The BAI issued revised versions of its General and Children's Commercial Communications Codes in 2013 and again in 2017 (Broadcasting Authority of Ireland., 2013) (Broadcasting Authority of Ireland, 2017)

General Commercial Communications Code

- Include advertising, sponsorship and other forms of commercial announcements. These rules state that commercial communications for HFSS food (including drinks) shall not be permitted in children's programmes. HFSS Food is a sub category of food that is deemed high in fat, sugar and/or salt by the application of the Nutrient Profile model used by the Broadcast Authority of Ireland. In addition, content rules will apply to commercial communications for HFSS food broadcast outside of children's programmes but which are directed at children. Children are those under the age of 18. Adults are therefore defined as those 18 years and over. As the Codes will make specific recommendations for different age groups of children, they will, where appropriate, make reference to these age groups (Broadcasting Authority of Ireland, 2017)

- Such commercial communications shall not:
  - Include celebrities or sports stars;
  - Include programme characters;
  - Include licensed characters e.g. characters and personalities from cinema releases;
  - Contain health or nutrition claims;
  - Include promotional offers;
  - No more than 25% of sold advertising time and only one in four advertisements for HFSS food are permissible across the broadcast day on radio and television services.

- These rules also state that all children's commercial communications for fast food products, outlets and/or brands must display an acoustic or visual message stating *'should be eaten in moderation and as part of a balanced diet'.* Children's commercial communications for confectionery products must display an acoustic or visual message stating that *'snacking on sugary foods and drinks can damage teeth.'* 'Confectionery' in this instance includes sugar, honey, preserves, chocolate covered bars (excluding biscuits), non-chocolate confectionery – e.g. cereal bars – and artificial sweeteners. Carbonated drinks are included with the exception of water (Broadcasting Authority of Ireland., 2013).

	• The BAI regulates all content broadcast on all Irish licensed broadcasters, both programming and commercial content. In addition to processing broadcasting complaints, the BAI monitors broadcast content for compliance with broadcasting codes and rules.<br>**Children's Commercial Communications Code**<br>• The Children's Commercial Communications Code deals with advertising, sponsorship, product placement and other forms of commercial promotion aimed at children or broadcast in or around children's programming. It includes rules on the promotion to children of food that is high in fat, salt or sugar (HFSS food).
**Evidence of implementation**	**General Commercial Communications Code**<br>• The General Communications Code came into effect on the 1st of June 2017(Broadcasting Authority of Ireland, 2017).<br>**Children's Commercial Communications Code**<br>• As provided under the Broadcasting Act 2009, the BAI is required to undertake a statutory review of the effectiveness of the Children's Code. It is expected that review will commence in the second half of 2018 and the revised code is expected in 2020 (Broadcasting Authority of Ireland, 2019).
**Comments/notes**	**A report of Joint Committee on Children and Youth Affairs: Motion**<br>• The Dáil Éireann considered the report of the Joint Committee on Children and Youth Affairs entitled 'Tackling Childhood Obesity', copies of which were laid before the Dáil Éireann on 14th November, 2018. "The Government must amend regulations for broadcast media… to ensure regulations which prevent the marketing of junk and unhealthy foods to children" (A. Farrell, 2019).

**PROMO2**<br>**Effective policies are implemented by the government to restrict exposure and power of promotion of unhealthy foods to children including adolescents through online and social media.**	
**Definitions**<br><br>**and scope**	• Includes online media (e.g. social media, branded education websites, online games, competitions and apps)<br>• Where the promotion is specifically through other non-broadcast media than online and social media, this should be captured in 'PROMO3 and PROMO5'.<br>• Where the promotion is specifically in a children's setting, this should be captured in 'PROMO4'.<br>• Effective means that the policies are likely to reduce overall exposure of children, including adolescents to unhealthy food advertising over the day.
**International best practice examples (benchmarks)**	**Chile:** In June 2015, the Chilean authority approved the regulatory norms required for the law of Nutritional Composition of Food and Advertising implementation. The regulatory norms define limits for calories, saturated fat, sugar and sodium content considered "high" in foods and beverages. The law restricts advertising directed to children under the age of 14 years of foods in the "high in" category. The regulatory norms define advertising targeted to children as websites directed to children or with an audience of greater than 20% children, and according to the design of the advertisement. Promotional strategies and incentives, such as cartoons, animations, and toys are included in the ban. The regulation took effect 1 July 2016 and applies to all advertising media (World Cancer Research Fund, 2016).<br><br>**Quebec:** In Québec, the Consumer Protection Act prohibits commercial advertising directed at children less than 13 years of age through all media. Account must be taken of the context of its presentation, and in particular of: a) the nature and intended purpose of the goods advertised; b) the manner of presenting such advertisement; and c) the time and place it is shown (Kent et al., 2011). Any stakeholder involved in a commercial process (from the request to create an advertisement to its distribution, including its design) may be accused of not complying with the legislation in force. Per indictment, that person is liable to: a fine ranging from $600 to $15,000 (in the case of a natural person); a fine ranging from $2,000 to $100,000 (in the case of a legal person).

	**Norway**: A government-approved and evaluated self-regulation scheme prohibits online food-marketing which is targeted at children under 13 years. Specifically, interactive games "specifically aimed at children and where a product's trademark, or other elements of the marketing of the product, form an integral part" will always be defined as child-targeted and a violation of the code (36). The scheme also applies to social media. In 2019, the code was revised. The age limit is still 13 years but in order to exercise caution in marketing to young people, it is advised to not conduct contests with age limit less than 16 years; to buy age groups less than 16 years in digital media; to use role models appealing to youth in media which is directly targeted to youth; and to encourage engagement (share, like, send in material) so that youth become marketing actors. Violations of the code results in no other sanctioning than "naming and shaming" of offenders (Matbransjens Faglige Utvalg, 2019).<br><br>**UK:** UK CAP rules have been reviewed so that online marketing targeted to under-16s is prohibited. This means that HFSS product ads are not permitted to appear in media that is specifically targeted at under-16s (for example, a children's magazine or on a website aimed at children); or where under-16s make up a significant proportion (more than 25%) of the audience (for example, advertorial content with an influencer that might have broad appeal but also a significant child audience) (Advertising Standards Authority, 2018).
**Context** e.g. EU action/ regulation / food industry action etc.	**Advertising Standards Authority for Ireland (ASAI) Voluntary Codes of Practice**<br><br>**Rules for Non-Broadcast Digital Media**<br><br>**1.** Where appropriate age-filters exist on websites and social media apps, marketing communications for HFSS foods are not permitted to target children under the age of 15.<br>**2.** Marketing Communications for HFSS food by means of e-mail and Short Message Service (SMS) shall not target children under the age of 15.<br>**3.** Marketing Communications for HFSS food by means of social media shall not target children under the age of 15.<br>**4.** Where Marketing Communications for HFSS food is permissible, it shall not exceed a maximum of 25% of total advertising space.<br>**5.** The websites of food businesses should not carry content that is designed to engage children under the age of 15 with HFSS food

	brands e.g. children's area, videos, 'webisodes', branded education and interactive features.(Healthy Ireland, 2017b)
Evidence of implementation	**Government of Ireland – voluntary codes of practice**<br>• The Government of Ireland has issued a set of voluntary codes of practice aimed at limiting the promotion, marketing and sponsorship of foods high in fats, sugar and/or salt (HFSS foods). The new voluntary rules will apply to non-broadcast media, including digital, out of home, print and cinema, as well as commercial sponsorship and retail product placement. The government of Ireland has committed to monitor compliance, and work is continuing on the development of a monitoring mechanism (Department of Health, 2017).<br>**Rules for Non-Broadcast Digital Media**<br>• Where appropriate age-filters exist on websites and social media apps, marketing communications for HFSS foods are not permitted to target children under the age of 15.<br>• Marketing Communications for HFSS food by means of e-mail and Short Message Service (SMS) shall not target children under the age of 15.<br>• Marketing Communications for HFSS food by means of social media shall not target children under the age of 15.<br>• Where Marketing Communications for HFSS food is permissible, it shall not exceed a maximum of 25% of total advertising space.<br>• The websites of food businesses should not carry content that is designed to engage children under the age of 15 with HFSS food brands e.g. children's area, videos, 'webisodes', branded education and interactive features (Department of Health, 2017).
**Comments/notes**	The Codes detailed are voluntary in nature. Companies and partner organisations will sign up to the Codes and a register of signatories will be maintained and published by the Department of Health or its designated monitoring body.<br>Currently digital marketing of food to children in Ireland is subject to voluntary regulation by the advertising industry's Code (Advertising Standards

	Authority for Ireland; ASAI, 2015). Although the Code defines children as those under 18, and states that marketing should not 'encourage an unhealthy lifestyle or unhealthy eating or drinking habits' (Rule 8.16), this is open to interpretation as no definition of unhealthy eating or drinking habits is given and no Nutrient Profiling system is applied to define items that should not be advertised to children (Irish Heart Foundation, 2016)

**PROMO3**<br>**Effective policies are implemented by the government to restrict exposure and power of promotion of unhealthy foods to children including adolescents through non-broadcast media other than packaging and online/social media.**	
**Definitions**<br><br>**and scope**	• Non-broadcast media promotion includes: print (e.g. children's magazines), on/around public transport (e.g. signage, posters and billboards), cinema advertising, product placement and brand integration (e.g. in television shows and movies), direct marketing (e.g. provision of show bags, samples or flyers), or point-of-sale (POS) displays.<br>• Non-broadcast media is excluding the media covered through other indicators like online and social media (PROMO2) and packaging (PROMO5).<br>• Where the promotion is specifically in a children's setting, this should be captured in 'PROMO4'.<br>• Effective means that the policies are likely to reduce overall exposure of children, including adolescents to unhealthy food advertising over the day.
**International best practice examples (benchmarks)**	**Chile:** In May 2018, a new regulation launched, extended marketing restrictions of regulated foods in cinema and TV to a 6 AM to 10 PM time frame, expanding the scope of the original law. Additionally, starting in June 2018, any marketing done for "High in" foods or beverages must also show the following statement: "Choose foods with less warning labels" and then "Ministry of Health," which needs to be placed next to the MoH logo. This applies to marketing done in billboard, cinema, and other vehicles but food packages (Corvalán C, 2018).<br><br>**London UK:** On 25 February 2019, the Mayor of London, introduced restrictions on the advertising of unhealthy food across the entire Transport for London (TfL) public transport network, as part of his work to help tackle childhood obesity in London. The policy specifies that food and non-alcoholic drinks high in fat, salt and sugar (according to the UK Nutrient Profiling Model, are not permitted to be advertised on TfL-controlled buses, underground and over ground train networks, taxis, river services, trams and other transport systems. Food and drink brands, restaurants, takeaways and ordering services are required to promote their healthier food and drink instead of just advertising their brand. Advertisements for food and non-alcoholic drink products that are considered to be high in fat, salt, sugar may be considered for an exception by TfL if the advertiser can demonstrate, with appropriate

	evidence, that the product does not contribute to child obesity (Greater London Authority, 2019)
**Context** e.g. EU action/ regulation / food industry action etc.	**EU Action**<br><br>**The WHO report 'Tackling food marketing to children'**<br><br>• The WHO report, 'Tackling food marketing to children' in a digital world: trans-disciplinary perspectives' (World Health Organisation, 2016), that European Union competence is largely limited to marketing between Member States. For example, case law at the European Court of Justice has determined that, in view of the internal market, European Union regulations cannot apply to static marketing within a country (e.g. advertisements in hotels and airports, on billboards and shop awnings, umbrellas, ashtrays and similar items), advertisements screened in cinemas or sponsorship of events that have no cross-border appeal.<br><br>• Furthermore, policy action in the European countries to address forms of marketing beyond broadcast media, digital media and school settings is generally very limited, according to the WHO. Marketing avenues and techniques like sponsorship, product packaging, in-store promotions (e.g. at checkout, in the aisles), street billboards and prizes/giveaways or multi-buy promotions are rarely covered by governmental policies. Neither are settings where children gather, apart from schools, such as recreation facilities and leisure centres often included. Recent policy monitoring suggests that less than 20% of countries in the European Region cover one or more of these avenues/techniques (World Health Organisation, 2016).
**Evidence of implementation**	**Non-Broadcast Media Advertising and Marketing of Food and Non-Alcoholic Beverages, including Sponsorship and Retail Product Placement: Voluntary Codes of Practice (December, 2017)**<br><br>• **Rules for Out of Home Media** - Out of Home Media includes all out of home advertising and marketing communications delivered via such formats as billboards or hoardings, public transport stops or shelters, interiors and exteriors of buses or trains, or building banners (Healthy Ireland, 2017b).

1. Space limitation across the various HFSS product categories will be a total of 33% of the available space by cycle2 and by format.
2. Marketing Communications for HFSS food is not allowed on building banners.
3. Marketing Communications utilising wraparounds or takeovers for HFSS foods will account for less than 5% of the total available advertising space (Healthy Ireland, 2017b).

Additional Rules for Print Media

1. Marketing Communications for HFSS foods will only be carried in consumer publications where the adult readership is 75% or greater. A consumer publication or issue is taken to mean the complete edition published that day to include any supplements or advertising inserts.
2. Where Marketing Communications for HFSS food is permissible, it shall not, in ordinary circumstances, exceed a maximum of 25% of total advertising space.
3. HFSS food sponsorship of sports pages or sports supplements is not allowed (Healthy Ireland, 2017b).

Additional Rule for Cinema

1. Where Marketing Communications for HFSS food is permissible, it shall not exceed a maximum of 25% of total advertising space by screening (Healthy Ireland, 2017b).

Code of Practice Relating to Retail Product Placement of HFSS Foods

The arrangement of food products in the retail environment influences the purchase decisions of consumers. It is noted that the retail food industry acknowledges this fact and has agreed to a set of product placement measures that are designed to increase healthy food choices. Nevertheless, the retail industry is encouraged to go above and beyond these measures in the interests of promoting healthy eating. Accordingly, the retail food industry in Ireland undertakes to operate the specific rules for retail product placement as detailed below in addition to any relevant general rules applicable to all codes.

	**2.** Active promotion of the consumption of 5 to 7 portions of fruit and vegetables a day by customers in store through point of sale materials and other online and offline communication platforms.<br>**3.** Provision of meal deal offers that deliver choice for customers in order to promote a healthy balanced diet.<br>**4.** Where practical, the provision of a HFSS food free checkout option to customers to allow the use of a checkout lane that does not stock HFSS foods. In retail environments with 4 or more checkout bays, a minimum of 1 in 4 bays should be free of HFSS foods. Other outlets with less than 4 checkouts are encouraged to provide 1 non-HFSS checkout, where practical.<br>**5.** Provision of calorie labelling information in food service areas such as delis and hot food counters. Provision of calorie labelling will be in line with legal requirements and in their absence with 'Putting Calories on Menus in Ireland - Draft Technical Guidance for Food Businesses', is available free from the Food Safety Authority of Ireland website (www.fsai.ie) (Healthy Ireland, 2017b)
**Comments/notes**	The Codes detailed are voluntary in nature. Companies and partner organisations will sign up to the Codes and a register of signatories will be maintained and published by the Department of Health or its designated monitoring body.

**PROMO4** **Effective policies are implemented by the government to ensure that unhealthy foods are not commercially promoted to children including adolescents in settings where children gather (e.g. preschools, schools, sport and cultural events).**	
**Definitions and scope**	• Children's settings include: areas in and around schools, preschools/ kindergartens, day-care centres, children's health services (including primary care, maternal and child health or tertiary settings), sport, recreation and play areas/ venues/ facilities and cultural/community events where children are commonly present.<br>• Includes restrictions on marketing in government-owned or managed facilities/venues (including within the service contracts where management is outsourced)<br>• Includes restriction on unhealthy food sponsorship in sport (e.g. junior sport, sporting events, venues)<br>• Effective means that the policies are likely to reduce overall exposure of children, including adolescents to unhealthy food advertising over the day.
**International best practice examples (benchmarks)**	**Chile:** In June 2015, the Chilean authority approved the regulatory norms required for the Law of Nutritional Composition of Food and advertisings implementation. The regulatory norms define limits for calories, saturated fat, sugar and sodium content considered "high" in foods and beverages. The law restricts advertising directed to children under the age of 14 of foods in the "high in" category on school grounds, including preschools, primary and secondary schools. Chile has also restricted outdoor advertising, with ten municipalities adopting legislations banning outdoor marketing one block around schools (The Organisation for Economic Co-operation and Development, 2019). The law is scheduled to take effect in July 2016 (New York City Health, 2017). has also restricted outdoor advertising, with ten municipalities adopting legislations banning outdoor marketing one block around schools. The law is scheduled to take effect in July 2016 (New York City Health, 2017).<br><br>**Uruguay:** In September 2013, the government of Uruguay adopted Law No 19.140 (Healthy foods in schools) (Morley et al., 2013). The law prohibits the advertising and marketing of foods and drinks that don't meet the nutrition

	standards. Advertising in all forms is prohibited, including posters, billboards, and use of logos/brands on school supplies, sponsorship, and distribution of prizes, free samples on school premises and the display and visibility of food. The implementation of the law started in 2015 (World Cancer Research Fund, 2016).<br><br>**Hungary:** Based on Section 8 of Act XLVIII on Basic Requirements and Certain Restrictions of Commercial Advertising Activities (2008), Hungary prohibits all advertising directed at children under 18 in child welfare and child protection institutes, kindergartens, elementary schools and their dormitories. Health promotion and prevention activities in schools may only involve external organizations and consultants who are recommended by the National Institute for Health Development (World Cancer Research Fund, 2016e).<br><br>**Spain:** In 2011 the Spanish Parliament approved a Law on Nutrition and Food Safety, which stated that kindergartens and schools should be free from all advertising. Criteria for the authorisation of food promotion campaigns, nutritional education and promotion of sports or physical activity campaigns were developed jointly by the Spanish Agency for Consumer Affairs, Food Safety and Nutrition (AECOSAN) and the Regional Health Authorities and implemented in July 2015. AECOSAN and the Spanish Regional Education and Health Administrations monitor the enforcement of the law (World Cancer Research Fund, 2016)
**Context** e.g. EU action/ regulation / food industry action etc.	**Advertising Standards Authority for Ireland (ASAI) Voluntary Codes of Practice**<br><br>• In addition to complying with the provisions set out in Section 5: Promotional Marketing Practices, promotions addressed to or likely to attract children: should be carried out responsibly, taking into account the location in which the promotion is conducted (Advertising Standards Authority for Ireland, 2015)
**Evidence of implementation**	**Non-Broadcast Media Advertising and Marketing of Food and Non-Alcoholic Beverages, including Sponsorship and Retail Product Placement: Voluntary Codes of Practice (December, 2017)**<br><br>**General Rules for all Codes of Practice**<br><br>• Locations primarily used by children shall be free from all forms of marketing communication for HFSS foods. Examples of such settings

	include registered crèches, pre-schools, nurseries, family and child clinics, paediatric services, schools, dedicated school transport, playgrounds and youth centres.<br><br>**Rules for Out of Home Media**<br><br>- Displays of HFSS foods will be restricted from 100 metres of school gate for large roadside billboard formats which include but is not limited to 48 sheet sizes3 and larger for example and 60 metres for 6 sheet sizes and particular attention will be given to HFSS foods that particularly appeal to children(Healthy Ireland, 2017b).
**Comments/notes**	

**PROMO5**<br><br>**Effective policies are implemented by the government to ensure that unhealthy foods are not commercially promoted to children, including adolescents on food packages.**	
**Definitions**<br><br>**and scope**	• Includes product design and packaging (e.g. use of celebrities or cartoons, competitions and give-aways)<br>• Where the promotion is specifically in a children's setting, this should be captured in 'PROMO4'<br>• Effective means that the policies are likely to reduce overall exposure of children, including adolescents to unhealthy food advertising over the day.
**International best practice examples (benchmarks)**	Chile: through Law Number 20.606, has passed a series of regulations on the advertising of processed foods high in calories, fat, sugar, or salt. These laws specifically seek to regulate companies with brands that target children through misleading advertising and the use of cartoon mascots on commercial packaging. Chile's National Consumer Service has determined that food labels may no longer feature cartoon mascots designed to appeal to children.
**Context** e.g. EU action/ regulation / food industry action etc.	• There are no objectives specifically related to the restriction of marketing to children on food packages. However the EU recognizes in the Plan the possible impact of marketing on food packages to children.
**Evidence of implementation**	• **No further evidence found from 2016 onwards**
**Comments/notes**	

DOMAIN 4 - FOOD PRICES: Food pricing policies (e.g., taxes and subsidies) are aligned with health outcomes by helping to make the healthy eating choices the easier, cheaper choices

**PRICES1**<br>**Taxes or levies on healthy foods are minimised to encourage healthy food choices (e.g. low or no sales tax, excise, value-added or import duties on fruit and vegetables).**	
**Definitions**<br><br>**and scope**	• Includes exemptions from excise tax, ad valorem tax or import duty.<br>• Includes differential application of excise tax, ad valorem tax or import duty.<br>• Excludes subsidies (see 'PRICES3') or food purchasing welfare support (see 'PRICES4')
**International best practice examples (benchmarks)**	**Australia:** Goods and services tax (GST) exemption exists for basic foods (including fresh fruits and vegetables)(Veerman, 2013).<br><br>**Tonga:** In 2013, as part of a broader package of fiscal measures, import duties were lowered from 20% to 5% for imported fresh, tinned or frozen fish in order to increase affordability and promote healthier diets (World Cancer Research Fund, 2016f).
**Context e.g.** EU action/ regulation / food industry action etc.	**EU Action**<br><br>• There is no evidence that the EU is proposing to Member States to minimize taxes or levies on healthy foods to encourage healthy food choices. The EU has set rules on the common system of value added tax.<br><br>**COUNCIL DIRECTIVE 2006/112/EC of 28 November 2006 on the common system of value added tax**<br><br>• This Directive has laid down that Member States shall apply a standard rate of VAT, which shall be fixed by each Member State as a percentage of the taxable amount and which shall be the same for the supply of goods and for the supply of services (Article 96). However, the standard rate may not be less than 15 % (article 97) (Official Journal of the European Union, 2006).

	• Member States may apply one or two reduced rates (Article 98, 99), which may not be less than 5% only to supplies of goods or services in the categories set out in Annex III. Included in this Annex III are:<br>- foodstuffs (including beverages but excluding alcoholic beverages) for human and animal consumption; live animals, seeds, plants and ingredients normally intended for use in the preparation of foodstuffs; products normally used to supplement foodstuffs or as a substitute for foodstuffs;<br><br>These rules mean that on fruit and vegetables a minimum of 5% VAT has to be applied by the Member States.
**Evidence of implementation**	**Zero Rate**<br>• The Zero rate of VAT applies to the supply of most foodstuffs, such as bread, butter, cheese, cereals, condiments, flour, fruit, herbs, meat, milk, pasta, pastes, sauces, soup, spices, sugar, and vegetables (fresh or frozen). This list is by no means exhaustive. However, it should be particularly noted that the supply of food and drink that would normally be liable to VAT at the Zero rate becomes liable to VAT at the Second Reduced rate when it is supplied in the course of catering or by means of a vending machine (Revenue, 2019)
**Comments/notes**	

**PRICES2**<br>**Taxes or levies on unhealthy foods (e.g. sugar-sweetened beverages, foods high in nutrients of concern) are in place and increase the retail prices of these foods by at least 10% to discourage unhealthy food choices, and these taxes are reinvested to improve population health.**	
**Definitions and scope**	• Includes differential application of excise tax, ad valorem tax or import duty on high calorie foods or foods that are high in nutrients of concern
**International best practice examples (benchmarks)**	**Ireland:** On 1 May 2018, the Republic of Ireland's Sugar Sweetened Drinks Tax came into force under the Finance Act 2017 (No. 41 of 2017). The tax applies to non-alcoholic, water-based and juice based drinks which have added sugar content of 5g per 100mL and above. Drinks with over 8g of sugar per 100mL are taxed at 30 cents per litre, and drinks with between 5g and 8g of sugar per 100mL are taxed at 20 cents per litre. Fruit juices and dairy products are excluded from the tax (World Cancer Research Fund, 2018)<br><br>**UK:** In April 2018 the UK government's Soft Drinks Industry Levy came into force (as outlined in the Finance Act 2017). The Soft Drink Industry Levy applies to any pre-packaged soft drink with added sugar, containing at least 5g of total sugars per 100mL of prepared drink. Soft drinks that have a total sugar content of more than 5g and less than 8g per 100mL are taxed 0.18 British pounds ($0.25) per litre and drinks that have a total sugar content of 8g or more per 100mL are taxed 0.24 British pounds ($0.34) per litre. Milk-based drinks, milk substitute drinks, pure fruit juices, or any other drinks with no added sugar, alcohol substitute drinks, and soft drinks of a specified description which are for use for medicinal or other specified purposes are exempt from the levy. The levy applies to soft drinks produced and packaged in the UK and soft drinks imported into the UK (World Cancer Research Fund, 2018). Manufacturers had two years to prepare ahead of this tax coming into effect and over 50% of them took action to cut sugar in their products during that period (Rathbone Greenbank Investments, 2019). It was forecasted that, the tax would bring in £520 million in its first year of operation, but this was revised down to £275 million as a result of company efforts to remove sugar from their products. Data from the first full year of the tax is not yet available, but receipts from April to October 2018 totalled £154 million. It was confirmed that the Department for Education would receive the full £1 billion

	funding that had originally been expected from the sugar tax in this Parliament (Parliment UK, 2017).<br><br>**Hungary:** A "public health tax" adopted in 2012 is applied on the salt, sugar and caffeine content of various categories of ready-to-eat foods, including soft drinks, energy drinks, and pre-packaged sugar-sweetened products. The tax is applied at varying rates. Soft drinks, for example, are taxed at $0.24 per litre and other sweetened products at $0.47 per litre. The tax also applies to products high in salt, including salty snacks with >1g salt per 100g, condiments with >5g salt per 100g and flavourings >15g salt per 100g (World Cancer Research Fund, 2016f, Biro, 2015).<br><br>**Mexico:** In December 2013, the Mexican legislature passed two new taxes as part of the national strategy for the prevention of overweight, obesity and diabetes. An excise duty of 1 peso ($0.80) per litre applies to sugary drinks. This is expected to increase the price of sugary drinks by around 10%. An ad valorem excise duty of 8% applies to foods with high caloric density, defined as equal to or more than 275 calories per 100 grams. The taxes entered into force on 1 January 2014. The aim is for the revenue of taxes to be reinvested in population health, namely providing safe drinking water in schools, but there is no evidence (yet) that this is the case as the taxes are not earmarked (World Cancer Research Fund, 2016f, Colchero, 2016). In 2019, a study was conducted to estimate changes in taxed and untaxed beverages by volume of beverage purchased after the sugar-sweetened beverage (SSB) tax was introduced in 2014 (Ng et al., 2019). Results found that, The HTLU-unhealthier and HTHU groups had the largest absolute and relative reductions in taxed beverages and increased their purchases of untaxed beverages. Households with lower purchases of untaxed beverages (HTLU unhealthier and LTLU) had the largest absolute and relative increases in untaxed beverages. Furthermore, among households with higher purchases of taxed beverages, the group with lowest socio-economic status had the greatest reduction in purchases of taxed beverages (Ng et al., 2019).<br><br>**Morocco**: On 1 January 2019, Article 5 of the Finance Act 2019 came into effect increasing Morocco's value-added tax on manufactured or imported soft and non-carbonated drinks with added sugars by 50%. Carbonated or non-carbonated water, mineral water, table water or others containing <10% of edible fruit juice or juice concentrates are taxed Moroccan Dirham (MAD)

	0.45 (about $0.04) per litre; or those with >10% fruit juice or juice concentrates taxed at MAD 0.15 (about $0.016) per litre. Lemonades containing sugar with <6% lemon juice or concentrate equivalent were taxed MAD 0.45 per litre; or those containing >6% lemon juice or concentrate equivalent taxed at MAD 0.15 per litre. Unfermented carbonated or non-carbonated beverages were taxed MAD 1.24 (about $0.13) per litre. Energy drinks containing at least two stimulant ingredients such as caffeine, taurine and glucuronolactone were taxed MAD 6.00 (about $0.62) per litre (World Cancer Research Fund, 2018).<br><br>**Qatar:** In 2018, the Government of Qatar introduced Law No. (25) the 'Qatar Excise Tax Law' that came into effect on 1 January 2019. The Qatar Excise Tax Law introduced a 50% ad valorem tax on carbonated waters with added sugar, sweeteners or flavours, as well as concentrates, powders, gels or extracts intended to be made into a carbonated beverage. A tax rate of 100% is applied to beverages sold as energy drinks that contain stimulant substances (e.g. caffeine, taurine, ginseng, guarana). Carbonated non-flavoured waters, coffee and tea are excluded from the excise tax. The excise tax applies to all imported, produced or stockpiled aerated beverages (except unflavoured aerated water) and energy drinks (World Cancer Research Fund, 2018).
**Context** e.g. EU action/ regulation / food industry action etc.	**EU Action**<br><br>• There is no evidence that the EU is proposing to Member States to implement and/or increase taxes or levies on unhealthy foods to discourage unhealthy food choices. The EU does have import conditions for foods, but these are completely focused on food safety (European Comission)
**Evidence of implementation**	**Government Regulation**<br><br>• Sugar Sweetened Drinks Tax (SSDT) is effective in Ireland from 1 May 2018. SSDT applies on the first supply in the State of sugar sweetened drinks. The supplier is liable to account for and pay the tax. The tax applies to water and juice-based drinks which have added sugar and a total sugar content of five grams or more per 100 milliliters. Products liable to the tax may be in ready to consume or in concentrated form.

	• The tax operates as an excise duty and is administered on a self-assessment basis. Suppliers are required to register with Revenue in advance of making first supplies of sugar sweetened drinks in the State. They must file returns within one month after the end of the accounting period during which the supplies were made.<br><br>• Ready to consume drinks are liable to SSDT if they satisfy three criteria:<br>- They are classified within particular headings of the Combined Nomenclature (CN) codes of the European Union. The Combined Nomenclature (CN) is a tool for classifying goods, set up to meet the requirements both of Common Customs Tariff, and of the EU's external trade statistics. The CN is also used in intra-EU trade statistics. The relevant headings, CN 2009 and CN 2202, cover juices and water and or juice-based drinks.<br>- They contain added sugar.<br>- The total sugar content of the drink must be five grams or more per 100 millilitres.<br>• Examples of liable ready to consume sugar sweetened drinks include:<br>- Flavoured waters*<br>- Carbonated drinks*<br>- Energy/sports drinks*<br>- Juice based drinks*<br>*With added sugar and a total sugar content of five grams or more per 100 millilitres.<br><br>• Specific products falling under CN 2202 subheadings are excluded from liability. These include:<br>- Alcohol-free beers and wines<br>- Drinks that are based on soya, cereals, nuts or seeds or that contain milk fats<br>- Products labelled as food supplements.<br><br>• In addition, any products excluded from EU food labelling obligations on the basis of their small-scale production will not be liable to the tax.

	• Concentrated Sugar Sweetened Drinks are solid or liquid substances that require preparation to produce ready to consume drinks. Preparation involves the addition of water and, or ice and, or carbon dioxide to the concentrated substance, in accordance with manufacturers' instructions. Concentrated products are liable to SSDT if:<br>- The ready to consume drinks prepared from them have the same characteristics as drinks classified within particular headings of the CN of the EU. The relevant headings, CN 2009 and CN 2202, cover juices and water or juice-based drinks.<br>- They contain added sugar.<br>- Their total sugar content when prepared is five grams or more per 100 milliliters.<br>**Examples of concentrated Sugar Sweetened Drinks include:**<br>- Concentrated products intended for preparation at catering level to produce ready to consume drinks that are supplied directly to final consumers. Examples are post mix concentrates supplied to cinemas and restaurants.<br>- Concentrated products intended for *"home"* preparation to produce ready to consume drinks. Some examples are bottled squashes, cordials and flavoured syrups.<br>- The exclusions from scope of the tax that apply to ready to consume drinks also apply to concentrated products.<br>• SSDT applies on a volumetric basis at one of two rates, dependent on the total sugar content of the "ready to consume" form of the sugar sweetened drink. The SSDT rates are:<br>- €16.26 per hectolitre on drinks with a total sugar content of five grams or more, but less than eight grams, per 100 millilitres.<br>- €24.39 per hectolitre on drinks with a total sugar content of eight grams or more per 100 millilitres (Revenue, 2018).
**Comments/notes**	• In Step one of the *Healthy Weight for Ireland Obesity Policy and Action Plan*, the Irish Government plan to *"review the evidence, including the effectiveness of implementation, for fiscal*

	measures on products that are high in *<u>fat</u>*, *<u>sugar</u> and <u>salt</u> to reduce their consumption"* (Department of Health., 2016).

**PRICES3**<br>**The intent of existing subsidies on foods, including infrastructure funding support (e.g. research and development, supporting markets or transport systems), is to favour healthy rather than unhealthy foods.**	
**Definitions and scope**	• Includes agricultural input subsidies, such as free or subsidised costs for water, fertiliser, seeds, electricity or transport (e.g., freight) where those subsidies specifically target healthy foods.<br>• Includes programs that ensure that farmers receive a certain price for their produce to encourage increased food production or business viability.<br>• Includes grants or funding support for food producers (i.e. farmers, food manufacturers) to encourage innovation via research and development where that funding scheme specifically targets healthy food.<br>• Includes funding support for wholesale market systems that support the supply of healthy foods.<br>• Includes population level food subsidies at the consumer end (e.g. subsidising staples such as rice or bread).<br>• Excludes incentives for the establishment of, or ongoing support for, retail outlets (including greengrocers, farmers' markets, food co-ops, etc. See 'RETAIL2').<br>• Excludes subsidised training, courses or other forms of education for food producers - Excludes the redistribution of excess or second grade produce.<br>• Excludes food subsidies related to welfare support (see 'PRICES4')<br>• Should be in line with population nutrition goals related to the prevention of obesity and diet-related NCDs (e.g., reducing intake of nutrients of concern, and should not related to micronutrient deficiencies).
**International best practice examples (benchmarks)**	**Singapore:** The government, through the Health Promotion Board (HPB) increases the availability and use of healthier ingredients through the "Healthier Ingredient Scheme" (formerly part of the "Healthier Hawker" programme, launched in 2011), which provides in the first instance transitional support to oil manufacturers and importers to help them increase the sale of healthier oils to the food service industry (World Cancer Research Fund, 2016a). The Healthier Ingredient Subsidy Scheme offers a subsidy to suppliers stocking healthier items. Cooking oil is the first ingredient under the scheme, which subsidises oils with a saturated fat level of 35 per cent or lower.

**Context** e.g. EU action/ regulation / food industry action etc.	**EU Common Agricultural Policy**<br><br>• The CAP is a common policy for all the countries of the European Union. The legal basis for the common agricultural policy is established in the treaty on the functioning of the European Union.<br><br>**The CAP consists of 3 components:**<br><br>• Income support for farmers through direct payments ensures income stability, and remunerates farmers for environmentally friendly farming and delivering public goods not normally paid for by the markets, such as taking care of the countryside. Rules for direct payments to farmers have been laid down in the EU regulation 1307/2013.<br><br>- Market measures to deal with difficult market situations such as a sudden drop in demand due to a health scare, or a fall in prices as a result of a temporary oversupply on the market. Rules for these market measures have been laid down in REGULATION (EU) No 1308/2013: Common Market Organisation<br><br>• Rural development measures with national and regional programmes to address the specific needs and challenges facing rural areas. Rules for these rural development support have been laid down in REGULATION (EU) No 1305/2013 on support for rural development by the European Agricultural Fund for Rural Development (EAFRD).
**Evidence of implementation**	**Government led schemes**<br><br>**Scheme of Aid for Producer Organisations in the Fruit & Vegetables Sector**<br><br>This scheme provides EU aid to producers grouping, on a voluntary basis, to form recognised Producer Organisations (POs) and implement approved operational programmes that improve the quality and efficiency of operations and achieve the following objectives:<br><br>• Improving the competitiveness and market orientation of the sector so as to contribute to achieving sustainable production that is competitive both on the EU and external markets;<br>• Reducing fluctuations in producers' incomes resulting from crises on the market;

	• Increasing the consumption of fruit and vegetables in the Community; and<br>• Continuing the efforts made by the sector to maintain and protect the environment. The Terms and Conditions of the Scheme of EU Aid For Producer Organisations in the Fruit and Vegetables Sector, (hereinafter called "the Scheme"), as laid down by the Department of Agriculture, Food and the Marine (hereinafter referred to as 'the Department') are in accordance with the provisions of Regulation (EU) No 1308/2013 of the European Parliament and of the Council, Commission Delegated Regulation (EU) 2017/891 and Commission Implementing Regulation (EU) 2017/892 and Commission Implementing Regulation (EU) 543/2011 (Department of Agriculture Food and Marine, 2018).<br><br>**The 2019 Scheme of Investment Aid for the Development of the Commercial Horticulture Sector**.<br><br>This scheme is intended to assist in the development of the horticulture sector, including beekeeping, by grant aiding capital investments in specialised plant and equipment as well as emerging technologies specific to commercial horticulture production.<br><br>**The scheme aims to:**<br><br>• Facilitate environmentally friendly practices, promote the diversification of on-farm activities, improve the quality of products and improve working conditions. Non-production investments, which are directly associated with primary production, may also be considered. Applicants other than companies or corporate bodies must be over 18 years of age.<br>• The minimum investment which will be considered for grant aid is €10,000 excluding VAT, except in the case of beekeeping where a minimum investment of €2,000 excluding VAT applies. The upper cumulative limit, per applicant, for investments under the scheme over the period 2014-2019 is €5m. Aid for each investment is decided based on the availability of funds and the ongoing priorities for each sector within the industry as well as the quality and scale of the proposals (Department of Agriculture Food and Marine, 2019).<br><br>**Beef Data and Genomics Programme (BDGP)**

	• This will make the scheme available to beef suckler farmers who are not already members of the scheme. The scheme will be known as BDGP II and will, like the original, commit to six years of payments to farmers for completion of actions aimed at delivering accelerated genetic improvement in the national herd and improvement of its environmental sustainability.<br>• Payments to participants will be the same as BDGPI i.e. €142.50 per hectare for the first 6.66 payable hectares under the scheme, and €120 per payable hectare after that, with the same timing requirements for returning of data in order to ensure payments can go out to as many farmers as possible in December of each scheme year (Department of Agriculture Food and Marine, 2017a).
**Comments/notes**	

**PRICES4**<br>**The government ensures that food-related income support programs are for healthy foods**	
**Definitions and scope**	• Includes programs such as 'food stamps' or other schemes where individuals can utilise government-administered subsidies, vouchers, tokens or discounts in retail settings for specific food purchasing.<br>• Excludes general programs that seek to address food insecurity such as government support for, or partnerships with, organisations that provide free or subsidised meals (including school breakfast programs) or food parcels or redistribute second grade produce for this purpose.<br>• Excludes food subsidies at the consumer end (e.g. subsidising staples at a population level – see 'PRICES3').
**International best practice examples (benchmarks)**	**USA:** In 2009, the U.S. Department of Agriculture's implemented revisions to the Special Supplemental Nutrition Program for Women, Infants, and Children (WIC) to improve the composition and quantities of WIC-provided foods from a health perspective. The revisions include: Increase the dollar amount for purchases of fruits and vegetables, expand whole-grain options, allow for yoghurt as a partial milk substitute, allow parents of older infants to buy fresh produce instead of jarred infant food and give states and local WIC agencies more flexibility in meeting the nutritional and cultural needs of WIC participants (World Cancer Research Fund, 2016f).<br><br>**USA:** In 2012, the USDA piloted a "Healthy Incentives Pilot" as part of the Supplemental Nutrition Assistance Program (SNAP, formerly "food stamps"). Participants received an incentive of 30 cents per US$ spent on targeted fruit and vegetables (transferred back onto their SNAP card). The Pilot included 7500 individuals (World Cancer Research Fund, 2016f). In New York City and Philadelphia, "Health Bucks" are distributed to farmer's markets. When customers use income support (e.g. Food Stamps) to purchase food at farmer's markets, they receive one Health Buck worth 2USD for each 5USD spent, which can then be used to purchase fresh fruit and vegetable products at a farmer's market (World Cancer Research Fund, 2016f). In Philadelphia, the programme has been expanded to other retail settings like supermarkets and corner store.<br><br>**UK:** The British Healthy Start programme provides pregnant women and/or families with children under the age of four with weekly vouchers to spend on foods including milk, plain yoghurt, and fresh and frozen fruit and

	vegetables. Participants or their family must be receiving income support/jobseekers' allowance or child tax credits. Pregnant women under the age of 18 can also apply. Full national implementation of the programme began in 2006 (World Cancer Research Fund, 2016f).
**Context** e.g. EU action/ regulation / food industry action etc.	**EU Action**<br><br>• In 2014 the Fund for European Aid to the Most Deprived (FEAD) was adopted as the successor programme to the former programme for the most deprived persons (MDP) (Auditors, 2019). It provides €3.8 billion of EU funding for the programme period 2014-2020 and it is implemented at national level through operational programmes. EU countries are to contribute at least 15% in national co-financing **to their national p**rogramme (European Comission, 2019a)<br><br>• However, despite these changes, the European Court of Auditors found that FEAD remains essentially a food support programme, as 83% of the Fund is devoted to food support.<br><br>• In May 2018, the European Commission adopted a legislative proposal for a new European Social Fund Plus (ESF+) Programme, based on the proposed Multiannual Financial Framework for the period 2021-2027. It will serve as the EU's main financial instrument guiding investment in people and implementation of the European Pillar of Social Rights, including health policies (European Comission, 2019a).<br><br>The ESF+ Programme merges existing funds and programmes including:<br><br>• The European Social Fund (ESF) and the Youth Employment Initiative (YEI)<br>• The Fund for European Aid to the Most Deprived (FEAD)<br>• The Employment and Social Innovation (EaSI) programme<br>• The Health Programme.<br><br>ESF+ Programme financing will focus on three main strands:<br><br>• The first covers the (ex-) ESF and basic material assistance to the most deprived people

	• The second will cover initiatives promoting employment and social innovation (EaSI)<br>• The third covers initiatives aiming at preventing health risks and promoting public health.<br><br>The new ESF+ has a strong **health dimension:** health policies will be funded both through dedicated funding (strand within the ESF+) as well as across other key financial instruments. ESF+ Programme specifically includes **€ 413 million for the Health strand**. The new architecture of ESF+ will not only preserve a specific health strand, but will also support the integration of health in other related policies and the coordination between complementary health-related budget lines.<br><br>**Charity sector – St Vincent De Paul**<br><br>• The St Vincent De Paul spends almost €10m annually, giving families and individuals in Ireland the ability to put food on the table. Families and individuals can use these vouchers to purchase food in stores such as, Dunnes Stores, Aldi. Lidl and Tesco. (Byrne. C, 2012)
**Evidence of implementation**	• **No evidence found from 2016 onwards**
**Comments/notes**	

DOMAIN 5 - FOOD PROVISON: The government ensures that there are healthy food service policies implemented in government-funded settings to ensure that food provision encourages healthy food choices, and the government actively encourages and supports private companies to implement similar.

**PROV1**<br>**The government ensures that there are clear, consistent policies (including nutrition standards) implemented in schools and early childhood education services for food service activities (canteens, food at events, fundraising, promotions, vending machines etc.) to provide and promote healthy food choices.**	
**Definitions and scope**	• Includes early childhood education and care services (0-5 years).<br>• Schools include government and non-government primary and secondary schools (up to age 18 years)<br>• Includes policies and nutrition standards to provide and promote healthy food choices or to limit or restrict the provision or promotion of unhealthy food choices<br>• Includes policies that relate to school meals programs, where the program is partly or fully funded, managed or overseen by the government<br>• Excludes programmes in schools that are targeted to children of low socioeconomic groups only (as these would be covered under PRICES4)
**International best practice examples (benchmarks)**	**Ireland:** The School Meals (Local Projects) Scheme, is an administrative scheme, operated directly by the Department of Employment Affairs and Social Protection (Healthy Ireland, 2017a). The Scheme provides funding to primary and post-primary schools, local groups, voluntary organisations and community-based not-for-profit preschools operating their own school meals projects. The 'Nutrition Standards for School Meals', are being implemented under this scheme and aim to ensure that children and young people in schools participating in the scheme are provided with healthy balanced meals that follow the Healthy Eating Guidelines. These Nutrition Standards are food-based, and are provided for each meal type funded by the Scheme, that is: Breakfast or snack Lunch or after-school meal Dinner, only healthy food choices that meet the standards will be funded. The Standards will also be used by those administering the Scheme in the schools, commencing in January 2018, to ensure that food purchased

	complies with the Nutrition Standards when food contracts are being specified in the procurement process, and should also be applied when planning menus (Healthy Ireland, 2017a).<br><br>**Jamaica:** In November 2018, the Ministry of Health published mandatory nutrient guidelines for beverages sold/served within all public educational institutions for children (i.e. early childhood, primary level and secondary level). The guidelines prohibit sweetened beverages that exceed a maximum sugar concentration of: 6g/100ml (effective 1 January 2019); 5g/100ml (effective 1 January 2020); 4g/100ml (effective 1 January 2021); and 2.5g/100ml (effective 1 January 2023). All unsweetened beverages are permitted The guidelines also caution against beverages containing >10mg/serve of caffeine, discourage the use of artificial sweeteners and recommend beverage portions sold/served of <12 ounces (not including water).<br><br>**Chile:** In 2012, the Chilean government approved a Law of Nutritional Composition of Food and Advertising [51]. In June 2015, the Chilean authority approved the regulatory norms required for the law's implementation. The regulatory norms define limits for calories, saturated fat, sugar and sodium content considered 'high' in foods and beverages. The law prohibits the sale of foods in the 'high in' category in schools. The law came into effect on 27 June, 2016. In 2019, a study conducted on the impact of this law funds that, foods exceeding any cut-offs decreased from 90.4% in 2014 to 15.0% in 2016. Solid products had a substantial reduction in calories, sugar, saturated fat, and sodium. Liquid products had a reduction in calories, total sugar, and saturated fat, whereas sodium increased. This was a result of changes in product mix (Massri et al., 2019).<br><br>**Finland**: In 2008, the National Nutrition Council approved nutrition recommendations for school meals. These include food and nutrient recommendations for salt, fibre, fat, starch, fat and salt maximums for meat and processed meat, and drinks. There are also criteria for snacks provided in schools. New recommendations on Eating and learning together - recommendations for school meals have been published in 2017 (National Nutrition Council, 2017). In 2018, the early childhood education: Health and joy from food - meal recommendations for early childhood education and care, were published (National Nutrition Council, 2018). The 2018 published

	recommendations for families with children: Eating together - food recommendations for families with children, were updated in 2019 (National Institute for Health and Welfare, 2019). Additionally, Finland published its first nutrition recommendations for upper secondary schools and vocational schools.<br><br>**UK:** England, Scotland, Wales and Northern Ireland have mandatory nutritional standards for school food, which also apply to food provided in schools other than school lunches. These standards apply to most state schools (with the exception of around 4,000 academies established between September 2010 and June 2014, which are exempt) and restrict foods high in fat, salt and sugar, as well as low quality reformed or reconstituted foods.<br><br>**Brazil:** The national school feeding programme (Fundo Nacional de Desinvolvimento da Educacao, 2016) mandates a weekly minimum of fruits and vegetables, regulates sodium content and restricts the availability of sweets in school meals. A school food procurement law (Ministry of Education, 2016), approved in 2001, limits the amount of processed foods purchased by schools to 30%, and bans the procurement of drinks with low nutritional value, such as sugary drinks. The law requires schools to buy locally grown or manufactured products, supporting small farmers and stimulating the local economy. Resolution no 38 (16 July 2009) sets food- and nutrition-based standards for the foods available in the national school meal programme (Law 11.947/2009). Article 17 prohibits drinks of low nutritional value (e.g. soda), canned meats, confectionary and processed foods with a sodium and saturated fat content higher than a specified threshold.<br><br>**Costa Rica:** Executive Decree No 36910-MEP-S (2012) of the Costa Rican Ministries of Health and Education sets restrictions on products sold to students in elementary and high schools, including food with high levels of fats, sugars and salt, such as chips, cookies, candy and carbonated sodas. Schools are only permitted to sell food and beverages that meet specific nutritional criteria. The restrictions were upheld by the Constitutional Court in 2012 following a challenge by the food industry (World Cancer Research Fund, 2016d).

	**Hungary**: Since 2012, food and beverages subject to the public health product tax may not be sold on school premises or at events organized for school children, including out of school events based on the Ministerial Decree 20/2012 (VIII.31) on the Operation of Public Education Institutions and the of Names of Public Education Institutions. Section 130(2) of the Decree requires the head of the educational institution to consult the school health service prior to entering into agreements with vending machine operators or food vending businesses. The school health service verifies whether the products to be sold meet the nutritional guidelines set by the National Institute of Pharmacy and Nutrition. Products that do not comply are prohibited (World Cancer Research Fund, 2016d).<br><br>**Uruguay**: In September 2013, the government of Uruguay adopted Law No 19.140 on 'healthy eating in schools. It mandated the Ministry of Health to develop standards for food available in canteens and kiosks in schools, prohibited advertising for these same foods and restricted the availability of saltshakers. The school food standards were elaborated in March 2014 and aimed to promote foods with natural nutritional value with a minimum degree of processing and to limit the intake of free sugars, saturated fat, trans fat and sodium. Limits are set per 100g of food, 100ml for drinks and also per 50g portion. This was implemented in public schools in 2015 (World Cancer Research Fund, 2016d, Fundo Nacional de Desinvolvimento da Educacao, 2016).
**Context** e.g. EU action/ regulation / food industry action etc.	<u>**EU Policy**</u><br><br>**EU School Scheme**<br><br>• On 1 August 2017, the School Fruit and Vegetables Scheme (SFVS) merged with the School Milk Scheme (SMS) under a single EU financial and legal framework. Previously operating as separate schemes for milk, fruit and vegetables, the new merged scheme, like the individual schemes it replaced, is designed to help promote the benefits of healthy eating to children and encourage them to increase their consumption of fruit, vegetables and milk (Department of Agriculture Food and Marine, 2017b).<br><br>**EU school fruit, vegetables and milk scheme**

	• The merger followed recommendations put forward by the European Court of Auditors and other external evaluations for improvements in the set-up and functioning of the school scheme. The rationale behind the recommendations was that the combination of the SFVS and the SMS under a joint new financial and legal framework would simplify the rules, reduce the administrative burden and adopt a more targeted approach.<br><br>**The new Scheme operates under a legal framework as follows:**<br>• **Basic Regulation**: Regulation (EU) 2016/791 of the European Parliament and of the Council of 11 May 2016 amending Regulations (EU) No 1308/2013 and (EU) No 1306/2013 as regards the aid scheme for the supply of fruit and vegetables, bananas and milk in educational establishments<br>• **Financial**: Council Regulation (EU) 2016/795 amending Regulation (EU) No 1370/2013 determining measures on fixing certain aids and refunds related to the common organisation of the markets in agricultural product.<br>• **Detailed technical rules**: Commission Delegated Regulation (EU) 2017/40 of 3 November 2016 supplementing Regulation (EU) No 1308/2013 of the European Parliament and of the Council with regard to Union aid for the supply of fruit and vegetables, bananas and milk in educational establishments and amending Commission Delegated Regulation (EU) No 907/2014.<br>• **Implementing Regulation**: Commission Implementing Regulation (EU) 2017/39 of 03 November 2016 on rules for the application of Regulation (EU) No 1308/2013 of the European Parliament and of the Council with regard to Union aid for the supply of fruit and vegetables, bananas and milk in educational establishments<br>• The EU School Scheme is funded through the European Union's Common Agricultural Policy (CAP), and has an overall combined annual budget of €250 million. Ireland is entitled to an annual draw down, subject to satisfying the relevant EU Regulations of €1.75 m for the SFVS and €0.9m for the SMS. National funding is also made available on an annual basis to run both the SFVS and the SMS in Ireland

**Evidence of implementation**	**Department of Employment Affairs and Social Protection – Hot School Meals Pilot Project (2019)**<br><br>• As part of Budget 2019, Minister Doherty announced that hot dinners will be provided on a pilot basis in up to 36 schools to some 7,200 children from September 2019 at a cost of €1m for 2019 and €2.5m in a full year. The pilot will initially be targeted at Primary Schools which do not currently avail of the dinner option under the Department of Employment Affairs and Social Protection's School Meals Programme and do not have canteen/kitchen facilities for preparation of the hot dinners on site.<br><br>• On Monday, 28 January, 2019: Minister for Employment Affairs and Social Protection, Regina Doherty, T.D. launched a Hot School Meals scheme for Our Lady of Lourdes Primary School in Golden bridge, Inchicore. This is the first Hot School Meals scheme to be introduced and it will serve to road-test the idea and identify any issues before a larger pilot initiative is rolled out later this year. During this initial pilot, almost 250 pupils attending this school will receive a hot meal at lunchtime each day. It will run until the end of the current school year (end June)(Department of Employment Affairs and Social Protection, 2019)<br><br>**'Healthy Weight for Ireland': Obesity Policy and Action Plan 2016-2025'.**<br><br>● Action 1.1 in Step 1 of the 'A *Healthy Weight for Ireland Obesity Policy and Action Plan 2016 – 2025 (OPAP) states; "as an integral part of the 'healthy school' concept, develop and implement a 'whole of school' healthy lifestyle programme (including but not limited to, the curriculum, on nutrition, physical activity, smoking, alcohol and mental wellbeing), incorporating knowledge, skills and greater understanding of environmental and factors that influence children and young people. National food standards for primary schools will be developed"*(Department of Health, 2016c).<br><br>● Action 1.6 in the *OPAP* sets also out to *"examine the expansion of current effective programmes, such as Food Dudes and The Incredible Edibles and develop further opportunities for collaboration with other government departments and state*

	agencies in the promotion of fresh produce and its role in a healthy, balanced diet". The aim of the incredible edibles is to communicate the importance of food origin and eating a healthy diet. The Department of Education and Skills and the Department of Health and Children, through the Healthy Ireland framework, have joined Agri Aware's incredible Edibles patrons along with the Department of Agriculture, Food and Marine, Bord Bia and the horticulture industry to support this important healthy eating educational programme for primary level students (Agri Aware's Incredible Edibles).<br><br>● Action 1.4 in the *Obesity Policy and Action Plan* also recommends providing potable water in all learning centres (from preschool and crèches to universities and adult learning centres) and ensure all new buildings provide potable water on opening. Child care regulations in 2006 by DCYA state that potable drinking water should be provided to children at all times, however there is no monitoring yet or evidence that progress has been made (Department of Health, 2016c).<br><br>● Eat Smart Move More was launched by the HSE in 2017. The booklet provides guidelines for healthy eating and physical activity for children. It provides ideas on ways to make exercising more fun and enjoyable and provides a variety of different meal and snack ideas. The initiative is set for review in 2020 (Health Service Executive, 2017a)<br><br>**Nutrition Standards for School Meals (2017)**<br><br>● In 2017, the new Nutrition Standards for School Meals were launched by the Minister for Health, the Minister for Education and Skills and the Minister for Employment Affairs and Social Protection. These Nutrition Standards are adapted from the Standards published to support the Food in Schools Policy in Northern Ireland. This work was coordinated by the Health and Wellbeing Unit, Department of Health and undertaken by a working group with representatives from safefood and the Healthy Eating Active Living

	(HEAL) programme in the Health Service Executive, in cooperation with members of the School Meals Programme in the Department of Employment Affairs and Social Protection and the Department of Education and Skills (Healthy Ireland, 2017a).<br><br>● The School Meals (Local Projects) Scheme, is an administrative scheme, operated directly by the Department of Employment Affairs and Social Protection (Healthy Ireland, 2017a). The Scheme provides funding to primary and post-primary schools, local groups, voluntary organisations and community-based not-for-profit preschools operating their own school meals projects. The 'Nutrition Standards for School Meals', are being implemented under this scheme and aim to ensure that children and young people in schools participating in the scheme are provided with healthy balanced meals that follow the Healthy Eating Guidelines.<br><br>● These Nutrition Standards are food-based, and are provided for each meal type funded by the Scheme, that is: Breakfast or snack Lunch or after-school meal Dinner, only healthy food choices that meet the standards will be funded. The Standards will also be used by those administering the Scheme in the schools, commencing in January 2018, to ensure that food purchased complies with the Nutrition Standards when food contracts are being specified in the procurement process, and should also be applied when planning menus (Healthy Ireland, 2017a)

Table 7: Snacks from the JUICY, THIRSTY, SMOOTH, CRUNCHY and CHEWY snack choices. DOH.

TABLE 2 - SNACKS

Juicy Snacks	Thirsty Snacks	Smooth Snacks	Crunchy Snacks	Chewy Snacks
Orange<br><br>Pineapple chunks<br><br>Plum<br><br>Pear<br><br>Tomato<br><br>Seedless grapes	Milk<br><br>Home-made soup<br><br>Check Drinks' *TABLE 4+5, (pages 19 + 20)*	Banana<br><br>Yogurt – natural or fruit<br><br>Milk pudding<br><br>Home-made milkshake using yogurt, milk and fruit	Raw vegetable slices, sticks or wedges – try them with yogurt dip<br><br>Apples<br><br>Toast<br><br>Breakfast cereal (without sugar, honey or chocolate coating)<br><br>Crackers (without salt on top)	Bread - rolls, baps, pitta, baguettes<br><br>Scones – plain, fruit or wholemeal<br><br>Cheese slices, cubes or strings<br><br>Cold meat slices

The Nurture Programme (2016)

- The Nurture Programme – Infant Health and Wellbeing, launched on the 23rd May 2016, is a partnership between the Health Service Executive, Atlantic Philanthropies, Katharine Howard Foundation and Centre for Effective Services.

- It is a programme of work which builds on the existing child health programme, strengthening a cohesive, integrated child health and wellbeing service for children aged 0-2 years and their families. The aim of the programme is to support parents and healthcare professionals in their caring and service provisions. (Health Service Executive, 2016b).

- In December 2018, Minister for Health Simon Harris TD launched new HSE resources for parents on December 6th including the mychild.ie website. This is a new website for parents which contains information and advice on pregnancy and the first 3 years of your child's life. The website is based on the most-up-date information available within the health service on pregnancy, parenting and child health. It was developed in conjunction with a wide range of health care professional including midwives, public health nurses,

<table>
<tr><td></td><td>doctors, psychologists and dietitians. In addition, the website also focuses on the experience and knowledge of parents themselves, with over 4,000 parents across Ireland involved in the design of mychild.ie. (Health Services Executive, 2018).

Childcare Regulations (2016)

● Under the Childcare Regulations 2016, pre-school providers must ensure that children are given regular drinks and food in adequate quantities for their needs, in consultation with parents where concerns exist (Tusla, 2018b). The food and drink supplied should be nutritious, varied and should take account of the Food and Nutrition Guideline for Pre-School Services as prepared by the Department of Health and Children. The guidelines recommend the following:

● Children in day care for more than 5 hours per session (full day care); Offer at least two meals (one hot) and two snacks. Snacks such as fruit, raw vegetables, or plain crackers are good, but sugary snacks are to be avoided. For example- breakfast, snack, lunch and snack. An evening meal may be provided for children staying for a longer day.

● Children in day care for a maximum of 5 hours per session (part-time day care); Offer at least two meals and one snack, for example-breakfast, snack, and lunch.

● Children in day care for up to 3.5 hours per session (sessional pre-school service); Offer one meal and one snack, for example; snack and lunch or breakfast and snack. Good snacks include raw vegetables, fruits, a glass of milk and plain crackers. Sugary snacks are to be avoided.</td></tr>
<tr><td>Comments/notes</td><td></td></tr>
</table>

**PROV2**<br>**The government ensures that there are clear, consistent policies in other public sector settings for food service activities (canteens, food at events, fundraising, promotions, vending machines, public procurement standards etc.) to provide and promote healthy food choices**.	
**Definitions and scope**	➢ Public sector settings include: - Government-funded or managed services where the government is responsible for the provision of food, including public hospitals and other in-patient health services (acute and sub-acute, including mental health services), residential care homes, aged and disability care settings, custodial care facilities, prisons and home/community care services - Government-owned, funded or managed services where the general public purchase foods including health services, parks, sporting and leisure facilities, community events etc. - Public sector workplaces<br>➢ Includes private businesses that are under contract by the government to provide food<br>➢ Excludes 'public settings' such as train stations, venues, facilities or events that are not funded or managed by the government (see 'RETAIL4')<br>➢ Excludes school and early childhood settings (see 'PROV1')<br>➢ Includes policies and nutrition standards to provide and promote healthy food choices or to limit or restrict the provision or promotion of unhealthy food choices<br>➢ Includes the strategic placement of foods and beverages in cabinets, fridges, on shelves or near the cashier.<br>➢ Includes the use of signage to highlight healthy options or endorsements (such as traffic lights or a recognised healthy symbol)<br>➢ Includes modifying ingredients to make foods and drinks healthier, or changing the menu to offer healthier options<br>➢ Excludes public procurement standards (see 'PROV3')
**International best practice examples (benchmarks)**	**Latvia:** In 2012, the government set salt levels for all foods served in hospitals and long-term social care institutions. Levels may not exceed 1.25g of salt/100g; fish products may contain up to 1.5g salt/100g (World Cancer Research Fund, 2016d).<br><br>**Ireland:** The HSE Vending Policy 2019 replaces the 2014 Healthier Vending Policy and applies to all vending machines that stock cold soft drinks, confectionery and snacks on HSE premises & premises funded by the HSE. Sugar sweetened beverages will not be stocked in vending machines, 50% of beverages stocked will be still water and the remaining beverages stocked will include non-sugar

	sweetened beverages e.g. diet drinks, juices, flavoured and sparkling water. Snacks containing more than 200 calories per packet will not be stocked in machines. An exception to this is 3 packets of dried fruits, nuts or seeds (plain and unsalted). Products will be clearly labelled with the number of calories per product related fields (Health Service Executive, 2019).<br><br>**Bermuda:** In 2008, the Government Vending Machine Policy was implemented in government offices and facilities to ensure access to healthy snacks and beverages for staff. The policy requires that all food and beverages in vending machines on government premises meet specific criteria based on levels of total fat, saturated fat, trans fat, sodium and sugar. Criteria exclude nuts and 100% fruit juices (World Cancer Research Fund, 2016d).<br><br>**New York**: New York City's Food Standards (enacted with Executive Order 122 of 2008) set nutritional standards for all food purchased or served by city agencies, which applies to prisons, hospitals and senior care centres. The Standards include: maximum and minimum levels of nutrients per serving; standards on specific food items (e.g. only no-fat or 1% fat milk); portion size requirements; the requirement that water be offered with food; a prohibition on the deep-frying of foods; and daily calorie and nutrient targets, including population-specific guidelines (e.g. children, seniors) (World Cancer Research Fund, 2016d, Lederer, 2014). As of 2015, 11 city agencies are subject to the NYC Food Standards, serving and selling almost 250 million meals a year. The Food Policy Coordinator has the responsibility of ensuring adherence with the Food Standards. Self-reported compliance with the standard is 96%.<br><br>**Wales:** Vending machines are prohibited in National Health Service Hospitals. The government issued a guidance defining what is allowed and not and has liaised with major vending providers to find ways to introduce healthier food options (Health Promoting Hospital Vending Directions and Guide 2008).<br><br>**The Netherlands:** The Netherlands Nutrition Centre introduced the 'Guidelines for Healthier Canteens', which can be applied in canteens at schools, sports clubs and workplaces to make them more healthy. The Guidelines for Healthier Canteens cover canteens at product level and at the level of the full range of food and drink being offered, together with the canteen's general display layout. The

	framework of the Guidelines for Healthier Canteens defines three different levels: bronze, silver and gold (Netherlands Nutrition Centre, 2017).
**Context** e.g. EU action/ regulation / food industry action etc.	**The Irish Heart Foundation**<br>• An Bord Bia recognizes Galway University Hospitals' commitment from the Aramark catering team toward offering healthier, more balanced foods, and the attainment of the Irish Heart Healthy Eating GOLD Award. In order to achieve the GOLD standard, hospitals must display calorie counts on menus, offer low fat and high fibre options and offer a larger selection of fruits and vegetables. Additional criteria, such as 50% of beverage offerings being healthier options (i.e. water) is also required.<br>**Happy Heart Healthy Eating award**<br>• The Happy Heart Healthy Eating award set up by the Irish heart foundation aims to create a culture of health and wellbeing and provide long-term sustainable programmes (Irish Heart Foundation). There are three different levels of achievement for the award: bronze, silver, and gold. There are many benefits for employees such as having better healthy meal options and obtaining a healthier work environment.<br>• The objective of the Healthy Eating Award programme is to assist a staff restaurant to adopt healthier cooking practices and provide healthier food choices, without incurring substantial cost. Recommendations given to the catering manager meet the Irish Heart Foundation and the Department of Health's Healthy Eating Guidelines.<br>• The new Silver and Gold criteria reflect all areas of healthy eating recommendations, covering fat, fibre, fruit and vegetables, salt and sugar and focuses on the areas that give employees the best impact for a healthier diet. Examples of Silver criteria are that oily fish is offered at least twice a week, at least two days per week are chip-free, and that calories are displayed on the hot lunch menu. Examples of Gold criteria are that fish is offered at least 50% of the week on the hot and/or cold menu, at least three days per week are chip-free, and that calories are displayed on all menu options.<br>**Schools Healthy Eating Award programme**

	● The schools Healthy Eating Award programme is helping to set and facilitate a nationwide School Catering Standard for post primary schools – for those who wish to promote and offer healthy food choices for their children and staff. Schools Healthy Eating awards have been developed as well as healthy schools guidelines to be followed (Irish Heart Foundation).<br><br>**HIQA The Review of Nutrition and Hydration Care in Public Acute Hospitals**<br><br>● In 2016, HIQA published a report titled; 'The Review of Nutrition and Hydration Care in Public Acute Hospitals'. The report from HIQA identified 4 main elements for improvement which, if successfully integrated across all public acute hospitals, could significantly improve nutritional and hydration patient care. They include:<br><br>● All hospitals should have a nutrition steering committee in place.<br>● All patients admitted to hospital should be screened for the risk of malnutrition.<br>● Hospitals must audit compliance with all aspects of patients' nutritional care and share the findings with all relevant staff groups involved in food service and patient care.<br>● Hospitals should strive to improve patients' experience with hospital food and drink by engaging with patients about food variety and choice.<br>● HIQA will continue to monitor public acute hospitals compliance to adequately assessing, monitoring and evaluating patients' nutritional and hydration needs. They will monitor these needs by carrying out unannounced hospital inspections and using the patient self-assessment tool (Health Information Quality Authority, 2016).<br><br>**Health Services Executive**<br><br>The HSE also identified two strategic priority actions in the National Service Plan for 2016 in relation to improving food and nutrition in hospitals:<br><br>● The development of a hospital food and nutrition policy, to be accompanied by an implementation plan. This plan has been finalised and is currently awaiting a date for the Minister for Health to launch it.<br>● A quality improvement programme in relation to nutrition and hydration which will be delivered across services, including acute hospital services (Health Service Executive, 2016a)

Evidence of implementation	**Healthy Weight for Ireland Obesity Policy and Action Plan**<br><br>- In Step 6 of the Healthy Weight for Ireland Obesity Policy and Action Plan, the Irish Government plan to "review and improve the quality of food in hospitals and develop a food and nutrition policy for hospitals". The key leads responsible for this review are the HSE and HIQA (Department of Health, 2016c)<br><br>**The HSE Food, Nutrition and Hydration Policy for Acute Hospitals was launched in 2019.**<br><br>- The purpose of this Policy is to provide a national framework for food, nutritional care and hydration provision for adult patients in acute hospitals.<br>- The Policy will apply to all staff involved in the provision and delivery of food, fluids and nutritional care for patients. All staff involved in the provision of nutritional care should use the Policy recommendations to review and develop services. Staff refers to clinical (medical, nursing, health care assistants, health and social care professionals) and non-clinical staff including catering services, catering support services and hospital managers. All locations in acute hospitals are included, Inpatients, Emergency Department and Day Procedures Units.<br><br>**The objectives of this Policy are to:**<br><br>- 1. Improve the quality and safety of food and nutritional care in acute hospitals.<br>- 2. Ensure that key areas of improvement recommended by the Health Information and Quality Authority are addressed.<br>- 3. To improve patient experience.<br>- 4. To support recommendations from the National Clinical Guideline (NCG): Nutrition Screening and Use of Oral Nutrition Support for Adults in the Acute Care Setting (Health Service Executive, 2018).<br><br>**The HSE Vending Policy 2019**<br><br>The HSE Vending Policy 2019 replaces the 2014 Healthier Vending Policy and applies to all vending machines that stick cold soft drinks, confectionery and snacks on HSE premises & premises funded by the HSE. It was developed in 2018 by the HSE Healthy Eating Active Living Programme and was informed by the HSE Healthier Vending Policy Assessment of Compliance Report 2018. A technical sub

	group was established to consider the nutrient profile of vending machines (Health Service Executive, 2019).<br><br>**Beverages:** Sugar sweetened beverages will not be stocked in vending machines, 50% of beverages stocked will be still water and remaining beverages stocked will include non-sugar sweetened beverages e.g. diet drinks, juices, flavoured and sparkling water.<br><br>**Snacks:** Snacks containing more than 200 calories per packet will not be stocked in machines and an exception to this is 3 packets of dried fruits, nuts or seeds (plain and unsalted)<br><br>**Calorie posting:** Products will be clearly labelled with the number of calories per product
**Comments/notes**	

**PROV3**<br>**The government ensures that there are clear, consistent public procurement standards in public sector settings for food service activities to provide and promote healthy food choices.**	
**Definitions and scope**	• Includes standards for the public sector which encourage the procurement of healthy foods.<br>• Includes standards for the public sector which discourage the procurement of unhealthy foods.<br>• Includes public sector settings as defined in PROV 1 and PROV 2
**International best practice examples (benchmarks)**	**Brazil:** A school food procurement law (Ministry of Education, 2016), approved in 2001, limits the amount of processed foods purchased by schools to 30%, and bans the procurement of drinks with low nutritional value, such as sugary drinks. The law requires schools to buy locally grown or manufactured products, supporting small farmers and stimulating the local economy<br><br>**UK:** The UK Government Buying Standard for Food and Catering Services (GBSF of 2014, updated March 2015, sets out standards for the public sector when buying food and catering services. It is supported by the Plan for Public Procurement: Food and Catering Services (2014). The nutrition requirements must be followed by schools, hospitals, care homes, communities and the armed forces. To improve diets, the GBSF sets maximum levels for sugar in cereals and generally for saturated fat and salt, in addition to minimum content of fibre in cereals and fruit in desserts. (World Cancer Research Fund, 2016e).
**Context** e.g. EU action/ regulation / food industry action etc.	**Examples of EU action**<br><br>• The EU GPP comprehensive criteria set higher targets for organic content and packaging, and also cover integrated production accreditations for multiple food categories (Environmental Protection Agency, 2014). The EU GPP criteria for food and catering services focus on the purchase of food from producers, wholesalers and plants, although predominantly through third party distribution companies and out sourced catering service providers.
**Evidence of implementation**	• In Ireland there is no healthy food procurement policy in place for the public sector. A public sector healthy procurement policy would aim to encourage public bodies to serve food that meets objectives on issues such

	as health, nutrition and waste (National Nutrition Surveillance Centre, 2009)
**Comments/notes**	

**PROV4**<br>**The Government ensures that there are good support and training systems to help schools and other public sector organisations and their caterers meet the healthy food service policies and guidelines**	
**Definitions and scope**	• Includes support for early childhood education services as defined in 'PROV1'<br>• Public sector organisations include settings defined in 'PROV2'<br>• Support and training systems include guidelines, toolkits, templates (e.g. policy/guidelines or contracts), recipes and menu planning tools, expert advice, menu and product assessments, online training modules, cook/caterer/other food service staff information and training workshops or courses
**International best practice examples (benchmarks)**	**Australia:** The Healthy Eating Advisory Service supports settings such as childcare centers, schools, workplaces, health services, food outlets, parks and sporting centres to provide healthy foods and drinks to the public in line with Victorian Government policies and guidelines. The Healthy Eating Advisory Service is delivered by experienced nutritionists and dieticians at Nutrition Australia Victorian Division. The support includes training cooks, chefs, foods service and other key staff, discovering healthier recipes, food ideas and other helpful resources to provide healthier menus and products (Healthy Eating Advisory Service, 2017).<br><br>**Singapore:** The National Workplace Health Promotion Programme, launched in Singapore in 2000, is run by the Health Promotion Board. Both private and public institutions are encouraged to improve the workplace environment by providing tools and grants. Grants are awarded to help companies start and sustain health promotion programmes. Tools include a sample Healthy Workplace Nutrition Policy, a sample Healthy Workplace Catering Policy, and a detailed Essential Guide to Workplace Health, setting out ways to transform the workplace into a health-supporting work environment (World Cancer Research Fund, 2016d).<br><br>**Netherlands:** The Healthy School Canteen Brigade (https://gezondeschoolkantine.voedingscentrum.nl/nl.aspx) is a team consisting of dieticians and health scientists of the Dutch Nutrition Center to help school realize healthy canteens. They visit schools in the Netherlands and give them advice. The Dutch Nutrition Center also developed the canteen scan (https://gezondeschoolkantine.voedingscentrum.nl/nl/stap-voor-

	stap/kantinescan.aspx), a tool to check the level of healthiness of canteens and which gives practical advises.
**Context** e.g. EU action/ regulation / food industry action etc.	**Industry**<br><br>**Food Choice at work**<br><br>● Food Choice at Work is a spin-out company from University College Cork, Ireland that has developed a Leading Edge Healthy Eating Management System for employees. Resting on over 6 years of scientific research led by Dr Fiona Geaney and Professor Ivan Perry. It re-engineer's workplace eating environments through tailored menu modifications, portion size guidance, daily application of our calorie and traffic light coding system, catering training, monthly nutrition education and health check and nutrition clinics. Food Choice at Work deals directly with caterers, workplace stakeholders and employees to make fundamental positive changes in food purchasing, preparation and presentation. Their data-driven programme is tailored to the culture and climate of each individual workplace. Clients include, Microsoft, KSG, UCC, Tyndall national institute and Cork City Council (Food Choice at Work, 2019).<br><br>**Safefood and Early Childhood Ireland**<br><br>● Have collaborated to develop Little bites, an online resource that provides information on food safety, food allergen and healthy eating advice for all early childcare providers (safefood, 2016) With regards to healthy eating it provides information on:<br>- Food and nutrition guidelines<br>- Healthy eating policies<br>- Child care regulations<br>- Menu plans and recipes<br>- Serving size guide<br>- Fussy eating<br>- Healthy snacks and food swaps<br>- Special occasions<br><br>**Safefood**<br><br>● In pre-school, primary and post-primary schools in the republic of Ireland, safefood provides teacher-led curriculum based educational resources for children and young people on healthy eating and food

	safety. These resources are free to schools on the island of Ireland and are designed to be interactive. Each is suitable for a particular age-group or key stage (safefood, 2016)<br><br>**Schools Healthy Eating Award programme**<br><br>● The schools Healthy Eating Award programme is helping to set and facilitate a nationwide School Catering Standard for post primary schools – for those who wish to promote and offer healthy food choices for their children and staff. Schools Healthy Eating awards have been developed as well as healthy schools guidelines to be followed (Irish Heart Foundation).
**Evidence of implementation**	**Nutritional Standards for School Meals**<br><br>● The Nutritional Standards for School Meals were designed to ensure the School Meals programmes follow the national Healthy Eating Guidelines (Healthy Ireland, 2017a).<br>● The 3-week menu plan resource, implemented by the HSE provides a practical guide for pre-schools to carry out the guidelines and to provide balanced, nutritious meal ideas. All recipes are suitable for children from 1 year onwards and the portion sizes are based on the requirements of children aged 1½ - 3 years<br>**The resource:**<br>● Caters for up to 30 child servings.<br>● Gives information on food safety and preparation.<br>● Provides balanced, nutritious meal ideas including recipes.<br>● Supplies recipes which are suitable for children from 1 year onwards.<br>● Offers tooth friendly drinks.<br>● Each recipe contains the ingredients, measurements, method, alternative options to certain ingredients, tips and hints.<br><br>**Food and nutrition guidelines for pre-school services**<br><br>● These guidelines are produced by the Department of Health that, assist pre-schools in providing healthy food. They also help in creating an environment that promotes positive attitudes to eating and physical

activity as part of a healthy lifestyle. They are relevant to pre-school children aged 0-5 years and are intended as a resource and guide for all relevant stakeholders which include carers, parents and pre-school inspectors. Currently, they're being developed into nutrition standards 2020

Early years Quality and Regulatory Framework

- The Early Years Inspectorate has developed a Quality and Regulatory Framework (QRF), to support registered Early Years Services to comply with the 2016 Regulations. The QRF does this by setting out the core regulatory requirements in a transparent way, bringing together evidence-based, national and international research and best practice in Early Years. The QRF aims to support registered providers in achieving compliance with the regulations and enhance the safety and care of children who attend these services. The QRF is child-centred, with a specific focus on the quality and safety of the care provided directly to children using the services. Tusla Early Years Inspectorate, in collaboration with the Department of Children and Youth Affairs, launched the Quality and Regulatory Framework on Wednesday the 5th of September 2018 in Dublin. (TUSLA, 2018a)

There is a QRD relevant to all services below:

1. Full day care service and part-time day care service
2. Sessional pre-school service
3. Childminding
4. Pre – school service in a drop-in centre
5. Overnight pre-school service (For a stand-alone overnight service)
6. Overnight pre-school service

First 5: whole-of-Government strategy

- First 5 is a whole-of-Government strategy to improve the lives of babies, young children and their families. It is a ten-year plan that, uses evidence to identify goals, objectives and the specific actions required from across Government to support children (and their families) in the early years of life (Department of Children and Youth Affairs, 2018a). First 5 commits to major initiatives on;

1. broader range of options for parents to balance working and caring
2. a new model of parenting support

	3. new developments in child health, including a dedicated child health workforce<br>4. reform of the Early Learning and Care (ELC) system, including a new funding model<br>5. a package of measures to tackle early childhood poverty
**Comments/ notes**	

**PROV5**<br>**The Government actively encourages and supports private companies to provide and promote healthy foods and meals in their workplaces**	
**Definitions**<br><br>**and scope**	• For the purpose of this indicator, 'private companies' includes for-profit companies and extends to non-government organisations (NGOs) including not-for-profit/charitable organisations, community-controlled organisations, etc.<br>• Includes healthy catering policies, fundraising, events - Includes support and training systems including guidelines, toolkits, templates (e.g. policy/guidelines or contracts), recipes and menu planning tools, expert advice, menu and product assessments, online training modules, cook/caterer/other food service staff information and training workshops or courses (where relevant to the provision of food in a workplace).<br>• Excludes the provision or promotion of food to people not employed by that organisation (e.g. visitors or customers).<br>• Excludes support for organisations to provide staff education on healthy foods.
**International best practice examples (benchmarks)**	• **Ireland:** A Healthy Workplaces Framework has been developed as part of the governments public health Framework, Healthy Ireland - A Framework for improved health and wellbeing: 2013 – 2025, by the Department of Health and the Department of Business, Enterprise and Innovation (Department of Health, 2013) (McAvoy, 2018b). It was developed following a public consultation with interested stakeholders and aims to enhance existing initiatives to, facilitate the sharing of experience and learning, and also provide the necessary supports and tools for organisations or companies who haven't yet developed their own resources. The Healthy Workplaces Framework is due to be implemented in 2020.
**Context** e.g. EU action/ regulation / food industry action etc.	**Industry action**<br><br>**National Workplace Wellbeing Day – Ibec and the Nutrition and Health Foundation**<br>• An initiative of Ibec and the Nutrition and Health Foundation, Ireland's National Workplace Wellbeing Day is a nationwide campaign designed to help improve employee health through promoting better nutrition and physical activity in the workplace. Ibec launched Ireland's fifth National Workplace Wellbeing Day, on Friday, 12th April 2019. Public and private sector organisations across the country are expected to

	participate in the day, which aims to improve employee health through promoting better physical activity and nutrition in the workplace (Nutrition and Health Foundation, 2019)<br><br>**The KeepWell Mark – Ibec**<br><br>• In 2017, Ibec introduced the KeepWell Mark, a workplace wellbeing accreditation which helps companies demonstrate their organization's commitment to improving the lives of those who work for them. The accreditation process includes benchmarking and an assessment against recognised standards in workplace health, safety and wellbeing with a personalised report being prepared with recommendations for corporate wellbeing strategy and a certificate of accreditation which is valid for two years<br>• It's an evidence based accreditation, that offers all types of employers, large and small, from the public, private and voluntary sectors, an opportunity to demonstrate their commitment to the health and wellbeing of their workforce (Irish Business and Employers Confederation, 2017)<br><br>**Irish Heart Foundation (IHF) Workplace Wellness Programmes**<br><br>• Over 1000 workplaces have received support from Irish Heart Foundation(Irish Heart Foundation, 2019). The workplace wellness programs include:<br>1. Physical activity - Slí@work comprises a series of indoor and outdoor motivational signage that encourage staff to add walking into their working day.<br>2. Healthy eating – the Happy Heart Healthy Eating award programme assists companies staff restaurant to adopt healthier cooking practices and providing healthier food choices, without incurring substantial cost. There are three different levels of achievement for the award: bronze, silver, and gold.<br>3. Health checks provided by qualified and experienced nurses to provide tailored lifestyle advice. These options include a combination of the following:<br><br>• Blood pressure check

	• Cholesterol check (Full lipid profile)<br>• Glucose check<br>• Weight management (Waist circumference & BMI)<br>• Carbon monoxide check (For smokers)<br>• All participants will receive a personal record card and tailored lifestyle advice from a cardiovascular-skilled nurse.
**Evidence of implementation**	**A Healthy Workplaces Framework**<br><br>• A Healthy Workplaces Framework is currently being developed as part of the governments public health Framework, Healthy Ireland - A Framework for improved health and wellbeing: 2013 – 2025 (Department of Health, 2013). This Framework is being developed by the Department of Health and the Department of Business, Enterprise and Innovation (McAvoy, 2018b). It is intended to provide a supportive, flexible, evidence-based structure that can be applied across all workplaces in Ireland regardless of their sector or size.<br><br>• In 2016, a Steering Group on the development of a Healthy Workplaces Framework was convened. The Steering Group is co-chaired by the Department of Health and the Department of Business, Enterprise and Innovation(McAvoy, 2018a).<br><br>• A consultation process was undertaken with interested stakeholders to inform the development of the Framework. Data collection occurred between March and May 2017. The consultation design and data collection were informed by pre-consultation expert focus groups and a cross-disciplinary steering committee. The process comprised of an online questionnaire as well as four regional workshops. The consultation sought views on strategic level issues such as the Framework vision, aim and strategic goals/objectives and potential priority health topics and subgroups of workers. It also sought views on operational level

	issues relating to resources to support implementation and monitoring/evaluation (McAvoy, 2018a)<br><br>• The Department of Health has produced 3 research papers that are intended to inform the development of the Framework:<br>1. An umbrella review of the effectiveness and cost-effectiveness of Workplace Wellbeing Programmes (Murphy, 2018c).<br>2. A description of public policy mechanisms to support Healthy Workplaces and Workplace Health Programmes (Murphy, 2018a).<br>3. Factors organisations should consider when developing Healthy Workplaces and Workplace Wellbeing Programmes (Murphy, 2018b).<br><br>• The Healthy Workplaces Framework is due to be implemented in 2020
**Comments/ notes**	

DOMAIN 6 - FOOD IN RETAIL: The government has the power to implement policies and programs to support the availability of healthy foods and limit the availability of unhealthy foods in communities (outlet density and locations) and in-store (product placement)

RETAIL1<br>**Zoning laws and policies are implemented to place limits on the density or placement of quick serve restaurants or other outlets selling mainly unhealthy foods in communities and/or access to these outlets (e.g. opening hours).**	
**Definitions and scope**	• Includes the consideration of public health in State/Territory Planning Acts that guide the policies, priorities and objectives to be implemented at the local government level through their planning schemes<br>• Includes the consideration of public health in State/Territory subordinate planning instruments and policies<br>• Includes a State/Territory guideline that sets the policy objective of considering public health when reviewing and approving fast food planning applications.<br>• Includes limitations to access of unhealthy food outlets (i.e. opening hours)<br>• Excludes laws, policies or actions of local governments
**International best practice examples (benchmarks)**	**South Korea**: In 2010 the Special Act on Children's Dietary Life Safety Management established the creation of 'Green Food Zones' around schools, banning the sale of foods (fast food and soda) deemed unhealthy by the Food and Drug Administration of Korea within 200 metres of schools (Ministry of Food and Drug Safety, 2017, Bae, 2012) In 2016, Green Food Zones existed at over 10000 schools.<br><br>**UK:** Around 15 local authorities have developed "supplementary planning documents" on the development of hot food takeaways. The policies typically exclude hot food takeaways from a 400m zone around the target location. All policies include secondary schools, some policies also include primary schools, parks and youth centres(World Cancer Research Fund, 2016).<br><br>**Detroit USA:** In Detroit, the zoning code prohibits the building of fast food restaurants within 500 ft. of all elementary, junior and senior high schools (World Cancer Research Fund, 2016).

**Context** e.g. EU action/ regulation / food industry action etc.	**EU regulation**<br><br>The EU does not have competence in the field of spatial planning, or on implementing zoning laws and policies to place limits on the density or placement of quick service restaurants selling mainly unhealthy foods or to encourage the availability of outlets selling fruits and vegetables.<br><br>**No Fry Zone 4 Kids committee**<br><br>• No Fry Zone for Kids is a local community action group based in Greystones, Co. Wicklow. From August 2015, the No Fry Zone 4 Kids Committee has worked closely with the Greystones councillors to develop a specific No Fry Zone objective (Philip Moyles, 2018). On the 7th of November, Wicklow County Council voted on Objective RT 17, with the following criteria taken into account in the assessment of development proposals for fast-food/takeaway outlets including those with a drive through facility:<br>1. Exclude any new fast-food/takeaway outlet from being built or from operating within 400m of the gates or site boundary of schools or playgrounds, excluding premises zoned town centre.<br>2. Fast food outlets/takeaways with proposed drive through facilities will generally only be acceptable within Major Town Centres or District Centres and will be assessed on a case-by-case basis<br>3. Location of vents and other external services and their impact on adjoining amenities in terms of noise/smell/visual impact (Oireachtas, 2018)<br>• It was ratified with 25 councilors voting to adopt this into the plan, out of 32 councillors (majority required). 3 voted against, 2 abstained and 2 were not present. Wicklow became the first county in Ireland to implement a No Fry Zone policy around schools with a specified distance of 400 metres (Oireachtas, 2018).
**Evidence of implementation**	**Department of Environment Guidelines for Planning Authorities 2013**<br><br>• The Guidelines for Planning Authorities, as issued under Section 28 of the Planning and Development Act, 2000, state that planning

	authorities “shall seek to promote active and healthier lifestyles by ensuring that exposure of children to the promotion of foods that are high schools in fat, salt or sugar is reduced through careful consideration of the appropriateness and/or location of fast food outlets in the vicinity of schools and parks”. Section 5.2 specifies that planning authorities should focus on the needs of local communities through the Local Area Plan (Department of the Environment, 2013).<br><br>**‘A Healthy Weight for Ireland: Obesity Policy and Action Plan 2016 – 2025’**<br><br>• Action 2.1 of the Obesity Policy and Action Plan states “Develop guidelines and support materials for those working in developing the built environment for urban development and planning in relation to reducing the obesogenic environment”. In line with this Action, and with Action 31 of the National Physical Activity Plan (NPAP) (Department of Health, 2016a), which commits to developing guidelines and support materials for those working in developing the built environment in order to promote the importance of physical activity, and Action 59 of the NPAP which commits to developing a programme of on-going stakeholder communication and engagement, a Stakeholder Forum was held on November 21st, 2018.<br><br>• This Forum, entitled *Connect, Collaborate, Create; Co-Designing Healthier Communities, A Healthy Ireland Stakeholder Forum on the Built Environment*, brought together those who have a responsibility for planning and developing public spaces with those who have expertise regarding the impacts of the built environment on health and wellbeing. It provided an opportunity for interdisciplinary discussion on how enhanced collaboration might be supported in the future, to create healthier communities (Oireachtas, 2019).
**Comments/notes**	

RETAIL2<br>**Zoning laws and policies are implemented to encourage the availability of outlets selling fresh fruit and vegetables and/or access to these outlets (e.g. opening hours, frequency i.e. for markets) .**	
**Definitions**<br><br>**and scope**	• Outlets include supermarkets, produce markets, farmers' markets, greengrocers, food cooperatives<br>• Includes fixed or mobile outlets<br>• Excludes community gardens, edible urban or backyard gardens (usually regulated by local governments)<br>• Includes policies that support local governments to reduce license or permit requirements or fees to encourage the establishment of such outlets<br>• Includes State/Territory policies to streamline and standardise planning approval processes or reduce regulatory burdens for these outlets<br>• Includes actions to improve access to healthy food outlets (i.e. opening hours; frequency i.e. for markets)<br>• Includes the provision of financial grants or subsidies to outlets
**International best practice examples (benchmarks)**	**USA:** February 2014 the US Congress formally established the Healthy Food Financing Initiative (following a three-year pilot) which provides grants to states to provide financial and/or other types of assistance to attract healthier retail outlets to underserved areas. The pilot distributed over 140 million USD in grants to states to provide financial and other types of assistance to attract healthier retail outlets in underserved areas. To date, 23 US states have implemented financing initiatives (World Cancer Research Fund, 2016).<br><br>**New York City (USA):** The 'Green Cart Permit' was developed with reduced restrictions on zoning requirements to increase the availability of fresh fruits and vegetables in designated, underserved neighbourhoods (World Cancer Research Fund, 2016). In 2008 New York City made 1000 licenses for green carts available to street vendors who exclusively sell fresh fruit and vegetables in neighbourhoods with limited access to healthy foods (World Cancer Research Fund, 2016). In addition, in 2009, New York City established the food retail expansion to support the health program of New York City (FRESH). Under the programme, financial and zoning incentives are offered to promote neighbourhood grocery stores offering fresh meat, fruit and vegetables in under-served communities. The financial benefits consist of an exemption or reduction of certain taxes. The zoning incentives consist of providing

	additional floor area in mixed buildings, reducing the amount of required parking, and permitting larger grocery stores in light manufacturing districts.
**Context** e.g. EU action/ regulation / food industry action etc.	**Support systems for food stores and food service outlets**<br><br>● There are no support systems at EU level to encourage food stores to promote the in-store availability of healthy foods and to limit the in-store availability of unhealthy foods. Neither are there support systems at EU level to encourage the promotion and availability of healthy foods in food service outlets and to discourage the promotion and availability of unhealthy foods in food service outlets.
**Evidence of implementation**	**Department of Agriculture, Food and Marine**<br><br>• There is no specific planning legislation in place in relation to encouraging outlets to sell fresh fruit and vegetables. However, the Department of Agriculture, Food and Marine provided a Code for Good Practice for Farmers Markets in 2013. The code of good practice is intended as a basic standard to promote the vision of- offering a route to market for local produce and small food producers, attracting consumers and promoting sustainable and diverse food cultures at county and local level. (Department of Agriculture Food and Marine and An Bord Bia, 2009)<br><br>**No further evidence found from 2016 onwards**
**Comments/notes**	

**RETAIL3**<br>**The Government ensures existing support systems are in place to encourage food stores to promote the in-store availability of healthy foods and to limit the in-store availability of unhealthy foods**	
**Definitions**<br><br>**and scope**	• Food stores include supermarkets, convenience stores (including 'general stores' or 'milk bars'), greengrocers and other speciality food retail outlets.<br>• Support systems include guidelines, resources or expert support<br>• Includes all settings with food retail stores, including but not exclusive to; train stations, venues, facilities or events frequented by the public etc.<br>• Excludes settings owned or managed by the government (see 'PROV2' and 'PROV4').<br>• Includes the strategic placement of foods and beverages in cabinets, fridges, on shelves or near the cashier - Includes the use of signage to highlight healthy options or endorsements (such as traffic lights or a recognised healthy symbol).<br>• Includes offering healthier food and drink products, or changing the menu or store layout to offer more healthy options<br>• Includes decreasing the offer of unhealthy food and drink products.<br>• Excludes reformulation and labelling in relation to nutrients of concern (COMP1; LABEL4).
**International best practice examples (benchmarks)**	**USA:** The Special Supplemental Nutrition Program for Women, Infants, and Children (WIC) requires WIC authorised stores to stock certain healthier products (e.g. wholegrain bread) (World Cancer Research Fund, 2016f).<br><br>**The Netherlands:** The National Action plan for vegetables and Fruit is a cooperation of government, industry and civil society organisations. The Goal is to increase the consumption of vegetables and fruits in 3 years (2018-2020) by linking and strengthening existing initiatives**.** The National Action Plan vegetables and fruit stimulates consumers to eat more vegetables and fruit using the motto 'Go for Colour'. As part of 'Go for Colour' an in-store experiment has taken place promoting the in-store availability of vegetables and fruit.
**Context** e.g. EU action/ regulation / food industry action etc.	**Food Industry Action**<br><br>• Companies in Ireland are not legally obliged to promote in store availability of healthier foods and limit in store availability of unhealthy foods.

**Evidence of implementation**	**Non-Broadcast Media Advertising and Marketing of Food and Non-Alcoholic Beverages, including Sponsorship and Retail Product Placement: Voluntary Codes of Practice**<br><br>Under the codes, the retail food industry in Ireland undertakes to operate the specific rules for retail product placement as detailed below in addition to any relevant general rules applicable to all codes:<br>1. Active promotion of the consumption of 5 to 7 portions of fruit and vegetables a day by customers in store through point of sale materials and other online and offline communication platforms.<br>2. Provision of meal deal offers that deliver choice for customers in order to promote a healthy balanced diet.<br>3. Where practical, the provision of a HFSS food free checkout option to customers to allow the use of a checkout lane that does not stock HFSS foods. In retail environments with 4 or more checkout bays, a minimum of 1 in 4 bays should be free of HFSS foods. Other outlets with less than 4 checkouts are encouraged to provide 1 non-HFSS checkout, where practical.<br>4. Provision of calorie labelling information in food service areas such as deli's and hot food counters (Healthy Ireland, 2017b).
**Comments/notes**	

RETAIL4<br>**The government ensures existing support systems are in place to encourage the promotion and availability of healthy foods in food service outlets and to discourage the promotion and availability of unhealthy foods in food service outlets**	
**Definitions<br><br>and scope**	• Food service outlets include for-profit quick service restaurants, eat-in or take-away restaurants, pubs, clubs<br>• Support systems include guidelines, resources or expert support<br>• Includes settings such as train stations, venues, facilities or events frequented by the public<br>• Excludes settings owned or managed by the government (see 'PROV2' and 'PROV4')<br>• Includes the strategic placement of foods and beverages in cabinets, fridges, on shelves or near the cashier - Includes the use of signage to highlight healthy options or endorsements (such as traffic lights or a recognised healthy symbol)<br>• Includes modifying ingredients to make foods and drinks more healthy, or changing the menu to offer more healthy options<br>• Excludes reformulation and labelling in relation to nutrients of concern (COMP2; LABEL4)
**International best practice examples (benchmarks)**	**USA:** In December 2011, San Francisco implemented the Health Food Incentives Ordinance which bans restaurants, including takeaway restaurants, to give away toys and other free incentive items with children's meals unless the meals meet nutritional standards as set out in the Ordinance: meals must not contain more than 600 calories and include a min amount of fruits and vegetables. It also applies to drinks with excessive calories, fat, excessive sugars ,added non-nutritive sweeteners or caffeine (World Cancer Research Fund, 2016e).<br><br>**France:** Since January 2017 France has banned unlimited offers of sweetened beverages for free or at a fixed price in public restaurants and other facilities accommodating or receiving children under the age of 18. Sweetened beverages are defined as any drink sweetened with sugar or artificial (caloric and non-caloric) sweeteners, including flavoured carbonated and still beverages, fruit syrups, sport and energy drinks, fruit and vegetable nectars, fruit- and vegetable-based drinks, as well as water- milk- or cereal based beverages (World Cancer Research Fund, 2016).<br><br>**Los Angles, USA:** In September 2013, the Los Angeles County Department of Public Health launched Choose Health LA Restaurants in partnership with

	local restaurants to promote healthier meal choices. Restaurants must apply to become a partner. Participating restaurants offer customers smaller portion size options (in addition to existing items on the menu), healthier meals for children that include vegetables and fruit, healthy beverages, non-fried food and free chilled water. Participating restaurants are recognised as Public Health partners in promoting healthier communities.
**Context** e.g. EU action/ regulation / food industry action etc.	**Food Industry Action**<br>• Companies in Ireland are not legally obliged to promote in store availability of healthier foods and limit in store availability of unhealthy foods
**Evidence of implementation**	**Alcoholic Beverages, including Sponsorship and Retail Product Placement: Voluntary Codes of Practice**<br><br>Under the codes, the retail food industry in Ireland undertakes to operate the specific rules for retail product placement as detailed below in addition to any relevant general rules applicable to all codes:<br><br>1. Active promotion of the consumption of 5 to 7 portions of fruit and vegetables a day by customers in store through point of sale materials and other online and offline communication platforms.<br>2. Provision of meal deal offers that deliver choice for customers in order to promote a healthy balanced diet.<br>3. Where practical, the provision of a HFSS food free checkout option to customers to allow the use of a checkout lane that does not stock HFSS foods. In retail environments with 4 or more checkout bays, a minimum of 1 in 4 bays should be free of HFSS foods. Other outlets with less than 4 checkouts are encouraged to provide 1 non-HFSS checkout, where practical.<br>4. Provision of calorie labelling information in food service areas such as deli's and hot food counters (Healthy Ireland, 2017b)
**Comments/notes**	

Healthy Food Environment Policy Index: Infrastructure domains

DOMAIN 8 – LEADERSHIP: The political leadership ensures that there is strong support for the vision, planning, communication, implementation and evaluation of policies and actions to create healthy food environments, improve population nutrition, and reduce diet-related inequalities

**LEAD1**<br>**There is strong, visible, political support (at the head of government or state/ ministerial level) for improving food environments, population nutrition, diet related NCDs and their related inequalities"**	
**Definitions and scope**	• Visible support includes statements of intent, election commitments, budget commitments, establishing priorities and targets, demonstration of support in the media, other actions that demonstrate support for new or strengthened policy<br>• Documents that contain evidence of strong political support include media releases, speeches, pre-election policy papers, introduction of a bill, State-level strategic plans with targets or key performance indicators
**International best practice examples (benchmarks)**	**New York City (USA):** As Mayor of New York City, Michael Bloomberg prioritised food policy and introduced a number of ground breaking policy initiatives including 'Health Bucks', a restriction on trans fats, establishment of an obesity taskforce, a portion size restriction on sugar-sweetened beverages, public awareness campaigns, etc. He showed strong and consistent leadership and a commitment to innovative approaches and cross-sectoral collaboration (Kelly, 2016).<br><br>**Brazil:** The Minister of Health showed leadership in developing new dietary guidelines that are drastically different from the majority of dietary guidelines created by any nation to date, and align with some of the most commonly cited recommendations for healthy eating (World Public Health Nutrition Association Update team, 2014).<br><br>**Some Caribbean Countries:** Active NCD commissions exist in six of the 20 CARICOM member states (Bahamas, Barbados, Bermuda, British Virgin

	Islands, Dominica, Grenada) which are all housed in their Ministries of Health, with members recommended by the Minister of Health and appointed by the Cabinet of Government for a fixed duration; all include government agencies and to a varying degree, civil society and the private sector<br><br>**Ireland:** Healthy Ireland "A Framework for Improved Health and Wellbeing 2013-2025", was launched in 2013 and aims to increase the proportion of people who are healthy at all stages of life, reduce health inequalities, protect the public from threats to health and wellbeing and create an environment where every individual and sector of society can play their part in achieving a healthy Ireland. In 2016, the Government approved the creation of a Healthy Ireland Fund with an initial allocation of €5 million approved in Budget 2017 to establish and support the implementation of Healthy Ireland programmes and projects in a variety of settings. The primary aim of the fund is to support innovative, cross-sectoral, evidence-based projects and initiatives that support the implementation of key national policies in areas such as obesity, smoking, alcohol, physical activity and sexual health(Pobal, 2016). The Department of Health has approved a third round of funding, which aims to support local and national organisations to deliver actions that will improve health and wellbeing in line with Healthy Ireland, A Framework for Improved Health and Wellbeing 2013-2025 (Department of Health, 2019b).<br><br>**The Netherlands:** In 2018, the Ministry of Health, together with more than 70 organizations signed the National Prevention Agreement. It aims to reduce smoking, overweight and problematic alcohol consumption. The agreement includes voluntary ambitions, objectives and actions on these three subjects for the period (2018-2040). The National prevention agreement acknowledges that peoples' contexts is important, and that, among other factors, a healthy environment is needed for those that need it in order to prevent overweight, obesity and NCD's. The agreement formulates that inhabitants of the Netherlands need a healthy social, economic and physical environment, that supports healthy living, including schools, care facilities, restaurants, cafes, caterers and supermarkets. Specific voluntary targets with respect to the food environment are:<br><br>• In 2020, 2,500 sports clubs will be working on providing a healthier range of food products in their sports canteens.

	• No later than 2025, 50% of hospitals will offer a healthy diet; no later than 2030, all of them will.<br><br>• In 2020, there will be 950 healthy school canteens. This means that 50% of all school canteens will be healthy.<br><br>• Businesses will develop healthier products (e.g. reformulation by limiting sugar content)<br><br>• The central government wants to introduce, no later than 2020, a new, broadly supported food-choice logo based on thorough, independent consumer research.<br><br>An example of a voluntary actions formulated is: Supermarkets will entice consumers to buy more products from the Wheel of Five, among other things by telling them which products are suitable (Netherlands, 2019).
**Context** e.g. EU action/ regulation / food industry action etc.	**EU Action**<br><br>**Strategic Plan DG Sante 2016-2020**<br>• The Strategic Plan of DG Sante refers to the Treaty on the Functioning of the European Union, which shapes the EU's degree of influence in health and food policies, saying that Member States are responsible for the definition of their health policy and for the organization and delivery of health services and medical care (European Comission, 2016a). DG Sante therefore states that EU action is mainly linked to incentive measure, e.g. raising awareness to prevent chronic disease and promote good health and cooperation measures. However, one of its missions is to 'improve and protect human health'.<br>• The Paper 'Initiatives on Nutrition and Physical Activity'(European Comission, 2019b) makes clear that the Commission offers practical tools to help Member States and EU citizens to make real progress in the areas of nutrition and physical activity.<br><br>**European Commission's Reflection Paper towards a sustainable Europe by 2030 (2019)**<br>• The Commission recognizes that overweight and obesity are the EU's central nutrition issues. Achieving safe and healthy diets and ensuring productive and sustainable agricultural systems, fisheries and aquaculture are a priority in the EU (European Comission, 2019d).

**Evidence of implementation**	**Healthy Ireland "A Framework for Improved Health and Wellbeing 2013-2025"**<br>• The Healthy Ireland Framework was launched in 2013 with a Foreword by the then Taoiseach Enda Kenny and an Introduction by the then Minister for Health Dr. James Reilly, T.D. It articulates four central goals for improved health and wellbeing:<br>- increase the proportion of people who are healthy at all stages of life<br>- reduce health inequalities<br>- protect the public from threats to health and well being<br>- create an environment where every individual and sector of society can play their part in achieving a healthy Ireland<br>- The Cabinet Committee on Social Policy oversees the delivery of this Framework. The Health and Wellbeing Programme in the Department of Health is responsible for strategic planning and co-ordination of the implementation of the Framework actions. A multi-stakeholder Healthy Ireland Council was established to provide a national advisory forum to support the implementation of the Framework across sectors. The Framework was accompanied later in 2013 by a high-level implementation plan and an Outcomes Framework (Department of Health, 2013)<br><br>**"A Healthy Weight for Ireland: Obesity Policy and Action Plan 2016-2025"**<br>• The OPAP was launched with a Foreword by the Minister for Health, Simon Harris TD and a Foreword by the then Minister of State for Health Promotion, Marcella Corcoran Kennedy TD. The policy sets out "Ten Steps Forward" which aim at reversing the obesity trend while preventing complications associated with obesity and reducing the overall burden for individuals, their families and the health system. The "Ten Steps Forward" are:<br>1. Embed multi-sectoral actions on obesity prevention with the support of government departments and public sector agencies.<br>2. Regulate for a healthier environment.

	3. Secure appropriate support from the commercial sector to play its part in obesity prevention.<br>4. Inform and empower change through a clear communications strategy.<br>5. The Department of Health will provide leadership.<br>6. Mobilise the health services with a focus on prevention.<br>7. Develop a service model for specialist care for children and adults.<br>8. Acknowledge the key role of physical activity in the prevention of overweight and obesity.<br>9. Allocate resources according to need in particular for children and disadvantaged groups.<br>10. Monitor research and review.<br><br>**Healthy Ireland fund**<br>• In 2016, the Government approved the creation of a Healthy Ireland Fund with an initial allocation of €5 million approved in Budget 2017 to establish and support the implementation of Healthy Ireland programmes and projects in a variety of settings. The primary aim of the fund is to support innovative, cross-sectoral, evidence-based projects and initiatives that support the implementation of key national policies in areas such as obesity, smoking, alcohol, physical activity and sexual health. The funding has been allocated to 2 Strands, both of which are closed calls for funding. Strand 1 focuses on the delivery of local initiatives and Strand 2 funds national actions (Pobal, 2016).<br><br>• The Department of Health has approved a third round of funding for the Healthy Ireland Fund (HIF). The aim of this funding is to support local and national organisations to deliver actions that will improve health and wellbeing in line with *Healthy Ireland, A Framework for Improved Health and Wellbeing 2013-2025* (Department of Health, 2019b).<br><br>**The specific objectives for Round 3 of the Healthy Ireland Fund are:**<br><br>1. To raise awareness of, and support for, Healthy Ireland through the funding of community-<br>2. based and national health promotion activities.

3. To facilitate and resource cross-sectoral and partnership approaches that promote health
4. and well-being.
5. To resource the development and implementation of locally-led, cross-sectoral strategies
6. promoting health and wellbeing.
7. To add value to existing health promotion initiatives through the provision of Healthy Ireland
8. resources.
9. To support and implement actions which will have a positive impact on the health and wellbeing of the most disadvantaged.

Target Groups

- Actions supported by HIF with a focus on supporting the health and wellbeing of the population in general are eligible. However, the following are the priority target groups for this round of funding:
  - Disadvantaged communities
  - Disadvantaged men and women
  - Disadvantaged families, including one parent families
  - Children and young people
  - People with disabilities, including people mental health issues
  - Unemployed young people and adults
  - Traveller and Roma communities
  - New communities, asylum seekers and refugees
  - LGBTI
  - Homeless people
  - Older people

Healthy Ireland campaign (2019)

- Taoiseach Leo Varadkar TD, Minister for Health Simon Harris TD and Minister for Health Promotion, Catherine Byrne TD have launched the 2019 Healthy Ireland campaign and announced funding of €1 million to boost community engagement on health and wellbeing in every county. The 2019 campaign seeks to raise public awareness of gov.ie/HealthyIreland as the place to go that brings trusted

	sources of information and supports together in one place to help people get started on making healthy changes.(Department of Health, 2019d)<br><br>**National Healthy Cities and Counties of Ireland Network**<br><br>• National Healthy Cities and Counties of Ireland Network was launched in 2016 by the Minister for Health Promotion. The network was launched under the Healthy Ireland “A Framework for Improved Health and Wellbeing 2013-2025”, and has representatives from government departments, the HSE, the Institute of Public Health, the Federation of Irish Sport and local political and community representative (Department of Health, 2019c) The network aims to:<br><br>- Promote lifelong health and wellbeing,<br>- Provide a means where local issues can influence national policy, and<br>- Provide a voice for Ireland in the WHO Network of European National Healthy Cities Networks (Department of Health, 2019c)
**Comments/notes**	

**LEAD2**<br>**Clear population intake targets have been established by the government for the nutrients of concern and / or relevant food groups to meet WHO and national recommended dietary intake levels**	
**Definitions**<br><br>**and scope**	• Includes targets which specify population intakes according to average reductions in percentage or volume (e.g. mg/g) for salt/sodium, saturated fat, trans fats or added or free sugars* Typically requires the government to establish clear dietary guidelines on the maximum daily intake of nutrients of concern.<br>• **Free sugar is defined as is the sugar no longer in its naturally-occurring state (i.e., no longer in whole fruits, vegetables, unsweetened dairy, and grains) and can be consumed as is or incorporated into other foods. Examples include table sugar, syrup, honey, fruit juice and nectars. Added sugar is defined as the free sugar that has been added to foods, however regulatory definitions vary widely under different jurisdictions, some of which are currently under review. These differ from naturally occurring sugars, defined as the sugar found naturally within whole foods (i.e., within whole fruits, vegetables, dairy, and some grains).
**International best practice examples (benchmarks)**	**Brazil:** The "Strategic Action Plan for Confronting NCDs in Brazil, 2011-2022 specifies a target of increasing adequate consumption of fruits and vegetables, from 18.2% to 24.3 % between 2010 and 2022 and reduction of the average salt intake of 12 g to 5 g, between 2010 and 2022 (Ministry of Health Brazil, 2011).<br><br>**South Africa:** The South African plan for the prevention and control of non-communicable diseases includes a target on reducing mean population intake of salt to <5 grams per day by 2020 (Ministry of Health South Africa, 2013).<br><br>**UK:** In August 2016, government set out its approach to reduce the prevalence of childhood obesity in 'Childhood obesity: a plan for action'. A key commitment in the plan was to launch a broad, structured sugar reduction programme to remove sugar from everyday products. All sectors of the food and drinks industry are challenged to reduce overall sugar across a range of products that contribute most to children's sugar intakes by at least 20% by 2020, including a 5% reduction in the first year of the programme (Public Health England, 2017).

	**The Netherlands:** On January 2014, the Dutch Ministry for Health, Welfare and Sport, signed an agreement with trade organisations representing food manufactures, supermarkets, hotels, restaurants, caterers and the hospitality industry. The agreement included intake targets for example; a maximum of 6 grams of salt consumption per day in 2020 and consuming a maximum of 10% energy from saturated fat per day in 2020 (The Central Government for the Netherlands, 2014)<br><br>**Norway:** The National Action Plan for a Better Diet (2017-2021) contains quantitative intake targets for nutrient of concern and specific food groups in the population. By 2021, the plan sets out a reduction of the following nutrients: Added sugar from 13 to 11E%; saturated fat from 14 to 12E%; and a 22% reduction in salt intake from 10 g/day. There are specific targets to halve the proportion of youth that consumes sugar-sweetened beverages or sweets more than 5 times per week; to double the proportion of youth that eats fruit and vegetables daily; and to increase by 20% the proportion of youth that eats fish at least once a week. There are also targets to increase the intake of fruit, vegetables, whole grain products and fish with 20% in the general population (Røynstrand, 2017).
**Context** e.g. EU action/ regulation / food industry action etc.	**European Commission-JRC**<br><br>• The Joint Research Centre has published dietary recommendations for nutrients of concern on the Health Promotion and Knowledge Gateway. It refers to different sources for their recommendations, like the EFSA, the WHO, WCRF/AICR, Food Based Dietary Guidelines in EU Countries, and the FAO. We can see from this Gateway, that EFSA does not establish clear population intakes for salt, sugar and fat. According to EFSA, the available data are not sufficient to establish an upper level for sodium and added sugars intake or an upper or lower level for total fat intake. The intake for saturated fatty acids and trans fatty acids should be as low as possible (European Commission, 2019,)
**Evidence of implementation**	**Department of Health and Food Safety Authority of Ireland recommendations**<br><br>• The population nutrition goals have been broadly adopted by the Department of Health and the Food Safety Authority of Ireland, in line with WHO/FAO recommendations as follows;

	- Less than 10% of daily energy from saturated fats<br>- Less than 1% of daily energy from trans fats<br>- Less than 10% of daily energy from added sugars progressively reducing to 5%*<br>- More than 400g fruits and vegetables a day<br>- Less than 5g a day of salt (6 g population recommendation FSAI 2016)<br><br>**Healthy Ireland and Department of Health**<br><br>• Together have established 'Eat Well'. Clear dietary guidelines using the Food pyramid on the maximum daily intake for foods and drinks high in fat, sugar and salt are established. It states, "*There are no recommended servings for this shelf because they are not needed for good health. Not every day – maximum once or twice a week*".<br><br>• Food guides have also been created to show people how to use the pyramid on a daily basis. For e.g. a guide for foods high in fat, sugar and salt has been created which contains a guide for portion size, cooking tips and shelf life for these foods, based on the food pyramid (Department of Health, 2019a).
**Comments/notes**	

**LEAD3**<br>**Clear, interpretive, evidenced-informed food based dietary guidelines have been established and implemented.**	
**Definitions and scope**	• Food-based dietary guidelines should be for both genders and key age groups including infants and pregnant women<br>• Evidence-informed includes extensive review of up-to-date research and mechanisms to seek expert input<br>• Evidence includes ways the FBDG have been used to develop/implement policies to improve diets
**International best practice examples (benchmarks)**	**Brazil:** The national dietary guidelines of Brazil address healthy eating from a cultural, ethical and environmental perspective, rather than based on number of servings per food group. The main recommendations are: 'Make natural or minimally processed foods the basis of your diet'; 'use oils, fats, salt, and sugar in small amounts for seasoning and cooking foods'; 'use processed foods in small amounts'; 'avoid ultra-processed foods'. They also provide advice on planning, shopping and sharing meals, as well as warning people to be wary of food marketing and advertising (Monteiro, 2015, Ministry of Health Brazil, 2014).<br><br>**Ireland:** A Healthy Weight for Ireland, Obesity Policy and Action Plan - 2016–2025 called for the development of a suite of Healthy Eating Guidelines for the general population. These revised Healthy Eating Guidelines and Food Pyramid resources, are an early action under the Plan (Action 5.3.) (Department of Health, 2016b). The revised Healthy Eating Guidelines and Food Pyramid are based on the FSAI Scientific Recommendations for Healthy Eating Guidelines in Ireland from 2011 (Food Safety Authority of Ireland, 2011). They describe how to build a healthy diet, for different age groups (from 5 years of age), depending also on gender and activity levels. The Guidelines, Food Pyramid and supporting resources have been published, disseminated and communicated in 2017, including dissemination of the new Guidelines to all primary and post-primary schools. The revised Healthy Eating Guidelines and Food Pyramid toolkit has been developed by the Department of Health and the Health Service Executive with key stakeholders and aims to help reduce the intake of high fat, salt and sugar (HFSS) foods and drinks from the Top Shelf of the Food Pyramid (Healthy Ireland, 2016a)<br><br>**The Netherlands:** The Dutch Health Council published the 'Guidelines Good Food' 2015. These guidelines advise to eat more plant-based and less animal-based food and include advice on the intake of different food products. The Dutch Nutrition Center published the 'Wheel of Five' Guidelines, based on the

	'Guidelines Good Food' of the Dutch Health Council. The 'Wheel of Five' includes advice on the ingredients of a healthy diet, making a distinction between five sections: (1) Vegetables and fruit (2) spread and cooking fats (3) Fish, legumes, meat, eggs, nuts and dairy products (4) Bread, cereal products and potatoes and (5) Drinks (Health Council of the Netherlands, 2015).
**Context** e.g. EU action/ regulation / food industry action etc.	**EU action**<br><br>The EU has not established food-based dietary guidelines for Member States. The Commission requested EFSA in 2010 to provide guidance on the translation of nutrient based dietary advice into food-based dietary guidelines (European food Safety Authority, 2010b) In reply to this, the EFSA Panel on Nutrition, Dietetic Products, and Allergies (NDA) published a Scientific Opinion on establishing Food-based dietary guidelines (European Food Safety Authority, 2010a). This Opinion states that the differences in EU countries regarding dietary habits, the prevalence of nutrient imbalances and diet-related public health issues require that food-based dietary guidelines are established by the country or region. All countries in the EU plus Switzerland, Norway and Iceland have FBDGs, which are published on the website of the European Commission (European Commission)
**Evidence of implementation**	**Department of Health – Healthy Eating Guidelines and the revised Food Pyramid**<br><br>• 'A Healthy Weight for Ireland', Obesity Policy and Action Plan 2016–2025 developed a suite of Healthy Eating Guidelines for the general population. These revised Healthy Eating Guidelines and Food Pyramid resources, are an early action under the Plan (Action 5.3.) The revised Healthy Eating Guidelines and Food Pyramid are based on the FSAI Scientific Recommendations for Healthy Eating Guidelines in Ireland from 2011 (Food Safety Authority of Ireland, 2011). They describe how to build a healthy diet, for different age groups (from 5 years of age), depending also on gender and activity levels. The Guidelines, Food Pyramid and supporting resources have been published, disseminated and communicated in 2017, including dissemination of the new

	Guidelines to all primary and post-primary schools (Healthy Ireland, 2016a).<br><br>• The revised Healthy Eating Guidelines and Food Pyramid toolkit has been developed by the Department of Health and the Health Service Executive with key stakeholders and aims to help reduce the intake of high fat, salt and sugar (HFSS) foods and drinks from the Top Shelf of the Food Pyramid (Healthy Ireland, 2016a). The revised Healthy Eating Guidelines and Food Pyramid are based on:<br>1. A review of the previous HEG conducted by the HRB (2013)<br>2. The Healthy Ireland Survey results (2015, 2016)<br>3. The FSAI Scientific Recommendations for Healthy Eating in Ireland (2011)<br>4. UK, EU, WHO and international recommendations (2012–2015)<br>5. Focus group testing with both consumers and health professionals.<br><br>• The Department of Health established a broad Stakeholder Working Group to develop the revised Healthy Eating Guidelines and Food Pyramid. This Working Group was chaired by the Department of Health and included representation from the Health Promotion Unit, Healthy Ireland and the Health Service Executive together with safefood, FSAI, INDI and the Health Research Board. A Department of Health led Technical Nutrition Sub-group of dietitians and nutritionists, on the Working Group worked on the technical nutrition details (Healthy Ireland, 2016b).<br><br>**The revised Food Pyramid model**<br><br>**The main revisions are**<br><br>**1.** A major focus on limiting Top Shelf Foods to only once or twice a week<br>**2.** Increasing the servings from the Vegetables, Salad and Fruit shelf from 5 a day up to 7. This means that the Vegetables, Salad and Fruit Shelf is the largest shelf and moves to the bottom of the Pyramid<br>**3.** The Wholemeal Cereals and Breads, Potatoes, Pasta and Rice Shelf then moves up from the bottom to be the second last shelf.<br>**4.** A single serving is now considered to be approximately 150kcal (plus or minus 25kcal) This now equates to 2 thin slices of regular pan bread at

	about 150 calories (FSAI, 2012). The range of servings from this Shelf is 3 to 5 servings/day – with up to 7 for teenage boys and men aged 19–50.<br>**5.** Limiting processed salty meats like sausages, bacon and ham to not every day, in line with the FSAI 2015 recommendations (Healthy Ireland, 2016b)<br><br>**Three key consumer messages are**:<br><br>**1.** Limit High Fat, Salt and Sugar (HFSS) foods and drinks from the Top Shelf of the Food Pyramid – NOT EVERY DAY – MAXIMUM ONCE OR TWICE A WEEK.<br>**2.** Eat more vegetables, salad and fruit – up to 7 servings a day. Encouraging a change in eating habits from HFFS snacks to eating more fruit and raw veg as snacks foods instead, for example fruit with yogurt, carrot sticks with low calorie dip.<br>**3.** Size matters – use the Food Pyramid as a guide for serving sizes, for example, bread in sandwiches at lunch time. 1 small wrap or a pitta pocket = 2 slices of bread. 1 demi baguette = 4 slices of bread (Healthy Ireland, 2016b)<br><br>• Work has also commenced on developing Healthy Eating Guidelines for the 1-5-year-old age group. As a first step in this work, the Scientific Committee of the Food Safety Authority of Ireland (FSAI) is currently developing scientific recommendations for food based dietary guidelines for 1 to 5 year olds (Oireachtas, 2019).<br><br>• A Food Guide outlining in detail the Healthy Eating Guidelines will be available for Health Professionals and Catering Services early in 2017. This Guide is being developed by the FSAI in conjunction with the Department of Health Technical Nutrition Sub-group. This Food Guide will form the basis of training for health professionals delivered by the HSE and supported by all stakeholders (Healthy Ireland, 2016b).<br><br>**Table 9: The Food Pyramid, Department of Health, 2016.**

	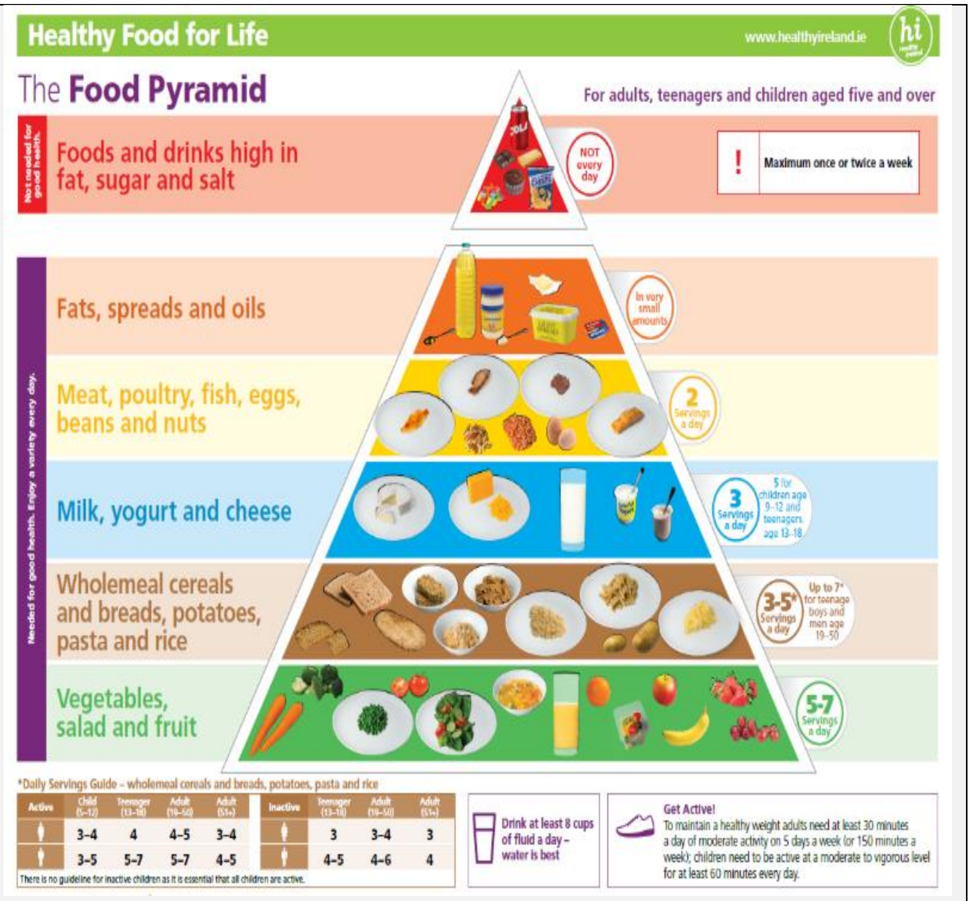
**Comments/notes**	

LEAD4<br>**There is a comprehensive, transparent, up-to-date implementation plan linked to national needs and priorities, to improve food environments , reduce the intake of the nutrients of concern to meet WHO and national recommended dietary intake levels, and reduce diet-related NCDs**	
**Definitions**<br><br>**and scope**	• Includes documented plans with specific actions and interventions (i.e. policies, programs, partnerships)<br>• Plans should be current (i.e. maintain endorsement by the current government and/or are being reported against)<br>• Plans should refer to actions to improve food environments (as defined in the policy domains above) and should include both policy and program strategies<br>• Excludes overarching frameworks that provide general guidance and direction<br>• Includes priority policy and program strategies, social media marketing for public awareness and threat of legislation for voluntary approaches.
**International best practice examples (benchmarks)**	**Ireland:** A Healthy Weight for Ireland', the Obesity Policy and Action Plan 2016-2025 (OPAP), was launched in September 2016 under the auspices of the Healthy Ireland agenda. The OPAP covers a ten-year period up to 2025, which prescribed 'Ten Steps Forward' that would be taken to reverse obesity trends, prevent health complications and reduce the overall burden for individuals, families, the health system, and the wider society and economy (Department of Health, 2016b). A new Obesity Policy Implementation Oversight Group (OPIOG) was established in October 2017 and a progress report on each recommendation in the OPAP is currently being finalised under the aegis of the OPIOG (Oireachtas, 2019). As set out in Healthy Ireland, integrated health and social impact assessments will be conducted on relevant policy areas to support other government departments in contributing towards the prevention of overweight and obesity. (Healthy Ireland, 2013-2025)
**Context** e.g. EU action/ regulation / food industry action etc.	**EU action**<br><br>• At EU level, there is one implementation plan to improve food environments, reduce the intake of the nutrients of concern and

	reduce diet-related NCDs, which is the EU Action Plan on Childhood Obesity 2014-2020.[1] This plan contains five areas of action:<br>1. Support a healthy start in life<br>2. Promote healthier environments, especially at schools and pre-schools<br>3. Make the healthy option, the easier option<br>4. Restrict marketing and advertising to children<br>5. Inform and empower families<br>Per area are operational objectives and responsible parties (EC, Member States, stakeholders) identified (Europa, 2014b).
**Evidence of implementation**	**Healthy Weight for Ireland: Obesity Policy and Action Plan 2016-2025**<br>• A Healthy Weight for Ireland', Obesity Policy and Action Plan 2016-2025 (OPAP), was launched in September 2016 under the auspices of the Healthy Ireland agenda. The OPAP covers a ten-year period up to 2025 and aims to reverse obesity trends, prevent health complications and reduce the overall burden for individuals, families, the health system, and the wider society and economy (Department of Health, 2016b).<br>• The OPAP policy prescribed 'Ten Steps Forward' that would be taken to prevent overweight and obesity:<br>1. Embed multi-sectoral actions on obesity prevention with the support of government departments and public sector agencies<br>2. Regulate for a healthier environment<br>3. Secure appropriate support from the commercial sector to play its part in obesity prevention<br>4. Implement a strategic and sustained communications strategy that empowers individuals, communities and service providers to become obesity aware and equipped to change, with a particular focus on families with children in the early years.

JPI a healthy diet for a healthy life
PEN Policy Evaluation Network
FOOD EPI
INFORMAS Benchmarking food environments

	5. The Department of Health, through Healthy Ireland, will provide leadership, engage and co-ordinate multi-sectoral action and implement best practice in the governance of the Obesity Policy and Action Plan.<br>6. Mobilise the health services to better prevent and address overweight and obesity through effective community-based health promotion programmes, training and skills development and through enhanced systems for detection and referrals of overweight and obese patients at primary care level.<br>7. Develop a service model for specialist care for children and adults<br>8. Acknowledge the key role of physical activity in the prevention of overweight and obesity<br>9. Allocate resources according to need, in particular to those population groups most in need of support in the prevention and management of obesity, with particular emphasis on families and children during the first 1,000 days of life<br>10. Develop a multi-annual research programme that is closely allied to policy actions, invest in surveillance and evaluate progress on an annual basis (Department of Health, 2016b)<br><br>**Obesity Policy Implementation Oversight Group (OPIOG)**<br>• A new Obesity Policy Implementation Oversight Group (OPIOG) was established in October 2017 and a progress report on each recommendation in the OPAP is currently being finalised under the aegis of the OPIOG. It is comprised of representatives from the following Departments and Agencies:<br>1. Department of Agriculture, Food and the Marine<br>2. Department of Children and Youth Affairs<br>3. Department of Employment Affairs and Social Protection<br>4. Department of Education and Skills; Department of Housing<br>5. Planning and Local Government<br>6. University College Cork<br>7. The Food Safety Authority of Ireland (FSAI)

	8. The Health Service Executive (HSE) - including the National Clinical Lead for Obesity; and Safefood.
**Comments/notes**	

**LEAD5**<br>**Government priorities have been established to reduce inequalities or protect vulnerable populations in relation to diet, nutrition, obesity and NCDs**	
**Definitions**<br><br>**and scope**	• Frameworks, strategies or implementation plans specify aims, objectives or targets to reduce inequalities including taking a preventive approach that addresses the social and environmental determinants of health<br>• Frameworks, strategies or implementation plans identify vulnerable populations or priority groups<br>• Implementation plans specify policies or programs that aim to reduce inequalities for specific population groups<br>• Excludes priorities to reduce inequalities in secondary or tertiary prevention
**International best practice examples (benchmarks)**	**New Zealand:** The Ministry of Health reports the estimates derived from health surveys and nutrition surveys by four subpopulation groups including age group, gender, ethnic group and an area level deprivation index. Similarly, estimates derived from other data types (e.g. mortality) are presented by these subpopulation groups. The contracts between MoH and NGOs or other institutions include a section on Maori Health and state: *"An overarching aim of the health and disability sector is the improvement of Maori health outcomes and the reduction of Maori health inequalities".* In the specific contract between the Ministry of Health and Agencies for Nutrition Action the first clause is on Maori Health relating to compliance with any Maori specific service requirements, quality requirements and specific monitoring requirements contained in the Service specifications to this agreement.<br><br>**Australia:** The National Indigenous Reform Agreement (Closing the Gap) is an agreement between the Commonwealth of Australia and the States and Territories. The objective of this agreement is to work together with Indigenous Australians to Close the Gap in Indigenous disadvantage. The targets agreed to by COAG relate to health or social determinants of health. For the target 'Closing the life expectancy gap within a generation (by 2031)', one of the performance indicators is the prevalence of overweight and obesity.<br><br>**Ireland**: **Step 9** of the Healthy Weight for Ireland: Obesity Policy and Action Plan 2016-2025 aims to, allocate resources to those population groups most

	in need of support in the prevention and management of obesity, with particular emphasis on families and children during the first 1,000 days of life. The priority actions to commence in first year were to, assess the needs of vulnerable groups as the basis of allocation of resources for preventative and treatment services for children and adults (Department of Health, 2016c). The Healthy Ireland fund was established in 2017 with an allocation of €5 million and with additional allocations of €5 million in 2018 and 2019. The first round of the Fund was distributed through Local Community Development Committees, Children and Young Person's Services Committees and statutory organisations. The Fund has been effective at targeting population groups that experience health inequalities. In Round 1 (2017/18), there was a focus on specific groups experiencing health inequalities, including people living in areas of social disadvantage (71% of actions), people with disabilities (45%), people from new communities including refugees and asylum seekers (39%) and members of the Traveller community (36%). Furthermore, of the local actions that were implemented in Round 1, 61% related to physical activity and 32% were related to food, nutrition and weight management. Round 2 of the Fund is currently being implemented (Oireachtas, 2019) The Healthy Ireland 2019 communications and citizen engagement campaign has continued on from 2018, launching on the 8th of April 2019, with an announced funding of €1 million to boost community engagement on health and wellbeing in every county (Department of Health, 2019d).
**Context** e.g. EU action/ regulation / food industry action etc.	**Safefood**<br><br>**START Campaign:** In late 2017, Safefood in partnership with the Department of Health and Public Health Agency in Northern Ireland (NI); and Healthy Ireland, Health Service Executive and Department of Health in the Republic of Ireland (ROI) launched a new 5-year public awareness campaign aimed at maintaining a healthy weight in children across the island of Ireland.<br>The campaign adopts a parenting approach that encourages role modelling and consistency. It offers families a number of key messages and practical, achievable tips to help make healthier lifestyle changes (Safefood, 2017).<br><br>The key campaign messages include:<br>• Minimise intake of foods high in fat, salt and sugar<br>• Establish water and milk as routine drinks<br>• Advocate appropriate child-sized portions<br>• Increase physical activity levels

	• Limit screen-time<br>• Increase sleep-time<br><br>The campaign is underpinned by obesity prevention policy documents in ROI and NI - A Healthy Weight for Ireland: Obesity Policy and Action Plan 2016 – 2025 and A Fitter Future for All 2012-2022. It was developed with parents to ensure the messages are relevant and realistic (Safefood, 2017).<br><br>**The Community Food Initiative- Safefood**<br><br>• The Community Food Initiative (CFI) programme funded by safefood aims to address diet related inequalities and overcome some of the problems faced by communities in eating healthy, affordable food. During the period 2010-2015, 17 projects were funded. The current CFI programme runs from 2016-2018 and focuses on supporting community food programmes across 13 health regions in Republic of Ireland and Northern Ireland with the aim of promoting food skills, planning, budgeting and cooking in low income areas (Safefood, 2018).<br>• There is a strong emphasis on shared learning and networking between the selected projects. The lessons learned from the programme contribute to informing and supporting policy initiatives in relation to food poverty in the community on the island of Ireland. During 2013-2015 the CFIs collectively resulted in:<br>- 9,381 individual attendances at one-off events such e.g. food taster sessions or single workshop.<br>- 7,635 participated in healthy eating events and in gardening pursuits<br>- 4,940 participated in cooking skills events.<br>- Reported outcomes included increased interest in food and its health effects; more experimenting with new foods and substitution of healthier foods; and enjoyment of growing produce.<br><br>**All-island Food Poverty Network**<br><br>• In 2009, safefood established a network in partnership with the Food Standards Agency Northern Ireland which involves representatives from Government departments and Agencies, academia and NGOs with the aim of ensuring a co-ordinated and strategic approach to

	tackling food poverty on the island of Ireland. The group works by developing consensus on related issues, collaborating and sharing learning (Safefood, 2018)<br><br>**Key achievements to date include:**<br><br>• Development of a food poverty measure for Republic of Ireland (ROI) and Northern Ireland (NI) pilot of food poverty indicator.<br>• Report on research into Food Basket costs in ROI (2014) and NI (2016)<br>• Food Poverty conferences in ROI (2014) and NI (2015 – 2017)<br>• Three all-island network meetings held annually.<br>• Development and publication of a bi-annual all-island Food Poverty e-newsletter.
**Evidence of implementation**	**Healthy Weight for Ireland: Obesity Policy and Action Plan 2016-2025**<br><br>• **Step 9** of the obesity policy and action plan aims to, allocate resources according to need, in particular to those population groups most in need of support in the prevention and management of obesity, with particular emphasis on families and children during the first 1,000 days of life. The priority actions to commence in first year were to, assess the needs of vulnerable groups as the basis of allocation of resources for preventative and treatment services for children and adults (Department of Health, 2016c).<br><br>• The Healthy Ireland fund was established in 2017 with an allocation of €5 million and with additional allocations of €5 million in 2018 and 2019. The first round of the Fund was distributed through Local Community Development Committees, Children and Young Person's Services Committees and statutory organisations. The aim of the Fund is to support innovative, cross sectoral, evidence-based projects, programmes and initiatives that support the implementation of key national policies in areas such as Obesity, Smoking Alcohol, Physical Activity and Sexual Health (Oireachtas, 2019).<br><br>• The Fund has been effective at targeting population groups that experience health inequalities. In Round 1 (2017/18), there was a

	focus on specific groups experiencing health inequalities, including people living in areas of social disadvantage (71% of actions), people with disabilities (45%), people from new communities including refugees and asylum seekers (39%) and members of the Traveller community (36%). Furthermore, of the local actions that were implemented in Round 1, 61% related to physical activity and 32% were related to food, nutrition and weight management. Round 2 of the Fund is currently being implemented (Oireachtas, 2019)<br><br>**Healthy Weight for Ireland: Obesity Policy and Action Plan 2016-2025**<br><br>• **Step 4:** The action plan also aims to implement a strategic and sustained communications strategy that empowers individuals, communities and service providers to become obesity aware and equipped to change, with a particular focus on families with children in the early years. The priority actions to commence in first year were to, develop a communications strategy to increase knowledge among the public and all other relevant stakeholders of the benefits of being a healthy weight and to stimulate healthy behaviour change. This included measures to communicate to disadvantaged and low income groups, including considering how best to link to measures already being taken by the Department of Social Protection (Department of Health, 2016b)<br><br>• The Healthy Ireland 2018 communications and citizen engagement campaign sought to encourage people to make small, healthy changes under the themes of Healthy Eating, Physical Activity and Mental Wellbeing, and to link them with partner organisations and initiatives providing information and support (Oireachtas, 2019). This campaign has continued and was launched on the 8th of April 2019, with an announced funding of €1 million to boost community engagement on health and wellbeing in every county. The 2019 campaign seeks to raise public awareness of the gov.ie/HealthyIreland website, as the place to go that brings trusted sources of information and supports together in one place to help people get started on making healthy changes (Department of Health, 2019d)

	**Health Service Executive: The Healthy Eating Active Living Policy Priority Programme - National Implementation Plan 2017-2020**<br><br>• A Healthy Eating, Active Living Programme has been established as a Policy Priority Programme within the HSE, to support the Healthy Ireland in Health Services Implementation Plan (Health Service Executive, 2015). A three-year plan for the programme has been finalised. It is supporting work in the education sector, as well as with parents, families and communities in delivering a more coordinated approach to prevention and early intervention in child obesity. The GP contract for the provision of free care to children under 6 years, already provides that the medical practitioner shall take an active approach toward promoting health and preventing disease through the provision of periodic assessments to child patients (health Service Executive, 2017b).<br><br>**Joint Initiative on Food Poverty**<br><br>• A Joint Initiative on Food Poverty relating to children and families, between Healthy Ireland, the Better Outcomes, Brighter Futures Advisory Council and the Department of Children and Youth Affairs, was launched in 2019 to report with a programme of actions in 2020 (Department for Children and Youth Affairs, 2014-2020)
**Comments/notes**	

DOMAIN 9 - GOVERNANCE: Governments have structures in place to ensure transparency and accountability, and encourage broad community participation and inclusion when formulating and implementing policies and actions to create healthy food environments, improve population nutrition, and reduce diet-related inequalities.

<table>
<tr><td colspan="2">GOVER1
There are procedures in place to restrict commercial influences on the development of policies related to food environments where they have conflicts of interest with improving population nutrition. for example: restricting lobbying influences.</td></tr>
<tr><td>Definitions

and scope</td><td>Includes government policies, guidelines, codes of conduct or other mechanisms to guide actions and decision-making by government employees, for example conflict of interest declaration procedures
Includes procedures to manage partnerships with private companies or peak bodies representing industries that are consulted for the purpose of developing policy, for examples committee procedural guidelines or terms of reference.
Includes publicly available, up to-to-date registers of lobbyist and/ or their activities</td></tr>
<tr><td>International best practice examples (benchmarks)</td><td>USA: Mandatory and publicly accessible lobby registers exist at the federal level, as well as in nearly every state. Financial information must be disclosed, and the register is enforced through significant sanctions. A number of pieces of legislation uphold compliance with the register including Lobbying Disclosure Act of 1995 and the Honest Leadership and Open Government Act 2007.

New Zealand: The State Services Commission has published Best Practice Guidelines for Departments Responsible for Regulatory Processes with Significant Commercial Implications. They cover the development and operation of a regulatory process and include specific references to principles around stakeholder relationship management (State Services Commission).

Australia: The Australian Public Service Commission's Values and Code of Conduct includes a number of relevant sections such as the Conflict of Interest, Working with the Private Sector and other Stakeholders and the Lobbying Code of Conduct.

Ireland: The Regulation of Lobbying Act 2015 was signed into law in March 2015. The purpose of the Act is to, provide for a web-based Register of</td></tr>
</table>

	Lobbying to make information available to the public on the identity of those communicating with designated public officials on specific policy, legislative matters or prospective decisions. In support of the Act's objectives to foster transparency and the proper conduct of lobbying activities, the Code of Conduct for persons carrying on lobbying activities was established. Its purpose is to govern the behaviour of persons carrying on lobbying activities. The provisions of the Act can apply to employers; to representative or advocacy bodies; to professional lobbyists or third parties who are being paid to communicate on behalf of a client or other person; and, significantly, to any person communicating about the development or zoning of land (Standards in Public Office Commission, 2019).
**Context** e.g. EU action/ regulation / food industry action etc.	**EU Transparency Register (European Commission)**<br>• The European Commission has set up a database of special interest groups whose goal is to influence policy and law-making at the European institutions. Registrants are bound by a Code of Conduct (Annex 3 of the Inter Institutional Agreement) (Europa, 2014a) which set out rules for all registrants and establishes the underlying principles for standards of behavior in all relations with the EU institutions.<br>• Commissioners, their cabinet members and Directors-General publish information on meetings held with organizations or self-employed individuals (European Commission). Meetings related to policy-making and implementation in the EU can only take place if the interest representatives are registered in the EU Transparency Register.
**Evidence of implementation**	**The Regulation of Lobbying Act 2015**<br>• The Regulation of Lobbying Act 2015 was signed into law in March 2015. The purpose of the Act is to, provide for a web-based Register of Lobbying to make information available to the public on the identity of those communicating with designated public officials on specific policy, legislative matters or prospective decisions. The Act also provides restrictions and conditions on the taking up of certain employments by certain designated officials, for a specified period of time where a possible conflict of interest arises. The Act provides that the Standards Commission will be the Regulator of Lobbying. The

	Standards Commission will oversee the implementation of the register, monitor compliance, provide guidance and assistance and where necessary investigate and pursue breaches of legal requirements in due course.<br><br>**The Regulation of Lobbying Act 2015**<br>• Part 4 of the Act, which provides for investigation and enforcement provisions, commenced on 1 January 2017<br>• 11th of June 2019 - Revised best practices for persons carrying on lobbying activities was published (Register of Lobbying, 2019b).<br>• 10th of May 2019 - Standards Commission published its submission to the second legislative review of the Regulation of Lobbying Act (Register of Lobbying, 2019a).<br>• 28th of November 2018 - The Commission launched the Code of Conduct for persons carrying on lobbying activities<br>• 1st of January 2019 - The Code of Conduct for persons carrying on lobbying activities came into effect under the lobbying act (Standards in Public Office Commission, 2019).<br><br>**The Code of Conduct for persons carrying on lobbying activities**<br>• In support of the Act's objectives to foster transparency and the proper conduct of lobbying activities, this Code of Conduct sets out several principles by which persons carrying on lobbying activities should govern themselves in the course of carrying out lobbying activities, namely:<br>1. Demonstrating respect for public bodies<br>2. Acting with honesty and integrity<br>3. Ensuring accuracy of information<br>4. Disclosure of identity and purpose of lobbying activities<br>5. Preserving confidentiality<br>6. Avoiding improper influence<br>7. Observing the provisions of the Regulation of Lobbying Act<br>8. Having regard to the Code of Conduct.<br>• The purpose of this Code is to govern the behaviour of persons carrying on lobbying activities. The provisions of the Act can apply to employers; to representative or advocacy bodies; to professional lobbyists or third parties who are being paid to communicate on behalf of a client or other person; and, significantly, to any person

	communicating about the development or zoning of land (Standards in Public Office Commission, 2019).
**Comments/notes**	

GOVER2<br>**Policies and procedures are implemented for using evidence in the development of food and nutrition policies**	
**Definitions**<br><br>**and scope**	• Includes policies, procedures or guidelines to support government employees in the use of evidence for policy development including best practice evidence review methodology (including types and strength of evidence needed) and policy implementation in the absence of strong evidence (where the potential risk or harms of inaction are great).<br>• Includes policies, procedures or guidelines that stipulate the requirements for the establishment of a scientific or expert committee to inform policy development.<br>• Includes the use of evidence-based models, algorithms and tools to guide policy development or within policy to guide implementation (e.g. nutrient profiling model)<br>• Includes government resourcing of evidence and research by specific units, either within or across government departments
**International best practice examples (benchmarks)**	**Australia:** The National Health and Medical Research Council Act 1992 (NHMRC Act) requires NHMRC to develop evidence-based guidelines. These national guidelines are developed by teams of specialists following a rigorous nine-step development process (Government of Canada, 2016).
**Context** e.g. EU action/ regulation / food industry action etc.	**The Institute of Public Health**<br><br>• The Institute of Public Health in Ireland have a Policy Team which support evidence-informed policy making at all stages of the policy cycle. The activities of the Policy team include evidence synthesis/reviews, consultations, health impact assessments and evaluations. The policy team responds to public consultations to maximise the impact of policies outside the health sector. The Policy team contributes to government policies on obesity, tobacco, breastfeeding, healthy workplaces and child wellbeing as well as contributing to EU funded projects relevant to public health (Institute of Public Health, 2019)
**Evidence of implementation**	**The Food Safety Authority of Ireland (FSAI)**<br><br>• The Food Safety Authority of Ireland (FSAI) is legally obliged to base its opinions on scientific grounds and to develop food standards on

	the basis of the best, most up-to-date scientific advice available. They also link with Healthy Ireland providing scientific recommendations on priority issues as required (Food Safety Authority of Ireland, 2018b)<br><br>• To aid the FSAI in risk assessment, which underpins risk management decisions, the current Scientific Committee was set up in 2016 in accordance with Article 34 of the Food Safety Authority of Ireland Act, 1998. The Committee is made up of scientists, from a variety of disciplines, working in a voluntary capacity. It has a major influence on policy decisions of the FSAI, including advice on the implementation and administration of food inspection services and on the nutritional value of food. It also provides clarity on scientific and technical issues relating to food safety and hygiene (Food Safety Authority of Ireland, 2018b).<br><br>**A Healthy Weight for Ireland: Obesity Policy and Action Plan 2016-2025**<br><br>• Action 1.8 under Step 1 of the OPAP, *“Embed multi-sectoral actions on obesity prevention with the support of government departments and public sector agencies”.* The Department of Health aims to develop proposals on the rollout of evidence-based fiscal measures to support healthy eating and lifestyles (Department of Health., 2016)
**Comments/notes**	

**GOVER3**<br>**Policies and procedures are implemented for ensuring transparency in the development of food and nutrition policies**	
**Definitions**<br><br>**and scope**	• Includes policies or procedures that guide the use of consultation in the development of food policy<br>• Includes policies or procedures to guide the online publishing of private sector and civil society submissions to government around the development of policy and subsequent government response to these.<br>• Includes policies or procedures to guide the online publishing of scoping papers, draft and final policies<br>• Include policies or procedures to guide public communications around all policies put forward but not progressed
**International best practice examples (benchmarks)**	**New Zealand:** Food Standards Australia New Zealand (FSANZ) is required by the Food Standards Australia New Zealand Act 1991 to engage stakeholders in the development of new standards. This process is open to everyone in the community including consumers, public health professionals, and industry and government representatives. FSANZ has developed a Stakeholder Engagement Strategy 2013-16 that outlines the scope and processes for engagement. Under the Stakeholder Engagement Priorities 2013-16, it outlined “maintain our open and transparent approach” as one of the first priorities (Food Standards Australia New Zealand, 2013).<br><br>**Norway:** The Public Administration Act provides general procedural rules for public administration. The Act regulates the administrative procedures when decisions are made, especially the rights of parties during the procedures. The Central Government Communication Policy contains the central goals and principles of the central government’s communication with citizens, businesses, organizations and other public sector activities. The goals of the central government communication policy state that the citizens shall: receive accurate and clear information about their rights, duties and opportunities; have access to information about central government activities; and be invited to participate in the formulation of policy, schemes and services (Norwegian Ministry of Local Government and Modernisation, 2019).
**Context** e.g. EU action/ regulation /	**EU Action**

food industry action etc.	• The general principle of transparency has been laid down in the Treaty on the Functioning of the European Union (Europa, 2012). Article 15 states that in order to promote good governance and ensure the participation of civil society, the Union's institutions, bodies, offices and agencies shall conduct their work as openly as possible.<br><br>• The Commission's Better Regulation Agenda was set up in 2015 to achieve better results, by opening up policy and law-making and listen more to people it affects (European Commission, 2015). Transparency in the preparation and reviewing of policies, in the legislative process and in public consultations is a very important theme of this Agenda.<br><br>• Regulations related to food and nutrition policies also include text phrases related to transparency. The Regulation on the provision of food information to consumers (European Union, 2011) and the General Food Law(European commission, 2002) prescribe that during the preparation, evaluation and revision of food (information) law an open and transparent public consultation shall be conducted. The Regulation on nutrition and health claims stipulates the establishment of a public register of claims for the sake of transparency (European Comission, 2006).
**Evidence of implementation**	**Transparency code**<br><br>• The Transparency Code was prepared in accordance with the Regulation of Lobbying Act 2015, which commenced on September 1st, 2015 (Department of Public Expenditure and Reform, 2015). The Transparency Code sets out how certain public bodies may conduct their activities in a transparent way. The Regulation of Lobbying Act 2015 aims to make the process of developing policies more transparent by providing for:<br>- The establishment and maintenance of a publicly accessible register of lobbying; Obligations on lobbyists to register and to provide information regularly about their lobbying activities, including, in the case of professional lobbyists, information about their clients.<br>- The introduction of a "cooling off" period during which lobbying activity may not be carried out by some former officials.

	- The Standards in Public Office Commission (The Standards Commission) to be the regulator of lobbying.<br>- As part of the Open Government Partnership National Action Plan 2014-2016, Ireland has committed to ‘greater citizen consultation and involvement to strengthen democracy and improve public services.
**Comments/notes**	

**GOVER4**<br>**The government ensures public access to comprehensive nutrition information and key documents (e.g. budget documents, annual performance reviews and health indicators) for the public**	
**Definitions**<br><br>**and scope**	• Includes policies and procedures to guide the timely, online publishing of government budgets, performance reviews, audits, evaluation reports or the findings of other reviews or inquiries<br>• Includes 'freedom of information' legislation and related processes to enable the public access to government information on request, with minimal restrictions and exemptions<br>• Includes policies or procedures to guide the timely, online publishing of population health data captured / owned by government
**International best practice examples (benchmarks)**	**New Zealand/Australia:** The freedom of information Act provides a legally enforceable right of the public to assess documents of government departments and most agencies.<br><br>**Ireland:** The Freedom of Information Act 2014 came into effect in October 2014 and repealed the 1997 and 2003 Acts. The 2014 Act now applies to all public bodies, unless they are specifically exempt. It also allows for the Government to prescribe (or designate) other bodies receiving significant public funds, so that the FOI legislation applies to them also. The old legislation continues to apply to any FOI request that was made before the 2014 Act came into effect. It also applies to any subsequent review or appeal. It provides the following statutory rights, (1) A legal right for each person to access information held by a body to which FOI legislation applies known as an *FOI body.* (2) A legal right for each person to have official information relating to himself/herself amended where it is incomplete, incorrect or misleading. (3) A legal right for each person to obtain reasons for decisions affecting himself/herself (Citizens Information, 2014).<br><br>**Norway:** The Freedom of Information Act grants everyone the right of access to case documents, journals and similar registers for any agencies encompassed by the Act. The Act applies to all government agencies, municipalities and county authorities. The general rule is that access shall be granted, and exceptions to this rule require legal authority prescribed by or pursuant to law. The Act also contains rules for handling right of access claims and the opportunity to appeal decisions in access matters (Norwegian Ministry of Local Government and Modernisation, 2019).
**Context** e.g. EU action/ regulation /	**EU Action**

food industry action etc.	• Regarding Public access to nutrition and health data, provisions have been included in several regulations. The Regulation on nutrition and health claims (European Comission, 2006) prescribes for example the establishment and maintenance of a Register on nutrition and health claims, which is available online. The General Food Law contains an article which prescribes that EFSA shall ensure wide access to the documents it possesses. With the revision of the General Food Law, citizens will have automatic access to all studies and information submitted by the industry in the risk assessment process.<br><br>• EUROSTAT is the main source of EU Health data. Health data is based on the Regulation on Community Statistics on public health and health and safety at work (No1338/2009) (European Comission, 2008). This Regulation prescribes that the Commission (Eurostat) shall take the necessary steps to improve the dissemination, accessibility and documentation of statistical information. Eurostat's dissemination policy is characterized by free access to European statistics for all users, which is together with other principles laid down in Regulation on European statistics (No 223/2009) and in the European Statistics Code of Practice.
**Evidence of implementation**	**The Reform of the Freedom of Information legislation**<br><br>• The Reform of the Freedom of Information legislation was announced by the Minister for Public Expenditure and Reform in 2012. The new Freedom of Information Act came into force in 2014, applying to all public bodies (Department of Public Expenditure and Reform, 2019)<br><br>**It provides the following statutory rights:**<br><br>- A legal right for each person to obtain reasons for decisions affecting himself/herself.<br>- A legal right for each person to access information held by a body to which Freedom of Information Legislation applies.<br><br>**A Freedom of Information body is required to:**<br><br>**1.** Publish information to assist members of the public in their understanding of body and its functions.

	2. Publish the information that it holds grouped under the information headings set out in the model publication scheme.<br>3. Explain the procedures to get access to information or to establish what information the body hold.<br><br>• Up-to-date information on a range of indicators including nutrition and diet-related indicators is made available to the public via the government websites: Various sources of data are available including survey data, annual reports and budget information https://health.gov.ie/.
**Comments/notes**	

DOMAIN 10 - MONITORING AND INTELLIGENCE: The government's monitoring and intelligence systems (surveillance, evaluation, research and reporting) are comprehensive and regular enough to assess the status of food environments, population nutrition and diet-related NCDs and their inequalities, and to measure progress on achieving the goals of nutrition and health plans.

**MONIT1**<br>**Monitoring systems, implemented by the government, are in place to regularly monitor food environments(especially for food composition for nutrients of concern, food promotion to children, and nutritional quality of food in schools and other public sector settings), against codes / guidelines / standards / targets**	
**Definitions and scope**	• Includes monitoring systems funded fully or in part by government that are managed by an academic institution or other organisation<br>• Includes regular monitoring and review of the impact of policies implemented by the government on food environments (as relevant to the individual State / Territory, and described in the policy domains above), in particular:<br>• Monitoring of compliance with voluntary food composition standards related to nutrients of concern in out-of-home meals (as defined in the 'Food composition' domain)<br>• Monitoring of compliance with food labelling regulations (as defined in the 'Food labelling' domain above)<br>• Monitoring of unhealthy food promoted to children via broadcast and non-broadcast media and in children's settings (as defined in the 'Food promotion' domain above)<br>• Monitoring of compliance with food provision policies in schools, early childhood services and public sector settings (as defined in the 'Food provision' domain above)
**International best practice examples (benchmarks)**	**Many countries:** have food composition databases available. For example, the New Zealand Institute for Plant & Food Research Limited and the Ministry of Health jointly own the New Zealand Food Composition Database (NZFCD) which is a comprehensive collection of nutrient data in New Zealand containing nutrient information on more than 2600 foods.<br><br>**New Zealand:** A national School and Early Childhood Education Services (ECES) Food and Nutrition Environment Survey was organised in all Schools and ECES across New Zealand in 2007 and 2009 by the Ministry of Health to measure the food environments in schools and ECEs in New Zealand.

	**UK:** In October 2005, the School Food Trust ('the Trust'; now called the Children's Food Trust) was established to provide independent support and advice to schools, caterers, manufacturers and others on improving the standard of school meals. They perform annual surveys, including the latest information on how many children are having school meals in England, how much they cost and how they're being provided.<br><br>**Germany:** The German Nutrition Report published by the DGE on behalf of the Federal Republic of Germany. The Nutrition Reports for the Federal Republic of Germany have been published by the DGE since 1969. Since 1972 it is provided every 4 years by directive of the Federal Ministry of Consumer Protection, Food and Agriculture. The subjects follow topics of current interest (German Nutrition Society).(German Nutrition Society). The Robert-Koch-Institute (RKI), the government's scientific institution in the field of public health, started in 2015 the project 'AdiMon' that collects regular data on influencing (environmental) factors and prevalence of childhood obesity, as well as interventions for prevention and health promotion (Robert Koch Institut).<br><br>**The Netherlands:** the progress in product improvement of salt, saturated fat and calories (sugar and/or (saturated) fat) is monitored by the Dutch Insititute of Public Health and Environment (Rijksinstituut voor Volksgezondheid en Milieu (RIVM)) at product level. RIVM uses the product databank (levensmiddelendatabank (LEDA) as basis for which companies have to provide information about product contents (Ministry of Public Health).
**Context** e.g. EU action/ regulation / food industry action etc.	**EU Action**<br><br>• According to the EU Action Plan on Childhood Obesity 2014-2020 (Europa, 2014c), Member States are responsible for implementing monitoring mechanisms like national food composition databases. The WHO is, according to the plan, responsible for collecting from the Member States on the monitored initiatives, e.g. via the WHO NOPA database and the WHO Health Behaviour among Schoolchildren and for Health-Promoting Schools (HBSC) surveys.<br><br>• Establishing a harmonized monitoring of school nutrition in the EU (primary and secondary schools) is an objective in the EU Action Plan on Childhood Obesity 2014-2020 (Europa, 2014c), for which Member States are according to the plan responsible. Additionally, it also

	refers to the WHO Health Behaviour among Schoolchildren and for Health-Promoting Schools (HBSC) surveys for monitoring. These surveys, which collect data every four years on 11-, 13- and 15-years old in 49 countries and regions, include questions around the school environment.<br><br>**Monitoring of unhealthy food promoted to children**<br>• Article 9 of the Audiovisual Media Directive (European Comission, 2018a), prescribes that Member States adopt code of conduct, which aim to reduce the exposure of children to unhealthy food promotion and which shall provide for regular, transparent and independent monitoring and evaluation of the objectives aimed at. In addition, these code of conducts shall also provide for effective enforcement including effective and proportionate sanctions.<br><br>• In addition to this, there are some monitoring initiatives at EU level: (1) DG Sante and DG CNECT launched in 2017 a study on the exposure of minors to TV and online marketing of unhealthy foods and (2) JRC was asked to produce (mid-2018) a mapping of initiatives to reduce marketing pressure (European Comission, 2019c)<br><br>**Monitoring of compliance with food composition standards**<br>• Although this has been designated by the EU as a Member State responsibility, EFSA has a food composition database as well (European Food Safety Authority). However, this database gives information on the amount of vitamins and minerals contained in foods but not on the nutrients of concern. The Consumers, Health, Agriculture and Food Executive Agency (Chafea) reserved funding to set up a feasibility study for a monitoring system on food reformulation initiatives for salt, sugars and fat. The tender for this feasibility study was closed February 2018 (Europa, 2017).
**Evidence of implementation**	**Food Safety Authority of Ireland (FSAI)**<br>• As part of their responsibility for verifying that the relevant requirements of food law are fulfilled by food business operators at all stages of production, processing and distribution, the FSAI and its

	agencies carry out monitoring and surveillance of food in the Irish market. These activities are undertaken in conjunction with the official agencies such as, the Health Service Executive, the Department of Agriculture, Food and the Marine, Local Authorities and the Sea Fisheries Protection Authority and their associated laboratories (Food Safety Authority of Ireland, 2014a)<br><br>**FSAI (2010) - Monitoring of Food Labelling**<br><br>• Monitoring of food labelling in Ireland is carried out to assess compliance with the labelling legislation and to check for authenticity of stated ingredients.<br>• In 2010, the FSAI carried out a survey to examine the accuracy of nutrition labelling on pre-packaged foods on the Irish market and to provide these data to the European Commission to inform their work in revision of nutrition labelling legislation. A secondary objective was to generate sufficient data on which to base national guidelines should a legislative route not be agreed at European level (Food Safety Authority of Ireland, 2010b)<br><br>**FSAI (2016) - Survey of the trans fatty acid content in processed food products in Ireland**<br><br>• Two previous surveys carried out by the Food Safety Authority of Ireland (FSAI) in 2007 and 2008 examined levels of TFA in a cross-section of processed foods (n=100) and fast foods (n=150) (Food Safety Authority of Ireland, 2008, Food safety Authority of Ireland, 2009). While both surveys concluded that the overall levels of TFA were low, i.e. <2 g TFA per/100 g fat, there were some foods which had high levels of TFA, as also identified in other published literature.<br><br>• During June 2016 the FSAI collected 240 branded and private label processed food products across all major supermarkets in Ireland. The samples comprised a cross-section of products grouped into 15 product categories. The latest FSAI survey indicates that overall levels of TFA in processed food products in Ireland are low and continue to decrease, with 97.5% of products having ≤2% TFA as a percentage of total fat. The results of this survey also verify the effectiveness of

	industry commitments and initiatives to reduce or exclude TFA from processed food products (Food Safety Authority of Ireland, 2016b).<br><br>**Nutrition Standards for School Meals**<br><br>• The standards were published under the auspices of ‘Healthy Ireland’, and will be implemented and monitored through the Department of Employment Affairs and Social Protection (Healthy Ireland, 2017a).<br><br>• Schools and organisations must reapply for funding in advance of each school year and are required to submit detailed records at the end of each school year. Expenditure on unhealthy food items is deducted from the following year’s funding allocation. The requirement to implement these nutrition standards is therefore largely met by that Department only funding healthy foods provided by schools under the school meals programme. In addition, the Department initiated an on-site inspection programme in 2012 in participating schools to ensure that the scheme rules are being adhered to. The inspection process is being rolled out across all schools and includes an assessment of the type of food provided. All schools participating in the School Meals Scheme are expected to comply with the standards from September 2018. Compliance with the new standards will form part of the inspection process from the 2018/2019 academic year (Department of Employment Affairs and Social Protection, 2018).
**Comments/notes**	

**MONIT2**<br>**There is regular monitoring of adult and childhood nutrition status and population intakes against specified intake targets or recommended daily intake levels**	
**Definitions and scope**	• Includes monitoring of adult and child intake in line with X Countries Food Guide and dietary recommendations<br>• Includes monitoring of adult and child intake of nutrients of concern and noncore/discretionary foods including sugar-sweetened beverages (even if there are no clear intake targets for all of these)<br>• 'Regular' is considered to be every five years or more frequently
**International best practice examples (benchmarks)**	**USA:** The National Health and Nutrition Examination Survey (NHANES) is a program of studies designed to assess the health and nutritional status of adults and children in the United States. The survey is unique in that it combines interviews and physical examinations (Centres for Disease Control, 2016) (Centres for Disease Control, 2016). The NHANES program began in the early 1960s and has been conducted as a series of surveys focusing on different population groups or health topics. In 1999, the survey became a continuous program that has a changing focus on a variety of health and nutrition measurements to meet emerging needs. The survey examines a nationally representative sample of about 5,000 persons each year. These persons are located in counties across the country, 15 of which are visited each year.<br><br>**The Netherlands:** The Dutch Institute of Public Health and Environment (Rijksinstituut voor Volksgezondheid en Milieu (RIVM)) is appointed by the Ministry of Health to periodically collect data about the food consumption and food condition of the Dutch population in general and of separate population groups via the Food Consumption Survey. Currently, a Food Consumption Survey (Dutch population 1-79 years) is being conducted for the years 2019-2021. Prior Food Consumption Surveys have been conducted for the years 2012-2016 (Dutch population 1-79 years), 2010-2012 (elderly 70+), 2007-2010 (7-69 years), 2005-2006 (2-6 years), 2003 (9-16 years) (National Institute for Public Health and the Environment Ministry of Health, 2011)
**Context** e.g. EU action/ regulation / food industry action etc.	• While there is no formal surveillance programme agreed on the island of Ireland, many surveys and initiatives contribute to nutrition surveillance in Ireland. Most of the activities have emerged from research and they have been 'one-off surveys' that aren't strictly surveillance.

	**EU Action**<br><br>**WHO - The health behaviour in School**<br><br>• The WHO HBSC surveys, which collect data every four years on 11-, 13- and 15-years old in 49 countries and regions, include questions around areas like eating behaviours, obesity, the socio- economic environment and the school environment.
**Evidence of implementation**	**The National Nutritional Surveillance Centre (NNSC)**<br><br>• The National Nutritional Surveillance Centre (NNSC) is required by the Health Service Executive to provide an independent source of information and advice and has the responsibility for collection and collation of available data relating to nutrition and health. Food and/or nutrition surveillance is based on the use of routinely collected or easily generated data from several sectors related to nutrition and health. These include: Food supply/ production/retail, Food consumption data, Nutritional knowledge, attitudes and beliefs, Information on effective nutrition programmes, Socio-economic indicators related to health, Nutrition related health status indicators, Food economics (National Nutrition Surveillance Centre).<br><br>• The aims of the NNSC include: To monitor trends in health status correlated with all aspects of the food chain and advice on these findings for health planners, to provide a source of information and research expertise, particularly in nutritional epidemiology and surveillance methodology to those wishing to mount specific projects such as micro-surveys. The reports produced by the NNSC have been widely disseminated among Health Board personnel, food agencies, producers, retailers, caterers, academics, teachers and the media (National Nutrition Surveillance Centre).<br><br>**Healthy Ireland Survey**<br><br>• Healthy Ireland survey is an annual interview administered face-to-face survey commissioned by the Department of Health, with a representative sample of the population aged 15 and older living in Ireland. It gathers information on the proportion of the population who drink sugar sweetened drinks daily, the proportion of the population who eat at least 5 portions of fruit and vegetables daily,

	the weight of people in the population, and also information on general health and utilisation of health services. Survey data play a number of roles, including supporting the Department in on-going engagement and awareness-raising activities in the various policy areas and supporting better understanding of policy priorities (Healthy Ireland & Department of Health, 2019).<br><br>**The health behaviour in School – Aged Children**<br><br>• Health Behaviour in School-aged Children (HBSC) is a cross-national research study conducted in collaboration with the World Health Organization (WHO) Regional Office for Europe. The study aims to gain new insight into, and increase our understanding of young people's health and well-being, health behaviours and their social context. In addition, the findings from the HBSC surveys are used to inform and influence children's policy and practice at national and international levels (National University of Ireland).<br><br>• In Ireland, the study is conducted by researchers in the Health Promotion Research Centre at the National University of Ireland, Galway. The network collects data every 4 years on 11-, 13-, and 15-year- old boy's and girl's health and well-being, social environments and health behaviours. This research collaboration brings in individuals with a wide range of expertise in areas such as clinical medicine, epidemiology, human biology, paediatrics, pedagogy, psychology, public health, public policy, and sociology. HBSC's findings show how young people's health changes as they move from childhood, through adolescence into adulthood. Member countries and stakeholders at national and international levels use this data to monitor young people's health, understand the social determinants of health, and determine effective health improvement interventions (National University of Ireland).
**Comments/notes**	

<table>
<tr><th colspan="2">MONIT3
There is regular monitoring of adult and childhood overweight and obesity prevalence using anthropometric measurements</th></tr>
<tr><td>Definitions

and scope</td><td>• Anthropometric measurements include height, weight and waist circumference
• ‘Regular’ is considered to be every five years or more frequently</td></tr>
<tr><td>International best practice examples (benchmarks)</td><td>UK: England’s National Child Measurement Programme was established in 2006 and aims to measure all children in England in the first (4-5) years and last (10-11 years) of primary school. In 2011-2012, 565 662 children (4-5 years) and 491118 children (10-11 years) were measured (Health and Social Care Information Centre, 2016).

Ireland: The 4th Childhood Obesity Surveillance Initiative report was launched in 2017. COSI collects data from children in primary schools in the Republic of Ireland. The survey is carried out periodically. Data was first collected from children in 2008 in first class and again in 2010 from first class and third class, in 2012 from first, third and fifth classes and in 2015 from first, fourth and sixth class. Trained researchers collected weight, height and waist circumference measurements. These figures were used to examine prevalence of normal weight, overweight, obesity and mean BMI (National nutrition Surveillance Centre).</td></tr>
<tr><td>Context e.g. EU action/ regulation / food industry action etc.</td><td>EU Action

WHO COSI, NOPA database and WHO HBSC surveys

In the EU Action Plan on Childhood Obesity 2014-2020, the EU refers to the WHO, as being responsible for collecting from the Member States on the monitored initiatives, e.g. via the WHO COSI, WHO NOPA and the WHO HBSC surveys (Europa, 2014c).

The WHO European Childhood Obesity Surveillance Initiative (COSI), measures trends in overweight and obesity among primary school aged children (6-9 years). It’s a survey based on nationally representative samples and takes standardized weight and height measurements and collects information on school environments and dietary habits (World Health Organisation).</td></tr>
</table>

	The WHO NOPA database compiles information to monitor progress on nutrition, diet, physical activity and obesity. The Country information contains surveillance data, policy documents, action to implement policy and examples of good practice in programmes and interventions (World Health Organisation).<br><br>These WHO HBSC surveys, which collect data every four years on 11-, 13- and 15-years old in 49 countries and regions, include questions around areas like eating behaviours, obesity, the socio- economic environment and the school environment (Health Behaviour in School-aged Children).
**Evidence of implementation**	**Healthy Ireland Survey**<br><br>• The Healthy Ireland Survey is an annual interviewer-administered face-to-face survey commissioned by the Department of Health. It is part of the Healthy Ireland Framework to improve the health and wellbeing of people living in Ireland. Survey data play a number of roles, including supporting the Department in ongoing engagement and awareness-raising activities in the various policy areas and supporting better understanding of policy priorities. The most recent survey contained a representative sample of 7,701 people aged 15 and older living in Ireland were interviewed between September 2017 and June 2018. As part of the survey, overweight and obesity level in the country are measured every 2 years (Healthy Ireland & Department of Health, 2019).<br><br>**Childhood Obesity Surveillance Initiative report**<br><br>• The COSI survey funded by the Health Service Executive (HSE), is a World Health Organization (WHO) led project that is part of a pan-European initiative. It is an ongoing, systematic process of collection, analysis, interpretation and dissemination of descriptive information for monitoring obesity, being identified as a serious public health problem in the WHO European Region and for use in programme planning and evaluation (National nutrition Surveillance Centre).<br>• The 4th Childhood Obesity Surveillance Initiative report was launched in 2017. COSI collects data from children in primary schools in the Republic of Ireland and is carried out periodically. Data was first

collected from children in 2008 in first class and again in 2010 from first class and third class, in 2012 from first, third and fifth classes and in 2015 from first, fourth and sixth class. Trained researchers collected weight, height and waist circumference measurements. These figures were used to examine prevalence of normal weight, overweight, obesity and mean BMI (National nutrition Surveillance Centre).

Growing up in Ireland: National Longitudinal Study of Children

- The Growing Up in Ireland study started in 2006 as the national longitudinal study of children in Ireland. It is funded by the Government of Ireland through the Department of Children and Youth Affairs (DCYA) with a contribution (in Phase 2) from The Atlantic Philanthropies. It is overseen and managed by the DCYA in conjunction with the Central Statistics Office and an inter-departmental Project Team and Steering Group. It is implemented by a consortium of researchers led by the Economic and Social Research Institute (ESRI) and Trinity College Dublin (Department of Children and Youth Affairs, 2018b).
- The growing up in Ireland study, the lives of 13-year-olds (2018), presented a broad, comprehensive overview of the lives of the Child Cohort at age 13 and to describe how they are faring in important areas of their lives. This included a section on weight status and activities (Department of Children and Youth Affairs, 2018b).

A Healthy Weight for Ireland: Obesity Policy and Action Plan 2016 – 2025

- Action 6.1 of the Obesity Policy and Action Plan provides for the inclusion of obesity prevention and care as part of the General Practitioner Contract. Under the GP contract for the provision of care to children under 6 years of age, medical practitioners are required to take an active approach toward promoting health and preventing disease through the provision of periodic assessments to child patients. Assessments involve the recording of age, gender, weight and height, and plotting on a centile chart at ages two and five, and taking appropriate follow-up action, including where appropriate, provision of health promotion advice, brief intervention and support, or referral to specialist services.(Department of Health, 2016b)

**Comments/notes**	

**MONIT4**<br>**There is regular monitoring of the prevalence of NCD metabolic risk factors and occurrence rates (e.g. prevalence, incidence, mortality) for the main diet-related NCDs**	
**Definitions**<br><br>**and scope**	• Diet-related NCD risk factors and NCDs include, amongst others, hypertension, hypercholesterolemia, Type 2 Diabetes, cardiovascular disease (including ischaemic heart disease, cerebrovascular disease and other diseases of the vessels), diet-related cancers.<br>• disease and other diseases of the vessels), diet-related cancers.<br>• May be collected through a variety of mechanisms such as population surveys or a notifiable diseases surveillance system.
**International best practice examples (benchmarks)**	• **OCED Countries:** Most of these countries have regular and robust prevalence, incidence and mortality data for the main diet related NCDs and NCD risk factors.
**Context** e.g. EU action/ regulation / food industry action etc.	• The WHO has started the Integrated surveillance on NCD's project in 2013 which was co-financed by the EU. The project aimed to describe how Member States of the European Union can optimize their use of the ECHI indicators to report on progress towards reaching the nine global targets of the Global Monitoring Framework on NCDs including the targets:<br>• 25% relative reduction in the overall mortality from cardiovascular diseases, cancer, diabetes, or chronic respiratory diseases.<br>• 30% relative reduction in mean population intake of salt/sodium.<br>• A 25% relative reduction in the prevalence of raised blood pressure or contain the prevalence of raised blood pressure, according to national circumstances - Halt the rise in diabetes & obesity.
**Evidence of implementation**	• Ireland has an operational NCD unit under the chief medical officer's unit in DOH
**Comments/notes**	

**MONIT5**<br>**Major programs and policies are regularly evaluated to assess their effectiveness and contributions to achieving the goals of the nutrition and health plans**	
**Definitions**<br><br>**and scope**	• Includes any policies, guidelines, frameworks or tools that are used to determine the depth and type (method and reporting) of evaluation required<br>• Includes a comprehensive evaluation framework and plan that aligns with the key preventive health or nutrition implementation plan<br>• The definition of a major programs and policies is to be defined by the relevant government department<br>• Evaluation should be in addition to routine monitoring of progress against a project plan or program logic
**International best practice examples (benchmarks)**	**USA:** The National Institutes for Health (NIH) provide funding for rapid assessments of natural experiments. The funding establishes an accelerated review/award process to support time-sensitive research to evaluate a new policy or program expected to influence obesity related behaviours (e.g., dietary intake, physical activity, or sedentary behaviour) and/or weight outcomes in an effort to prevent or reduce obesity (US National Institutes of Health, 2016).<br><br>**The Netherlands:** The Dutch Insititute of Public Health and Environment (Rijksinstituut voor Volksgezondheid en Milieu (RIVM)) conducted in 2017 a midterm evaluation to calculate the effect of the agreed maximum norms for salt and sugar in the Agreement on Product Improvement (The Dutch Insititute of Public Health and Environment 2017). A midterm evaluation has been performed to calculate the effect of the agreed maximum norms for salt and sugar reduction, and four scenarios have been calculated with the Food Consumption Survey.<br><br>**Ireland:** Under the 'Healthy Weight for Ireland', Obesity Policy and Action Plan 2016-2025, a new Obesity Policy Implementation Oversight Group (OPIOG) was established in October 2017 and a progress report on each recommendation in the OPAP is currently being finalised under the aegis of the OPIOG (Oireachtas, 2019). As set out in Healthy Ireland, integrated health and social impact assessments will be conducted on relevant policy areas to support other government departments in contributing towards the prevention of overweight and obesity.
**Context** e.g. EU action/ regulation /	**EU Action**

food industry action etc.	• In the Strategic Plan of DG Sante 2016-2020 it is emphasized that Better Regulation is a key horizontal priority for the Commission (European Comission, 2016b). Impact assessments, public consultations, ex-post evaluations and Fitness Checks are instruments for the Commission to ensure the regulatory cycle is transparent and of good quality. The Commission published Guidelines on evaluation and Fitness Checks (Europa). A Fitness Check is a type of evaluation that assesses several related actions like the interaction of laws, policies and programmes.<br><br>Evaluations or fitness checks that have been completed or are in progress, are:<br>- Monitoring the activities of the EU Platform for Action on Diet, Physical Activity and Health (annual monitoring reports)<br>- Evaluation of the implementation of the Strategy for Europe on Nutrition, Overweight and Obesity related issues (covering 2007-2011, report published in 2013)<br>- Mid-term evaluation of the third Health Programme 2014-2020 (covering 2016-2017)<br>- REFIT Evaluation of the General Food Law (published in 2018)<br>- Supporting the mid-term evaluation of the EU Action Plan on Childhood Obesity 2014-2020 (covering 2014-2017, published in 2018)<br>- REFIT: Evaluation of the Regulation on nutrition and health claims (started in 2016, in progress)<br>- Monitoring of the EU Fruit and Vegetable Scheme (countries provide annual monitoring reports, Evaluation reports cover five school years and will be available from 1 March 2023)
**Evidence of implementation**	**Food Safety Authority of Ireland (FSAI)**<br><br>• **<u>Labelling:</u>** monitoring of food labelling in Ireland is carried out to assess compliance with the labelling legislation and to check for authenticity of stated ingredients. For e.g. The objective of this survey was to examine the accuracy of nutrition labelling on pre-packaged foods on the Irish market and to provide these data to the European

	Commission to inform their work in revision of nutrition labelling legislation. A secondary objective was to generate sufficient data on which to base national guidelines should a legislative route not be agreed at European level (Food Safety Authority of Ireland, 2010a).<br>• **Nutrition:** the FSAI produce reports on surveys of the trans fatty acid content in processed food products in Ireland.<br><br>**Government monitoring of voluntary codes of practice**<br><br>• Regarding the issued set of voluntary codes of practice aimed at limiting the promotion, marketing and sponsorship of foods high in fats, sugar and/or salt (HFSS foods). The government of Ireland has committed to monitor compliance, and work is continuing on the development of a monitoring mechanism.
**Comments/notes**	

**MONIT6**<br>**Progress towards reducing health inequalities or health impacts in vulnerable populations and social and economic determinants of health are regularly monitored.**	
**Definitions**<br><br>**and scope**	• Monitoring of overweight and obesity and main diet-related NCDs includes stratification or analysis of population groups where there are the greatest health inequalities including Indigenous peoples and socio-economic strata<br>• Includes reporting against targets or key performance indicators related to health inequalities
**International best practice examples (benchmarks)**	**New Zealand:** All annual Ministry of Health Surveys report estimates by subpopulations in particular by ethnicity (including Maori and Pacific peoples), by age, by gender, and by New Zealand area deprivation.
**Context** e.g. EU action/ regulation / food industry action etc.	**Safefood - Food on a low income report**<br><br>• The aim of this research was to investigate the everyday experiences of food on a low income among people in four household types on the island of Ireland (IOI). This research will inform safefood and other stakeholders targeting vulnerable groups. It should influence both policy and practical programmes such as community food initiatives and awareness campaigns (Safefood, 2011).<br>• The All-Island Food Poverty Network, chaired by safefood and the Food Standards Agency of Northern Ireland, was set up to support a coordinated and strategic approach to tackling food poverty on the island of Ireland through the development of consensus on related issues, collaboration and shared learning.<br><br>**Irish Medical Organisation – Position Paper on Health Inequalities**<br><br>• The Irish Medical Organisation (IMO) highlights the social, economic and environmental factors affecting the health of their patients, factors recognised by all IMO doctors- whether working in a hospital, general practice, or public or community health setting.<br>• Analyses The All Ireland Traveller Health Study as well as the Prevalence of Chronic Conditions Ireland – including the prevalence of chronic conditions in more deprived areas. (Irish Medical organisation, 2012).

**Evidence of implementation**	**Healthy Ireland Survey**<br><br>• The Healthy Ireland Survey 2018, for the first time measured and addressed health inequalities in Ireland and will continue to monitor inequalities annually. Results found that, those in more deprived areas are less likely to rate their health as good or very good, and more likely to have a long-term health problem. Similarly, those in more deprived areas are more likely to smoke and binge drink. Self-rated good health declines earlier for men in deprived areas (around the age of 55 to 64) than it does for both men in affluent areas and women in deprived areas. Lastly, women living in more deprived areas are more likely than those in affluent areas to continue smoking and binge drinking into their 50s (Healthy Ireland, 2018).<br><br>**Healthy Ireland Fund**<br><br>• In 2016, the Government approved the creation of a Healthy Ireland Fund, with an allocation of €5 million approved in Budget 2017 and 2018 to establish and support the implementation of Healthy Ireland programmes and projects in a variety of settings. The new round will run from 01 April 2018 to 31 March 2019. The funding goes towards a number of local projects in Ireland for socially disadvantaged groups including travellers and homeless families.<br><br>• The new structures established under Healthy Ireland to enable and support its implementation will provide a focus on addressing the health needs of all groups experiencing health inequalities, including travellers, and provides an opportunity to take a new approach to tackling issues such as health inequalities and the social determinants of health more effectively. The All-Ireland Traveller Health Study (AITHS) was a large-scale study focusing on key aspects of traveller health, social status and service utilisation (Government of Ireland, 2016).
**Comments/notes**	

DOMAIN 11 - FUNDING AND RESOURCES: Sufficient funding is invested in 'Population Nutrition Promotion' (estimated from the investments in population promotion of healthy eating and healthy food environments for the prevention of obesity and diet-related NCDs, excluding all one-on-one promotion (primary-care, antenatal services, maternal and child nursing services etc.), food safety, micronutrient deficiencies (e.g. folate fortification and undernutrition) to create healthy food environments, improved population nutrition, reductions in obesity, diet-related NCDs and their related inequalities.

**FUND1**<br>**The 'population nutrition' budget, as a proportion of total health spending and/or in relation to the diet-related NCD burden sufficiently contributes to reducing diet-related NCD's.**<br>**(Not being included for benchmarking)**	
**Definitions and scope**	• 'Population nutrition' includes promotion of healthy eating, and policies and programs that support healthy food environments for the prevention of obesity and diet-related NCDs<br>• The definition excludes all one-on-one and group-based promotion (primary care, antenatal services, maternal and child nursing services etc.), food safety, micronutrient deficiencies (e.g. folic acid fortification) and undernutrition<br>• Includes estimates for the budget allocated to the unit within the Department of Health that has primary responsibility for population nutrition.<br>• The workforce comprises anyone whose primary role relates to population nutrition and who is employed full time, part time or casually by the Department of Health or contracted by the Department of Health to perform a population nutrition-related role (including consultants or funding of a position in another government or nongovernment agency). The number of full time equivalent persons in the workforce will be reported in 'FUND4<br>• Excludes budget items related to physical activity promotion
**International best practice examples (benchmarks)**	**New Zealand:** The total funding for population nutrition was estimated at about $67 million or 0.6% of the health budget during 2008/09 Healthy Eating Healthy Action period. Dietary risk factors account for 11.4% of health loss in New Zealand.

	**Thailand:** According to the most recent report on health expenditure in 2012 the government greatly increased budget spent on policies and actions related to nutrition (excluding food, hygiene and drinking water control). Total expenditure on health related to nutrition specifically from local governments was 29,434.5 million Baht (7.57% of total health expenditure from public funding agencies), which was ten times over the budget spending on nutrition in 2011. Dietary risk factors account for more than 10% of health loss in Thailand
**Context** e.g. EU action/ regulation / food industry action etc.	**EU Action**<br><br>• Expenditure of the Health Programme is directly managed by DG SANTE and the Consumer, Health and Food Executive Agency (CHAF-EA) (European Comission, 2016b). The Commission also receives additional funding from the European Parliament for pilot projects to improve future policy action in the area of nutrition and physical activity and to identify good practices.<br><br>• For the EU Fruit and Vegetable Scheme which falls into the first pillar of the CAP (direct payments and market measures, 312,735 million euros) (European Comission), is a maximum of 150 million euros per school year available for requesting Member States, taking into account their number of six-to ten-year-old children and the degree of development of their regions (European Parliment, 2019). All Member States except Sweden and the UK have received EU Fruit and Vegetable Scheme funds for the 2017-2018 and 2018-2019 school years.<br><br>• Next to the Public Health Programme, the EU provides other funds to promote investing in health as a broader means of achieving smart and inclusive growth. These funds are the European and Structural Investment Funds (ESIF) and the European Fund for Strategic Investments (EFSI) (European Comission). The health sector receives ESIF for investments through the European Regional Development Fund and the European Social Fund. For 2014-2020 more than 9 billion euros was foreseen in all Member States for health-related investments (European Comission). EFSI is partnership of the Commission and the EIB, providing a financial guarantee to the value

	of 315 billion euros over a three year period and with an extension up to 500 billion euros until 2020 (European Comission).<br><br>• For the next Multiannual Financial Framework 2021-2027, the European Social Fund Plus Programme will be the main investment fund for investing in Health (European Comission). This programme merges existing funds like the Health Programme, The European Social Fund (ESF) and the Youth Employment Initiative (YEI), The Fund for European Aid to the Most Deprived (FEAD) and The Employment and Social Innovation (EaSI) programme. For the Health strand an amount of 413 billion euros will be available.
**Evidence of implementation**	**Government Expenditure Report 2018**<br><br>• In 2018, the funding level available to health reached €16.2 billion. The 2018 allocation marked a record as the highest in the history of the State. From 2016 onwards the Budget provided increased the annual allocation and on average provided an additional €431m annually to health. Despite this increase, the health sector continued to overspend and the supplementary funding provided ranged from €195m to around €645m(Government of Ireland, 2018).
**Comments/notes**	

FUND2<br>**Government funded research is targeted for improving food environments, reducing obesity, NCDs and their related inequalities**	
**Definitions and scope**	• Includes the clear identification of research priorities related to improving food environments, reducing obesity, NCDs and their related inequalities in health or medical research strategies or frameworks<br>• Includes identifying research projects conducted or commissioned by the government specifically targeting food environments, prevention of obesity or NCDs (excluding secondary or tertiary prevention)<br>• It is limited to research projects committed to or conducted within the last 12 months<br>• Excludes research grants administered by the government (including statutory agencies) to a research group where the allocation of a pool of funding was determined by an independent review panel<br>• Excludes evaluation of interventions (this is explored in 'MONIT5' and should be part of an overall program budget)
**International best practice examples (benchmarks)**	**Australia:** The National Health and Medical Research Council (NHMRC) Act requires the CEO to identify major national health issues likely to arise. The National Health Priority Areas (NHPAs) articulate priorities for research and investment and have been designated by Australian governments as key targets because of their contribution to the burden of disease in Australia. For the 2015-16 Corporate Plan, obesity, diabetes and cardiovascular health are three of these NHPAs.<br><br>**Thailand:** The National Research Council funded more research projects on obesity and diet-related chronic diseases (such as diabetes, cardiovascular diseases and hypertension) in 2014, accountable for almost six times over the research funding in 2013 (from 6,875,028 baht in 2013 to 37,872,416 baht in 2014)<br><br>**Ireland:** The Food Institutional Research Measure (FIRM) is funded by the Department of Agriculture, Food and the Marine and is the primary national funding mechanism for food research in higher education institutions and other public research institutes. Beneficiaries are required to widely disseminate the results of their research. FIRM aims to develop public good technologies that will underpin a competitive, innovative and sustainable food manufacturing and marketing sector. The programme is creating a base of knowledge and expertise in generic technologies that will support a modern, consumer-focused industry and build Ireland's capacity for R&D

	(Marine, 2017).(Marine, 2017). The Health Research Board (HRB) is a statutory agency under the aegis of the Department of Health. It's the lead agency in Ireland responsible for supporting and funding health research, information and evidence, which aims to improve people's health and to enhance healthcare delivery (Health Research Board, 2016).
**Context** e.g. EU action/ regulation / food industry action etc.	**EU Action**<br><br>• The European Union's strategy 2020 for smart, sustainable and inclusive growth, sets the strengthening of research and innovation as one of its main objectives. The implementation of the Innovation Union (to create jobs and growth by improving conditions and access to finance for research and innovation) (Marie Curie Alumni Association), including the European Research Area (European Comission) are part of this Strategy. Horizon 2020 and the Joint Programming Initiative 'A Healthy Diet for a Healthy Life' are research programmes contributing to a fully operational European Research Area on the prevention of diet-related diseases '.<br><br>• Horizon 2020 invests in innovative solutions that help tackle the adverse effects on human health, with funding of nearly 80 billion euros available (2014-2020) (European Comission)'Food and Healthy Diet' is marked as an area of Horizon 2020, with issues as 'promoting informed consumer choices' and delivering strategic solutions for healthy and safe foods and diets for all' (European Comission). An example of a project which has received funding from Horizon 2020 is 'FOOD 2030' which addresses Nutrition for sustainable and healthy diets.<br><br>• The Joint Programming Initiative 'A Healthy Diet for a Healthy Life' started in 2014 and is voluntary partnership between Member States and Associated Countries of the European Union, to address societal challenges as Health and Nutrition (JPI a healthy diet for a healthy life). This includes research to increase the understanding of health-impacting behavior with respect to making food choices, to create insight in how the environment influences this behavior and to raise consumer understanding of healthy foods and diets. One of the

	projects financed by this JPI is the 'Policy-Evaluation Network (PEN)- Public policies addressing health- related behaviours in Europe'.
**Evidence of implementation**	**Healthy Ireland, A Framework for Improved Health and Wellbeing 2013-2025**<br><br>• Healthy Ireland, A Framework for Improved Health and Wellbeing 2013-2025 is the national framework for action to improve the health and wellbeing of Ireland over the coming generation. Pobal administer this fund on behalf of the Department of Health (Healthy Ireland, 2019).<br><br>• In 2016, the Government approved the creation of a Healthy Ireland Fund with an initial allocation of €5 million approved in Budget 2017 to establish and support the implementation of Healthy Ireland programmes and projects in a variety of settings. The primary aim of the fund is to support innovative, cross-sectoral, evidence-based projects and initiatives that support the implementation of key national policies in areas such as obesity, smoking, alcohol, physical activity and sexual health. The funding has been allocated to 2 Strands, both of which are closed calls for funding. There will be a new round of funding in 2018. The new round will run from 01 April 2018 to 31 March 2019. Strand 1 focuses on the delivery of local initiatives and Strand 2 funds national actions (Healthy Ireland, 2019).
**Comments/notes**	

**FUND3**<br>**There is a statutory health promotion agency in place that includes an objective to improve population nutrition with a secure funding stream**	
**Definitions**<br><br>**and scope**	• Agency was established through legislation<br>• Includes objective to improve population nutrition in relevant legislation, strategic plans or on agency website<br>• Secure funding stream involves the use of a hypothecated tax or other secure source
**International best practice examples (benchmarks)**	**Australia:** The Victorian Health Promotion Foundation (VicHealth) was the world's first health promotion foundation, established by the Victorian Parliament as part of the Tobacco Act of 1987 (for the first 10 years through a hypothecated tobacco tax) through which the objectives of VicHealth are stipulated. VicHealth continues to maintain bipartisan support.<br><br>**Germany:** The **Federal Centre for Health Education** and the Federal Center for Nutrition disseminate guidelines and health promotion strategies to the general public and stakeholders and multipliers<br><br>**The Netherlands:** The Dutch Nutrition Center (https://www.voedingscentrum.nl/nl/service/over-ons.aspx) is 100% funded by the government and offers consumers and professionals scientific and independent information about a healthy, safe and sustainable food choice. The famous 'Wheel of Five' Guidelines is one of their products.
**Context** e.g. EU action/ regulation / food industry action etc.	**EU Action - Statutory health promotion agency**<br><br>• At EU level there is a Steering Group on Health Promotion, Disease Prevention and Management of Non-Communicable Diseases, which was set up in July 2018 (European Comission, 2018b). This Steering Group assists and advises the Commission on for example the coordination between Member States in addressing challenges caused by NCD's, the selection of best practices regarding health promotion, disease prevention and management of NCDs and monitoring progress towards reducing mortality due to NCD's
**Evidence of implementation**	**Healthy Ireland**<br><br>• Healthy Ireland is a Government-led initiative which aims to create an Irish society where everyone can enjoy physical and mental health, and where wellbeing is valued and supported at every level of society.

	● Resources such as the HSE are currently working on the ground on 'A Healthy weight for Ireland Obesity Policy and Action Plan 2016-2025'. The Department of Health will be the main source of management in relation to this action plan, while working closely with a number of other key stakeholders. These leads include:<br><br>• Department of Agriculture, Food and the Marine<br>• Department of Children and Youth Affairs<br>• Department of Education and Skills<br>• Department of Health<br>• Department of Transport, Tourism and Sport<br>• Food and Drink Industry Ireland<br>• Food Safety Authority of Ireland<br>• Health Information Quality Authority<br>• Health Research Board<br>• Health Service Executive<br>• Irish Business and Employers' Confederation<br>• Safefood<br><br>**HSE – Health and Wellbeing division**<br><br>● The Health and Wellbeing Division of the HSE is focused on helping people to stay healthy and well, reducing health inequalities and protecting people from threats to their health and wellbeing. It was established based on two fundamental policy shifts within the health service – Future Health, which describes the new structures currently being established for the healthcare system, and Healthy Ireland, the Government Framework to improve the health and wellbeing of our population. The services within Health and Wellbeing support people and communities to protect and improve their health and wellbeing; turning research, evidence and knowledge into action; acting as the authority on health, wellbeing and policy development; building an intelligent health system and a healthier population.
**Comments/notes**	

DOMAIN 12 - PLATFORMS FOR INTERACTION: There are coordination platforms and opportunities for synergies across government departments, levels of government, and other sectors (NGOs, private sector, and academia) such that policies and actions in food and nutrition are coherent, efficient and effective in improving food environments, population nutrition, diet-related NCDs and their related inequalities.

**PLAT1**<br>**There are robust coordination mechanisms across departments and levels of government (national, state and local) to ensure policy coherence, alignment, and integration of food, obesity and diet-related NCD prevention policies across governments.**	
**Definitions**<br><br>**and scope**	• Includes cross-government or cross-departmental governance structures, committees or working groups (at multiple levels of seniority), agreements, memoranda of understanding, etc<br>• Includes cross-government or cross-departmental shared priorities, targets or objectives<br>• Includes strategic plans or frameworks that map the integration and alignment of multiple policies or programs across governments and across departments<br>• Includes cross-government or cross-departmental collaborative planning, implementation or reporting processes, consultation processes for the development of new policy or review of existing policy
**International best practice examples (benchmarks)**	**Finland:** The Finnish National Nutrition Council is an inter-governmental expert body under the Ministry of Agriculture and Forestry with advisory, coordinating and monitoring functions. It is composed of representatives elected for three-year terms from government authorities dealing with nutrition, food safety, health promotion, catering, food industry, trade and agriculture (World Cancer Research Fund, 2016a).<br><br>**Malta:** Based on the Healthy Lifestyle Promotion and Care of NCDs Act (2016), Malta established an inter-ministerial Advisory Council on Healthy Lifestyles in August 2016 to advise the Minister of Health on any matter related to healthy lifestyles. In particular, the Advisory Council advises on a life course approach to physical activity and nutrition, and on policies, action plans and regulations intended to reduce the occurrence of NCDs. The prime minister appoints the chair and the secretary of the Advisory Council, while the ministers of education, health, finance, social policy, sports, local

	government, and home affairs appoint one member each (World Cancer Research Fund, 2016a).<br><br>**Australia:** There are several forums and committees for the purpose of strengthening food regulation with representation from New Zealand and Health Ministers from Australian States and Territories, the Australian Government, as well as other Ministers from related portfolios (e.g. Primary Industries). Where relevant, there is also representation from the Australian Local Government Association.<br><br>**Ireland:** The Department of Health, through 'A Healthy weight for Ireland', Obesity Policy and Action Plan 2016 - 2025, will provide leadership, engage and co-ordinate multi-sectorial action and implement best practice in the governance of the OPAP. The department of health and safefood are taking action to establish a multi stakeholder partnership to share knowledge and initiative on healthy weight initiatives (Department of Health, 2016b).
**Context** e.g. EU action/ regulation / food industry action etc.	**EU Action**<br>• There are mechanisms at EU level, which aim to ensure cooperation and coordination at the level of the European Commission, the European Parliament, the Council, between the EU and the Member States, between Member States, and with subnational governments and social partners.
**Evidence of implementation**	**Department of Health**<br><br>• The Obesity Policy and Action Plan is a cross-sectoral and whole-of-government approach towards tackling the causes of obesity in Ireland. It is overseen by the Obesity Policy Implementation Oversight Group, which is comprised of representatives from a range of Government Departments and agencies.<br><br>• The Obesity Policy and Action Plan was developed under the Healthy Ireland Framework. For information, the WHO selected the Healthy Ireland Framework as an example of good practice at the 69th session of the WHO Regional Committee for Europe, as it is one of the flagship whole-of-government approaches from across the European Region.

	● 'The Department of Health, through Healthy Ireland, will provide leadership, engage and co-ordinate multi-sectorial action and implement best practice in the governance of the 'Obesity Policy Action Plan 2016-2025'. Leadership and coordinated action will be provided by the department of health. The department of health and safefood are taking action to establish a multi stakeholder partnership to share knowledge and initiative on healthy weight initiatives (Department of Health, 2016b).<br><br>**FSAI and safefood**<br><br>● There is a memorandum of understanding between the FSAI and other agencies, for example, the Food Safety Authority of Ireland and Safefood. This looks at a framework for cooperation in activities to do with food safety. The FSAI and Safefood agree on certain actions such as:<br>- To provide each other with full and timely access to research, findings, and data as well as other relevant information.<br>- To regularly inform each other about work that interests both organisations.<br>- To take opportunities that involve joint planning and to fully cooperate when needed (Food Safety Authority of Ireland & safefood, 2016)
**Comments/notes**	

**PLAT2**<br>**There are formal platforms (with clearly defined mandates, roles and structures) for regular interactions between government and the commercial food sector on the implementation of healthy food policies and other related strategies**	
**Definitions and scope**	• The commercial food sector includes food production, food technology, manufacturing and processing, marketing, distribution, retail and food service, etc. For the purpose of this indicator, this extends to commercial non-food sectors (e.g. advertising and media, sports organisations, land/housing developers, private childcare, education and training institutes) that are indirectly related to food<br>• Includes established groups, forums or committees active within the last 12 months for the purpose of information sharing, collaboration, seeking advice on healthy food policies<br>• Includes platforms to support, manage or monitor private sector pledges, commitment or agreements<br>• Includes platforms for open consultation<br>• Includes platforms for the government to provide resources or expert support to the commercial food sector to implement policy<br>• Excludes joint partnerships on projects or co-funding schemes<br>• Excludes platforms to engage with industry in relation to development of policies.<br>• Excludes initiatives covered by 'RETAIL3' and 'RETAIL4'
**International best practice examples (benchmarks)**	**UK:** The UK 'Responsibility Deal' was a UK government initiative to bring together food companies and NGOs to take steps (through voluntary pledges) to address NCDs. It was chaired by the Secretary of State for Health and included senior representatives from the business community (as well as NGOs, public health organisations and local government). A number of other subgroups were responsible for driving specific programs relevant to the commercial food sector.<br><br>**Norway:** The letter of intent (Memorandum of Understanding, MoU) for facilitating a healthier diet in the population is a signed agreement between the Norwegian health authorities and food industry (food and trade organizations, food and beverage manufacturers, food retailers and food service industry) in a *Partnership for a healthier diet.* The MoU was signed in 2016 and lasts until 2021. The agreement contains specific quantitative goals related to reducing the intake of salt, added sugar and saturated fat, and increasing the intake of fruits and berries, vegetables, whole grain foods, fish and seafood in the population. The Partnership is organized in a Coordination group with representatives from the main partners including the health

	authorities. The Coordination group reports to the Minister's food industry group (lead by the Minister for the Elderly and Public Health) that ensures dialogue and political focus on the areas of action. The Coordination group is assisted by a Secretariat organized by the Directorate of Health. A Reference group of scientists within nutrition, food technology, consumer behaviour, psychology and marketing provide expert advice to the coordination group (Helsedirektoratet, 2016).
**Context** e.g. EU action/ regulation / food industry action etc.	**EU Action**<br><br>**The EU Platform for action on diet, physical activity and health**<br>• The EU Platform for action on diet, physical activity and health is a forum for European-level organisations which was founded in 2005 and includes food business operators, consumer organisations, public health NGO's and scientific and professional associations (European Comission). The Platform receives guidance from the High Level Group on Nutrition and Physical Activity and holds joint meetings with the high level group.<br><br>**The EU Pledge**<br>• The EU Pledge was launched in 2007 as part of a commitment to the Platform for action on diet, physical activity and health (Eu-Pledge, 2019)The EU Pledge is a voluntary initiative by food and beverage companies to change advertising to children under 12 years in the European Union.
**Evidence of implementation**	**The Obesity Policy and Action Plan 2016-2025**<br><br>• Two stakeholder meetings in relation to reformulation were held between the Reformulation Sub-Group of the Obesity Policy Implementation Oversight Group and food industry representatives, in September 2018 and February 2019.<br><br>• Step 3, priority action 2 in the 'Obesity Policy and Action Plan 2016-2025', the Department of Health developed, implemented a code of practice for food and beverages promotion, marketing and sponsorship. Engagement between partners involved (food industry, HSE, DCYA, safefood, FSAI, advertiser organisations) was successful and will be continued on a regular basis.

	• There are also commercial bodies that regularly communicate with different sections of the government. Food Drink Ireland (FDI) is the main trade association for the food and drink industry in Ireland. It represents the interests of over 150 foods, drink and non-food grocery manufacturers and suppliers.
**Comments/notes**	

PLAT3<br>**There are formal platforms (with clearly defined mandates, roles and structures) for regular interactions between government and civil society on the development, implementation and evaluation of healthy food policies and other related strategies.**	
**Definitions and scope**	• Civil society includes community groups and consumer representatives, NGOs, academia, professional associations, etc.<br>• Includes established groups, forums or committees active within the last 12 months for the purpose of information sharing, collaboration, seeking advice<br>• Includes platforms for consultation on proposed plans, policy or public inquiries<br>• Excludes policies or procedures that guide consultation in the development of food policy (see 'GOVER3')
**International best practice examples (benchmarks)**	**Brazil:** The National Council of Food and Nutrition Security (CONSEA) is a formal advisory platform made up of civil society (2/3) and government reps (1/3). It is a participatory instrument for designing, suggesting, implementing and evaluating food and nutritional security policy (CONSEA). Through CONSEA, civil society has been able to influence policy directions more directly. CONSEA supported Congress to pass a bill obliging local governments to buy at least 30% of the food destined for school meals from small-scale farmers.
**Context** e.g. EU action/ regulation / food industry action etc.	**EU Action**<br><br>**European Health Policy Platform**<br>• The European Health Policy Platform is a collaborative initiative under the 3rd Health Programme to ease communication among health stakeholders and with the European Commission (European Comission). DG SANTE act's as the secretary of the Platform.<br><br>**European Economic and Social Committee**<br>• The EESC enables civil society organisations (350 members) from the Member States to express their views at European level (European Economic and Social Committee). Its opinions are addressed to the European Commission, Parliament and Council. Consultation of the EESC is mandatory for public health policies (Article 168 of the TFEU) (Official Journal of the European Union, 2012). The EESC may also adopt opinions on its own initiative (European Sources Online, 2013).

	• There are a number of civil society groups that campaign to influence the government food and public health policies and there are formal platforms for interaction at ministerial level
**Evidence of implementation**	• Under the Healthy Ireland Framework and through the Healthy Ireland Fund, the Healthy Counties and Cities approach is supporting existing cross-partnership groups that have been set up in each local authority
**Comments/notes**	

**PLAT4**<br>**The governments work with a system-based approach with (local and national) organisations/partners/groups to improve the healthiness of food environments at a national level.**	
**Definitions and scope**	• Systems-based approaches may include policies within other domains of health<br>• May include a social-determinants of health approach<br>• May bring together multiple departments or ministries to approach health<br>• Includes multiple levels of government<br>• Aim of a systems-based approach is:<br>o resourcing and supporting a dedicated, reflective and skilled workforce at a state and/or local level to engage, activate and influence at multiple levels of the system to combat obesity and chronic disease<br>o building relationships with prevention partners across the system, and across sectors and industries, to strengthen positive health outcomes on multiple fronts<br>o capturing and feeding back knowledge and data on progress, impact and effectiveness and calling for new types of research, policy and practice collaborations<br>o allocating resources based on best possible investment to effect change and population need, seeding long term change by resourcing local governments to lead action towards public health<br>o building leadership for sustained prevention across the system to drive effective and long lasting change.
**International best practice examples (benchmarks)**	**New Zealand:** Healthy Families NZ is a large-scale initiative that brings community leadership together in a united effort for better health. It aims to improve people's health where they live, learn, work and play, in order to prevent chronic disease. Led by the Ministry of Health, the initiative will focus on ten locations in New Zealand in the first instance. It has the potential to impact the lives of over a million New Zealanders. The Government has allocated $40 million over four years to support Healthy Families NZ (Ministry of Health New Zealand, 2016).<br><br>**Australia:** Healthy together Victoria in Australia focuses on addressing the underlying causes of poor health in children's settings, workplaces and communities by encouraging healthy eating and physical activity and reducing

	smoking and harmful alcohol use. Healthy Together Victoria incorporates policies and strategies to support good health across Victoria, as well as locally-led Healthy Together Communities. The initiative was originally jointly funded by the State Government of Victoria and the Australian Government through the National Partnership Agreement on Preventive Health (Government of South Australia, 2016). It is unclear at this stage whether funding for Healthy Together Victoria will continue or not.
**Context** e.g. EU action/ regulation / food industry action etc.	**EU Action**<br><br>• We have no found any evidence that the EU is using a system-based approach to improve the healthiness of food environments in EU countries. However a lot of different organisations have recommended such an approach, like the EESC (opinion on civil society's contribution to the development of a comprehensive food policy in the EU) (European Economic and Social Committee, 2019), the WHO (Connecting Food Systems for co-benefits Policy Brief) (World Health Organisation, 2018) and IPES (Towards a Common Food Policy for the European Union report) (IPES - Food, 2019).<br><br>• The FAO published in the frame of the Strategic Partnership with the Directorate for International Cooperation and Development of the European Commission a guideline (Food Systems for healthy Diets) to support the use of a comprehensive food system approach for policy makers in countries (FAO, 2018)
**Evidence of implementation**	**Healthy Ireland framework 2013-2025**<br><br>• The Healthy Ireland Framework draws on existing policies but proposes new arrangements to ensure effective co-operation and collaboration and to implement evidence-based policies at government, sectoral, community and local levels. It proposes a necessary shift towards a broader, more inclusive approach to governance for health, moving beyond the health service, across national and local authorities, involving all sectors of society, and the people themselves. The framework aims to draw up specific proposals in relation to the potential role of local authorities in the area of health and wellbeing. Furthermore, Local health partners will engage with local authorities in their work to address local and

	community development, with the aim of coordinating actions and improving information-sharing for improved health and wellbeing (Healthy Ireland, 2013-2025).
**Comments/notes**	

DOMAIN 13 - HEALTH IN ALL POLICIES: Processes are in place to ensure policy coherence and alignment, and that population health impacts are explicitly considered in the development of government policies.

<table>
<tr><td colspan="2">HIAP1
There are processes in place to ensure that population nutrition, health outcomes and reducing health inequalities or health impacts in vulnerable populations are considered and prioritised in the development of all government policies relating to food</td></tr>
<tr><td>Definitions

and scope</td><td>• Includes policies, procedures, guidelines, tools and other resources that guide the consideration and assessment of nutrition, health outcomes and reducing health inequalities or health impacts in vulnerable populations prior to, during and following implementation of food-related policies.
• Includes the establishment of cross-department governance and coordination structures while developing food-related policies.</td></tr>
<tr><td>International best practice examples (benchmarks)</td><td>Slovenia: A Health Impact Assessment was undertaken in Slovenia to assess the health effects of agricultural policy at national level policy analysis, rapid appraisal workshops with stakeholders from a range of backgrounds, review of research evidence relevant to the agricultural policy, analysis of Slovenian data for key health-related indicators, a report on the findings to a key cross-government group and evaluation (Lock, 2003).

Ireland: Step 9 of the ‘ A Healthy Weight for Ireland’ Obesity Policy and Action Plan 2016-2025 aims to, allocate resources according to need, in particular to those population groups most in need of support in the prevention and management of obesity, with particular emphasis on families and children during the first 1,000 days of life. The priority actions to commence in first year were to, assess the needs of vulnerable groups as the basis of allocation of resources for preventative and treatment services for children and adults (Department of Health, 2016c). The Healthy Ireland fund was established in 2017 with an allocation of €5 million and with additional allocations of €5 million in 2018 and 2019. The first round of the Fund was distributed through Local Community Development Committees, Children and Young Person’s Services Committees and statutory organisations (Oireachtas, 2019).</td></tr>
</table>

**Context** e.g. EU action/ regulation / food industry action etc.	**Health Service Executive (HSE) Health Promotion Strategic Framework (2011)**<br><br>• The Health Service Executive (HSE) Health Promotion Strategic Framework places the emphasis for health promotion activity on addressing the determinants of health and health inequalities which will primarily be achieved through strong national leadership aimed at putting health on the agenda of all those involved in planning and decision making. This includes advocating for a Health in All Policies approach (HiAP) as well as building and strengthening cross-sectoral and inter-departmental Government partnerships (Health Service Executive, 2011)
**Evidence of implementation**	• No further evidence found from 2016 onwards
**Comments/notes**	

HIAP2<br>**There are processes e.g. Health Impact Assessment's (HIAs) to assess and consider health impacts during the development of other non-food policies.**	
**Definitions**<br><br>**and scope**	• Includes a government-wide HiAP strategy or plan with clear actions for non-health sectors.<br>• Includes policies, guidelines, tools and other resources that guide the consideration and assessment of health impacts prior to, during and following implementation of non-food related policies (e.g. HIAs or health lens analysis).<br>• Includes the establishment of cross-department or cross-sector governance and coordination structures to implement a HiAP approach.<br>• Includes workforce training and other capacity building activities in healthy public policy for non-health departments (e.g. agriculture, education, communications, trade).<br>• Includes monitoring or reporting requirements related to health impacts for non-health departments.
**International best practice examples (benchmarks)**	**South Australia:** Established in 2007, the implementation of Health in All Policies (HiAP) in South Australia has been supported by a high-level mandate from central government, an overarching framework which is supportive of a diverse program of work, a commitment to work collaboratively and in partnership across agencies, and a strong evaluation process. The government has established a dedicated HiAP team within South Australia Health to build workforce capacity and support Health Lens Analysis projects. Since 2007, the South Australian HiAP approach has evolved to remain relevant in a changing context. However, the purpose and core principles of the approach remain unchanged. There have been five phases to the work of HiAP in South Australia between 2007 and 2016: 1) Prove concept and practice emerges (2007-2008), 2) Establish and apply methodology (2008-2009), 3) Consolidate and grow (2009-2013), 4) Adapt and review (2014) and 5) Strengthen and systematize (2015-2016).
**Context** e.g. EU action/ regulation / food industry action etc.	
**Evidence of implementation**	**Healthy Ireland framework 2013-2025**<br><br>• The Republic of Ireland National Framework to improve health and wellbeing 'Healthy Ireland' acknowledges that intersectoral working

	or Health in All Policies is a politically challenging strategy that requires deliberate efforts to be promoted. The intersectoral approach requires the use of validated tools and support mechanisms to drive this agenda, including for example, Health Impact Assessment, inter-ministerial and inter-departmental committees, cross-sector action teams, joined-up workforce development, legislative frameworks.<br><br>• In Ireland, the Government has committed to incorporating poverty impact assessment as part of an integrated social impact assessment. Health impacts will be a core feature of this new tool. The Outcomes Framework aims to provide a structured approach to collect and report relevant and appropriate data which can be used to build awareness of these social determinants of health, to support assessment of the impact of policies on the agreed outcomes, and to monitor progress on the whole of government response needed to improve health and wellbeing (Healthy Ireland, 2013-2025).
**Comments/notes**	**The institute of Public Health in Ireland and the Department of Health and Children**<br><br>• The Institute of Public Health in Ireland developed the health impact assessment, a practical guidance manual in 2003, that was the first detailed methodology for HIA in Ireland. This document was updated in 2009 and describes Health Impact Assessment (HIA) and the steps involved in HIA. It gives advice based on the experience of HIA practitioners and provides tools to help carry out these steps and to adapt HIA to local circumstances. It aims to provide a user friendly and practical framework to guide policy makers through the HIA process and to enable them to undertake a HIA is responsible for providing up to date Health Information Assessment (HIA) guidance. The Department of Health and Children commissioned the Institute of Public Health in Ireland to assist in this task by advising on methodology, producing guidance and facilitating training. The purpose of HIA is to influence decision making in favour of health by providing decision-makers with evidence- based recommendations to maximise the positive and minimise the negative health impacts of proposals (Doyle. C and Metcalve. O, 2003)

References

A. FARRELL. 2019. *Report of Joint Committee on Children and Youth Affairs: Motion* [Online]. Available: https://www.oireachtas.ie/en/debates/debate/dail/2019-02-07/56/ [Accessed].

ADMINISTRATION, F. A. D. 2019. FDA Nutrition Innovation Strategy. .

ADVERTISING STANDARDS AUTHORITY. 2018. *Food: HFSS Media Placement* [Online]. Available: https://www.asa.org.uk/advice-online/food-hfss-media-placement.html [Accessed].

ADVERTISING STANDARDS AUTHORITY FOR IRELAND 2015. Section 7: Children. [online] available: https://www.asai.ie/asaicode/section-7-children/.

AGRI AWARE'S INCREDIBLE EDIBLES. *https://www.incredibleedibles.ie/the-incredible-edibles-project.html* [Online]. [Accessed].

AUDITORS, E. C. O. 2019. Report FEAD-Fund for European Aid to the Most Deprived: Valuable support but its contribution to reducing poverty is not yet established. [online] available https://www.eca.europa.eu/lists/ecadocuments/sr19_05/sr_fead_en.pdfSpecial

BAE, S. G., J.Y. KIM, K.Y. KIM, S.W. PARK, J. BAE, AND W.K. LEE, 2012. Changes in dietary behavior among adolescents and their association with government nutrition policies in Korea, 2005-2009. J Prev Med Public Health, 45(1): p. 47-59.

BIRO, A. 2015. Did the junk food tax make the Hungarians eat healthier? Food Policy. 54: p. 107-115.

BREDA, J., JEWELL, J., WEBBER, L. & GALEA, G. 2015. WHO projections in adults to 2030. *Obesity Facts. The European Journal of Obesity,* 8**,** 18.

BROADCASTING AUTHORITY OF IRELAND 2013. BAI Children's Commercial Communications Code.

BROADCASTING AUTHORITY OF IRELAND 2017. General commercial Communications Code

BROADCASTING AUTHORITY OF IRELAND. 2019. *Codes and Standards* [Online]. Available: https://www.bai.ie/en/codes-standards/#al-block-5 [Accessed].

BROADCASTING AUTHORITY OF IRELAND. 2013. BAI Children's Commercial Communications Code.

BYRNE. C. 2012. How St Vincent de Paul €10m voucher system gives people back their dignity. *The Irish Independent*

CENTRES FOR DISEASE CONTROL 2016. National Health and Nutrition Examination Survey. Available from: http://www.cdc.gov/nchs/nhanes.htm.

CHANTAL, J. A. H., S., 2017. Development of a new front-of-pack nutrition label in France: the five colour Nutri-Score.

COLCHERO, M. A., B.M. POPKIN, J.A. RIVERA, AND S.W. NG, 2016. Beverage purchases from stores in Mexico under the excise tax on sugar sweetened beverages: observational study. BMJ 352: p. h6704.

COLRUYT GROUP. 2018. *What is the Nutri-Score? [online] available: https://nutriscore.colruytgroup.com/colruytgroup/en/about-nutri-score/* [Online]. [Accessed].

COMMONWEALTH OF AUSTRALIA 2016. Health Star Rating System. Available from: http://healthstarrating.gov.au/internet/healthstarrating/publishing.nsf/content/home.

CORPORATE EUROPE OBSERVATORY 2010. A red light for consumer information. The food industry's €1-billion campaign to block health

warnings on food. [online] available: http://images.smh.com.au/file/2011/09/06/2606842/CEO-Food-Labelling.pdf.

CORREA, T., FIERRO, C., REYES, M., DILLMAN CARPENTIER, F. R., TAILLIE, L. S. & CORVALAN, C. 2019. "Responses to the Chilean law of food labeling and advertising: exploring knowledge, perceptions

and behaviors of mothers of young children". *International Journal of Behavioral Nutrition and Physical Activity,* 16**,** 21.

CORVALÁN C, R. M., GARMENDIA ML, UAUY R, 2018. Structural responses to the obesity and non-communicable diseases epidemic: Update on the Chilean law of food labelling and advertising. Obesity Reviews. 2018;1–8. https://

doi.org/10.1111/obr.12802.

DEE, A., CALLINAN, A., DOHERTY, E., O'NEILL, C., MCVEIGH, T., SWEENEY, M., STAINES, A., KEARNS, K., FITZGERALD, S., SHARP, L., KEE, F., HUGHES, J., BALANDA, K. & PERRY, I. 2015. Overweight and obesity on the island of Ireland: an estimation of costs. *BMJ Open,* 5**,** e006189.

DEPARMENT OF HEALTH 2013. Guide to creating a front of pack (FoP) nutrition label for pre-packed products sold through retail outlets. Available from: https://www.gov.uk/government/uploads/system/uploads/attachment_data/file/300886/2902158_FoP_Nutrition_2014.pdf.

DEPARTMENT FOR CHILDREN AND YOUTH AFFAIRS 2014-2020. Better Outcomes Brighter Futures. The national policy framework for children and yound people, 2014-2020.

DEPARTMENT OF AGRICULTURE FOOD AND MARINE 2017a. Creed Announces Re-Opening of Beef Data and Genomics Programme.

DEPARTMENT OF AGRICULTURE FOOD AND MARINE. 2017b. *EU School Scheme. [online] https://www.agriculture.gov.ie/customerservice/euschoolscheme/* [Online]. [Accessed].

DEPARTMENT OF AGRICULTURE FOOD AND MARINE 2018. Terms and conditons of the scheme of EU AID for producer organisations in the fruit and vegetable sector

DEPARTMENT OF AGRICULTURE FOOD AND MARINE 2019. 2019 scheme of investment aid for the development of the commercial horticulture sector.

DEPARTMENT OF AGRICULTURE FOOD AND MARINE AND AN BORD BIA 2009. Voluntary Good Practice Standard for Farmers' Markets 2012-2013.

DEPARTMENT OF CHILDREN AND YOUTH AFFAIRS 2018a. First 5: A Whole-of-Government Strategy for Babies, Young Children and their Families.

DEPARTMENT OF CHILDREN AND YOUTH AFFAIRS 2018b. Growing Up in Ireland National Longitudinal Study of Children. The Lives of 13-Year-Olds.

DEPARTMENT OF EMPLOYMENT AFFAIRS AND SOCIAL PROTECTION 2018. Written submission by the Department of Employment Affairs and Social Protection regarding the School Meals Programme to the Joint Committee on Children and Youth Affairs on the topic 'Tackling Childhood Obesity'. [online] Accessed on https://data.oireachtas.ie/ie/oireachtas/committee/dail/32/joint_committee_on_children_and_youth_affairs/submissions/2018/2018-08-22_submission-department-of-employment-affairs-and-social-protection_en.pdf.

DEPARTMENT OF EMPLOYMENT AFFAIRS AND SOCIAL PROTECTION 2019. First Serving of Hot School Meals Project Commences in Inchicore. https://www.welfare.ie/en/pressoffice/Pages/prb280119.aspx.

DEPARTMENT OF HEALTH 2013. Healthy Ireland - a framework for improved health and wellbeing 2013 - 2025. Dublin: Department of Health.

DEPARTMENT OF HEALTH. 2014. *National salth reduction initiative corporate achievements 2009-2014.* [Online]. Available: https://www1.nyc.gov/assets/doh/downloads/pdf/cardio/nsri-corporate-commitments.pdf [Accessed].

DEPARTMENT OF HEALTH 2016a. Get Ireland Active! The National Physical Activity Plan for Ireland.

DEPARTMENT OF HEALTH 2016b. A Healthy Weight for Ireland. Obesity Policy and Action Plan: 2016-2025.

DEPARTMENT OF HEALTH 2016c. A Healthy Weight for Ireland: Obesity Policy and Action Plan 2016 – 2025.

DEPARTMENT OF HEALTH 2017. Non-Broadcast Media Advertising and Marketing of Food and Non-Alcoholic Beverages, including Sponsorship and Retail Product Placement:Voluntary Codes of Practice.

DEPARTMENT OF HEALTH. 2019a. *Healthy Ireland Food Plans and Guides* [Online]. Available: https://www.gov.ie/en/publication/b01555-healthy-ireland-food-plans-and-guides/ [Accessed].

DEPARTMENT OF HEALTH 2019b. Healthy Ireland Fund 2019- 2021. Expression of Interest Guidelines. [online] Accessed on https://drive.google.com/file/d/1rqVbr_YILdi12GqeGNgPnILPaEbrCBKB/view.

DEPARTMENT OF HEALTH. 2019c. *National Healthy Cities and Counties of Ireland Network. [online] Accessed on https://www.gov.ie/en/publication/d4fa22-healthy-counties-and-cities/#national-healthy-cities-and-counties-of-ireland-network* [Online]. [Accessed].

DEPARTMENT OF HEALTH 2019d. Taoiseach and Ministers launch Healthy Ireland campaign. [online] Accessed on https://www.gov.ie/en/news/20cff4-taoiseach-and-health-ministers-to-launch-2019-healthy-ireland-campai/.

DEPARTMENT OF HEALTH AND MENTAL HYGIENE Food Preparation and Food Establishments (Article 81, Section 49) - Sodium Warning. Available from: http://rules.cityofnewyork.us/content/food-preparation-and-food-establishments-article-81-section-49-sodium-warning-0.

DEPARTMENT OF HEALTH. 2016. A Healthy Weight for Ireland. Obesity Policy and Action Plan: 2016–2025. Dublin.

DEPARTMENT OF PUBLIC EXPENDITURE AND REFORM. 2015. *Transparency Code Prepared in Accordance with Section 5(7) of the Regulation of Lobbying Act 2015. [online] Accessed on https://www.lobbying.ie/media/5986/2015-08-06-transparency-code-eng.pdf* [Online]. [Accessed].

DEPARTMENT OF PUBLIC EXPENDITURE AND REFORM 2019. FOI Publication Scheme. [online] Accessed on https://www.gov.ie/en/organisation-information/60131b-foi-publication-scheme/.

DEPARTMENT OF THE ENVIRONMENT, C. A. L. G. 2013. Local Area Plans Guidelines for Planning Authorities.

DOWNS, S., BLOEM, M AND GRAZIOSE, MM., 2017. Salt and the city: a preliminary examination of New York City's sodium warning labels. .

DOYLE. C AND METCALVE. O 2003. Health Impact Assessment. A practical guidance manual. .

ENVIRONMENTAL PROTECTION AGENCY 2014. Green procurement. Guidance for the Public Sector

EU-PLEDGE 2019. About the EU pledge. [online] available: https://eu-pledge.eu/about-the-eu-pledge/.

EURACTIVE. 2013. *EU lawmakers reject colour-coded system for food labels. [online] available: https://www.euractiv.com/section/health-consumers/news/eu-lawmakers-reject-colour-coded-system-for-food-labels/* [Online]. [Accessed].

EURACTIVE 2014. Food industry wins battle on 'traffic light' labels [online] available: https://www.euractiv.com/section/health-consumers/news/food-industry-wins-battle-on-traffic-light-labels/.

EUROPA. *Chapter VI Guidelines on evaluation (including fitness checks). [online]* *https://ec.europa.eu/info/sites/info/files/better-regulation-guidelines-evaluation-fitness-checks.pdf* [Online]. [Accessed].

EUROPA. 2012. *Consolidated ersion of the treaty on the functioning of the European Union. [online] available: https://eur-lex.europa.eu/legal-content/EN/TXT/PDF/?uri=CELEX:12012E/TXT&from=EN* [Online]. [Accessed].

EUROPA 2014a. Code of Conduct. [online] available: http://ec.europa.eu/transparencyregister/public/staticPage/displayStaticPage.do?locale=en&reference=CODE_OF_CONDUCT.

EUROPA 2014b. EU Action Plan on Childhood Obesity 2014-2020. [online]

EUROPA 2014c. EU Action Plan on Childhood Obesity 2014-2020. [online] available https://ec.europa.eu/health/sites/health/files/nutrition_physical_activity/docs/childhoodobesity_actionplan_2014_2020_en.pdf.

EUROPA 2017. Call for tenders' details. [online] available: https://etendering.ted.europa.eu/cft/cft-display.html?cftId=3068.

EUROPEAN COMISSION EU Health Policy Platform. [online] available: https://webgate.ec.europa.eu/hpf/.

EUROPEAN COMISSION European Research Area (ERA). Policy initiatives and practices of a unified common European research area, partnership details and progress reports. [online] available: https://ec.europa.eu/info/research-and-innovation/strategy/era_en.

EUROPEAN COMISSION. *Food information to consumers - legislation. [online] available: https://ec.europa.eu/food/safety/labelling_nutrition/labelling_legislation_en* [Online]. [Accessed].

EUROPEAN COMISSION Funding: Health policies in the future EU budget (2021-2027). [online] available: https://ec.europa.eu/health/funding/future_health_budget_en.

EUROPEAN COMISSION Funding: Investment plan. [online] available: https://ec.europa.eu/health/funding/investment_plan_en.

EUROPEAN COMISSION Funding: Structural funds. [online] available: https://ec.europa.eu/health/funding/structural_funds_en.

EUROPEAN COMISSION Heading 2: Sustainable growth: natural resources. A list of the programmes funded under heading 2 of the current long-term budget, with reference amounts and legal bases. [online] available: https://ec.europa.eu/info/strategy/eu-budget/spending/topic/eu-funding-programmes-2014-2020/heading-2-sustainable-growth-natural-resources_en#common-agricultural-policy-pillar-i.

EUROPEAN COMISSION Health Investments by European Structural and Investment Funds (ESIF). Fact Sheet. 2014-2020. [online] available: https://ec.europa.eu/health/sites/health/files/health_structural_funds/docs/esif_factsheet_en.pdf.

EUROPEAN COMISSION Horizon 2020. [online] available: https://ec.europa.eu/programmes/horizon2020/en/what-horizon-2020.

EUROPEAN COMISSION Horizon 2020. Food & Healthy Diet. [online] available: https://ec.europa.eu/programmes/horizon2020/en/area/food-healthy-diet.

EUROPEAN COMISSION Import Conditions. [online] available: https://ec.europa.eu/food/safety/international_affairs/trade_en.

EUROPEAN COMISSION Nutrition and physical activity. EU platform for action on diet, physical activity and health. [online] available: https://ec.europa.eu/health/nutrition_physical_activity/platform_en.

EUROPEAN COMISSION 2006. REGULATION (EC) No 1924/2006 OF THE EUROPEAN PARLIAMENT AND OF THE COUNCIL of 20 December 2006 on nutrition and health claims made on foods. [online] available on https://eur-lex.europa.eu/legal-content/EN/TXT/PDF/?uri=CELEX:32006R1924&from=en.

EUROPEAN COMISSION 2008. REGULATION (EC) No 1338/2008 OF THE EUROPEAN PARLIAMENT AND OF THE COUNCIL of 16 December 2008 on Community statistics on public health and health and safety at work. [online] available: https://eur-lex.europa.eu/legal-content/EN/TXT/PDF/?uri=CELEX:32008R1338&from=EN.

EUROPEAN COMISSION 2016a. Strategic plan 2016-2020 – Health and Food Safety. .

EUROPEAN COMISSION 2016b. Strategic plan 2016-2020 – Health and Food Safety. [online] https://ec.europa.eu/info/publications/strategic-plan-2016-2020-health-and-food-safety_en.

EUROPEAN COMISSION 2018a. Revision of the Audiovisual Media Services Directive (AVMSD). [online] available https://ec.europa.eu/digital-single-market/en/revision-audiovisual-media-services-directive-avmsd.

EUROPEAN COMISSION 2018b. Steering Group on Health Promotion, Disease Prevention and Management of Non Communicable Diseases (E03622). [online] available: https://ec.europa.eu/transparency/regexpert/index.cfm?do=groupDetail.groupDetail&groupID=3622.

EUROPEAN COMISSION 2019a. Fund for European Aid to the Most Deprived (FEAD). [online] available: https://ec.europa.eu/social/main.jsp?catId=1089.

EUROPEAN COMISSION 2019b. Initiatives on Nutrition and Physical Activity.

EUROPEAN COMISSION 2019c. Initiatives on Nutrition and Physical Activity. [online] available https://ec.europa.eu/health/sites/health/files/nutrition_physical_activity/docs/2019_initiatives_npa_en.pdf.

EUROPEAN COMISSION 2019d. Reflection paper towards a sustainable Europe by 2030.

EUROPEAN COMMISSION. *Food-Based Dietary Guidelines in Europe. [online] available: https://ec.europa.eu/jrc/en/health-knowledge-gateway/promotion-prevention/nutrition/food-based-dietary-guidelines* [Online]. [Accessed].

EUROPEAN COMMISSION. *Transparency register. [online] available: https://ec.europa.eu/info/about-european-commission/service-standards-and-principles/transparency/transparency-register_en* [Online]. [Accessed].

EUROPEAN COMMISSION 2002. REGULATION (EC) No 178/2002 OF THE EUROPEAN PARLIAMENT AND OF THE COUNCIL

of 28 January 2002.

EUROPEAN COMMISSION 2015. Communication from the commission to the European Parliment, the Council, the European Economic and Social Committee and the Committee of the Regions. Better regulation for better results - An EU agenda. [online] available: https://eur-lex.europa.eu/legal-content/EN/TXT/?qid=1451989021436&uri=CELEX:52015DC0215.

EUROPEAN COMMISSION. 2019,. *Health promotion and disease prevention knowledge gateway. [online] Accessed on https://ec.europa.eu/jrc/en/health-knowledge-gateway/promotion-prevention/nutrition* [Online]. [Accessed].

EUROPEAN ECONOMIC AND SOCIAL COMMITTEE. *About. [online] available https://www.eesc.europa.eu/en/about* [Online]. [Accessed].

EUROPEAN ECONOMIC AND SOCIAL COMMITTEE 2019. Promoting healthy and sustainable diets in the EU (own-initiative opinion). [online] availablë: https://www.eesc.europa.eu/en/our-work/opinions-information-reports/opinions/promoting-healthy-and-sustainable-diets-eu-own-initiative-opinion.

EUROPEAN FOOD SAFETY AUTHORITY. *Food composition* [Online]. [Accessed].

EUROPEAN FOOD SAFETY AUTHORITY 2010a. Scientific Opinion on establishing Food-Based Dietary Guidelines. [online] available: https://www.efsa.europa.eu/en/efsajournal/pub/1460.

EUROPEAN FOOD SAFETY AUTHORITY 2010b. Scientific Opinion on establishing Food-Based Dietary Guidelines. EFSA Panel on Dietetic Products, Nutrition, and Allergies (NDA). European Food Safety Authority (EFSA), Parma, Italy. [online] available: https://efsa.onlinelibrary.wiley.com/doi/epdf/10.2903/j.efsa.2010.1460.

EUROPEAN PARLIMENT 2019. The EU fruit and vegetable sector Main features, challenges and prospects, [online] available: http://www.europarl.europa.eu/RegData/etudes/BRIE/2019/635563/EPRS_BRI(2019)635563_EN.pdf.

EUROPEAN SOURCES ONLINE 2013. Information Guide. European Economic and Social Committee. [online] available: http://aei.pitt.edu/75338/1/European_Economic_Social_Committee.pdf.

EUROPEAN UNION 2011. Regulation (EU) No 1169/2011 of the European Parliment and of the Council of 25 October 2011. [online] available: https://eur-lex.europa.eu/LexUriServ/LexUriServ.do?uri=OJ:L:2011:304:0018:0063:EN:PDF.

FAO 2018. Strengthening sector policies for better food security and nutrition results. [online] available: http://www.fao.org/3/CA2797EN/ca2797en.pdf.

FITZGERALD, S., GILGAN, L., MCCARTHY, M., PERRY, I. J. & GEANEY, F. J. P. H. N. 2018. An evaluation and exploration of Irish food-service businesses' uptake of and attitudes towards a voluntary government-led menu energy (calorie) labelling initiative. 21**,** 3178-3191.

FOOD AND DRINK IRELAND 2019. The evolution of food and drink in Ireland 2005-2017. Reformulation and innovation: supporting Irish diets. .

FOOD CHOICE AT WORK 2019. Our Approach. [online] available: https://www.foodchoiceatwork.com/.

FOOD DRINK IRELAND 2016 The FDII/ Creme Global Reformulation Project. Estimating the impact of reformulation by 14 FDII members on the Irish population

FOOD SAFETY AUTHORITY OF IRELAND 2008. Trans Fatty Acid Survey – Retail Products (2007). Dublin: Food Safety Authority of Ireland, 2008.

FOOD SAFETY AUTHORITY OF IRELAND 2009. Trans Fatty Acid Survey of Fast Foods in Ireland (2008). Dublin: Food Safety Authority of Ireland, 2009.

FOOD SAFETY AUTHORITY OF IRELAND 2010a. Accuracy of Nutrition Labelling of Pre-Packaged Food in Ireland. Monitoring & Surveillance Series.

FOOD SAFETY AUTHORITY OF IRELAND 2010b. Monitoring and Surveillance Series. Accuracy of Nutrition Labelling of Pre-Packaged Food in Ireland. July 2010.

FOOD SAFETY AUTHORITY OF IRELAND 2011. Scientific Recommendations for Healthy Eating Guidelines in Ireland [Online]. Dublin: Food Safety Authority of Ireland.

FOOD SAFETY AUTHORITY OF IRELAND 2012. Putting calories on menus in Ireland. Technical Guidance for Food Businesses. [online]

FOOD SAFETY AUTHORITY OF IRELAND. 2014a. *Labelling Monitoring and Surveillance. [online] Accessed on https://www.fsai.ie/enforcement_audit/monitoring/food_monitoring_surveillance/labelling.html* [Online]. [Accessed].

FOOD SAFETY AUTHORITY OF IRELAND. 2014b. *Objectives of Salt Reduction Programme. https://www.fsai.ie/science_and_health/salt_and_health/objectives_of_salt_programme.html* [Online]. [Accessed].

FOOD SAFETY AUTHORITY OF IRELAND 2016a. Information on Nutrition and Health Claims.

FOOD SAFETY AUTHORITY OF IRELAND 2016b. Monitoring and Surveillance Series. Survey of the trans fatty acid content in processed food products in Ireland. .

FOOD SAFETY AUTHORITY OF IRELAND 2016c. Salt and Health: Review of the Scientific Evidence and Recommendations for Public Policy in Ireland (Revision 1). .

FOOD SAFETY AUTHORITY OF IRELAND 2016d. Salt reducing in processed foods - Salt in diet still high. .

FOOD SAFETY AUTHORITY OF IRELAND 2018a. Monitoring of Sodium and Potassium in Processed Foods. Period: September 2003 to December 2018.

FOOD SAFETY AUTHORITY OF IRELAND 2018b. Scientific Committee. [online] Accessed on https://www.fsai.ie/about_us/scientific_committee.html.

FOOD SAFETY AUTHORITY OF IRELAND 2019. About MenuCal. https://www.fsai.ie/food_businesses/menucal.html [online]

FOOD SAFETY AUTHORITY OF IRELAND & SAFEFOOD 2016. Memorandum of understanding between the Food Safety Authority of Ireland and safefood - the Food Safety Promotion Board

FOOD SAFETY AUTHORITY OF IRELAND. 2012. Calories on menus in Ireland: REPORT ON A NATIONAL CONSULTATION.

FUNDO NACIONAL DE DESINVOLVIMENTO DA EDUCACAO 2016. Sobre o PNAE. Available from: http://www.fnde.gov.br/programas/alimentacao-escolar.

GERMAN NUTRITION SOCIETY. *Nutrition Reports. [online] available: https://www.dge.de/en/nutrition-reports/* [Online]. [Accessed].

GLOBAL AGRICULTURAL INFORMATION NETWORK 2018. Update on Chilean Advertising of Packaged Foods and Beverages. [online] available: https://apps.fas.usda.gov/newgainapi/api/report/downloadreportbyfilename?filename=Update%20on%20Chilean%20Advertising%20of%20Packaged%20Foods%20and%20Beverages%20_Santiago_Chile_2-14-2018.pdf.

GOVERNMENT OF IRELAND 2016. Healthy Ireland Fund

GOVERNMENT OF IRELAND 2018. Health Budget Oversight & Management: Alignment of Health Budget and National Service Plan. .

GOVERNMENT OF SOUTH AUSTRALIA 2016. Health in All Policies: The South Australian Approach. Available from: http://www.sahealth.sa.gov.au/wps/wcm/connect/public+content/sa+health+internet/health+reform/health+in+all+policies.

GREATER LONDON AUTHORITY 2019. TfL junk food ads ban will tackle child obesity. [online] available: https://www.london.gov.uk/what-we-do/business-and-economy/food/tfl-junk-food-ads-ban-will-tackle-child-obesity.

HEALTH BEHAVIOUR IN SCHOOL-AGED CHILDREN About. [online] available http://www.hbsc.org/about/index.html.

HEALTH COUNCIL OF THE NETHERLANDS 2015. Dutch dietary guidelines 2015. [online] available: file:///C:/Users/charlottegriffin/Downloads/advisory-report-dutch-dietary-guidelines-2015.pdf.

HEALTH INFORMATION QUALITY AUTHORITY 2016. Report of the review of nutrition and hydration care in public acute hospitals.

HEALTH SERVICE EXECUTIVE 2011. The Health Promotion Strategic Framwork

HEALTH SERVICE EXECUTIVE 2015. Healthy Ireland in the Health Service Implementation Plan.

HEALTH SERVICE EXECUTIVE 2016a. Health Service Executive National Service Plan. .

HEALTH SERVICE EXECUTIVE. 2016b. *The Nurture Programme - Infant Health & Wellbeing* [Online]. [Accessed].

HEALTH SERVICE EXECUTIVE 2017a. Eat Smart A Guide for Healthy Eating and Getting Active for Children. .

HEALTH SERVICE EXECUTIVE 2017b. Healthy Eating and Active Living Programme | National Implementation Plan 2017-2020.

HEALTH SERVICE EXECUTIVE 2018. Food, Nutrition and Hydration Policy for adult patietns in acute hospitals

HEALTH SERVICE EXECUTIVE 2019. HSE Vending Policy

HEALTH SERVICES EXECUTIVE. 2018. *Brief updates from The Nurture Programme. https://www.hse.ie/eng/health/child/nurture/updates.html* [Online]. [Accessed].

HEALTHY IRELAND 2013-2025. A framework for improved health and wellbeing 2013-2025.

HEALTHY IRELAND 2016a. Healthy Food for Life. Revised healthy eating guidelines and Food Pyramid rationale. .

HEALTHY IRELAND 2016b. Revised healthy eating guidelines and Food Pyramid rationale.

HEALTHY IRELAND 2017a. Nutrition Standards for School Meals

HEALTHY IRELAND 2018. Healthy Ireland Survey 2018. [online] available: https://www.hse.ie/eng/about/who/healthwellbeing/healthy-ireland/publications/healthy-ireland-survey-2018.pdf.

HEALTHY IRELAND 2019. Healthy Ireland Fund. [online] available: https://www.pobal.ie/programmes/healthy-ireland-fund/.

HEALTHY IRELAND 2017b. Non- Broadcast Media Advertising and Marketing of food and Non-Alcoholic Beverages, including Sponsorship and Retail Product Placement: Voluntary Codes of Practice. .

HEALTHY IRELAND & DEPARTMENT OF HEALTH 2019. Healthy Ireland Survey documents. [online] available: https://www.gov.ie/en/collection/231c02-healthy-ireland-survey-wave/.

HOFMAN, K. J. & TOLLMAN, S. M. 2013. Population health in South Africa: a view from the salt mines. *The Lancet Global Health,* 1**,** e66-e67.

INFORMAS. 2019. *INFORMAS : FOOD-EPI. [online] https://www.informas.org/food-epi/* [Online]. [Accessed].

INSTITUTE OF PUBLIC HEALTH. 2019. *Policy making and public health. [online] Accessed on https://www.publichealth.ie/policy* [Online]. [Accessed].

IPES - FOOD 2019. Towards a common food policy for the European Union. The policy reform and realignment that is required to build sustainable food systems in Europe. [online] available: http://www.ipes-food.org/_img/upload/files/CFP_FullReport.pdf.

IRISH BUSINESS AND EMPLOYERS CONFEDERATION 2017. The KeepWell Mark. An Ibec Initiative. .

IRISH HEART FOUNDATION. *https://irishheart.ie/your-health/our-health-programmes/healthy-workplaces/healthy-eating-award/* [Online]. [Accessed].

IRISH HEART FOUNDATION Schools Healthy Eating Award. [online] available https://irishheart.ie/your-health/our-health-programmes/healthy-schools/schools-eating-award/.

IRISH HEART FOUNDATION 2016 Who's feeding the kids online? Digital Food Marketing and children in Ireland

IRISH HEART FOUNDATION. 2019. *Healthy Workplaces. Accessed on https://irishheart.ie/your-health/our-health-programmes/healthy-workplaces/* [Online]. [Accessed].

IRISH MEDICAL ORGANISATION 2012. IMO Position Paper on Health Inequalities

JPI A HEALTHY DIET FOR A HEALTHY LIFE Our Vision. [online] available: https://www.healthydietforhealthylife.eu/index.php/about/vision.

KEANE, E., KEARNEY, P., PERRY, I., KELLEHER, C. & HARRINGTON, J. 2014. Trends and prevalence of overweight and obesity in primary school aged children in the Republic of Ireland from 2002-2012: a systematic review. *BMC Public Health,* 14**,** 974.

KELLY, P. M., A. DAVIES, A.J. GREIG, AND K.K. LEE, 2016. Obesity Prevention in a City State: Lessons from New York City during the Bloomberg Administration. Front Public Health, 4: p. 60.

KENT, M. P., DUBOIS, L. & WANLESS, A. J. I. J. O. P. O. 2011. Food marketing on children's television in two different policy environments. 6**,** e433-441.

LAYTE, R. & MCCRORY, C. 2011. Growing Up in Ireland. Overweight and Obesity among 9-year olds. Dublin: Department of Children and Youth Affairs.

LEDERER, A., C.J. CURTIS, L.D. SILVER, AND S.Y. ANGELL, 2014. Toward a healthier city: nutrition standards for New York City government. Am J Prev Med 46(4): p. 423-8.

LIDL. 2019. *Lidl Ireland commits to significant salt and sugar reduction by 2020* [Online]. Available: https://www.abettertomorrow-lidl.ie/news/lidl-ireland-commits-to-significant-salt-sugar-reductions-by-2020/ [Accessed].

MARIE CURIE ALUMNI ASSOCIATION All You Need Know About Innovation Union Iu. [online] available https://www.mariecuriealumni.eu/newsletter/all-you-need-know-about-innovation-union-iu.

MARINE, D. O. A. F. A. 2017. *Food Institutional Research Measure* [Online]. Available: https://www.agriculture.gov.ie/research/competitivenationalprogrammes/foodinstitutionalresearchmeasurefirm/ [Accessed].

MASSRI, C., SUTHERLAND, S., KÄLLESTÅL, C. & PEÑA, S. J. A. J. O. P. H. 2019. Impact of the Food-Labeling and Advertising Law Banning Competitive Food and Beverages in Chilean Public Schools, 2014–2016. 109**,** 1249-1254.

MATBRANSJENS FAGLIGE UTVALG 2019. Guidelines for marketing food and drink aimed at children. [online] available: http://www.mfu.as/85197-Retningslinjer-for-markedsforing-oppdatert-1-10-2019.

MCAVOY, H., BERGIN, D., KIERNAN, R., KEATING, T, 2018a. Consultation on the development of a Healthy Workplaces Framework for Ireland: A report prepared for the Department of Health. Dublin: Institute of Public Health in Ireland. .

MCAVOY, H., BERGIN, D., KIERNAN, R., KEATING, T., 2018b. Consultation on the development of a Healthy Workplaces Framework for Ireland: A report prepared for the Department of Health. Dublin: Institute of Public Health in Ireland.

MFU. 2016. *Code for marketing of food and drink aimed at children updated 1/9-2016* [Online]. Available: http://www.mfu.as/44552-Code-for-marketing-of-food-and-drink-aimed-at-children-updated-1-9-2016 [Accessed].

MICHELLE RUSSELL. 2012. Ireland: Tesco, Lidl to roll out hybrid nutrition labels/ [online] available: https://www.just-food.com/news/tesco-lidl-to-roll-out-hybrid-nutrition-labels_id120983.aspx.

MINISTRY OF FOOD AND DRUG SAFETY 2017. The Special Act on the Safety Management of Children's Dietary Life. Available from: https://www.mfds.go.kr/eng/index.do?nMenuCode=66.

MINISTRY OF HEALTH BRAZIL 2011. Health Surveillance Secretariat: Health situation analysis department. Strategic action plan to tackle noncommunicable diseases in Brazil 2011-2022. Ministry of Health: Brazil.

MINISTRY OF HEALTH SOUTH AFRICA 2013. Strategic Plan for the Prevention and Control of Non-Communicable Diseases 2013-17. Available from: http://www.hsrc.ac.za/uploads/pageContent/3893/NCDs%20STRAT%20PLAN%20%20CONTENT%208%20april%20proof.pdf.

MINISTRY OF PUBLIC HEALTH, W. A. S. Food Database. [online] available: https://www.levensmiddelendatabank.nl/Security/Login.aspx?ReturnUrl=%2f.

MORLEY, B., SCULLY, M., MARTIN, J., NIVEN, P., DIXON, H. & WAKEFIELD, M. 2013. What types of nutrition menu labelling lead consumers to select less energy-dense fast food? An experimental study. *Appetite,* 67**,** 8-15.

MURPHY, R., & O'DONOGHUE, E., 2018a. A description of public policy mechanisms to support healthy workplaces and workplace health programmes, Research Services Unit, Department of Health. .

MURPHY, R., O'DONOGHUE, E., & DOYLE, C., 2018b. Factors organisations should consider when developing healthy workplaces and workplace wellbeing programmes, Research Services Unit, Department of Health.

MURPHY, R., O'DONOGHUE, E., DOYLE, C. & TAAFFE, C., 2018c. An umbrella review of the effectiveness and cost-effectiveness of workplace wellbeing programmes, Research Services Unit, Department of Health. .

NATIONAL AGREEMENT TO IMPROVE PRODUCT COMPOSITION 2017. Available from: http://www.akkoordverbeteringproductsamenstelling.nl/dsresource?type=pdf&disposition=inline&objectid=rivmp:303682&versionid=&subobjectname=.

NATIONAL INSTITUTE FOR HEALTH AND WELFARE 2019. EATING TOGETHER -food recommendations for families with children.

NATIONAL NUTRITION COUNCIL 2017. Eating and learning together – recommendations for school meals.

NATIONAL NUTRITION COUNCIL 2018. HEALTH AND JOY FROM FOOD - meal recommendations for early childhood education and care.

NATIONAL NUTRITION SURVEILLANCE CENTRE Childhood Obesity Surveillance Initiative (COSI). [online] available: http://www.ucd.ie/nnsc/research/ourresearch/childhoodobesitysurveillanceinitiativecosi/.

NATIONAL NUTRITION SURVEILLANCE CENTRE Our Aims. [online] Available: http://www.ucd.ie/nnsc/about/ouraims/.

NATIONAL NUTRITION SURVEILLANCE CENTRE. *What we do. [online] Available:* *http://www.ucd.ie/nnsc/about/whatwedo/#d.en.418586* [Online]. [Accessed].
NATIONAL NUTRITION SURVEILLANCE CENTRE 2009. Public Sector Healthy Food Procurement - A review to identify best practice
NATIONAL UNIVERSITY OF IRELAND, G. *Health Behaviour in School-aged Children (HBSC) Ireland. [online] available* *http://www.nuigalway.ie/hbsc/hbscireland/backgroundinformation/* [Online]. [Accessed].
NETHERLANDS, G. O. T. 2019. The National Prevention Agreement.
NETHERLANDS NUTRITION CENTRE 2017. Guidelines for Healthier Canteens Fact sheet. [online] available: https://mobiel.voedingscentrum.nl/Assets/Uploads/voedingscentrum/Documents/Professionals/Pers/Factsheets/English/Fact%20sheet%20Guidelines%20for%20Healthier%20Canteens.pdf.
NEW YORK CITY HEALTH 2017 National Salt Reduction Initiative (NSRI). Available from: https://www1.nyc.gov/site/doh/health/health-topics/national-salt-reduction-initiative.page.
NG, S. W., RIVERA, J. A., POPKIN, B. M. & COLCHERO, M. A. J. P. H. N. 2019. Did high sugar-sweetened beverage purchasers respond differently to the excise tax on sugar-sweetened beverages in Mexico? 22**,** 750-756.
NIKOLAAS TILKIN- FRANSSENS Addressing Obesity through Food Labelling Regulation. [online] available https://edepot.wur.nl/371106.
NORWEGIAN MINISTRY OF LOCAL GOVERNMENT AND MODERNISATION 2019. Fourth Norwegian Action Plan Open Government Partnership (OGP) 2019 - 2021.
NUTRITION AND HEALTH FOUNDATION 2019. National Workplace Wellbeing Day: Working together for a healthier future.
NUTRITION RESOURCE CENTRE 2017. Ontario’s Healthy Menu Choices Act In Effect. [online] available https://opha.on.ca/Nutrition-Resource-Centre/News/NRC-News/2017/Ontario%E2%80%99s-Healthy-Menu-Choices-Act-In-Effect.aspx.
NYC HEALTH DEPARTMENT 2019. National Salt and Sugar Reduction Initiative (NSSRI). .
OFFICE OF THE FEDERAL REGISTER 2013. Food Labeling: Nutrition Labeling of Standard Menu Items in Restaurants and Similar Retail Food Establishments. Available from: https://www.federalregister.gov/regulations/0910-AG57/food-labeling-nutrition-labeling-of-standard-menu-items-in-restaurants-and-similar-retail-food-estab.
OFFICIAL JOURNAL OF THE EUROPEAN UNION 2006. Council Directice 2006/112/EC of 28 November 2006 on the common system of value added tax. [online] available: https://eur-lex.europa.eu/legal-content/EN/TXT/PDF/?uri=CELEX:32006L0112&from=en.
OFFICIAL JOURNAL OF THE EUROPEAN UNION 2012. Consolidated version of the treaty on the functioning of the European Union. [online] available: https://eur-lex.europa.eu/legal-content/EN/TXT/PDF/?uri=CELEX:12012E/TXT&from=EN.
OIREACHTAS 2018. Submission - No Fry Zone 4 Kids committee
OIREACHTAS 2019. Obesity Strategy Dáil Éireann Debate, Tuesday - 12 March 2019. [online] Accessed https://www.oireachtas.ie/en/debates/question/2019-03-12/275/.
PARLIMENT UK 2017. Financial Statement. [online] available on https://hansard.parliament.uk/Commons/2017-03-08/debates/9128238B-4A1C-4EEA-A6F0-6FEB0B5024D8/FinancialStatement#contribution-7B30FFC0-4B7A-4D63-8075-3C7F83EDC5.

PETERS, S., DUNFORD, E., WARE, L., HARRIS, T., WALKER, A., WICKS, M., VAN ZYL, T., SWANEPOEL, B., CHARLTON, K. & WOODWARD, M. J. N. 2017. The sodium content of processed foods in South Africa during the introduction of mandatory sodium limits. 9, 404.

PHILIP MOYLES 2018. No Fry Zone 4 Kids Committee. Submission to Oireachtas Committee on Children and Youth Affairs. [online] available https://data.oireachtas.ie/ie/oireachtas/committee/dail/32/joint_committee_on_children_and_youth_affairs/submissions/2018/2018-08-22_submission-no-fry-zone-4-kids-committee_en.pdf.

POBAL 2016. Healthy Ireland Fund. [online] Accessed on https://www.pobal.ie/programmes/healthy-ireland-fund/.

PUBLIC HEALTH ENGLAND 2017. Sugar Reduction: Achieving the 20% A technical report outlining progress to date, guidelines for industry, 2015 baseline levels in key foods and next steps. .

PUBLIC HEALTH ENGLAND 2018. Sugar Reduction Programme. Progress made by industry in the first year.

PUBLIC HEALTH ENGLAND 2019. Sugar reduction: Report on progress between 2015 and 2018. September 2019.

RATHBONE GREENBANK INVESTMENTS 2019. The UK sugar tax: one year on. [online] available: https://www.rathbonegreenbank.com/insight/uk-sugar-tax-one-year.

REGISTER OF LOBBYING. 2019a. *2019 Legislative Review of the Regulation of Lobbying Act 2015: Submission by the Standards in Public Office Commission. [online] Accessed on https://www.lobbying.ie/about-us/legislation/2019-legislative-review-of-the-regulation-of-lobbying-act-2015-submission-by-the-standards-in-public-office-commission/* [Online]. [Accessed].

REGISTER OF LOBBYING. 2019b. *Best Practices for persons carrying on lobbying activities. [online] Accessed on https://www.lobbying.ie/help-resources/information-for-lobbyists/best-practices-for-lobbying/* [Online]. [Accessed].

REVENUE. 2018. *Sugar Sweetened Drinks Tax (SSDT)* [Online]. Available: https://www.revenue.ie/en/companies-and-charities/excise-and-licences/sugar-sweetened-drinks-tax/index.aspx [Accessed].

REVENUE 2019. Vat on food and drink

ROBERT KOCH INSTITUT. *The AdiMon Indicator System. [online] available: https://www.rki.de/EN/Content/Health_Monitoring/HealthSurveys/AdiMon/AdiMon_inhalt.html* [Online]. [Accessed].

RØYNSTRAND, S. 2017. *Spiser mosjonister og supermosjonister sunt?: En kartlegging av kostholdet til mannlige utholdenhetsmosjonister og supermosjonister.* Universitetet i Agder; University of Agder.

SAFEFOOD 2011 Food on a low income. Four households tell their story.

SAFEFOOD. 2016. *Little Bites (ROI)* [Online]. Available: https://www.safefood.eu/Education/Pre-school/Little-Bites-(ROI).aspx [Accessed].

SAFEFOOD. 2017. *START campaign. [online] accessed on https://www.safefood.eu/Professional/Nutrition/Nutrition-News/Nutrition-News/November-2017/START-campaign.aspx* [Online]. [Accessed].

SAFEFOOD 2018. Tackling Childhood Obesity. [online] Accessed on https://data.oireachtas.ie/ie/oireachtas/committee/dail/32/joint_committee_on_children_and_youth_affairs/submissions/2018/2018-08-22_submission-safefood_en.pdf.

SAUDI FOOD & DRUF AUTHORITY 2018. SFDA launches Healthy Food Regulation Strategy. [online] available: https://www.sfda.gov.sa/en/food/news/Pages/f11-9-2018a1.aspx.

STANDARDS IN PUBLIC OFFICE COMMISSION 2019. 2018 Code of Conduct for persons carrying on lobbying acitivities. [online] Accessed on https://www.lobbying.ie/media/6119/code-of-conduct-english-final-version-for-web.pdf.

TESCO 2008. Customer choice, health and nutrition and Community impact. [online] available: https://www.tesco.ie/crreview08/downloads/tesco_customer_choice_and_community.pdf.

THE CENTRAL GOVERNMENT FOR THE NETHERLANDS 2014. Agreed improvement product composition salt, saturated fat, sugar. [online] available: https://www.rijksoverheid.nl/documenten/convenanten/2014/01/23/akkoord-verbetering-productsamenstelling-zout-verzadigd-vet-suiker.

THE CHIP GROUP 2016. The Chip Group: Helping you make better chips. Available from: http://blog.thechipgroup.co.nz/.

THE COMMISSION OF THE EUROPEAN COMMUNITIES 2006. Commission Directive 2006/125/EC. Processed cereal-based foods and baby foods for infants and young children. Accessed on: https://www.fsai.ie/uploadedFiles/Dir2006_125.pdf.

THE DUTCH INSITITUTE OF PUBLIC HEALTH AND ENVIRONMENT 2017. Agreed improvement product composition salt, saturated fat, sugar. [online] available: https://www.rijksoverheid.nl/documenten/convenanten/2014/01/23/akkoord-verbetering-productsamenstelling-zout-verzadigd-vet-suiker.

THE IRISH TIMES 2019. Restaurant menus must display calorie counts under new proposals. [online] available: https://www.irishtimes.com/life-and-style/food-and-drink/restaurant-menus-must-display-calorie-counts-under-new-proposals-1.3808080.

THE ORGANISATION FOR ECONOMIC CO-OPERATION AND DEVELOPMENT 2019. OECD Reviews of Public Health: Chile: A Healthier Tomorrow, OECD Publishing, Paris.

https://doi.org/10.1787/9789264309593-en.

TUSLA 2018a. Early Years Quality and Regulatory Framework.

TUSLA 2018b. Quality and Regulatory Framework Full Day Care Service and Part-Time Day Care Service.

US NATIONAL INSTITUTES OF HEALTH 2016. Time-Sensitive Obesity Policy and Program Evaluation (R01). Available from: http://grants.nih.gov/grants/guide/pa-files/PAR-12-257.html.

VANDEVIJVERE, S. & SWINBURN, B. 2015. Pilot test of the Healthy Food Environment Policy Index (Food-EPI) to increase government actions for creating healthy food environments. *BMJ Open,* 5.

VEERMAN, J. L. A. L. J. C. 2013 Removing the GST exemption for fresh fruits and vegetables could cost lives. Med J Aust, 199(8): p. 534-5.

WORLD CANCER RESEARCH FUND 2016a. NOURISHING Framework - Harness food supply chain. Available from: http://www.wcrf.org/int/policy/nourishing-framework/harness-food-supply-chain.

WORLD CANCER RESEARCH FUND 2016b. NOURISHING Framework - Improving the food supply. Available from: http://www.wcrf.org/int/policy/nourishing-framework/improve-food-supply.

WORLD CANCER RESEARCH FUND 2016c. NOURISHING Framework - Nutrition labels. Available from: http://www.wcrf.org/int/policy/nourishing-framework/nutrition-labels.

WORLD CANCER RESEARCH FUND 2016d. NOURISHING Framework - Offer healthy foods. Available from: http://www.wcrf.org/int/policy/nourishing-framework/offer-healthy-foods.

WORLD CANCER RESEARCH FUND 2016e. NOURISHING Framework - Restrict food marketing. Available from: http://www.wcrf.org/int/policy/nourishing-framework/restrict-food-marketing.

WORLD CANCER RESEARCH FUND 2016f. NOURISHING Framework - Use economic tools. Available from: http://www.wcrf.org/int/policy/nourishing-framework/use-economic-tools.

WORLD CANCER RESEARCH FUND 2016 NOURISHING Framework - Set retail environment incentives. Available from: http://www.wcrf.org/int/policy/nourishing-framework/set-retail-environment-incentives.

WORLD CANCER RESEARCH FUND 2018. NOURISHING framework. Use economic tools to address food affordability and purchase incentives. [online] available on https://www.wcrf.org/sites/default/files/Use-economic-tools.pdf.

WORLD HEALTH ORGANISATION WHO European Childhood Obesity Surveillance Initiative (COSI). [online] available http://www.euro.who.int/en/health-topics/disease-prevention/nutrition/activities/who-european-childhood-obesity-surveillance-initiative-cosi/about-cosi.

WORLD HEALTH ORGANISATION WHO European Database on Nutrition, Obesity and Physical Activity (NOPA). [online] available http://data.euro.who.int/nopa/.

WORLD HEALTH ORGANISATION 2016. Tackling food marketing to children in a digital world: trans-disciplinary perspectives Children’s rights, evidence of impact, methodological challenges, regulatory options and policy implications for the WHO European Region.

WORLD HEALTH ORGANISATION 2017. France becomes one of the first countries in Region to recommend colour-coded front-of-pack nutrition labelling system.

WORLD HEALTH ORGANISATION 2018. Connecting food systems for co-benefits: How can food systems combine diet-related health with environmental and economic policy goals? [online] available: http://www.euro.who.int/__data/assets/pdf_file/0007/387070/policy-brief-31-austria-eng.pdf?ua=1

WORLD PUBLIC HEALTH NUTRITION ASSOCIATION UPDATE TEAM 2014. Brazilian dietary guidelines. At last! Guidelines based on food and meals!. Available from: http://wphna.org/wp-content/uploads/2015/01/WN-2014-05-12-1050-1051-Update-Guia.pdf.

ANNEX 4B:
THE FOOD-ENVIRONMENT POLICY INDEX (FOOD-EPI): IRELAND
EVIDENCE SUMMARIES

Evidence summary

Food - Environment Policy Index: Policy domains

DOMAIN 1 - FOOD COMPOSITION: There are government systems implemented to ensure that, where practicable, processed foods minimise the energy density and the nutrients of concern (salt, fat, saturated fat, added sugar).

COMP1 - Food composition targets/standards have been established for processed foods by the government for the content of the nutrients of concern in certain foods or food groups if they are major contributors to population intakes of these nutrients of concern (added sugars in processed foods, salt in bread, saturated fat in commercial frying fats).

- A subgroup of the Obesity Policy Implementation Oversight Group (OPIOG) was established with a term of reference to recommend guidelines for food reformulation and a mechanism for engagement with the food industry. This food reformulation subgroup was established in January 2018, is chaired by Prof Ivan Perry, School of Public Health in UCC and is scheduled to report to the Department of Health by Q1 2019.
- In Step three of the '*A Healthy Weight for Ireland: Obesity Policy and Action Plan 2016 -2025',* the Irish Government planned to agree food reformulation targets with the food industry (Department of Health., 2016). Leads were identified and deemed responsible for this step including, the Department of Health, Irish Business and Employers' Confederation, Food Drink Ireland (FDI) and the Food Safety Authority of Ireland (FSAI), Department of Agriculture, Food and the Marine as a potential partner.
- In January 2016, Food and Drink Ireland (FDI) issued a first report estimating the impact of reformulation by some of its food industry members on the Irish population (Food Drink Ireland, 2016).
- The FDI published a further report in 2019 entitled "The Evolution of Food and Drink in Ireland, 2005 – 2017" that showed, the decreases in sugar and saturated fat in Irish diets between 2005 and 2017 as a result of voluntary undertakings by food and drink companies.
- Results for the other nutrients were more modest, with sodium, total fat and energy intake remaining relatively stable over the period (Food and Drink Ireland, 2019).

**Direct reformulation of products on the market in both 2005 and 2017**	**Reductions**
Sodium	28%
Saturated fat	10.1%
Sugar	8%
Energy	1.6%
Total fat	0.3%
**Reductions in sugar intake between 2005 and 2017**	**Reductions**
Adult sugar intake	0.8g/day
Teen sugar intake	2.7g/day
Child sugar intake	3.2g/day
Pre-schooler sugar intake	2.0g/day
**Reductions in saturated fat intake between 2005 and 2017:**	**Reductions**
Adult saturated fat intake	0.5g/day
Teen saturated fat intake	0.2g/day
Child saturated fat intake	0.2g/day
Pre-schooler saturated fat intake	remained constant

- Coupled with this industry-led reformulation programme will be a continuation of the FSAI's independent surveillance and commentary on the commitments and achievements of the industry in relation to salt reduction

- The FSAI published an update of its annual salt monitoring programme in November 2016. An examination of 530 samples of processed food was conducted in 2015, across four food product categories including: processes meats, breads, breakfast cereals and spreadable fats. Significant reductions in salt were found across a variety of products, most notably in processed meats such as rashers, cooked ham and sausages. However, while levels of salt have decreased in processed foods, the average dietary salt intake in Irish adults is currently 11.1 g salt per day in men and 8.5 g salt per day in women (Food Safety Authority of Ireland, 2016b).

COMP2 - Food composition targets/standards/restrictions have been established by the government for the content of the nutrients of concern (trans fats, added sugars, salt, saturated fat) in meals sold from food service outlets, in particular for those food groups that are major contributors to population intakes of those nutrients of concern.

- **No evidence found from 2016 onwards**

DOMAIN 2 - FOOD LABELLING: There is a regulatory system implemented by the government for consumer-oriented labelling on food packaging and menu boards in restaurants to enable consumers to easily make informed food choices and to prevent misleading claims.

LABEL1 - Ingredient lists and nutrient declarations in line with Codex recommendations are present on the labels of all packaged foods

- Label 1 will not be including for benchmarking at national level as it's being addressed at EU level

LABEL2 - Evidence-based regulations are in place for approving and/or reviewing claims on foods, so that consumers are protected against unsubstantiated and misleading nutrition and health claims.

- Label 2 will not be including for benchmarking at national level as it's being addressed at EU level

LABEL3 - One or more interpretive, evidence-informed front-of-pack supplementary nutrition information system(s) endorsed by the Government, which readily allow consumers to assess a product's healthiness, is/are applied to all packaged foods (examples are the NutriScore and traffic lights

- Currently there isn't any work being done to consider the implementation of an interpretive, evidence informed front of pack supplementary nutrition information system in Ireland**.**

- According to FSAI, Front of Pack (FoP) labelling is a voluntary initiative that gives the consumer a quick glimpse of 5 main nutrients in the product, namely calories, sugar, fats, saturates (saturated fat), and salt. Where the mandatory nutrition information is declared on the label, certain nutrients may be repeated in the 'principal field of vision', i.e. the front of pack. This repeated information is a voluntary measure but where a food business chooses to provide this additional declaration, only the following information can be provided: Energy only or energy along with fat, saturates, sugar and salt.

- **This repeated information may be provided:**
  - Per 100g/ml only
  - Per 100g/ml and per portion or
  - On a per portion basis only
  - When providing this 'Front of Pack' information Energy must always be indicated per 100g/ml as a minimum.

LABEL4 - A simple and clearly-visible system of labelling the menu boards of all quick service restaurants (i.e. fast food chains) is applied by the government, which allows consumers to interpret the nutrient quality and energy content of foods and meals on sale.

- In Step two of the 'A *Healthy Weight for Ireland: Obesity Policy and Action Plan 2016-2025'*, the Irish Government plan to *"regulate for a healthier environment"* by developing, implementing and evaluating calorie posting legislation. This will be led by the Department of Health with input from public sector organisations, with the view of implementing evidence informed regulation. The timeline is 2016-2025 (Department of Health., 2016).

- At present, calorie posting legislation is currently in preparation(The Irish Times, 2019).

- Food service businesses have been provided with initial support to help them implement calorie menu labelling. MenuCal, the menu calculator has been developed to assist food businesses in Ireland comply with their legal requirements to display allergen information on foods. It also enables food businesses to calculate calories for their menus. It has been developed with the input of chefs, caterers and small business owners (Food Safety Authority of Ireland, 2019).

DOMAIN 3 - FOOD PROMOTION: There is a comprehensive policy implemented by the government to reduce the impact (exposure and power) of promotion of unhealthy foods to children across all media.

PROMO1 - Effective policies are implemented by the government to restrict exposure and power of promotion of unhealthy foods to children including adolescents through broadcast media (TV, radio).

General Commercial Communications Code

- The General Communications Code came into effect on the 1st of June 2017(Broadcasting Authority of Ireland, 2017).

Children's Commercial Communications Code

- As provided under the Broadcasting Act 2009, the BAI is required to undertake a statutory review of the effectiveness of the Children's Code. It is expected that review will commence in the second half of 2018 with revision and finalisation of the Code in 2019 (Broadcasting Authority of Ireland, 2019)

PROMO2 - Effective policies are implemented by the government to restrict exposure and power of promotion of unhealthy foods to children including adolescents through online and social media.

Government of Ireland – voluntary codes of practice

- The Government of Ireland has issued a set of voluntary codes of practice aimed at limiting the promotion, marketing and sponsorship of foods high in fats, sugar and/or salt (HFSS foods). The new voluntary rules will apply to non-broadcast media, including digital, out of home, print and cinema, as well as commercial sponsorship and retail product placement. The government of Ireland has committed to monitor compliance, and work is continuing on the development of a monitoring mechanism (Department of Health, 2017).

Rules for Non-Broadcast Digital Media

- Where appropriate age-filters exist on websites and social media apps, marketing communications for HFSS foods are not permitted to target children under the age of 15.

- Marketing Communications for HFSS food by means of e-mail and Short Message Service (SMS) shall not target children under the age of 15.
- Marketing Communications for HFSS food by means of social media shall not target children under the age of 15.
- Where Marketing Communications for HFSS food is permissible, it shall not exceed a maximum of 25% of total advertising space.
- The websites of food businesses should not carry content that is designed to engage children under the age of 15 with HFSS food brands e.g. children's area, videos, 'webisodes', branded education and interactive features (Department of Health, 2017).

PROMO3 - Effective policies are implemented by the government to restrict exposure and power of promotion of unhealthy foods to children including adolescents through non-broadcast media other than packaging and online/social media.

Non-Broadcast Media Advertising and Marketing of Food and Non-Alcoholic Beverages, including Sponsorship and Retail Product Placement: Voluntary Codes of Practice (December, 2017)

- **Rules for Out of Home Media** - Out of Home Media includes all out of home advertising and marketing communications delivered via such formats as billboards or hoardings, public transport stops or shelters, interiors and exteriors of buses or trains, or building banners (Healthy Ireland, 2017b).

1. Space limitation across the various HFSS product categories will be a total of 33% of the available space by cycle2 and by format.
2. Marketing Communications for HFSS food is not allowed on building banners.
3. Marketing Communications utilising wraparounds or takeovers for HFSS foods will account for less than 5% of the total available advertising space (Healthy Ireland, 2017b).

Additional Rules for Print Media

1. Marketing Communications for HFSS foods will only be carried in consumer publications where the adult readership is 75% or greater. A consumer publication or issue is taken to mean the complete edition published that day to include any supplements or advertising inserts.
2. Where Marketing Communications for HFSS food is permissible, it shall not, in ordinary circumstances, exceed a maximum of 25% of total advertising space.
3. HFSS food sponsorship of sports pages or sports supplements is not allowed (Healthy Ireland, 2017b).

Additional Rule for Cinema

1. Where Marketing Communications for HFSS food is permissible, it shall not exceed a maximum of 25% of total advertising space by screening (Healthy Ireland, 2017b).

Code of Practice Relating to Retail Product Placement of HFSS Foods

2. The arrangement of food products in the retail environment influences the purchase decisions of consumers. It is noted that the retail food industry acknowledges this fact and has agreed to a set of product placement measures that are designed to increase healthy food choices. Nevertheless, the retail industry is encouraged to go above and beyond these measures in the interests of promoting healthy eating. Accordingly, the retail food industry in Ireland undertakes to operate the specific rules for retail product placement as detailed below in addition to any relevant general rules applicable to all codes.
3. Active promotion of the consumption of 5 to 7 portions of fruit and vegetables a day by customers in store through point of sale materials and other online and offline communication platforms.
4. Provision of meal deal offers that deliver choice for customers in order to promote a healthy balanced diet.
5. Where practical, the provision of a HFSS food free checkout option to customers to allow the use of a checkout lane that does not stock HFSS foods. In retail environments with 4 or more checkout bays, a minimum of 1 in 4 bays should be free of HFSS foods. Other outlets with less than 4 checkouts are encouraged to provide 1 non-HFSS checkout, where practical.
6. Provision of calorie labelling information in food service areas such as delis and hot food counters. Provision of calorie labelling will be in line with legal requirements and in their absence with 'Putting Calories on Menus in Ireland - Draft Technical Guidance for Food Businesses', is available free from the Food Safety Authority of Ireland website (www.fsai.ie) (Healthy Ireland, 2017b)

PROMO4 - Effective policies are implemented by the government to ensure that unhealthy foods are not commercially promoted to children including adolescents in settings where children gather (e.g. preschools, schools, sport and cultural events).

Non-Broadcast Media Advertising and Marketing of Food and Non-Alcoholic Beverages, including Sponsorship and Retail Product Placement: Voluntary Codes of Practice (December, 2017)

General Rules for all Codes of Practice

- Locations primarily used by children shall be free from all forms of marketing communication for HFSS foods. Examples of such settings include registered crèches, pre-schools, nurseries, family and child clinics, paediatric services, schools, dedicated school transport, playgrounds and youth centres.

Rules for Out of Home Media

Displays of HFSS foods will be restricted from 100 metres of school gate for large roadside billboard formats which include but is not limited to 48 sheet sizes3 and larger for example and 60 metres for 6 sheet sizes and particular attention will be given to HFSS foods that particularly appeal to children(Healthy Ireland, 2017b).

PROMO5 - Effective policies are implemented by the government to ensure that unhealthy foods are not commercially promoted to children, including adolescents on food packages.

- **No further evidence found from 2016 onwards**

DOMAIN 4 - FOOD PRICES: Food pricing policies (e.g., taxes and subsidies) are aligned with health outcomes by helping to make the healthy eating choices the easier, cheaper choices

PRICES1 - Taxes or levies on healthy foods are minimised to encourage healthy food choices (e.g. low or no sales tax, excise, value-added or import duties on fruit and vegetables).

- The Zero rate of VAT applies to the supply of most foodstuffs, such as bread, butter, cheese, cereals, condiments, flour, fruit, herbs, meat, milk, pasta, pastes, sauces, soup, spices, sugar, and vegetables (fresh or frozen). This list is by no means exhaustive. However, it should be particularly noted that the supply of food and drink that would normally be liable to VAT at the Zero rate becomes liable to VAT at the Second Reduced rate when it is supplied in the course of catering or by means of a vending machine (Revenue, 2019)

PRICES2 - Taxes or levies on unhealthy foods (e.g. sugar-sweetened beverages, foods high in nutrients of concern) are in place and increase the retail prices of these foods by at least 10% to discourage unhealthy food choices, and these taxes are reinvested to improve population health.

- On 1 May 2018, the Republic of Ireland's Sugar Sweetened Drinks Tax came into force under the Finance Act 2017 (No. 41 of 2017). The tax applies to non-alcoholic, water-based and juice based drinks which have added sugar content of 5g per 100mL and above. Drinks with over 8g of sugar per 100mL are taxed at 30 cents per litre, and drinks with between 5g and 8g of sugar per 100mL are taxed at 20 cents per litre. Fruit juices and dairy products are excluded from the tax (Revenue, 2018).

PRICES3 - The intent of existing subsidies on foods, including infrastructure funding support (e.g. research and development, supporting markets or transport systems), is to favour healthy rather than unhealthy foods.

- The Scheme of Aid for Producer Organisations in the Fruit & Vegetables Sector provides aid to producers grouping, on a voluntary basis, to form recognised Producer Organisations (POs) and implement approved operational programmes that improve the quality and efficiency of operations. One objective being to Increase the consumption of fruit and vegetables in the Community (Department of Agriculture Food and Marine, 2018).

- The 2019 Scheme of Investment Aid for the Development of the Commercial Horticulture Sector. Aims to facilitate environmentally friendly practices, promote the diversification of on-farm activities, improve the quality of products (Department of Agriculture Food and Marine, 2019).

- Beef Data and Genomics Programme (BDGP) - aimed at delivering accelerated genetic improvement in the national herd and improvement of its environmental sustainability (Department of Agriculture Food and Marine, 2017).

PRICES4 - The government ensures that food-related income support programs are for healthy foods

- **No evidence found from 2016 onwards**

DOMAIN 5 - FOOD PROVISON: The government ensures that there are healthy food service policies implemented in government-funded settings to ensure that food provision encourages healthy food choices, and the government actively encourages and supports private companies to implement similar.

- Nutritional Standards for School Meals were launched by the government in 2017 to ensure that schools follow National Healthy Eating Guidelines.
- The HSE and HIQA are responsible for the Review of Nutrition and Hydration Care in Public Acute Hospitals. HIQA recommended that "all hospitals should have a nutrition steering committee in place" in order to improve nutrition and hydration in acute hospitals.
- Under the *Childcare Regulations 2016,* pre-school providers must ensure that children are given regular drinks and food in adequate quantities for their needs, in consultation with parents where concerns exist.
- Evidence shows that workplace health initiatives such as the Healthy Eating Awards, Active@Work Programme, Sli@Work and Health Checks deliver a wide range of benefits, to both employers and staff, and encourage healthier food choices and lifestyles.

PROV1 - The government ensures that there are clear, consistent policies (including nutrition standards) implemented in schools and early childhood education services for food service activities (canteens, food at events, fundraising, promotions, vending machines etc.) to provide and promote healthy food choices.

Department of Employment Affairs and Social Protection – Hot School Meals Pilot Project (2019)

- As part of Budget 2019, Minister Doherty announced that hot dinners will be provided on a pilot basis in up to 36 schools to some 7,200 children from September 2019 at a cost of €1m for 2019 and €2.5m in a full year. The pilot will initially be targeted at Primary Schools which do not currently avail of the dinner option under the Department of Employment Affairs and Social Protection's School Meals Programme and do not have canteen/kitchen facilities for preparation of the hot dinners on site.

- On Monday, 28 January, 2019: Minister for Employment Affairs and Social Protection, Regina Doherty, T.D. launched a Hot School Meals scheme for Our Lady of Lourdes Primary School in Golden bridge, Inchicore. This is the first Hot School Meals scheme to be introduced and it will serve to road-test the idea and identify any issues before a larger pilot initiative is rolled out later this year. During this initial pilot, almost 250 pupils attending this school will receive a hot meal at lunchtime each day. It will run until the end of the current school year (end June)(Department of Employment Affairs and Social Protection, 2019)

'Healthy Weight for Ireland': Obesity Policy and Action Plan 2016-2025'.

- Action 1.6 in the *OPAP* sets also out to *"examine the expansion of current effective programmes, such as Food Dudes and The Incredible Edibles and develop further opportunities for collaboration with other government departments and state agencies in the promotion of fresh produce and its role in a healthy, balanced diet".*

The Department of Education and Skills and the Department of Health and Children, through the Healthy Ireland framework, have joined Agri Aware's incredible Edibles patrons along with the Department of Agriculture, Food and Marine, Bord Bia and the horticulture industry to support this important healthy eating educational programme for primary level students (Agri Aware's Incredible Edibles).

- Action 1.4 in the *Obesity Policy and Action Plan* also recommends “providing potable water in all learning centres (from preschool and crèches to universities and adult learning centres) and ensure all new buildings provide potable water on opening. Child care regulations in 2006 by DCYA state that potable drinking water should be provided to children at all times, however there is no monitoring yet or evidence that progress has been made (Department of Health, 2016c).

- Eat Smart Move More was launched by the HSE in 2017. The booklet provides guidelines for healthy eating and physical activity for children. It provides ideas on ways to make exercising more fun and enjoyable and provides a variety of different meal and snack ideas. The initiative is set for review in 2020 (Health Service Executive, 2017a)

Nutrition Standards for School Meals (2017)

- In 2017, the new Nutrition Standards for School Meals were launched by the Minister for Health, the Minister for Education and Skills and the Minister for Employment Affairs and Social Protection. These Nutrition Standards are adapted from the Standards published to support the Food in Schools Policy in Northern Ireland. This work was coordinated by the Health and Wellbeing Unit, Department of Health and undertaken by a working group with representatives from safefood and the Healthy Eating Active Living (HEAL) programme in the Health Service Executive, in cooperation with members of the School Meals Programme in the Department of Employment Affairs and Social Protection and the Department of Education and Skills (Healthy Ireland, 2017a).
- The School Meals (Local Projects) Scheme, is an administrative scheme, operated directly by the Department of Employment Affairs and Social Protection (Healthy Ireland, 2017a). The Scheme provides funding to primary and post-primary schools, local groups, voluntary organisations and community-based not-for-profit preschools operating their own school meals projects. The ‘Nutrition Standards for School Meals’, are being implemented under this scheme and aim to ensure that children and young people in schools participating in the scheme are provided with healthy balanced meals that follow the Healthy Eating Guidelines.

- These Nutrition Standards are food-based, and are provided for each meal type funded by the Scheme, that is: Breakfast or snack Lunch or after-school meal Dinner, only healthy food choices that meet the standards will be funded. The Standards will also be used by those administering the Scheme in the schools, commencing in January 2018, to ensure that food purchased complies with the Nutrition Standards when food contracts are being specified in the procurement process, and should also be applied when planning menus (Healthy Ireland, 2017a)

The Nurture Programme (2016)

- The Nurture Programme – Infant Health and Wellbeing, launched on the 23rd May 2016, is a partnership between the Health Service Executive, Atlantic Philanthropies, Katharine Howard Foundation and Centre for Effective Services.

- It is a programme of work which builds on the existing child health programme, strengthening a cohesive, integrated child health and wellbeing service for children aged 0-2 years and their families. The aim of the programme is to support parents and healthcare professionals in their caring and service provisions. (Health Service Executive, 2016).
- In December 2018, Minister for Health Simon Harris TD launched new HSE resources for parents on December 6th including the mychild.ie website. This is a new website for parents which contains information and advice on pregnancy and the first 3 years of your child's life. The website is based on the most-up-date information available within the health service on pregnancy, parenting and child health. It was developed in conjunction with a wide range of health care professional including midwives, public health nurses, doctors, psychologists and dietitians. In addition, the website also focuses on the experience and knowledge of parents themselves, with over 4,000 parents across Ireland involved in the design of mychild.ie. (Health Services Executive, 2018).

Childcare Regulations (2016)

- Under the *Childcare Regulations 2016,* pre-school providers must ensure that children are given regular drinks and food in adequate quantities for their needs, in consultation with parents where concerns exist (Tusla, 2018b). The food and drink supplied should be nutritious, varied and should take account of the Food and Nutrition Guideline for Pre-School Services as prepared by the Department of Health and Children. The guidelines recommend the following:

- *Children in day care for more than 5 hours per session (full day care);* Offer at least two meals (one hot) and two snacks. Snacks such as fruit, raw vegetables, or plain crackers

are good, but sugary snacks are to be avoided. For example- breakfast, snack, lunch and snack. An evening meal may be provided for children staying for a longer day.

- *Children in day care for a maximum of 5 hours per session (part-time day care);* Offer at least two meals and one snack, for example- breakfast, snack, and lunch.

- *Children in day care for up to 3.5 hours per session (sessional pre-school service);* Offer one meal and one snack, for example; snack and lunch or breakfast and snack. Good snacks include raw vegetables, fruits, a glass of milk and plain crackers. Sugary snacks are to be avoided.

PROV2 - The government ensures that there are clear, consistent policies in other public sector settings for food service activities (canteens, food at events, fundraising, promotions, vending machines, public procurement standards etc.) to provide and promote healthy food choices.

Healthy Weight for Ireland Obesity Policy and Action Plan

- In Step 6 of the Healthy Weight for Ireland Obesity Policy and Action Plan, the Irish Government plan to "review and improve the quality of food in hospitals and develop a food and nutrition policy for hospitals". The key leads responsible for this review are the HSE and HIQA (Department of Health, 2016c)

The HSE Food, Nutrition and Hydration Policy for Acute Hospitals was launched in 2019.

- The purpose of this Policy is to provide a national framework for food, nutritional care and hydration provision for adult patients in acute hospitals.

- The Policy will apply to all staff involved in the provision and delivery of food, fluids and nutritional care for patients. All staff involved in the provision of nutritional care should use the Policy recommendations to review and develop services. Staff refers to clinical (medical, nursing, health care assistants, health and social care professionals) and non- clinical staff including catering services, catering support services and hospital managers. All locations in acute hospitals are included, Inpatients, Emergency Department and Day Procedures Units (Health Service Executive, 2018).

The HSE Vending Policy 2019

- The HSE Vending Policy 2019 replaces the 2014 Healthier Vending Policy and applies to all vending machines that stock cold soft drinks, confectionery and snacks on HSE premises & premises funded by the HSE.

- It was developed in 2018 by the HSE Healthy Eating Active Living Programme and was informed by the HSE Healthier Vending Policy Assessment of Compliance Report 2018. A technical sub group was established to consider the nutrient profile of vending machine products (Health Service Executive, 2019).

- **Beverages:** Sugar sweetened beverages will not be stocked in vending machines, 50% of beverages stocked will be still water and remaining beverages stocked will include non-sugar sweetened beverages e.g. diet drinks, juices, flavoured and sparkling water.

- **Snacks:** Snacks containing more than 200 calories per packet will not be stocked in machines and an exception to this is 3 packets of dried fruits, nuts or seeds (plain and unsalted)

- **Calorie posting:** Products will be clearly labelled with the number of calories per product

PROV3 - The government ensures that there are clear, consistent public procurement standards in public sector settings for food service activities to provide and promote healthy food choices.

- In Ireland there is no healthy food procurement policy in place for the public sector. A public sector healthy procurement policy would aim to encourage public bodies to serve food that meets objectives on issues such as health, nutrition and waste (National Nutrition Surveillance Centre, 2009)

PROV4 - The Government ensures that there are good support and training systems to help schools and other public sector organisations and their caterers meet the healthy food service policies and guidelines

Nutritional Standards for School Meals

- The Nutritional Standards for School Meals were designed to ensure the School Meals programmes follow the national Healthy Eating Guidelines (Healthy Ireland, 2017a).

- The 3-week menu plan resource, implemented by the HSE provides a practical guide for pre-schools to carry out the guidelines and to provide balanced, nutritious meal ideas. All recipes are suitable for children from 1 year onwards and the portion sizes are based on the requirements of children aged 1½ - 3 years

Food and nutrition guidelines for pre-school services

- These guidelines are produced by the Department of Health that, assist pre-schools in providing healthy food. They also help in creating an environment that promotes positive attitudes to eating and physical activity as part of a healthy lifestyle. They are relevant to pre-school children aged 0-5 years and are intended as a resource and guide for all relevant stakeholders which include carers, parents and pre-school inspectors. Currently, they're being developed into nutrition standards 2020

Early years Quality and Regulatory Framework

- The Early Years Inspectorate has developed a Quality and Regulatory Framework (QRF), to support registered Early Years Services to comply with the 2016 Regulations. The QRF does this by setting out the core regulatory requirements in a transparent way, bringing together evidence-based, national and international research and best practice in Early Years. The QRF aims to support registered providers in achieving compliance with the regulations and enhance the safety and care of children who attend these services. The QRF is child-centred, with a specific focus on the quality and safety of the care provided directly to children using the services. Tusla Early Years Inspectorate, in collaboration with the Department of Children and Youth Affairs, launched the Quality and Regulatory Framework on Wednesday the 5th of September 2018 in Dublin (TUSLA, 2018a).

First 5: whole-of-Government strategy

- First 5 is a whole-of-Government strategy to improve the lives of babies, young children and their families. It is a ten-year plan that, uses evidence to identify goals, objectives and the specific actions required from across Government to support children (and their families) in the early years of life (Department of Children and Youth Affairs, 2018a). First 5 commits to major initiatives on;

1. broader range of options for parents to balance working and caring
2. a new model of parenting support
3. new developments in child health, including a dedicated child health workforce
4. reform of the Early Learning and Care (ELC) system, including a new funding model
5. a package of measures to tackle early childhood poverty

PROV5 - The Government actively encourages and supports private companies to provide and promote healthy foods and meals in their workplaces

A Healthy Workplaces Framework

- A Healthy Workplaces Framework is currently being developed as part of the governments public health Framework, Healthy Ireland - A Framework for improved health and wellbeing: 2013 – 2025 (Department of Health, 2013). This Framework is being developed by the Department of Health and the Department of Business, Enterprise and Innovation (McAvoy, 2018). It is intended to provide a supportive, flexible, evidence-based structure that can be applied across all workplaces in Ireland regardless of their sector or size. The Healthy Workplaces Framework is due to be implemented in 2020

DOMAIN 6 - FOOD IN RETAIL: The government has the power to implement policies and programs to support the availability of healthy foods and limit the availability of unhealthy foods in communities (outlet density and locations) and in-store (product placement)

RETAIL1 - Zoning laws and policies are implemented to place limits on the density or placement of quick serve restaurants or other outlets selling mainly unhealthy foods in communities and/or access to these outlets (e.g. opening hours).

A Healthy Weight for Ireland: Obesity Policy and Action Plan 2016 – 2025'

- Action 2.1 of the Obesity Policy and Action Plan states "Develop guidelines and support materials for those working in developing the built environment for urban development and planning in relation to reducing the obesogenic environment". In line with this Action, and with Action 31 of the National Physical Activity Plan (NPAP) (Department of Health, 2016a), which commit to developing guidelines and support materials for those working in developing the built environment in order to promote the importance of physical activity, and Action 59 of the NPAP which commits to developing a programme of on-going stakeholder communication and engagement, a Stakeholder Forum was held on November 21st, 2018.

- This Forum, entitled *Connect, Collaborate, Create; Co-Designing Healthier Communities, A Healthy Ireland Stakeholder Forum on the Built Environment*, brought together those who have a responsibility for planning and developing public spaces with those who have expertise regarding the impacts of the built environment on health and wellbeing. It provided an opportunity for interdisciplinary discussion on how enhanced collaboration might be supported in the future, to create healthier communities (Oireachtas, 2019).

RETAIL2 - Zoning laws and policies are implemented to encourage the availability of outlets selling fresh fruit and vegetables and/or access to these outlets (e.g. opening hours, frequency i.e. for markets)

Department of Agriculture, Food and Marine

- There is no specific planning legislation in place in relation to encouraging outlets to sell fresh fruit and vegetables. However, the Department of Agriculture, Food and Marine provided a Code for Good Practice for Farmers Markets in 2013. The code of good practice is intended as a basic standard to promote the vision of- offering a route to market for local produce and small food producers, attracting consumers and promoting sustainable and diverse food cultures at county and local level. (Department of Agriculture Food and Marine and An Bord Bia, 2009)

- No further evidence found from 2016 onwards

RETAIL3 - The Government ensures existing support systems are in place to encourage food stores to promote the in-store availability of healthy foods and to limit the in-store availability of unhealthy foods

Non-Broadcast Media Advertising and Marketing of Food and Non-Alcoholic Beverages, including Sponsorship and Retail Product Placement: Voluntary Codes of Practice

- Under the codes, the retail food industry in Ireland undertakes to operate the specific rules for retail product placement as detailed below in addition to any relevant general rules applicable to all codes:

1. Active promotion of the consumption of 5 to 7 portions of fruit and vegetables a day by customers in store through point of sale materials and other online and offline communication platforms.
2. Provision of meal deal offers that deliver choice for customers in order to promote a healthy balanced diet.
3. Where practical, the provision of a HFSS food free checkout option to customers to allow the use of a checkout lane that does not stock HFSS foods. In retail environments with 4 or more checkout bays, a minimum of 1 in 4 bays should be free of HFSS foods. Other outlets with less than 4 checkouts are encouraged to provide 1 non-HFSS checkout, where practical.
4. Provision of calorie labelling information in food service areas such as deli's and hot food counters (Healthy Ireland, 2017b).

RETAIL4 - The government ensures existing support systems are in place to encourage the promotion and availability of healthy foods in food service outlets and to discourage the promotion and availability of unhealthy foods in food service outlets

Alcoholic Beverages, including Sponsorship and Retail Product Placement: Voluntary Codes of Practice

- Under the codes, the retail food industry in Ireland undertakes to operate the specific rules for retail product placement as detailed below in addition to any relevant general rules applicable to all codes:

1. Active promotion of the consumption of 5 to 7 portions of fruit and vegetables a day by customers in store through point of sale materials and other online and offline communication platforms.
2. Provision of meal deal offers that deliver choice for customers in order to promote a healthy balanced diet.
3. Where practical, the provision of a HFSS food free checkout option to customers to allow the use of a checkout lane that does not stock HFSS foods. In retail environments with 4 or more checkout bays, a minimum of 1 in 4 bays should be free of HFSS foods. Other outlets with less than 4 checkouts are encouraged to provide 1 non-HFSS checkout, where practical.
4. Provision of calorie labelling information in food service areas such as deli's and hot food counters (Healthy Ireland, 2017b)

Healthy Food Environment Policy Index: Infrastructure domains

DOMAIN 8 – LEADERSHIP: The political leadership ensures that there is strong support for the vision, planning, communication, implementation and evaluation of policies and actions to create healthy food environments, improve population nutrition, and reduce diet-related inequalities

LEAD1 - There is strong, visible, political support (at the head of government or state/ ministerial level) expressed at both national as well as international level for improving food environments, population nutrition, diet related NCDs and their related inequalities.

Healthy Ireland "A Framework for Improved Health and Wellbeing 2013-2025"

- The Healthy Ireland Framework was launched in 2013 with a Foreword by the then Taoiseach Enda Kenny and an Introduction by the then Minister for Health Dr. James Reilly, T.D. It articulates four central goals for improved health and wellbeing: increase the proportion of people who are healthy at all stages of life, reduce health inequalities, protect the public from threats to health and wellbeing and create an environment where every individual and sector of society can play their part in achieving a healthy Ireland
- The Cabinet Committee on Social Policy oversees the delivery of this Framework. The Health and Wellbeing Programme in the Department of Health is responsible for strategic planning and co-ordination of the implementation of the Framework actions. A multi-stakeholder Healthy Ireland Council was established to provide a national advisory forum to support the implementation of the Framework across sectors. The Framework was accompanied later in 2013 by a high-level implementation plan and an Outcomes Framework (Department of Health, 2013)

'A Healthy Weight for Ireland, Obesity Policy and Action Plan 2016 – 2025'

- The OPAP was launched with a Foreword by the Minister for Health, Simon Harris TD and a Foreword by the then Minister of State for Health Promotion, Marcella Corcoran Kennedy TD. The policy sets out "Ten Steps Forward" which aim at reversing the obesity trend while preventing complications associated with obesity and reducing the overall burden for individuals, their families and the health system. The "Ten Steps Forward" are:

1. Embed multi-sectoral actions on obesity prevention with the support of government departments and public sector agencies.
2. Regulate for a healthier environment.
3. Secure appropriate support from the commercial sector to play its part in obesity prevention.
4. Inform and empower change through a clear communications strategy.
5. The Department of Health will provide leadership.

6. Mobilise the health services with a focus on prevention.
7. Develop a service model for specialist care for children and adults.
8. Acknowledge the key role of physical activity in the prevention of overweight and obesity.
9. Allocate resources according to need in particular for children and disadvantaged groups.
10. Monitor research and review.

Healthy Ireland fund

- In 2016, the Government approved the creation of a Healthy Ireland Fund with an initial allocation of €5 million approved in Budget 2017 to establish and support the implementation of Healthy Ireland programmes and projects in a variety of settings. The primary aim of the fund is to support innovative, cross-sectoral, evidence-based projects and initiatives that support the implementation of key national policies in areas such as obesity, smoking, alcohol, physical activity and sexual health. The funding has been allocated to 2 Strands, both of which are closed calls for funding. Strand 1 focuses on the delivery of local initiatives and Strand 2 funds national actions (Pobal, 2016).

- The Department of Health has approved a third round of funding for the Healthy Ireland Fund (HIF). The aim of this funding is to support local and national organisations to deliver actions that will improve health and wellbeing in line with *Healthy Ireland, A Framework for Improved Health and Wellbeing 2013-2025* (Department of Health, 2019b).

Healthy Ireland campaign (2019)

- Taoiseach Leo Varadkar TD, Minister for Health Simon Harris TD and Minister for Health Promotion, Catherine Byrne TD have launched the 2019 Healthy Ireland campaign and announced funding of €1 million to boost community engagement on health and wellbeing in every county. The 2019 campaign seeks to raise public awareness of gov.ie/HealthyIreland as the place to go that brings trusted sources of information and supports together in one place to help people get started on making healthy changes.(Department of Health, 2019d)

National Healthy Cities and Counties of Ireland Network

- National Healthy Cities and Counties of Ireland Network was launched in 2016 by the Minister for Health Promotion. The network was launched under the Healthy Ireland "A Framework for Improved Health and Wellbeing 2013-2025", and has

representatives from government departments, the HSE, the Institute of Public Health, the Federation of Irish Sport and local political and community representative (Department of Health, 2019c)

LEAD2 - Clear population intake targets have been established by the government for the nutrients of concern and / or relevant food groups to meet WHO and national recommended dietary intake levels

Department of Health and Food Safety Authority of Ireland recommendations

- The population nutrition goals have been broadly adopted by the Department of Health and the Food Safety Authority of Ireland, in line with WHO/FAO recommendations as follows; Less than 10% of daily energy from saturated fats, less than 1% of daily energy from trans fats, less than 10% of daily energy from added sugars progressively reducing to 5%*, More than 400g fruits and vegetables a day and less than 5g a day of salt (6 g population recommendation FSAI 2016).

Healthy Ireland and Department of Health

- Together have established 'Eat Well'. Clear dietary guidelines using the Food pyramid on the maximum daily intake for foods and drinks high in fat, sugar and salt are established. It states, "*There are no recommended servings for this shelf because they are not needed for good health. Not every day – maximum once or twice a week*".

- Food guides have also been created to show people how to use the pyramid on a daily basis. For e.g. a guide for foods high in fat, sugar and salt has been created which contains a guide for portion size, cooking tips and shelf life for these foods, based on the food pyramid (Department of Health, 2019a).

LEAD3 - Clear, interpretive, evidenced-informed food based dietary guidelines have been established and implemented.

Department of Health – Healthy Eating Guidelines and the revised Food Pyramid

- 'A Healthy Weight for Ireland', Obesity Policy and Action Plan 2016–2025 developed a suite of Healthy Eating Guidelines for the general population. These revised Healthy Eating Guidelines and Food Pyramid resources, are an early action under the Plan (Action 5.3.) The revised Healthy Eating Guidelines and Food Pyramid are based on the FSAI Scientific Recommendations for Healthy Eating Guidelines in Ireland from 2011 (Food Safety Authority of Ireland, 2011). They describe how to build a healthy diet, for different age groups (from 5 years of age), depending also on gender and activity levels. The Guidelines, Food Pyramid and supporting resources have been published, disseminated and communicated in 2017, including dissemination of the new Guidelines to all primary and post-primary schools (Healthy Ireland, 2016a).

- The revised Healthy Eating Guidelines and Food Pyramid toolkit has been developed by the Department of Health and the Health Service Executive with key stakeholders and aims to help reduce the intake of high fat, salt and sugar (HFSS) foods and drinks from the Top Shelf of the Food Pyramid (Healthy Ireland, 2016a). **The revised Food Pyramid model main revisions are;**
- A major focus on limiting Top Shelf Foods to only once or twice a week
- Increasing the servings from the Vegetables, Salad and Fruit shelf from 5 a day up to 7. This means that the Vegetables, Salad and Fruit Shelf is the largest shelf and moves to the bottom of the Pyramid
- The Whole meal Cereals and Breads, Potatoes, Pasta and Rice Shelf then moves up from the bottom to be the second last shelf.
- A single serving is now considered to be approximately 150kcal (plus or minus 25kcal) This now equates to 2 thin slices of regular pan bread at about 150 calories (FSAI, 2012). The range of servings from this Shelf is 3 to 5 servings/day – with up to 7 for teenage boys and men aged 19–50.
- Limiting processed salty meats like sausages, bacon and ham to not every day, in line with the FSAI 2015 recommendations (Healthy Ireland, 2016b)

LEAD4 - There is a comprehensive, transparent, up-to-date implementation plan linked to national needs and priorities, to improve food environments, reduce the intake of the nutrients of concern to meet WHO and national recommended dietary intake levels, and reduce diet-related NCDs

Healthy Weight for Ireland: Obesity Policy and Action Plan 2016-2025

- A Healthy Weight for Ireland', Obesity Policy and Action Plan 2016-2025 (OPAP), was launched in September 2016 under the auspices of the Healthy Ireland agenda. The OPAP covers a ten-year period up to 2025 and aims to reverse obesity trends, prevent health complications and reduce the overall burden for individuals, families, the health system, and the wider society and economy (Department of Health, 2016b).

- The OPAP policy prescribed 'Ten Steps Forward' that would be taken to prevent overweight and obesity:
    1. Embed multi-sectoral actions on obesity prevention with the support of government departments and public sector agencies
    2. Regulate for a healthier environment
    3. Secure appropriate support from the commercial sector to play its part in obesity prevention
    4. Implement a strategic and sustained communications strategy that empowers individuals, communities and service providers to become

obesity aware and equipped to change, with a particular focus on families with children in the early years.

5. The Department of Health, through Healthy Ireland, will provide leadership, engage and co-ordinate multi-sectoral action and implement best practice in the governance of the Obesity Policy and Action Plan.
6. Mobilise the health services to better prevent and address overweight and obesity through effective community-based health promotion programmes, training and skills development and through enhanced systems for detection and referrals of overweight and obese patients at primary care level.
7. Develop a service model for specialist care for children and adults
8. Acknowledge the key role of physical activity in the prevention of overweight and obesity
9. Allocate resources according to need, in particular to those population groups most in need of support in the prevention and management of obesity, with particular emphasis on families and children during the first 1,000 days of life
10. Develop a multi-annual research programme that is closely allied to policy actions, invest in surveillance and evaluate progress on an annual basis (Department of Health, 2016b)

Obesity Policy Implementation Oversight Group (OPIOG)

- A new Obesity Policy Implementation Oversight Group (OPIOG) was established in October 2017 and a progress report on each recommendation in the OPAP is currently being finalised under the aegis of the OPIOG.

LEAD5 - Government priorities have been established to reduce inequalities or protect vulnerable populations in relation to diet, nutrition, obesity and NCDs

Healthy Weight for Ireland: Obesity Policy and Action Plan 2016-2025

- **Step 9:** of the obesity policy and action plan aims to, allocate resources according to need, in particular to those population groups most in need of support in the prevention and management of obesity, with particular emphasis on families and children during the first 1,000 days of life. The priority actions to commence in first year were to, assess the needs of vulnerable groups as the basis of allocation of resources for preventative and treatment services for children and adults (Department of Health, 2016c).

- The Healthy Ireland fund was established in 2017 with an allocation of €5 million and with additional allocations of €5 million in 2018 and 2019. The first round of the Fund was distributed through Local Community Development Committees, Children and Young Person's Services Committees and statutory organisations. The aim of the Fund is to support innovative, cross sectoral, evidence-based projects, programmes and initiatives that support the implementation of key national policies in areas such as Obesity, Smoking Alcohol, Physical Activity and Sexual Health (Oireachtas, 2019).

- The Fund has been effective at targeting population groups that experience health inequalities. In Round 1 (2017/18), there was a focus on specific groups experiencing health inequalities, including people living in areas of social disadvantage (71% of actions), people with disabilities (45%), people from new communities including refugees and asylum seekers (39%) and members of the Traveller community (36%). Furthermore, of the local actions that were implemented in Round 1, 61% related to physical activity and 32% were related to food, nutrition and weight management. Round 2 of the Fund is currently being implemented (Oireachtas, 2019)

Healthy Weight for Ireland: Obesity Policy and Action Plan 2016-2025

- **Step 4:** The action plan also aims to implement a strategic and sustained communications strategy that empowers individuals, communities and service providers to become obesity aware and equipped to change, with a particular focus on families with children in the early years. The priority actions to commence in first year were to, develop a communications strategy to increase knowledge among the public and all other relevant stakeholders of the benefits of being a healthy weight and to stimulate healthy behaviour change. This included measures to communicate to disadvantaged and low income groups, including considering how best to link to measures already being taken by the Department of Social Protection (Department of Health, 2016b)

- The Healthy Ireland 2018 communications and citizen engagement campaign sought to encourage people to make small, healthy changes under the themes of Healthy Eating, Physical Activity and Mental Wellbeing, and to link them with partner organisations and initiatives providing information and support (Oireachtas, 2019). This campaign has continued and was launched on the 8th of April 2019, with an announced funding of €1 million to boost community engagement on health and wellbeing in every county. The 2019 campaign seeks to raise public awareness of the gov.ie/HealthyIreland website, as the place to go that brings trusted sources of information and supports together in one place to help people get started on making healthy changes (Department of Health, 2019d)

Health Service Executive: The Healthy Eating Active Living Policy Priority Programme - National Implementation Plan 2017-2020

- A Healthy Eating, Active Living Programme has been established as a Policy Priority Programme within the HSE, to support the Healthy Ireland in Health Services Implementation Plan (Health Service Executive, 2015). A three-year plan for the programme has been finalised. It is supporting work in the education sector, as well as with parents, families and communities in delivering a more coordinated approach to prevention and early intervention in child obesity. The GP contract for the provision of free care to children under 6 years, already provides that the medical practitioner shall take an active approach toward promoting health and preventing disease through the provision of periodic assessments to child patients (health Service Executive, 2017b).

Joint Initiative on Food Poverty

- A Joint Initiative on Food Poverty relating to children and families, between Healthy Ireland, the Better Outcomes, Brighter Futures Advisory Council and the Department of Children and Youth Affairs, was launched in 2019 to report with a programme of actions in 2020 (Department for Children and Youth Affairs, 2014-2020)

DOMAIN 9 - GOVERNANCE: Governments have structures in place to ensure transparency and accountability, and encourage broad community participation and inclusion when formulating and implementing policies and actions to create healthy food environments, improve population nutrition, and reduce diet-related inequalities.

GOVER1 - There are procedures in place to restrict commercial influences on the development of policies related to food environments where they have conflicts of interest with improving population nutrition. for example: restricting lobbying influences.

The Code of Conduct for persons carrying on lobbying activities

- In support of the Regulation of Lobbying Act 2015 Act's objectives, to foster transparency and the proper conduct of lobbying activities, this Code of Conduct sets out several principles by which persons carrying on lobbying activities should govern themselves in the course of carrying out lobbying activities, namely:
    1. Demonstrating respect for public bodies
    2. Acting with honesty and integrity
    3. Ensuring accuracy of information
    4. Disclosure of identity and purpose of lobbying activities
    5. Preserving confidentiality
    6. Avoiding improper influence
    7. Observing the provisions of the Regulation of Lobbying Act
    8. Having regard to the Code of Conduct.

- The purpose of this Code is to govern the behaviour of persons carrying on lobbying activities. The provisions of the Act can apply to employers; to representative or advocacy bodies; to professional lobbyists or third parties who are being paid to communicate on behalf of a client or other person; and, significantly, to any person communicating about the development or zoning of land (Standards in Public Office Commission, 2019).

GOVER2 - Policies and procedures are implemented for using evidence in the development of food and nutrition policies

The Food Safety Authority of Ireland (FSAI)

- The Food Safety Authority of Ireland (FSAI) is legally obliged to base its opinions on scientific grounds and to develop food standards on the basis of the best, most up-to-date scientific advice available. They also link with Healthy Ireland providing scientific recommendations on priority issues as required (Food Safety Authority of Ireland, 2018)

- To aid the FSAI in risk assessment, which underpins risk management decisions, the current Scientific Committee was set up in 2016 in accordance with Article 34 of the Food Safety Authority of Ireland Act, 1998. The Committee is made up of scientists, from a variety of disciplines, working in a voluntary capacity. It has a major influence on policy decisions of the FSAI, including advice on the implementation and

administration of food inspection services and on the nutritional value of food. It also provides clarity on scientific and technical issues relating to food safety and hygiene (Food Safety Authority of Ireland, 2018).

A Healthy Weight for Ireland: Obesity Policy and Action Plan 2016-2025

- Action 1.8 under Step 1 of the OPAP, *"Embed multi-sectoral actions on obesity prevention with the support of government departments and public sector agencies".* The Department of Health aims to develop proposals on the rollout of evidence-based fiscal measures to support healthy eating and lifestyles (Department of Health., 2016)

GOVER3 - Policies and procedures are implemented for ensuring transparency in the development of food and nutrition policies

Transparency code

- The Transparency Code was prepared in accordance with the Regulation of Lobbying Act 2015, which commenced on September 1st, 2015 (Department of Public Expenditure and Reform, 2015). The Transparency Code sets out how certain public bodies may conduct their activities in a transparent way. The Regulation of Lobbying Act 2015 aims to make the process of developing policies more transparent by providing for:

- The establishment and maintenance of a publicly accessible register of lobbying; Obligations on lobbyists to register and to provide information regularly about their lobbying activities, including, in the case of professional lobbyists, information about their clients.
- The introduction of a "cooling off" period during which lobbying activity may not be carried out by some former officials.
- The Standards in Public Office Commission (The Standards Commission) to be the regulator of lobbying.
- As part of the Open Government Partnership National Action Plan 2014-2016, Ireland has committed to 'greater citizen consultation and involvement to strengthen democracy and improve public services.

No further evidence was found for 2016 onwards

GOVER4 - The government ensures public access to comprehensive nutrition information and key documents (e.g. budget documents, annual performance reviews and health indicators) for the public

The Reform of the Freedom of Information legislation

- The Reform of the Freedom of Information legislation was announced by the Minister for Public Expenditure and Reform in 2012. The new Freedom of Information Act came

into force in 2014, applying to all public bodies (Department of Public Expenditure and Reform, 2019). It provides the following statutory rights: 1. A legal right for each person to obtain reasons for decisions affecting himself/herself. 2. A legal right for each person to access information held by a body to which Freedom of Information Legislation applies.

A Freedom of Information body is required to:

1. Publish information to assist members of the public in their understanding of body and its functions.
2. Publish the information that it holds grouped under the information headings set out in the model publication scheme.
3. Explain the procedures to get access to information or to establish what information the body hold.

- Up-to-date information on a range of indicators including nutrition and diet-related indicators is made available to the public via the government websites: Various sources of data are available including survey data, annual reports and budget information https://health.gov.ie/.

DOMAIN 10 - MONITORING AND INTELLIGENCE: The government's monitoring and intelligence systems (surveillance, evaluation, research and reporting) are comprehensive and regular enough to assess the status of food environments, population nutrition and diet-related NCDs and their inequalities, and to measure progress on achieving the goals of nutrition and health plans.

MONIT1 - Monitoring systems, implemented by the government, are in place to regularly monitor food environments (especially for food composition for nutrients of concern, food promotion to children, and nutritional quality of food in schools and other public sector settings), against codes / guidelines / standards / targets

Food Safety Authority of Ireland (FSAI)

- As part of their responsibility for verifying that the relevant requirements of food law are fulfilled by food business operators at all stages of production, processing and distribution, the FSAI and its agencies carry out monitoring and surveillance of food in the Irish market. These activities are undertaken in conjunction with the official agencies such as, the Health Service Executive, the Department of Agriculture, Food and the Marine, Local Authorities and the Sea Fisheries Protection Authority and their associated laboratories (Food Safety Authority of Ireland, 2014)

FSAI (2010) - Monitoring of Food Labelling

- Monitoring of food labelling in Ireland is carried out to assess compliance with the labelling legislation and to check for authenticity of stated ingredients.
- In 2010, the FSAI carried out a survey to examine the accuracy of nutrition labelling on pre-packaged foods on the Irish market and to provide these data to the European Commission to inform their work in revision of nutrition labelling legislation. A secondary objective was to generate sufficient data on which to base national guidelines should a legislative route not be agreed at European level (Food Safety Authority of Ireland, 2010b)

FSAI (2016) - Survey of the trans fatty acid content in processed food products in Ireland

- During June 2016 the FSAI collected 240 branded and private label processed food products across all major supermarkets in Ireland. The samples comprised a cross-section of products grouped into 15 product categories. The latest FSAI survey indicates that overall levels of TFA in processed food products in Ireland are low and continue to decrease, with 97.5% of products having ≤2% TFA as a percentage of total fat. The results of this survey also verify the effectiveness of industry commitments and initiatives to reduce or exclude TFA from processed food products (Food Safety Authority of Ireland, 2016a).

Nutrition Standards for School Meals

- The standards were published under the auspices of 'Healthy Ireland', and will be implemented and monitored through the Department of Employment Affairs and Social Protection (Healthy Ireland, 2017a).

- Schools and organisations must reapply for funding in advance of each school year and are required to submit detailed records at the end of each school year. Expenditure on unhealthy food items is deducted from the following year's funding allocation. The requirement to implement these nutrition standards is therefore largely met by that Department only funding healthy foods provided by schools under the school meals programme. In addition, the Department initiated an on-site inspection programme in 2012 in participating schools to ensure that the scheme rules are being adhered to. The inspection process is being rolled out across all schools and includes an assessment of the type of food provided. All schools participating in the School Meals Scheme are expected to comply with the standards from September 2018. Compliance with the new standards will form part of the inspection process from the 2018/2019 academic year (Department of Employment Affairs and Social Protection, 2018).

MONIT2 - There is regular monitoring of adult and childhood nutrition status and population intakes against specified intake targets or recommended daily intake levels

The National Nutritional Surveillance Centre (NNSC)

- The National Nutritional Surveillance Centre (NNSC) is required by the Health Service Executive to provide an independent source of information and advice and has the responsibility for collection and collation of available data relating to nutrition and health. Food and/or nutrition surveillance is based on the use of routinely collected or easily generated data from several sectors related to nutrition and health. These include: Food supply/ production/retail, Food consumption data, Nutritional knowledge, attitudes and beliefs, Information on effective nutrition programmes,

Socio-economic indicators related to health, Nutrition related health status indicators, Food economics (National Nutrition Surveillance Centre).

- The aims of the NNSC include: To monitor trends in health status correlated with all aspects of the food chain and advice on these findings for health planners, to provide a source of information and research expertise, particularly in nutritional epidemiology and surveillance methodology to those wishing to mount specific projects such as micro-surveys. The reports produced by the NNSC have been widely disseminated among Health Board personnel, food agencies, producers, retailers, caterers, academics, teachers and the media (National Nutrition Surveillance Centre).

Healthy Ireland Survey

- Healthy Ireland survey is an annual interview administered face-to-face survey commissioned by the Department of Health, with a representative sample of the population aged 15 and older living in Ireland. It gathers information on the proportion of the population who drink sugar sweetened drinks daily, the proportion of the population who eat at least 5 portions of fruit and vegetables daily, the weight of people in the population, and also information on general health and utilisation of health services. Survey data play a number of roles, including supporting the Department in on-going engagement and awareness-raising activities in the various policy areas and supporting better understanding of policy priorities (Healthy Ireland & Department of Health, 2019).

The health behaviour in School – Aged Children

- Health Behaviour in School-aged Children (HBSC) is a cross-national research study conducted in collaboration with the World Health Organization (WHO) Regional Office for Europe. The study aims to gain new insight into, and increase our understanding of young people's health and well-being, health behaviours and their social context. In addition, the findings from the HBSC surveys are used to inform and influence children's policy and practice at national and international levels (National University of Ireland). In Ireland, the study is conducted by researchers in the Health Promotion Research Centre at the National University of Ireland, Galway. The network collects data every 4 years on 11-, 13-, and 15- year- old boy's and girl's health and well-being, social environments and health behaviours.

MONIT3 - There is regular monitoring of adult and childhood overweight and obesity prevalence using anthropometric measurements

Healthy Ireland Survey

- The Healthy Ireland Survey is an annual interviewer-administered face-to-face survey commissioned by the Department of Health. It is part of the Healthy Ireland Framework to improve the health and wellbeing of people living in Ireland. Survey

data play a number of roles, including supporting the Department in ongoing engagement and awareness-raising activities in the various policy areas and supporting better understanding of policy priorities. The most recent survey contained a representative sample of 7,701 people aged 15 and older living in Ireland were interviewed between September 2017 and June 2018. As part of the survey, overweight and obesity level in the country are measured every 2 years (Healthy Ireland & Department of Health, 2019).

Childhood Obesity Surveillance Initiative report

- The COSI survey funded by the Health Service Executive (HSE), is a World Health Organization (WHO) led project that is part of a pan-European initiative. It is an ongoing, systematic process of collection, analysis, interpretation and dissemination of descriptive information for monitoring obesity, being identified as a serious public health problem in the WHO European Region and for use in programme planning and evaluation (National nutrition Surveillance Centre).
- The 4th Childhood Obesity Surveillance Initiative report was launched in 2017. COSI collects data from children in primary schools in the Republic of Ireland and is carried out periodically. Data was first collected from children in 2008 in first class and again in 2010 from first class and third class, in 2012 from first, third and fifth classes and in 2015 from first, fourth and sixth class. Trained researchers collected weight, height and waist circumference measurements. These figures were used to examine prevalence of normal weight, overweight, obesity and mean BMI (National nutrition Surveillance Centre).

Growing up in Ireland: National Longitudinal Study of Children

- The Growing Up in Ireland study started in 2006 as the national longitudinal study of children in Ireland. It is funded by the Government of Ireland through the Department of Children and Youth Affairs (DCYA) with a contribution (in Phase 2) from The Atlantic Philanthropies. It is overseen and managed by the DCYA in conjunction with the Central Statistics Office and an inter-departmental Project Team and Steering Group. It is implemented by a consortium of researchers led by the Economic and Social Research Institute (ESRI) and Trinity College Dublin (Department of Children and Youth Affairs, 2018b).
- The growing up in Ireland study, the lives of 13-year-olds (2018), presented a broad, comprehensive overview of the lives of the Child Cohort at age 13 and to describe how they are faring in important areas of their lives. This included a section on weight status and activities (Department of Children and Youth Affairs, 2018b).

A Healthy Weight for Ireland: Obesity Policy and Action Plan 2016 – 2025

- Action 6.1 of the Obesity Policy and Action Plan provides for the inclusion of obesity prevention and care as part of the General Practitioner Contract. Under the GP contract for the provision of care to children under 6 years of age, medical practitioners are required to take an active approach toward promoting health and preventing disease through the provision of periodic assessments to child patients. Assessments involve the recording of age, gender, weight and height, and plotting on a centile chart at ages two and five, and taking appropriate follow-up action, including where appropriate, provision of health promotion advice, brief intervention and support, or referral to specialist services.(Department of Health, 2016b)

MONIT4 - There is regular monitoring of the prevalence of NCD metabolic risk factors and occurrence rates (e.g. prevalence, incidence, mortality) for the main diet-related NCDs

- According to the WHO Ireland doesn't have an NCD surveillance and monitoring system in place to enable reporting against the nine global NCD targets (World Health Organisation, 2014)

MONIT5 - Major programs and policies are regularly evaluated to assess their effectiveness and contributions to achieving the goals of the nutrition and health plans

Food Safety Authority of Ireland (FSAI)

- **Labelling:** monitoring of food labelling in Ireland is carried out to assess compliance with the labelling legislation and to check for authenticity of stated ingredients. For e.g. The objective of this survey was to examine the accuracy of nutrition labelling on pre-packaged foods on the Irish market and to provide these data to the European Commission to inform their work in revision of nutrition labelling legislation. A secondary objective was to generate sufficient data on which to base national guidelines should a legislative route not be agreed at European level (Food Safety Authority of Ireland, 2010a).
- **Nutrition:** the FSAI produce reports on surveys of the trans fatty acid content in processed food products in Ireland.

Government monitoring of voluntary codes of practice

- Regarding the issued set of voluntary codes of practice aimed at limiting the promotion, marketing and sponsorship of foods high in fats, sugar and/or salt (HFSS foods). The government of Ireland has committed to monitor compliance, and work is continuing on the development of a monitoring mechanism.

MONIT6 - Progress towards reducing health inequalities or health impacts in vulnerable populations and social and economic determinants of health are regularly monitored.

Healthy Ireland Survey

- The Healthy Ireland Survey 2018, for the first time measured and addressed health inequalities in Ireland and will continue to monitor inequalities annually. Results found that, those in more deprived areas are less likely to rate their health as good or very good, and more likely to have a long-term health problem. Similarly, those in more deprived areas are more likely to smoke and binge drink. Self-rated good health declines earlier for men in deprived areas (around the age of 55 to 64) than it does for both men in affluent areas and women in deprived areas. Lastly, women living in more deprived areas are more likely than those in affluent areas to continue smoking and binge drinking into their 50s (Healthy Ireland, 2018).

Healthy Ireland Fund

- In 2016, the Government approved the creation of a Healthy Ireland Fund, with an allocation of €5 million approved in Budget 2017 and 2018 to establish and support the implementation of Healthy Ireland programmes and projects in a variety of settings. The new round will run from 01 April 2018 to 31 March 2019. The funding goes towards a number of local projects in Ireland for socially disadvantaged groups including travellers and homeless families. The new structures established under Healthy Ireland to enable and support its implementation will provide a focus on addressing the health needs of all groups experiencing health inequalities, including travellers. The All-Ireland Traveller Health Study (AITHS) was a large-scale study focusing on key aspects of traveller health, social status and service utilisation (Government of Ireland, 2016).

DOMAIN 11 - FUNDING AND RESOURCES: Sufficient funding is invested in 'Population Nutrition Promotion' (estimated from the investments in population promotion of healthy eating and healthy food environments for the prevention of obesity and diet-related NCDs, excluding all one-on-one promotion (primary-care, antenatal services, maternal and child nursing services etc.), food safety, micronutrient deficiencies (e.g. folate fortification and undernutrition) to create healthy food environments, improved population nutrition, reductions in obesity, diet-related NCDs and their related inequalities.

FUND1 – The 'population nutrition' budget, as a proportion of total health spending and/or in relation to the diet-related NCD burden sufficiently contributes to reducing diet-related NCD's.

(Not being included in the online rating survey)

Government Expenditure Report 2018

- In 2018, the funding level available to health reached €16.2 billion. The 2018 allocation marked a record as the highest in the history of the State. From 2016 onwards the Budget provided increased the annual allocation and on average provided an additional €431m annually to health. Despite this increase, the health sector continued to overspend and the supplementary funding provided ranged from €195m to around €645m(Government of Ireland, 2018).

FUND2 - Government funded research is targeted for improving food environments, reducing obesity, NCDs and their related inequalities

Healthy Ireland, A Framework for Improved Health and Wellbeing 2013-2025

- Healthy Ireland, A Framework for Improved Health and Wellbeing 2013-2025 is the national framework for action to improve the health and wellbeing of Ireland over the coming generation. Pobal administer this fund on behalf of the Department of Health (Healthy Ireland, 2019).

- In 2016, the Government approved the creation of a Healthy Ireland Fund with an initial allocation of €5 million approved in Budget 2017 to establish and support the implementation of Healthy Ireland programmes and projects in a variety of settings. The primary aim of the fund is to support innovative, cross-sectoral, evidence-based projects and initiatives that support the implementation of key national policies in areas such as obesity, smoking, alcohol, physical activity and sexual health. The funding has been allocated to 2 Strands, both of which are closed calls for funding. There will be a new round of funding in 2018. The new round will run from 01 April 2018 to 31 March 2019. Strand 1 focuses on the delivery of local initiatives and Strand 2 funds national actions (Healthy Ireland, 2019).

FUND3 - There is a statutory health promotion agency in place that includes an objective to improve population nutrition with a secure funding stream

Healthy Ireland

- Healthy Ireland is a Government-led initiative which aims to create an Irish society where everyone can enjoy physical and mental health, and where wellbeing is valued and supported at every level of society.

- Resources such as the HSE are currently working on the ground on ‘A Healthy weight for Ireland Obesity Policy and Action Plan 2016-2025’. The Department of Health will be the main source of management in relation to this action plan, while working closely with a number of other key stakeholders

HSE – Health and Wellbeing division

The Health and Wellbeing Division of the HSE is focused on helping people to stay healthy and well, reducing health inequalities and protecting people from threats to their health and wellbeing. It was established based on two fundamental policy shifts within the health service – Future Health, which describes the new structures currently being established for the healthcare system, and Healthy Ireland, the Government Framework to improve the health and wellbeing of our population. The services within Health and Wellbeing support people and communities to protect and improve their health and wellbeing; turning research, evidence and knowledge into action; acting as the authority on health, wellbeing and policy development; building an intelligent health system and a healthier population.

DOMAIN 12 - PLATFORMS FOR INTERACTION: There are coordination platforms and opportunities for synergies across government departments, levels of government, and other sectors (NGOs, private sector, and academia) such that policies and actions in food and nutrition are coherent, efficient and effective in improving food environments, population nutrition, diet-related NCDs and their related inequalities.

PLAT1 - There are robust coordination mechanisms across departments and levels of government (national, state and local) to ensure policy coherence, alignment, and integration of food, obesity and diet-related NCD prevention policies across governments.

START campaign

- As one of the actions of the ‘A Healthy Weight for Ireland, Obesity Policy and Action Plan 2016 – 2025’ strategy, the Department of Health, Healthy Ireland, Health Service

Executive and safefood have been engaged in developing a new 5-year public awareness campaign to address maintaining a healthy weight in children (Healthy Ireland, 2017c). The Start campaign was developed using a co-creation approach, which involved working with parents in particular, and key stakeholders to ensure the campaign was relevant, realistic and could achieve results. It seeks to help families take that first step and then to continue ongoing steps towards a healthier lifestyle for their children, with the target audience being parents and carers of children aged between 6 months and 12 years of age.

- The Campaign messages are to; Minimise intake of foods high in fat, salt and sugar, establish water and milk as routine drinks, Advocate appropriate child-sized portion sizes, increase healthier food choices – more fruit, vegetables and salad, Increase physical activity levels, limit screen-time and Increase sleep-time Communication channels. The first phase of the campaign advertising will feature on TV, radio, video on demand, outdoor and digital platforms. The campaign will be supported by media relations and social media. Timings 30th October – 25th November 2017 (Healthy Ireland, 2017c).

Department of Health

- 'The Department of Health, through Healthy Ireland, will provide leadership, engage and co-ordinate multi-sectorial action and implement best practice in the governance of the 'Obesity Policy Action Plan 2016-2025'. Leadership and coordinated action will be provided by the department of health. The department of health and safefood are taking action to establish a multi stakeholder partnership to share knowledge and initiative on healthy weight initiatives (Department of Health, 2016b).

FSAI and safefood

- There is a memorandum of understanding between the FSAI and other agencies, for example, the Food Safety Authority of Ireland and Safefood. This looks at a framework for cooperation in activities to do with food safety. The FSAI and Safefood agree on certain actions such as:
  - To provide each other with full and timely access to research, findings, and data as well as other relevant information.
  - To regularly inform each other about work that interests both organisations.
  - To take opportunities that involve joint planning and to fully cooperate when needed (Food Safety Authority of Ireland & safefood, 2016)

PLAT2 - There are formal platforms (with clearly defined mandates, roles and structures) for regular interactions between government and the commercial food sector on the implementation of healthy food policies and other related strategies

The Obesity Policy and Action Plan 2016-2025

- Step 3, priority action 2 in the 'Obesity Policy and Action Plan 2016-2025', the Department of Health developed, implemented a code of practice for food and beverages promotion, marketing and sponsorship. Engagement between partners involved (food industry, HSE, DCYA, safefood, FSAI, advertiser organisations) was successful and will be continued on a regular basis.

- There are also commercial bodies that regularly communicate with different sections of the government. Food Drink Ireland (FDI) is the main trade association for the food and drink industry in Ireland. It represents the interests of over 150 foods, drink and non-food grocery manufacturers and suppliers. FDI is committed to ensuring an environment exists which is conducive to the success and further growth of the food and drink industry in Ireland. FDI provides leadership and direction on a number of key strategic issues, including the economic importance of the sector, competitiveness, research & development, trade, skills and education.

PLAT3 - There are formal platforms (with clearly defined mandates, roles and structures) for regular interactions between government and civil society on the development, implementation and evaluation of healthy food policies and other related strategies.

- **No further evidence found from 2016 onwards**

PLAT4 - The governments work with a system-based approach with (local and national) organisations/partners/groups to improve the healthiness of food environments at a national level.

Healthy Ireland framework 2013-2025

- The Healthy Ireland Framework draws on existing policies but proposes new arrangements to ensure effective co-operation and collaboration and to implement evidence-based policies at government, sectoral, community and local levels. It proposes a necessary shift towards a broader, more inclusive approach to governance for health, moving beyond the health service, across national and local authorities, involving all sectors of society, and the people themselves. The framework aims to

draw up specific proposals in relation to the potential role of local authorities in the area of health and wellbeing. Furthermore, Local health partners will engage with local authorities in their work to address local and community development, with the aim of coordinating actions and improving information-sharing for improved health and wellbeing (Healthy Ireland, 2013-2025).

DOMAIN 13 - HEALTH IN ALL POLICIES: Processes are in place to ensure policy coherence and alignment, and that population health impacts are explicitly considered in the development of government policies.

HIAP1 - There are processes in place to ensure that population nutrition, health outcomes and reducing health inequalities or health impacts in vulnerable populations are considered and prioritised in the development of all government policies relating to food.

Healthy Ireland framework 2013-2025

- The Republic of Ireland National Framework to improve health and wellbeing 'Healthy Ireland' acknowledges that intersectoral working or Health in All Policies is a politically challenging strategy that requires deliberate efforts to be promoted. Healthy Ireland

seeks to address these challenges through its vision, goals, structures and actions. In Ireland, the Government has committed to incorporating poverty impact assessment as part of an integrated social impact assessment. Health impacts will be a core feature of this new tool. The Outcomes Framework aims to provide a structured approach to collect and report relevant and appropriate data which can be used to build awareness of these social determinants of health, to support assessment of the impact of policies on the agreed outcomes, and to monitor progress on the whole of government response needed to improve health and wellbeing (Healthy Ireland, 2013-2025).

HIAP2 - There are processes e.g. Health Impact Assessment's (HIAs) to assess and consider health impacts during the development of other non-food policies.

- **No further evidence found post 2016**

Bibliography

AGRI AWARE'S INCREDIBLE EDIBLES. *https://www.incredibleedibles.ie/the-incredible-edibles-project.html* [Online]. [Accessed].

BROADCASTING AUTHORITY OF IRELAND 2017. General commercial Communications Code

BROADCASTING AUTHORITY OF IRELAND. 2019. *Codes and Standards* [Online]. Available: https://www.bai.ie/en/codes-standards/#al-block-5 [Accessed].

DEPARTMENT FOR CHILDREN AND YOUTH AFFAIRS 2014-2020. Better Outcomes Brighter Futures. The national policy framework for children and yound people, 2014-2020.

DEPARTMENT OF AGRICULTURE FOOD AND MARINE 2017. Creed Announces Re-Opening of Beef Data and Genomics Programme.

DEPARTMENT OF AGRICULTURE FOOD AND MARINE 2018. Terms and conditons of the scheme of EU AID for producer organisations in the fruit and vegetable sector

DEPARTMENT OF AGRICULTURE FOOD AND MARINE 2019. 2019 scheme of investment aid for the development of the commercial horticulture sector.

DEPARTMENT OF AGRICULTURE FOOD AND MARINE AND AN BORD BIA 2009. Voluntary Good Practice Standard for Farmers' Markets 2012-2013.

DEPARTMENT OF CHILDREN AND YOUTH AFFAIRS 2018a. First 5: A Whole-of-Government Strategy for Babies, Young Children and their Families.

DEPARTMENT OF CHILDREN AND YOUTH AFFAIRS 2018b. Growing Up in Ireland National Longitudinal Study of Children. The Lives of 13-Year-Olds.

DEPARTMENT OF EMPLOYMENT AFFAIRS AND SOCIAL PROTECTION 2018. Written submission by the Department of Employment Affairs and Social Protection regarding the School Meals Programme to the Joint Committee on Children and Youth Affairs on the topic 'Tackling Childhood Obesity'. [online] Accessed on https://data.oireachtas.ie/ie/oireachtas/committee/dail/32/joint_committee_on_children_and_youth_affairs/submissions/2018/2018-08-22_submission-department-of-employment-affairs-and-social-protection_en.pdf.

DEPARTMENT OF EMPLOYMENT AFFAIRS AND SOCIAL PROTECTION 2019. First Serving of Hot School Meals Project Commences in Inchicore. https://www.welfare.ie/en/pressoffice/Pages/prb280119.aspx.

DEPARTMENT OF HEALTH 2013. Healthy Ireland - a framework for improved health and wellbeing 2013 - 2025. Dublin: Department of Health.

DEPARTMENT OF HEALTH 2016a. Get Ireland Active! The National Physical Activity Plan for Ireland.

DEPARTMENT OF HEALTH 2016b. A Healthy Weight for Ireland. Obesity Policy and Action Plan: 2016-2025.

DEPARTMENT OF HEALTH 2016c. A Healthy Weight for Ireland: Obesity Policy and Action Plan 2016 – 2025.

DEPARTMENT OF HEALTH 2017. Non-Broadcast Media Advertising and Marketing of Food and Non-Alcoholic Beverages, including Sponsorship and Retail Product Placement:Voluntary Codes of Practice.

DEPARTMENT OF HEALTH. 2019a. *Healthy Ireland Food Plans and Guides* [Online]. Available: https://www.gov.ie/en/publication/b01555-healthy-ireland-food-plans-and-guides/ [Accessed].

DEPARTMENT OF HEALTH 2019b. Healthy Ireland Fund 2019- 2021. Expression of Interest Guidelines. [online] Accessed on https://drive.google.com/file/d/1rqVbr_YILdi12GqeGNgPnILPaEbrCBKB/view.

DEPARTMENT OF HEALTH. 2019c. *National Healthy Cities and Counties of Ireland Network. [online] Accessed on https://www.gov.ie/en/publication/d4fa22-healthy-counties-and-cities/#national-healthy-cities-and-counties-of-ireland-network* [Online]. [Accessed].

DEPARTMENT OF HEALTH 2019d. Taoiseach and Ministers launch Healthy Ireland campaign. [online] Accessed on https://www.gov.ie/en/news/20cff4-taoiseach-and-health-ministers-to-launch-2019-healthy-ireland-campai/.

DEPARTMENT OF HEALTH. 2016. A Healthy Weight for Ireland. Obesity Policy and Action Plan: 2016–2025. Dublin.

DEPARTMENT OF PUBLIC EXPENDITURE AND REFORM. 2015. *Transparency Code Prepared in Accordance with Section 5(7) of the Regulation of Lobbying Act 2015. [online] Accessed on https://www.lobbying.ie/media/5986/2015-08-06-transparency-code-eng.pdf* [Online]. [Accessed].

DEPARTMENT OF PUBLIC EXPENDITURE AND REFORM 2019. FOI Publication Scheme. [online] Accessed on https://www.gov.ie/en/organisation-information/60131b-foi-publication-scheme/.

FOOD AND DRINK IRELAND 2019. The evolution of food and drink in Ireland 2005-2017. Reformulation and innovation: supporting Irish diets. .

FOOD DRINK IRELAND 2016 The FDII/ Creme Global Reformulation Project. Estimating the impact of reformulation by 14 FDII members on the Irish population

FOOD SAFETY AUTHORITY OF IRELAND 2010a. Accuracy of Nutrition Labelling of Pre-Packaged Food in Ireland. Monitoring & Surveillance Series.

FOOD SAFETY AUTHORITY OF IRELAND 2010b. Monitoring and Surveillance Series. Accuracy of Nutrition Labelling of Pre-Packaged Food in Ireland. July 2010.

FOOD SAFETY AUTHORITY OF IRELAND 2011. Scientific Recommendations for Healthy Eating Guidelines in Ireland [Online]. Dublin: Food Safety Authority of Ireland.

FOOD SAFETY AUTHORITY OF IRELAND. 2014. *Labelling Monitoring and Surveillance. [online] Accessed on https://www.fsai.ie/enforcement_audit/monitoring/food_monitoring_surveillance/labelling.html* [Online]. [Accessed].

FOOD SAFETY AUTHORITY OF IRELAND 2016a. Monitoring and Surveillance Series. Survey of the trans fatty acid content in processed food products in Ireland. .

FOOD SAFETY AUTHORITY OF IRELAND 2016b. Salt reducing in processed foods - Salt in diet still high. .

FOOD SAFETY AUTHORITY OF IRELAND 2018. Scientific Committee. [online] Accessed on https://www.fsai.ie/about_us/scientific_committee.html.

FOOD SAFETY AUTHORITY OF IRELAND 2019. About MenuCal. https://www.fsai.ie/food_businesses/menucal.html [online]

FOOD SAFETY AUTHORITY OF IRELAND & SAFEFOOD 2016. Memorandum of understanding between the Food Safety Authority of Ireland and safefood - the Food Safety Promotion Board

GOVERNMENT OF IRELAND 2016. Healthy Ireland Fund

GOVERNMENT OF IRELAND 2018. Health Budget Oversight & Management: Alignment of Health Budget and National Service Plan. .

HEALTH SERVICE EXECUTIVE 2015. Healthy Ireland in the Health Service Implementation Plan.

HEALTH SERVICE EXECUTIVE. 2016. *The Nurture Programme - Infant Health & Wellbeing* [Online]. [Accessed].

HEALTH SERVICE EXECUTIVE 2017a. Eat Smart A Guide for Healthy Eating and Getting Active for Children. .

HEALTH SERVICE EXECUTIVE 2017b. Healthy Eating and Active Living Programme | National Implementation Plan 2017-2020.

HEALTH SERVICE EXECUTIVE 2018. Food, Nutrition and Hydration Policy for adult patietns in acute hospitals

HEALTH SERVICE EXECUTIVE 2019. HSE Vending Policy

HEALTH SERVICES EXECUTIVE. 2018. *Brief updates from The Nurture Programme. https://www.hse.ie/eng/health/child/nurture/updates.html* [Online]. [Accessed].

HEALTHY IRELAND 2013-2025. A framework for improved health and wellbeing 2013-2025.

HEALTHY IRELAND 2016a. Healthy Food for Life. Revised healthy eating guidelines and Food Pyramid rationale. .

HEALTHY IRELAND 2016b. Revised healthy eating guidelines and Food Pyramid rationale.

HEALTHY IRELAND 2017a. Nutrition Standards for School Meals

HEALTHY IRELAND 2018. Healthy Ireland Survey 2018. [online] available: https://www.hse.ie/eng/about/who/healthwellbeing/healthy-ireland/publications/healthy-ireland-survey-2018.pdf.

HEALTHY IRELAND 2019. Healthy Ireland Fund. [online] available: https://www.pobal.ie/programmes/healthy-ireland-fund/.

HEALTHY IRELAND 2017b. Non- Broadcast Media Advertising and Marketing of food and Non-Alcoholic Beverages, including Sponsorship and Retail Product Placement: Voluntary Codes of Practice. .

HEALTHY IRELAND & DEPARTMENT OF HEALTH 2019. Healthy Ireland Survey documents. [online] available: https://www.gov.ie/en/collection/231c02-healthy-ireland-survey-wave/.

HEALTHY IRELAND, S. A. H. 2017c. Start campaign

MCAVOY, H., BERGIN, D., KIERNAN, R., KEATING, T., 2018. Consultation on the development of a Healthy Workplaces Framework for Ireland: A report prepared for the Department of Health. Dublin: Institute of Public Health in Ireland.

NATIONAL NUTRITION SURVEILLANCE CENTRE Childhood Obesity Surveillance Initiative (COSI). [online] available: http://www.ucd.ie/nnsc/research/ourresearch/childhoodobesitysurveillanceinitiativecosi/.

NATIONAL NUTRITION SURVEILLANCE CENTRE Our Aims. [online] Available: http://www.ucd.ie/nnsc/about/ouraims/.

NATIONAL NUTRITION SURVEILLANCE CENTRE. *What we do. [online] Available: http://www.ucd.ie/nnsc/about/whatwedo/#d.en.418586* [Online]. [Accessed].

NATIONAL NUTRITION SURVEILLANCE CENTRE 2009. Public Sector Healthy Food Procurement - A review to identify best practice

NATIONAL UNIVERSITY OF IRELAND, G. *Health Behaviour in School-aged Children (HBSC) Ireland. [online] available http://www.nuigalway.ie/hbsc/hbscireland/backgroundinformation/* [Online]. [Accessed].

OIREACHTAS 2019. Obesity Strategy Dáil Éireann Debate, Tuesday - 12 March 2019. [online] Accessed https://www.oireachtas.ie/en/debates/question/2019-03-12/275/.

POBAL 2016. Healthy Ireland Fund. [online] Accessed on https://www.pobal.ie/programmes/healthy-ireland-fund/.

REVENUE. 2018. *Sugar Sweetened Drinks Tax (SSDT)* [Online]. Available: https://www.revenue.ie/en/companies-and-charities/excise-and-licences/sugar-sweetened-drinks-tax/index.aspx [Accessed].

REVENUE 2019. Vat on food and drink

STANDARDS IN PUBLIC OFFICE COMMISSION 2019. 2018 Code of Conduct for persons carrying on lobbying acitivities. [online] Accessed on https://www.lobbying.ie/media/6119/code-of-conduct-english-final-version-for-web.pdf.

THE IRISH TIMES 2019. Restaurant menus must display calorie counts under new proposals. [online] available: https://www.irishtimes.com/life-and-style/food-and-drink/restaurant-menus-must-display-calorie-counts-under-new-proposals-1.3808080.

TUSLA 2018a. Early Years Quality and Regulatory Framework.

TUSLA 2018b. Quality and Regulatory Framework Full Day Care Service and Part-Time Day Care Service.

WORLD HEALTH ORGANISATION. 2014. *Non-communicable Diseases (NCD) Country Profiles* . [Online]. Available: https://www.who.int/nmh/countries/2014/irl_en.pdf

[Accessed].

-

ANNEX 5:
THE FOOD-ENVIRONMENT POLICY INDEX (FOOD-EPI): NORWAY

The Healthy Food Environment Policy Index (FOOD-EPI)

Evidensdokument for Norge

17. januar 2020

Forord

Om Food-EPI i Norge

The Healthy Food Environment Policy Index (FOOD-EPI) i Norge er et forskningsprosjekt som utføres ved OsloMet ved SIFO og forskningsgruppen samfunnsernæring. Hensikten med prosjektet er å vurdere norske myndigheters arbeid for å fremme sunne matomgivelser (*food environments*). Den overordnede hensikten er å bidra til å styrke innsatsen for å forebygge overvekt, fedme og ikke-smittsomme sykdommer (NCDs) i befolkningen. Prosjektet er knyttet til det internasjonale forskningsnettverket INFORMAS[1] og gjennomføres i forbindelse med JPI-prosjektet Policy Evaluation Network, PEN[2]. Fem europeiske land er tilknyttet PEN og skal gjennomføre Food-EPI prosjekter parallelt med Norge. I tillegg skal seks andre europeiske land tilknyttet STOP-prosjektet (et Horizon2020-prosjekt)[3] også gjennomføre Food-EPI. Det betyr at resultatene fra prosjektet vil kunne sammenliknes med resultatene fra 10 andre europeiske land. Fra før er Food-EPI prosjekter gjennomført i om lag 15 land internasjonalt.

I Norge ledes Food-EPI av Liv Elin Torheim. De andre involverte er Anne Lene Løvhaug, Laura Terragni og Sigrun Henjum (alle fra forskningsgruppen samfunnsernæring) og Gun Roos (SIFO). Laura Terragni har bidratt til å integrere et sterkere fokus på sosial ulikhet i prosjektet. I tillegg til forskningsgruppen har Camilla Sanne Huseby gjort en betydelig innsats for prosjektet ved å samle data for politikkområdene som en del av sin masteroppgave i samfunnsernæring ved OsloMet. Erica Wie Dia har også bistått med praktisk hjelp med prosjektet.

Vi har kvalitetssikret innholdet i evidensdokumentet ved at ressurspersoner fra myndighetene ved Helsedirektoratet, Mattilsynet, Helse- og omsorgsdepartementet og Nærings- og fiskeridepartementet har spilt inn på relevante deler av dokumentet. Vi ønsker å takke for hjelpen vi har mottatt. Så langt som mulig har vi oppgitt referanser til offentlige kilder for at opplysningene skal være etterprøvbare.[4] Til syvende og sist er innholdet i evidensdokumentet prosjektgruppens fulle og hele ansvar.

---

[1] INFORMAS star for *International Network for Food and Obesity / Non-communicable Diseases (NCDs) Research, Monitoring and Action Support*. https://www.informas.org/about-informas/

[2] https://www.jpi-pen.eu/

[3] STOP står for Science and Technology in childhood Obesity Policy: http://www.stopchildobesity.eu/what-is-stop/

[4] I dokumentet er det mange referanser til World Cancer Research Fund Internationals NOURISHING-rammeverk og -database for politikk. Alle referansene lenker til databasens startside. Man finner fram til riktig del av rammeverket ved å se på tittelen i hver enkelt referanse.

Innholdsfortegnelse

Definisjoner ... iv
Forkortelser som brukes i rapporten ... iv
Introduksjon ... 1
Om Food-EPI rammeverket og om oppbygningen av dokumentet ... 2
Veiledning til vurdering av Norges innsats ... 3
Evidens for Food-EPI del 1: Politikkområder ... 4
OMRÅDE 1: SAMMENSETNING AV MATVARER ... 4
SAMM 1 – standarder for sammensetningen av bearbeidet mat ... 5
SAMM 2 – standarder for serveringsbransjen ... 8
OMRÅDE 2: MERKING AV MAT ... 10
MERK 1 – ingrediensliste og næringsdeklarasjon ... 11
MERK 2 – ernærings- og helsepåstander ... 13
MERK 3 – sunnhetsmerking ... 15
MERK 4 – merking av menyer i restauranter ... 18
OMRÅDE 3: MARKEDSFØRING AV MAT ... 20
MARK 1 – regler for kringkastingsmedier ... 21
MARK 2 – regler for digitale og sosiale medier ... 23
MARK 3 – regler i andre medier ... 26
MARK 4 – regler for markedsføring der barn samles ... 28
MARK 5 – regler for markedsføring på emballasje ... 30
OMRÅDE 4: MATPRISER ... 32
PRIS 1 – lave avgifter på sunne matvarer ... 33
PRIS 2 – avgifter på usunne matvarer ... 34
PRIS 3 – subsidier favoriserer sunne matvarer ... 37
PRIS 4 – matrelaterte støtteordninger for sunne matvarer ... 39
OMRÅDE 5: MATOMSORG OG -TILBUD ... 40
MATO 1 – retningslinjer i skoler og barnehager ... 41
MATO 2 – retningslinjer i andre offentlige sammenhenger ... 45
MATO 3 – retningslinjer for matanskaffelse i offentlig sektor ... 48
MATO 4 – støtte- og opplæringssystemer for offentlig sektor ... 49
MATO 5 – støtte- og opplæringssystemer for private bedrifter ... 51
OMRÅDE 6: MAT I HANDELEN ... 52
HANDEL 1 – lovverk og reguleringsplaner begrenser tilgang til usunn mat ... 53
HANDEL 2 - lovverk og reguleringsplaner fremmer tilgang til sunn mat ... 55
HANDEL 3 – støttesystemer til dagligvarehandelen ... 57

HANDEL 4 – støttesystemer for serveringsbransjen 58
Food-EPI del 2 – Infrastruktur-områder 60
OMRÅDE 7: LEDERSKAP 60
LEDER 1 – sterk, synlig politisk støtte 61
LEDER 2 – etablerte mål for kostholdet i befolkningen 63
LEDER 3 – etablerte/implementerte kostråd 65
LEDER 4 – omfattende handlingsplan for ernæring 68
LEDER 5 – redusere sosiale ulikheter i kosthold og helse 71
OMRÅDE 8: STYRESETT OG FORVALTNING 74
STYRE 1 – begrense interessekonflikt i politikkutvikling 75
STYRE 2 – kunnskapsbasert mat- og ernæringspolitikk 77
STYRE 3 – åpenhet i mat- og ernæringspolitikk 79
STYRE 4 – tilgang til informasjon 81
OMRÅDE 9: OVERVÅKING OG DATAINNSAMLING 83
OVER 1 – overvåking av matomgivelser 84
OVER 2 – overvåking av kosthold 87
OVER 3 – overvåking av overvekt og fedme 89
OVER 4 – overvåking av biologiske risikofaktorer og forekomst av NCDs 91
OVER 5 – evaluering av planer og tiltak 93
OVER 6 – overvåking av sosiale helsedeterminanter 94
OMRÅDE 10: FINANSIERING OG RESSURSER 95
FINANS 1 – budsjett som går til ernæring 96
FINANS 2 – finansiering av forskning rundt fedme og NCD-forebygging 98
FINANS 3 – helsefremmende myndighetsorgan 101
OMRÅDE 11: SAMHANDLINGSMEKANISMER 103
SAMHA 1 – koordineringsmekanismer hos myndighetene 104
SAMHA 2 – samarbeid mellom myndighetene og matbransjen 106
SAMHA 3 – samarbeid mellom myndighetene og sivilsamfunn 108
SAMHA 4 – systembasert tilnærming med lokale organisasjoner 109
OMRÅDE 12: "HELSE I ALT VI GJØR" 111
HIAV 1 – vurdering av helsekonsekvenser i matpolitikk 112
HIAV 2 – vurdering av helsekonsekvenser i annen politikk 114
Litteraturliste 116

Definisjoner

Grad av implementering

I hvilken grad myndighetene har implementert politikk eller tiltak sammenlignet med den beste praksisen internasjonalt (referansemåling). Graden av implementering tar i betraktning intensjoner og planer fra myndighetene; tiltak og politikk som helt eller delvis er implementert av myndighetene og i hvilken grad disse er håndhevet; samt myndighetenes finansiering av implementering av tiltak som gjennomføres av ikke-statlige organisasjoner inkludert akademia og private aktører.

Mat

Mat og ikke-alkoholholdig drikke. Alkoholholdig drikke, morsmelk og morsmelkerstatning omfattes ikke av begrepet «mat».

Myndigheter

Statlige myndigheter og andre offentlige instanser.

Næringsstoffer som bør begrenses: salt, mettet fett, transfett og tilsatt sukker.

Politikk (policies)

Politikk brukes her i vid forstand og inkluderer all offentlig politikk, planer, strategier og aktiviteter. Kun gjeldende politikk og tiltak omfattes, generelt definert som politikk og tiltak som implementeres innen den gjeldende stortingsperioden (hvis ikke annet er spesifisert). I vurdering av hvorvidt politikk er implementert, skal det tas hensyn til **grader av implementering**. Dette baseres på hele politikksyklusen, fra agendaen blir satt via utvikling av politikk og tiltak til implementering og monitorering. «Politikk» kan dermed omfatte:

- Reguleringer/lovverk/forskrifter
- Nedsettelse av styringsgrupper, arbeidsgrupper, ekspertpaneler etc.
- Tildeling av ansvar til en person eller et team (dokumentert for eksempel i en arbeidsplan eller en opprettelse av en ny stilling)
- Kartleggingsundersøkelser
- Høringsprosesser og politiske forslag som er sendt til behandling
- Gjennomføringer av ulike konsekvensutredninger
- Monitoreringsdata
- Evalueringer

Sunn/usunn mat: kategorisering av mat som sunn eller usunn er i samsvar med nasjonale kostholdsråd eller definerte standarder, for eksempel nasjonale eller WHO næringsstoffanbefalinger og kostråd, eller ernæringsprofiler.

Forkortelser som brukes i rapporten

FHI:	Folkehelseinstituttet
NCD(s):	Non-communicable diseases dvs. ikke-smittsomme sykdommer
HOD:	Helse- og omsorgsdepartementet

Introduksjon

Hvorfor er det viktig å bedre matomgivelser og forebygge overvekt og fedme i Norge?

I 2020 har de fleste voksne i Norge enten overvekt eller fedme. Omtrent 1 av 4 menn og 1 av 5 kvinner har fedme, altså om lag 23 prosent av befolkningen. Mens forekomsten av overvekt og fedme blant barn tilsynelatende har stabilisert seg det siste tiåret, har den økt blant ungdom, der omtrent 1 av 4 har overvekt eller fedme. Dette er høyere enn i de andre nordiske landene. Både blant barn, ungdom og voksne er det i tillegg en klar sosial gradient, der de med lavest sosioøkonomisk status har høyere risiko for å ha overvekt og fedme (2).

Overvekt og fedme er en av de viktigste risikofaktorene for dødsfall og helsetap i Norge. Nær 8 prosent av alle tapte leveår og 6,5 prosent av landets sykdomsbyrde kan tilskrives høy kroppsmasseindeks, ifølge FHIs rapport om sykdomsbyrden i Norge (3).

Norge har forpliktet seg internasjonalt til å stoppe økningen i overvekt og diabetes. Målet om å stoppe økningen i fedme i Norge har imidlertid ikke lykkes, og utviklingen går i stedet i feil retning (4). Situasjonen tilsier at det behov for økt oppmerksomhet og styrket innsats som kan bidra til å snu utviklingen. Forebygging av overvekt og fedme i befolkingen må rette seg mot en bedring av kostholdet, som fremdeles har vesentlige mangler (5).

En rekke internasjonale strategier og planer som er utarbeidet på høynivå, anbefaler at nasjonale myndigheter implementerer politikk og tiltak for å bedre *matomgivelser* som én del av innsatsen for å nå de globale målene om å redusere forekomsten av ikke-smittsomme sykdommer (NCDs) (6, 7).

INFORMAS definerer matomgivelser som «de kollektive fysiske, økonomiske, politiske og sosiokulturelle omgivelsene, mulighetene og forholdene som påvirker befolkningens mat- og drikkevalg og derigjennom dens ernæringsmessige status» status» (1)

Matbransjen og myndighetene er de to aktørene som har mest makt til å endre på matomgivelsene, gjennom forhold som tilgjengelighet, matens ernæringsmessige kvalitet, pris, merking og markedsføring. Myndighetenes rolle ligger blant annet i å legge rammene som matbransjen må forholde seg til gjennom både obligatoriske og frivillige virkemidler (1). INFORMAS har utviklet en egen modul som fokuserer på matbransjens rolle, mens Food-EPI-modulen kun fokuserer på myndighetenes innsats.

INFORMAS og Food-EPI skal bidra til å ansvarliggjøre myndighetene gjennom monitorering og sammenlikning av politikk mellom land, og i land over tid. Det er behov for å øke oppmerksomheten rundt overvekt og fedme og NCDs som samfunnsutfordringer, slik at innsatsen for å bedre matomgivelser gjennom effektive tiltak kan styrkes. Ekspertpanelet som skal gjennomføre vurderingen kan bidra til en slik økt mobilisering (8).

Om Food-EPI rammeverket og om oppbygningen av dokumentet

Til å vurdere politikk og innsats for å bedre matomgivelser på nasjonalt nivå har INFORMAS utviklet Food-EPI rammeverket (8, 9). Det har to deler: Først en politikk-del som dreier seg om politikk og tiltak som myndigheter kan gjennomføre for å bidra til sunne matomgivelser og å fremme tilgjengelige, rimelige og enkle sunne valg. Politikk-delen består av seks politikkområder der hvert område reflekterer konkrete sider ved matomgivelser der myndighetene kan gjennomføre tiltak. Politikkområdene er valgt ut basert på en litteraturgjennomgang av anbefalte tiltak fra internasjonale organisasjoner, og samsvarer med Word Cancer Research Fund International sin NOURISHING-database (10). Disse politikkområdene er: *1) Sammensetning av matvarer, 2) matmerking, 3) markedsføring av mat, 4) matpriser, 5) matomsorg og -tilbud* og *6) mat i handelen.* Hvert område består igjen av flere *indikatorer* som er utformet som ideelle god praksis-beskrivelser.

Deretter kommer en infrastruktur-del som dreier seg om forhold og systemer som legger til rette for utvikling og implementering av politikk og tiltak. Denne delen består av seks områder: *7) lederskap, 8) styresett og forvaltning, 9) overvåking og datainnsamling, 10) finansiering og ressurser, 11) samhandlingsmekanismer* og *12) «helse i alt vi gjør».* Også her består hvert område av flere indikatorer. Til sammen består rammeverket av 48 indikatorer.

Organiseringen av evidensdokumentet reflekterer inndelingen beskrevet over. For hvert politikk- og infrastruktur-område presenteres først en *visjon* for politikkområdet, for eksempel:

OMRÅDE 1: SAMMENSETNING AV MATVARER

Det er implementert offentlige systemer for å sikre, der det er praktisk mulig, at energitetthet og innholdet av næringsstoff som bør begrenses i bearbeidede matvarer, er minimert (salt, fett, mettet fett, transfett, tilsatt sukker)

Hvert område består av to til seks *indikatorer*, som konkretiserer politikk- eller infrastrukturområdene. For alle indikatorene presenteres følgende informasjon satt opp på denne måten (forklaring i kursiv):

**INDIKATOR 1-6:** ***En kort tittel på indikatoren angir hva den handler om.***	
**God praksis-beskrivelse**<br>*Beskrivelsen utdyper hva indikatoren dreier seg om, og tilsvarer en* ***teoretisk gullstandard****.*	
**Definisjoner og omfang**	*Dette feltet viser hva som omfattes av indikatoren og eventuelle definisjoner.*
**Internasjonale eksempler på god praksis** (referansemål)	*Konkrete eksempler på implementert politikk internasjonalt som vurderes som gode tiltak. Disse eksemplene skal brukes som referansemåling når man vurderer Norges innsats. Eksemplene er satt sammen av INFORMAS og er i mange tilfeller hentet fra Nourishing-databasen. Eksemplene gis på engelsk.*
**Kontekst**	*Dette feltet brukes noen ganger for å informere om forhold som kan ha noe å si for vurderingen av norsk politikk og tiltak, for eksempel om det finnes EU-reguleringer som har innvirkning nasjonalt.*

Evidens for implementering: *Her beskrives tiltak i Norge. Vi presenterer de sterkeste politiske virkemidlene først og de svakeste sist, slik at lover og forskrifter presenteres først og deretter kommer nasjonale retningslinjer og anbefalinger, handlingsplaner, ulike frivillige ordninger osv. Food-EPI-protokollen tilsier at alle deler av politikksyklusen kan tas med i evidensdokumentet. I noen tilfeller har vi derfor valgt å ta med forslag og innspill som ikke er vedtatt politikk.*

Kommentarer: *Dette feltet brukes noen steder til å gi ytterligere informasjon om implementeringen av politikk.*

Veiledning til vurdering av Norges innsats

Et sentralt element i Food-EPI er at politikk og tiltak i Norge skal vurderes opp mot eksemplene på beste praksis internasjonalt. Dette er et bevisst valg fra INFORMAS' side: vurderingen av et lands politikk skal ikke være unødvendig streng, som den kunne blitt dersom man sammenliknet med en teoretisk gullstandard.

I vurderingen, som foregår i Nettskjema, kan du ta hensyn til følgende faktorer:

- *Kvaliteten* på politikk/tiltak sammenliknet med det som er god praksis internasjonalt. For eksempel vil en frivillig ordning ofte vurderes som svakere enn en implementert regulering.
- *Graden av implementering* i forhold til politikksyklusen, fra agenda-setting til implementering og evaluering, sammenliknet med internasjonal god praksis. Høyere grad av implementering gir høyere score/vurdering.
- Du skal **ikke** ta hensyn til helseutfall, slik som overvekt og fedme i befolkningen
- Du skal vurdere status for politikk slik den er nå, og ikke endring over tid.
- I de tilfellene der EU-reguleringer legger føringer på Norges handlingsrom, skal dette tas hensyn til.

Ved vurderingen skal du angi en av følgende vurderinger:

1. 0-20% implementering sammenliknet med internasjonal god praksis (tilsvarer liten eller ingen implementering)
2. 20-40% implementering sammenliknet med internasjonal god praksis
3. 40-60% implementering sammenliknet med internasjonal god praksis
4. 60-80% implementering sammenliknet med internasjonal god praksis
5. 80-100% implementering sammenliknet med internasjonal god praksis (tilsvarer høy grad av implementering)

Når vi analyserer data vil resultatene slås sammen til fire kategorier. Det er dermed ikke av vesentlig betydning å velge akkurat «riktig» kategori.

Fordi vi sammenlikner med internasjonal god praksis, vil det i noen tilfeller ikke finnes eksempler på sterk, implementert politikk. Hvis Norge da har politikk som ikke er perfekt men som er på linje med god praksis, bør det i henhold til INFORMAS-protokollen gis en sterk vurdering. Det samme gjelder der hvor Norge er listet som et eksempel på internasjonal god praksis. I workshopen som skal holdes 11. februar skal det utarbeides forslag til tiltak som Norge kan implementere. Her blir det opp til ekspertpanelet å fremheve områder der det er behov for tiltak, og det kan gjelde både områder der Norge har fått en lav vurdering og områder med et sterkere resultat.

I Nettskjema, der vurderingen foregår, blir det også mulig å gi korte kommentarer hvis det er behov for utdypning.

Evidens for Food-EPI del 1: Politikkområder

OMRÅDE 1: SAMMENSETNING AV MATVARER

Det er implementert offentlige systemer for å sikre, der det er praktisk mulig, at energitetthet og innholdet av næringsstoff som bør begrenses i bearbeidede matvarer, er minimert (salt, fett, mettet fett, transfett, tilsatt sukker).

Det er to indikatorer for område 1:

- SAMM 1 – standarder for sammensetningen av bearbeidet mat
- SAMM 2 – standarder for serveringsbransjen

SAMM 1 – standarder for sammensetningen av bearbeidet mat

<table>
<tr><td colspan="2"><u>God praksis-beskrivelse</u>
Myndighetene har etablert mål eller standarder/grenseverdier for sammensetningen av industrielt bearbeidet mat når det gjelder innholdet av næringsstoff som bør begrenses, spesielt i de matvarene eller matvaregruppene som utgjør en betydelig kilde til befolkningens inntak av disse næringsstoffene (salt, mettet fett, transfett og tilsatt sukker i bearbeidet mat).</td></tr>
<tr><td>Definisjoner og omfang</td><td>• Inkluderer pakkede matvarer produsert i Norge eller produsert i utlandet og importert til Norge.
• Inkluderer pakkede ferdigmatmåltider solgt i supermarkeder.
• Inkluderer obligatoriske eller frivillige mål eller standarder/grenseverdier (for eksempel X% reduksjon, maksimum X mg/g per 100 gram eller porsjon).
• Inkluderer lovfestet forbud mot visse næringsstoffer som bør begrenses.

• Ekskluderer lovfestet forbud som er relatert til andre ingredienser (for eksempel tilsetningsstoffer).
• Ekskluderer obligatorisk regulering av matens sammensetning når det gjelder andre næringsstoffer (for eksempel berikning med folsyre eller jod).
• Ekskluderer mål for inntaket i befolkningen eller standarder (food consumption targets/standards) for fiber og sunne ingredienser som frukt og grønnsaker.
• Ekskluderer sammensetning av ferdigmåltider som tilbys av serveringsbransjen (se SAMM2).
• Ekskluderer generelle retningslinjer som oppfordrer matprodusenter om å redusere næringsstoffer som bør begrenses.
• Ekskluderer ressurser eller ekspertise som støtter individuelle matprodusenter med reformulering (se HANDEL4).

«Industrielt bearbeidet mat» er matvarer som er definert som prosessert eller ultra-prosessert i henhold NOVA-klassifiseringen:
https://world.openfoodfacts.org/nova</td></tr>
<tr><td>Internasjonale eksempler på god praksis (referansemål)</td><td>UK: In 2016, a key commitment of the ‘Childhood obesity: a plan for action’ was to launch a broad, structured sugar reduction programme to remove sugar from everyday products. All sectors of the food and drinks industry were challenged to reduce overall sugar across a range of products that contribute most to children’s sugar intakes by at least 20% by 2020, including a 5% reduction in the first year of the programme (August 2016 to August 2017). The overall reduction between 2015 -2018 (in total sugar per 100 g) was -2.9% (11). Only three food groups of the eight measured have managed at least a 5% reduction in the first year: sweet spreads and sauces, yoghurts and fromage frais, and breakfast cereals. There has been no sugar reduction in biscuits and chocolate bars (12). In contrast to this co-regulation, for products where the sugar tax applies over the same period a reduction in sugar of about 30% was found.</td></tr>
</table>

	**South Africa:** In 2013, the South African Department of Health adopted mandatory targets for salt reduction in 13 food categories by means of regulation (Foodstuffs, Cosmetics and Disinfectants Act). There is a stepped approach with food manufacturers given until June 2016 to meet one set of category-based targets and another three years until June 2019 to meet the next (13, 14). Overall, 67% of targeted foods had a sodium level at or below the legislated limit. About half (49%) of targeted foods not meeting the legislated limits were less than 25% above the maximum sodium level (15).<br><br>**The Netherlands:** In January 2014, the Dutch Ministry of Health, Welfare and Sport signed an agreement with trade organizations representing food manufacturers, supermarkets, hotels, restaurants, caterers and the hospitality industry to lower the levels of salt, saturated fat and calories in food products. The agreement includes voluntary ambitions for the period up to 2020 and aims to increase the healthiness of the food supply (14, 16). Voluntary agreements have been made for the reduction of salt, saturated fat and energy/sugars in a variety of product groups and soft drinks.
**Kontekst**<br>for eksempel EU-reguleringer, tiltak fra matindustrien etc.	En ny EU-forskrift om begrensning i innhold av transfettsyrer i næringsmidler vil bli gjeldende i Norge fra 2021. Denne forskriften vil erstatte den nåværende nasjonale forskriften. Innholdet vil være tilsvarende som i dag, maksimalt 2 gram transfett per 100 gram fett i næringsmidler (17).

Evidens for implementering

Lover og forskrifter

Transfett

Det er gitt en maksverdi på 2 % transfett i fettandel i forskrift om transfettsyrer i næringsmidler, unntatt produkter med naturlig innhold av transfett (kjøtt og melk) (18). Brudd på forskriften kan i henhold til forskriftens §6, straffes med bøter eller fengsel med hjemmel i matlovens § 28 (19).

Frivillige ordninger mellom myndighetene og matvarebransjen

Saltpartnerskapet

Saltinntaket i Norge skal reduseres med 15 prosent innen 2018, 20 prosent innen 2021 og 30 prosent innen 2025 (20). Helse- og omsorgsdepartementet etablerte i 2015 et samarbeid med matvarebransjen, serveringsbransjen, FoU-miljøer og aktuelle bransje- og interesseorganisasjoner om reduksjon av salt. I juni 2019 var rundt 90 aktører tilsluttet Saltpartnerskapet, som har som overordnet målsetning å redusere saltinntaket til 8 g per person per dag innen 2021, til 7,5 gram innen 2025 og på lang sikt 5 g per person per dag. Det er fastsatt konkrete mål for saltinnhold i fem kategorier matvarer: brød og korn, kjøttprodukter, fiskeprodukter, meieri og spisefett og andre matvarer samt en rekke undergrupper for hver av kategoriene. Saltmål for alle kategorier inkludert undergrupper er tilgjengelig på Helsedirektoratets nettside (21), og alle produsenter kan benytte disse uavhengig av deltakelse i partnerskapet. Helsedirektoratet leder arbeidet og har sekretariatsfunksjon. Produktdatabasen Tradesolution er valgt system for monitorering. I tillegg er det gjort analyser av saltinnholdet i 200 indikatormatvarer. I 2019 ble resultater fra perioden 2015-2018 publisert og her vises det at gjennomsnittlig saltinnhold ligger på eller under saltmålet i omtrent 40% av matvarekategoriene. Ved å ta hensyn til grossistvolum viser et vektet gjennomsnitt

at omtrent 60% av kategoriene er på eller under saltmålet (22). Saltpartnerskapet videreføres i perioden 2019-2021.

Intensjonsavtalen
Intensjonsavtalen om tilrettelegging for et sunnere kosthold mellom næringsorganisasjoner, mat- og drikkeprodusenter og dagligvarehandel ble etablert i 2016, som en videreføring av Saltpartnerskapet etter initiativ fra NHO Mat og Drikke. Helsedirektoratet har sekretariatsfunksjon og har ansvar for koordinering og overordnet monitorering av arbeidet. Intensjonsavtalen består av 6 innsatsområder hvor aktørene selv velger hvilke av innsatsområdene 1–4 som er aktuelle for sin virksomhet (1: saltreduksjon, 2: reduksjon av tilsatt sukker, 3: reduksjon av mettet fett, 4: øke inntak av frukt og bær, grønnsaker, grove kornprodukter og sjømat) (20).

Arbeidet med reduksjon av **sukker** ledes av NHO Mat og Drikke. Arbeidet er stilt i bero som resultat av at deler av industrien trakk seg ut av arbeidet i innsatsområdet etter det de opplevde som liten forutsigbarhet pga. endringene i den såkalte sukkeravgiften i statsbudsjettet 2018.

Det er satt konkrete mål for inntaket av **mettet fett** fra kosten. I intensjonsavtalen er målet å redusere inntaket av mettet fett til 13 E% (% av energiinntaket) innen 2018 og på lengre sikt 10 E%. Målet om reduksjon av mettet fett til 13 E% er forlenget til 2021. Innsatsområdet ledes av NHO Mat og Drikke. Det foreligger ikke konkrete mål for matvarekategorier og undergrupper for mettet fett.

Innsatsområde 4 **(øke inntak av frukt og bær, grønnsaker, grove kornprodukter og sjømat)** ledes av tre bransjeorganisasjoner.

Ifølge *Utviklingen i norsk kosthold* har det i perioden 2015-2018 vært ca. 1 prosent økt inntak av grønnsaker, ca. 4 prosent reduksjon av frukt og bær, 15 prosent reduksjon av fisk og om lag 1 prosent økning i andel sammalt mel fra norske møller (23).

Monitorering av intensjonsavtalen baseres på data fra nasjonale undersøkelser som Tromsøundersøkelsen, og andre kilder som Tradesolution, Nielsen og Flesland markedsinfo. Intensjonsavtalen blir også evaluert underveis av en tredjepart, forskningsstiftelsen Fafo (24).

SAMM 2 – standarder for serveringsbransjen

<table>
<tr><td colspan="2"><u>God praksis-beskrivelse</u>
Myndighetene har etablert mål eller standarder/grenseverdier for innholdet av næringsstoff som bør begrenses i måltider som selges av serveringsbransjen, spesielt når det gjelder matvarer eller matvaregrupper som utgjør en betydelig kilde til befolkningens inntak av disse næringsstoffene (transfett, tilsatt sukker, salt, mettet fett).</td></tr>
<tr><td>Definisjoner og omfang</td><td>• «måltider som selges av serveringsbransjen» omfatter måltider som tilbys på hurtigmatkjeder, take-away-steder, kantiner, restauranter, hoteller, bakerier, kaffebarer og snackbarer (også mobile tilbydere slik som food trucks). Kan også omfatte catering og måltider som leveres ferdig på døren.
• Inkluderer lovfestet forbud mot visse næringsstoffer som bør begrenses.
• Inkluderer obligatoriske eller frivillige mål eller standarder/grenseverdier (for eksempel X% reduksjon, maksimum X mg/g per 100 gram eller porsjon).

• Ekskluderer lovfestet forbud som er relatert til andre ingredienser (for eksempel tilsetningsstoffer).
• Ekskluderer obligatorisk regulering av matens sammensetning når det gjelder andre næringsstoffer (for eksempel berikning med folsyre eller jod).
• Ekskluderer kostholdsmål eller standarder (food consumption targets/standards) for fiber og sunne ingredienser som frukt og grønnsaker.
• Ekskluderer generelle retningslinjer som oppfordrer matprodusenter om å redusere næringsstoffer som bør begrenses.
• Ekskluderer ressurser eller ekspertise som støtter individuelle serveringssteder med reformulering (se HANDEL4).</td></tr>
<tr><td>Internasjonale eksempler på god praksis (referansemål)</td><td>New Zealand: In New Zealand, The Chip group, funded 50% by the Ministry of Health and 50% by industry, aims to improve the nutritional quality of deep-fried chips served by food service outlets by setting an industry standard for deep frying oils. The standard for deep frying oil is maximum 28% of saturated fat, 3% linoleic acid and 1% of trans-fat. The Chip group oil logo for use on approved oil packaging was developed in 2010 (25).

New York City (US): In 2009, New York City established voluntary salt guidelines for restaurant and store-bought foods. In 2010, this evolved into the National Salt Reduction Initiative that encouraged nationwide partnerships among food manufacturers and restaurants involving more than 100 city and state health authorities to reduce excess sodium by 25% in packaged and restaurant foods. In 2012, 26% of the categories met the targets, and 3% met the targets by the end of 2014. Between 2009 and 2014, there was nearly a 7% reduction in sodium levels in the U.S. food supply (26). There are 28 companies, including packaged food corporations and restaurants, who are committed to the salt reduction targets (27). In July 25, 2019, the Voluntary Sugar Reduction Targets from the National Salt and Sugar</td></tr>
</table>

	Reduction Initiative were revised. There is an open technical comment period until September 30, 2019 (26).
**Kontekst**<br>for eksempel EU-reguleringer, tiltak fra matindustrien etc.	En ny EU-forskrift om begrensning i innhold av transfettsyrer i næringsmidler vil bli gjeldende i Norge fra 2021. Denne forskriften vil erstatte den nåværende nasjonale forskriften. Innholdet vil være tilsvarende som i dag, maksimalt 2 gram transfett per 100 gram fett i næringsmidler (17).

Evidens for implementering

Lover og forskrifter

Transfett

Gjennom forskrift om transfettsyrer i næringsmidler er det forbudt å omsette produkter med for høy andel transfett, det vil si mer enn 2 gram per 100 gram fett i næringsmidler (18). Forskriften gjelder salg til sluttforbruker, og gjester ved et serveringssted er å regne for sluttforbrukere. Forskrift om internkontroll for å oppfylle næringsmiddellovgivningen (1994) stiller krav om at alle aktører som driver med næringsmidler skal gjennomføre tiltak for å sikre at de er kjent med og etterlever alt regelverk (28).

Frivillige ordninger

Saltpartnerskapet

Flere aktører fra serveringsbransjen deltar i Saltpartnerskapet, blant annet kantiner, restaurantgrupper og drivstoffselskaper. Serveringsbransjens bidrag i avtalen er å arbeide for å redusere saltinnholdet i mat som serveres og selges gjennom: a) Kriterier for saltinnhold ved innkjøp, b) Kompetansehevning for å redusere bruk av salt i tilberedning, servering og salg av mat, og c) Bevisstgjøre forbrukere til å etterspørre mat med mindre salt (22).

Dette inkluderer også å markedsføre produkter, menyer etc. med lavere saltinnhold. Data for omsetning i serveringsbransjen fra Tradesolution (som er en viktig database for monitorering av produkter i matindustrien) er ikke inkludert i monitorering av denne delen av avtalen. Det er i stedet utviklet en rapporteringsmal for kvalitative indikatorer for å følge effekten av saltreduksjonsarbeidet. Det er også igangsatt et utviklingsarbeid for rapportering på kvantitative indikatorer. Resultater fra dette arbeidet vil først foreligge i løpet av neste avtaleperiode.

Intensjonsavtalen

Intensjonsavtalen om tilrettelegging for et sunnere kosthold mellom næringsorganisasjoner, mat- og drikkeprodusenter og dagligvarehandel, ble etablert i 2016. Helsedirektoratet har sekretariatsfunksjon og har ansvar for koordinering og overordnet monitorering av arbeidet. I juni 2018 ble intensjonsavtalen utvidet slik at også aktører fra serveringsbransjen kunne slutte seg til. Ifølge Helsedirektoratet har flere store aktører signert tilslutning, blant annet alle de store drivstoffselskapene. Det er aktører fra serveringsbransjen i alle innsatsområdene. Fafo skal evaluere intensjonsavtalen og midtveisevaluering ventes i 2020 (20).

OMRÅDE 2: MERKING AV MAT

Myndighetene har implementert et forbrukerorientert regelverk for merking av mat på emballasje og av menyer i restauranter, for å gjøre det enkelt for forbrukerne å ta informerte valg og for å forhindre villedende påstander.

Det er fire indikatorer for område 2:

- MERK 1 – ingrediensliste og næringsdeklarasjon
- MERK 2 – ernærings- og helsepåstander
- MERK 3 – sunnhetsmerking
- MERK 4 – merking av menyer i restauranter

MERK 1 – ingrediensliste og næringsdeklarasjon

**God praksis-beskrivelse**<br>**Alle ferdigpakkede matvarer er merket med ingrediensliste og næringsdeklarasjon i tråd med Codex-standarder.**	
**Definisjoner og omfang**	• Inkluderer pakkede matvarer produsert i Norge eller produsert i utlandet og importert til Norge.<br>• «Næringsdeklarasjon» betyr en standardisert beskrivelse av eller liste over næringsinnholdet i en matvare.<br>• Inkluderer transfett og tilsatt sukker, som ikke er spesifisert i de syv elementene i Codex-standarden for næringsdeklarasjon (energi, totalt fett, mettet fett, karbohydrat, sukkerarter, protein, salt).<br>------------------<br>• Ekskluderer helse- og ernæringspåstander (se MERK2).
**Internasjonale eksempler på god praksis (referansemål)**	**EU Regulation** 1169/2011 on the Provision of Food Information to Consumers, passed in 2011, requires a list of the nutrient content of most pre-packaged food to be provided on the back of the pack from 13 December 2016. This Regulation is also applicable in Iceland, Norway and Liechtenstein as members of the European Economic Area (29). The mandatory nutrition declaration shall include the following: Energy value; and the amounts of fat, saturates, carbohydrate, sugars, protein and salt. Where appropriate, a statement indicating that the salt content is exclusively due to the presence of naturally occurring sodium may appear in close proximity to the nutrition declaration (30).
**Kontekst**<br>for eksempel EU-reguleringer, tiltak fra matindustrien etc.	Norge har gjennom EØS-avtalen tatt inn EU-forordningene om matinformasjon og om helse- og ernæringspåstander på næringsmidler. Norge har dermed lite handlingsrom til å innføre merking av næringsstoffer som ikke er oppgitt i EU-forordningen. På grunn av prinsippet om fri bevegelighet av varer og tjenester i EU/EØS er det ønskelig med harmonisering av produktkrav i Europa. Derfor er det lite trolig at Norge kan kreve merking av for eksempel tilsatt sukker. Det vil også være krevende for produsentene å operere med ulik næringsdeklarasjon for indre og ytre marked.

Evidens for implementering

Lover og forskrifter

Matinformasjonsforskriften (Matinformasjonsforskriften, 2014) er en gjennomføringsforskrift for EU-forordninger (31). Forordningen fastsetter at næringsdeklarasjonen skal opplyse om hva ferdigpakkede matvarer inneholder av energi, fett, mettede fettsyrer, karbohydrater, sukkerarter, protein og salt. I tillegg kan det på frivillig basis opplyses om innholdet av enumettede fettsyrer, flerumettede fettsyrer, polyoler, stivelse eller kostfiber. Det er ikke krav om å merke innholdet av tilsatt sukker. Ingredienslisten skal oppgi ingrediensene i en matvare etter vekt. Det er krav om å angi mengde av innholdet av en særlig ingrediens i visse tilfeller, for eksempel hvis en ingrediens

framkommer i matvarens betegnelse (slik som «kjøttpølse»). Mattilsynet har publisert veiledningsmateriell til matinformasjonsforskriften og fører tilsyn med at regelverket overholdes, gjennom løpende tilsyn med næringsmiddelvirksomheter (32, 33).

Kommentarer

Vurdering av denne indikatoren tar utgangspunkt i EU-regelverket, da det per dags dato ikke finnes europeiske eksempler på god praksis.

MERK 2 – ernærings- og helsepåstander

**God praksis-beskrivelse**<br>**Evidensbasert regelverk er på plass for å godkjenne og vurdere påstander på matvarer, slik at forbrukerne er beskyttet mot ubegrunnede og villedende ernærings- og helsepåstander.**	
**Definisjoner og omfang**	• "Ernæringspåstander" omfatter påstander om næringsinnholdet i matvarer (for eksempel "lavt fettinnhold").<br>• "Helsepåstander" omfatter påstander om effekt (slik som "kalsium styrker skjelettet") og påstander som gjelder sammenhengen mellom kosthold, matvarer eller matvarers næringsinnhold og en helseeffekt (slik som "et sunt kosthold med mye grønnsaker og frukt kan bidra til å redusere risikoen for visse kreftformer").<br>• Inkluderer anvendelse av et system for ernæringsprofilering for å klassifisere matvarer som kan/ikke kan benytte helse- eller ernæringspåstander.<br>• "Evidensbasert" betegner systemer som anvender robuste kriterier (som omfattende kunnskapsgjennomganger eller ekspert-vurderinger) eller en oppdatert ernæringsprofil når ernærings- eller helsepåstander vurderes.
**Internasjonale eksempler på god praksis (referansemål)**	**EU Regulation** 1924/2006 establishes EU-wide rules on the use of specified nutrient content and comparative claims (ie levels of fat for a low-fat claim). As of January 2010, only nutrition claims as listed in the Regulation's annex are permitted. In theory, these nutrition claims may only be used on food defined as "healthy" by a nutrient profile. This nutrient profiling restriction was due to be implemented in 2010 but no model has yet been established. Therefore, permitted nutrition claims can be used as long as the conditions for use of the claim as set out in the annex are met. This Regulation is also applicable in Iceland, Norway and Liechtenstein as members of the European Economic Area (29).
**Kontekst** for eksempel EU-reguleringer, tiltak fra matindustrien	EU-regelverket setter ikke krav til at ernærings- og helsepåstander kun kan brukes på visse matvaregrupper. Påstander kan dermed brukes på alle matvarer så lenge kriteriene er oppfylt.

Evidens for implementering

Lover og forskrifter

Forskrift om ernærings- og helsepåstander (34) er en gjennomføringsforskrift for EU-forordning nr. 1924/2006 (påstandsforordningen). Forskriften er hjemlet i matloven. Påstandsforordningen fastsetter at det bare er lov å bruke *tillatte* ernæringspåstander og *godkjente* helsepåstander. En ernæringspåstand sier noe om *«innhold av ernæringsmessig gunstige stoffer»*. En helsepåstand er *«enhver påstand som angir, antyder eller gir inntrykk av at det er sammenheng mellom helse og en næringsmiddelgruppe, et næringsmiddel eller en av dets bestanddeler».* Ernærings- og helsepåstander skal bygge på og være dokumentert ved hjelp av allment anerkjente vitenskapelige

bevis. Tillatte ernæringspåstander og godkjente helsepåstander er vurdert av EFSA og godtatt av EU-kommisjonen.

Godtatte ernæringspåstander finnes som vedlegg til påstandsforordningen. Her angis krav for eksempel for påstander som «lavt energiinnhold», «redusert energiinnnhold», «fettfri», «sukkerfri» etc. Eksempelvis er det tillatt å merke et produkt med «mindre sukker» dersom energiinnholdet fra sukker er redusert med minst 30 %, eller «fri for mettet fett» gitt at mengden mettet fett og transfett ikke til sammen overstiger 0,1 g per 100 g eller ml. Oversikt over godkjente eller avslåtte helsepåstander er tilgjengelig på Mattilsynets nettsider (35).

Mattilsynet har publisert en veileder om bruk av ernæringspåstander og helsepåstander (36). I veilederens kapittel 7.2.1 oppfordrer Mattilsynet matbransjen om å ikke bruke ernærings- og helsepåstander på matvarer med en ugunstig ernæringsprofil:

> *Det er likevel klokt å bruke ernærings- og helsepåstander bare om de næringsmidlene som er ernæringsmessig gunstige. Et viktig formål med påstandsregelverket er å beskytte forbrukerne mot villedende markedsføring og å gjøre det enklere å velge matvarer som bidrar til et sunt og variert kosthold. Dette vil være enklere å oppfylle dersom påstandene forbeholdes matvarer som kan kalles sunne.*
>
> *Dere må selv vurdere om en påstand som er brukt på et konkret produkt, kan være villedende fordi den fremmer bruk av matvarer helsemyndighetene fraråder et høyt eller jevnlig inntak av. Som hjelpemiddel kan det være naturlig å bruke helsemyndighetenes føringer gjennom for eksempel Helsedirektoratets kostråd med utdypende undertekster, og Nøkkelhullet. Dere kan også bruke den såkalte MFU-listen fra Matbransjens faglige utvalg*. (s.102)

Mattilsynet fører tilsyn med at næringsmidler er merket i samsvar med regelverket. Dette gjelder både generell merking (ingrediensliste og næringsdeklarasjon) og ernærings- og helsepåstander. Mattilsynet kan fatte vedtak ved brudd på regelverket. Mattilsynet har gjennomført ett nasjonalt tilsynsprosjekt om bruk av ernærings- og helsepåstander, i 2014 (37).

MERK 3 – sunnhetsmerking

**God praksis-beskrivelse**<br>**Et (eller flere) veiledende, evidensbaserte FOP (*front-of-pack*) supplerende ernærings-informasjonssystem(er), som er godkjent av helsemyndighetene og som gjør det enkelt for forbrukere å vurdere produktets sunnhet, brukes på alle ferdigpakkede matvarer (eksempler: Nutriscore og trafikklys-merking)**.	
**Definisjoner og omfang**	• FOP har ingen etablert norsk betegnelse. Begrepet henviser til ulike typer veiledende merking som settes på fremsiden av en forpakning for å hjelpe forbrukeren med å forstå næringsinnholdet i en matvare.<br>• Supplerende betyr at merkingen kommer i tillegg til ingrediensliste og næringsdeklarasjon.<br>• Kategorien FOP-merking som kalles ernærings-informasjonssystemer kan være av typen *summerende indikatorsystemer* der nivåer av næringsinnhold kombineres til å gi en helhetsvurdering av en matvares sunnhet (eksempel: NutriScore eller angivelse av næringsinnhold med stjerner eller poeng), *næringsstoff-spesifikke veiledende merker* der informasjon om næringsstoffene presenteres separat (slik som trafikklys eller Percent Daily Intake) eller *næringsstoff-spesifikke advarselsmerker*.<br>• "Evidensbasert" betegner systemer som anvender robuste kriterier (som omfattende kunnskapsgjennomganger eller ekspert-vurderinger) eller en oppdatert ernæringsprofil når ernærings- eller helsepåstander vurderes.<br>• Nøkkelhullet og det finske hjertesymbolet regnes i Food-EPI ikke som FOP-merking/ernærings-informasjonssystemer, men som ernæringspåstander fordi merkene brukes på sunnere alternativer basert på en helhetsvurdering og kun brukes på utvalgte matvarekategorier.
**Internasjonale eksempler på god praksis (referansemål)**	**UK:** In 2013, the Government published national guidance for voluntary 'traffic light' labelling for use on the front of pre-packaged food products. The label uses green, amber and red to identify whether products contain low, medium or high levels of energy, fat, saturated fat, salt and sugar. A combination of colour coding and nutritional information is used to show how much fat, salt and sugar and how many calories are in each product. The voluntary scheme is used by all the major retailers and some manufacturers (38).<br><br>**Australia/New Zealand:** The government-approved, voluntary 'Health Star Rating' (HSR) scheme applies a stare rating system where ratings range from ½ star (least healthy) to 5 stars (most healthy). The rating is based on the content of energy, saturated fat, sodium and total sugars content, along with certain 'positive' aspects of a food such as fruit and vegetable content, and in some instances, dietary fibre and protein content. Implementation of the system began in June 2014 and is overseen by a number of governmental instances, one of which evaluates progress. As of 2016, about 900 products had stars on them (39).

	**France/Belgium/Germany/Spain**: Since October 2017, the five-colour NutriScore, the official voluntary label for France has been implemented as the official, voluntary FOP scheme in four European countries. It aims to limit the consumption of foods high in energy, saturated fats, sugar or salt, in the context of an overall improvement in the nutritional quality of diets. Based on a scientific algorithm, each product is given a score based on the content of the nutrients of concern (energy value and the amount of sugars, saturated fats and salt) and positive ones (the amount of fibre, protein, fruit, vegetables and nuts) (40). The system was developed by the Nutritional Epidemiology research Team at the University of Paris (41).<br>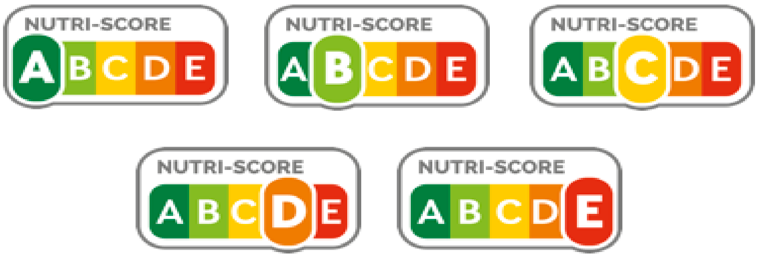<br>*Figur 1. NutriScore*
**Kontekst**<br>for eksempel EU-reguleringer, tiltak fra matindustrien etc.	Norge har felles regelverk med EU for merking av matvarer. Det er i praksis liten anledning til å etablere særregler for ett land hva angår merking. Nye merkeordninger må meldes til EU, og kan ikke gjennomføres hvis det kommer negative tilbakemeldinger. På nåværende tidspunkt tillater ikke EU obligatorisk merking med sunnhetsmerker/FOP-merking. Dette må eventuelt vedtas på europeisk nivå.

Evidens for implementering

Det er to supplerende sunnhetsmerker støttet av myndighetene, i bruk i Norge: Nøkkelhullet og Brødskalaen. Begge ordningene er frivillige.

Nøkkelhullet er et sunnhetsmerke som brukes av helse- og matmyndighetene i Sverige, Danmark, Island og Norge. Nøkkelhullet skal gjøre det enkelt for folk å velge sunnere matvarer *innen matvaregrupper* og dermed øke muligheten til å sette sammen et sunt og variert kosthold i tråd med myndighetenes kostråd (42). Premisset i ordningen er at matvarer merket med Nøkkelhull er et sunnere valg sammenliknet med andre matvarer av samme type. Nøkkelhullet er et symbol for ernæringspåstander og faller inn under bestemmelsene i forskrift om helse- og ernæringspåstander (34).

Nøkkelhullet setter krav til minimumsinnhold av fiber og maksimumsinnhold av fett, mettet fett, salt og sukker innenfor 33 matvaregrupper. I tillegg er det satt krav til minimumsinnhold av sammalt mel (fullkorn), grønnsaker, frukt og bær i noen matvaregrupper. Samtlige kriterier i den aktuelle kategorien må være oppfylt for å kunne merke produkter med Nøkkelhullet. De ernæringsmessige kriteriene i ordningen tar

utgangspunkt i de felles nordiske ernæringsanbefalingene og nasjonale kostanbefalinger. Ordningen gjelder først og fremst ferdigpakkede matvarer, men kan også brukes på uemballert fisk, frukt og grønnsaker, brød/knekkebrød, ost og kjøtt. Kriteriene for bruk av Nøkkelhullet er i Norge fastsatt i forskrift om frivillig merking med Nøkkelhullet (43).

Ifølge Helsedirektoratet er kunnskapen om og tilliten til merkeordningen god i befolkningen: nær alle kjenner til ordningen, og 7 av 10 opplever ordningen som god (44). Det har vært mer utfordrende å få matbransjen til å bruke ordningen. Ved innføring av ordningen i 2009 var 430 produkter merket med Nøkkelhullet. Antallet økte til 2284 i 2016, og har deretter sunket til 1807 i 2018 (23).

Brødskalamerket Brødskala'n er ikke initiert av myndighetene, men er tatt med i dette dokumentet siden merkeordningen er utarbeidet i samarbeid mellom myndighetene og bransjen, og fordi Helsedirektoratet aktivt promoterer merket blant annet på helsenorge.no. NHO Mat og Drikke, Baker- og Konditorbransjens Landsforening, Forbrukerrådet, Sosial- og helsedirektoratet og Mattilsynet deltok ved utviklingen av merket i 2006. Merkeordningen eies og administreres av mel- og bakeribransjen (45).

Merkeordningen er utviklet for å gjøre det lettere å anslå grovheten på brød. Brødskala'n er en sirkel med fire «kakestykker» hvor antallet fargelagte «kakestykker» viser andelen fullkorn i produktet. Ett «kakestykke» representerer 0 - 25,9 % fullkorn, to «kakestykker» 26 - 50,9 %, tre «kakestykker» 51 - 75,9 % og fire «kakestykker» 76 - 100 % fullkorn. I tilknytning til merket skal også andelen fullkorn angis i %, etter en revisjon i 2017 (45). Mattilsynets årlige merkeprosjekt hadde i 2018 fokus på brød og brødvarer, blant annet bruk av Brødskala'n (46). Mattilsynet fattet ikke vedtak ved feil bruk, men veiledet fordi Brødskalaen og kriteriene for å bruke merkeordningen ikke er hjemlet i regelverket. Rapporten oppgir ikke andel brød og brødvarer som var merket med brødskalaen.

Kommentarer

Fordi Nøkkelhullet er et symbol for ernæringspåstander, faller ordningen egentlig utenfor omfanget av indikatoren. Vi har imidlertid valgt å ta med ordningen for å vurdere Nøkkelhullet som sunnhetsmerking opp mot andre ordninger som myndighetene har mulighet til å implementere i tråd med EØS-avtalen, spesielt NutriScore. Brødskala'n faller heller ikke inn under kriteriene til et sunnhetsmerke som definert av INFORMAS, men tas med her fordi merket i praksis promoteres som et sunnhetsmerke av Helsedirektoratet.

MERK 4 – merking av menyer i restauranter

**<u>God praksis-beskrivelse</u>**<br>**Myndighetene har implementert et enkelt og godt synlig system for merking av menyer hos alle hurtigmatkjeder, slik at forbrukere kan forstå næringsverdien og energiinnholdet i mat og måltider som tilbys.**	
**Definisjoner og omfang**	• "Hurtigmatkjeder" omfatter fast food-steder/-kjeder, men også kaffebarer-, -bakeri- og kiosk-kjeder samt bensinstasjon-kjeder. Det kan også omfatte supermarkeder som selger ferdigmat.<br>• "Merking" omfatter alle former for ernæringsinformasjon ved salgspunktet, som totalt energiinnhold, prosentandel av daglig inntak, trafikklys, poenggivning eller spesifikt innhold av næringsstoffer som bør begrenses.<br>• Inkluderer godkjente sunnhetsmerker på menyer eller matvarer som oppfyller krav (for eksempel Nøkkelhull).
**Internasjonale eksempler på god praksis (referansemål)**	**South Korea:** Since 2010, the Special Act on Safety Control of Children’s Dietary Life has required all chain restaurants with 100 or more establishments to display nutrient information on menus including energy, total sugars, protein, saturated fat and sodium (29).<br><br>**Canada:** In effect since 1 January 2017, Ontario’s Healthy Menu Choices Act 2015, requires food service premises that are part of a chain of 20 or more food service premises in Ontario (as well as certain cafeteria-style food service premises) to display calories for “standard food items” on menus, labels and display tags. The Act’s regulations specify where caloric information is to be displayed on the menus, as well as the size, format and prominence of the display (47). Food service premises must also display information on daily caloric requirements: “Adults and youth (ages 13 and older) need an average of 2,000 calories a day, and children (ages 4 to 12) need an average of 1,500 calories a day. However, individual needs vary.” Ontario’s 36 public health units are responsible for implementation of the Act (47).<br><br>**Saudi Arabia:** In 2018, the Saudi Food and Drug Authority (SFDA) introduced mandatory measures on calorie labels on menus. These measures apply to all food facilities including restaurants, ice cream parlours, juice and fresh fruit vendors, bakeries, sweets shops, cafeterias, supermarkets, recreation facilities, colleges, universities and government agencies. Calories will be displayed at cashier desks, menu boards, table menus, drive-through menus, phone and web applications (48).

Evidens for implementering

I Norge er det ikke krav til opplysninger av næringsinnhold på menyer til serveringsbransjen. Eventuell frivillig merking skal være i tråd med regelverket for merking av emballert mat (31). Noen

serveringssteder har næringsinnhold på retter tilgjengelig for sine kunder. Det finnes ingen oversikt over hvor mange dette gjelder.

Nøkkelhullet kan brukes av serveringsbransjen på de matvaregruppene som kan omsettes ikke ferdigpakket. Dette er gruppene grønnsaker, frukt/bær, brød/knekkebrød, ost, samt uforedlede fiskerivarer og kjøtt. Det finnes ingen oversikt over bruken av Nøkkelhullet i serveringsbransjen.

OMRÅDE 3: MARKEDSFØRING AV MAT

Myndighetene har implementert omfattende ordninger for å redusere påvirkningen (eksponering og styrke) av markedsføring av usunn mat til barn i alle medier.

Det er fem indikatorer for dette området:

- MARK 1 – regler for kringkastingsmedier
- MARK 2 – regler for digitale og sosiale medier
- MARK 3 – regler i andre medier
- MARK 4 – regler for markedsføring der barn samles
- MARK 5 – regler for markedsføring på emballasje

Kontekst – informasjon som skal tas i betraktning ved vurdering av alle indikatorene:

I 2012 fremmet helsemyndighetene et forslag til en forskrift som skulle regulere markedsføring av usunn mat til barn og unge under 18 år. Forslaget ble senere endret til å skulle gjelde barn under 16 år. Etter planen skulle Helsedirektoratet være tilsynsmyndighet for regelverket, og Markedsrådet skulle være klageinstans (49). I 2014 inngikk myndighetene i stedet en avtale med matvarebransjen om en selvreguleringsordning for markedsføring av mat og drikke rettet mot barn. Matbransjens faglige utvalg (MFU) ble opprettet i 2014 og forvalter selvreguleringsordningen. MFU er opprettet for formålet og eies av Annonsørforeningen (ANFO), Næringslivets hovedorganisasjon (NHO) og Virke (hovedorganisasjonen for tjenestenæringene) (50). Ordningen omfatter en retningslinje for markedsføring av mat og drikke rettet mot barn (51), en veileder til retningslinjen (52) og en veileder til bruk av sosiale medier (53). Produktene som omfattes er gitt i en produktliste med forklaring hvor det defineres hvilke produkter retningslinjen og veilederne omfatter (54). Retningslinjen og veilederne gjelder som et supplement til markedsføringsloven og kringkastingsloven (MFU, 2016b). Markedsføring av produkter angitt på produktlisten skal ikke være «særlig rettet mot barn under 13 år» (51). Sanksjonene som gis av MFU består ifølge MFU selv i at vedtak som er fattet publiseres via nettsiden (51) Sanksjonene som gis av MFU består ifølge MFU selv i at vedtak som er fattet publiseres via nettsiden www.mfu.as, på Facebook og via NTB info.

Høsten 2019 ble retningslinjene i MFU endret og retningslinjene om aktsomhet overfor ungdom har blitt strengere. Markedsføringsaktiviteter som i hovedregel *ikke regnes som aktsomme* omfatter nå blant annet «*konkurranser med lavere aldersgrense enn 16 år; kjøp av målgrupper under 16 år for markedsføring på nett, sosiale medier e.l; bruk av rollemodeller med særlig appell til ungdom i markedsføring som, gjennom valg av markedsføringskanal, er direkte rettet til ungdom; og oppfordringer til handling/liking/deling/sende inn materiale, hvor ungdommen selv blir spreder av et reklamebudskap*» (51).

Helsemyndighetene er representert i MFUs utvalg som ett av syv medlemmer. Utvalget har ansvar for behandling av klagesaker og kan anbefale revidering av retningslinjer, veiledning og produktliste. Helsemyndighetene har gjennomført evalueringer av MFU. Ordningen ble evaluert av Helsedirektoratet i 2016 og ble evaluert av Folkehelseinstituttet i 2019, etter oppdrag fra Helse- og omsorgsdepartementet. Resultatet av evalueringen var ikke klart da dette dokumentet ble ferdigstilt.

MARK 1 – regler for kringkastingsmedier

<table>
<tr><td colspan="2"><u>God praksis-beskrivelse</u>
Myndighetene har implementert effektive ordninger for å begrense eksponeringen og styrken fra markedsføring av usunn mat og drikke til barn og ungdom gjennom kringkasting (TV og radio).</td></tr>
<tr><td>Definisjoner og omfang</td><td>• Inkluderer regulering og lovgivning eller frivillige regler, avtaler eller retningslinjer som er utarbeidet enten av myndighetene eller av matvarebransjen men der myndighetene spiller en rolle i utvikling, monitorering, håndhevelse, eller klagehåndtering.
• Omfatter TV og radio, inkludert abonnement/strømmetjenester.
• «Effektiv» betyr at ordningene kan ventes å redusere barn og ungdoms totale eksponering av markedsføring for mat og drikke.</td></tr>
<tr><td>Internasjonale eksempler på god praksis (referansemål)</td><td>Quebec: In Québec, the Consumer Protection Act prohibits commercial advertising (including food and non-food) directed at children less than 13 years of age through television, radio and other media. Account must be taken of the context of its presentation, and in particular of: a) the nature and intended purpose of the goods advertised; b) the manner of presenting such advertisement; and c) the time and place it is shown. A cut-off of 15% share of child audience is used to protect children from TV advertising. Per indictment, a person is liable to: a fine ranging from $600 to $15,000 (in the case of a natural person); a fine ranging from $2,000 to $100,000 (in the case of a legal person) (55).

Norway/Sweden: Under the Broadcasting Act, advertisements (food and non-food) may not be broadcast on television directed to children or in connection with children’s programs. (24). Norway has implemented a self-regulation scheme approved and evaluated by Government. The scheme prohibits child-targeted unhealthy food marketing before 21:00 (9 PM) (56).

Ireland: Advertising, sponsorship, teleshopping and product placement of foods high in fats, sugars and salt, as defined by a nutrient profiling model, are prohibited during children’s TV and radio programmes where over 50% of the audience are under 18 years old. In addition, there is an overall limit on advertising of foods high in fats, sugars and salt adverts at any time of day to no more than 25% of sold advertising time and to only one in four advertisements. Remaining advertising targeted at children under the age of 18 must not include nutrient or health claims or include licensed characters As provided under the Broadcasting Act 2009, the BAI is required to undertake a statutory review of the effectiveness of the Children’s Code (57).

Chile: In June 2016, The Law of Nutritional Composition of Food and Advertising was enforced and restricts advertising directed to children under 14 years (for foods exceeding limits for calories, sugar, saturated fat and/or sodium in food and beverages). The regulatory norms define advertising targeted to children as programmes with an audience of greater than 20% children. Promotional strategies and incentives, such as cartoons, animations, and toys that could attract the attention of children are included in the ban. Monitoring and enforcement of the law are carried out</td></tr>
</table>

	by both regional and national public health authorities. Inspections are conducted on-site and online. After more than 2000 inspections, compliance with the law is improving, rising from under 40% to over 60% (58). A qualitative study carried out in 2017, found that the regulation has made mothers more aware of the importance of eating healthy, made it easier to choose healthy foods, and also made children actors in their own food choices (59).

Evidens for implementering

Lover og forskrifter
Kringkastingsloven trådte i kraft i 1993/4, og omfatter kringkastede medier inkludert audiovisuelle bestillingstjenester i Norge (60). Markedsføring av alle typer produkter som er «særlig rettet mot barn» samt produktplassering og sponsing er forbudt i forbindelse med barneprogrammer. Dette inkluderer reklame for mat og drikke. Forskrift om kringkasting omtaler «reklame og forholdet til barn og unge under 18 år» og påpeker at det må vises særlig aktsomhet overfor barn og unges påvirkelighet og manglende erfaring (61).

Forbrukertilsynet fører tilsyn med reklame rettet mot barn og unge, og har publisert en veiledning om handelspraksis overfor barn og unge (62). I vurderingen av om reklame er «særlig rettet mot barn», skal det tas hensyn til om reklamen gjelder et produkt eller en tjeneste av særlig interesse for barn, sendetidspunkt for reklamen, om det medvirker barn under 13 år eller om det benyttes animasjon eller annen presentasjonsform som særlig appellerer til barn. Det er dermed flere kriterier som må oppfylles hvis en reklame skal bli vurdert som «særlig rettet mot barn». En animert reklamefilm for Litago som ble sendt på TV kl. 20, der målgruppen var barn, ble av Markedsrådet (klageorgan til Forbrukertilsynet) ikke vurdert som «særlig rettet mot barn».

Frivillige ordninger
MFUs retningslinjer for markedsføring av mat og drikke rettet mot barn gjelder for barn under 13 år, og angir at reklame som er «særlig rettet mot barn» ikke skal vises i fjernsyn før kl. 21 (52). Barneprogrammer i TV og radio er beskrevet som medier som normalt vil kunne anses for å ha særlig appell til barn.

I vurderingen av om en markedsføringsaktivitet er særlig rettet mot barn skal det tas hensyn til tre faktorer som inngår i en helhetsvurdering hvor produktets grad av appell til barn under 13 år avgjør hvor strengt bruken av medier og virkemidler skal bedømmes:

- I hvor stor grad det markedsførte produktet appellerer særlig til barn.
- I hvor stor grad medier som er brukt særlig appellerer til barn.
- I hvor stor grad virkemidlene som er brukt særlig appellerer til barn.

MARK 2 – regler for digitale og sosiale medier

**<u>God praksis-beskrivelse</u>**<br>**Myndighetene har implementert effektive ordninger for å begrense eksponeringen og styrken fra markedsføring av usunn mat og drikke til barn og ungdom gjennom digitale og sosiale medier.**	
**Definisjoner og omfang**	• Inkluderer online media (for eksempel sosiale medier, nettsteder som eies av matbransjen, online spill, konkurranser og andre apper).<br>• «Effektiv» betyr at ordningene kan ventes å redusere barn og ungdoms totale eksponering av markedsføring for mat og drikke.<br>• Omfatter ikke markedsføring i andre medier (se MARK 1, 3 og 5).<br>• Omfatter ikke markedsføring i sammenhenger der barn samles (se MARK 4).
**Internasjonale eksempler på god praksis (referansemål)**	**Chile:** In June 2015, the Chilean authority approved the regulatory norms required for the law of Nutritional Composition of Food and Advertising implementation. The regulatory norms define limits for calories, saturated fat, sugar and sodium content considered "high" in foods and beverages. The law restricts advertising directed to children under the age of 14 years of foods in the "high in" category. The regulatory norms define advertising targeted to children as websites directed to children or with an audience of greater than 20% children, and according to the design of the advertisement. Promotional strategies and incentives, such as cartoons, animations, and toys are included in the ban. The regulation took effect 1 July 2016 and applies to all advertising media (55).<br><br>**Quebec:** In Québec, the Consumer Protection Act prohibits commercial advertising directed at children less than 13 years of age through all media. Account must be taken of the context of its presentation, and in particular of: a) the nature and intended purpose of the goods advertised; b) the manner of presenting such advertisement; and c) the time and place it is shown (63). Any stakeholder involved in a commercial process (from the request to create an advertisement to its distribution, including its design) may be accused of not complying with the legislation in force. Per indictment, that person is liable to: a fine ranging from \$600 to \$15,000 (in the case of a natural person); a fine ranging from \$2,000 to \$100,000 (in the case of a legal person).<br><br>**Norway:** A government-approved and evaluated self-regulation scheme prohibits online food-marketing which is targeted at children under 13 years. Specifically, interactive games "specifically aimed at children and where a product's trademark, or other elements of the marketing of the product, form an integral part" will always be defined as child-targeted and a violation of the code. The scheme also applies to social media. In 2019, the code was revised. The age limit is still 13 years but in order to exercise caution in marketing to young people, it is advised to not conduct contests with age limit less than 16 years; to buy age groups less than 16 years in digital media; to use role models appealing to youth in media which is directly targeted to youth; and to encourage engagement (share, like, send in material) so that youth become marketing actors. Violations of the code

	results in no other sanctioning than “naming and shaming” of offenders (56).<br><br>**UK:** UK CAP rules have been reviewed so that online marketing targeted to under-16s is prohibited. This means that HFSS product ads are not permitted to appear in media that is specifically targeted at under-16s (for example, a children’s magazine or on a website aimed at children); or where under-16s make up a significant proportion (more than 25%) of the audience (for example, advertorial content with an influencer that might have broad appeal but also a significant child audience) (64).

Evidens for implementering

Lover og forskrifter
Markedsføringsloven (65) omfatter reklame i alle medier (unntatt de som faller inn under kringkastingsloven) og håndheves av Forbrukertilsynet. Det er ikke noe forbud mot markedsføring rettet mot barn, men det skal vises særlig aktsomhet overfor barn under 18 år. Dette kommer frem i markedsføringslovens barnekapittel.

Forbrukertilsynet har utarbeidet en veileder om handelspraksis overfor barn og unge, som også gjelder i digitale medier. Markedsføringslovens barnekapittel sier at det ved markedsføring må vises særlig aktsomhet overfor barn, som er definert som mindreårige under 18 år. Det skal «vises særlig aktsomhet overfor barns påvirkelighet, manglende erfaring og naturlige godtroenhet». Dette innebærer at reklame ikke skal være skjult, aggressiv eller villedende; og at den ikke skal inneholde direkte kjøpsoppfordringer («løp og kjøp»). Barnebegrepet er imidlertid fleksibelt, hvilket betyr at når Forbrukertilsynet vurderer et markedsføringstiltak vil vektlegge målgruppens alder og utvikling. For yngre barn vil vurderingen være strengere (62). Konkurranser vil kunne ses på som urimelig handelspraksis når barn er målgruppen, men dette avhenger av en helhetsvurdering. Det beskrives også at deling i sosiale medier er et eksempel på en handelspraksis «vil kunne være» urimelig ovenfor barn. Ved brudd på Markedsføringsloven kan Forbrukertilsynet fatte vedtak om forbud, påbud, tvangsmulkt og overtredelsesgebyr.

Forbrukertilsynet har også publisert en veileder om merking av reklame i sosiale medier (66). I veilederen defineres barn som under 18 år. Veilederen er rettet mot alle som legger ut reklame i sosiale medier. Den forklarer i detalj hvordan merkingen skal være slik at det tydelig fremgår at innlegget er reklame. Det gis også forklaringer og eksempler på hva det innebærer å være varsom overfor barn. Her legges det vekt på forbudet mot skjult reklame; direkte kjøpsoppfordringer; og villedende reklame.

Frivillige ordninger
MFU retningslinjer for markedsføring av mat og drikke rettet mot barn omfatter også digitale medier. Retningslinjene slår fast at «interaktive spill særlig rettet mot barn og hvor et produkts varemerke, eller andre elementer fra markedsføringen av produktet inngår som en integrert del» alltid skal regnes som markedsføring rettet mot barn under 13 år og dermed vil være i strid med retningslinjene (51). Figur 2 viser eksempel på et interaktivt spill som har blitt felt av MFU.

MFU har også utarbeidet en egen veileder for markedsføring av mat i sosiale medier (53). Aldersgrensen for markedsføring mot barn er 13 år. Veilederen påpeker at det ved markedsføring via sosiale medier må tas hensyn til at disse mediene kan oppsøkes døgnet rundt, i motsetning til

kringkasting hvor markedsføringen kan tilpasses etter når på døgnet reklamen skal sendes. MFU tilbyr forhåndsvurdering av markedsføring også på sosiale medier. I vurderingen legges det vekt på i hvilken grad det markedsførte produktet herunder emballasjen, virkemidlene og det aktuelle mediet appellerer til barn.

Høsten 2019 ble MFUs retningslinjer endret. Ifølge de reviderte retningslinjene skal det utvises aktsomhet overfor ungdom under 16 år. For eksempel vil kjøp av målgrupper under 16 år eller bruk av rollemodeller som er attraktive for ungdom under 16 år i hovedregel ikke regnes som aktsomme.

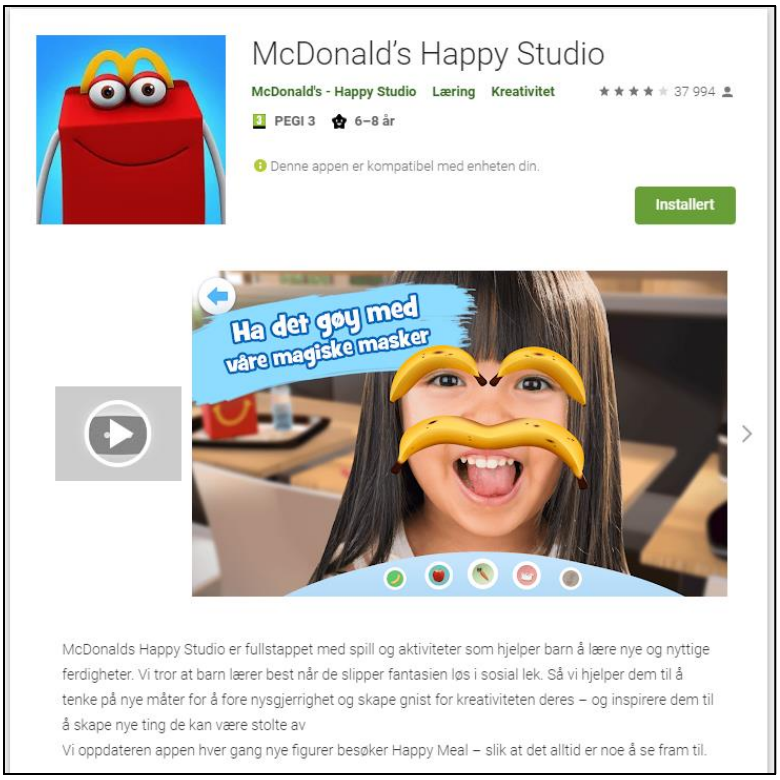

Figur 2. En klage på app'en Happy Studio ble tatt til følge av MFU i 2019.

MARK 3 – regler i andre medier

<table>
<tr><td colspan="2"><u>God praksis-beskrivelse</u>
Myndighetene har implementert effektive ordninger for å begrense eksponeringen og styrken fra markedsføring av usunn mat og drikke til barn og ungdom gjennom alle medier unntatt kringkasting, digitale medier og matemballasje.</td></tr>
<tr><td>Definisjoner og omfang</td><td>Definisjonen omfatter alle andre medier unntatt kringkasting, digital og emballasje:
• Trykket (magasiner, blader og plakater)
• På offentlig transport (skilt, postere, reklametavler)
• Kinoreklame
• Produktplassering
• Ved kjøpspunkt og direkte markedsføring [utdeling av vareprøver etc]
• «Effektiv» betyr at ordningene kan ventes å redusere barn og ungdoms totale eksponering av markedsføring for mat og drikke.
• Omfatter ikke medier som er omfattet av MARK 1, 2 og 5.
• Omfatter ikke markedsføring i sammenhenger der barn samles (se MARK 4).</td></tr>
<tr><td>Internasjonale eksempler på god praksis (referansemål)</td><td>Chile: In May 2018, a new regulation launched, extended marketing restrictions of regulated foods in cinema and TV to a 6 AM to 10 PM time frame, expanding the scope of the original law. Additionally, starting in June 2018, any marketing done for “High in” foods or beverages must also show the following statement: “Choose foods with less warning labels” and then “Ministry of Health,” which needs to be placed next to the MoH logo. This applies to marketing done in billboard, cinema, and other vehicles but food packages (67).

London UK: On 25 February 2019, the Mayor of London introduced restrictions on the advertising of unhealthy food across the entire Transport for London (TfL) public transport network, as part of his work to help tackle childhood obesity in London. The policy specifies that food and non-alcoholic drinks high in fat, salt and sugar (according to the UK Nutrient Profiling Model), are not permitted to be advertised on TfL-controlled buses, underground and over ground train networks, taxis, river services, trams and other transport systems. Food and drink brands, restaurants, takeaways and ordering services are required to promote their healthier food and drink instead of just advertising their brand. Advertisements for food and non-alcoholic drink products that are considered to be high in fat, salt, sugar may be considered for an exception by TfL if the advertiser can demonstrate, with appropriate evidence, that the product does not contribute to child obesity (68).</td></tr>
</table>

Evidens for implementering

Lover og forskrifter
Markedsføringsloven omfatter som tidligere nevnt reklame i alle medier unntatt de som faller inn under kringkastingsloven (65). Det er ikke noe forbud mot markedsføring rettet mot barn, men det skal vises særlig aktsomhet overfor barn under 18 år. Dette innebærer blant annet at reklame ikke skal være skjult, villedende eller inneholde direkte kjøpsoppfordringer. Forbrukertilsynet fører tilsyn og kan fatte vedtak om forbud, påbud, tvangsmulkt og overtredelsesgebyr.

Frivillige ordninger
MFUs retningslinjer angir at følgende markedsføringsaktiviteter alltid skal anses som en *overtredelse* av retningslinjene:

- reklame på kino i tilknytning til filmer som er særlig rettet mot barn under 13 år, og som starter før kl. 18.30
- alle former for konkurranser med aldersgrense lavere enn 13 år
- reklame i tegneseriehefter rettet mot barn

Følgende aktiviteter er *unntatt* fra retningslinjene:

- utdeling av vareprøver etter samtykke fra foreldre eller andre ansvarlige
- alminnelig oppstilling av produkter på utsalgssted

Unntaket om alminnelig oppstilling gjelder likevel ikke hvis en utstilling benytter seg av virkemidler som særlig appellerer til barn, slik som tegneseriefigurer etc. (51). Figur 3 viser eksempel på en utstilling som ble felt av MFU.

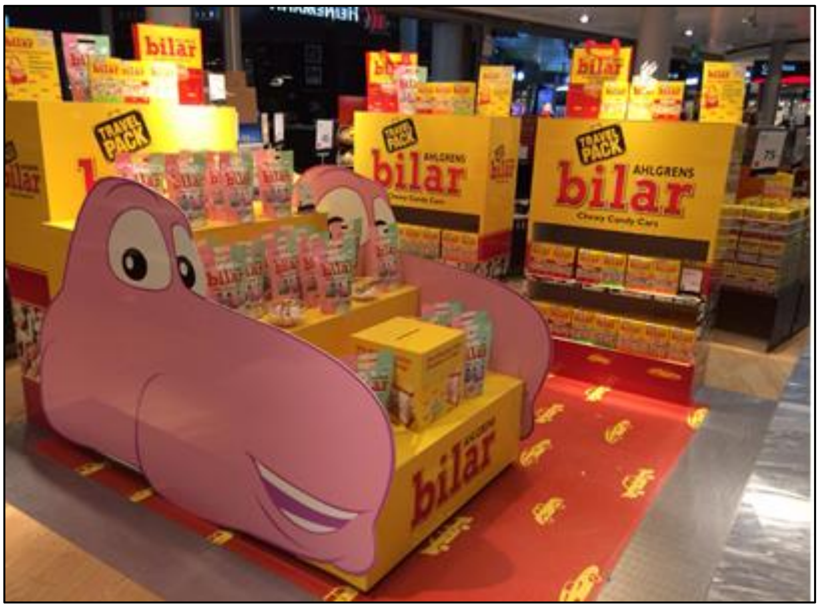

Figur 3. Eksempel på markedsføring som har blitt felt i MFU. Bildet er hentet fra MFU-vedtak 1 2015: http://www.mfu.as/44500-Vedtak-2015

MARK 4 – regler for markedsføring der barn samles

<u>**God praksis-beskrivelse**</u><br>**Myndighetene har implementert effektive ordninger for å sikre at usunn mat ikke markedsføres til barn og ungdom i situasjoner eller steder der barn og unge samles (for eksempel barnehager, skoler, sports- og kulturarrangementer).**	
**Definisjoner og omfang**	• «Situasjoner eller steder der barn og unge samles» omfatter: Områder i og rundt skoler, barnehager, barnehelsetjenestene (inkludert primærhelsetjeneste, mødre- og barnehelse og spesialisthelsetjenesten), idrett, rekreasjon og lekeområder, -arenaer og –fasiliteter samt kultur- eller samfunnsarrangementer der barn ofte er til stede.<br>• Omfatter restriksjoner på markedsføring i statlig eide eller forvaltede fasiliteter/arenaer.<br>• Omfatter restriksjon på sponsing som fremmer usunn mat i idrett (for eksempel i barneidrett, på sportsarrangementer, i idrettshaller og på idrettsplasser).<br>• «Effektiv» betyr at ordningene kan ventes å redusere barn og ungdoms totale eksponering av markedsføring for mat og drikke.
**Internasjonale eksempler på god praksis (referansemål)**	**Chile:** In June 2015, the Chilean authority approved the regulatory norms required for the Law of Nutritional Composition of Food and advertisings implementation. The regulatory norms define limits for calories, saturated fat, sugar and sodium content considered "high" in foods and beverages. The law restricts advertising directed to children under the age of 14 of foods in the "high in" category on school grounds, including preschools, primary and secondary schools. Chile has also restricted outdoor advertising, with ten municipalities adopting legislations banning outdoor marketing one block around schools (69).<br><br>**Uruguay:** In September 2013, the government of Uruguay adopted Law No 19.140 (Healthy foods in schools). The law prohibits the advertising and marketing of foods and drinks that do not meet the nutrition standards. Advertising in all forms is prohibited, including posters, billboards, and use of logos/brands on school supplies, sponsorship, and distribution of prizes, free samples on school premises and the display and visibility of food. The implementation of the law started in 2015 (55).<br><br>**Hungary:** Based on Section 8 of Act XLVIII on Basic Requirements and Certain Restrictions of Commercial Advertising Activities (2008), Hungary prohibits all advertising directed at children under 18 in child welfare and child protection institutes, kindergartens, elementary schools and their dormitories. Health promotion and prevention activities in schools may only involve external organizations and consultants who are recommended by the National Institute for Health Development (55).

	**Spain:** In 2011 the Spanish Parliament approved a Law on Nutrition and Food Safety, which stated that kindergartens and schools should be free from all advertising. Criteria for the authorisation of food promotion campaigns, nutritional education and promotion of sports or physical activity campaigns were developed jointly by the Spanish Agency for Consumer Affairs, Food Safety and Nutrition (AECOSAN) and the Regional Health Authorities and implemented in July 2015. AECOSAN and the Spanish Regional Education and Health Administrations monitor the enforcement of the law (55).
**Kontekst**<br>for eksempel EU-reguleringer, tiltak fra matindustrien etc.	Forbrukertilsynet sendte i 2019 ut et orienteringsbrev til grunnskoler om reklame i skolen (70). Her kommer det fram at Forbrukertilsynet jevnlig mottar klager om at barn utsettes for reklame gjennom skolens undervisningsmateriell, områder eller arrangementer. Brevet gjør oppmerksom på at det kan forekomme skjult reklame i skolen for eksempel gjennom materiell fra næringsdrivende som benyttes i undervisningen.

Evidens for implementering

Lover og forskrifter
I Norge er det lovfestet at skolen skal være fri for reklame. Opplæringslova og friskolelova (71, 72) legger ansvaret på skoleeier for å skjerme barn og ungdom mot reklame på skolen og i forbindelse med undervisning. Kravet er utdypet i Faglige retningslinje - Mat og måltider i skolen (73). Retningslinjene omfatter barneskole, ungdomsskole og videregående skole. Det påpekes at skolen har plikt til «å sørge for at elevene ikke utsettes for reklame som er egnet til å skape kommersielt press eller som i stor grad kan påvirke holdninger, adferd og verdier» (73). Reklame tolkes i vid forstand og omfatter både reklame, sponsing og andre salgsfremmende tiltak. Det finnes ikke en tilsvarende bestemmelse i barnehageloven, hvilket betyr at barnehagen ikke er reklamefri ved lov som skolen.

Reklame i nærområdet rundt skoler er ikke lovregulert i Norge, utover de generelle reglene om markedsføring i markedsføringsloven (65).

Frivillige ordninger
Skole: MFU slår fast at skolen ikke skal være en arena for reklame (51).

Når det gjelder idrett- og andre arrangementer har MFU følgende retningslinjer:

- Sponsing som kun innebærer bruk av sponsors navn og/eller sponsors eller et produkts varemerke er *unntatt* fra definisjonen av markedsføring og dermed lovlig.
- Utdeling av vareprøver etter samtykke fra foreldre eller andre ansvarlige er også *unntatt* fra retningslinjene og dermed lovlig.

MARK 5 – regler for markedsføring på emballasje

<u>**God praksis-beskrivelse**</u><br>**Myndighetene har implementert effektive ordninger for å sikre at usunn mat ikke markedsføres til barn og ungdom gjennom matens emballasje.**	
**Definisjoner og omfang**	• Omfatter produktdesign og innpakning (for eksempel bruk av tegneserier og kjendiser, konkurranser etc.).<br>• «Effektiv» betyr at ordningene kan ventes å redusere barn og ungdoms totale eksponering av markedsføring for mat og drikke.
**Internasjonale eksempler på god praksis (referansemål)**	**Chile**, through Law Number 20.606, has passed a series of regulations on the advertising of processed foods high in calories, fat, sugar, or salt. These laws specifically seek to regulate companies with brands that target children through misleading advertising and the use of cartoon mascots on commercial packaging. Chile's National Consumer Service has determined that food labels may no longer feature cartoon mascots designed to appeal to children (74).
**Kontekst**<br>for eksempel EU-reguleringer, tiltak fra matindustrien etc.	WHOs anbefalinger for begrensning av markedsføring av usunn mat og drikke rettet mot barn omtaler markedsføring på emballasje som en spesifikk markedsføringsteknikk som myndigheter kan vurdere å legge begrensinger på (75). I en evalueringsrapport fra WHOs Europakontor om implementering av anbefalingene kommer det fram at denne typen markedsføring sjeldent er omfattet i nasjonale ordninger i Europa (76). På tidspunktet da dette evidensdokumentet ble ferdigstilt var det ikke funnet EU-regelverk som forhindrer nasjonale myndigheter å innføre slike begrensninger.

Evidens for implementering

Frivillige ordninger:
MFUs retningslinjer unntar selve produktet, herunder emballasjen, fra definisjonen av markedsføring. Dette innebærer at produktutforming, emballasje, innpakking etc. *i seg selv ikke anses som markedsføring* (51). Hvis emballasjen er av en slik karakter at produktet er underordnet, kan produktet likevel bryte med retningslinjene. Figur 4 viser to eksempler på emballasje som har fått ulike vedtak i MFU.

I hvilken grad produktet appellerer til barn vil være av betydning med tanke på hvordan produktet kan markedsføres. Selv om det er lov å bruke for eksempel tegneseriefigurer på en frokostblanding som faller inn under MFUs produktliste, vil mulighetene for å reklamere for produktet være begrenset ifølge MFU (51).

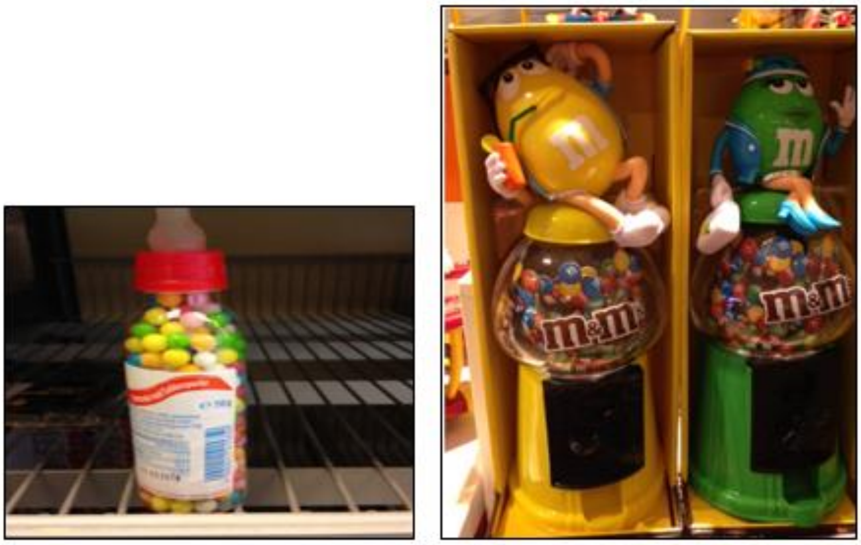

Figur 4. Emballasje til venstre regnes ikke som markedsføring av MFU. Emballasjen til høyre har derimot blitt felt i MFU fordi produktet vurderes som underordnet emballasjen. Bildene er hentet fra MFU-saker 7-2015 og 12-2016 http://www.mfu.as/44514-Vedtak

OMRÅDE 4: MATPRISER

Virkemidler for prising av matvarer (for eksempel skatter og subsidier) er i tråd med offentlige mål og anbefalinger for kosthold ved å bidra til å gjøre det enklere og billigere å velge sunt.

Kommentar: Det er i utgangspunktet fire indikatorer for dette området. Indikator PRIS 4 er imidlertid ikke relevant i Norge, og skal derfor ikke vurderes i Nettskjema. Indikatoren er tatt med kun som informasjon.

- PRIS 1 – lave avgifter på sunne matvarer
- PRIS 2 – avgifter på usunne matvarer
- PRIS 3 – subsidier favoriserer sunne matvarer
- PRIS 4 – matrelaterte støtteordninger for sunne matvarer

PRIS 1 – lave avgifter på sunne matvarer

<table>
<tr><td colspan="2"><u>God praksis-beskrivelse</u>
Skatter eller avgifter på sunne matvarer er minimert for å oppmuntre til sunne valg der det er mulig (f.eks. lav eller ingen merverdiavgift, omsetningsavgift eller toll på frukt og grønnsaker).</td></tr>
<tr><td>Definisjoner og omfang</td><td>• Omfatter unntak fra særavgifter, merverdiavgift eller importavgift.
• Omfatter differensiering av særavgifter, merverdiavgift eller importavgift.
• Unntatt subsidier (se PRIS 3) eller inntektsstøtte til matinnkjøp (se PRIS 4).</td></tr>
<tr><td>Internasjonale eksempler på god praksis (referansemål)</td><td>Australia: Goods and services tax (GST) exemption exists for basic foods (including fresh fruits and vegetables) (77).

Tonga: In 2013, as part of a broader package of fiscal measures, import duties were lowered from 20% to 5% for imported fresh, tinned or frozen fish in order to increase affordability and promote healthier diets (78).</td></tr>
</table>

Evidens for implementering

Gjeldende prisvirkemidler

Det er redusert merverdiavgift på næringsmidler (15 % i stedet for 25 %), med unntak av for mat som omsettes som del av en serveringstjeneste, med mindre den ikke skal spises på serveringstedet. Helse og kosthold er ikke inkludert i kriteriene for redusert merverdiavgift på matvarer og det er ingen differensiering mellom ulike kategorier. Tradisjonelle kioskvarer (sjokolade, pastiller, sukkertøy, frukt og emballert is) skal alltid selges med redusert takst, selv om de selges på et serveringssted og spises på stedet. Kriteriene er gitt i merverdiavgiftsloven (79).

Foreslåtte tiltak

På oppdrag fra Helse- og omsorgsdepartementet la Helsedirektoratet i 2018 fram dokumentet *Ti tiltak for å redusere sykdomsbyrden og bedre folkehelsen*, som skal inngå i beslutningsgrunnlaget for en ny NCD-strategi. Ett av ti foreslåtte tiltak var «sunn skatteveksling og gratis skolemåltid». Her foreslås det å innføre en gradert avgift på tilsatt sukker og samtidig prisreduksjon på frukt, bær og grønnsaker (80).

I 2019 sendte Finansdepartementet *NOU 2019:11 Enklere merverdiavgift med én sats* på høring. Utredningen gir en vurdering av omlegging av merverdiavgifts-systemet slik at det får færre satser (fra dagens 12, 15 og 25 % i tillegg til nullsatser). Utvalget anbefaler en omlegging til kun én sats – 25 % – også for næringsmidler, det vil si en økning av merverdiavgiften for matvarer fra 15 til 25 %. Utvalget anser ikke at merverdiavgiften er et egnet virkemiddel for å stimulere til et helsebringende kosthold (81).

I sitt høringssvar skriver Helsedirektoratet at differensiering av mva-satser for næringsmidler kan og bør brukes som strukturelt virkemiddel for å bedre folkehelsen. Direktoratet anbefaler nullsats for næringsmidler som kan påvirke folkehelsen positivt, en forhøyet sats på 25% for usunne næringsmidler, mens 15 % satsen kan beholdes for andre matvarer (82).

PRIS 2 – avgifter på usunne matvarer

**<u>God praksis-beskrivelse</u>**<br>**Skatter eller avgifter på usunn mat (for eksempel sukkerholdige drikkevarer og mat som har høyt innhold av næringsstoffer som bør begrenses) er implementert, slik at utsalgsprisene på disse matvarene øker med minst 10% for å motvirke usunne valg der det er mulig, og disse skattene reinvesteres til å forbedre befolkningens helse.**	
**Definisjoner og omfang**	Omfatter differensiert anvendelse av særavgifter, merverdiavgift eller importavgift på energitett mat eller mat som har høyt innhold av næringsstoffer som bør begrenses.
**Internasjonale eksempler på god praksis (referansemål)**	**Ireland:** In May 2018, the Republic of Ireland's Sugar Sweetened Drinks Tax came into force under the Finance Act 2017 (No. 41 of 2017). The tax applies to non-alcoholic, water-based and juice based drinks which have added sugar content of 5g per 100mL and above. Drinks with over 8g of sugar per 100mL are taxed at 30 cents per litre, and drinks with between 5g and 8g of sugar per 100mL are taxed at 20 cents per litre. Fruit juices and dairy products are excluded from the tax (78).<br><br>**UK:** In April 2018 the UK government's Soft Drinks Industry Levy came into force (as outlined in the Finance Act 2017). The Soft Drink Industry Levy applies to any pre-packaged soft drink with added sugar, containing at least 5g of total sugars per 100mL of prepared drink. Soft drinks that have a total sugar content of more than 5g and less than 8g per 100mL are taxed 0.18 British pounds ($0.25) per litre and drinks that have a total sugar content of 8g or more per 100mL are taxed 0.24 British pounds ($0.34) per litre. Milk-based drinks, milk substitute drinks, pure fruit juices, or any other drinks with no added sugar, alcohol substitute drinks, and soft drinks of a specified description which are for use for medicinal or other specified purposes are exempt from the levy. The levy applies to soft drinks produced and packaged in the UK and soft drinks imported into the UK (78). Manufacturers had two years to prepare ahead of this tax coming into effect and over 50% of them took action to cut sugar in their products during that period (83). It was forecasted that the tax would bring in £520 million in its first year of operation, but this was revised down to £275 million as a result of company efforts to remove sugar from their products. Data from the first full year of the tax is not yet available, but receipts from April to October 2018 totalled £154 million (84).<br><br>**Hungary:** A "public health tax" adopted in 2012 is applied on the salt, sugar and caffeine content of various categories of ready-to-eat foods, including soft drinks, energy drinks, and pre-packaged sugar-sweetened products. The tax is applied at varying rates. Soft drinks, for example, are taxed at $0.24 per litre and other sweetened products at $0.47 per litre. The tax also applies to products high in salt, including salty snacks with >1g salt per 100g, condiments with >5g salt per 100g and flavourings >15g salt per 100g (78, 85).

	**Mexico:** In December 2013, the Mexican legislature passed two new taxes as part of the national strategy for the prevention of overweight, obesity and diabetes. An excise duty of 1 peso ($0.80) per litre applies to sugary drinks. This is expected to increase the price of sugary drinks by around 10%. An ad valorem excise duty of 8% applies to foods with high caloric density, defined as equal to or more than 275 calories per 100 grams. The taxes entered into force on 1 January 2014. The aim is for the revenue of taxes to be reinvested in population health, namely providing safe drinking water in schools, but there is no evidence (yet) that this is the case as the taxes are not earmarked (78, 86). In 2019, a study was conducted to estimate changes in taxed and untaxed beverages by volume of beverage purchased after the sugar-sweetened beverage (SSB) tax was introduced in 2014. Households with lower purchases of untaxed beverages had the largest absolute and relative increases in untaxed beverages. Furthermore, among households with higher purchases of taxed beverages, the group with lowest socio-economic status had the greatest reduction in purchases of taxed beverages (87).<br><br>**Qatar:** In 2018, the Government of Qatar introduced Law No. 25, the 'Qatar Excise Tax Law' that came into effect on 1 January 2019. The Qatar Excise Tax Law introduced a 50% ad valorem tax on carbonated waters with added sugar, sweeteners or flavours, as well as concentrates, powders, gels or extracts intended to be made into a carbonated beverage. A tax rate of 100% is applied to beverages sold as energy drinks that contain stimulant substances (e.g. caffeine, taurine, ginseng, guarana). Carbonated non-flavoured waters, coffee and tea are excluded from the excise tax. The excise tax applies to all imported, produced or stockpiled aerated beverages (except unflavoured aerated water) and energy drinks (78).

Evidens for implementering

Gjeldende prisvirkemidler

I Norge er det to ulike avgifter på sukkerholdige varer som anses som relevante i en helsesammenheng: Avgifter på sjokolade- og sukkervarer (innført 1922) og avgift på alkoholfrie drikkevarer (opprinnelig innført 1924). Begge avgiftene er fiskale og ikke helsemessig begrunnet, men det fremheves også at avgiftene har også helsemessige egenskaper ved at de kan bidra til å redusere forbruket av sjokolade- og sukkervarer og alkoholfrie drikkevarer (88).

I forbindelse med statsbudsjettet for 2018 ble avgiften på sjokolade- og sukkervarer og avgiften på alkoholfrie drikkevarer økt med henholdsvis 80 og 40 % for å skaffe inntekter til statskassen. Som respons på at matvarebransjen reagerte negativ på avgiftsøkningen foreslo regjeringen i budsjettet for 2019 å sette avgiften tilbake til 2017-nivå, bare prisregulert (89). Finansdepartementet (FIN) satte ned et utvalg i november 2018 for å vurdere om avgiftene kan utformes slik at de i større grad ivaretar helse- og ernæringsformål. Utvalgets rapport ble overlevert FIN i april 2019 (88). Utvalget konkluderte ikke entydig, og flere utforminger av sukkeravgiften ble diskutert. Utvalgets flertall mente imidlertid at avgiftene slik de er utformet i dag bør oppheves og erstattes av nye, helsebegrunnede avgifter (88). NOU'en ble sendt på høring i april, med høringsfrist juli 2019.

Høringsinstansene var ikke samstemte (90). I statsbudsjettet 2020 ble begge avgiftene videreført og prisjustert (91).

Foreslåtte tiltak
I 2019 sendte Finansdepartementet *NOU 2019:11 Enklere merverdiavgift med én sats* på høring. Utredningen gir en vurdering av omlegging av merverdiavgifts-systemet slik at det får færre satser (fra dagens 12, 15 og 25 % i tillegg til nullsatser). Utvalget anbefaler en omlegging til kun én sats – 25 % – også for næringsmidler, det vil si en økning av merverdiavgiften for matvarer fra 15 til 25 %. Utvalget anser ikke at merverdiavgiften er et egnet virkemiddel for å stimulere til et helsebringende kosthold (81).

I sitt høringssvar skriver Helsedirektoratet at differensiering av mva-satser for næringsmidler kan og bør brukes som strukturelt virkemiddel for å bedre folkehelsen. Direktoratet anbefaler nullsats for næringsmidler som kan påvirke folkehelsen positivt, en forhøyet sats på 25% for usunne næringsmidler, mens 15 % satsen kan beholdes for andre matvarer (82).

PRIS 3 – subsidier favoriserer sunne matvarer

**God praksis-beskrivelse**<br>**Formålet med eksisterende subsidier på matvarer inkludert finansieringsstøtte til infrastruktur (for eksempel forskning og utvikling, støtte til markeder eller transportsystemer), er å favorisere sunn og ikke usunn mat.**	
**Definisjoner og omfang**	• Inkluderer landbrukssubsidier, for eksempel gratis eller subsidierte kostnader for vann, gjødsel, frø, elektrisitet eller transport (f.eks. frakt) der disse subsidiene er spesifikt rettet mot sunn mat.<br>• Inkluderer programmer som sikrer at bønder får en viss pris for sine produkter for å oppmuntre til økt matproduksjon eller økonomisk overlevelse.<br>• Inkluderer tilskudd eller støtte til matvareprodusenter (dvs. bønder, matprodusenter) for å oppmuntre til innovasjon gjennom forskning og utvikling når finansieringsordningen spesielt retter seg mot sunn mat.<br>• Inkluderer finansieringsstøtte til grossistsystemer som støtter forsyning/tilbud av sunne matvarer.<br>• Inkluderer matvaresubsidier på populasjonsnivå ved forbrukersiden (for eksempel subsidiering av basismatvarer som ris eller brød).<br>--------------------<br>• Inkluderer ikke insentiver for etablering av, eller kontinuerlig støtte til, handelen (inkludert grønnsakshandlere, bondens marked, matvaresamvirker etc. Se 'HANDEL 2').<br>• Ekskluderer subsidiert opplæring, kurs eller annen utdanning for matprodusenter.<br>• Ekskluderer omfordeling av overskuddslagre eller av sekunda vare<br>• Ekskluderer støtte knyttet til inntekt (se 'PRIS 4').<br>-------------------<br>• Bør være i tråd med offentlige mål for kosthold og ernæring relatert til forebygging av overvekt og NCDs (for eksempel å redusere inntak av næringsstoffer som bør begrenses), og bør ikke være relatert til mangel på mikronæringsstoffer.
**Internasjonale eksempler på god praksis (referansemål)**	**Singapore:** The government, through the Health Promotion Board (HPB) increases the availability and use of healthier ingredients through the “Healthier Ingredient Scheme” (formerly part of the "Healthier Hawker" programme, launched in 2011), which provides in the first instance transitional support to oil manufacturers and importers to help them increase the sale of healthier oils to the food service industry. The Healthier Ingredient Subsidy Scheme offers a subsidy to suppliers stocking healthier items. Cooking oil is the first ingredient under the scheme, which subsidises oils with a saturated fat level of 35 per cent or lower (92).

Evidens for implementering

Regelverk og subsidier på matvarer
Forskrift om tilskudd til frukt i skolen (2004) har som formål «å stimulere til et økt forbruk av frukt og grønnsaker ved å tilby alle elever i grunnskolen en frukt eller grønnsak alle skoledager» (93). Tilskuddssatsen fastsettes for to år av gangen, av Helsedirektoratet. Inneværende år er foreldrebetalingen satt til kr. 3,20 per dag og det offentlige subsidierer hver frukt/grønnsak med kr. 1,50.

Det er åpnet for at kommuner kan gi skoleelevene gratis frukt, og kommunen får da kjøpe subsidiert frukt og grønt (94). I 2017 fikk ca. 64 000 barn subsidiert skolefrukt. I Norge er det i overkant av 450 000 barn i alderen 6-12 år (95).

Hensikten i omsetningsloven (96) er å øke omsetningen av landbruksprodukter gjennom å ta inn avgifter som brukes til tiltak for å regulere produksjonen for å sikre stabile priser, og til å øke forbruket gjennom samarbeid. Det er ingen øremerking av midler til å fremme produksjon av sunne matvarer. En andel av avgiftene som tas inn går til skolemelk-ordningen, gjennom fondet for omsetningsavgift for melk. Andre deler av avgiftene som kreves inn etter omsetningsloven går til faglige tiltak og generisk opplysningsarbeid og til finansiering av opplysningskontorene. Formålet med opplysningskontorene er å øke forbruket av norske landbruksprodukter (97).

PRIS 4 – matrelaterte støtteordninger for sunne matvarer

Følgende indikator er ikke relevant for Norge og skal ikke vurderes i Nettskjema. Følgende tekst er derfor bare til informasjon.

**God praksis-beskrivelse**<br>**Myndighetene sikrer at matrelaterte støtteordninger er for sunne matvarer.**	
**Definisjoner og omfang**	• Omfatter programmer som "matmerker" eller andre ordninger der enkeltpersoner kan benytte statlig administrerte subsidier, kuponger, eller rabatter for matinnkjøp i dagligvarehandelen.<br>------------------<br>• Omfatter ikke generelle programmer eller tiltak for å sikre matsikkerhet, for eksempel statlig støtte til organisasjoner som tilbyr gratis eller subsidierte måltider (inkludert skolefrokost-programmer) eller distribusjon av overskuddsvarer til dette formålet (f.eks. best før-produkter, overskuddsmat fra butikker, Matvaresentralen etc.).<br>• Omfatter ikke subsidier til forbruker-siden, for eksempel subsidier til basismatvarer på befolkningsnivå - se PRIS 3.
**Internasjonale eksempler på god praksis (referansemål)**	**USA:** In 2009, the U.S. Department of Agriculture's implemented revisions to the Special Supplemental Nutrition Program for Women, Infants, and Children (WIC) to improve the composition and quantities of WIC-provided foods from a health perspective. The revisions include: Increase the dollar amount for purchases of fruits and vegetables, expand whole-grain options, allow for yoghurt as a partial milk substitute, allow parents of older infants to buy fresh produce instead of jarred infant food (78).<br><br>**USA:** In 2012, the USDA piloted a "Healthy Incentives Pilot" as part of the Supplemental Nutrition Assistance Program (SNAP, formerly "food stamps"). Participants received an incentive of 30 cents per US$ spent on targeted fruit and vegetables (78). In New York City and Philadelphia, “Health Bucks” are distributed to farmer’s markets. When customers use income support (e.g. Food Stamps) to purchase food at farmer’s markets, they receive one Health Buck worth 2USD for each 5USD spent, which can then be used to purchase fresh fruit and vegetable products at a farmer’s market (78).<br><br>**UK:** The British Healthy Start programme provides pregnant women and/or families with children under the age of four with weekly vouchers to spend on foods including milk, plain yoghurt, and fresh and frozen fruit and vegetables. Participants or their family must be receiving income support/jobseekers’ allowance or child tax credits. Pregnant women under the age of 18 can also apply. Full national implementation of the programme began in 2006 (78).

Evidens for implementering

Det ligger ingen premisser om at støtte til livsopphold skal brukes til sunne matvarer i Statlige veiledende retningslinjer for økonomisk stønad for 2019 (98).

OMRÅDE 5: MATOMSORG OG -TILBUD

Myndighetene sørger for at det er implementert sunn måltidspolitikk i offentlig finansierte steder og sammenhenger slik at mattilbud bidrar til sunne valg, og oppmuntrer aktivt bedrifter til å implementere lignende retningslinjer.

Det er fem indikatorer for dette området:

- MATO 1 – retningslinjer i skoler og barnehager
- MATO 2 – retningslinjer i andre offentlige sammenhenger
- MATO 3 – retningslinjer for matanskaffelse i offentlig sektor
- MATO 4 – støtte- og opplæringssystemer for offentlig sektor
- MATO 5 – støtte- og opplæringssystemer for private bedrifter

Felles for alle indikatorene i dette området – informasjon som skal tas i betraktning i vurderingen av norsk politikk:

Nasjonal handlingsplan for bedre kosthold 2017-2021 vektlegger betydningen felles måltider har for alle mennesker uavhengig av livsfase og helse (99). Det fremheves også at det er viktig at barn og unge utvikler et bredt spekter av smaksreferanser i ung alder, slik at de blir inspirert til å spise mer variert. Handlingsplanen peker på flere aktuelle tiltak innenfor matomsorg:

- fremme gode mat- og måltidsvaner i barnehagen
- fremme gode måltider og matordninger i skole og skolefritidsordningen
- motivere til matglede og bedre matomsorg for eldre
- ernæringshensyn i anbud i helseforetak og andre offentlige instanser
- kompetanse om mat, måltider og ernæring i helse- og omsorgstjenesten
- innføre selvforpleining og utarbeide kokebok som støtte til sunnere mat i fengsler.

MATO 1 – retningslinjer i skoler og barnehager

<table>
<tr><td colspan="2"><u>God praksis-beskrivelse</u>
Myndighetene sikrer at klare, konsekvente retningslinjer (inkludert ernæringskriterier) er implementert for mattilbudet/servering i skole og barnehage (kantiner, på dugnadsaktiviteter, mat på arrangementer, i salgsautomater etc.) for å tilby og fremme sunne matvalg.</td></tr>
<tr><td>Definisjoner og omfang</td><td>• Omfatter offentlige og private barnehager, barne- og ungdomsskoler og videregående skoler.
• Inkluderer retningslinjer og ernærings-standarder for å tilby og fremme sunne matvalg eller for å begrense tilbudet eller markedsføringen av usunn mat.
• Inkluderer retningslinjer for skolefrokostprogrammer, der programmet er helt eller delvis finansiert av myndighetene.

• Omfatter ikke utdanning, kursing og ressurser som støtter implementeringen av retningslinjene over (se MATO 4).</td></tr>
<tr><td>Internasjonale eksempler på god praksis (referansemål)</td><td>Ireland: The School Meals (Local Projects) Scheme is an administrative scheme operated by the Department of Employment Affairs and Social Protection (100). The Scheme provides funding to primary and post-primary schools, local groups, voluntary organisations and community-based not-for-profit preschools operating their own school meals projects. The ‘Nutrition Standards for School Meals’, are being implemented under this scheme and aim to ensure that children and young people in schools participating in the scheme are provided with healthy balanced meals that follow the Healthy Eating Guidelines. These Nutrition Standards are food-based, and are provided for each meal type funded by the Scheme, that is: Breakfast or snack Lunch or after-school meal Dinner, only healthy food choices that meet the standards will be funded. The Standards will also be used by those administering the Scheme in the schools, commencing in January 2018, to ensure that food purchased complies with the Nutrition Standards when food contracts are being specified in the procurement process, and should also be applied when planning menus (100).

Chile: In 2012, the Chilean government approved a Law of Nutritional Composition of Food and Advertising (101). In June 2015, the Chilean authority approved the regulatory norms required for the law’s implementation. The regulatory norms define limits for calories, saturated fat, sugar and sodium content considered ‘high’ in foods and beverages. The law prohibits the sale of foods in the ‘high in’ category in schools. The law came into effect in June, 2016. In 2019, a study conducted on the impact of this law finds that, foods exceeding any cut-offs decreased from 90.4% in 2014 to 15.0% in 2016. Solid products had a substantial reduction in calories, sugar, saturated fat, and sodium. Liquid products had a reduction in calories, total sugar, and saturated fat, whereas sodium increased. This was a result of changes in product mix (102).</td></tr>
</table>

	**Finland:** In 2008, the National Nutrition Council approved nutrition recommendations for school meals. These include food and nutrient recommendations for salt, fibre, fat, starch, fat and salt maximums for meat and processed meat, and drinks. There are also criteria for snacks provided in schools. New recommendations on Eating and learning together - recommendations for school meals have been published in 2017 (103). In 2018, the early childhood education: Health and joy from food - meal recommendations for early childhood education and care, were published (104). Additionally, Finland published its first nutrition recommendations for upper secondary schools and vocational schools.<br><br>**UK:** England, Scotland, Wales and Northern Ireland have mandatory nutritional standards for school food, which also apply to food provided in schools other than school lunches. These standards apply to most state schools (with the exception of around 4,000 academies established between September 2010 and June 2014, which are exempt) and restrict foods high in fat, salt and sugar, as well as low quality reformed or reconstituted foods (105).<br><br>**Brazil:** The national school feeding programme mandates a weekly minimum of fruits and vegetables, regulates sodium content and restricts the availability of sweets in school meals (101). A school food procurement law, approved in 2013, limits the amount of processed foods purchased by schools to 30%, and bans the procurement of drinks with low nutritional value, such as sugary drinks (106). The law requires schools to buy locally grown or manufactured products, supporting small farmers and stimulating the local economy. Resolution no 38 (16 July 2009) sets food- and nutrition-based standards for the foods available in the national school meal programme (Law 11.947/2009). Article 17 prohibits drinks of low nutritional value (e.g. soda), canned meats, confectionary and processed foods with a sodium and saturated fat content higher than a specified threshold.<br><br>**Uruguay:** In September 2013, the government of Uruguay adopted Law No 19.140 on “healthy eating in schools”. It mandated the Ministry of Health to develop standards for food available in canteens and kiosks in schools, prohibited advertising for these same foods and restricted the availability of saltshakers. The school food standards were elaborated in March 2014 and aimed to promote foods with natural nutritional value with a minimum degree of processing and to limit the intake of free sugars, saturated fat, trans fat and sodium. Limits are set per 100g of food, 100ml for drinks and also per 50g portion. This was implemented in public schools in 2015 (107).

Evidens for implementering

Regelverk og retningslinjer for barnehage
Forskrift om rammeplan for barnehagens innhold og oppgaver (108) sier at «barnehagen skal legge til rette for at alle barn kan oppleve matglede og matkultur». Rammeplanen fokuserer på at barns medvirkning i mat- og måltidsaktiviteter skal motivere barna til å spise sunt og å lære sammenhengen mellom sunt kosthold og helse, slik at de tar med seg gode vaner for hygiene og et sunt og variert kosthold. Det skal *«legges til rette for at måltider og matlaging bidrar til måltidsglede, samtaler og fellesskapsfølelse».*

I 2018 ble det publisert en ny *nasjonal retningslinje for mat og måltider i barnehagen*. Retningslinjen er faglig normerende og ikke rettslig bindende. Anbefalingene i retningslinjen henviser til å følge Helsedirektoratets kostholdsråd og at ernæringskriterier bør ligge til grunn, både ved anbud og ved løpende innkjøp. Bruk av Nøkkelhullet og brødskalaen anbefales (109).

Regelverk og retningslinjer for skole
Opplæringsloven (71) stiller krav om et godt fysisk og psykososialt skolemiljø som fremmer helse, trivsel og læring. Den sier ikke noe spesifikt om mat eller måltider. Det gjør heller ikke *folkehelseloven* (110), men den stiller krav til skoleeier (kommune, fylkeskommune, private skoleeiere) om å fremme helse og trivsel, samt bidra til å utjevne sosiale ulikheter.

Helsedirektoratet oppdaterte de *nasjonale retningslinjene for mat og måltider i skolen* i 2015 (73). Retningslinjene er tredelt og omfatter grunnskole og SFO, ungdomsskole og videregående skole. En av begrunnelsene for revisjonen er at de forrige retningslinjene var basert på at elevene har med matpakke, mens stadig flere SFO nå har begynt å servere mat. Retningslinjene er likevel basert på at de fleste har medbragt matpakke, og at skolen skal formidle til elever og foresatte hva en sunn og god matpakke er. Nytt i revidert retningslinje er anbefalinger om gjennomføring av måltid med spisetid, tilsyn og fysisk og sosial tilrettelegging, samt anbefalinger for den ernæringsmessige kvaliteten på mat og drikke som tilbys, mattrygghet, hygiene og miljøhensyn. Målet for retningslinjene er å bidra til at elevene sikres gode rammer for måltidene og god ernæringsmessig kvalitet på mat og drikketilbudet, og er ment som et verktøy som kan bidra til å redusere sosiale forskjeller i levevaner. Det er ikke noe krav om å tilby mat, men å tilrettelegge. Det oppfordres også til å tilby enkel mat til elever som unntaksvis mangler matpakke eller penger. Til grunn for anbefalingene ligger de offisielle anbefalingene for kosthold, ernæring og fysisk aktivitet (73).

I tillegg til anbefalinger om rammene rundt måltidet mht. tid, hygiene, tilsyn etc. anbefales det at *elevene bør tilbys ordninger som sikrer tilgang til grønnsaker, frukt eller bær daglig* og at *elevene bør tilbys ordninger som sikrer tilgang til skummet melk, lettmelk med 0,7 prosent fett eller mindre eller lettmelk til måltidene.*

Abonnements-ordningen med skolefrukt benyttes av om lag 10% av elevene. Omtrent en tredjedel av disse fikk gratis skolefrukt gjennom kommunale eller andre lokale ordninger (111). Skolemelk er en abonnementsordning betalt av foreldre og subsidiert igjennom omsetningsavgiften. Bestillingsportalen drives av Tine.

Andre dokumenter som nevner skolemat:
Helsedirektoratets dokument *ti tiltak for å redusere sykdomsbyrden og bedre folkehelsen* ble overlevert Helse- og omsorgsdepartementet i september 2018. Leveransen skal inngå som en del av departementets beslutningsgrunnlag for ny NCD-strategi. En av anbefalingene i rapporten er å innføre gratis frukt og grønt til alle elever i grunnskolen, og en pilotering av gratis skolefrokost i grunnskole og VGO med tiltaksevaluering (80).

Skolemåltid var en viktig sak i *kommunevalgkampen* høsten 2019. På rødgrønn side var det støtte for et nasjonalt regelverk som skulle pålegge kommunene å servere gratis skolemåltid. På blåblå side var det motstand mot nasjonale ordninger, men åpning for at kommuner selv kan tilrettelegge for ulike ordninger med skolemåltid basert på lokalt behov (112).

MATO 2 – retningslinjer i andre offentlige sammenhenger

<table>
<tr><td colspan="2"><u>God praksis-beskrivelse</u>
Myndighetene sikrer at det er klare, konsekvente retningslinjer for mattilbud i andre offentlige sammenhenger (kantiner, arrangementer, automater) for å fremme sunne matvalg.</td></tr>
<tr><td>Definisjoner og omfang</td><td>"Andre offentlige sammenhenger" omfatter:
• Offentlige finansierte eller forvaltede tjenester der staten har ansvaret for levering av mat, inkludert offentlige sykehus og andre pasienthelsetjenester (akutt og subakutt, inkludert psykiske helsetjenester), alders- og sykehjem, rehabiliteringshjem, boliger for funksjonshemmede, omsorgstjenester, fengsler og hjemmetjenesten. Indikatoren omfatter imidlertid ikke mat som serveres til innlagte pasienter.
• Offentlige eide, finansierte eller administrerte tjenester der allmennheten kan kjøpe matvarer, inkludert helsetjenester, parker, idrettsanlegg og fritidsfasiliteter, lokale tilstelninger etc.
• Arbeidsplasser innen offentlig sektor.
• Omfatter private selskaper som tilbyr mat gjennom kontrakt med det offentlige.
• Inkluderer retningslinjer og ernærings-standarder for å tilby og fremme sunne matvalg eller for å begrense tilbudet eller markedsføringen av usunn mat.
• Inkluderer strategisk plassering av mat og drikke i skap, kjøleskap, på hyller eller nær betalingspunktet.
• Inkluderer bruk av skilting for å fremme sunne valg (trafikklys eller godkjente sunnhetsmerker).
• Inkluderer å endre resepter for å gjøre mat og drikke sunnere, eller å endre menyen for å tilby sunnere valg.

• Ekskluderer steder eller sammenhenger som ikke er finansiert eller administrert av myndighetene (togstasjoner, tilstelninger etc.) - se HANDEL 4
• Ekskluderer skoler og barnehager (se MATO 1)
• Omfatter ikke retningslinjer for offentlig anskaffelse (se MATO 3)</td></tr>
<tr><td>Internasjonale eksempler på god praksis (referansemål)</td><td>Ireland: The Health Service Executive (HSE) Vending Policy 2019 applies to all vending machines that stock cold soft drinks, confectionery and snacks on HSE premises & premises funded by the HSE. Sugar sweetened beverages will not be stocked in vending machines, 50% of beverages stocked will be still water and the remaining beverages stocked will include non-sugar sweetened beverages e.g. diet drinks, juices, flavoured and sparkling water. Snacks containing more than 200 calories per packet will not be stocked in machines. An exception to this is 3 packets of dried fruits, nuts or seeds (plain and unsalted). Products will be clearly labelled with the number of calories per product related fields (113).
Bermuda: In 2008, the Government Vending Machine Policy was implemented in government offices and facilities to ensure access to healthy snacks and beverages for staff. The policy requires that all food</td></tr>
</table>

	and beverages in vending machines on government premises meet specific criteria based on levels of total fat, saturated fat, trans fat, sodium and sugar. Criteria exclude nuts and 100% fruit juices (107).<br><br>**New York:** New York City's Food Standards (enacted with Executive Order 122 of 2008) set nutritional standards for all food purchased or served by city agencies, which applies to prisons, hospitals and senior care centres. The Standards include: maximum and minimum levels of nutrients per serving; standards on specific food items (e.g. only no-fat or 1% fat milk); portion size requirements; the requirement that water be offered with food; a prohibition on the deep-frying of foods; and daily calorie and nutrient targets, including population-specific guidelines (e.g. children, seniors) As of 2015, 11 city agencies are subject to the NYC Food Standards, serving and selling almost 250 million meals a year. The Food Policy Coordinator has the responsibility of ensuring adherence with the Food Standards. Self-reported compliance with the standard is 96% (107, 114).<br><br>**Wales:** Vending machines with unhealthy foods are prohibited in National Health Service Hospitals. In 2008, the government issued a guidance defining what is allowed and not and has liaised with major vending providers to find ways to introduce healthier food options (115).<br><br>**The Netherlands:** The Netherlands Nutrition Centre introduced the 'Guidelines for Healthier Canteens', which can be applied in canteens at schools, sports clubs and workplaces to make them more healthy. The Guidelines for Healthier Canteens cover canteens at product level and at the level of the full range of food and drink being offered, together with the canteen's general display layout. The framework of the Guidelines for Healthier Canteens defines three different levels: bronze, silver and gold (116).

Evidens for implementering

Retningslinjer, veiledninger og andre ressurser

Helsedirektoratet har publisert «Nasjonale anbefalinger for mat og drikke i arbeidslivet» (117). Disse er rettet mot arbeidslivet uavhengig om det er offentlig eller privat sektor. Anbefalingene er basert på de generelle kostholdsrådene og inneholder eksempler på praktiske løsninger for å følge kostholdsrådene og legge til rette for sunne valg på arbeidsplasser med kantine, spiserom eller liknende. Blant annet finnes forslag til anbefalt grunnsortiment i kantiner eller for enklere servering, og bevertning ved møter. Det er også laget en «verktøykasse» som består av folder med anbefalingene, et hefte som utdyper anbefalingene og gir praktiske råd til gjennomføring, og en presentasjon med kommentarer (118).

Helse- og omsorgstjenesten

Helsedirektoratet har ansvar for å gi anbefalinger for kostholdet i helse- og omsorgstjenesten. Kosthåndboken er nasjonal veileder i ernæringsarbeid i helse- og omsorgstjenesten (119). Veilederen er ment for både friske og syke som bor i helseinstitusjon, men de omfatter ikke maten som serveres i kantiner etc. i disse institusjonene. Rådene anbefaler Nøkkelrådskosten til alle friske

og syke med god ernæringsstatus. Nøkkelrådskosten følger Helsedirektoratets generelle kostråd. I rapporten Ernæring i helse- og omsorgstjenesten. Status, utfordringer og eksempler på gode tiltak fra 2017 kommer det fram at det mangler kunnskap om i hvilken grad Kosthåndbokens føringer for standardkostholdet er implementert i helse- og omsorgsinstitusjoner i Norge (120).

Fengsel
I veilederen Helse- og omsorgstjenester til innsatte i fengsel, står det at de innsatte har krav på trygg mat og et ernæringsmessig fullverdig kosthold. Det forventes at fengsler og andre offentlig institusjoner utenfor helse- og omsorgssektoren gir et mattilbud i tråd med Kosthåndbokens anbefalinger (121).

Barnevernsinstitusjon
Nasjonal handlingsplan for bedre kosthold 2017-2021 viser til at Bufdir ferdigstiller en faglig anbefaling og et opplæringsprogram for barnevernsinstitusjoner, der kosthold vil omfattes av en anbefaling og være ett tema i en opplæringsmodul (99).

MATO 3 – retningslinjer for matanskaffelse i offentlig sektor

**<u>God praksis-beskrivelse</u>**<br>**Myndighetene sikrer at det er klare, konsekvente retningslinjer for offentlig innkjøp for mattilbud og -servering i offentlig sektor for å tilby og fremme sunne matvalg.**	
**Definisjoner og omfang**	• Omfatter standarder for offentlig sektor som oppfordrer til innkjøp av sunn mat.<br>• Omfatter standarder for offentlig sektor som fraråder innkjøp av usunn mat.<br>• Omfatter barnehager, skoler og offentlige sammenhenger som definert i MATO 1 og 2.
**Internasjonale eksempler på god praksis (referansemål)**	**Brazil:** A school food procurement law, approved in 2013, limits the amount of processed foods purchased by schools to 30%, and bans the procurement of drinks with low nutritional value, such as sugary drinks. The law requires schools to buy locally grown or manufactured products, supporting small farmers and stimulating the local economy (106).<br><br>**UK:** The UK Government Buying Standard for Food and Catering Services sets out standards for the public sector when buying food and catering services. It is supported by the Plan for Public Procurement: Food and Catering Services (2014). The nutrition requirements must be followed by schools, hospitals, care homes, communities and the armed forces. To improve diets, the GBSF sets maximum levels for sugar in cereals and generally for saturated fat and salt, in addition to minimum content of fibre in cereals and fruit in desserts. In 2019, a public consultation was carried out regarding revision of the nutrition standards (122).

Evidens for implementering

Veileder– ernæringskrav- og kriterier i offentlige anskaffelser
Med forankring i Nasjonal handlingsplan for bedre kosthold har Helsedirektoratet utarbeidet en veileder for å ivareta ernæringsmessig kvalitet i offentlige anskaffelser av mat- og drikkevarer. Veilederen lanseres i starten av 2020 på helsedirektoratet.no. Den inkluderer anskaffelse av måltidstjenester (ikke for heldøgns forpleining), som til kantiner og egner seg også for bruk i privat og frivillig sektor. Et utkast til veileder har vært på høring og gitt mange nyttige innspill. Direktoratet for forvaltning og IKT (Difi) har bidratt anskaffelsesfaglig. Helsedirektoratets kostråd, retningslinjer og anbefalinger ligger til grunn. Kravene bygger på ovennevnte og vilkårene i Nøkkelhullet.

Målgruppe for veilederen er oppdragsgivere i fylker og kommuner som anskaffer mat/måltider for offentlige virksomheter som barnehager, SFO/skole, kantiner, kafeer, kiosker mv. Helsedirektoratet ønsker på sikt å utvikle egne krav og kriterier for anskaffelser som omfatter heldøgns institusjoner, hjemmeboende eldre og andre med spesielle behov. Kosthåndboken, veileder i ernæringsarbeid i helse- og omsorgstjenesten, vil være retningsgivende frem til disse kriteriene foreligger (119).

MATO 4 – støtte- og opplæringssystemer for offentlig sektor

God praksis-beskrivelse<br>**Myndighetene sørger for at det finnes gode støtte- og opplæringssystemer for å hjelpe skoler og andre offentlige institusjoner og deres leverandører med å følge retningslinjene for å servere sunn mat.**	
**Definisjoner og omfang**	• Inkluderer støttesystemer for barnehager og skoler som definert i MATO 1.<br>• Inkluderer støttesystemer for offentlige sammenhenger som definert i MATO 2.<br>• Støtte- og opplæringssystemer omfatter retningslinjer, verktøy, maler (som retningslinjer eller kontrakter), verktøy for planlegging av oppskrifter og menyer, ekspertråd, vurderinger av menyer og produkter, e-læringsressurser, og informasjon og opplæring til kokker eller andre som produserer mat.
**Internasjonale eksempler på god praksis (referansemål)**	**Australia:** The Healthy Eating Advisory Service supports settings such as childcare centers, schools, workplaces, health services, food outlets, parks and sporting centres to provide healthy foods and drinks to the public in line with Victorian Government policies and guidelines. The Healthy Eating Advisory Service is delivered by experienced nutritionists and dieticians at Nutrition Australia Victorian Division. The support includes training cooks, chefs, foods service and other key staff, discovering healthier recipes, food ideas and other helpful resources to provide healthier menus and products (123).<br><br>**Singapore:** The National Workplace Health Promotion Programme, launched in Singapore in 2000, is run by the Health Promotion Board. Both private and public institutions are encouraged to improve the workplace environment by providing tools and grants. Grants are awarded to help companies start and sustain health promotion programmes. Tools include a sample Healthy Workplace Nutrition Policy, a sample Healthy Workplace Catering Policy, and a detailed Essential Guide to Workplace Health, setting out ways to transform the workplace into a health-supporting work environment (107).<br><br>**Netherlands:** The Healthy School Canteen Brigade is a team consisting of dieticians and health scientists of the Dutch Nutrition Center to help school realize healthy canteens (124). They visit schools in the Netherlands and give them advice. The Dutch Nutrition Center also developed the canteen scan a tool to check the level of healthiness of canteens and which gives practical advise (125).

Evidens for implementering

Ressurser knyttet til matservering i barnehage og skole

I tillegg til de nasjonale faglige retningslinjene for måltider i skolen har Helsedirektoratet publisert ressurser i tråd med kostholdsrådene på nettsidene. Kantinekurset *Påfyll* er et verktøy som gir ansvarlige og øvrige som jobber med kantina/matboden kompetanseheving og praktiske grep for å

kunne tilby en kantine i tråd med retningslinjen (126). Kurset består av ulike bolker, blant annet om kantinedrift og kostrådene. Kurset er i tillegg tilgjengelig som e-læringskurs på nettsidene til Nasjonalt senter for mat, helse og fysisk aktivitet (127).

Nasjonalt senter for mat, helse og fysisk aktivitet er et kompetansesenter for å fremme sunt kosthold og fysisk aktivitet i barnehagen og skolen, tilknyttet Høgskulen på Vestlandet. På nettsiden www.mhfa.no tilbys en rekke læringsressurser tilpasset til barnehage, grunnskole, videregående skole og SFO. Læringsressursene består i tillegg til retningslinjene og kostholdsrådene, av plakater, filmer, presentasjoner, konkurranser og mye annet. Det er også et kartleggingsverktøy beregnet til bruk i barnehager for å kartlegge og gi faglig inspirasjon og praksiseksempler rettet mot ledere og ansatte, for å oppfylle rammeplanen for barnehager knyttet til mat og helse. Ressursene formidles hovedsakelig gjennom nettsiden, gjennom ressurser utviklet for lærer- og barnehagelærerutdanning på universitet og høgskoler samt konferanser og webinarer. I forbindelse med større satsinger som reviderte nasjonale retningslinjer har senteret arrangert en rekke konferanser for å spre informasjon og bidra til implementering.

Fiskesprell er et nasjonalt kostholdsprogram med kurs og opplæringsmateriell. Målet er å øke forbruket av fisk og sjømat barnehagen og skole. Fiskesprell er et samarbeid mellom Helse- og omsorgsdepartementet, Nærings- og fiskeridepartementet, Norges sjømatråd, Helsedirektoratet, Havforskningsinstituttet og Fiskesalgslagene. Her er det også mulighet til å søke støtte til råvarer.

Andre ressurser knyttet til offentlig sektor
Som beskrevet under MATO 2 har Helsedirektoratet utgitt, og jobber med implementering av, Nasjonale anbefalinger for mat og drikke i arbeidslivet og anbefalingene Små grep for å tilby sunn mat på farten. Disse er rettet mot arbeidslivet uavhengig om det er offentlig eller privat sektor.

MATO 5 – støtte- og opplæringssystemer for private bedrifter

<table>
<tr><td colspan="2"><u>God praksis-beskrivelse</u>
Myndighetene støtter og oppfordrer aktivt private bedrifter til å tilby og fremme sunn mat og måltider på sine arbeidsplasser.</td></tr>
<tr><td>Definisjoner og omfang</td><td>• "Private bedrifter" omfatter kommersielle bedrifter, men omfatter også ikke-statlige organisasjoner (NGOer), inkludert frivillige eller ideelle og lokale organisasjoner.
• Omfatter retningslinjer for sunt mattilbud (også ved tilstelninger).
• Omfatter støtte- og opplæringssystemer slik som retningslinjer, verktøy, maler (retningslinjer eller kontrakter), verktøy for planlegging av oppskrifter og menyer, ekspertråd, vurderinger av menyer og produkter, e-læringsressurser, og informasjon og opplæring til kokker eller andre som produserer mat.

• Omfatter ikke tilbud av mat til personer som ikke er ansatt i organisasjonen (besøkende, kunder).
• Omfatter ikke støtte for at organisasjoner skal lære sine ansatte om sunn mat generelt.</td></tr>
<tr><td>Internasjonale eksempler på god praksis (referansemål)</td><td>Ireland: A Healthy Workplaces Framework has been developed as part of the governments public health Framework, Healthy Ireland - A Framework for improved health and wellbeing: 2013 – 2025, by the Department of Health and the Department of Business, Enterprise and Innovation (128). It was developed following a public consultation with interested stakeholders and aims to enhance existing initiatives to, facilitate the sharing of experience and learning, and also provide the necessary supports and tools for organizations or companies who haven’t yet developed their own resources. The Healthy Workplaces Framework is due to be implemented in 2020.</td></tr>
</table>

Evidens for implementering

Veiledere og annet

Helsedirektoratet har publisert Nasjonale anbefalinger for mat og drikke i arbeidslivet, som er beregnet på arbeidslivet uavhengig om det er offentlig eller privat sektor. Anbefalingene er basert på de generelle kostholdsrådene og inneholder eksempler på praktiske løsninger for å følge kostholdsrådene og legge til rette for sunne valg på arbeidsplasser med kantine, spiserom og for «mat på farten». En brosjyre Mat og helse – Små grep for å tilby sunn mat og drikke, utdyper rådene. Det er også laget egne anbefalinger for salgssteder som tilbyr «mat på farten». Helsedirektoratet arbeider med å formidle anbefalingene via aktørene i Intensjonsavtale og Saltpartnerskap, via nyhetsbrev til fylker og kommuner via fagpresse, bedriftshelsetjeneste og kampanjer.

OMRÅDE 6: MAT I HANDELEN

Myndighetene har mandat til å implementere politikk/retningslinjer og tiltak for å fremme tilgjengelighet av sunn mat og begrense tilgjengelighet av usunn mat i lokalsamfunn (tetthet og plassering av salgs- og serveringssteder) og i butikk (plassering av produkter).

Det er fire indikatorer for dette området:

- HANDEL 1 – lovverk og reguleringsplaner begrenser tilgang til usunn mat
- HANDEL 2 – lovverk og reguleringsplaner fremmer tilgang til sunn mat
- HANDEL 3 – støttesystemer til dagligvarehandelen
- HANDEL 4 – støttesystemer for serveringsbransjen

HANDEL 1 – lovverk og reguleringsplaner begrenser tilgang til usunn mat

**God praksis-beskrivelse**<br>**Reguleringsplaner og politikk for stedsutvikling er implementert for å begrense tetthet, plassering av, og/eller tilgang (dvs. åpningstider) til serverings- og utsalgssteder som i hovedsak selger usunn mat i lokalsamfunn.**	
**Definisjoner og omfang**	• Omfatter vurdering av folkehelse i nasjonalt eller regionalt lovverk eller styringsdokumenter som styrer politikk, prioriteringer og mål som skal implementeres på lokalt nivå, gjennom for eksempel kommuneplaner.<br>• Omfatter vurdering av folkehelse i andre nasjonale eller regionale planleggingsdokumenter og -retningslinjer.<br>• Omfatter en nasjonal eller regional retningslinje som fastsetter mål om å vurdere folkehelse ved vurdering og godkjennelse av planer for etablering av hurtigmatsteder.<br>• Omfatter begrensninger på utsalgssteder av usunn mat (for eksempel åpningstider).<br>-----------------------<br>• Omfatter ikke lokale myndigheters lover, retningslinjer eller tiltak.
**Internasjonale eksempler på god praksis (referansemål)**	**South Korea:** In 2010 the Special Act on Children's Dietary Life Safety Management established the creation of 'Green Food Zones' around schools, banning the sale of foods (fast food and soda) deemed unhealthy by the Food and Drug Administration of Korea within 200 metres of schools. In 2016, Green Food Zones existed at over 10000 schools (129, 130).<br><br>**UK:** Around 15 local authorities have developed "supplementary planning documents" on the development of hot food takeaways. The policies typically exclude hot food takeaways from a 400m zone around the target location. All policies include secondary schools, some policies also include primary schools, parks and youth centres (131).<br><br>**Detroit USA:** In Detroit, the zoning code prohibits the building of fast food restaurants within 500 ft. of all elementary, junior and senior high schools (131).

Evidens for implementering

Lovverk og veiledere

Folkehelseloven slår fast at kommuner og fylkeskommuner skal fremme helse innen de oppgaver og med de virkemidler de er tillagt (110). Kommuner og fylkeskommuner skal ha oversikt over helsetilstanden til befolkningen i sin kommune/sitt fylke, og de faktorer som påvirker helsetilstanden. Denne kunnskapen skal legges til grunn i arbeidet med planstrategi og som grunnlag for fastsetting av mål og strategier gjennom kommuneplanarbeidet. Loven bygger på, og er samordnet med plan- og bygningsloven som er det generelle regelverk for å fremme bærekraftig utvikling til beste for den enkelte, samfunnet og framtidige generasjoner.

Helsedirektoratet har utarbeidet Veiviser i lokale folkehelsetiltak, som tilsvarer Nasjonale faglige råd (132). Veiviseren beskrives som et praktisk hjelpemiddel i kommunenes arbeid med tiltak for å

bedre befolkningens helse. Kapittel 10 handler om ernæringsarbeid i kommunen. Rådet støtte ernæringsarbeidet som plan- og folkehelsemyndighet i det tverrsektorielle arbeidet (kapittel 10.2) sier at «*kommunen kan*: *(…) sikre at utsalgssteder og automater for usunn mat ikke lokaliseres i nærheten av skoler».* Det utdypes at *«(…) Det bør være oppmerksomhet rundt utsalgssteder for hurtigmat som hovedsakelig tilbyr usunn mat, særlig i forbindelse med skole og skolevei. I skolen er det forbudt med reklame, men det er viktig at barn også skjermes mot slik markedsføring på andre arenaer i nærmiljøet.».*

Kommentarer

Det er ikke funnet evidens rundt kommuners bruk av veiviseren eller rådene som er beskrevet over.

HANDEL 2 - lovverk og reguleringsplaner fremmer tilgang til sunn mat

**<u>God praksis-beskrivelse</u>**<br>**Reguleringsplaner og politikk for stedsutvikling er implementert for å fremme tilgjengelighet av utsalgssteder som selger fersk frukt og grønnsaker.**	
**Definisjoner og omfang**	• "Utsalgssteder" omfatter supermarkeder, torg, bondens marked, grønnsakshandlere, matbutikker og samvirker.<br>• Inkluderer både stedfaste og mobile utsalgssteder.<br>• Inkluderer politikk som støtter lokale myndigheter med å begrense avgifter og andre krav for å stimulere etablering av utsalgssteder som selger fersk frukt og grønnsaker.<br>• Inkluderer nasjonal eller regional politikk for å strømlinjeforme og standardisere godkjenningsprosesser eller redusere den byråkratiske byrden for slike utsalgssteder.<br>• Inkluderer tiltak for å bedre tilgangen til utsalgssteder for F&G, for eksempel åpningstider, hyppighet av markeder etc.<br>• Inkluderer tilgang til støtteordninger og subsidier til F&G utsalgssteder.<br>---------------------------<br>• Omfatter ikke tiltak som offentlige grønnsakshager som er etablert i lokalsamfunn og som vanligvis administreres av lokale myndigheter.<br>• Omfatter ikke generelle retningslinjer om etablering og markedsføring av F&G utsalgssteder.<br>• Ekskluderer lokale myndigheters lover, retningslinjer eller tiltak.
**Eksempler på internasjonal god praksis**	**USA:** In February 2014, the US Congress formally established the Healthy Food Financing Initiative (following a three-year pilot) which provides grants to states to provide financial and/or other types of assistance to attract healthier retail outlets to underserved areas. The pilot distributed over 140 million USD in grants to states to provide financial and other types of assistance to attract healthier retail outlets in underserved areas. To date, 23 US states have implemented financing initiatives (131).<br><br>**New York City (USA):** The 'Green Cart Permit' was developed with reduced restrictions on zoning requirements to increase the availability of fresh fruits and vegetables in designated, underserved neighbourhoods. In 2008 1000 licenses for green carts were made available to street vendors who exclusively sell fresh fruit and vegetables (131). In addition, in 2009, New York City established the food retail expansion to support the health program of New York City (FRESH). Under the programme, financial and zoning incentives are offered to promote neighbourhood grocery stores offering fresh meat, fruit and vegetables in under-served communities. The financial benefits consist of an exemption or reduction of certain taxes. The zoning incentives consist of providing additional floor area in mixed buildings, reducing the amount of required parking, and permitting larger grocery stores in light manufacturing districts (131).

Evidens for implementering

Lovverk og veiledere
Folkehelseloven slår fast at kommuner og fylkeskommuner skal fremme helse innen de oppgaver og med de virkemidler de er tillagt (110). Kommuner og fylkeskommuner skal ha oversikt over helsetilstanden til befolkningen i sin kommune/sitt fylke, og de faktorer som påvirker helsetilstanden. Denne kunnskapen skal legges til grunn i arbeidet med planstrategi og som grunnlag for fastsetting av mål og strategier gjennom kommuneplanarbeidet. Loven bygger på, og er samordnet med plan- og bygningsloven som er det generelle regelverk for å fremme bærekraftig utvikling til beste for den enkelte, samfunnet og framtidige generasjoner. Fylkesmannen skal bidra til å iverksette nasjonal politikk på folkehelseområdet og være pådriver for kunnskapsbasert folkehelsearbeid på lokalt og regionalt nivå, blant annet gjennom råd og veiledning til kommuner og fylkeskommuner

Helsedirektoratet har utarbeidet Veiviser i lokale folkehelsetiltak (132). Veiviseren beskrives som et praktisk hjelpemiddel i kommunenes arbeid med tiltak for å bedre befolkningens helse. Veiviseren har et kapittel om ernæringsarbeid i kommunen. Rådet støtte ernæringsarbeidet som plan- og folkehelsemyndighet i det tverrsektorielle arbeidet sier at kommunen kan *«Sikre tilgjengelighet av sunne valg og mulighet for et sunt kosthold i kommunens arbeidsplasser, institusjoner og øvrige arenaer»* og at kommunen kan *«Samarbeide med lokalt næringsliv om å gjøre sunne valg lettere tilgjengelig i kafeer og andre serveringssteder, kiosker, bensinstasjoner og andre hurtigmatutsalg, og spesielt på fritidsarenaer for barn og unge»*.

Det utdypes videre at *«gjennom kommunale planer kan kommunen i stor grad påvirke til at egne virksomheter og lokalmiljøene tilrettelegger for sunne valg og tilgang til rent drikkevann i det offentlige rom.»*

Kommentarer

Det er ikke funnet noe evidens rundt kommuners eller fylkesmenn sin bruk av veiviseren eller rådene som er beskrevet over.

HANDEL 3 – støttesystemer til dagligvarehandelen

**<u>God praksis-beskrivelse</u>**<br>**Myndighetene sikrer at det eksisterer støttesystemer som oppmuntrer matbutikker til å fremme tilgjengelighet av sunne matvarer og begrense tilgjengelighet av usunn mat i butikklokalene.**	
**Definisjoner og omfang**	• "Matbutikker" omfatter nærbutikker, supermarkeder, grønnsakhandlere og andre spesialiserte matbutikker.<br>• "Støttesystemer" omfatter retningslinjer, ressurser eller ekspertråd.<br>• Omfatter alle steder og sammenhenger som har utsalgssteder for mat, slik som togstasjoner, tilstelninger, fasiliteter og arrangementer som oppsøkes av offentligheten/befolkningen.<br>• "I butikklokaler" omfatter bruk av viktige markedsføringsområder som hylle-ender og betalingspunkt samt bruk av skilting, gulvmerking og andre reklamemetoder.<br>• Omfatter bruk av skilting og merking for å fremme sunne matvalg (for eksempel trafikklys eller godkjente sunnhetsmerker).<br>• Omfatter å endre på ingredienser for å gjøre oppskrifter sunnere, eller endring av menyer eller butikkens fysiske innretning/planløsning for å tilby sunnere valg.<br>• Omfatter å redusere tilbudet av usunne mat- og drikkevarer.<br>-------------------<br>• Omfatter ikke settings/steder og sammenhenger som eies eller drives av myndighetene (se MATO 2 og 4).<br>• Omfatter ikke reformulering og merking i forbindelse med næringsstoffer som bør begrenses som er omfattet av SAMM 1 og MERK 4.
**Internasjonale eksempler på god praksis (referansemål)**	**USA:** The Special Supplemental Nutrition Program for Women, Infants, and Children (WIC) requires WIC authorised stores to stock certain healthier products (e.g. wholegrain bread) (78).<br><br>**The Netherlands:** The National Action plan for vegetables and Fruit is a cooperation of government, industry and civil society organisations. The goal is to increase the consumption of vegetables and fruits in 3 years (2018-2020) by linking and strengthening existing initiatives. The National Action Plan for vegetables and fruit stimulates consumers to eat more vegetables and fruit using the motto 'Go for Colour'. As part of 'Go for Colour' an in-store experiment has taken place promoting the in-store availability of vegetables and fruit (133).

Evidens for implementering

Aktørene i Intensjonsavtalen får tilgang på kampanjeelementer som Helsedirektoratet utvikler blant annet når det gjelder #MerAv satsingen og Nøkkelhullet.

HANDEL 4 – støttesystemer for serveringsbransjen

<table>
<tr><td colspan="2"><u>God praksis-beskrivelse</u>
Myndighetene sikrer at det eksisterer støttesystemer som oppmuntrer til promotering og tilgjengelighet av sunn mat hos serveringssteder, på bekostning av usunn mat.</td></tr>
<tr><td>Definisjoner og omfang</td><td>• "Serveringssteder" omfatter kommersielle hurtigmatsteder (inkludert steder som kun serverer take-away), restauranter, kafeer, kiosker, bensinstasjoner og puber.
• "Støttesystemer" omfatter retningslinjer, ressurser eller ekspertråd.
• Omfatter alle steder og sammenhenger som har utsalgssteder for mat, slik som togstasjoner, tilstelninger, fasiliteter og arrangementer som oppsøkes av offentligheten/befolkningen.
• Inkluderer strategisk plassering av mat og drikke i skap, kjøleskap, på hyller eller nær betalingspunktet.
• Inkluderer bruk av skilting for å fremme sunne valg (trafikklys eller andre sunnhetsmerkinger).
• Inkluderer å endre resepter for å gjøre mat og drikke sunnere, eller å endre menyen for å tilby sunnere valg.

• Omfatter ikke settings/steder og sammenhenger som eies eller drives av myndighetene (se MATO 2 og 4).
• Omfatter ikke reformulering og merking i forbindelse med næringsstoffer som bør begrenses som er omfattet av SAMM 1 og MERK 4.</td></tr>
<tr><td>Internasjonale eksempler på god praksis (referansemål)</td><td>USA: In December 2011, San Francisco implemented the Health Food Incentives Ordinance which bans restaurants, including takeaway restaurants, to give away toys and other free incentive items with children's meals unless the meals meet nutritional standards as set out in the Ordinance: meals must not contain more than 600 calories and include a min amount of fruits and vegetables. It also applies to drinks with excessive calories, fat, excessive sugars, added non-nutritive sweeteners or caffeine (55).

France: Since January 2017 France has banned unlimited offers of sweetened beverages for free or at a fixed price in public restaurants and other facilities accommodating or receiving children under the age of 18. Sweetened beverages are defined as any drink sweetened with sugar or artificial (caloric and non-caloric) sweeteners, including flavoured carbonated and still beverages, fruit syrups, sport and energy drinks, fruit and vegetable nectars, fruit- and vegetable-based drinks, as well as water- milk- or cereal based beverages (131).

Los Angles, USA: In September 2013, the Los Angeles County Department of Public Health launched Choose Health LA Restaurants in partnership with local restaurants to promote healthier meal choices. Restaurants must apply to become a partner. Participating restaurants offer customers smaller portion size options (in addition to existing items on the menu), healthier meals for children that include vegetables and fruit, healthy beverages, non-fried food and free chilled water. Participating restaurants are recognised as Public Health partners in promoting healthier communities (134).</td></tr>
</table>

Evidens for implementering

Helsedirektoratet har publisert en anbefaling for mat- og drikketilbudet i serveringsbransjen med tittel Serveringsmarkedet - mat- og drikketilbud i tråd med kostrådene (135). Anbefalingene består kort av generelle mål og anbefalinger for hvordan man kan følge de generelle kostholdsrådene, samt henvisninger til aktuelt støttemateriell utarbeidet for institusjoner, kantiner i bedriftsmarkedet med mer (nevnt i MATO 4 og 5).

Helsedirektoratet bidrar med formidling av ressursene overfor serveringsbransjen via aktørene i Intensjonsavtale og Saltpartnerskap, og via fagpresse og kampanjer.

Food-EPI del 2 – Infrastruktur-områder

OMRÅDE 7: LEDERSKAP

Den politiske ledelsen sikrer at det er sterk støtte til visjonen for, samt planleggingen, kommunikasjonen, gjennomføringen og evalueringen av politikk og tiltak for å skape sunne matomgivelser, forbedre ernæring i befolkningen og redusere sosiale ulikheter i kosthold og helse.

Det er fem indikatorer for dette området:

- LEDER 1 – sterk, synlig politisk støtte
- LEDER 2 – etablerte mål for kostholdet i befolkningen
- LEDER 3 – etablerte/implementerte kostråd
- LEDER 4 – omfattende handlingsplan for ernæring
- LEDER 5 – redusere sosiale ulikheter i kosthold og helse

LEDER 1 – sterk, synlig politisk støtte

**God praksis-beskrivelse**<br>**Det er sterk, synlig, politisk støtte (på regjeringsnivå) for forbedring av matomgivelser, befolkningens kosthold, kostholdsrelaterte ikke-smittsomme sykdommer (NCDs) og relaterte sosiale ulikheter.**	
**Definisjoner og omfang**	• Synlig støtte omfatter uttalelser om intensjoner, valgforpliktelser, budsjettforpliktelser, etablering av mål og prioriteringer, uttalt støtte for saker i media og andre handlinger som viser støtte for ny eller forsterket politikk/tiltak.<br>• Dokumenter som kan bekrefte sterk politisk støtte inkluderer medieoppslag, taler, politiske valgdokumenter, lovforslag, eller nasjonale strategiske planer med mål eller nøkkelindikatorer.
**Internasjonale eksempler på god praksis (referansemål)**	**New York City (USA):** As Mayor of New York City, Michael Bloomberg prioritised food policy and introduced a number of ground breaking policy initiatives including 'Health Bucks', a restriction on trans fats, establishment of an obesity taskforce, a portion size restriction on sugar-sweetened beverages, public awareness campaigns, etc. He showed strong and consistent leadership and a commitment to innovative approaches and cross-sectoral collaboration (136).<br><br>**Brazil:** The Minister of Health showed leadership in developing new dietary guidelines that are drastically different from the majority of dietary guidelines created by any nation to date, and align with some of the most commonly cited recommendations for healthy eating (137).<br><br>**Ireland:** Healthy Ireland "A Framework for Improved Health and Wellbeing 2013-2025", was launched in 2013 and aims to increase the proportion of people who are healthy at all stages of life, reduce health inequalities, protect the public from threats to health and wellbeing and create an environment where every individual and sector of society can play their part in achieving a healthy Ireland. In 2016, the Government approved the creation of a Healthy Ireland Fund with an initial allocation of €5 million approved in Budget 2017 to establish and support the implementation of Healthy Ireland programmes and projects in a variety of settings. The primary aim of the fund is to support innovative, cross-sectoral, evidence-based projects and initiatives that support the implementation of key national policies in areas such as obesity, smoking, alcohol, physical activity and sexual health (138). The Department of Health has approved a third round of funding, which aims to support local and national organisations to deliver actions that will improve health and wellbeing.<br><br>**The Netherlands:** In 2018, the Ministry of Health, together with more than 70 organizations signed the National Prevention Agreement. It aims to reduce smoking, overweight and problematic alcohol consumption. The agreement includes voluntary ambitions, objectives and actions on these three subjects for the period (2018-2040). The National prevention agreement acknowledges that peoples' contexts is important, and that, among other factors, a healthy environment is needed for those that need it in order to

	prevent overweight, obesity and NCDs. The agreement formulates that inhabitants of the Netherlands need a healthy social, economic and physical environment that supports healthy living, including schools, care facilities, restaurants, cafes, caterers and supermarkets. Specific voluntary targets with respect to the food environment are:<br>- In 2020, 2,500 sports clubs will be working on providing a healthier range of food products in their sports canteens.<br>- No later than 2025, 50% of hospitals will offer a healthy diet; no later than 2030, all of them will.<br>- In 2020, there will be 950 healthy school canteens. This means that 50% of all school canteens will be healthy.<br>- Businesses will develop healthier products (e.g. reformulation by limiting sugar content)<br>- The central government wants to introduce, no later than 2020, a new, broadly supported food-choice logo based on thorough, independent consumer research (139).
**Kontekst**	Kostholdsområdet lå fram til 2018 under helse- og omsorgsministeren. I 2018 ble kostholdsområdet overtatt av den nyopprettede eldre- og folkehelseministerposten.

Evidens for implementering

I 2014 initierte helse- og omsorgsminister Bent Høie en næringslivsgruppe for matområdet for å etablere et tettere samarbeid mellom myndigheter og matvarebransjen. Opprettelsen av Næringslivsgruppen førte til at Helsedirektoratet etablerte Saltpartnerskapet i 2015. Senere ble det frivillige samarbeidet (etter et forslag fra NHO Mat og Drikke) utvidet til Intensjonsavtalen for et sunnere kosthold, signert av helse- og omsorgsministeren og næringsorganisasjoner, mat- og drikkevareprodusenter og dagligvarebransjen i 2016 (140).

Ifølge Folkehelsemeldinga — Gode liv i eit trygt samfunn skal regjeringen utarbeide en ny NCD-strategi (4). Arbeidet med denne igangsettes i 2020.

Da Helsedirektoratets fagrapport med innspill til NCD-strategien som blant annet inneholdt et forslag om å vurdere restriksjoner på størrelsen på vin ble publisert (80), ble forslaget umiddelbart forkastet av daværende eldre- og folkehelseminister Åse Michaelsen (141). I 2019 fikk den nye eldre- og folkehelseministeren Sylvi Listhaug mye medieoppmerksomhet da hun uttalte at hun som folkehelseminister ikke hadde *«planer om å være moralpoliti»* og at *«(…) folk skal få lov til å røyke, drikke og spise så mye rødt kjøtt de bare vil»* (142). Ny eldre- og folkehelseminister fra desember 2019, Terje Søviknes, uttalte seg også til pressen: *«Jeg er ikke glad i moralpoliti. Som folkehelseminister skal jeg spre informasjon slik at alle kan ta sunnere valg om for eksempel ernæring, tobakk og alkohol. Vi driver med forebygging, men valget skal tas av den enkelte»* (143).

LEDER 2 – etablerte mål for kostholdet i befolkningen

God praksis-beskrivelse<br>**Myndighetene har etablert klare mål for befolkningens kosthold når det gjelder næringsstoff som bør begrenses og/eller relevante matvaregrupper, for å nå næringsstoff- og/eller kostholdsanbefalinger gitt av WHO og nasjonale myndigheter.**	
**Definisjoner og omfang**	• Inkluderer mål som oppgir inntak i befolkningen basert på gjennomsnittlig reduksjon i prosent eller volum (mg, g) for næringsstoffene salt, mettet fett, transfett eller tilsatt sukker/frie sukkerarter (sukkerarter som ikke er en del av matvarens naturlige næringsinnhold).<br>• Indikatoren vil typisk kreve at myndighetene etablerer klare næringsstoffanbefalinger med maksimumsanbefalinger for næringsstoffer som bør begrenses.
**Internasjonale eksempler på god praksis (referansemål)**	**Brazil:** The "Strategic Action Plan for Confronting NCDs in Brazil, 2011-2022" specifies a target of increasing adequate consumption of fruits and vegetables, from 18.2% to 24.3 % between 2010 and 2022 and reduction of the average salt intake of 12 g to 5 g, between 2010 and 2022 (144).<br><br>**South Africa:** The South African plan for the prevention and control of non-communicable diseases includes a target on reducing mean population intake of salt to <5 grams per day by 2020 (145).<br><br>**UK:** In August 2016, government set out its approach to reduce the prevalence of childhood obesity in 'Childhood obesity: a plan for action'. A key commitment in the plan was to launch a broad, structured sugar reduction programme to remove sugar from everyday products. All sectors of the food and drinks industry are challenged to reduce overall sugar across a range of products that contribute most to children's sugar intakes by at least 20% by 2020, including a 5% reduction in the first year of the programme (146).<br><br>**The Netherlands:** On January 2014, the Dutch Ministry for Health, Welfare and Sport, signed an agreement with trade organisations representing food manufactures, supermarkets, hotels, restaurants, caterers and the hospitality industry. The agreement included intake targets for example; a maximum of 6 grams of salt consumption per day in 2020 and consuming a maximum of 10% energy from saturated fat per day in 2020 (147).<br><br>**Norway:** The National Action Plan for a Better Diet (2017-2021) contains quantitative intake targets for nutrient of concern and specific food groups in the population. By 2021, the plan sets out a reduction of the following nutrients: Added sugar from 13 to 11E%; saturated fat from 14 to 12E%; and a 22% reduction in salt intake from 10 g/day. There are specific targets to halve the proportion of youth that consumes sugar-sweetened beverages or sweets more than 5 times per week; to double the proportion of youth that eats fruit and vegetables daily; and to increase by 20% the proportion of youth that eats fish at least once a week. There are also targets to

	increase the intake of fruit, vegetables, whole grain products and fish with 20% in the general population (148).

Evidens for implementering

Nasjonal handlingsplan for bedre kosthold (2017-2021) er utgitt av åtte departementer. Planen har som et av hovedmålene å legge til rette for å endre befolkningens kosthold i tråd med helsemyndighetenes næringsstoffanbefalinger og kostråd (99). Handlingsplanen har også kvantitative mål for endring av kosten for både næringsstoffer som bør begrenses og for flere matvaregrupper.

<u>Næringsstoffer</u>
Det er satt følgende kvantitative mål for E% i kosten for næringsstoffer som bør begrenses og som skal nås innen 2021:

- Tilsatt sukker: 11 E% (reduseres fra 13 E%)
- Mettet fett: 12 E% (reduseres fra 14 E%)
- For salt er målet en reduksjon på 22% (fra 10 gram/dag)

<u>Matvaregrupper</u>
Det legges opp til 20% økt forbruk av matvaregruppene grønnsaker, frukt og bær, grove kornvarer og fisk innen 2021. Det er i tillegg egne mål for barn og unge både når det gjelder reduksjon og økning i inntak:

- Andelen 15-åringer som spiser godteri 5 ganger/uke eller oftere og andelen 15-åringer som drikker brus med sukker 5 g/uken eller oftere skal reduseres med 50% (fra ca. 16% for godteri og 19% for brus, gjennomsnitt for begge kjønn)
- Andelen 15-åringer som daglig spiser grønnsaker og andelen som daglig spiser frukt skal økes med 50% (fra 32% daglig for grønnsaker og 36% daglig for frukt)

Andelen unge som spiser fisk til middag minst en gang i uken, og andelen unge som spiser fiskepålegg minst tre ganger i uken skal økes med 20% (fra 71% for fisk ukentlig og 7% for fiskepålegg).

LEDER 3 – etablerte/implementerte kostråd

**<u>God praksis-beskrivelse</u>**<br>**Klare, veiledende, evidens- og matvarebaserte kostråd har blitt fastsatt og implementert.**	
**Definisjoner og omfang**	• Matvarebaserte kostråd bør være angitt for begge kjønn og viktige aldersgrupper, inkludert spedbarn og gravide.<br>• «Veiledende» betyr at det er utviklet verktøy eller informasjon slik som tallerkenmodeller eller matvarepyramider som visualiserer rådene.<br>• «Evidensbasert» inkluderer omfattende gjennomganger av oppdatert forskning og metoder/mekanismer for å innhente ekspertuttalelser/input.<br>• Evidens for denne indikatoren inkluderer måten kostholdsråd har blitt brukt for å utvikle og implementere politikk og tiltak for å bedre kosthold.
**Internasjonale eksempler på god praksis (referansemål)**	**Brazil:** The national dietary guidelines of Brazil address healthy eating from a cultural, ethical and environmental perspective, rather than based on number of servings per food group. The main recommendations are: 'Make natural or minimally processed foods the basis of your diet'; 'use oils, fats, salt, and sugar in small amounts for seasoning and cooking foods'; 'use processed foods in small amounts'; 'avoid ultra-processed foods'. They also provide advice on planning, shopping and sharing meals, as well as warning people to be wary of food marketing and advertising (149, 150).<br><br>**Ireland:** A Healthy Weight for Ireland, Obesity Policy and Action Plan - 2016–2025 called for the development of a suite of Healthy Eating Guidelines for the general population. These revised Healthy Eating Guidelines and Food Pyramid resources, are an early action under the Plan (Action 5.3) (151). The revised Healthy Eating Guidelines and Food Pyramid are based on the FSAI Scientific Recommendations for Healthy Eating Guidelines in Ireland from 2011 (152). They describe how to build a healthy diet, for different age groups (from 5 years of age), depending also on gender and activity levels. The Guidelines, Food Pyramid and supporting resources have been published, disseminated and communicated in 2017, including dissemination of the new Guidelines to all primary and post-primary schools. The revised Healthy Eating Guidelines and Food Pyramid toolkit has been developed by the Department of Health and the Health Service Executive with key stakeholders and aims to help reduce the intake of high fat, salt and sugar (HFSS) foods and drinks from the Top Shelf of the Food Pyramid (153).<br><br>**The Netherlands:** The Dutch Health Council published the 'Guidelines Good Food' 2015. These guidelines advise to eat more plant-based and less animal-based food and include advice on the intake of different food products. The Dutch Nutrition Center published the 'Wheel of Five' Guidelines, based on the 'Guidelines Good Food' of the Dutch Health Council. The 'Wheel of Five' includes advice on the ingredients of a healthy diet, making a distinction between five sections: 1) Vegetables and fruit 2) Spread and cooking fats 3) Fish, legumes, meat, eggs, nuts and dairy products 4) Bread, cereal products and potatoes and 5) Drinks (154).

Evidens for implementering

Nasjonale kostråd i Norge er basert på rapporten *Kostråd for å fremme folkehelsen og forebygge kroniske sykdommer* publisert av Nasjonalt råd for ernæring i 2011 (155), og Nordic Nutrition Recommendations 2012 (156). Kostrådene utarbeides etter en protokoll basert på systematisk oppsummering av systematiske litteraturoversikter.

Det er tolv kostråd der de to første er oppsummerende råd om helheten i kostholdet og om energibalanse. Ni råd omhandler ulike matvaregrupper eller matvarer/ingredienser (salt og sukker). Fem av rådene er kvantitative, det vil si at det oppgis antall porsjoner eller mengder som anbefales inntatt hver dag. Det siste rådet omhandler fysisk aktivitet. Det presenteres også oppsummerende råd (figur 5).

Kostrådene kort oppsummert

Spis mer av dette:

- Grønnsaker
- Frukt
- Bær
- Fisk og fiskeprodukter

Spis mindre av dette:

- Rødt kjøtt og kjøttprodukter
- Salt og matvarer med mye salt
- Sukker, brus, saft og godteri

Velg disse matvarene:

- Grove kornprodukter i stedet for fine
- Olje og myk margarin i stedet for smør
- Magre meieriprodukter i stedet for fete
- Vann i stedet for saft og brus

Slik kan du følge kostrådene

1. Ha et variert kosthold med mye grønnsaker, frukt og bær, grove kornprodukter og fisk, og begrensede mengder bearbeidet kjøtt, rødt kjøtt, salt og sukker.
2. Ha en god balanse mellom hvor mye energi du får i deg gjennom mat og drikke, og hvor mye du forbruker gjennom aktivitet.
3. Spis minst fem porsjoner grønnsaker, frukt og bær hver dag.
4. Spis grove kornprodukter hver dag.
5. Spis fisk til middag to til tre ganger i uken. Bruk også gjerne fisk som pålegg.
6. Velg magert kjøtt og magre kjøttprodukter. Begrens mengden bearbeidet kjøtt og rødt kjøtt.
7. La magre meieriprodukter være en del av det daglige kostholdet.
8. Velg matoljer, flytende margarin og myk margarin, fremfor hard margarin og smør.
9. Velg matvarer med lite salt, og begrens bruken av salt i matlagning og på maten.
10. Unngå mat og drikke med mye sukker til hverdags.
11. Velg vann som tørstedrikk.
12. Husk #Dine30 – hver dag

Figur 5. Oppsummerende kostråd fra https://helsenorge.no/kosthold-og-ernaring/kostrad/helsedirektoratets-kostrad

Den primære kanalen for å formidle kostrådene til befolkningen er nettstedet helsenorge.no og gjennom befolkningskampanjer. I tillegg benytter helsemyndighetene kanaler i sosiale medier (Facebook og Instagram).

Mengdene som er angitt i kostrådene er tilpasset behovet til en normalt fysisk aktiv voksen person. Kostrådene er ikke videre angitt for kjønn eller aldersgrupper. Det er ikke utarbeidet veiledende visuelt materiale som formidler kostrådene, men helsenorge.no har også fakta-sider om kosthold som viser til for eksempel tallerkenmodellen (157).

Kostrådene er en sentral del av norsk ernæringspolitikk og gjenspeiles i gjeldende Handlingsplan for bedre kosthold (2017-2021) samt i annen politikk på kostholdsområdet. For eksempel er flere nasjonale faglige retningslinjer og veiledere for ernæring basert på kostrådene. Dette gjelder blant annet:

1) Nasjonal faglig retningslinje for mat og måltider i barnehagen (109)
2) Nasjonal faglig retningslinje for mat og måltider skolen (omfatter barneskole og skolefritidsordning, ungdomsskolen og videregående skole) (73)
3) Kosthåndboken – veileder i ernæringsarbeid i helse- og omsorgstjenesten (119)

Kostrådene er også et sentralt element i Intensjonsavtalen for et sunnere kosthold (20), og i mat- og helsefaget i barne- og ungdomsskolen.

LEDER 4 – omfattende handlingsplan for ernæring

<table>
<tr><td colspan="2"><u>God praksis-beskrivelse</u>
Det finnes en omfattende, åpen (transparent) og oppdatert handlingsplan som er koblet til nasjonale behov og prioriteringer. Planen skal bidra til å bedre matomgivelser og redusere inntaket av næringsstoff som bør begrenses for å nå WHOs og nasjonale næringsstoffanbefalinger og kostråd, og redusere ikke-smittsomme sykdommer.</td></tr>
<tr><td>Definisjoner og omfang</td><td>• Omfatter dokumenterte planer med spesifikke handlinger (actions) og intervensjoner, det vil si policies, programmer, partnerskap.
• Planene bør være gjeldende, det vil si at de er støttet av sittende regjering og/eller at planen blir overvåket av sittende myndigheter.
• Planer bør referere til tiltak for å bedre matomgivelser (som beskrevet i politikk-områdene overfor) og bør inkludere både policy- og programstrategier (dvs. både på overordnet og mer spesifikt nivå).
• Planen kan omfatte prioriterte strategier, kommunikasjonstiltak overfor befolkningen og trussel om regulering i forbindelse med frivillige tiltak.

• Omfatter ikke overordnede rammeverk som angir generell veiledning og retning.</td></tr>
<tr><td>Internasjonale eksempler på god praksis (referansemål)</td><td>Ireland: 'A Healthy Weight for Ireland', the Obesity Policy and Action Plan 2016-2025 (OPAP), was launched in September 2016 under the auspices of the Healthy Ireland agenda. The OPAP covers a ten-year period up to 2025, which prescribed 'Ten Steps Forward' that would be taken to reverse obesity trends, prevent health complications and reduce the overall burden for individuals, families, the health system, and the wider society and economy (151). A new Obesity Policy Implementation Oversight Group (OPIOG) was established in October 2017 and a progress report on each recommendation in the OPAP is currently being finalised under the aegis of the OPIOG. As set out in Healthy Ireland, integrated health and social impact assessments will be conducted on relevant policy areas to support other government departments in contributing towards the prevention of overweight and obesity (128).</td></tr>
</table>

Evidens for implementering

Nasjonal handlingsplan for bedre kosthold 2017-2021 (99) er det førende styringsdokumentet for kosthold i Norge. Den forrige nasjonale handlingsplanen for kosthold gikk fra 2007 til 2011. I sin sluttrapport for handlingsplanen fra 2012 anbefalte Helsedirektoratet at «det bør lages et nytt styringsdokument om kosthold og ernæring med presise mål og overbyggende tiltak i et langsiktig tidsperspektiv». Etter at Helse- og sosialkomiteen behandlet Stortingsmelding 19 (2015-2015) *Folkehelsemeldingen – Mestring og muligheter,* valgte Stortinget å pålegge regjeringen å lage en handlingsplan for bedre kosthold. I 2017 ble Nasjonal handlingsplan for bedre kosthold publisert, signert av åtte departementer. Handlingsplanen ble midtveisevaluert av et ekspertutvalg, koordinert av FHI, i 2019. Notatet ble oversendt HOD i november 2019 (158).

Kvantitative mål for handlingsplanen er nevnt tidligere, under indikatoren LEDER 2. Planen har også følgende overordnede mål og delmål:

Overordnet mål
Et sunt og variert kosthold i hele befolkningen uavhengig av kjønn, alder, geografi, sosioøkonomisk status, kulturell bakgrunn, funksjonsevne, religion og livssyn.

Delmål

- Legge til rette for å endre kostholdet i tråd med helsemyndighetenes råd
- Redusere sosiale forskjeller i kosthold
- Styrke kunnskap om og synliggjøre sammenhenger mellom kosthold og fysisk og psykisk helse
- Fremme utvikling av sunne og trygge matvarer og tilstrebe en helse- og miljøvennlig praksis i produksjon og forbruk av mat
- Styrke og kvalitetssikre mat-, måltids- og ernæringsarbeid i helse-, omsorgs- og sosialtjenesten

Tiltakene i handlingsplanen er samlet i fem *tiltaksområder* der hvert tiltaksområde består av flere tiltak eller tiltakspakker. Konkrete handlinger innenfor hvert tiltak nevnes i *oppfølgingspunkter* der ansvarlig departement også er oppført. Tiltakene omfatter i stor grad samarbeid med matvarebransjen, revisjon og oppfølging av ulike retningslinjer der ernæring inngår, og ulike former for kommunikasjonstiltak.

- **Måltidsglede og sunt kosthold**
  Tiltakene i dette området handler primært om å fremme gode måltid i barnehage, skole og for eldre, gjennom for eksempel revisjon av rammeplaner og retningslinjer, utvikling av ulike kommunikasjonstiltak og spredning av gode erfaringer og å «videreføre støtte til ulike tiltak som fremmer praktiske matlagingsferdigheter».
- **Gode og enkle valg**
  Tiltak og oppfølgingspunkter i dette området omfatter bl.a. videreføring av samarbeid med matvarebransjen gjennom Intensjonsavtalen og saltpartnerskapet, kommunikasjonstiltak for å redusere inntak av sukker og mettet fett, videreføring av Nøkkelhullsordningen, oppfølgning av selvreguleringsordningen (MFU) av markedsføring av usunn mat og drikke rettet mot barn, og kommunikasjonstiltak for å fremme forbruk av frukt og grønt samt sjømat.
- **Kommunikasjon og kunnskap**
  Eksempler på tiltak og oppfølgningspunkter i dette området omfatter utvikling av verktøy til bruk i mat og helse-faget, videreføring av eksisterende tiltak som Fiskesprell, utvikling av merkevaren *Små grep, stor forskjell* og målretting av kommunikasjon mot spesielle grupper som innvandrere, barn og unge.
- **Mat, måltider og ernæring i helse- og omsorgstjenesten**
  Eksempler på tiltak og oppfølgingspunkt i dette området omfatter kompetanseheving i den kommunale helse- og omsorgstjenesten, oppfølging, formidling og styrking av nasjonale faglige retningslinjer og videreføring av Mor-barn-vennlig initiativ.
- **Forskning, utvikling og innovasjon**
  Eksempler på tiltak og oppfølgingspunkt i dette området omfatter videreføring av kostholdsundersøkelser, kartlegge behovet for relevante kostholdsindikatorer, samt å *be Norges forskningsråd vurdere å videreføre felles utlysning for programmene BEDREHELSE og BIONÆR og eventuelt andre relevante programmer, og å legge til rette for systematisk utprøving og evaluering av tiltak, inkludert bruk av atferdsøkonomi, for å fremme befolkningenes kosthold og helse.*

Kommentarer

Utvalget som har evaluert handlingsplanen kommenterer at det ikke er noen tydelig sammenheng mellom mål og tiltak i planen, men at planen heller må forståes som en samling av kostholdstiltak på nasjonalt nivå. Det kommenteres at mange av tiltakene er videreføring av tiltak som har blitt startet opp ved tidligere anledninger. Av 129 oppfølgingspunkter har omtrent 40 status som «ikke startet» eller «satt på vent». Flere av disse er rettet mot målgrupper som innvandrere, ungdom eller asylsøkere. En del av tiltakene som ikke er påbegynt henger igjen fra forrige handlingsplan. Videre kommenterer utvalget at handlingsplanen i liten grad retter seg mot lokalt nivå, og at det i utstrakt grad er mangel på kunnskap om hvordan tiltak i planen faktisk når ut til målgruppene (for eksempel i forbindelse med kommunikasjonstiltak). Utvalget påpeker også at det ikke følger et budsjett eller ressurser med planen, og at mer kan gjøres hvis det følger midler med en omfattende handlingsplan (158).

Andre tiltaksområder blir omtalt som mer vellykket, for eksempel arbeid med eldre og underernæring. I dette tilfellet er arbeidet også knyttet til en egen kvalitetsreform (*Leve hele livet*) som ble lansert omtrent samtidig med handlingsplanen.

LEDER 5 – redusere sosiale ulikheter i kosthold og helse

<u>**God praksis-beskrivelse**</u><br>**Myndighetene har fastsatt prioriteringer for å redusere sosiale ulikheter eller for å beskytte sårbare grupper i forbindelse med kosthold, ernæring, overvekt og ikke-smittsomme sykdommer.**	
**Definisjoner og omfang**	• Rammeverk, strategier eller implementeringsplaner oppgir spesifikke mål, delmål eller indikatorer for å redusere sosiale ulikheter, inkludert forebyggende tilnærminger som tar for seg sosiale og miljømessige helsedeterminanter.<br>• Rammeverk, strategier eller implementeringsplaner identifiserer sårbare grupper eller prioriteringsgrupper.<br>• Implementeringsplaner inneholder spesifikke policies/tiltak som skal redusere sosial ulikhet for særskilte grupper.<br>---------------------<br>• Omfatter ikke tiltak/prioriteringer for å redusere sosiale ulikheter innen sekundær- eller tertiærforebygging.
**Internasjonale eksempler på god praksis (referansemål)**	**New Zealand:** The Ministry of Health reports the estimates derived from health surveys and nutrition surveys by four subpopulation groups including age group, gender, ethnic group and an area level deprivation index. Similarly, estimates derived from other data types (e.g. mortality) are presented by these subpopulation groups. The contracts between MoH and NGOs or other institutions include a section on Maori Health and state: “An overarching aim of the health and disability sector is the improvement of Maori health outcomes and the reduction of Maori health inequalities”. In the specific contract between the Ministry of Health and Agencies for Nutrition Action the first clause is on Maori Health relating to compliance with any Maori specific service requirements, quality requirements and specific monitoring requirements contained in the Service specifications to this agreement.<br><br>**Australia:** The National Indigenous Reform Agreement (Closing the Gap) is an agreement between the Commonwealth of Australia and the States and Territories. The objective of this agreement is to work together with Indigenous Australians to Close the Gap in Indigenous disadvantage. The targets agreed to by COAG relate to health or social determinants of health. For the target ‘Closing the life expectancy gap within a generation (by 2031)’, one of the performance indicators is the prevalence of overweight and obesity (159).<br><br>**Ireland:** Step 9 of the Healthy Weight for Ireland: Obesity Policy and Action Plan 2016-2025 aims to allocate resources to those population groups most in need of support in the prevention and management of obesity, with particular emphasis on families and children during the first 1,000 days of life. The priority actions to commence in first year were to assess the needs of vulnerable groups as the basis of allocation of resources for preventative and treatment services for children and adults (151). The Healthy Ireland fund was established in 2017 with an allocation of €5 million and with additional allocations of €5 million in 2018 and 2019. The first round of the

	Fund was distributed through Local Community Development Committees, Children and Young Person's Services Committees and statutory organisations. The Fund has been effective at targeting population groups that experience health inequalities. In Round 1 (2017/18), there was a focus on specific groups experiencing health inequalities, including people living in areas of social disadvantage (71% of actions), people with disabilities (45%), people from new communities including refugees and asylum seekers (39%) and members of the Traveller community (36%). Furthermore, of the local actions that were implemented in Round 1, 61% related to physical activity and 32% were related to food, nutrition and weight management. Round 2 of the Fund is currently being implemented. The Healthy Ireland 2019 communications and citizen engagement campaign has continued on from 2018, launching on the 8th of April 2019, with an announced funding of €1 million to boost community engagement on health and wellbeing in every county (160).

Evidens for implementering

Mld.St. 13 (2018-2019). Muligheter for alle. Fordeling og sosial bærekraft
Denne meldingen til Stortinget sammenfatter kunnskap om sosial ulikhet blant annet i helse. Når det gjelder konkrete tiltak på kostholdsfeltet, vises det til ny folkehelsemelding som ble lagt fram våren 2019 (dvs. Meld St. 19 (2018-2019)), samt nasjonal handlingsplan for bedre kosthold (161).

Meld. St. 19 (2018-2019). Folkehelsemeldinga – Gode liv i eit trygt samfunn
Det nasjonale målet for folkehelsepolitikken i Norge, er at Norge skal være blant de landene i verden med lengst levealder, at befolkningen skal oppleve flere leveår med god helse og mindre sosiale helseforskjeller, og det skal skapes et samfunn som fremmer helse i hele befolkningen. Meldingen beskriver at det er store sosiale og geografiske ulikheter i fedme, og at enkelte innvandrergrupper samt menn fra den samiske befolkningen er spesielt utsatt.

Når det gjelder kosthold og ernæring, framholder Regjeringen at den vil *systematisere og gjere tilgjengeleg relevant informasjonsmateriell om kosthald som er kulturelt og språkleg tilpassa ulike målgrupper* (4).

Nasjonal handlingsplan for bedre kosthold
Det overordnede målet i nasjonal handlingsplan for bedre kosthold er: *Et sunt og variert kosthold i hele befolkningen uavhengig av kjønn, alder, geografi, sosioøkonomisk status, kulturell bakgrunn, funksjonsevne, religion og livssyn.*

Ett av delmålene i handlingsplanen er å redusere sosiale ulikheter i kosthold. Det beskrives at det er betydelig sosial ulikhet i kosthold i Norge, og at befolkningsrettede, strukturelle tiltak kan være kostnadseffektive og bidra til å redusere sosiale ulikheter. I et avsnitt om mål og målgrupper skrives det at «hensynet til at det er kostholdsforskjeller mellom kjønn og ulike sosiale grupper, skal ivaretas» uten at noen konkrete målgrupper er nevnt.

Handlingsplanen nevner ingen spesifikke delmål eller indikatorer i forbindelse med sosiale ulikhet, men sosial ulikhet nevnes i forbindelse med flere tiltaksområder. For eksempel blir tiltaksområdet «gode og enkle valg» beskrevet som et befolkningsrettet tiltak som er egnet til å motvirke sosial ulikhet i helse, der samarbeid med matbransjen om produktutvikling er sentralt. Fiskesprell er

omtalt som et program som skal bidra til å utjevne sosiale ulikheter knyttet til kosthold. Tiltak knyttet til amming beskrives også som utjevnende.

I tiltaksområdet «kommunikasjon og kunnskap» framholdes det at «det er viktig å kommunisere tilgjengelig kunnskap til befolkningen generelt og sårbare grupper spesielt». Kommunikasjonssatsningen *Små grep stor forskjell* skal ivareta sosial ulikhet. Ett oppfølgingspunkt i tiltaksområde 3.2 er å *målrette kommunikasjon mot innvandrere og tilpasse informasjonen kulturelt og språklig* (99).

Kommentarer

I forkant av **folkehelsemeldingen** fra 2019, utga Fagrådet for sosial ulikhet i helse en rapport med anbefalte tiltak mot sosial ulikhet i helse (162). To av de foreslåtte tiltakene dreide seg om gratis sunt måltid til alle barn i alle skoler i hele landet hver dag og sunn skatteveksling for frukt og grønt versus sukker, salt og fett. Disse forslagene ble imidlertid ikke fulgt opp med konkrete tiltak i folkehelsemeldingen

I midtveisevaleringen til **handlingsplanen** uttrykker utvalget at en del tiltak som handlingsplanen *beskriver* at er sosialt utjevnende, faktisk ikke er *implementert* (slik som prisvirkemidler og gratis skolefrukt). Det beskrives også at det er «*påfallende at en del av oppfølgingspunktene som har status som 'ikke startet' eller 'på vent' er rettet mot noen av målgruppene som skal gis særskilt oppmerksomhet i handlingsplanen, som f.eks. innvandrergrupper, ungdom og asylsøkere (…) Mangel på spesifikke konkrete tiltak til visse grupper (for eksempel minoritet - og innvandrergrupper) gir uttrykk for at disse ikke er prioritert og målene blir derfor uoppnåelige* (s.16) (158).

OMRÅDE 8: STYRESETT OG FORVALTNING

Myndighetene har etablert strukturer og prosedyrer for å sikre transparens og ansvarlighet. Ved utvikling og implementering av tiltak for å bedre matomgivelser og ernæring og for å redusere ikke-smittsomme sykdommer, oppfordres det til bred deltakelse og inkludering av befolkningen.

Det er fem indikatorer for dette området:

- STYRE 1 – begrense interessekonflikt i politikkutvikling
- STYRE 2 – kunnskapsbasert mat- og ernæringspolitikk
- STYRE 3 – åpenhet i mat- og ernæringspolitikk
- STYRE 4 – tilgang til informasjon

STYRE 1 – begrense interessekonflikt i politikkutvikling

**<u>God praksis-beskrivelse</u>**<br>**Det finnes robuste prosedyrer for å begrense påvirkning fra kommersielle aktører på utviklingen av politikk knyttet til matomgivelser, når deres interesser står i konflikt med tiltak for å forbedre befolkningens ernæring og kosthold.**	
**Definisjoner og omfang**	• Inkluderer offisielle policies, regler, retningslinjer eller andre mekanismer for å styre handlinger og beslutninger for ansatte i forvaltningen, for eksempel prosedyrer for oppgivelse av interessekonflikter.<br>• Inkluderer prosedyrer for å håndtere partnerskap med private bedrifter, eller sammenslutninger som representerer industrien, som blir konsultert med den hensikt å utvikle politikk. For eksempel: retningslinjer for prosedyre.<br>• Inkluderer offentlig tilgjengelig, oppdaterte registre over lobbyister og/eller deres aktiviteter.
**Internasjonale eksempler på god praksis (referansemål)**	**USA:** Mandatory and publicly accessible lobby registers exist at the federal level, as well as in nearly every state. Financial information must be disclosed, and the register is enforced through significant sanctions. A number of pieces of legislation uphold compliance with the register including Lobbying Disclosure Act of 1995 and the Honest Leadership and Open Government Act 2007 (163).<br><br>**New Zealand:** The State Services Commission has published Best Practice Guidelines for Departments Responsible for Regulatory Processes with Significant Commercial Implications. They cover the development and operation of a regulatory process and include specific references to principles around stakeholder relationship management (164).<br><br>**Australia:** The Australian Public Service Commission's Values and Code of Conduct includes a number of relevant sections such as the Conflict of Interest, Working with the Private Sector and other Stakeholders and the Lobbying Code of Conduct (165).<br><br>**Ireland:** The purpose of the Regulation of Lobbying Act (2015) is to provide for a web-based Register of Lobbying to make information available to the public on the identity of those communicating with designated public officials on specific policy, legislative matters or prospective decisions. In support of the Act's objectives to foster transparency and the proper conduct of lobbying activities, the Code of Conduct for persons carrying on lobbying activities was established. Its purpose is to govern the behaviour of persons carrying on lobbying activities. The provisions of the Act can apply to employers; to representative or advocacy bodies; to professional lobbyists or third parties who are being paid to communicate on behalf of a client or other person; and, significantly, to any person communicating about the development or zoning of land (166).
**Kontekst**	Norge er på 7. plass i Transparency Internationals (TI) Corruption Perception Index 2018 med en score på 84%, av totalt 180 land (167). Norge har falt fra

	5. plass med 88% i 2015. TI peker på at det har vært saker i Norge både innenfor politiet og kommunal byggesaksbehandling som bør lede til at flere sektorer kartlegger risiko og iverksetter forebyggende tiltak når det gjelder samrøre mellom ulike interesser (168).

Evidens for implementering

Norge har ikke noe lobby-register. Det er blitt fremsatt som forslag til Stortinget flere ganger, senest i 2017, men har ikke fått gjennomslag. Norge har **regelverk som skal sikre habilitet** og motvirke interessekonflikter i forvaltningen, gjennom **Forvaltningslovens kap. II** (169). En offentlig tjenestemann kan ikke tilrettelegge grunnlaget for en avgjørelse eller til å treffe avgjørelse i en forvaltningssak ved to hovedtilfeller (forvaltningsloven, §6): 1) når vedkommende eller noen i nær familie er part i saken; 2) når det foreligger særegne forhold som egner seg til å svekke tilliten til ens upartiskhet. Ifølge alminnelig forvaltningsrett og lovens § 10 gjelder tilsvarende regler om habilitet også for andre som «utfører tjeneste eller arbeid for et forvaltningsorgan». Forvaltningslovens regler om habilitet omfatter alle som avgir uttalelse eller råd til forvaltningen i tilknytning til enkeltsaker eller saksområder, selv om de formelt sett ikke fatter vedtak i saken. Helseforvaltningen, som Helsedirektoratet, Folkehelseinstituttet, Mattilsynet og deres underliggende rådgivende organer, plikter å følge disse lovene.

For alle fagråd og arbeidsgrupper i Helsedirektoratet vurderes habilitet av medlemmer og gruppedeltakere basert på utfylt habilitetsskjema. Habilitet vurderes på nytt ved ny periode. Dette gjelder for eksempel for deltakere i utvikling av nasjonale retningslinjer og for medlemmene i Nasjonalt råd for ernæring.

Også Vitenskapskomiteen for mat og miljø (VKM) har regler for habilitet som gjelder for alle ansatte i VKM, for medlemmer oppnevnt av HOD og for alle som utfører oppdrag for VKM, iht. forvaltningslovens §10 (170). Disse må fylle ut habilitetserklæring hvor de erklærer om de kjenner til forhold som gjør dem inhabile. Videre må medlemmer og eksperter gi erklæring om habilitet i forkant av hvert møte i faggrupper og arbeidsgrupper. Medlemmer, ansatte og de som utfører oppdrag på vegne av VKM har selv plikt til å påse at de ikke behandler saker hvor de er inhabile.

Tiltak for å redusere risiko for interessekonflikter i arbeidet med Intensjonsavtalen som helseministeren har undertegnet med parter fra matvareindustrien, er: Helsemyndighetene leder arbeidet (Helsedirektoratet har sekretariatet), helsemyndighetene lager agenda og møtereferat til alle møtene, all kommunikasjon er offentliggjort, inkludert: Avtalen og hvem de involverte partene er, agenda og møtereferat, liste av medlemmer og mandatene til koordineringsgruppen, målsettingene for avtalen, resultater av monitorering og måloppnåelse, og evalueringsrapportene. Evalueringen gjøres av en ekstern tredjepart og består av årsrapporter, midtveis-evaluering og sluttevaluering (171). Vedlegg 1 til intensjonsavtalen inneholder overordnede konkurranserettslige retningslinjer for å bidra til å sikre at intensjonsavtalens parter ikke overtrer konkurransereglene i aktiviteter som springer ut av intensjonsavtalen. I større møter i medhold av intensjonsavtalen må alle møtedeltakere signere en skriftlig protokoll om at de kjenner og følger innholdet i vedlegg 1 både før, under og etter møtene (20).

STYRE 2 – kunnskapsbasert mat- og ernæringspolitikk

**<u>God praksis-beskrivelse</u>**<br>**Det er implementert tiltak og prosedyrer som sikrer at utvikling av mat- og ernæringspolitikk er kunnskapsbasert.**	
**Definisjoner og omfang**	• Inkluderer policies, prosedyrer eller retningslinjer for å støtte ansatte i forvaltningen til å bruke kunnskap i utvikling av politikkutvikling, inkludert metodologi for gjennomgang av god praksis (inkludert type forskning og nødvendig styrke av evidens) og implementering av policy når sterkt evidensgrunnlag ikke foreligger (når potensiell risiko er stor, eller når det er risiko for skade ved fravær av tiltak).<br>• Inkluderer policies, prosedyrer eller retningslinjer som fastsetter kravene til etablering av en vitenskapelig eller ekspertkomité som skal gi råd til politikkutvikling.<br>• Inkluderer bruken av evidensbaserte modeller, algoritmer og verktøy for å veilede politikkutvikling, eller til bruk til implementering av en policy, for eksempel ernæringsprofiler.<br>• Inkluderer offentlig finansierte enheter, enten innen eller på tvers av departementer, som ivaretar kunnskap og forskning.
**Internasjonale eksempler på god praksis (referansemål)**	**Australia:** The National Health and Medical Research Council Act 1992 (NHMRC Act) requires NHMRC to develop evidence-based guidelines. These national guidelines are developed by teams of specialists following a rigorous nine-step development process (172).

Evidens for implementering

Folkehelseloven slår fast at folkehelsearbeidet skal være «langsiktig og systematisk», og ifølge forskriften innebærer dette at folkehelsearbeidet skal være kunnskapsbasert (173). Folkehelsearbeidet skal bygge på faglig anerkjente metoder, og på kunnskap om de faktiske ressurser og folkehelseutfordringer. Det er Helsedirektoratet (gjennom folkehelselovens §24) som har ansvar for å være pådriver for kunnskapsbasert folkehelsearbeid, blant annet gjennom utvikling av nasjonale normer og standarder for godt folkehelsearbeid.

Etter folkehelseloven skal Helsedirektoratet følge med på forhold som påvirker folkehelsen og bidra til å iverksette nasjonal politikk på folkehelseområdet. Helsedirektoratet utgir *Folkehelsepolitisk rapport* annethvert år (174).

Kommuner og fylkeskommuner skal, i henhold til folkehelseloven, ha løpende oversikt over helsetilstand og påvirkningsfaktorer (inkludert kosthold og ernæring). Folkehelseinstituttet skal støtte og legge til rette for kommunene og fylkeskommunenes oversikter samt samordne eventuelle fylkeshelseundersøkelser.

Folkehelseinstituttet skal overvåke utviklingen av folkehelsen, utarbeide oversikt over befolkningens helsetilstand og faktorer som påvirker denne, samt utføre helseanalyser og drive forskning på folkehelseområdet (folkehelseloven §25). Folkehelseinstituttet utgir *Folkehelserapporten* som presenterer kunnskap og statistikk om helsetilstanden i Norge, og som oppdateres jevnlig (175). Rapportene fra Helsedirektoratet og Folkehelseinstituttet danner kunnskapsgrunnlag som benyttes i

utformingen av folkehelsepolitikken blant annet gjennom folkehelsemeldingene som utgis hvert fjerde år.

Helsedirektoratets veileder for **utvikling av kunnskapsbaserte retningslinjer** skal benyttes i utviklingen av nasjonale faglige retningslinjer (176). Slike retningslinjer inneholder systematisk utviklede råd og anbefalinger som etablerer en nasjonal standard for forebygging, diagnostisering, behandling og/eller oppfølging av pasientgrupper, bruker-grupper eller diagnosegrupper innenfor helse- og omsorgstjenestene. Eksempler på ernæringsrelaterte retningslinjer er Retningslinjer for spedbarnsernæring, Retningslinjer for forebygging, utredning og behandling av overvekt og fedme hos voksne, m. fl. GRADE-metodikken anbefales for å gradere kvaliteten på kunnskapsgrunnlaget.

Nasjonalt råd for ernæring er et uavhengig ekspertråd som gir faglige, kunnskapsbaserte råd til Helsedirektoratet for å støtte deres faglige rolle innen kosthold og ernæring (177). Nasjonalt råd for ernæring utga i 2011 rapporten Kostråd for å fremme folkehelsen og forebygge kroniske sykdommer (155). Rapporten baserte seg i stor grad på metodikken utviklet av World Cancer Research Fund. Deler av rapporten er blitt oppdatert (178).

Norge utgir i samarbeid med de andre nordiske land anbefalinger for inntak av næringsstoff samt kostråd. Neste versjon skal foreligge i 2022 og denne skal følge systematisk metoder for innhenting og vurdering av kunnskapsgrunnlaget (179).

STYRE 3 – åpenhet i mat- og ernæringspolitikk

<table>
<tr><td colspan="2"><u>God praksis-beskrivelse</u>
Det er implementert tiltak og prosedyrer for å sikre åpenhet i utvikling av mat- og ernæringspolitikk.</td></tr>
<tr><td>Definisjoner og omfang</td><td>• Omfatter retningslinjer eller prosedyrer som styrer bruken av offentlig konsultasjon/høring i utviklingen av mat- og ernæringspolitikk.
• Inkluderer retningslinjer eller prosedyrer for offentlig (nett-) publisering av innspill fra privat og sivil sektor til myndighetene når det gjelder politikkutvikling og påfølgende respons fra myndighetene på disse.
• Inkluderer retningslinjer eller prosedyrer for offentlig (nett-) publisering av utredninger, høringer og endelig politikk.
• Inkluderer retningslinjer eller prosedyrer for å styre kommunikasjonsarbeid rundt all politikk som er vedtatt men som ikke har blitt gjennomført.</td></tr>
<tr><td>Internasjonale eksempler på god praksis (referansemål)</td><td>New Zealand: Food Standards Australia New Zealand (FSANZ) is required by the Food Standards Australia New Zealand Act 1991 to engage stakeholders in the development of new standards. This process is open to everyone in the community including consumers, public health professionals, and industry and government representatives. FSANZ has developed a Stakeholder Engagement Strategy 2013-16 that outlines the scope and processes for engagement. Under the Stakeholder Engagement Priorities 2013-16, it outlined “maintain our open and transparent approach” as one of the first priorities (180).

Norway: The Public Administration Act provides general procedural rules for public administration. The Act regulates the administrative procedures when decisions are made, especially the rights of parties during the procedures. The Central Government Communication Policy contains the central goals and principles of the central government’s communication with citizens, businesses, organizations and other public sector activities. The goals of the central government communication policy state that the citizens shall: receive accurate and clear information about their rights, duties and opportunities; have access to information about central government activities; and be invited to participate in the formulation of policy, schemes and services (181).</td></tr>
</table>

Evidens for implementering

Norge er et samfunn med lang tradisjon for **åpenhet i forvaltningen**. Norge har en egen offentlighetslov (182), og også en forvaltningslov (169) og en arkivlov (183). Disse lovene er med på å sikre en åpen og transparent forvaltning (181).

Utredningsinstruksen har blant annet som hensikt å sikre tidlig involvering og deltakelse i høringsrunder (184). Dette gjelder involvering både av sivilsamfunnsorganisasjoner og enkeltindivider.

Helse- og omsorgsdepartementet har vanligvis åpne prosesser når det gjelder å utvikle stortingsmeldinger og handlingsplaner. Høringsinnspill legges vanligvis ut offentlig tilgjengelig på nettsidene. Eksempelvis var det en åpen invitasjon til å komme med innspill til Handlingsplan for bedre kosthold. Totalt 226 forslag til tiltak ble spilt inn fra en rekke aktører/avsendere. Innspillene var tilgjengelige på Helsedirektoratets hjemmeside mens handlingsplanen ble utarbeidet.

STYRE 4 – tilgang til informasjon

**<u>God praksis-beskrivelse</u>**<br>**Myndighetene sikrer adgang til omfattende ernæringsinformasjon og nøkkeldokumenter (som budsjett-dokumenter, årlige resultatvurderinger og helseindikatorer) for offentligheten.**	
**Definisjoner og omfang**	• Inkluderer retningslinjer eller prosedyrer for å styre publisering av statlige budsjetter, resultatrapporter, revisjoner, evalueringsrapporter eller resultater fra andre gjennomganger eller henvendelser.<br>• Inkluderer "rett til informasjon"-/offentlighetslovgivning og liknende prosesser for å gi offentligheten adgang til statlig informasjon ved etterspørsel, med minst mulig restriksjoner og unntak.<br>• Inkluderer retningslinjer eller prosedyrer til å styre offentlig (nett-) publisering av helsedata som er samlet inn eller eid av myndighetene.
**Internasjonale eksempler på god praksis (referansemål)**	**Ireland:** The Freedom of Information Act 2014 came into effect in October 2014 and repealed the 1997 and 2003 Acts. The 2014 Act now applies to all public bodies, unless they are specifically exempt. It also allows for the Government to prescribe (or designate) other bodies receiving significant public funds, so that the FOI legislation applies to them also. The old legislation continues to apply to any FOI request that was made before the 2014 Act came into effect. It also applies to any subsequent review or appeal. It provides the following statutory rights, 1) A legal right for each person to access information held by a body to which FOI legislation applies known as an FOI body. 2) A legal right for each person to have official information relating to himself/herself amended where it is incomplete, incorrect or misleading. 3) A legal right for each person to obtain reasons for decisions affecting himself/herself (185).<br><br>**Norway:** The Freedom of Information Act grants everyone the right of access to case documents, journals and similar registers for any agencies encompassed by the Act. The Act applies to all government agencies, municipalities and county authorities. The general rule is that access shall be granted, and exceptions to this rule require legal authority prescribed by or pursuant to law. The Act also contains rules for handling right of access claims and the opportunity to appeal decisions in access matters (182).

Evidens for implementering

Som nevnt under STYRE 3, så har Norge lang tradisjon for **åpenhet i forvaltningen**. Norge har en egen offentlighetslov, og også en forvaltningslov og arkivlov (172, 185, 186).

Som konsekvens skal alle offentlige dokumenter, inkludert budsjetter, rapporter, revisjoner, evalueringsrapporter og resultater fra gjennomganger og henvendelser, være offentlig tilgjengelig. Det finnes unntak, og disse gjelder forsvarshemmeligheter, eksamensoppgaver og saker med taushetsplikt i forhold til personvern. Interne dokumenter, for eksempel møtereferater, fra offentlig forvaltning også er unntatt innsynsretten. Det er kun det endelige resultatet som offentliggjøres, med en begrunnelse.

I praksis legges mange dokumenter innen helseforvaltningen ut lett tilgjengelig på offentlige hjemmesider. Dette inkluderer møtereferat eksempelvis fra møter i intensjonsavtalens koordineringsgruppe. En del dokumenter gjøres tilgjengelig kun etter forespørsel. Eksempelvis er ikke evalueringen av MFU som ble gjennomført i 2017 av Helsedirektoratet lagt ut tilgjengelig på noen nettside, men den tilsendes de som etterspør den.

Det er også et uttalt mål om at helsedata skal bli mer tilgjengelig. Norske helseregistre er siden 2017 blitt gradvis samlet i en felles metadatabase (186). HOD sendte i juli på høring forslag til *Tilgjengeliggjøring av helsedata - forslag om endringer i helseregisterloven m.m.* med formål å gjøre helseopplysninger og andre helsedata i helseregistre mer tilgjengelig (187). En enklere tilgang til helsedata kan benyttes til statistikk, helseanalyser, forskning, kvalitetsforbedring, planlegging, styring og beredskap.

OMRÅDE 9: OVERVÅKING OG DATAINNSAMLING

Myndighetenes systemer for datainnsamling og monitorering (overvåking, evaluering, forskning og rapportering) er omfattende og jevnlige nok til å vurdere statusen til matomgivelser, ernæring i befolkningen og ikke-smittsomme sykdommer samt relaterte sosiale ulikheter, og til å måle fremdrift på oppnåelse av mål i offentlige planer for helse og kosthold.

Det er fem indikatorer for dette området:

- OVER 1 – overvåking av matomgivelser
- OVER 2 – overvåking av kosthold
- OVER 3 – overvåking av overvekt og fedme
- OVER 4 – overvåking av biologiske risikofaktorer og forekomst av NCDs
- OVER 5 – evaluering av planer og tiltak
- OVER 6 – overvåking av sosiale helsedeterminanter

OVER 1 – overvåking av matomgivelser

**God praksis-beskrivelse**<br>**Monitorerings-systemer som er etablert av myndighetene, overvåker jevnlig matomgivelser (særlig når det gjelder matens sammensetning for næringsstoffer som bør begrenses, markedsføring av mat mot barn, og kvaliteten på mat i skoler og andre offentlige steder) opp mot anbefalinger, regelverk og mål.**	
**Definisjoner og omfang**	• Omfatter overvåkningssystemer som er helt eller delvis finansiert av myndighetene og som forvaltes/ivaretas av en akademisk eller annen institusjon.<br>• Omfatter jevnlig overvåking og evaluering av effekten til tiltak som er implementert av myndighetene når det gjelder matomgivelser som beskrevet i politikkområdene 1-7, spesielt:<br>▪ Overvåking av mat- og serveringsbransjens overholdelse av frivillige mål/retningslinjer for næringsstoffer som bør begrenses (I området Sammensetning av matvarer).<br>▪ Overvåking av overholdelse av retningslinjer for matvaremerking (området Merking av mat).<br>▪ Overvåking av markedsføring av usunn mat til barn (området Markedsføring).<br>▪ Overvåking av overholdelse av retningslinjer for mattilbud i skoler, barnehager og offentlige sammenhenger (området Matomsorg og -tilbud).<br>▪ "Regelmessig" er vurdert til å være hvert femte år eller oftere.
**Internasjonale eksempler på god praksis (referansemål)**	**Many countries:** have food composition databases available. For example, the New Zealand Institute for Plant & Food Research Limited and the Ministry of Health jointly own the New Zealand Food Composition Database (NZFCD) which is a comprehensive collection of nutrient data in New Zealand containing nutrient information on more than 2600 foods (188).<br><br>**New Zealand:** A national School and Early Childhood Education Services (ECES) Food and Nutrition Environment Survey was organised in all Schools and ECES across New Zealand in 2007 and 2009 by the Ministry of Health to measure the food environments in schools and ECEs in New Zealand.<br><br>**UK:** In October 2005, the School Food Trust was established to provide independent support and advice to schools, caterers, manufacturers and others on improving the standard of school meals. They perform annual surveys, including the latest information on how many children are having school meals in England, how much they cost and how they're being provided.<br><br>**Germany:** The German Nutrition Report has been published by the German Nutrition Society (DGE) on behalf of the Federal Republic of Germany since 1969. Since 1972 it is provided every 4 years by directive of the Federal Ministry of Consumer Protection, Food and Agriculture. The subjects follow topics of current interest (189). The Robert-Koch-Institute, the government's

	scientific institution in the field of public health, started in 2015 the project 'AdiMon' that collects regular data on influencing (environmental) factors and prevalence of childhood obesity, as well as interventions for prevention and health promotion (190).<br><br>**The Netherlands:** the progress in product improvement of salt, saturated fat and calories (sugar and/or (saturated) fat) is monitored by the Dutch Instititute of Public Health and Environment (Rijksinstituut voor Volksgezondheid en Milieu (RIVM)) at product level. RIVM uses the product databank (levensmiddelendatabank (LEDA) as basis for which companies have to provide information about product contents (191).

Evidens for implementering

Generelt

Matvaretabellen er en database som gir en oversikt over innhold av energi og næringsstoffer for de vanligste matvarene i Norge. Tabellen inneholder 1721 matvarer per november 2019. Mattilsynet finansierer Matvaretabellen mens det praktiske arbeidet forvaltes av Mattilsynet og Avdeling for ernæringsvitenskap, Universitetet i Oslo (192). Midtveisevalueringen av handlingsplanen for sunnere kosthold trekker fram at det er behov for oppdatering av Matvaretabellen slik at endringer i produktutvikling kan vurderes (158).

Overvåking av matomgivelser

Intensjonsavtalen for et sunnere kosthold

Monitorering av avtalemålene er et eget innsatsområde i Intensjonsavtalen som alle partene forplikter å være med på. Nullpunkt for målene som er satt for reduksjon av sukker, mettet fett samt økning av inntaket av frukt og bær, grønnsaker, grove kornprodukter og sjømat er tall fra matforsyningsstatistikk 2015. Enkelte av målene vurderes også opp mot SSBs forbrukerundersøkelse fra 2012. Nullpunkt for saltinntak er basert på Norkost3 og SSB 2012. Data for monitorering av avtalen kommer fra matforsyningsstatistikk og SSBs forbruksundersøkelse 2012. Monitorering av saltmålene baserer seg på data fra Tradesolution. Helsedirektoratet er ansvarlig for å monitorere befolkningens inntak av næringsstoffene og matvarene som er dekket av intensjonsavtalen. Intensjonsavtalen blir også evaluert underveis av en tredjepart, forskningsstiftelsen Fafo. Følgerapporter for tiltak som er gjort av matvarebransjen i 2017 og 2018 ble publisert i 2019, basert på bransjens egenrapportering. Senere evalueringer skal også se på effekten av intensjonsavtalen på folkehelse (24). SSB er i gang med å utvikle en ny metode for forbruksundersøkelser som også skal benyttes for å monitorere intensjonsavtalen, men arbeidet er blitt forsinket (193).

Merking av mat

Bransjen har selv ansvar for å sette seg inn i og følge regelverket for matmerking. Mattilsynet fører også tilsyn med at næringsmidler er merket i samsvar med regelverket. Dette gjelder både generell merking (ingrediensliste og næringsdeklarasjon) og ernærings- og helsepåstander, samt merking med Nøkkelhullet. Mattilsynet kan fatte vedtak ved brudd på regelverket. Mattilsynet har gjennomført ett nasjonalt tilsynsprosjekt om bruk av ernærings- og helsepåstander, i 2014 (37). Tilsyn med Nøkkelhullsordningen skjer ifølge Mattilsynet ved revisjon, inspeksjon ved enkeltstående tilsyn eller som et ledd i større tilsynskampanjer (194).

Markedsføring av mat
Det foreligger ikke noen systematisk monitorering av omfanget av markedsføring av usunn mat rettet mot barn i Norge, eller av hvordan matbransjen overholder retningslinjene i Matbransjens faglige utvalg (MFU). Helsedirektoratet evaluerte MFU i 2016. I forbindelse med evalueringen ble det gjennomført en SIFO-kartlegging av markedsføring av usunn mat rettet mot barn og unge på TV og Internett. Internett-reklamen ble kartlagt ved at 10 ungdommer sendte inn skjermdumper av reklame på nettsider og sosiale medier som de besøkte. Helsedirektoratet konkluderte blant annet med at det var nødvendig med jevnlig overvåking av markedsføring.

I 2018 utarbeidet de nordiske landene i samarbeid med internasjonale eksperter en protokoll for kartlegging av markedsføring av usunn mat og drikke rettet mot barn. Det foreligger så langt ingen studier som har anvendt denne protokollen i Norge (195).

I 2019 ble MFU-ordningen evaluert av Folkehelseinstituttet. Denne gangen ble det ikke gjennomført noen kartlegging av markedsføring. Evalueringen var ikke ferdig da dette evidensdokumentet ble ferdigstilt.

I 2020 skal SIFO gjennomføre en kartlegging av usunn matreklame på digitale medier som rettes mot barn opptil 18 år i Norge. For å samle inn data skal det brukes en app som (med samtykke) samler inn store mengder data på tvers av digitale plattformer som barn bruker. Prosjektet finansieres av WHOs Europakontor samt Barne- og familiedepartementet og Helse og Omsorgsdepartementet. Det er også planlagt en komparativ rapport med alle medlemslandene i regi av WHO.

Matomsorg og -tilbud
Det foregår ingen regelmessig kartlegging av retningslinjer for skolemåltid. I nasjonal handlingsplan for bedre kosthold er et av oppfølgingspunktene å innlemme spørsmål om mat og måltider i en årlig undersøkelse som går ut til alle barnehager, Spørsmål til Barnehage-Norge (99).

OVER 2 – overvåking av kosthold

<table>
<tr><td colspan="2"><u>God praksis-beskrivelse</u>
Det foregår regelmessig overvåking av barn og voksnes ernæringsstatus og befolkningens kosthold opp mot spesifikke mål/grenseverdier for inntak eller anbefalt daglig inntak.</td></tr>
<tr><td>Definisjoner og omfang</td><td>• Omfatter overvåking av kosthold hos barn og voksne for å vurdere samsvar med myndighetenes kostholdsråd.
• Omfatter overvåking av næringsstoffinntak når det gjelder næringsstoff som bør begrenses og av usunne matvaregrupper som søtet drikke, også hvis det ikke foreligger inntaksmål for disse.
• "Regelmessig" er vurdert til å være hvert femte år eller oftere.</td></tr>
<tr><td>Internasjonale eksempler på god praksis (referansemål)</td><td>USA: The National Health and Nutrition Examination Survey (NHANES) is a program of studies designed to assess the health and nutritional status of adults and children in the United States. The survey is unique in that it combines interviews and physical examinations. The NHANES program began in the early 1960s and has been conducted as a series of surveys focusing on different population groups or health topics. In 1999, the survey became a continuous program that has a changing focus on a variety of health and nutrition measurements to meet emerging needs. The survey examines a nationally representative sample of about 5,000 persons each year. These persons are located in counties across the country, 15 of which are visited each year (196).

The Netherlands: The Dutch Institute of Public Health and Environment (Rijksinstituut voor Volksgezondheid en Milieu (RIVM)) is appointed by the Ministry of Health to periodically collect data about the food consumption and food condition of the Dutch population in general and of separate population groups via the Food Consumption Survey. Currently, a Food Consumption Survey (Dutch population 1-79 years) is being conducted for the years 2019-2021. Prior Food Consumption Surveys have been conducted for the years 2012-2016 (Dutch population 1-79 years), 2010-2012 (elderly 70+), 2007-2010 (7-69 years), 2005-2006 (2-6 years), 2003 (9-16 years) (197).</td></tr>
</table>

Evidens for implementering

Kostholdsundersøkelsene

Kostholdsundersøkelsene måler inntaket av mat og drikke i landsrepresentative utvalg av befolkningen og gir data for inntak av energi og næringsstoffer (198). Siden 1992 er det gjennomført åtte slike undersøkelser i ulike aldersgrupper:

- Spedkost (0–12 måneder) og Småbarnskost (2 år) ble gjennomført i 1998-99 og i 2006-07. Nye undersøkelser med både Spedkost og Småbarnskost er under gjennomføring og resultatene forventes i 2020 (199, 200).
- Ungkost (4, 9 og 13 år) ble gjennomført i 1993, 2000 og 2015-16
- Norkost (18–70 år) som ble gjennomført i 1993, 1997 og 2010-11.

Regionale helseundersøkelser
Tromsøundesøkelsen og Helseundersøkelsen i Nord-Trøndelag (HUNT) er store befolkningsundersøkelser som gjennomføres regelmessig. Begge undersøkelsene gir opplysninger om kosthold og brukes blant annet til å estimere inntak av salt (Tromsøundersøkelsen), mettet fett og inntak av frukt og grønnsaker. Tromsøundersøkelsen ble sist gjennomført i 2015-16 og HUNT ble gjennomført i 2017-19 (201, 202).

Annet
HEVAS er en landsdekkende spørreskjemaundersøkelse blant norske 11-, 13-, 15- og 16-åringer som gjennomføres hvert fjerde år. Formålet er å kartlegge faktorene som bidrar til god utvikling i helse, trivsel og læring blant barn og unge. Spørreskjemaet inneholder fem spørsmål knyttet til kostholdsvaner og måltidsvaner, blant annet hvor ofte man spiser frukt, grønnsaker og godteri.

Matforsyningsstatistikken gir informasjon om den totale omsetningen av matvarer i landet og viser den mengden av ulike matvaregrupper som står til rådighet for hele befolkningen. Statistikken er basert på tall for produksjon og import av matvarer minus eksport. Matforsyningsstatistikken er utgitt årlig siden 1950-årene og presenteres i den årlige rapporten Utviklingen i norsk kosthold som utgis av Helsedirektoratet.

Statistisk sentralbyrås (SSB) ***forbruksundersøkelser*** viser hvor mye mat som blir anskaffet (dvs. kjøpt, tatt av egen produksjon, fått som gave o.l.) av et representativt utvalg av privathusholdninger i en 14 dagers periode. Undersøkelsen ble gjennomført årlig i perioden 1975–2009 og i 2012 og resultater fra disse undersøkelsene er presentert i rapporten Utviklingen i norsk kosthold.

Kommentarer

Rapportering om kosthold til den globale og nasjonale NCD-strategien baserer seg på tall fra Norkost 2010-11 sammen med data fra matforsyningsstatistikk. Saltinntak er basert på Tromsøundersøkelsen (203).

I midtveisevalueringen av handlingsplanen for bedre kosthold kommer det fram at mangel på kostholdsdata gjør det vanskelig å evaluere framgangen i å nå målene i handlingsplanen. Utvalget ønsker bedre og hyppigere data om kostholdet til befolkningen i Norge og utviklingen av kostholdet til befolkingen i Norge, samt data om undergrupper utover alder og kjønn (158).

OVER 3 – overvåking av overvekt og fedme

**God praksis-beskrivelse**<br>**Det foregår regelmessig overvåking av forekomst av overvekt og fedme blant barn og voksne ved bruk av antropometriske mål.**	
**Definisjoner og omfang**	• Antropometriske mål inkluderer høyde, vekt og livomkrets.<br>• "Regelmessig" er vurdert til å være hvert femte år eller oftere.
**Internasjonale eksempler på god praksis (referansemål)**	**UK:** England's National Child Measurement Programme was established in 2006 and aims to measure all children in England in the first (4-5) years and last (10-11 years) of primary school. In 2011-2012, 565 662 children (4-5 years) and 491118 children (10-11 years) were measured (204).<br><br>**Ireland:** The 4th Childhood Obesity Surveillance Initiative report was launched in 2017. COSI collects data from children in primary schools in the Republic of Ireland. The survey is carried out periodically. Data was first collected from children in 2008 in first class and again in 2010 from first class and third class, in 2012 from first, third and fifth classes and in 2015 from first, fourth and sixth class. Trained researchers collected weight, height and waist circumference measurements. These figures were used to examine prevalence of normal weight, overweight, obesity and mean BMI (205).

Evidens for implementering

Overvåking av overvekt og fedme blant barn gjøres gjennom den landsrepresentative Barnevekststudien. For voksne finnes det ikke nasjonale tall. Følgende studier bidrar med data om overvekt og fedme i Norge:

- Barnevekststudien (8-9-åringer) er en del av overvåkningsprogrammet WHO European Childhood Obesity Surveillance Initiative (COSI), og har så langt blitt gjennomført i 2008, 2010, 2012, 2015 og 2019.
- Nasjonal kartlegging av fysisk aktivitet UngKAN – (6-, 9- og 15-åringer) gir også nasjonale data på overvekt og fedme
- Helseundersøkelsen i Nord-Trøndelag (HUNT) (ungdom, voksne) har blitt gjennomført omtrent en gang i tiåret siden 1984-86, siste runde var i 2017-2019.
- Tromsøundersøkelsen (ungdom og voksne) har blitt gjennomført sju ganger siden 1974, de siste rundene var i 2007-08 og 2015-16. En ny runde planlegges i 2021.

I Barnevekststudien, HUNT og Tromsøundersøkelsen blir vekt og høyde målt av helsepersonell.

Kommentarer

Rapportering om overvekt og fedme til den nasjonale og globale NCD-strategien baserer seg på data fra Barnevekststudien og UngKAN for barn, og Tromsøundersøkelsen og HUNT for voksne (203).

Den nasjonale NCD-gruppen skriver i sin rapport om utvikling av NCD-indikatorer at det er et klart behov for nasjonalt representativ datainnsamling for vekt/fedme innen nasjonal helseanalyse, og at data ideelt sett skulle vært hentet fra undersøkelser i nasjonalt representative utvalg (206).

OVER 4 – overvåking av biologiske risikofaktorer og forekomst av NCDs

<table>
<tr><td colspan="2"><u>God praksis-beskrivelse</u>
Det foregår regelmessig overvåking av forekomsten av biologiske risikofaktorer for ikke-smittsomme sykdommer, samt forekomst, insidens og dødelighet av de viktigste kostholdsrelaterte, ikke-smittsomme sykdommene.</td></tr>
<tr><td>Definisjoner og omfang</td><td>• Kostholdsrelaterte NCDs inkluderer bl.a. høyt blodtrykk, hyperkolesterolomi, diabetes type 2, hjerte-karsykdommer (inkludert iskemisk hjertesykdom, cerebrovaskulær sykdom og andre karsykdommer) og kostholdsrelaterte kreftformer.
• "Regelmessig" er vurdert til å være hvert femte år eller oftere.
• Kan samles gjennom ulike mekanismer slik som befolkningsundersøkelser eller overvåkningssystemer for spesifikke sykdommer.</td></tr>
<tr><td>Internasjonale eksempler på god praksis (referansemål)</td><td>OECD Countries: Most of those countries have regular and robust prevalence, incidence and mortality data for the main diet-related NCDs and NCD risk factors.</td></tr>
</table>

Evidens for implementering

En rekke registre og datakilder er relevante i forbindelse med NCD-situasjonen i Norge. Nasjonal NCD-gruppe har utviklet indikatorer for rapportering av oppnåelse opp mot den globale og nasjonale NCD-strategien, og legger følgende kilder til grunn (206):

- **For tidlig død av NCDs**: Dødsårsaksregisteret (DÅR) har medisinske opplysninger om 98% av dødsfallene i Norge. Sammen med Folkeregisteret brukes DÅR som kilde til å rapportere summen av NCD-dødsfall.
- **Insidens av NCDs:** hjerte- og karregisteret og CVDNOR-prosjektet kan i kombinasjon brukes til å gi pålitelige tall for insidens av de to største gruppene av hjerte- og karsykdommer. I tillegg til disse kan Norsk pasientregister, Kontroll av utbetaling og helserefusjon- KUHR, Reseptregisteret og Kreftregisteret samt til en viss grad Nasjonalt register for kronisk obstruktiv lungesykdom (KOLS) brukes til å estimere nye NCD-tilfeller av de ulike sykdommene. Tromsøundersøkelsen og HUNT kan gi et estimat av kjent og ikke-kjent diabetes.
- **Metabolske risikofaktorer:**
    - *Høyt blodtrykk:* Det foreligger ikke nasjonale data med blodtrykksmålinger i Norge. Det finnes regionale data fra Tromsøundersøkelsen og HUNT. Reseptregisteret kan benyttes for å finne data for medisinsk behandlet hypertensjon.
    - *Høyt blodsukker:* De siste rundene av Tromsøundersøkelsen og HUNT har målt langtidsblodsukker (HbA1C).
    - *Kolesterol:* Det finnes ikke nasjonale data for kolesterol. Tromsøundersøkelsen og HUNT gir regionale data.

Overvåking av NCD-indikatorene mot de ni globale målene, presenteres i en nettpublikasjon utarbeidet av NCD-gruppen (203). Gruppen bidrar på den måten til at HOD kan rapportere på ulike indikatorer nasjonalt og internasjonalt.

Kommentarer

Ifølge den nasjonale NCD-gruppen mangler det norske nasjonale data på relevante NCD-risikofaktorer som blodtrykk, kolesterol, blodsukker og høy samlet risiko for hjerte- og karsykdom i Norge. NCD-gruppen peker på behovet for nasjonal datainnsamling. Tatt høyde for utfordringer knyttet til befolkningens deltakelse i nasjonale datainnsamlinger, foreslår gruppen at nye studier i Osloområdet og i Hordaland (som ville bygge på tidligere studier i disse områdene) kunne være gode supplement til Tromsøundersøkelsen og HUNT, slik at data samlet sett bedre ville ha representert landet enn de nå gjør (206).

OVER 5 – evaluering av planer og tiltak

<table>
<tr><td colspan="2"><u>God praksis-beskrivelse</u>
Sentrale planer og programmer evalueres regelmessig for å vurdere deres effektivitet og bidrag til å nå oppsatte mål.</td></tr>
<tr><td>Definisjoner og omfang</td><td>• Inkluderer alle policies, rammeverk, retningslinjer eller verktøy som brukes for å bestemme hensiktmessig/riktig metode og rapporteringsform for evalueringen.
• Inkluderer et omfattende evalueringsrammeverk og -plan som samsvarer med viktige implementeringsplaner for helse- eller ernæring.
• Hva som regnes som "viktige" planer skal defineres av myndighetene selv via relevant departement.
• Evaluering bør skje i tillegg til rutinemessig oppfølging av gjennomføring/måloppnåelse opp mot en handlingsplan.</td></tr>
<tr><td>Internasjonale eksempler på god praksis (referansemål)</td><td>USA: The National Institutes for Health (NIH) provide funding for rapid assessments of natural experiments. The funding establishes an accelerated review/award process to support time-sensitive research to evaluate a new policy or program expected to influence obesity related behaviours (e.g., dietary intake, physical activity, or sedentary behaviour) and/or weight outcomes in an effort to prevent or reduce obesity (207).

The Netherlands: The Dutch Institute of Public Health and Environment (Rijksinstituut voor Volksgezondheid en Milieu) conducted in 2017 a midterm evaluation to calculate the effect of the agreed maximum norms for salt and sugar in the Agreement on Product Improvement. The evaluation presented results from four different scenarios depending on the level and reach of use by the food industry using the Food Consumption Survey (208).

Ireland: Under the 'Healthy Weight for Ireland' Obesity Policy and Action Plan 2016-2025, a new Obesity Policy Implementation Oversight Group (OPIOG) was established in October 2017 and a progress report on each recommendation in the OPAP is currently being finalised under the aegis of the OPIOG. As set out in Healthy Ireland, integrated health and social impact assessments will be conducted on relevant policy areas to support other government departments in contributing towards the prevention of overweight and obesity (151).</td></tr>
</table>

Evidens for implementering

Nasjonal handlingsplan for bedre kosthold ble midtveisevaluert i 2019 (158). Intensjonsavtalen for sunnere kosthold skal midtveisevalueres av Fafo i 2020 (24). Koordineringsgruppen for intensjonsavtalen utarbeider status for arbeidet i intensjonsavtalen i perioden 2016-2019.

OVER 6 – overvåking av sosiale helsedeterminanter

**God praksis-beskrivelse** **Det foregår overvåking av sosiale og økonomiske helsedeterminanter (påvirkningsfaktorer) og av fremdrift for å redusere sosiale ulikheter eller helseutfall i sårbare grupper.**	
**Definisjoner og omfang**	• Overvåking av overvekt og fedme samt viktige kostholdsrelaterte NCDs inkluderer stratifisiering eller analyse av de befolkningsgruppene der det er størst helseforskjeller, inkludert sosioøkonomiske strata og minoritetsgrupper.<br>• Inkluderer å rapportere opp mot konkrete mål eller indikatorer som er knyttet til sosiale helseforskjeller.
**Internasjonale eksempler på god praksis (referansemål)**	**New Zealand**:<br>All annual Ministry of Health Surveys report health outcomes estimates by subpopulations in particular by ethnicity (including Maori and Pacific peoples), by age, by gender, and by New Zealand area of deprivation.

Evidens for implementering

Sosial ulikhet i helse er et satsingsområde for helsemyndighetene og for Folkehelseinstituttet som er ansvarlig for monitorering av kostholdsområdet. Relevante datakilder for NCD-området inkludert forskningsprosjekter rundt kosthold, overvekt/fedme, biologiske risikofaktorer og NCD-sykelighet og -dødelighet samler inn data om sosioøkonomisk posisjon (SEP) og presenterer resultater blant annet etter dette. For eksempel viser de nasjonale kostholdsundersøkelsene data om kosthold presentert i forhold til SEP målt ved utdanningslengde. Data om sosial ulikhet i helse og kosthold presenteres blant annet i Helsedirektoratets Folkehelsepolitisk rapport (174) og i Folkehelseinstituttets Folkehelserapport (175). I 2019 presenterte den nasjonale NCD-gruppen tall for sosial ulikhet i helse der også data for kosthold, overvekt og fedme ble presentert (209). Her ble også data for utvalg fra innvandrerbefolkningen presentert.

Kommentarer

Midtveisevalueringen til handlingsplanen for bedre kosthold kommenterer at det på bakgrunn av datagrunnlaget er vanskelig å si noe om endringen i inntaket til spesifikke grupper av befolkningen. Det påpekes videre at det mangler kunnskap om hvordan konkrete virkemidler (som Nøkkelhullsordningen og intensjonsavtalen) påvirker grupper med lav sosioøkonomisk status (158).

OMRÅDE 10: FINANSIERING OG RESSURSER

*Det er tilstrekkelig finansiering av offentlige tiltak for å fremme god ernæring i befolkningen til å bidra til sunne matomgivelser, bedre ernæring i befolkningen og redusere overvekt, fedme og kostholdsrelaterte NCDs og relaterte sosiale ulikheter.*5

Kommentar: Det er i utgangspunktet tre indikatorer for dette området. Indikator 1 og 2 vil imidlertid ikke bli vurdert i Nettskjema. Dette er fordi både datainnsamling og sammenlikning mellom land er utfordrende for disse indikatorene. Det blir likevel mulighet for å diskutere budsjett og finansiering i workshop'en, og våre funn for indikatorene blir derfor presentert her.

- FINANS 1 – budsjett som går til ernæring
- FINANS 2 – finansiering av forskning rundt fedme og NCD-forebygging
- FINANS 3 – helsefremmende myndighetsorgan

[5] *Estimert ut fra investeringer i tiltak som skal fremme og promotere sunt kosthold og sunne matomgivelser for å forebygge fedme og kostholdsrelaterte NCDs, men unntatt individuelle tiltak (i primærhelsetjeneste, svangerskapsomsorg, helsestasjon og liknende), og unntatt tiltak knyttet til mattrygghet, mangel på mikronæringsstoffer (for eksempel jodberikning) og underernæring.*

FINANS 1 – budsjett som går til ernæring

Denne indikatoren skal ikke vurderes i Nettskjema – står kun her som informasjon

<table>
<tr><td colspan="2"><u>God praksis-beskrivelse</u>
Budsjettet som går til tiltak for å fremme god ernæring i befolkningen, vurdert som andelen av det totale helsebudsjettet eller relatert til byrden av kostholdsrelaterte NCDs, er tilstrekkelig til å bidra til å redusere kostholdsrelaterte NCDs.</td></tr>
<tr><td>Definisjoner og omfang</td><td>
• "Fremme god ernæring i befolkningen" inkluderer promotering av sunt kosthold samt policies, tiltak og programmer som støtter sunne matomgivelser for å forebygge overvekt og kostholdsrelaterte NCDs.
• Omfatter estimater for budsjettet som er satt av til myndighetsavdelingen som har det primære ansvaret for ernæring, inkludert utgifter til ansatte.
• "Ansatte" utgjør alle som har en primær rolle knyttet til ernæring i befolkningen uavhengig av stillingsbrøk eller type ansettelse, inkludert konsulenter eller finansiering av en stilling i et annet departement.
• "Det totale helsebudsjettet" innebærer hele budsjettet for Helse- og omsorgsdepartementet for siste tilgjengelige budsjettår.

• Indikatoren omfatter ikke tiltak og tjenester på individnivå (primærhelsetjenesten, helsestasjon, svangerskapsomsorg og liknende) samt mattrygghet, underernæring og mangel på mikronæringsstoff.
• Omfatter ikke deler av budsjett som er satt av til å stimulere fysisk aktivitet.</td></tr>
<tr><td>Internasjonale eksempler på god praksis (referansemål)</td><td>New Zealand: The total funding for population nutrition was estimated at about $67 million or 0.6% of the health budget during 2008/09 Healthy Eating Healthy Action period. Dietary risk factors account for 11.4% of health loss in New Zealand.

Thailand: According to the most recent report on health expenditure in 2012 the government greatly increased budget spent on policies and actions related to nutrition (excluding food, hygiene and drinking water control). Total expenditure on health related to nutrition specifically from local governments was 29,434.5 million Baht (7.57% of total health expenditure from public funding agencies), which was ten times over the budget spending on nutrition in 2011. Dietary risk factors account for more than 10% of health loss in Thailand.</td></tr>
<tr><td>Kontekst</td><td>Folkehelseloven regulerer ansvarsområdet når det gjelder å fremme et sunt kosthold i befolkningen. Det er Helsedirektoratet som har ansvar for å implementere slike tiltak nasjonalt, i henhold til politiske føringer. Kommunene har ansvar for å iverksette tiltak lokalt i henhold til lokale behov og ressurser.</td></tr>
</table>

Evidens for implementering

Stortingsproposisjon 1 S fra Helse- og omsorgsdepartementet angir budsjettpostene som er relevante for folkehelse (programkategori 10.10) og for ernæringsrelaterte tiltak (210).

I budsjettet for budsjettåret 2020 er det planlagt å bruke om lag 457 millioner til folkehelse, og dette er en økning på 3% sammenlignet med forbruket i 2019. Dette utgjør 0,2% av det totale budsjettet til Helse- og omsorgsdepartementet. I budsjettet for 2020, kapittel 714 Folkehelse, angis budsjett for ulike ernæringstiltak for budsjettåret 2020:

- Skolefrukt: 20,6 mill
- Kommunikasjon om levevaner og helse: 27,7 mill
- Kostholdstiltak for å støtte opp under handlingsplanen for bedre kosthold: 7 mill
- Driftstilskudd Ammehjelpen: 1,5 mill
- Driftsstøtte til Norges sjømatråd til det nasjonale kostholdsprogrammet Fiskesprell: 1 mill

Når det gjelder Fiskesprell, så bidrar både Nærings- og fiskeridepartementet med 4 mill og Sjømatrådet med 4 mill hver, i tillegg til HODs bidrag (tall fra 2019).

I tillegg er det avsatt midler til kommunale tiltak på 94,8 mill hvorav noe potensielt kan benyttes til ernæringstiltak.

Helsedirektoratets totale budsjett for 2020 er 1,4 milliarder kr, opp fra 1,37 milliarder i 2019. Kosthold og ernæring er ikke spesifikt nevnt under budsjettposter til Helsedirektoratet, men en del av de ansatte ved Helsedirektoratet har ansvarsområder knyttet til å fremme kosthold og ernæring.

FINANS 2 – finansiering av forskning rundt fedme og NCD-forebygging

Denne indikatoren skal ikke vurderes i Nettskjema – står kun her som informasjon

<table>
<tr><td colspan="2"><u>God praksis-beskrivelse</u>
Myndighetene finansierer forskning som er rettet mot å bedre matomgivelser og å redusere overvekt og fedme samt NCDs og relaterte sosiale ulikheter.</td></tr>
<tr><td>Definisjoner og omfang</td><td>• Inkluderer en klar identifisering av forskning knyttet til forbedring av matomgivelser, reduksjon av overvekt og NCDs og relaterte sosiale ulikheter, og en prioritering av disse i forskningsstrategier eller rammeverk innen helse eller medisin.
• Innebærer forskningsprosjekter som er utført av eller bestilt av myndighetene, som spesifikt retter seg mot matomgivelser, og/eller forebygging av overvekt/fedme/NCDs (ekskludert sekundær- eller tertiærforebygging).
• Inkluderer forskningsprosjekter som er godkjent eller utført i løpet av det siste året.
• Omfatter ikke evaluering av intervensjoner (dette er dekket av OVER5 og bør være del av et overordnet programbudsjett).</td></tr>
<tr><td>Internasjonale eksempler på god praksis (referansemål)</td><td>Australia: The National Health and Medical Research Council (NHMRC) Act requires the CEO to identify major national health issues likely to arise. The National Health Priority Areas (NHPAs) articulate priorities for research and investment and have been designated by Australian governments as key targets because of their contribution to the burden of disease in Australia. For the 2015-16 Corporate Plan, obesity, diabetes and cardiovascular health are three of these NHPAs.

Thailand: The National Research Council funded more research projects on obesity and diet-related chronic diseases (such as diabetes, cardiovascular diseases and hypertension) in 2014, accountable for almost six times over the research funding in 2013 (from 6,875,028 baht in 2013 to 37,872,416 baht in 2014)

Ireland: The Food Institutional Research Measure (FIRM) is funded by the Department of Agriculture, Food and the Marine and is the primary national funding mechanism for food research in higher education institutions and other public research institutes. Beneficiaries are required to widely disseminate the results of their research. FIRM aims to develop public good technologies that will underpin a competitive, innovative and sustainable food manufacturing and marketing sector. The programme is creating a base of knowledge and expertise in generic technologies that will support a modern, consumer-focused industry and build Ireland's capacity for R&D (211). The Health Research Board (HRB) is a statutory agency under the aegis of the Department of Health. It's the lead agency in Ireland responsible for supporting and funding health research, information and evidence, which aims to improve people's health and to enhance healthcare delivery (212).</td></tr>
</table>

**Kontekst**	Forskning innen mat, kosthold, ernæring og helse skjer i hovedsak gjennom Norges forskningsråds programmer. Forskningsrådets programmer for forebygging og folkehelse Bedre helse og livskvalitet (BEDREHELSE), Behandlingsrettet forskning (BEHANDLING) og Helse- og omsorgstjenesteforskning (HELSEVEL) skal fremme forskning og forskningsbasert innovasjon på ulike områder, inkludert ernæring, mat og helse (99). HOD bevilger mesteparten av forskningsmidlene innen helseprogrammene og gir overordnede føringer, mens Norges forskningsråd administrerer forskningsmidlene.

Evidens for implementering

I HODs tildelingsbrev til Forskningsrådet for 2019 vises det til at midlene fra HOD skal bidra til å oppfylle overordnede forskningspolitiske målsettinger inkludert å løse store samfunnsutfordringer. Overvekt, kosthold og ernæring er ikke nevnt i tildelingsbrevet (213).

Programmet BedreHelse skal bidra til forskning for bedre helse, økt livskvalitet og redusert sosial ulikhet i helse og inkluderer bl.a. forskning innenfor psykisk helse, fysisk aktivitet, rus og ernæring, og er det mest relevante programmet her.

Ved gjennomgang av Forskningsrådets prosjektbank (214), er det per desember 2019 to større pågående forskningsprosjekter som er finansiert gjennom BedreHelse, som dreier seg om overvekt og fedme:

- *Tackling socioeconomic differences in weight development among youth: assessing trends, mechanisms and potential intervention* (15 mill), UiO
- *Evaluation of free school fruit on childhood growth and obesity: a natural experiment* (10 mill), Folkehelseinstituttet

To av tre institusjonsforankrede strategisk prosjekt (ISP) finansiert gjennom BedreHelse i 2018, gikk til prosjekter som omhandler sosial ulikhet i helse: *Healthy choices and the social gradient* (50 mill) ved Universitetet i Tromsø og *Centre for Global Health Inequalities Research (CHAIN)* (36,8 mill) ved Universitetet i Bergen.

Andre relevante pågående prosjekter finansiert gjennom Forskningsrådet er følgende:

- *Økt fysisk aktivitet og sunnere livsstil hos Innvandrerkvinner* (7 mill) som er finansisert gjennom ordningen Innovasjonsprosjekt i offentlig sektor, UiO
- *Inequalities in non-communicable diseases: Indirect selection or social causation?* (10 mill), Frimedbio, UiO
- *The burden of obesity in Norway: morbidity, mortality, health service use and productivity loss* (7 mill), Frimedbio, FHI
- *Skolen som folkehelseaktør: Effektive verktøy for god implementering av retningslinjer for mat og måltider* (1,6 mill) offentlig PhD, Helsedirektoratet
- *Contextual interventions influence on food choices in a real-life grocery store setting* (1,7 mill), nærings-PhD, GreeNudge

Handlingsplanen for bedre kosthold har som oppfølgingspunkt å be Norges forskningsråd vurdere å videreføre felles utlysning for programmene BEDREHELSE og BIONÆR og eventuelt andre relevante programmer. Endelig beslutning er tillagt programstyrene (99).

Norge deltar i tre europeiske samarbeidsprogrammer – Joint Programming Initiatives (JPI): A Healthy Diet for a Healthy Life (JPI HDHL), Healthy and Productive Seas and Oceans (JPI Oceans) og JPI Agriculture, Food Security and Climate Change (JPI FACCE). Programmene har gått sammen om en tverrfaglig tilnærming til mat, matproduksjon, ernæring og helse. Hvert land finansierer egen deltakelse. Norsk deltakelse i JPI HDHL administreres av BedreHelse-programmet. To ulike forskningsgrupper deltar i HDHL-prosjektet *Policy Evaluation Network* (PEN) i perioden 2019-2022, med en støtte på til sammen 4.4 mill NOK (dette Food-EPI forskningsprosjektet er ett av de to PEN-prosjektene).

FINANS 3 – helsefremmende myndighetsorgan

<u>God praksis-beskrivelse</u><br>**Det finnes et lovfestet helsefremmende myndighetsorgan som inkluderer et formål om å bedre ernæringstilstanden i befolkningen og som har en sikker finansiering.**	
**Definisjoner og omfang**	• Organet ble etablert gjennom lovgivning.<br>• Inkluderer et mål om å bedre befolkningens ernæring gjennom relevant lovgivning, strategiske planer eller på organets nettside.<br>• En sikker finansiering innebærer bruk av lovmessig, øremerket skattlegging eller annen sikker finansiering.
**Internasjonale eksempler på god praksis (referansemål)**	**Australia:** The Victorian Health Promotion Foundation (VicHealth) was the world's first health promotion foundation, established by the Victorian Parliament as part of the Tobacco Act of 1987 (for the first 10 years through a hypothecated tobacco tax) through which the objectives of VicHealth are stipulated. VicHealth continues to maintain bipartisan support.<br><br>**Germany:** The Federal Centre for Health Education and the Federal Center for Nutrition disseminate guidelines and health promotion strategies to the general public and stakeholders and multipliers (215).<br><br>**The Netherlands:** The Dutch Nutrition Center is 100% funded by the government and offers consumers and professionals scientific and independent information about a healthy, safe and sustainable food choice. The famous 'Wheel of Five' Guidelines is one of their products (216).
**Kontekst**	Folkehelseloven regulerer ansvarsområdet når det gjelder å fremme et sunt kosthold i befolkningen (110). Det er Helsedirektoratet som har ansvar for å implementere slike tiltak nasjonalt, i henhold til politiske føringer. Kommunene har ansvar for å iverksette tiltak lokalt i henhold til lokale behov og ressurser.

Evidens for implementering

Helsedirektoratet og dets arbeidsoppgaver er lovfestet gjennom ulike lover. I folkehelselovgivningen (110) er Helsedirektoratets ansvar innen folkehelsearbeid fastsatt. Paragraf 24 sier:

- Helsedirektoratet skal følge med på forhold som påvirker folkehelsen og bidra til å iverksette nasjonal politikk på folkehelseområdet og være en pådriver for kunnskapsbasert folkehelsearbeid, blant annet gjennom utvikling av nasjonale normer og standarder for godt folkehelsearbeid.
- Helsedirektoratet skal gi kommuner, fylkeskommuner, fylkesmenn og andre statlige institusjoner, helsepersonell og befolkningen informasjon, råd og veiledning om strategier og tiltak i folkehelsearbeidet. Direktoratet skal videre samarbeide med Folkehelseinstituttet om å gjøre tilgjengelig opplysninger om helsetilstand og påvirkningsfaktorer etter § 25.

Det står ikke eksplisitt i folkehelseloven at kosthold og ernæring inngår i «forhold som påvirker folkehelsen», men både tildelingsbrev fra HOD (217), Helsedirektoratets strategi (218) og satsing innen kostholdsfeltet viser at ernæringsarbeid er sentralt i Helsedirektoratets arbeid. Helsedirektoratets totale budsjett for 2020 er 1,4 milliarder kr, opp fra 1,37 milliarder i 2019. Kosthold og ernæring er ikke spesifikt nevnt under budsjettposter til Helsedirektoratet, men en del av de ansatte ved Helsedirektoratet har ansvarsområder knyttet til å fremme kosthold og ernæring. Regjeringen innførte Avbyråkratiserings- og effektiviseringsreformen (ABE-reformen) i 2015, og har siden kuttet budsjettet til alle statlige virksomheter med 0,5 prosent hvert år (219).

OMRÅDE 11: SAMHANDLINGSMEKANISMER

Det finnes koordineringsmekanismer og muligheter for synergier på tvers av regjeringsavdelinger, myndighetsnivåer og andre sektorer (frivillige organisasjoner, privat sektor og akademia) slik at politikk og tiltak innen mat og ernæring er sammenhengende og effektive for å forbedre matomgivelser, ernæring i befolkningen, kostholdsrelaterte NCDs og relaterte ulikheter.

Det er fire indikatorer for dette området:

- SAMHA 1 – koordineringsmekanismer hos myndighetene
- SAMHA 2 – samarbeid mellom myndighetene og matbransjen
- SAMHA 3 – samarbeid mellom myndighetene og sivilsamfunn
- SAMHA 4 – systembasert tilnærming med lokale organisasjoner

SAMHA 1 – koordineringsmekanismer hos myndighetene

<table>
<tr><td colspan="2"><u>God praksis-beskrivelse</u>
Det finnes robuste koordineringsmekanismer på tvers av departementer og myndighetsnivåer (nasjonalt og lokalt) for å sikre sammenheng og samordning av forebyggingspolitikk for kosthold, overvekt og fedme og ikke-smittsomme sykdommer.</td></tr>
<tr><td>Definisjoner og omfang</td><td>• Omfatter strukturer på tvers av regjering eller departementer, komiteer eller arbeidsgrupper (på ulike ledernivåer), avtaler, intensjonsavtaler og liknende.
• Omfatter prioriteringer eller mål som er delt på tvers av departement eller regjering.
• Omfatter strategier eller rammeverk som kartlegger integrasjon og justering av politikk eller programmer på tvers av regjering og departementer.
• Omfatter felles, tverrdepartementale prosesser for planlegging, implementering eller rapportering samt konsultasjonsprosesser for utvikling av ny politikk/tiltak eller revisjon av eksisterende tiltak.</td></tr>
<tr><td>Internasjonale eksempler på god praksis (referansemål)</td><td>Finland: The Finnish National Nutrition Council is an inter-governmental expert body under the Ministry of Agriculture and Forestry with advisory, coordinating and monitoring functions. It is composed of representatives elected for three-year terms from government authorities dealing with nutrition, food safety, health promotion, catering, food industry, trade and agriculture (92).

Malta: Based on the Healthy Lifestyle Promotion and Care of NCDs Act (2016), Malta established an inter-ministerial Advisory Council on Healthy Lifestyles in August 2016 to advise the Minister of Health on any matter related to healthy lifestyles. In particular, the Advisory Council advises on a life course approach to physical activity and nutrition, and on policies, action plans and regulations intended to reduce the occurrence of NCDs. The prime minister appoints the chair and the secretary of the Advisory Council, while the ministers of education, health, finance, social policy, sports, local government, and home affairs appoint one member each (92).

Australia: There are several forums and committees for the purpose of strengthening food regulation with representation from New Zealand and Health Ministers from Australian States and Territories, the Australian Government, as well as other Ministers from related portfolios (e.g. Primary Industries). Where relevant, there is also representation from the Australian Local Government Association.

Ireland: The Department of Health, through ‘A Healthy weight for Ireland’, Obesity Policy and Action Plan 2016 - 2025, will provide leadership, engage and co-ordinate multi-sectorial action and implement best practice in the governance of the OPAP. The department of health and safefood are taking action to establish a multi stakeholder partnership to share knowledge and initiative on healthy weight initiatives (151).</td></tr>
</table>

Evidens for implementering

I Norge finnes det ikke noen egen koordineringsmekanisme på tvers av departementer og myndighetsnivåer når det gjelder forebyggende arbeid for kosthold, overvekt og fedme og ikke-smittsomme sykdommer, utover den koordinerende rollen som Helsedirektoratet har. Samarbeid foregår mer *ad hoc* i forbindelse med utvikling av for eksempel stortingsmeldinger (f.eks. Folkehelsemeldingene), strategier og handlingsplaner.

Handlingsplan for bedre kosthold (99) er undertegnet av åtte departement og oppfølgingen av denne planen fører til jevnlig samarbeid og samhandling mellom departementene. Oppfølgingsansvaret for tiltakene i handlingsplanen er fordelt mellom ulike departementer, men Helsedirektoratet har i oppdrag å koordinere arbeidet med planen, samt følge opp tiltakene innen eget ansvarsområde. De aller fleste tiltakene er under Helse- og omsorgsdepartementets ansvarsområde og gjennomføres av underliggende etater. Det er ett møte i året mellom departementene hvor status og veien videre diskuteres. Departementer og underliggende etater rapporterer årlig i Helsedirektoratets matrise med status på de ulike tiltakene og oppfølgingspunktene. De enkelte departementene har ansvar for oppfølging av tiltak innen eget politikkområde, men flere av tiltakene krever samarbeid og koordinering mellom flere departementer (158). Det påpekes imidlertid i Midtveisevalueringen av handlingsplanen, at departementene ikke har avsatt øremerket tid eller ressurser til å følge opp arbeidet med handlingsplanen, og at inntrykket er at noen av departementene ikke har et særlig stort eierskap til handlingsplanen som sådan, som gjør implementeringen sårbar (158).

Når det gjelder koordineringsmekanismer mot fylkes- og kommunenivå, så samarbeider Helsedirektoratet med mange ulike aktører og også bla. a. mot Fylkesmennene. Fylkesmennene får også oppdragsbrev fra Helsedirektoratet, men de er ikke lenger like spesifikke som tidligere (158). Midtveisevalueringen beskriver at Handlingsplanen ikke ser ut til å bli oppfattet som en handlingsplan for lokale tiltak, at det er mangel på oversikt over hva som gjøres i de ulike kommunene og at det som gjøres ofte er basert på «ildsjeler» (158).

SAMHA 2 – samarbeid mellom myndighetene og matbransjen

**God praksis-beskrivelse**<br>**Det finnes formelle samarbeidsplattformer (med definerte mandater, roller og strukturer) for regelmessig samhandling mellom myndighetene og matbransjen for implementering av mat- og ernæringspolitikk og andre strategier for å bedre kostholdet i befolkningen.**	
**Definisjoner og omfang**	• "Matbransjen" inkluderer matproduksjon, industri, -teknologi, -prosessering, markedsføring, distribusjon, handel og servering, etc.<br>• Inkluderer etablerte grupper, forum eller komiteer som har vært aktive de siste 12 månedene med det formål å dele informasjon, samarbeide, eller få råd om sunn matpolitikk.<br>• Inkluderer plattformer for å støtte, administrere eller overvåke løfter, forpliktelser eller avtaler fra den private sektoren.<br>• Inkluderer plattformer for åpen konsultasjon/høringer.<br>• Inkluderer plattformer der regjeringen kan bistå med ressurser eller ekspertstøtte til matvarebransjen for å implementere politikk.<br>---------------------------<br>• Omfatter ikke felles partnerskap på prosjekter eller medfinansieringsordninger.<br>• Omfatter ikke initiativ som er dekket av HANDEL 3 og 4.<br>• Omfatter ikke plattformer der matbransjen bidrar til **utvikling** av politikk.
**Internasjonale eksempler på god praksis (referansemål)**	**UK:** The UK 'Responsibility Deal' was a UK government initiative to bring together food companies and NGOs to take steps (through voluntary pledges) to address NCDs. It was chaired by the Secretary of State for Health and included senior representatives from the business community (as well as NGOs, public health organisations and local government). A number of other subgroups were responsible for driving specific programs relevant to the commercial food sector (220).<br><br>**Norway:** The letter of intent (Memorandum of Understanding, MoU) for facilitating a healthier diet in the population is a signed agreement between the Norwegian health authorities and food industry (food and trade organizations, food and beverage manufacturers, food retailers and food service industry) in a *Partnership for a healthier diet.* The MoU was signed in 2016 and lasts until 2021. The agreement contains specific quantitative goals related to reducing the intake of salt, added sugar and saturated fat, and increasing the intake of fruits and berries, vegetables, whole grain foods, fish and seafood in the population. The Partnership is organized in a Coordination group with representatives from the main partners including the health authorities. The Coordination group reports to the Minister's food industry group (lead by the Minister for the Elderly and Public Health) that ensures dialogue and political focus on the areas of action. The Coordination group is assisted by a Secretariat organized by the Directorate of Health. A Reference group of scientists within nutrition, food technology, consumer behaviour, psychology and marketing provide expert advice to the coordination group (221).

Evidens for implementering

Intensjonsavtalen for et sunnere kosthold er en signert avtale mellom helsemyndighetene og matbransjen (næringsorganisasjoner, mat- og drikkeprodusenter, dagligvarehandelen og serveringsbransjen) (20). Avtalen ble signert i 2016 og løper til og med 2021. Som beskrevet tidligere har avtalen kvantitative mål for inntak av næringsstoffer og matvaregrupper i befolkningen. Avtalen er organisert som en to-partsavtale (uten representasjon av sivilsamfunn) etter følgende modell:

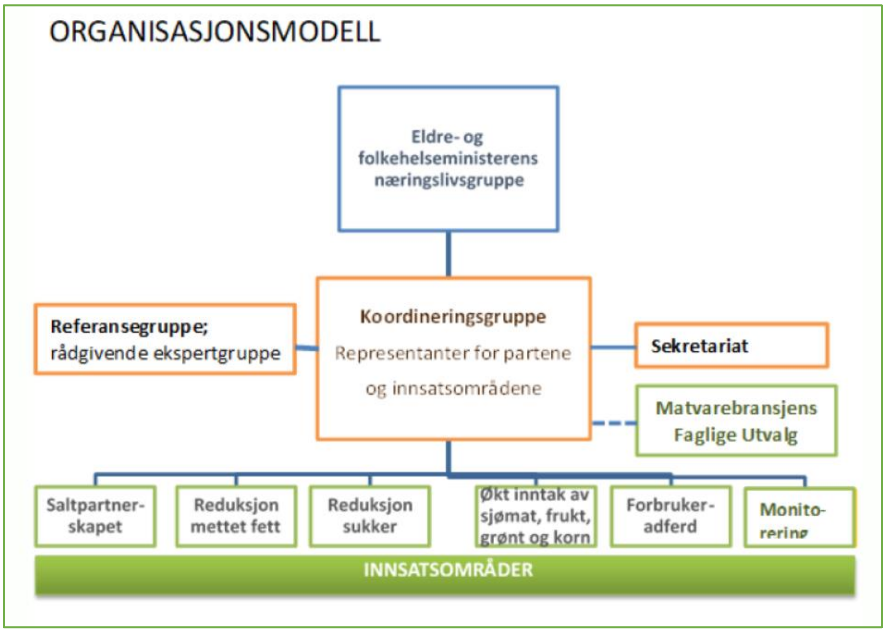

Figur 6. intensjonsavtalens organisasjonsmodell. Hentet fra https://www.helsedirektoratet.no/tema/kosthold-og-ernaering/matbransje-serveringsmarked-og-arbeidsliv/intensjonsavtalen-for-et-sunnere-kosthold

- *Eldre- og folkehelseministerens næringslivsgruppe* er en møtearena mellom statsråden og ledere i matvarebransjen som sikrer dialog og politisk fokus. Gruppen møtes to ganger i året.
- En *koordineringsgruppe* bestående av representanter fra partene (matbransjen og helsemyndighetene) har det overordnede ansvaret for å koordinere arbeidet med intensjonsavtalen. Gruppen møtes fire ganger i året.
- Det er etablert en *referansegruppe* bestående av forskere innen ernæring, matteknologi, forbrukeratferd og markedsføring som kan gi ekspertråd til koordineringsgruppen og innsatsområdene. Referansegruppen brukes kun ved behov.
- Et *sekretariat* ledes av Helsedirektoratet bistår koordineringsgruppen, referansegruppen og partene med gjennomføringen av arbeidet.

SAMHA 3 – samarbeid mellom myndighetene og sivilsamfunn

God praksis-beskrivelse	
**Det finnes formelle samarbeidsplattformer for regelmessig samhandling mellom myndighetene og sivilsamfunn/frivillige organisasjoner (med definerte mandater, roller og strukturer) for utvikling, implementering og evaluering av mat- og ernæringspolitikk og andre strategier for å bedre ernæring i befolkningen.**	
**Definisjoner og omfang**	• "Sivilsamfunn/frivillige organisasjoner" omfatter samfunnsgrupper og forbrukerrepresentanter/organisasjoner, frivillige organisasjoner, akademia, fagforeninger etc.<br>• Inkluderer etablerte grupper, fora eller komiteer som har vært aktive i løpet av de siste 12 månedene med det formål å dele informasjon, samarbeide og søke råd.<br>• Inkluderer plattformer for konsultasjon om foreslåtte planer, policies eller høringer.<br>---------------------------<br>• Omfatter ikke retningslinjer eller prosedyrer som styrer konsultasjoner ved utviklingen av mat- og ernæringspolitikk (se STYRING 3).
**Internasjonale eksempler på god praksis (referansemål)**	**Brazil:** The National Council of Food and Nutrition Security (CONSEA) is a formal advisory platform made up of civil society (2/3) and government reps (1/3). It is a participatory instrument for designing, suggesting, implementing and evaluating food and nutritional security policy. Through CONSEA, civil society has been able to influence policy directions more directly. CONSEA supported Congress to pass a bill obliging local governments to buy at least 30% of the food destined for school meals from small-scale farmers (222).

Evidens for implementering

Norske helsemyndigheter har ikke noe formell samarbeidsplattform med frivillige organisasjoner innen mat- og ernæringsområdet. Representanter fra frivillig sektor er med som en del av styringsgruppa til saltpartnerskapet.

Kommentarer

Utvalget bak Midtveisevalueringen av Handlingsplanen foreslår å vurdere å inkludere frivillige aktører i intensjonsavtalen som en mer uavhengig instans som også kan brukes som støttespiller (158).

SAMHA 4 – systembasert tilnærming med lokale organisasjoner

**<u>God praksis-beskrivelse</u>**<br>**Myndighetene arbeider med en systembasert tilnærming med lokale og nasjonale organisasjoner for å forbedre matomgivelser på nasjonalt nivå.**	
**Definisjoner og omfang**	• En «systembasert tilnærming» kan inkludere politikk innenfor andre helseområder.<br>• Kan inkludere en tilnærming som tar utgangspunkt i sosiale helsedeterminanter.<br>• Kan bringe flere avdelinger eller departementer sammen for å arbeide opp mot helse.<br>• Inkluderer flere forvaltnings/myndighetsnivåer.<br><br>Målet til en systembasert tilnærming er ifølge INFORMAS å:<br>▪ Bidra til en dedikert og kompetent arbeidsstyrke på nasjonalt og/eller lokalt nivå som kan engasjere, stimulere og påvirke ulike nivåer av systemet for å bekjempe overvekt og NCDs.<br>▪ Bygge nettverk med forebyggende aktører på tvers av systemet og på tvers av sektorer for å styrke helseutfall på mange områder.<br>▪ Fange opp og formidle kunnskap og data om fremgang, utvikling, resultat og effektivitet og etterspørre nye former for forskning, politikk og samarbeid.<br>▪ Tildele ressurser basert på den beste investeringen for å oppnå endring basert på behov i befolkningen, for å bidra til langsiktig endring ved å sette lokale myndigheter i stand til å ta lederskap og utvikle tiltak for folkehelse.<br>▪ Bygge lederskap for vedvarende forebygging på tvers av hele systemet, for å føre til effektiv og langvarig endring.
**Internasjonale eksempler på god praksis (referansemål)**	**New Zealand:** Healthy Families NZ is a large-scale initiative that brings community leadership together in a united effort for better health. It aims to improve people’s health where they live, learn, work and play, in order to prevent chronic disease. Led by the Ministry of Health, the initiative will focus on ten locations in New Zealand in the first instance. It has the potential to impact the lives of over a million New Zealanders. The Government has allocated $40 million over four years to support Healthy Families NZ (223).<br><br>**Australia:** Healthy together Victoria in Australia focuses on addressing the underlying causes of poor health in children's settings, workplaces and communities by encouraging healthy eating and physical activity and reducing smoking and harmful alcohol use. Healthy Together Victoria incorporates policies and strategies to support good health across Victoria, as well as locally-led Healthy Together Communities. The initiative was originally jointly funded by the State Government of Victoria and the Australian Government through the National Partnership Agreement on Preventive Health (224). It is unclear at this stage whether funding for Healthy Together Victoria will continue or not.

Evidens for implementering

I Norge finnes det ingen systembasert tilnærming hvor myndighetene samarbeider med lokale og nasjonale organisasjoner for å forbedre matomgivelser på nasjonalt nivå. Kommunene skal, i henhold til folkehelseloven og lov om kommunale helse- og omsorgstjenester, fastsette overordnede mål og strategier for folkehelsearbeidet med utgangspunkt i identifiserte folkehelseutfordringer (110, 225). Helsedirektoratet legger til rette for slikt arbeid gjennom retningslinjer og veivisere for lokalt folkehelsearbeid inkludert innen ernæring (132) og kursing f.eks. av kursholdere for «Bra mat»-kurs . Dette er imidlertid ikke innenfor et system eller nettverk.

OMRÅDE 12: "HELSE I ALT VI GJØR"

Det er etablert prosesser for å sikre politisk samsvar og samordning, og at effekt på folkehelse er et uttalt vurderingskriterium ved utvikling av politikk.

Det er to indikatorer for dette området:

- HIAV 1 – vurdering av helsekonsekvenser i matpolitikk
- HIAV 2 – vurdering av helsekonsekvenser i annen politikk

HIAV 1 – vurdering av helsekonsekvenser i matpolitikk

**<u>God praksis-beskrivelse</u>**<br>**Det er etablert prosesser for å sikre at utvikling av all politikk som er relatert til mat og ernæring tar hensyn til og prioriterer ernæring og helse i befolkningen samt reduksjon av sosiale ulikheter, og at det tas hensyn til sårbare grupper.**	
**Definisjoner og omfang**	• Inkluderer retningslinjer, prosedyrer, verktøy og ressurser som bidrar til å ta hensyn til konsekvenser på ernæring og helse, sosial ulikhet samt effekter i sårbare populasjoner før, under og etter implementering av mat- og ernæringspolitikk.<br>• Inkluderer etablering av tverrdepartementale styrings- og samordningsstrukturer når man utvikler mat- og ernæringsrelatert politikk.
**Internasjonale eksempler på god praksis (referansemål)**	**Slovenia:** A Health Impact Assessment was undertaken in Slovenia to assess the health effects of agricultural policy at national level, encompassing policy analysis, rapid appraisal workshops with stakeholders from a range of backgrounds, review of research evidence relevant to the agricultural policy, analysis of Slovenian data for key health-related indicators, a report on the findings to a key cross-government group and evaluation (226).<br><br>**Ireland:** Step 9 of the ' A Healthy Weight for Ireland':, Obesity Policy and Action Plan 2016-2025 aims to allocate resources according to need, in particular to those population groups most in need of support in the prevention and management of obesity, with particular emphasis on families and children during the first 1,000 days of life. The priority actions to commence in first year were to assess the needs of vulnerable groups as the basis of allocation of resources for preventative and treatment services for children and adults. The Healthy Ireland fund was established in 2017 with an allocation of €5 million and with additional allocations of €5 million in 2018 and 2019. The first round of the Fund was distributed through Local Community Development Committees, Children and Young Person's Services Committees and statutory organisations (151).

Evidens for implementering

"Helse i alt vi gjør" er ett av fem grunnleggende prinsipper som folkehelseloven bygger på (110).

Statlige myndigheter skal i sin virksomhet vurdere konsekvenser for befolkningens helse der det er relevant. Fylkesmennene og Helsedirektoratet har delegert sektoransvar for fortolkning og oppfølging av folkehelseloven. Direktoratet skal videre bidra til å iverksette nasjonal politikk på folkehelseområdet, og være pådriver for kunnskapsbasert folkehelsearbeid lokalt, regionalt og nasjonalt.

Folkehelseinstituttet og Helsedirektoratet arbeider med å utvikle verktøy og metoder for implementering av folkehelseloven.

Helsekonsekvensutredning (HKU) er et verktøy som kan synliggjøre hvordan beslutninger og tiltak i ulike sektorer kan påvirke befolkningens helse. Helsedirektoratet har utarbeidet informasjonsmateriale til folkehelsearbeid i kommunene som beskriver når og hvordan man kan gjøre en HKU. Direktoratet skriver om sektoren **landbruk, fiskeri og mat** at «matsikkerhet og mattrygghet styrkes ved å ta helsehensyn i matproduksjon, markedshensyn og distribusjon, gjennom å fremme forbrukertillit og ved å sørge for bærekraftig fiskeri- og landbrukspraksis. Sunn mat er avgjørende for folks helse». Det gis også eksempler på spørsmål som kan stilles i en HKU, for eksempel:

- *Påvirkes forutsetningene for å drive med fiskeri eller landbruk?*
- *Påvirkes tilgjengeligheten til sunn eller usunn mat?*
- *Påvirkes matproduksjon eller matsikkerhet?*
- *Påvirkes forutsetningene for gode matvaner (inkl amming)?*

Kommentarer

Det er ikke funnet evidens rundt implementering av verktøyene og rådene som er beskrevet over.

HIAV 2 – vurdering av helsekonsekvenser i annen politikk

**<u>God praksis-beskrivelse</u>**<br>**Det er etablert prosesser (for eksempel helsekonsekvensutredninger) for å vurdere påvirkning på helse i utviklingen av annen politikk som ikke er direkte relatert til mat og ernæring.**	
**Definisjoner og omfang**	• Omfatter en overordnet HIAV-strategi eller -plan med klare tiltak utenfor helsesektoren på regjeringsnivå.<br>• Omfatter retningslinjer, prosedyrer, verktøy og ressurser som bidrar til å ta hensyn til helsekonsekvenser før, under og etter implementering av politikk som ikke dreier seg om mat eller ernæring.<br>• Omfatter etablering av tverrdepartementale eller tverrsektorielle styringsstrukturer for å implementere HIAV.<br>• Omfatter kapasitetsbygging innen folkehelse for departementer utenfor helse, for eksempel landbruk, utdanning og handel.<br>• Omfatter rapporteringskrav for helseutfall for andre departementer enn HOD.
**Internasjonale eksempler på god praksis (referansemål)**	**South Australia:** Established in 2007, the implementation of Health in All Policies (HiAP) in South Australia has been supported by a high-level mandate from central government, an overarching framework which is supportive of a diverse program of work, a commitment to work collaboratively and in partnership across agencies, and a strong evaluation process. The government has established a dedicated HiAP team within South Australia Health to build workforce capacity and support Health Lens Analysis projects. Since 2007, the South Australian HiAP approach has evolved to remain relevant in a changing context. However, the purpose and core principles of the approach remain unchanged. There have been five phases to the work of HiAP in South Australia between 2007 and 2016: 1) Prove concept and practice emerges (2007-2008), 2) Establish and apply methodology (2008-2009), 3) Consolidate and grow (2009-2013), 4) Adapt and review (2014) and 5) Strengthen and systematize (2015-2016) (224).

Evidens for implementering

"Helse i alt vi gjør" er ett av fem grunnleggende prinsipper som folkehelseloven bygger på (227). Loven setter krav til politisk forankring av folkehelsearbeidet og for en langsiktig, systematisk innsats.

Ifølge loven skal statlige myndigheter i sin virksomhet vurdere konsekvenser for befolkningens helse der det er relevant. Fylkesmennene og Helsedirektoratet skal videre bidra til å iverksette nasjonal politikk på folkehelseområdet, og være pådrivere for kunnskapsbasert folkehelsearbeid lokalt, regionalt og nasjonalt. Folkehelseinstituttet og Helsedirektoratet arbeider med å utvikle verktøy og metoder for implementering av folkehelseloven (110).

Kommentarer

Det er ikke funnet evidens rundt implementering av verktøyene og rådene som er beskrevet over.

Litteraturliste

1. Swinburn B, Sacks G, Vandevijvere S, Kumanyika S, Lobstein T, Neal B, et al. INFORMAS (International Network for Food and Obesity/non-communicable diseases Research, Monitoring and Action Support): overview and key principles. Obesity reviews. 2013;14 Suppl 1:1.
2. Folkehelseinstituttet. Overvekt og fedme i Noreg 2017 [Available from: https://www.fhi.no/nettpub/hin/levevaner/overvekt-og-fedme/.
3. Øverland S, Knudsen A, Vollset S, Kinge J, Skirbekk V, Tollånes M. Sykdomsbyrde i Norge 2016. Resultater fra Global Burden of Diseases, Injuries, and Risk Factors Study 2016 (GBD 2016). Rapport 2018. Bergen/Oslo: Folkehelseinstituttet; 2018.
4. Helse- og omsorgsdepartementet. Folkehelsemeldinga. Gode liv i eit trygt samfunn. 2018.
5. Folkehelseinstituttet. Kosthaldet i Noreg 2018 [Available from: https://www.fhi.no/nettpub/hin/levevaner/kosthald/.
6. WHO. Global action plan for the prevention and control of noncommunicable diseases 2013-2020. Geneva: World Health Organization; 2013.
7. WHO. The updated Appendix 3 of the WHO Global NCD Action Plan 2013-2020: 'Best buys' and other recommended interventions. Geneva World Health Organization 2017.
8. Swinburn B, Vandevijvere S, Kraak V, Sacks G, Snowdon W, Hawkes C, et al. Monitoring and benchmarking government policies and actions to improve the healthiness of food environments: a proposed Government Healthy Food Environment Policy Index. Obesity reviews : an official journal of the International Association for the Study of Obesity. 2013;14 Suppl 1:24-37.
9. Vandevijvere S, Swinburn B. INFORMAS Public sector module. Healthy Food Environment Policy Index (Food-EPI) 2017 [Available from: https://figshare.com/articles/INFORMAS_Protocol_Public_Sector_Module_-_Healthy_Food_Environment_Policy_Index_Food-EPI_/5673439.
10. World Cancer Research Fund International. NOURISHING database 2019 [Available from: https://www.wcrf.org/int/policy/nourishing-database.
11. Public Health England. Sugar reduction: From evidence to action. Public Health England: London. 2015 [Available from: https://www.gov.uk/government/publications/sugar-reduction-from-evidence-into-action.
12. Public Health England. Sugar reduction: report on first year progress 2018 [Available from: https://www.gov.uk/government/publications/sugar-reduction-report-on-first-year-progress.
13. Hofman KJ, Tollman SM. Population health in South Africa: a view from the salt mines. The Lancet Global Health. 2013;1(2):e66-e7.
14. NOURISHING Framework - Improve nutritional quality of the whole food supply [Internet]. 2016. Available from: https://www.wcrf.org/int/policy/nourishing-database.
15. Peters S, Dunford E, Ware L, Harris T, Walker A, Wicks M, et al. The sodium content of processed foods in South Africa during the introduction of mandatory sodium limits. Nutrients. 2017;9(4).
16. National Agreement to improve Product Composition. Available from: http://www.akkoordverbeteringproductsamenstelling.nl/dsresource?type=pdf&disposition=inline&objectid=rivmp:303682&versionid=&subobjectname=. 2017.
17. Mattilsynet. Regelverksprosess. Forskrift om begrensning i innhold av transfettsyrer i næringsmidler 2019 [Available from:

https://www.mattilsynet.no/mat_og_vann/merking_av_mat/generelle_krav_til_merking_av_mat/forskrift_om_begrensning_i_innhold_av_transfettsyrer_i_naeringsmidler.36415.

18. Forskrift om transfettsyrer i næringsmidler. Forskrift om transfettsyrer i næringsmidler. LOV-2003-12-19-124-§9, FOR-2003-12-19-1790 2014 [Available from: https://lovdata.no/dokument/SF/forskrift/2014-01-16-34.

19. Matloven. Lov om matproduksjon og mattrygghet mv. LOV-2003-12-19-124 2003 [Available from: https://lovdata.no/dokument/NL/lov/2003-12-19-124?q=matloven.

20. Helsedirektoratet. Intensjonsavtalen for et sunnere kosthold 2019 [Available from: https://www.helsedirektoratet.no/tema/kosthold-og-ernaering/matbransje-serveringsmarked-og-arbeidsliv/intensjonsavtalen-for-et-sunnere-kosthold.

21. Helsedirektoratet. Salt og saltpartnerskapet 2019 [Available from: https://www.helsedirektoratet.no/tema/kosthold-og-ernaering/matbransje-serveringsmarked-og-arbeidsliv/salt-og-saltpartnerskapet#veiledende-saltm%C3%A5l-i-partnerskapet.

22. Helsedirektoratet. Saltpartnerskapet 2015–2018 – Fremdrift og måloppnåelse 2019 [Available from: https://www.helsedirektoratet.no/rapporter/saltpartnerskapet-2015-2018-fremdrift-og-maloppnaelse.

23. Helsedirektoratet. Utviklingen i norsk kosthold 2019. IS-2866. Oslo: Helsedirektoratet 2019.

24. FAFO. Intensjonsavtalen om sunnere kosthold 2019 [Available from: https://www.fafo.no/index.php/prosjekter/aktive-prosjekter/item/intensjonsavtalen-om-sunnere-kosthold?category_id=170.

25. The Chip Group. The Chip Group: Helping you make better chips. Available from: http://blog.thechipgroup.co.nz/. 2016.

26. NYC Health Department. National Salt and Sugar Reduction Initiative (NSSRI) 2019 [Available from: https://www1.nyc.gov/site/doh/health/health-topics/national-salt-sugar-reduction-initiative.page.

27. Department of Health. National salth reduction initiative corporate achievements 2009-2014 2014 [Available from: https://www1.nyc.gov/assets/doh/downloads/pdf/cardio/nsri-corporate-commitments.pdf.

28. Internkontrollforskriften for næringsmidler. Forskrift om internkontroll for å oppfylle næringsmiddellovgivningen. FOR-1994-12-15-1187 1994 [Available from: https://lovdata.no/dokument/SF/forskrift/1994-12-15-1187#KAPITTEL_2.

29. NOURISHING Framework - Nutrition label standards and regulations on the use of claims and implied claims on food [Internet]. 2016. Available from: https://www.wcrf.org/int/policy/nourishing-database.

30. Regulation (EU) No 1169/2011. EU Regulation 1169/2011 on the Provision of Food Information to Consumers 2011. Available from: https://eur-lex.europa.eu/legal-content/EN/ALL/?uri=CELEX%3A32011R1169.

31. Matinformasjonsforskriften. Forskrift om matinformasjon til forbrukerne. FOR-2014-11-28-1497 2014 [Available from: https://lovdata.no/dokument/SF/forskrift/2014-11-28-1497?q=Matinformasjonsforskriften.

32. Mattilsynet. Nye krav til næringsdeklarasjonen 2016 [Available from: https://www.mattilsynet.no/mat_og_vann/merking_av_mat/generelle_krav_til_merking_av_mat/nye_krav_til_naeringsdeklarasjonen.20419.

33. Mattilsynet. Generelle krav til merking av mat 2020 [Available from: https://www.mattilsynet.no/mat_og_vann/merking_av_mat/generelle_krav_til_merking_av_mat/.

34. Forskrift om ernærings- og helsepåstander. Forskrift om ernærings- og helsepåstander om næringsmidler. FOR-2010-02-17-187 2010 [Available from: https://lovdata.no/dokument/SF/forskrift/2010-02-17-187?q=Forskrift%20om%20ern%C3%A6rings-%20og%20helsep%C3%A5stander.

35. Mattilsynet. Oversikt over de tillatte ernæringspåstandene og de godkjente eller avslåtte helsepåstandene 2019 [Available from: https://www.mattilsynet.no/mat_og_vann/merking_av_mat/ernarings_og_helsepastander/oversikt_over_de_tillatte_ernaeringspaastandene_og_de_godkjente_eller_avslaatte_helsepaastandene.30508#factStaticShortcuts.

36. Mattilsynet. Veiledning om bruk av ernæringspåstander og helsepåstander 2018. Available from: https://www.mattilsynet.no/om_mattilsynet/gjeldende_regelverk/veiledere/veiledning_om_bruk_av_ernaeringspaastander_og_helsepaastander.30798/binary/Veiledning%20om%20bruk%20av%20ern%C3%A6ringsp%C3%A5stander%20og%20helsep%C3%A5stander.

37. Mattilsynet. Mange feilaktige ernærings- og helsepåstander i dagligvarer 2015 [Available from: https://www.mattilsynet.no/mat_og_vann/merking_av_mat/ernarings_og_helsepastander/mange_feilaktige_ernaerings_og_helsepaastander_i_dagligvarer.19133.

38. Department of Health. Guide to creating a front of pack (FoP) nutrition label for pre-packed products sold through retail outlets 2016 [Available from: https://assets.publishing.service.gov.uk/government/uploads/system/uploads/attachment_data/file/566251/FoP_Nutrition_labelling_UK_guidance.pdf.

39. Commonwealth of Australia. Health Star Rating System. Available from: http://healthstarrating.gov.au/internet/healthstarrating/publishing.nsf/content/home. 2016.

40. Colruyt Group. What is the Nutri-Score? n.d. [Available from: https://nutriscore.colruytgroup.com/colruytgroup/en/about-nutri-score/.

41. Chantal J, Hercberg S, World Health Organization. Regional Office for Europe. Development of a new front-of-pack nutrition label in France: the five-colour Nutri-Score. Public health panorama. 2017;03(04):712-25.

42. Mattilsynet. Veileder til nøkkelhullforskriften 2015 2015. Available from: https://www.mattilsynet.no/om_mattilsynet/gjeldende_regelverk/veiledere/veileder_til_nokkelhullsforskriften.18846/binary/Veileder%20til%20N%C3%B8kkelhullsforskriften.

43. Forskrift om frivillig merking med Nøkkelhullet. In: Helse- og omsorgsdepartementet, editor. 2015.

44. Helsedirektoratet. 10 år med nøkkelhull-mat 2019 [Available from: https://www.matportalen.no/merking/tema/nokkelhullet/10_aar_med_nokkelhull-mat.

45. Baker- og konditorbransjens Landsforening. Brødskala´n – spis litt grovere. n.d. [Available from: http://www.bakerkonditor.no/bklf/brodskalan-spis-litt-grovere/.

46. Mattilsynet. Merkesjekken 2018 - Mattilsynet ser på brød og brødvarer 2019 [Available from: https://www.mattilsynet.no/mat_og_vann/merking_av_mat/generelle_krav_til_merking_av_mat/merkesjekken_2018__mattilsynet_ser_paa_brod_og_brodvarer.33530.

47. Nutrition Resource Centre. Ontario's Healthy Menu Choices Act In Effect 2017 [Available from: https://opha.on.ca/Nutrition-Resource-Centre/News/NRC-News/2017/Ontario%E2%80%99s-Healthy-Menu-Choices-Act-In-Effect.aspx.

48. Saudi Food & Drug Authority. SFDA launches Healthy Food Regulation Strategy 2018 [Available from: http://sfda.gov.sa/en/food/news/Pages/f11-9-2018a1.aspx.
49. Helse- og omsorgsdepartementet. Høring - revidert forslag til forskrift om markedsføring rettet mot barn og unge av usunn mat og drikke 2013 [Available from: https://www.regjeringen.no/no/dokumenter/horing---revidert-forslag-til-forskrift-/id727092/?expand=horingsnotater.
50. Matbransjens faglige utvalg. MFUs oppgaver n.d. [Available from: http://www.mfu.as/.
51. Matbransjens faglige utvalg. Retningslinjer for markedsføring av mat og drikke rettet mot barn 2019 [Available from: http://www.mfu.as/85197-Retningslinjer-for-markedsforing-oppdatert-1-10-2019.
52. Matbransjens faglige utvalg. Veiledning til retningslinjer oppdatert 1/9-2016 2016 [Available from: Veiledning til retningslinjer oppdatert 1/9-2016.
53. Matbransjens faglige utvalg. MFU veiledning til bruk av sosiale medier n.d. [Available from: http://www.mfu.as/60775-MFU-veiledning-til-bruk-av-sosiale-medier.
54. Matbransjens faglige utvalg. Produktlisten n.d. [Available from: http://www.mfu.as/36577-Produktlisten.
55. NOURISHING Framework - Restrict food advertising and other forms of commercial promotion. [Internet]. 2016. Available from: https://www.wcrf.org/int/policy/nourishing-database.
56. Matbransjens faglige utvalg. Code for marketing of food and drink aimed at children updated 1/9-2016 2016 [Available from: http://www.mfu.as/44552-Code-for-marketing-of-food-and-drink-aimed-at-children-updated-1-9-2016.
57. Broadcasting Authority of Ireland. Broadcasting Authority of Ireland Children's Commercial Communications Code. Guidance Notes & Direction in respect of product placement and commercial communications for food (including HFSS food). 2013.
58. USDA Foreign Agricultural Service. Update on Chilean Advertising of Packaged Foods and Beverages 2018 [Available from: https://apps.fas.usda.gov/newgainapi/api/report/downloadreportbyfilename?filename=Update%20on%20Chilean%20Advertising%20of%20Packaged%20Foods%20and%20Beverages%20_Santiago_Chile_2-14-2018.pdf.
59. Correa T, Fierro C, Reyes M, Dillman Carpentier FR, Taillie LS, Corvalan C. Responses to the Chilean law of food labeling and advertising: exploring knowledge, perceptions and behaviors of mothers of young children. International Journal of Behavioral Nutrition and Physical Activity. 2019;16.
60. Kringkastingsloven. Lov om kringkasting og audiovisuelle bestillingstjenester. LOV-1992-12-04-127 1993 [Available from: https://lovdata.no/dokument/NL/lov/1992-12-04-127?q=kringkastingsloven.
61. Forskrift om kringkasting. Forskrift om kringkasting og audiovisuelle bestillingstjenester. FOR-1997-02-28-153 1997 [Available from: https://lovdata.no/dokument/SF/forskrift/1997-02-28-153?q=Forskrift%20om%20kringkasting.
62. Forbrukertilsynet. Forbrukertilsynets veiledning om handelspraksis overfor barn og unge 2018 [Available from: https://www.forbrukertilsynet.no/lov-og-rett/veiledninger-og-retningslinjer/forbrukertilsynets-veiledning-om-handelspraksis-overfor-barn-og-unge#chapter-8-4.
63. Kent MP, Dubois L, Wanless AJIJoPO. Food marketing on children's television in two different policy environments. 2011;6(sup3):e433-41.

64. Advertising Standards Authority. Food: HFSS Media Placement 2018 [Available from: https://www.asa.org.uk/advice-online/food-hfss-media-placement.html.

65. Markedsføringsloven. Lov om kontroll med markedsføring og avtalevilkår mv. LOV-2009-01-09-2 2009 [Available from: https://lovdata.no/dokument/NL/lov/2009-01-09-2?q=Markedsf%C3%B8ringsloven.

66. Forbrukertilsynet. Forbrukertilsynets veileder om merking av reklame i sosiale medier 2018 [Available from: https://www.forbrukertilsynet.no/content/2017/12/Forbrukertilsynets-veileder-om-merking-av-reklame-i-sosiale-medier.pdf.

67. Corvalán C, Reyes M, Garmendia ML, Uauy R. Structural responses to the obesity and non-communicable diseases epidemic: Update on the Chilean law of food labelling and advertising. Obesity Reviews. 2019;20(3):367-74.

68. Greater London Authority. TfL junk food ads ban will tackle child obesity 2019 [Available from: https://www.london.gov.uk/what-we-do/business-and-economy/food/tfl-junk-food-ads-ban-will-tackle-child-obesity.

69. The Organisation for Economic Co-operation and Development. OECD Reviews of Public Health: Chile: A Healthier Tomorrow 2019 [Available from: http://www.oecd.org/publications/oecd-reviews-of-public-health-chile-9789264309593-en.htm.

70. Forbrukertilsynet. Reklame i skolen 2019 [Available from: https://www.forbrukertilsynet.no/reklame-i-skolen.

71. Opplæringslova. Lov om grunnskolen og den vidaregåande opplæringa (opplæringslova). LOV-1998-07-17-61 1998 [Available from: https://lovdata.no/dokument/NL/lov/1998-07-17-61?q=Oppl%C3%A6ringsloven.

72. Friskolelova. Lov om frittståande skolar (friskolelova). LOV-2003-07-04-84 2003 [Available from: https://lovdata.no/dokument/NL/lov/2003-07-04-84?q=friskolelova.

73. Helsedirektoratet. Nasjonal faglig retningslinje. Mat og måltider i skolen. 2015 [Available from: https://www.helsedirektoratet.no/retningslinjer/mat-og-maltider-i-skolen.

74. Hunter College NYC Food Policy Center. Chile Banishes Cartoon Mascots from Supermarket Shelves 2018 [Available from: https://www.nycfoodpolicy.org/chile-banishes-cartoon-mascots-supermarket-shelves/.

75. WHO. Set of recommendations on the marketing of foods and non-alcoholic beverages to children.: World Health Organization; 2010. Available from: http://apps.who.int/iris/bitstream/10665/44416/1/9789241500210_eng.pdf.

76. WHO Regional Office for Europe. Evaluating implementation of the WHO Set of Recommendations on the marketing of foods and non-alcoholic beverages to children 2018. Available from: http://www.euro.who.int/__data/assets/pdf_file/0003/384015/food-marketing-kids-eng.pdf.

77. Veerman JL, Cobiac LJ. Removing the GST exemption for fresh fruits and vegetables could cost lives. . Med J Aust. 2013;199:p. 534-5.

78. NOURISHING Framework - Use economic tools to address food affordability and purchase incentives [Internet]. 2016. Available from: https://www.wcrf.org/int/policy/nourishing-database.

79. Merverdiavgiftsloven. Lov om merverdiavgift (merverdiavgiftsloven). LOV-2009-06-19-58 2009 [Available from: https://lovdata.no/dokument/NL/lov/2009-06-19-58?q=merverdiavgiftsloven.

80. Helsedirektoratet. Ti tiltak for å redusere sykdomsbyrden og bedre folkehelsen. IS-2810. Oslo: Helsedirektoratet; 2018.

81. NOU 2019: 11. Enklere merverdiavgift med én sats. Oslo: Finansdepartementet; 2019.

82. Helsedirektoratet. Høringssvar fra Helsedirektoratet 2019 [Available from: https://www.regjeringen.no/no/dokumenter/horing--nou-2019-11-enklere-merverdiavgift-med-en-sats/id2652783/?uid=52dc5df2-e6a1-4f05-87bd-064486f16751.

83. Rathbone Greenbank Investments. The UK sugar tax: one year on 2019 [Available from: https://www.rathbonegreenbank.com/insight/uk-sugar-tax-one-year.

84. UK Parliament. Financial Statement 2017 [Available from: https://hansard.parliament.uk/Commons/2017-03-08/debates/9128238B-4A1C-4EEA-A6F0-6FEB0B5024D8/FinancialStatement#contribution-7B30FFC0-4B7A-4D63-8075-3C7F83EDC5.

85. Biro A. Did the junk food tax make the Hungarians eat healthier? Food Policy. 54: p. 107-115. 2015.

86. Colchero MA, Popkin BM, Rivera JA, Ng SW. Beverage purchases from stores in Mexico under the excise tax on sugar sweetened beverages: observational study. BMJ. 2016;352.

87. Ng SW, Rivera JA, Popkin BM, Colchero MA. Did high sugar-sweetened beverage purchasers respond differently to the excise tax on sugar-sweetened beverages in Mexico? Public health nutrition. 2019;22(4):750-6.

88. NOU 2019: 8. Særavgiftene på sjokolade- og sukkervarer og alkoholfrie drikkevarer. Oslo: Finansdepartementet; 2019.

89. Nærings- og fiskeridepartementet. Prop. 1 S (2018–2019) FOR BUDSJETTÅRET 2019 Utgiftskapitler: 900–950, 2421, 2426, 2429, 2460, 2540 Inntektskapitler: 3900–3961, 5325, 5326, 5329, 5460, 5574, 5612, 5613, 5625, 5629, 5656 2018 [Available from: https://www.regjeringen.no/no/dokumenter/prop.-1-s-20182019/id2613238/.

90. Finansdepartementet. Høring - NOU 2019: 8 Særavgiftene på sjokolade- og sukkervarer og alkoholfrie drikkevarer 2019 [Available from: https://www.regjeringen.no/no/dokumenter/horing---nou-2019-8-saravgiftene-pa-sjokolade--og-sukkervarer-og-alkoholfrie-drikkevarer/id2642058/?expand=horingsinstanser.

91. Finansdepartementet. Statsbudsjettet fra A til Å. Sukkeravgift 2020 [Available from: https://www.statsbudsjettet.no/Statsbudsjettet-2020/Statsbudsjettet-fra-A-til-A/Sukkeravgift/.

92. NOURISHING Framework - Harness supply chain & actions across sectors to ensure coherence with health [Internet]. 2016. Available from: https://www.wcrf.org/int/policy/nourishing-database.

93. Forskrift om tilskudd til frukt i skolen. Forskrift om tilskudd til prisnedskrivning av frukt og grønnsaker i grunnskolen. FOR-2004-04-01-623 2004 [Available from: https://lovdata.no/dokument/SF/forskrift/2004-04-01-623?q=Forskrift%20om%20tilskudd%20til%20prisnedskrivning.

94. Helsedirektoratet, opplysningskontoret for frukt og grønt. Hva er Skolefrukt? n.d. [Available from: https://skolefrukt.no/hva-er-skolefrukt/.

95. Statistisk Sentralbyrå. Befolkning 2019 [Available from: https://www.ssb.no/befolkning/statistikker/folkemengde/aar-per-1-januar.

96. Omsetningsloven. Lov til å fremja umsetnaden av jordbruksvaror 1936 [Available from: https://lovdata.no/dokument/NL/lov/1936-07-10-6?q=lov%20til%20%C3%A5%20fremja%20umsetnaden.

97. Omsetningsrådet. Omsetningsrådets årsmelding 2018 2019 [Available from: https://www.landbruksdirektoratet.no/no/styrer-rad-utvalg/omsetningsradet/arsmeldinger.

98. Arbeids- og sosialdepartementet. Rundskriv A-2/18 Statlige veiledende retningslinjer for økonomisk stønad for 2019 2018 [Available from: https://www.regjeringen.no/no/dokumenter/rundskriv-a-218-statlige-veiledende-retningslinjer-for-okonomisk-stonad-for-2019/id2622819/.

99. Departementene. Nasjonal handlingsplan for bedre kosthold (2017-2021). Sunt kosthold, måltidsglede og god helse for alle! Oslo: Helse- og omsorgsdepartementet; 2017.

100. Healthy Ireland. Nutrition Standards for School Meals 2017 [Available from: https://www.gov.ie/en/publication/484f17-nutrition-standards-for-school-meals/.

101. Fundo Nacional de Desinvolvimento da Educacao. Sobre o PNAE. Available from: http://www.fnde.gov.br/programas/alimentacao-escolar. 2016.

102. Massri C, Sutherland S, Källestål C, Peña S. Impact of the Food-Labeling and Advertising Law Banning Competitive Food and Beverages in Chilean Public Schools, 2014–2016. American journal of public health. 2019;109(9).

103. National Nutrition Council Finland. Eating and learning together - recommendations for school meals 2017 [Available from: https://www.julkari.fi/handle/10024/134867.

104. National Nutrition Council Finland. Health and joy from food - meal recommendations for early childhood education and care 2018 [Available from: https://www.julkari.fi/handle/10024/135969.

105. British Nutrition Foundation. Food provision 2015 [Available from: https://www.nutrition.org.uk/foodinschools/foodprovision/food-provision.html?__cf_chl_jschl_tk__=82c0368621883eda3e04007656bc6f936d3199d4-1578477749-0-AQT4_qf2dt6KtAUVK4JDw7173sIPtemonSECefe1dwQtrYHT9jVCPJA8WW111pa6VG0mLxi1dTrd9w6vOBIBseRdKnEc-49xiPw6gVTyGhnRnLQ53k_Fl0CyvaOQNwzCOUNktrFKrVZPQUbBBiQY8QWSYPGML_OmV-OmqIYVLo5vvuS9kZG0hie3G9WP9XAs32OYmf7ZnfeGABUBGOvyczxBuvzhh3fDrZ1YerKLBkRkCOW8dNJYFtEGyKzcBObivDA0lNXsMKycUOTOVAW9y3G_9I8wKNs7-wMG1KYXIRCZSkg3mjQXLGw36wO2QmYqKqqFroeG312y33QBAELKhzGQS7mzfbzlZhxa_hS7QSDhvwaLL7ZNWW5jeaMZmo9Ucw.

106. Ministry of Education NFfED. Resolution No 26 of 17 June 2013 (Ministério da Educação, Fundo Nacional de Desenvolvimento da Educação, Resolução No 26 de 17 de junho de 2013). In: Education Mo, editor. Brazil2013.

107. NOURISHING Framework - Offer healthy food and set standards in public institutions and other specific settings. [Internet]. 2016. Available from: https://www.wcrf.org/int/policy/nourishing-database.

108. Forskrift om rammeplan for barnehagens innhold og oppgaver. Forskrift om rammeplan for barnehagens innhold og oppgaver. FOR-2017-04-24-487 2017 [Available from: https://lovdata.no/dokument/SF/forskrift/2017-04-24-487?q=Forskrift%20om%20rammeplan%20for%20barnehagens.

109. Helsedirektoratet. Nasjonal faglig retningslinje. Mat og måltider i barnehagen 2018 [Available from: https://www.helsedirektoratet.no/retningslinjer/mat-og-maltider-i-barnehagen.

110. Folkehelseloven. Lov om folkehelsearbeid (folkehelseloven). LOV-2011-06-24-29 2011 [Available from: https://lovdata.no/dokument/NL/lov/2011-06-24-29?q=folkehelseloven.

111. Helsedirektoratet. Årsrapport 2017 (IS 2728). Oslo: Helsedirektoratet; 2018.

112. Birkelund HR. Dette mener partiene om gratis skolemat 2019 [Available from: https://frifagbevegelse.no/nyheter/dette-mener-partiene-om-gratis-skolemat-6.158.633073.2ac715ac48.

113. Health Service Executive Ireland. Healthier Vending 2019 [Available from: https://www.hse.ie/eng/about/who/healthwellbeing/our-priority-programmes/heal/healthier-vending/.

114. Lederer A, C.J. Curtis, L.D. Silver, and S.Y. Angell,. Toward a healthier city: nutrition standards for New York City government. Am J Prev Med 46(4): p. 423-8. 2014.

115. Department for Public Health Wales. Health Promoting Hospital Vending Directions and Guide 2008 2008 [Available from: https://gov.wales/sites/default/files/publications/2019-07/directions-to-local-health-boards-in-wales-and-velindre-national-health-service-trust-2012-no-5-guidance.pdf.

116. Netherlands Nutrition Centre. Guidelines for Healthier Canteens Fact sheet 2017 [Available from: https://mobiel.voedingscentrum.nl/Assets/Uploads/voedingscentrum/Documents/Professionals/Pers/Factsheets/English/Fact%20sheet%20Guidelines%20for%20Healthier%20Canteens.pdf.

117. Helsedirektoratet. Nasjonale anbefalinger for mat og drikke i arbeidslivet 2014 [Available from: https://www.helsedirektoratet.no/tema/kosthold-og-ernaering/matbransje-serveringsmarked-og-arbeidsliv/arbeidslivet-mat-og-drikketilbud-i-trad-med-kostradene.

118. Helsedirektoratet. Materiell og verktøy til hjelp i arbeidet 2014 [Available from: https://www.helsedirektoratet.no/tema/kosthold-og-ernaering/matbransje-serveringsmarked-og-arbeidsliv/arbeidslivet-mat-og-drikketilbud-i-trad-med-kostradene#materiell-og-verktoy-til-hjelp-i-arbeidet.

119. Helsedirektoratet. Kosthåndboken – veileder i ernæringsarbeid i helse- og omsorgstjenesten (IS-1972). Oslo: Helsedirektoratet; 2012.

120. Helsedirektoratet. Ernæring i helse- og omsorgstjenesten. Status, utfordringer og eksempler på gode tiltak. IS-2591. Oslo: Helsedirektoratet; 2017.

121. Helsedirektoratet. Helse- og omsorgstjenester til innsatte i fengsel. (IS-1971). Oslo: Helsedirektoratet; 2013.

122. Department of Health & Social Care UK. The Government Buying Standards for Food and Catering Services (GBSF): updating the nutrition standards 2019 [Available from: https://assets.publishing.service.gov.uk/government/uploads/system/uploads/attachment_data/file/802933/gbsf-nutrition-standards-consultation.pdf.

123. Healthy Eating Advisory Service. Improving wellbeing through healthy eating. 2017.

124. De gezonde schoolkantine. De Gezonde Schoolkantine. Jongeren zijn de toekomst n.d. [Available from: https://gezondeschoolkantine.voedingscentrum.nl/nl.aspx.

125. De gezonde schoolkantine. Check je kantine n.d. [Available from: https://gezondeschoolkantine.voedingscentrum.nl/nl/stap-voor-stap/kantinescan.aspx.

126. Helsedirektoratet. Matkurs for skolekantiner 2019 [Available from: https://www.helsedirektoratet.no/tema/mat-og-maltider-i-skolen/matkurs-for-skolekantiner.

127. Nasjonalt senter for mat helse og fysisk aktivitet. Påfyll kursopplegg – for ungdomsskolekantiner og matboder. 2019.

128. Healthy Ireland. A framework for improved health and wellbeing 2013-2025 2013 [Available from: https://www.hse.ie/eng/services/publications/corporate/hienglish.pdf.

129. The Special Act on the Safety Management of Children's Dietary Life. Available from: https://www.mfds.go.kr/eng/index.do?nMenuCode=66., (2017).

130. Bae SG, J.Y. Kim, K.Y. Kim, S.W. Park, J. Bae, and W.K. Lee, . Changes in dietary behavior among adolescents and their association with government nutrition policies in Korea, 2005-2009. J Prev Med Public Health, 45(1): p. 47-59. 2012.

131. NOURISHING Framework - Set incentives and rules to create a healthy retail and food service environment [Internet]. 2016. Available from: https://www.wcrf.org/int/policy/nourishing-database.

132. Helsedirektoratet. Lokale folkehelsetiltak – veiviser for kommunen 2018 [Available from: https://www.helsedirektoratet.no/faglige-rad/lokale-folkehelsetiltak-veiviser-for-kommunen.

133. GroentenFruit Huis. NAGF - National Fruit and Vegetable Action Plan n.d. [Available from: https://groentenfruithuis.nl/english/fields-of-activity/nutrition/nagf.

134. County of Los Angeles Public Health. Choose health LA - restaurants n.d. [Available from: http://www.choosehealthla.com/eat/restaurants/.

135. Helsedirektoratet. Serveringsmarkedet – mat- og drikketilbud i tråd med kostrådene 2017 [Available from: https://www.helsedirektoratet.no/tema/kosthold-og-ernaering/matbransje-serveringsmarked-og-arbeidsliv/serveringsmarkedet-mat-og-drikketilbud-i-trad-med-kostradene.

136. Kelly PM, A. Davies, A.J. Greig, and K.K. Lee, . Obesity Prevention in a City State: Lessons from New York City during the Bloomberg Administration. Front Public Health, 4: p. 60. 2016.

137. World Public Health Nutrition Association Update team. Brazilian dietary guidelines. At last! Guidelines based on food and meals!. Available from: http://wphna.org/wp-content/uploads/2015/01/WN-2014-05-12-1050-1051-Update-Guia.pdf. 2014.

138. Pobal & Government of Ireland. Healthy Ireland Fund 2016 [Available from: https://www.pobal.ie/programmes/healthy-ireland-fund/.

139. Government of the Netherlands. The National Prevention Agreement 2019 [Available from: https://www.government.nl/documents/reports/2019/06/30/the-national-prevention-agreement.

140. Berg O. Intensjonsavtalen for et sunnere kosthold mellom helsemyndighetene og matbransjen. Norsk tidsskrift for ernæring [Internet]. 2018; 3 2018. Available from: http://www.ntfe.no/i/2018/3/tfe-2018-03-b-3346.

141. Nordahl AM, Gjellerud I. – Uaktuelt med pappvinforbud nrk.no2019 [Available from: https://www.nrk.no/buskerud/_-uaktuelt-med-pappvinforbud-1.14397147.

142. Fossen CH. Listhaug blåser i «moralpolitiet»: – La folk røyke, drikke og spise rødt kjøtt 2019 [Available from: https://www.nrk.no/norge/folkehelseminister-listhaug-blaser-i-_moralpolitiet__-_-la-folk-royke_-drikke-og-spise-rodt-kjott-1.14539286.

143. Flydahl EF, Eertesvåg F. Søviknes ny folkehelseminister: Vil stumpe røyken og bygge ut statlig eldreomsorg 2019 [Available from: https://www.vg.no/nyheter/innenriks/i/lAWn8A/soeviknes-ny-folkehelseminister-vil-stumpe-roeyken-og-bygge-ut-statlig-eldreomsorg.

144. Ministry of Health Brazil. Health Surveillance Secretariat: Health situation analysis department. Strategic action plan to tackle noncommunicable diseases in Brazil 2011-2022. Ministry of Health: Brazil. 2011.

145. Ministry of Health South Africa. Strategic Plan for the Prevention and Control of Non-Communicable Diseases 2013-17. Available from:

http://www.hsrc.ac.za/uploads/pageContent/3893/NCDs%20STRAT%20PLAN%20%20CONTENT%208%20april%20proof.pdf. 2013.

146. Public Health England. Sugar Reduction: Achieving the 20%. A technical report outlining progress to date, guidelines for industry, 2015 baseline levels in key foods and next steps. 2017 [Available from: https://assets.publishing.service.gov.uk/government/uploads/system/uploads/attachment_data/file/604336/Sugar_reduction_achieving_the_20_.pdf.

147. The Central Government for the Netherlands. Agreed improvement product composition salt, saturated fat, sugar. 2014 [Available from: https://www.rijksoverheid.nl/documenten/convenanten/2014/01/23/akkoord-verbetering-productsamenstelling-zout-verzadigd-vet-suiker.

148. Departementene. Nasjonal handlingsplan for bedre kosthold (2017–2021). Sunt kosthold, måltidsglede og god helse for alle! Oslo: Helse- og omsorgsdepartementet,; 2017.

149. Monteiro CA, G. Cannon, J.C. Moubarac, A.P. Martins, C.A. Martins, J. Garzillo, P.C. Jaime,. Dietary guidelines to nourish humanity and the planet in the twenty-first century. A blueprint from Brazil. Public Health Nutr, 18(13): p. 2311-22. 2015.

150. Ministry of Health Brazil. Dietary guidelines for the Brazilian population. Available from: http://www.foodpolitics.com/wp-content/uploads/Brazilian-Dietary-Guidelines-2014.pdf. 2014.

151. Department of Health Ireland. A Healthy Weight for Ireland: Obesity Policy and Action Plan 2016 – 2025 2016 [Available from: https://www.gov.ie/en/publication/c778a9-a-healthy-weight-for-ireland-obesity-policy-and-action-plan-2016-202/?referrer=/wp-content/uploads/2016/09/a-healthy-weight-for-ireland-obesity-policy-and-action-plan-2016-2025.pdf/.

152. Food Safety Authority of Ireland. Scientific Recommendations for Healthy Eating Guidelines in Ireland 2011 [Available from: https://www.lenus.ie/handle/10147/216750.

153. Healthy Ireland. Healthy Food for Life. Revised healthy eating guidelines and Food Pyramid rationale 2016 [Available from: https://www.hse.ie/eng/about/who/healthwellbeing/our-priority-programmes/heal/food-pyramid-images/foodforlifefoodpyramidrationale2016.pdf.

154. Health Council of the Netherlands. Dutch dietary guidelines 2015 2015 [Available from: https://www.healthcouncil.nl/documents/advisory-reports/2015/11/04/dutch-dietary-guidelines-2015.

155. Nasjonalt råd for ernæring. Kostråd for å fremme folkehelsen og forebygge kroniske sykdommer: metodologi og vitenskapelig kunnskapsgrunnlag. Oslo: Helsedirektoratet; 2011.

156. Nordic Council of Ministers. Nordic nutrition recommendations: NNR 2011 : integrating nutrition and physical activity. 4th ed. ed. Copenhagen: Nordic Council of Ministers; 2011.

157. Helsedirektoratet. Slik kan du sette sammen et sunt måltid 2018 [Available from: https://helsenorge.no/kosthold-og-ernaring/kostrad/dagens-maltider.

158. Folkehelseinstituttet. Midtveisevaluering av Nasjonal handlingsplan for bedre kosthold (2017-2021). Oslo: Folkehelseinstituttet; 2019.

159. Australian Institute of Health and Welfare. National Indigenous Reform Agreement (2019) 2019 [Available from: https://meteor.aihw.gov.au/content/index.phtml/itemId/697092.

160. Department of Health Ireland. Taoiseach and Ministers launch Healthy Ireland campaign 2019 [Available from: https://www.gov.ie/en/news/20cff4-taoiseach-and-health-ministers-to-launch-2019-healthy-ireland-campai/.

161. Finansdepartementet. Muligheter for alle. Fordeling og sosial bærekraft (Meld. St. 13 (2018 – 2019). Oslo: Finansdepartementet; 2018.

162. Fagrådet for sosial ulikhet i helse. Anbefalte tiltak mot sosial ulikhet i helse (IS-2749). Oslo: Helsedirektoratet; 2018.

163. Lobby register. n.d. [Available from: https://en.wikipedia.org/wiki/Lobby_register.

164. State Services Commission. Best Practice Guidelines for Departments Responsible for Regulatory Processes with Significant Commercial Implications. Available from: http://www.ssc.govt.nz/resources/2814/all-pages.

165. Australian Public Service Commission. Code of Conduct 2018 [Available from: https://www.apsc.gov.au/code-conduct.

166. Standards in Public Office Commission. 2018 Code of Conduct for persons carrying on lobbying acitivities 2018 [Available from: https://www.lobbying.ie/media/6119/code-of-conduct-english-final-version-for-web.pdf.

167. Transparency International. Corruptions Perception Index 2018 [Available from: https://www.transparency.org/cpi2018.

168. Transparency International Norge. Korrupsjonsindeksen 2018 2019 [Available from: http://transparency.no/2019/01/29/korrupsjonsindeksen-2018/.

169. Forvaltningsloven. Lov om behandlingsmåten i forvaltningssaker (forvaltningsloven) 1967 [Available from: https://lovdata.no/dokument/NL/lov/1967-02-10/KAPITTEL_2#KAPITTEL_2.

170. Vitenskapskomiteen for mat og miljø. Regler om habilitet ved VKM 2018 [Available from: https://vkm.no/download/18.7e19596115dabec04172b138/1535092702082/Regler%20om%20habilitet%20for%20VKM.pdf.

171. Berg O. Norwegian experience on engaging non-state actors in nutrition 2019 [Available from: https://www.who.int/nutrition/events/2019-memberstates-consultation-COI-4Feb-presentation-Norway.pdf?ua=1.

172. National Health and Medical Research Council Act 1992. Act No. 225 of 1992 as amended. 2006.

173. Forskrift om oversikt over folkehelsen. Forskrift om oversikt over folkehelsen. FOR-2012-06-28-692. 2012.

174. Helsedirektoratet. Folkehelsepolitisk rapport 2018 [Available from: https://www.helsedirektoratet.no/rapporter/folkehelsepolitisk-rapport.

175. Folkehelseinstituttet. Folkehelserapporten - Helsetilstanden i Norge n.d. [Available from: https://www.fhi.no/nettpub/hin/.

176. Helsedirektoratet. Utvikling av kunnskapsbaserte retningslinjer 2012 [Available from: https://www.helsedirektoratet.no/veiledere/utvikling-av-kunnskapsbaserte-retningslinjer.

177. Helsedirektoratet. Nasjonalt råd for ernæring 2019 [Available from: https://www.helsedirektoratet.no/om-oss/organisasjon/rad-og-utvalg/nasjonalt-rad-for-ernaering.

178. Nasjonalt råd for ernæring. Kostråd om fett - en oppdatering og vurdering av kunnskapsgrunnlaget (IS-2625). Oslo: Helsedirektoratet; 2017.

179. Helsedirektoratet. Nordic Nutrition Recommendations 2022 2019 [Available from: https://www.helsedirektoratet.no/english/nordic-nutrition-recommendations-2022.

180. Food Standards Australia New Zealand. Stakeholder Engagement Strategy 2013-2016. FSANZ New Zealand: Wellington. 2013.

181. Kommunal- og moderniseringsdepartementet. Hva er Open Government Partnership (OGP)? n.d. [Available from: https://open.regjeringa.no/open-government-partnership/.

182. Offentleglova. Lov om rett til innsyn i dokument i offentleg verksemd. LOV-2006-05-19-16 https://lovdatano/dokument/NL/lov/2006-05-19-162019.

183. Arkivlova. Lov om arkiv [arkivlova]. LOV-1992-12-04-126 1992 [Available from: https://lovdata.no/dokument/NL/lov/1992-12-04-126?q=arkivloven.

184. Finansdepartementet. Utredningsinstruksen 2016 [Available from: https://www.regjeringen.no/no/dokumenter/instruks-om-utredning-av-statlige-tiltak-utredningsinstruksen/id2476518/.

185. Citizens Information. Freedom of information 2018 [Available from: https://www.citizensinformation.ie/en/government_in_ireland/national_government/standards_and_accountability/freedom_of_information.html.

186. Health Registries for Research Norway. HRR metadatabase for Nasjonale Helseregistre n.d. [Available from: http://hrr.uit.no/hrr/.

187. Helse- og omsorgsdepartementet. Høring - tilgjengeliggjøring av helsedata (endringer i helseregisterloven m.m.) 2019 [Available from: https://www.regjeringen.no/no/dokumenter/tilgjengeliggjoring-av-helsedata/id2662764/.

188. FAO. International food composition table/database directory 2017 [Available from: http://www.fao.org/infoods/infoods/tables-and-databases/en/.

189. German Nutrition Society. Nutrition Reports n.d. [Available from: https://www.dge.de/en/nutrition-reports/.

190. Robert Koch Institut. The AdiMon Indicator System 2019 [Available from: https://www.rki.de/EN/Content/Health_Monitoring/HealthSurveys/AdiMon/AdiMon_inhalt.html.

191. Netherlands Food Database [Internet]. n.d. . Available from: https://www.levensmiddelendatabank.nl/Security/Login.aspx?ReturnUrl=%2f.

192. Mattilsynet. Matvaretabellen n.d. [Available from: https://www.matportalen.no/verktoy/matvaretabellen/#tabs-1-1-anchor.

193. Helsedirektoratet. Referat fra koordineringsgruppemøte 2017 [Available from: https://www.helsedirektoratet.no/tema/kosthold-og-ernaering/matbransje-serveringsmarked-og-arbeidsliv/intensjonsavtalen-for-et-sunnere-kosthold/Referat%20fra%20koordineringsgruppem%C3%B8te%203%20%E2%80%93%202017-09-07.pdf/_/attachment/inline/bb0dc1e0-4e3e-4e1c-8ae3-3f92fcfa3d32:4ee6fb09edde0452be7005d5520626694a860612/Referat%20fra%20koordineringsgruppem%C3%B8te%203%20%E2%80%93%202017-09-07.pdf.

194. Mattilsynet. Tilsyn og kontroll av nøkkelhullmerking 2018 [Available from: https://www.mattilsynet.no/mat_og_vann/merking_av_mat/nokkelhullet/tilsyn_og_kontroll_av_nokkelhullmerking.33078.

195. Nordic Council of Ministers. Monitoring food marketing to children: A joint Nordic monitoring protocol for marketing of foods and beverages high in fat, salt and sugar (HFSS) towards children and young people 2018 [Available from: http://norden.diva-portal.org/smash/record.jsf?dswid=3428&faces-redirect=true&language=no&searchType=LIST_LATEST&query=&af=%5B%5D&aq=%5B%5B%5D%5D&aq2=%5B%5B%5D%5D&aqe=%5B%5D&pid=diva2%3A1183357&noOfRows=50&sortOrder=author_sort_asc&sortOrder2=title_sort_asc&onlyFullText=false&sf=all.

196. Centres for Disease Control. National Health and Nutrition Examination Survey. Available from: http://www.cdc.gov/nchs/nhanes.htm. 2016.

197. The Dutch Institute for Public Health and the Environment. Overview of food consumption surveys 2019 [Available from: https://www.rivm.nl/voedselconsumptiepeiling/overzicht-voedselconsumptiepeilingen.

198. Folkehelsinstituttet. Om de nasjonale kostholdsundersøkelsene 2017 [Available from: https://www.fhi.no/studier/nasjonale-kostholdsundersokelser/om-de-nasjonale-kostholdsundersokelsene/.

199. UiO Institutt for medisinske basalfag. 2019 [Available from: https://www.med.uio.no/imb/forskning/prosjekter/smabarnskost/.

200. UiO Institutt for medisinske basalfag. Spedkost 3 2019 [Available from: https://www.med.uio.no/imb/forskning/prosjekter/spedkost/.

201. UiT Norges arktiske universitet. Tromsøundersøkelsen 2019 [Available from: https://uit.no/forskning/forskningsgrupper/gruppe?p_document_id=367276.

202. NTNU, HUNT Forskningssenter. HUNT 2019 [Available from: https://www.ntnu.no/hunt.

203. Folkehelseinstituttet. Indikatorer for ikke-smittsomme sykdommer knyttet til den nasjonale og globale NCD-strategien n.d. [Available from: https://www.fhi.no/nettpub/ncd/.

204. Health and Social Care Information Centre. National Child Measurement Programme. Available from: http://www.hscic.gov.uk/ncmp. 2016.

205. National Nutrition Surveillance Centre. Childhood Obesity Surveillance Initiative (COSI) n.d. [Available from: http://www.ucd.ie/nnsc/research/ourresearch/childhoodobesitysurveillanceinitiativecosi.

206. Folkehelseinstituttet. Utvikling av indikatorer på NCD-området knyttet til rapportering for den globale og nasjonale NCD-strategien 2017 [Available from: https://www.fhi.no/publ/2017/utvikling-av-indikatorer-pa-ncd-omradet-knyttet-til-rapportering-for-den-gl/.

207. US National Institutes of Health. Time-Sensitive Obesity Policy and Program Evaluation (R01). Available from: http://grants.nih.gov/grants/guide/pa-files/PAR-12-257.html. 2016.

208. The Dutch Institute of Public Health and Environment. Agreed improvement product composition salt, saturated fat, sugar. 2017 [Available from: https://www.rijksoverheid.nl/documenten/convenanten/2014/01/23/akkoord-verbetering-productsamenstelling-zout-verzadigd-vet-suiker.

209. Den nasjonale NCD gruppen. Sosiale forskjeller i ikke smittsomme sykdommer 2019 [Available from: https://www.slideshare.net/Folkehelseinstituttet/del-1-2019-sosiale-forskjeller-i-ikke-smittsomme-sykdommer?qid=d7965e01-c537-4add-83c2-554db76b06dc&v=&b=&from_search=3.

210. Finansdepartementet. Prop. 1 S Gul bok (2019–2020) 2019 [Available from: https://www.regjeringen.no/no/dokumenter/prop.-1-s-gul-bok-20192020/id2671985/?ch=1.

211. Irish Department of Agriculture Food and the Marine. Food Institutional Research Measure (FIRM) 2017 [Available from: https://www.agriculture.gov.ie/research/competitivenationalprogrammes/foodinstitutionalresearchmeasurefirm/.

212. The Irish Health Research Board. Research. Evidence. Action. HRB Strategy 2016-2020 2016 [Available from: http://hrbstrategy.ie/wp-content/uploads/2016/01/HRB_Strategy.pdf.

213. Helse- og omsorgsdepartementet. Tildelingsbrevet til Norges forskningsråd for 2019 2018 [Available from: https://www.regjeringen.no/globalassets/departementene/hod/tildeling-oppdrag-og-arsrapporter/2019/tildelingsbrev-norges-forskningsrad-for-budsjettaret-2019.pdf.

214. Forskningsrådet. Prosjektbanken n.d. [Available from: https://prosjektbanken.forskningsradet.no/.

215. The Federal Centre for Nutrition. The Federal Centre for Nutrition (BZfE) n.d. [Available from: http://www.bzfe.de/.

216. The Dutch Nutrition Center. Over het Voedingscentrum 2018 [Available from: https://www.voedingscentrum.nl/nl/service/over-ons.aspx.

217. Helse- og omsorgsdepartementet. Statsbudsjettet 2019 - kap. 740 Helsedirektoratet - tildeling av bevilgning 2018 [Available from: https://www.regjeringen.no/globalassets/departementene/hod/tildeling-oppdrag-og-arsrapporter/2019/statsbudsjettet-2019---kap.-740-helsedirektoratet---tildeling-av-bevilgning1405121.pdf.

218. Helsedirektoratet. Dette gjør Helsedirektoratet 2017 [Available from: https://www.helsedirektoratet.no/om-oss/dette-gjor-helsedirektoratet.

219. Finansdepartementet. 8.1 Avbyråkratiserings- og effektiviseringsreformen 2018 [Available from: https://www.regjeringen.no/no/dokumenter/prop.-1-s-gul-bok-20192020/id2671985/sec8.

220. UK Department of Health and Social Care. Public health responsibility deal 2011 [Available from: https://www.gov.uk/government/news/public-health-responsibility-deal.

221. Norwegian Directorate of Health. Partnership for a healthier diet 2016 [Available from: https://www.helsedirektoratet.no/english/partnership-for-a-healthier-diet.

222. CONSEA. Building up the National Policy and System for Food and Nutrition Security: the Brazilian experience. Brasilia: CONSEA; 2009.

223. Ministry of Health New Zealand. Healthy Families NZ. Available from: http://www.health.govt.nz/our-work/preventative-health-wellness/healthy-families-nz. 2016.

224. Government of South Australia. South Australia's HiAP approach 2018 [Available from: https://www.sahealth.sa.gov.au/wps/wcm/connect/public+content/sa+health+internet/about+us/about+sa+health/health+in+all+policies/south+australias+hiap+approach.

225. Helse- og omsorgstjenesteloven. Lov om kommunale helse- og omsorgstjenester m.m. (helse- og omsorgstjenesteloven). LOV-2011-06-24-30 2011 [Available from: https://lovdata.no/dokument/NL/lov/2011-06-24-30.

226. Lock K, M. Gabrijelcic-Blenkus, M. Martuzzi, P. Otorepec, P. Wallace, C. Dora, J.M. Zakotnic,. Health impact assessment of agriculture and food policies: lessons learnt from the Republic of Slovenia. Bull World Health Organ, 81(6): p. 391-8. 2003.

227. Helse- og omsorgsdepartementet. Folkehelseloven 2019 [Available from: https://www.regjeringen.no/no/tema/helse-og-omsorg/folkehelse/innsikt/folkehelsearbeid/id673728/.
