# Supplementary material for: The Healthy Food Environment Policy Index in Poland: Implementation Gaps and Actions for Improvement
Source: Foods. 2022 Jun 2;11(11):1648. doi: 10.3390/foods11111648 (PMC9180244; doi:10.3390/foods11111648)
Supplement: Supplementary file 1 [file foods-11-01648-s001.zip › File 2 International Best Practices.pdf]

ANNEX 2: BEST PRACTICE EXEMPLARS (2019)

The Best practice Exemplars are based on the version of the INFORMAS benchmarks from August 2017

DOMAIN 1 - FOOD COMPOSITION

**COMP1**	
**Food composition targets/standards/restrictions have been established by the government for the content of the nutrients of concern (added sugars, salt, saturated fat) in industrially processed foods, in particular for those food groups that are major contributors to population intakes of those nutrients of concern**<br>**(Trans fat has been excluded as it falls under EU regulation)**	
**International best practice examples (benchmarks)**	**UK:** In 2016**,** a key commitment of the 'Childhood obesity: a plan for action' was to launch a broad, structured sugar reduction programme to remove sugar from everyday products. All sectors of the food and drinks industry were challenged to reduce overall sugar across a range of products that contribute most to children's sugar intakes by at least 20% by 2020, including a 5% reduction in the first year of the programme (August 2016 to August 2017). The overall reduction between 2015 - 2018 (in total sugar per 100g) was -2.9%(Public Health England, 2019). Only three food groups of the eight measured have managed at least a 5% reduction in the first year: sweet spreads and sauces, yoghurts and fromage frais, and breakfast cereals. There has been no sugar reduction in biscuits and chocolate bars(Public Health England, 2018). In contrast to this co-regulation, for products where the sugar tax applies over the same period a reduction in sugar of about 30% was found.<br><br>**South Africa:** In 2013, the South African Department of Health adopted mandatory targets for salt reduction in 13 food categories by means of regulation (Foodstuffs, Cosmetics and Disinfectants Act). There is a stepped approach with food manufacturers given until June 2016 to meet one set of category-based targets and another three years until June 2019 to meet the next (World Cancer Research Fund, 2016b, Hofman and Tollman, 2013). Overall, 67% of targeted foods had a sodium level at or below the legislated limit. About half (49%) of targeted foods not meeting the legislated limits were less than 25% above the maximum sodium level (Peters et al., 2017).<br><br>**The Netherlands:** On January 2014, the Dutch Ministry of Health, Welfare and Sport signed an agreement with trade organizations representing food manufacturers, supermarkets, hotels, restaurants, caterers and the hospitality industry to lower the levels of salt, saturated fat and calories in food products. The agreement includes voluntary ambitions for the period up to 2020 and aims to increase the healthiness of the food supply (World Cancer Research Fund, 2016b, National Agreement to improve Product Composition, 2017). Voluntary agreements have been made for the reduction of salt, saturated fat and energy/sugars in a variety of product groups and soft drinks.

COMP2	
**Food composition targets/standards/restrictions have been established by the government for the content of the nutrients of concern (added sugars, salt, saturated fat) in meals sold from food service outlets, in particular for those food groups that are major contributors to population intakes of those nutrients of concern.** **(Trans fat has been excluded as it falls under EU regulation)**	
**International best practice examples (benchmarks)**	**New Zealand:** In New Zealand, The Chip group, funded 50% by the Ministry of Health and 50% by industry, aims to improve the nutritional quality of deep-fried chips served by food service outlets by setting an industry standard for deep frying oils. The standard for deep frying oil is maximum 28% of saturated fat, 3% linoleic acid and 1% of trans-fat. The Chip group oil logo for use on approved oil packaging was developed in 2010 (The Chip Group, 2016).<br><br>**New York City (US):** In 2009, New York City established voluntary salt guidelines for restaurant and store-bought foods. In 2010, this evolved into the National Salt Reduction Initiative that encouraged nationwide partnerships among food manufacturers and restaurants involving more than 100 city and state health authorities to reduce excess sodium by 25% in packaged and restaurant foods. In 2012, 26% of the categories met the targets, and 3% met the targets by the end of 2014. Between 2009 and 2014, there was nearly a 7% reduction in sodium levels in the U.S. food supply (New York City Health, 2017). There are 28 companies, including packaged food corporations and restaurants, who are committed to the salt reduction targets (Department of Health, 2014). In July 25, 2019, the Voluntary Sugar Reduction Targets from the National Salt and Sugar Reduction Initiative were revised. There is an open technical comment period until September 30, 2019 (NYC Health Department, 2019).

DOMAIN 2 – FOOD LABELLING

**LABEL1**
**Ingredient lists and nutrient declarations in line with Codex recommendations are present on the labels of all packaged foods**
Label 1 will not be included for benchmarking at national level as it’s being addressed at EU level

**LABEL2**
**Evidence-based regulations are in place for approving and/or reviewing claims on foods, so that consumers are protected against unsubstantiated and misleading nutrition and health claims.**
Label 2 will not be included for benchmarking at national level as it’s being addressed at EU level

**LABEL3**	
**One or more interpretive, evidence-informed front-of-pack supplementary nutrition information system(s) endorsed by the Government, which readily allow consumers to assess a product’s healthiness, is/are applied to all packaged foods (examples are the Nutri-Score and traffic lights).**	
**International best practice examples (benchmarks)**	**UK:** In 2013, the Government published national guidance for voluntary 'traffic light' labelling for use on the front of pre-packaged food products. The label uses green, amber and red to identify whether products contain low, medium or high levels of energy, fat, saturated fat, salt and sugar. A combination of colour coding and nutritional information is used to show how much fat, salt and sugar and how many calories are in each product. The voluntary scheme is used by all the major retailers and some manufacturers (Deparment of Health, 2013).<br><br>**Australia/New Zealand:** The government-approved, voluntary 'Health Star Rating' (HSR) scheme applies a stare rating system where ratings range from ½ star (least healthy) to 5 stars (most healthy). The rating is based on the content of energy, saturated fat, sodium and total sugars content, along with certain 'positive' aspects of a food such as fruit and vegetable content, and in some instances, dietary fibre and protein content. Implementation of the system began in June 2014 and is overseen by a number of governmental instances, one of which evaluates progress. As of 2016, about 900 products had stars on them (Commonwealth of Australia, 2016).<br><br>**France/ Belgium/Germany/Spain:** Since October 2017, the five-colour NutriScore, the official voluntary label for France has been implemented as the official, voluntary FOP scheme in four European countries. It aims to limit the consumption of foods high in energy, saturated fats, sugar or salt, in the context

	of an overall improvement in the nutritional quality of diets (World Health Organisation, 2017). Based on a scientific algorithm, each product is given a score based on the content of the nutrients of concern (energy value and the amount of sugars, saturated fats and salt) and positive ones (the amount of fibre, protein, fruit, vegetables and nuts) (Colruyt Group, 2018). The system was developed by the Nutritional Epidemiology research Team at the University of Paris (Chantal, 2017).<br><br>**Table1: The Nutri-Score (Colruyt Group, 2018)**<br><br>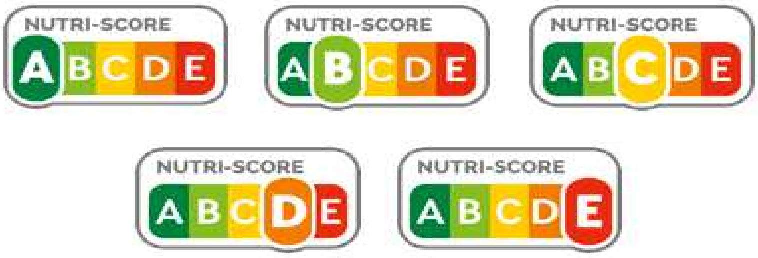

LABEL4	
**A simple and clearly-visible system of labelling the menu boards of all quick service restaurants (i.e. fast food chains) is applied by the government, which allows consumers to interpret the nutrient quality and energy content of foods and meals on sale.**	
**International best practice examples (benchmarks)**	**South Korea**: Since 2010, the Special Act on Safety Control of Children's Dietary Life has required all chain restaurants with 100 or more establishments to display nutrient information on menus including energy, total sugars, protein, saturated fat and sodium (World Cancer Research Fund, 2016c).<br><br>**Canada:** In effect since 1 January 2017, Ontario's Healthy Menu Choices Act 2015, requires food service premises that are part of a chain of 20 or more food service premises in Ontario (as well as certain cafeteria-style food service premises) to display calories for "standard food items" on menus, labels and display tags. The Act's regulations specify where caloric information is to be displayed on the menus, as well as the size, format and prominence of the display (Nutrition Resource Centre, 2017). Food service premises must also display information on daily caloric requirements: "*Adults and youth (ages 13 and older) need an average of 2,000 calories a day, and children (ages 4 to 12) need an average of 1,500 calories a day. However, individual needs vary."*<br>Ontario's 36 public health units are responsible for implementation of the Act (Nutrition Resource Centre, 2017)<br><br>**Saudi Arabia**: In 2018, the Saudi Food and Drug Authority (SFDA) introduced mandatory measures on calorie labels on menus. These measures apply to all food facilities including restaurants, ice cream parlours, juice and fresh fruit vendors, bakeries, sweets shops, cafeterias, supermarkets, recreation facilities, colleges, universities and government agencies. Calories will be displayed at cashier desks, menu boards, table menus, drive-through menus, phone and web applications (Saudi Food & Druf Authority, 2018)<br><br>**USA:** In the US, the Patient Protection and Affordable Care Act (2010) (Office of the Federal Register, 2013) requires that all chain restaurants with 20 or more establishments to display energy information on menus. The menu labelling rule was implemented in May 2018 (Administration, 2019). The regulations will be pre-empted by the national law once implemented; local governments will still be able to enact menu labelling regulations for establishments not covered by national law. The regulations require vending machine operators of more than 20 vending machines to post calories for foods where the on-pack label is not visible to consumers by 26 July 2018 (World Cancer Research Fund, 2016c).<br><br>**Australia:** Legislation in Australian Capital Territory (Food Regulation 2002) and the States of New South Wales (Food Regulation 2010) and South Australia (Food Regulation 2002) requires restaurant chains (e.g. fast food chains, ice cream bars) with ≥20 outlets in the state (or seven in the case of ACT), or 50 or more across Australia, to display the kilojoule content of food products on their menu boards.

	Average adult daily energy intake of 8700kJ must also be prominently featured. Other chains/food outlets are allowed to provide this information on a voluntary basis but must follow the provisions of the legislation (World Cancer Research Fund, 2016c).<br><br>**New York City, USA:** Chain restaurants are required to put a warning label on menus and menu boards, in the form of a salt-shaker symbol (saltshaker inside a triangle), when dishes contain 2,300 mg of sodium or more. It applies to food service establishments with 15 or more locations nationwide. In addition, a warning statement is required to be posted at the point of purchase: This came into effect 1 December 2015 (World Cancer Research Fund, 2016c) (Department of Health and Mental Hygiene) and the Health Department started issuing violations June 6, 2016. Findings showed that directly following the sodium warning label regulation coming into effect, about 21% of restaurants had implemented the labels. By the end of February 2015, almost 70% of restaurants (from six of the ten chains) had implemented labels at one location or more. Overall, the findings suggested that the majority of restaurants were complying with the sodium warning label policy, despite issues with visibility, but that the labels may not be influencing consumer purchasing decisions (Downs, 2017).

DOMAIN 3 - FOOD PROMOTION

PROMO1	
**Effective policies are implemented by the government to restrict exposure and power of promotion of unhealthy foods to children including adolescents through broadcast media (TV, radio).**	
**International best practice examples (benchmarks)**	**Quebec:** In Québec, the Consumer Protection Act prohibits commercial advertising (including food and non-food) directed at children less than 13 years of age through television, radio and other media. Account must be taken of the context of its presentation, and in particular of: a) the nature and intended purpose of the goods advertised; b) the manner of presenting such advertisement; and c) the time and place it is shown. A cut-off of 15% share of child audience is used to protect children from TV advertising (Kent et al., 2011). Per indictment, a person is liable to: a fine ranging from $600 to $15,000 (in the case of a natural person); a fine ranging from $2,000 to $100,000 (in the case of a legal person).<br><br>**Norway/Sweden:** Under the Broadcasting Act, advertisements (food and non-food) may not be broadcast on television directed to children or in connection with children's programs. (World Cancer Research Fund, 2016e). Norway has implemented a self-regulation scheme approved and evaluated by Government. The scheme prohibits child-targeted unhealthy food marketing before 21:00 (9 PM) (MFU, 2016)<br><br>**Ireland:** Advertising, sponsorship, teleshopping and product placement of foods high in fats, sugars and salt, as defined by a nutrient profiling model, are prohibited during children's TV and radio programmes where over 50% of the audience are under 18 years old (Broadcasting Authority of Ireland, 2013). In addition, there is an overall limit on advertising of foods high in fats, sugars and salt adverts at any time of day to no more than 25% of sold advertising time and to only one in four advertisements. Remaining advertising targeted at children under the age of 18 must not include nutrient or health claims or include licensed characters (World Cancer Research Fund, 2016). As provided under the Broadcasting Act 2009, the BAI is required to undertake a statutory review of the effectiveness of the Children's Code. It is expected that review will commence in the second half of 2018 with revision and finalisation of the Code in 2019 (Broadcasting Authority of Ireland, 2019).<br><br>**Chile:** In June 2016, The Law of Nutritional Composition of Food and Advertising was enforced and restricts advertising directed to children under 14 years (for foods exceeding limits for calories, sugar, saturated fat and/or sodium in food and beverages). The regulatory norms define advertising targeted to children as programmes with an audience of greater than 20% children. Promotional strategies and incentives, such as cartoons, animations, and toys that could attract the attention of children are included in the ban. Monitoring and enforcement of the law are carried out by both regional and national public health authorities. Inspections are conducted on-site and online. After more than 2000 inspections, compliance with the law is improving, rising from under 40% to over 60% (Global Agricultural Information Network, 2018). A qualitative study carried out in 2017, found that the regulation has

	made mothers more aware of the importance of eating healthy, made it easier to choose healthy foods, and also made children actors in their own food choices (Correa et al., 2019).

**PROMO2**	
**Effective policies are implemented by the government to restrict exposure and power of promotion of unhealthy foods to children including adolescents through online and social media.**	
**International best practice examples (benchmarks)**	**Chile:** In June 2015, the Chilean authority approved the regulatory norms required for the law of Nutritional Composition of Food and Advertising implementation. The regulatory norms define limits for calories, saturated fat, sugar and sodium content considered "high" in foods and beverages. The law restricts advertising directed to children under the age of 14 years of foods in the "high in" category. The regulatory norms define advertising targeted to children as websites directed to children or with an audience of greater than 20% children, and according to the design of the advertisement. Promotional strategies and incentives, such as cartoons, animations, and toys are included in the ban. The regulation took effect 1 July 2016 and applies to all advertising media (World Cancer Research Fund, 2016).<br><br>**Quebec:** In Québec, the Consumer Protection Act prohibits commercial advertising directed at children less than 13 years of age through all media. Account must be taken of the context of its presentation, and in particular of: a) the nature and intended purpose of the goods advertised; b) the manner of presenting such advertisement; and c) the time and place it is shown (Kent et al., 2011). Any stakeholder involved in a commercial process (from the request to create an advertisement to its distribution, including its design) may be accused of not complying with the legislation in force. Per indictment, that person is liable to: a fine ranging from $600 to $15,000 (in the case of a natural person); a fine ranging from $2,000 to $100,000 (in the case of a legal person).<br><br>**Norway**: A government-approved and evaluated self-regulation scheme prohibits online food-marketing which is targeted at children under 13 years. Specifically, interactive games "specifically aimed at children and where a product's trademark, or other elements of the marketing of the product, form an integral part" will always be defined as child-targeted and a violation of the code (36). The scheme also applies to social media. In 2019, the code was revised. The age limit is still 13 years but in order to exercise caution in marketing to young people, it is advised to not conduct contests with age limit less than 16 years; to buy age groups less than 16 years in digital media; to use role models appealing to youth in media which is directly targeted to youth; and to encourage engagement (share, like, send in material) so that youth become marketing actors. Violations of the code results in no other sanctioning than "naming and shaming" of offenders (Matbransjens Faglige Utvalg, 2019).

	**UK:** UK CAP rules have been reviewed so that online marketing targeted to under-16s is prohibited. This means that HFSS product ads are not permitted to appear in media that is specifically targeted at under-16s (for example, a children's magazine or on a website aimed at children); or where under-16s make up a significant proportion (more than 25%) of the audience (for example, advertorial content with an influencer that might have broad appeal but also a significant child audience) (Advertising Standards Authority, 2018).

PROMO3	
**Effective policies are implemented by the government to restrict exposure and power of promotion of unhealthy foods to children including adolescents through non-broadcast media other than packaging and online/social media.**	
**International best practice examples (benchmarks)**	**Chile:** In May 2018, a new regulation launched, extended marketing restrictions of regulated foods in cinema and TV to a 6 AM to 10 PM time frame, expanding the scope of the original law. Additionally, starting in June 2018, any marketing done for "High in" foods or beverages must also show the following statement: "Choose foods with less warning labels" and then "Ministry of Health," which needs to be placed next to the MoH logo. This applies to marketing done in billboard, cinema, and other vehicles but food packages (Corvalán C, 2018).<br><br>**London UK:** On 25 February 2019, the Mayor of London, introduced restrictions on the advertising of unhealthy food across the entire Transport for London (TfL) public transport network, as part of his work to help tackle childhood obesity in London. The policy specifies that food and non-alcoholic drinks high in fat, salt and sugar (according to the UK Nutrient Profiling Model, are not permitted to be advertised on TfL-controlled buses, underground and over ground train networks, taxis, river services, trams and other transport systems. Food and drink brands, restaurants, takeaways and ordering services are required to promote their healthier food and drink instead of just advertising their brand. Advertisements for food and non-alcoholic drink products that are considered to be high in fat, salt, sugar may be considered for an exception by TfL if the advertiser can demonstrate, with appropriate evidence, that the product does not contribute to child obesity (Greater London Authority, 2019)

PROMO4	
**Effective policies are implemented by the government to ensure that unhealthy foods are not commercially promoted to children including adolescents in settings where children gather (e.g. preschools, schools, sport and cultural events).**	
**International best practice examples (benchmarks)**	**Chile:** In June 2015, the Chilean authority approved the regulatory norms required for the Law of Nutritional Composition of Food and advertisings implementation. The regulatory norms define limits for calories, saturated fat, sugar and sodium content considered “high” in foods and beverages. The law restricts advertising directed to children under the age of 14 of foods in the “high in” category on school grounds, including preschools, primary and secondary schools. Chile has also restricted outdoor advertising, with ten municipalities adopting legislations banning outdoor marketing one block around schools (The Organisation for Economic Co-operation and Development, 2019). The law is scheduled to take effect in July 2016 (New York City Health, 2017) has also restricted outdoor advertising, with ten municipalities adopting legislations banning outdoor marketing one block around schools. The law is scheduled to take effect in July 2016 (New York City Health, 2017).<br><br>**Uruguay:** In September 2013, the government of Uruguay adopted Law No 19.140 (Healthy foods in schools) (Morley et al., 2013). The law prohibits the advertising and marketing of foods and drinks that do not meet the nutrition standards. Advertising in all forms is prohibited, including posters, billboards, and use of logos/brands on school supplies, sponsorship, and distribution of prizes, free samples on school premises and the display and visibility of food. The implementation of the law started in 2015 (World Cancer Research Fund, 2016).<br><br>**Hungary:** Based on Section 8 of Act XLVIII on Basic Requirements and Certain Restrictions of Commercial Advertising Activities (2008), Hungary prohibits all advertising directed at children under 18 in child welfare and child protection institutes, kindergartens, elementary schools and their dormitories. Health promotion and prevention activities in schools may only involve external organizations and consultants who are recommended by the National Institute for Health Development (World Cancer Research Fund, 2016e).<br><br>**Spain:** In 2011 the Spanish Parliament approved a Law on Nutrition and Food Safety, which stated that kindergartens and schools should be free from all advertising. Criteria for the authorisation of food promotion campaigns, nutritional education and promotion of sports or physical activity campaigns were developed jointly by the Spanish Agency for Consumer Affairs, Food Safety and Nutrition (AECOSAN) and the Regional Health Authorities and implemented in July 2015. AECOSAN and the Spanish Regional Education and Health Administrations monitor the enforcement of the law (World Cancer Research Fund, 2016)

PROMO5	
**Effective policies are implemented by the government to ensure that unhealthy foods are not commercially promoted to children including adolescents, on food packages.**	
**International best practice examples (benchmarks)**	To be checked with the legal expert Amandine Garde)

DOMAIN 4 - FOOD PRICES

PRICES1	
**Taxes or levies on healthy foods are minimised to encourage healthy food choices (e.g. low or no sales tax, excise, value-added or import duties on fruit and vegetables).**	
**International best practice examples (benchmarks)**	**Australia:** Goods and services tax (GST) exemption exists for basic foods (including fresh fruits and vegetables)(Veerman, 2013).<br><br>**Tonga:** In 2013, as part of a broader package of fiscal measures, import duties were lowered from 20% to 5% for imported fresh, tinned or frozen fish in order to increase affordability and promote healthier diets (World Cancer Research Fund, 2016f).

PRICES2	
**Taxes or levies on unhealthy foods (e.g. sugar-sweetened beverages, foods high in nutrients of concern) are in place and increase the retail prices of these foods by at least 10% to discourage unhealthy food choices, and these taxes are reinvested to improve population health.**	
**International best practice examples (benchmarks)**	**Ireland:** On 1 May 2018, the Republic of Ireland's Sugar Sweetened Drinks Tax came into force under the Finance Act 2017 (No. 41 of 2017). The tax applies to non-alcoholic, water-based and juice based drinks which have added sugar content of 5g per 100mL and above. Drinks with over 8g of sugar per 100mL are taxed at 30 cents per litre, and drinks with between 5g and 8g of sugar per 100mL are taxed at 20 cents per litre. Fruit juices and dairy products are excluded from the tax (World Cancer Research Fund, 2018)<br><br>**UK:** In April 2018 the UK government's Soft Drinks Industry Levy came into force (as outlined in the Finance Act 2017). The Soft Drink Industry Levy applies to any pre-packaged soft drink with added sugar, containing at least 5g of total sugars per 100mL of prepared drink. Soft drinks that have a total sugar content of more than 5g

	and less than 8g per 100mL are taxed 0.18 British pounds ($0.25) per litre and drinks that have a total sugar content of 8g or more per 100mL are taxed 0.24 British pounds ($0.34) per litre. Milk-based drinks, milk substitute drinks, pure fruit juices, or any other drinks with no added sugar, alcohol substitute drinks, and soft drinks of a specified description which are for use for medicinal or other specified purposes are exempt from the levy. The levy applies to soft drinks produced and packaged in the UK and soft drinks imported into the UK (World Cancer Research Fund, 2018). Manufacturers had two years to prepare ahead of this tax coming into effect and over 50% of them took action to cut sugar in their products during that period (Rathbone Greenbank Investments, 2019). It was forecasted that, the tax would bring in £520 million in its first year of operation, but this was revised down to £275 million as a result of company efforts to remove sugar from their products. Data from the first full year of the tax is not yet available, but receipts from April to October 2018 totalled £154 million. It was confirmed that the Department for Education would receive the full £1 billion funding that had originally been expected from the sugar tax in this Parliament (Parliment UK, 2017).<br><br>**Hungary:** A "public health tax" adopted in 2012 is applied on the salt, sugar and caffeine content of various categories of ready-to-eat foods, including soft drinks, energy drinks, and pre-packaged sugar-sweetened products. The tax is applied at varying rates. Soft drinks, for example, are taxed at $0.24 per litre and other sweetened products at $0.47 per litre. The tax also applies to products high in salt, including salty snacks with >1g salt per 100g, condiments with >5g salt per 100g and flavourings >15g salt per 100g (World Cancer Research Fund, 2016f, Biro, 2015).<br><br>**Mexico:** In December 2013, the Mexican legislature passed two new taxes as part of the national strategy for the prevention of overweight, obesity and diabetes. An excise duty of 1 peso ($0.80) per litre applies to sugary drinks. This is expected to increase the price of sugary drinks by around 10%. An ad valorem excise duty of 8% applies to foods with high caloric density, defined as equal to or more than 275 calories per 100 grams. The taxes entered into force on 1 January 2014. The aim is for the revenue of taxes to be reinvested in population health, namely providing safe drinking water in schools, but there is no evidence (yet) that this is the case as the taxes are not earmarked (World Cancer Research Fund, 2016f, Colchero, 2016). In 2019, a study was conducted to estimate changes in taxed and untaxed beverages by volume of beverage purchased after the sugar-sweetened beverage (SSB) tax was introduced in 2014 (Ng et al., 2019). Results found that, The HTLU-unhealthier and HTHU groups had the largest absolute and relative reductions in taxed beverages and increased their purchases of untaxed beverages. Households with lower purchases of untaxed beverages (HTLU unhealthier and LTLU) had the largest absolute and relative increases in untaxed beverages. Furthermore, among households with higher purchases of taxed beverages, the group with lowest socio-economic status had the greatest reduction in purchases of taxed beverages (Ng et al., 2019).

	**Morocco**: On 1 January 2019, Article 5 of the Finance Act 2019 came into effect increasing Morocco's value-added tax on manufactured or imported soft and non-carbonated drinks with added sugars by 50%. Carbonated or non-carbonated water, mineral water, table water or others containing <10% of edible fruit juice or juice concentrates are taxed Moroccan Dirham (MAD) 0.45 (about $0.04) per litre; or those with >10% fruit juice or juice concentrates taxed at MAD 0.15 (about $0.016) per litre. Lemonades containing sugar with <6% lemon juice or concentrate equivalent were taxed MAD 0.45 per litre; or those containing >6% lemon juice or concentrate equivalent taxed at MAD 0.15 per litre. Unfermented carbonated or non- carbonated beverages were taxed MAD 1.24 (about $0.13) per litre. Energy drinks containing at least two stimulant ingredients such as caffeine, taurine and glucuronolactone were taxed MAD 6.00 (about $0.62) per litre (World Cancer Research Fund, 2018).<br><br>**Qatar:** In 2018, the Government of Qatar introduced Law No. (25) the 'Qatar Excise Tax Law' that came into effect on 1 January 2019. The Qatar Excise Tax Law introduced a 50% ad valorem tax on carbonated waters with added sugar, sweeteners or flavours, as well as concentrates, powders, gels or extracts intended to be made into a carbonated beverage. A tax rate of 100% is applied to beverages sold as energy drinks that contain stimulant substances (e.g. caffeine, taurine, ginseng, guarana). Carbonated non-flavoured waters, coffee and tea are excluded from the excise tax. The excise tax applies to all imported, produced or stockpiled aerated beverages (except unflavoured aerated water) and energy drinks (World Cancer Research Fund, 2018).

PRICES3	
**The intent of existing subsidies on foods, including infrastructure funding support (e.g. research and development, supporting markets or transport systems), is to favour healthy rather than unhealthy foods.**	
**International best practice examples (benchmarks)**	**Singapore:** The government, through the Health Promotion Board (HPB) increases the availability and use of healthier ingredients through the "Healthier Ingredient Scheme" (formerly part of the "Healthier Hawker" programme, launched in 2011), which provides in the first instance transitional support to oil manufacturers and importers to help them increase the sale of healthier oils to the food service industry (World Cancer Research Fund, 2016a). The Healthier Ingredient Subsidy Scheme offers a subsidy to suppliers stocking healthier items. Cooking oil is the first ingredient under the scheme, which subsidises oils with a saturated fat level of 35 per cent or lower.

PRICES4	
**The government ensures that food-related income support programs are for healthy foods.**	
**International best practice examples (benchmarks)**	**USA:** In 2009, the U.S. Department of Agriculture's implemented revisions to the Special Supplemental Nutrition Program for Women, Infants, and Children (WIC) to improve the composition and quantities of WIC-provided foods from a health perspective. The revisions include: Increase the dollar amount for purchases of fruits and vegetables, expand whole-grain options, allow for yoghurt as a partial milk substitute, allow parents of older infants to buy fresh produce instead of jarred infant food and give states and local WIC agencies more flexibility in meeting the nutritional and cultural needs of WIC participants (World Cancer Research Fund, 2016f).<br><br>**USA:** In 2012, the USDA piloted a "Healthy Incentives Pilot" as part of the Supplemental Nutrition Assistance Program (SNAP, formerly "food stamps"). Participants received an incentive of 30 cents per US$ spent on targeted fruit and vegetables (transferred back onto their SNAP card). The Pilot included 7500 individuals (World Cancer Research Fund, 2016f). In New York City and Philadelphia, "Health Bucks" are distributed to farmer's markets. When customers use income support (e.g. Food Stamps) to purchase food at farmer's markets, they receive one Health Buck worth 2USD for each 5USD spent, which can then be used to purchase fresh fruit and vegetable products at a farmer's market (World Cancer Research Fund, 2016f). In Philadelphia, the programme has been expanded to other retail settings like supermarkets and corner store.<br><br>**UK:** The British Healthy Start programme provides pregnant women and/or families with children under the age of four with weekly vouchers to spend on foods including milk, plain yoghurt, and fresh and frozen fruit and vegetables. Participants or their family must be receiving income support/jobseekers' allowance or child tax credits. Pregnant women under the age of 18 can also apply. Full national implementation of the programme began in 2006 (World Cancer Research Fund, 2016f).

DOMAIN 5 - FOOD PROVISON

PROV1	
**The government ensures that there are clear, consistent policies (including nutrition standards) implemented in schools and early childhood education services for food service activities (canteens, food at events, fundraising, promotions, vending machines etc.) to provide and promote healthy food choices.**	
**International best practice examples (benchmarks)**	**Ireland:** The School Meals (Local Projects) Scheme, is an administrative scheme, operated directly by the Department of Employment Affairs and Social Protection (Healthy Ireland, 2017). The Scheme provides funding to primary and post-primary schools, local groups, voluntary organisations and community-based not-for-profit preschools operating their own school meals projects. The 'Nutrition Standards for School Meals', are being implemented under this scheme and aim to ensure that children and young people in schools participating in the scheme are provided with healthy balanced meals that follow the Healthy Eating Guidelines. These Nutrition Standards are food-based, and are provided for each meal type funded by the Scheme, that is: Breakfast or snack Lunch or after-school meal Dinner, only healthy food choices that meet the standards will be funded. The Standards will also be used by those administering the Scheme in the schools, commencing in January 2018, to ensure that food purchased complies with the Nutrition Standards when food contracts are being specified in the procurement process, and should also be applied when planning menus (Healthy Ireland, 2017).<br><br>**Jamaica:** In November 2018, the Ministry of Health published mandatory nutrient guidelines for beverages sold/served within all public educational institutions for children (i.e. early childhood, primary level and secondary level). The guidelines prohibit sweetened beverages that exceed a maximum sugar concentration of: 6g/100ml (effective 1 January 2019); 5g/100ml (effective 1 January 2020); 4g/100ml (effective 1 January 2021); and 2.5g/100ml (effective 1 January 2023). All unsweetened beverages are permitted The guidelines also caution against beverages containing >10mg/serve of caffeine, discourage the use of artificial sweeteners and recommend beverage portions sold/served of <12 ounces (not including water).<br><br>**Chile:** In 2012, the Chilean government approved a Law of Nutritional Composition of Food and Advertising [51]. In June 2015, the Chilean authority approved the regulatory norms required for the law's implementation. The regulatory norms define limits for calories, saturated fat, sugar and sodium content considered 'high' in foods and beverages. The law prohibits the sale of foods in the 'high in' category in schools. The law came into effect on 27 June, 2016. In 2019, a study conducted on the impact of this law funds that, foods exceeding any cut-offs decreased from 90.4% in 2014 to 15.0% in 2016. Solid products had a substantial reduction in calories, sugar, saturated fat, and sodium. Liquid products had a reduction in calories, total sugar, and saturated fat, whereas sodium increased. This was a result of changes in product mix (Massri et al., 2019).

	**Finland**: In 2008, the National Nutrition Council approved nutrition recommendations for school meals. These include food and nutrient recommendations for salt, fibre, fat, starch, fat and salt maximums for meat and processed meat, and drinks. There are also criteria for snacks provided in schools. New recommendations on Eating and learning together - recommendations for school meals have been published in 2017 (National Nutrition Council, 2017). In 2018, the early childhood education: Health and joy from food - meal recommendations for early childhood education and care, were published (National Nutrition Council, 2018). The 2018 published recommendations for families with children: Eating together - food recommendations for families with children, were updated in 2019 (National Institute for Health and Welfare, 2019). Additionally, Finland published its first nutrition recommendations for upper secondary schools and vocational schools.<br><br>**UK:** England, Scotland, Wales and Northern Ireland have mandatory nutritional standards for school food, which also apply to food provided in schools other than school lunches. These standards apply to most state schools (with the exception of around 4,000 academies established between September 2010 and June 2014, which are exempt) and restrict foods high in fat, salt and sugar, as well as low quality reformed or reconstituted foods .<br><br>**Brazil:** The national school feeding programme mandates a weekly minimum of fruits and vegetables, regulates sodium content and restricts the availability of sweets in school meals. A school food procurement law, approved in 2001, limits the amount of processed foods purchased by schools to 30%, and bans the procurement of drinks with low nutritional value, such as sugary drinks. The law requires schools to buy locally grown or manufactured products, supporting small farmers and stimulating the local economy. Resolution no 38 (16 July 2009) sets food- and nutrition-based standards for the foods available in the national school meal programme (Law 11.947/2009). Article 17 prohibits drinks of low nutritional value (e.g. soda), canned meats, confectionary and processed foods with a sodium and saturated fat content higher than a specified threshold.<br><br>**Costa Rica:** Executive Decree No 36910-MEP-S (2012) of the Costa Rican Ministries of Health and Education sets restrictions on products sold to students in elementary and high schools, including food with high levels of fats, sugars and salt, such as chips, cookies, candy and carbonated sodas. Schools are only permitted to sell food and beverages that meet specific nutritional criteria. The restrictions were upheld by the Constitutional Court in 2012 following a challenge by the food industry.<br><br>**Hungary**: Since 2012, food and beverages subject to the public health product tax may not be sold on school premises or at events organized for school children, including out of school events based on the Ministerial Decree 20/2012 (VIII.31) on the Operation of Public Education Institutions and the of Names of Public Education Institutions. Section 130(2) of the Decree requires the head of the educational institution to consult the school health service prior to entering into agreements with

	vending machine operators or food vending businesses. The school health service verifies whether the products to be sold meet the nutritional guidelines set by the National Institute of Pharmacy and Nutrition. Products that do not comply are prohibited.<br><br>**Uruguay**: In September 2013, the government of Uruguay adopted Law No 19.140 on 'healthy eating in schools. It mandated the Ministry of Health to develop standards for food available in canteens and kiosks in schools, prohibited advertising for these same foods and restricted the availability of saltshakers. The school food standards were elaborated in March 2014 and aimed to promote foods with natural nutritional value with a minimum degree of processing and to limit the intake of free sugars, saturated fat, trans fat and sodium. Limits are set per 100g of food, 100ml for drinks and also per 50g portion. This was implemented in public schools in 2015 (World Cancer Research Fund, 2016d, Fundo Nacional de Desinvolvimento da Educacao, 2016). This was implemented in public schools in 2015.

**PROV2**	
**The government ensures that there are clear, consistent policies in other public sector settings for food service activities (canteens, food at events, fundraising, promotions, vending machines, public procurement standards etc.) to provide and promote healthy food choices**.	
**International best practice examples (benchmarks)**	**Latvia:** In 2012, the government set salt levels for all foods served in hospitals and long-term social care institutions. Levels may not exceed 1.25g of salt/100g; fish products may contain up to 1.5g salt/100g.<br><br>**Ireland:** The HSE Vending Policy 2019 replaces the 2014 Healthier Vending Policy and applies to all vending machines that stock cold soft drinks, confectionery and snacks on HSE premises & premises funded by the HSE. Sugar sweetened beverages will not be stocked in vending machines, 50% of beverages stocked will be still water and the remaining beverages stocked will include non-sugar sweetened beverages e.g. diet drinks, juices, flavoured and sparkling water. Snacks containing more than 200 calories per packet will not be stocked in machines. An exception to this is 3 packets of dried fruits, nuts or seeds (plain and unsalted). Products will be clearly labelled with the number of calories per product related fields.<br><br>**Bermuda:** In 2008, the Government Vending Machine Policy was implemented in government offices and facilities to ensure access to healthy snacks and beverages for staff. The policy requires that all food and beverages in vending machines on government premises meet specific criteria based on levels of total fat, saturated fat, trans fat, sodium and sugar. Criteria exclude nuts and 100% fruit juices.<br><br>**New York**: New York City's Food Standards (enacted with Executive Order 122 of

	2008) set nutritional standards for all food purchased or served by city agencies, which applies to prisons, hospitals and senior care centres. The Standards include: maximum and minimum levels of nutrients per serving; standards on specific food items (e.g. only no-fat or 1% fat milk); portion size requirements; the requirement that water be offered with food; a prohibition on the deep-frying of foods; and daily calorie and nutrient targets, including population-specific guidelines (e.g. children, seniors) (World Cancer Research Fund, 2016d, Lederer, 2014). As of 2015, 11 city agencies are subject to the NYC Food Standards, serving and selling almost 250 million meals a year. The Food Policy Coordinator has the responsibility of ensuring adherence with the Food Standards. Self-reported compliance with the standard is 96%.<br><br>**Wales:** Vending machines are prohibited in National Health Service Hospitals. The government issued a guidance defining what is allowed and not and has liaised with major vending providers to find ways to introduce healthier food options (Health Promoting Hospital Vending Directions and Guide 2008).<br><br>**The Netherlands:** The Netherlands Nutrition Centre introduced the 'Guidelines for Healthier Canteens', which can be applied in canteens at schools, sports clubs and workplaces to make them more healthy. The Guidelines for Healthier Canteens cover canteens at product level and at the level of the full range of food and drink being offered, together with the canteen's general display layout. The framework of the Guidelines for Healthier Canteens defines three different levels: bronze, silver and gold (Netherlands Nutrition Centre, 2017).

PROV3	
**The government ensures that there are clear, consistent public procurement standards in public sector settings for food service activities to provide and promote healthy food choices.**	
**International best practice examples (benchmarks)**	**Brazil:** A school food procurement law , approved in 2001, limits the amount of processed foods purchased by schools to 30%, and bans the procurement of drinks with low nutritional value, such as sugary drinks. The law requires schools to buy locally grown or manufactured products, supporting small farmers and stimulating the local economy<br><br>**UK:** The UK Government Buying Standard for Food and Catering Services (GBSF of 2014, updated March 2015, sets out standards for the public sector when buying food and catering services. It is supported by the Plan for Public Procurement: Food and Catering Services (2014). The nutrition requirements must be followed by schools, hospitals, care homes, communities and the armed forces. To improve diets, the GBSF sets maximum levels for sugar in cereals and generally for saturated fat and salt, in addition to minimum content of fibre in cereals and fruit in desserts. (World Cancer Research Fund, 2016e).
**PROV4**	

The Government ensures that there are good support and training systems to help schools and other public sector organisations and their caterers meet the healthy food service policies and guidelines	
**International best practice examples (benchmarks)**	**Australia:** The Healthy Eating Advisory Service supports settings such as childcare centres, schools, workplaces, health services, food outlets, parks and sporting centres to provide healthy foods and drinks to the public in line with Victorian Government policies and guidelines. The Healthy Eating Advisory Service is delivered by experienced nutritionists and dieticians at Nutrition Australia Victorian Division. The support includes training cooks, chefs, foods service and other key staff, discovering healthier recipes, food ideas and other helpful resources to provide healthier menus and products.<br><br>**Singapore:** The National Workplace Health Promotion Programme, launched in Singapore in 2000, is run by the Health Promotion Board. Both private and public institutions are encouraged to improve the workplace environment by providing tools and grants. Grants are awarded to help companies start and sustain health promotion programmes. Tools include a sample Healthy Workplace Nutrition Policy, a sample Healthy Workplace Catering Policy, and a detailed Essential Guide to Workplace Health, setting out ways to transform the workplace into a health-supporting work environment.<br><br>**Netherlands:** The Healthy School Canteen Brigade (https://gezondeschoolkantine.voedingscentrum.nl/nl.aspx) is a team consisting of dieticians and health scientists of the Dutch Nutrition Center to help school realize healthy canteens. They visit schools in the Netherlands and give them advice. The Dutch Nutrition Center also developed the canteen scan (https://gezondeschoolkantine.voedingscentrum.nl/nl/stap-voor-stap/kantinescan.aspx), a tool to check the level of healthiness of canteens and which gives practical advises.

PROV5	
**The Government actively encourages and supports private companies to provide and promote healthy foods and meals in their workplaces**	
**International best practice examples (benchmarks)**	**Ireland:** A Healthy Workplaces Framework has been developed as part of the governments public health Framework, Healthy Ireland - A Framework for improved health and wellbeing: 2013 – 2025, by the Department of Health and the Department of Business, Enterprise and Innovation (Department of Health, 2013) (McAvoy, 2018). It was developed following a public consultation with interested stakeholders and aims to enhance existing initiatives to, facilitate the sharing of experience and learning, and also provide the necessary supports and tools for organisations or companies who have not yet developed their own resources. The Healthy Workplaces Framework is due to be implemented in 2020.

DOMAIN 6 - FOOD IN RETAIL

RETAIL1	
**Zoning laws and policies are implemented to place limits on the density or placement of quick serve restaurants or other outlets selling mainly unhealthy foods in communities and/or access to these outlets (e.g. opening hours).**	
**International best practice examples (benchmarks)**	**South Korea**: In 2010 the Special Act on Children's Dietary Life Safety Management established the creation of 'Green Food Zones' around schools, banning the sale of foods (fast food and soda) deemed unhealthy by the Food and Drug Administration of Korea within 200 metres of schools In 2016, Green Food Zones existed at over 10000 schools.<br><br>**UK:** Around 15 local authorities have developed "supplementary planning documents" on the development of hot food takeaways. The policies typically exclude hot food takeaways from a 400m zone around the target location. All policies include secondary schools, some policies also include primary schools, parks and youth centres(World Cancer Research Fund, 2016).<br><br>**Detroit USA:** In Detroit, the zoning code prohibits the building of fast food restaurants within 500 ft. of all elementary, junior and senior high schools (World Cancer Research Fund, 2016).

RETAIL2	
**Zoning laws and policies are implemented to encourage the availability of outlets selling fresh fruit and vegetables and/or access to these outlets (e.g. opening hours, frequency i.e. for markets).**	
**International best practice examples (benchmarks)**	**USA:** February 2014 the US Congress formally established the Healthy Food Financing Initiative (following a three-year pilot) which provides grants to states to provide financial and/or other types of assistance to attract healthier retail outlets to underserved areas. The pilot distributed over 140 million USD in grants to states to provide financial and other types of assistance to attract healthier retail outlets in underserved areas. To date, 23 US states have implemented financing initiatives (World Cancer Research Fund, 2016).<br><br>**New York City (USA):** The 'Green Cart Permit' was developed with reduced restrictions on zoning requirements to increase the availability of fresh fruits and vegetables in designated, underserved neighbourhoods (World Cancer Research Fund, 2016). In 2008 New York City made 1000 licenses for green carts available to street vendors who exclusively sell fresh fruit and vegetables in neighbourhoods with limited access to healthy foods (World Cancer Research Fund, 2016). In addition, in 2009, New York City established the food retail expansion to support

	the health program of New York City (FRESH). Under the programme, financial and zoning incentives are offered to promote neighbourhood grocery stores offering fresh meat, fruit and vegetables in under-served communities. The financial benefits consist of an exemption or reduction of certain taxes. The zoning incentives consist of providing additional floor area in mixed buildings, reducing the amount of required parking, and permitting larger grocery stores in light manufacturing districts.

**RETAIL3**	
**The Government ensures existing support systems are in place to encourage food stores to promote the in-store availability of healthy foods and to limit the in-store availability of unhealthy foods**	
**International best practice examples (benchmarks)**	**USA:** The Special Supplemental Nutrition Program for Women, Infants, and Children (WIC) requires WIC authorised stores to stock certain healthier products (e.g. wholegrain bread) (World Cancer Research Fund, 2016f).<br><br>**The Netherlands:** The National Action plan for vegetables and Fruit is a cooperation of government, industry and civil society organisations. The Goal is to increase the consumption of vegetables and fruits in 3 years (2018-2020) by linking and strengthening existing initiatives**.** The National Action Plan vegetables and fruit stimulates consumers to eat more vegetables and fruit using the motto 'Go for Colour'. As part of 'Go for Colour' an in-store experiment has taken place promoting the in-store availability of vegetables and fruit.

**RETAIL 4**	
**The government ensures existing support systems are in place to encourage the promotion and availability of healthy foods in food service outlets and to discourage the promotion and availability of unhealthy foods in food service outlets**	
**International best practice examples (benchmarks)**	**USA:** In December 2011, San Francisco implemented the Health Food Incentives Ordinance which bans restaurants, including takeaway restaurants, to give away toys and other free incentive items with children's meals unless the meals meet nutritional standards as set out in the Ordinance: meals must not contain more than 600 calories and include a min amount of fruits and vegetables. It also applies to drinks with excessive calories, fat, excessive sugars ,added non-nutritive sweeteners or caffeine (World Cancer Research Fund, 2016e).<br><br>**France:** Since January 2017 France has banned unlimited offers of sweetened beverages for free or at a fixed price in public restaurants and other facilities accommodating or receiving children under the age of 18. Sweetened beverages are defined as any drink sweetened with sugar or artificial (caloric and non-caloric) sweeteners, including flavoured carbonated and still beverages, fruit syrups, sport and energy drinks, fruit and vegetable nectars, fruit- and vegetable-based drinks,

	as well as water- milk- or cereal based beverages (World Cancer Research Fund, 2016).<br><br>**Los Angles, USA:** In September 2013, the Los Angeles County Department of Public Health launched Choose Health LA Restaurants in partnership with local restaurants to promote healthier meal choices. Restaurants must apply to become a partner. Participating restaurants offer customers smaller portion size options (in addition to existing items on the menu), healthier meals for children that include vegetables and fruit, healthy beverages, non-fried food and free chilled water. Participating restaurants are recognised as Public Health partners in promoting healthier communities.

DOMAIN 8 – LEADERSHIP

**LEAD1**	
**There is strong, visible, political support (at the head of government or state/ ministerial level) for improving food environments, population nutrition, diet related NCDs and their related inequalities"**	
**International best practice examples (benchmarks)**	**New York City (USA):** As Mayor of New York City, Michael Bloomberg prioritised food policy and introduced a number of ground breaking policy initiatives including 'Health Bucks', a restriction on trans fats, establishment of an obesity taskforce, a portion size restriction on sugar-sweetened beverages, public awareness campaigns, etc. He showed strong and consistent leadership and a commitment to innovative approaches and cross-sectoral collaboration.<br><br>**Brazil:** The Minister of Health showed leadership in developing new dietary guidelines that are drastically different from the majority of dietary guidelines created by any nation to date, and align with some of the most commonly cited recommendations for healthy eating .<br><br>**Some Caribbean Countries:** Active NCD commissions exist in six of the 20 CARICOM member states (Bahamas, Barbados, Bermuda, British Virgin Islands, Dominica, Grenada) which are all housed in their Ministries of Health, with members recommended by the Minister of Health and appointed by the Cabinet of Government for a fixed duration; all include government agencies and to a varying degree, civil society and the private sector<br><br>**Ireland:** Healthy Ireland "A Framework for Improved Health and Wellbeing 2013-2025", was launched in 2013 and aims to increase the proportion of people who are healthy at all stages of life, reduce health inequalities, protect the public from threats to health and wellbeing and create an environment where every individual and sector of society can play their part in achieving a healthy Ireland. In 2016, the

	Government approved the creation of a Healthy Ireland Fund with an initial allocation of €5 million approved in Budget 2017 to establish and support the implementation of Healthy Ireland programmes and projects in a variety of settings. The primary aim of the fund is to support innovative, cross-sectoral, evidence-based projects and initiatives that support the implementation of key national policies in areas such as obesity, smoking, alcohol, physical activity and sexual health(Pobal, 2016). The Department of Health has approved a third round of funding, which aims to support local and national organisations to deliver actions that will improve health and wellbeing in line with Healthy Ireland, A Framework for Improved Health and Wellbeing 2013-2025 (Department of Health, 2019a).<br><br>**The Netherlands:** In 2018, the Ministry of Health, together with more than 70 organizations signed the National Prevention Agreement. It aims to reduce smoking, overweight and problematic alcohol consumption. The agreement includes voluntary ambitions, objectives and actions on these three subjects for the period (2018-2040). The National prevention agreement acknowledges that peoples' contexts is important, and that, among other factors, a healthy environment is needed for those that need it in order to prevent overweight, obesity and NCD's. The agreement formulates that inhabitants of the Netherlands need a healthy social, economic and physical environment, that supports healthy living, including schools, care facilities, restaurants, cafes, caterers and supermarkets. Specific voluntary targets with respect to the food environment are:<br><br>• In 2020, 2,500 sports clubs will be working on providing a healthier range of food products in their sports canteens.<br>• No later than 2025, 50% of hospitals will offer a healthy diet; no later than 2030, all of them will.<br>• In 2020, there will be 950 healthy school canteens. This means that 50% of all school canteens will be healthy.<br>• Businesses will develop healthier products (e.g. reformulation by limiting sugar content)<br>• The central government wants to introduce, no later than 2020, a new, broadly supported food-choice logo based on thorough, independent consumer research.<br><br>An example of a voluntary actions formulated is: Supermarkets will entice consumers to buy more products from the Wheel of Five, among other things by telling them which products are suitable (Netherlands, 2019).

LEAD2	
**Clear population intake targets have been established by the government for the nutrients of concern and / or relevant food groups to meet WHO and national recommended dietary intake levels**	
**International best practice examples (benchmarks)**	**Brazil:** The "Strategic Action Plan for Confronting NCDs in Brazil, 2011-2022 specifies a target of increasing adequate consumption of fruits and vegetables, from 18.2% to 24.3 % between 2010 and 2022 and reduction of the average salt intake of 12 g to 5 g, between 2010 and 2022 .<br><br>**South Africa:** The South African plan for the prevention and control of non-communicable diseases includes a target on reducing mean population intake of salt to <5 grams per day by 2020.<br><br>**UK:** In August 2016, government set out its approach to reduce the prevalence of childhood obesity in 'Childhood obesity: a plan for action'. A key commitment in the plan was to launch a broad, structured sugar reduction programme to remove sugar from everyday products. All sectors of the food and drinks industry are challenged to reduce overall sugar across a range of products that contribute most to children's sugar intakes by at least 20% by 2020, including a 5% reduction in the first year of the programme (Public Health England, 2017).<br><br>**The Netherlands:** On January 2014, the Dutch Ministry for Health, Welfare and Sport, signed an agreement with trade organisations representing food manufactures, supermarkets, hotels, restaurants, caterers and the hospitality industry. The agreement included intake targets for example; a maximum of 6 grams of salt consumption per day in 2020 and consuming a maximum of 10% energy from saturated fat per day in 2020 (The Central Government for the Netherlands, 2014).<br><br>**Norway:** The National Action Plan for a Better Diet (2017-2021) contains quantitative intake targets for nutrient of concern and specific food groups in the population. By 2021, the plan sets out a reduction of the following nutrients: Added sugar from 13 to 11E%; saturated fat from 14 to 12E%; and a 22% reduction in salt intake from 10 g/day. There are specific targets to halve the proportion of youth that consumes sugar-sweetened beverages or sweets more than 5 times per week; to double the proportion of youth that eats fruit and vegetables daily; and to increase by 20% the proportion of youth that eats fish at least once a week. There are also targets to increase the intake of fruit, vegetables, whole grain products and fish with 20% in the general population (Røynstrand, 2017).

LEAD3	
**Clear, interpretive, evidenced-informed food based dietary guidelines have been established and implemented.**	
**International best practice examples (benchmarks)**	**Brazil:** The national dietary guidelines of Brazil address healthy eating from a cultural, ethical and environmental perspective, rather than based on number of servings per food group. The main recommendations are: 'Make natural or minimally processed foods the basis of your diet'; 'use oils, fats, salt, and sugar in small amounts for seasoning and cooking foods'; 'use processed foods in small amounts'; 'avoid ultra-processed foods'. They also provide advice on planning, shopping and sharing meals, as well as warning people to be wary of food marketing and advertising.<br><br>**Ireland:** A Healthy Weight for Ireland, Obesity Policy and Action Plan - 2016–2025 called for the development of a suite of Healthy Eating Guidelines for the general population. These revised Healthy Eating Guidelines and Food Pyramid resources, are an early action under the Plan (Action 5.3.) (Department of Health, 2016a). The revised Healthy Eating Guidelines and Food Pyramid are based on the FSAI Scientific Recommendations for Healthy Eating Guidelines in Ireland from 2011 (Food Safety Authority of Ireland, 2011). They describe how to build a healthy diet, for different age groups (from 5 years of age), depending also on gender and activity levels. The Guidelines, Food Pyramid and supporting resources have been published, disseminated and communicated in 2017, including dissemination of the new Guidelines to all primary and post-primary schools. The revised Healthy Eating Guidelines and Food Pyramid toolkit has been developed by the Department of Health and the Health Service Executive with key stakeholders and aims to help reduce the intake of high fat, salt and sugar (HFSS) foods and drinks from the Top Shelf of the Food Pyramid (Healthy Ireland, 2016)<br><br>**The Netherlands:** The Dutch Health Council published the 'Guidelines Good Food' 2015. These guidelines advise to eat more plant-based and less animal-based food and include advice on the intake of different food products. The Dutch Nutrition Center published the 'Wheel of Five' Guidelines, based on the 'Guidelines Good Food' of the Dutch Health Council. The 'Wheel of Five' includes advice on the ingredients of a healthy diet, making a distinction between five sections: (1) Vegetables and fruit (2) spread and cooking fats (3) Fish, legumes, meat, eggs, nuts and dairy products (4) Bread, cereal products and potatoes and (5) Drinks (Health Council of the Netherlands, 2015)

LEAD4	
**There is a comprehensive, transparent, up-to-date implementation plan linked to national needs and priorities, to improve food environments , reduce the intake of the nutrients of concern to meet WHO and national recommended dietary intake levels, and reduce diet-related NCDs**	
**International best practice examples (benchmarks)**	**Ireland:** A Healthy Weight for Ireland', the Obesity Policy and Action Plan 2016-2025 (OPAP), was launched in September 2016 under the auspices of the Healthy Ireland agenda. The OPAP covers a ten-year period up to 2025, which prescribed 'Ten Steps Forward' that would be taken to reverse obesity trends, prevent health complications and reduce the overall burden for individuals, families, the health system, and the wider society and economy (Department of Health, 2016a). A new Obesity Policy Implementation Oversight Group (OPIOG) was established in October 2017 and a progress report on each recommendation in the OPAP is currently being finalised under the aegis of the OPIOG (Oireachtas, 2019). As set out in Healthy Ireland, integrated health and social impact assessments will be conducted on relevant policy areas to support other government departments in contributing towards the prevention of overweight and obesity.

LEAD5	
**Government priorities have been established to reduce inequalities or protect vulnerable populations in relation to diet, nutrition, obesity and NCDs**	
**International best practice examples (benchmarks)**	**New Zealand:** The Ministry of Health reports the estimates derived from health surveys and nutrition surveys by four subpopulation groups including age group, gender, ethnic group and an area level deprivation index. Similarly, estimates derived from other data types (e.g. mortality) are presented by these subpopulation groups. The contracts between MoH and NGOs or other institutions include a section on Maori Health and state: "*An overarching aim of the health and disability sector is the improvement of Maori health outcomes and the reduction of Maori health inequalities".* In the specific contract between the Ministry of Health and Agencies for Nutrition Action the first clause is on Maori Health relating to compliance with any Maori specific service requirements, quality requirements and specific monitoring requirements contained in the Service specifications to this agreement.<br><br>**Australia:** The National Indigenous Reform Agreement (Closing the Gap) is an agreement between the Commonwealth of Australia and the States and Territories. The objective of this agreement is to work together with Indigenous Australians to Close the Gap in Indigenous disadvantage. The targets agreed to by COAG relate to health or social determinants of health. For the target 'Closing the life expectancy gap within a generation (by 2031)', one of the performance indicators is the prevalence of overweight and obesity.

	**Ireland**: **Step 9** of the Healthy Weight for Ireland: Obesity Policy and Action Plan 2016-2025 aims to, allocate resources to those population groups most in need of support in the prevention and management of obesity, with particular emphasis on families and children during the first 1,000 days of life. The priority actions to commence in first year were to, assess the needs of vulnerable groups as the basis of allocation of resources for preventative and treatment services for children and adults (Department of Health, 2016b). The Healthy Ireland fund was established in 2017 with an allocation of €5 million and with additional allocations of €5 million in 2018 and 2019. The first round of the Fund was distributed through Local Community Development Committees, Children and Young Person's Services Committees and statutory organisations. The Fund has been effective at targeting population groups that experience health inequalities. In Round 1 (2017/18), there was a focus on specific groups experiencing health inequalities, including people living in areas of social disadvantage (71% of actions), people with disabilities (45%), people from new communities including refugees and asylum seekers (39%) and members of the Traveller community (36%). Furthermore, of the local actions that were implemented in Round 1, 61% related to physical activity and 32% were related to food, nutrition and weight management. Round 2 of the Fund is currently being implemented (Oireachtas, 2019) The Healthy Ireland 2019 communications and citizen engagement campaign has continued on from 2018, launching on the 8th of April 2019, with an announced funding of €1 million to boost community engagement on health and wellbeing in every county (Department of Health, 2019b).

DOMAIN 9 - GOVERNANCE

**GOVER1**	
**There are procedures in place to restrict commercial influences on the development of policies related to food environments where they have conflicts of interest with improving population nutrition. For example: restricting lobbying influences.**	
**International best practice examples (benchmarks)**	**USA:** Mandatory and publicly accessible lobby registers exist at the federal level, as well as in nearly every state. Financial information must be disclosed, and the register is enforced through significant sanctions. A number of pieces of legislation uphold compliance with the register including Lobbying Disclosure Act of 1995 and the Honest Leadership and Open Government Act 2007.<br><br>**New Zealand:** The State Services Commission has published Best Practice Guidelines for Departments Responsible for Regulatory Processes with Significant Commercial Implications. They cover the development and operation of a regulatory process and include specific references to principles around stakeholder relationship management.

	**Australia:** The Australian Public Service Commission's Values and Code of Conduct includes a number of relevant sections such as the Conflict of Interest, Working with the Private Sector and other Stakeholders and the Lobbying Code of Conduct.<br><br>**Ireland:** The Regulation of Lobbying Act 2015 was signed into law in March 2015. The purpose of the Act is to, provide for a web-based Register of Lobbying to make information available to the public on the identity of those communicating with designated public officials on specific policy, legislative matters or prospective decisions. In support of the Act's objectives to foster transparency and the proper conduct of lobbying activities, the Code of Conduct for persons carrying on lobbying activities was established. Its purpose is to govern the behaviour of persons carrying on lobbying activities. The provisions of the Act can apply to employers; to representative or advocacy bodies; to professional lobbyists or third parties who are being paid to communicate on behalf of a client or other person; and, significantly, to any person communicating about the development or zoning of land (Standards in Public Office Commission, 2019).

**GOVER2**	
**Policies and procedures are implemented for using evidence in the development of food and nutrition policies**	
**International best practice examples (benchmarks)**	**Australia:** The National Health and Medical Research Council Act 1992 (NHMRC Act) requires NHMRC to develop evidence-based guidelines. These national guidelines are developed by teams of specialists following a rigorous nine-step development process.

GOVER3	
**Policies and procedures are implemented for ensuring transparency in the development of food and nutrition policies**	
**International best practice examples (benchmarks)**	**New Zealand:** Food Standards Australia New Zealand (FSANZ) is required by the Food Standards Australia New Zealand Act 1991 to engage stakeholders in the development of new standards. This process is open to everyone in the community including consumers, public health professionals, and industry and government representatives. FSANZ has developed a Stakeholder Engagement Strategy 2013-16 that outlines the scope and processes for engagement. Under the Stakeholder Engagement Priorities 2013-16, it outlined "maintain our open and transparent approach" as one of the first priorities.<br><br>**Norway:** The Public Administration Act provides general procedural rules for public administration. The Act regulates the administrative procedures when decisions are made, especially the rights of parties during the procedures. The Central Government Communication Policy contains the central goals and principles of the central government's communication with citizens, businesses, organizations and other public sector activities. The goals of the central government communication policy state that the citizens shall: receive accurate and clear information about their rights, duties and opportunities; have access to information about central government activities; and be invited to participate in the formulation of policy, schemes and services (Norwegian Ministry of Local Government and Modernisation, 2019).

GOVER4	
**The government ensures public access to comprehensive nutrition information and key documents (e.g. budget documents, annual performance reviews and health indicators) for the public**	
**International best practice examples (benchmarks)**	**New Zealand/Australia:** The freedom of information Act provides a legally enforceable right of the public to assess documents of government departments and most agencies.<br><br>**Ireland:** The Freedom of Information Act 2014 came into effect in October 2014 and repealed the 1997 and 2003 Acts. The 2014 Act now applies to all public bodies, unless they are specifically exempt. It also allows for the Government to prescribe (or designate) other bodies receiving significant public funds, so that the FOI legislation applies to them also. The old legislation continues to apply to any FOI request that was made before the 2014 Act came into effect. It also applies to any subsequent review or appeal. It provides the following statutory rights, (1) A legal right for each person to access information held by a body to which FOI legislation applies known as an *FOI body.* (2) A legal right for each person to have official information relating to himself/herself amended where it is incomplete, incorrect or misleading. (3) A legal right for each person to obtain reasons for decisions affecting himself/herself.

	**Norway:** The Freedom of Information Act grants everyone the right of access to case documents, journals and similar registers for any agencies encompassed by the Act. The Act applies to all government agencies, municipalities and county authorities. The general rule is that access shall be granted, and exceptions to this rule require legal authority prescribed by or pursuant to law. The Act also contains rules for handling right of access claims and the opportunity to appeal decisions in access matters (Norwegian Ministry of Local Government and Modernisation, 2019).

DOMAIN 10 - MONITORING AND INTELLIGENCE

**MONIT1**	
**Monitoring systems, implemented by the government, are in place to regularly monitor food environments(especially for food composition for nutrients of concern, food promotion to children, and nutritional quality of food in schools and other public sector settings), against codes / guidelines / standards / targets**	
**International best practice examples (benchmarks)**	**Many countries:** have food composition databases available. For example, the New Zealand Institute for Plant & Food Research Limited and the Ministry of Health jointly own the New Zealand Food Composition Database (NZFCD) which is a comprehensive collection of nutrient data in New Zealand containing nutrient information on more than 2600 foods.<br><br>**New Zealand:** A national School and Early Childhood Education Services (ECES) Food and Nutrition Environment Survey was organised in all Schools and ECES across New Zealand in 2007 and 2009 by the Ministry of Health to measure the food environments in schools and ECEs in New Zealand.<br><br>**UK:** In October 2005, the School Food Trust ('the Trust'; now called the Children's Food Trust) was established to provide independent support and advice to schools, caterers, manufacturers and others on improving the standard of school meals. They perform annual surveys, including the latest information on how many children are having school meals in England, how much they cost and how they're being provided.<br><br>**Germany:** The German Nutrition Report published by the DGE on behalf of the Federal Republic of Germany. The Nutrition Reports for the Federal Republic of Germany have been published by the DGE since 1969. Since 1972 it is provided every 4 years by directive of the Federal Ministry of Consumer Protection, Food and Agriculture. The subjects follow topics of current interest (German Nutrition Society). The Robert-Koch-Institute (RKI), the government's scientific institution in the field of public health, started in 2015 the project 'AdiMon' that collects regular data on influencing (environmental) factors and prevalence of childhood obesity, as well as interventions for prevention and health promotion (Robert Koch

	Institut).<br><br>**The Netherlands:** the progress in product improvement of salt, saturated fat and calories (sugar and/or (saturated) fat) is monitored by the Dutch Institute of Public Health and Environment (Rijksinstituut voor Volksgezondheid en Milieu (RIVM)) at product level. RIVM uses the product databank (levensmiddelendatabank (LEDA) as basis for which companies have to provide information about product contents (Ministry of Public Health).

**MONIT2**	
**There is regular monitoring of adult and childhood nutrition status and population intakes against specified intake targets or recommended daily intake levels**	
**International best practice examples (benchmarks)**	**USA:** The National Health and Nutrition Examination Survey (NHANES) is a program of studies designed to assess the health and nutritional status of adults and children in the United States. The survey is unique in that it combines interviews and physical examinations (Centres for Disease Control, 2016)(Centres for Disease Control, 2016). The NHANES program began in the early 1960s and has been conducted as a series of surveys focusing on different population groups or health topics. In 1999, the survey became a continuous program that has a changing focus on a variety of health and nutrition measurements to meet emerging needs. The survey examines a nationally representative sample of about 5,000 persons each year. These persons are located in counties across the country, 15 of which are visited each year.<br><br>**The Netherlands:** The Dutch Institute of Public Health and Environment (Rijksinstituut voor Volksgezondheid en Milieu (RIVM)) is appointed by the Ministry of Health to periodically collect data about the food consumption and food condition of the Dutch population in general and of separate population groups via the Food Consumption Survey. Currently, a Food Consumption Survey (Dutch population 1-79 years) is being conducted for the years 2019-2021. Prior Food Consumption Surveys have been conducted for the years 2012-2016 (Dutch population 1-79 years), 2010-2012 (elderly 70+), 2007-2010 (7-69 years), 2005-2006 (2-6 years), 2003 (9-16 years)

MONIT3	
**There is regular monitoring of adult and childhood overweight and obesity prevalence using anthropometric measurements**	
**International best practice examples (benchmarks)**	**UK:** England's National Child Measurement Programme was established in 2006 and aims to measure all children in England in the first (4-5) years and last (10-11 years) of primary school. In 2011-2012, 565 662 children (4-5 years) and 491118 children (10-11 years) were measured.<br><br>**Ireland:** The 4th Childhood Obesity Surveillance Initiative report was launched in 2017. COSI collects data from children in primary schools in the Republic of Ireland. The survey is carried out periodically. Data was first collected from children in 2008 in first class and again in 2010 from first class and third class, in 2012 from first, third and fifth classes and in 2015 from first, fourth and sixth class. Trained researchers collected weight, height and waist circumference measurements. These figures were used to examine prevalence of normal weight, overweight, obesity and mean BMI.

MONIT4	
**There is regular monitoring of the prevalence of NCD metabolic risk factors and occurrence rates (e.g. prevalence, incidence, mortality) for the main diet-related NCDs**	
**International best practice examples (benchmarks)**	

MONIT5	
**Major programs and policies are regularly evaluated to assess their effectiveness and contributions to achieving the goals of the nutrition and health plans**	
**International best practice examples (benchmarks)**	**USA:** The National Institutes for Health (NIH) provide funding for rapid assessments of natural experiments. The funding establishes an accelerated review/award process to support time-sensitive research to evaluate a new policy or program expected to influence obesity related behaviours (e.g., dietary intake, physical activity, or sedentary behaviour) and/or weight outcomes in an effort to prevent or reduce obesity (US National Institutes of Health, 2016).<br><br>**The Netherlands:** The Dutch Institute of Public Health and Environment (Rijksinstituut voor Volksgezondheid en Milieu (RIVM)) conducted in 2017 a midterm evaluation to calculate the effect of the agreed maximum norms for salt

	and sugar in the Agreement on Product Improvement (The Dutch Insititute of Public Health and Environment 2017). A midterm evaluation has been performed to calculate the effect of the agreed maximum norms for salt and sugar reduction, and four scenarios have been calculated with the Food Consumption Survey.<br><br>**Ireland:** Under the 'Healthy Weight for Ireland', Obesity Policy and Action Plan 2016-2025, a new Obesity Policy Implementation Oversight Group (OPIOG) was established in October 2017 and a progress report on each recommendation in the OPAP is currently being finalised under the aegis of the OPIOG (Oireachtas, 2019). As set out in Healthy Ireland, integrated health and social impact assessments will be conducted on relevant policy areas to support other government departments in contributing towards the prevention of overweight and obesity.

**MONIT6**	
**Progress towards reducing health inequalities or health impacts in vulnerable populations and social and economic determinants of health are regularly monitored.**	
**International best practice examples (benchmarks)**	**New Zealand:** All annual Ministry of Health Surveys report estimates by subpopulations in particular by ethnicity (including Maori and Pacific peoples), by age, by gender, and by New Zealand area deprivation.

DOMAIN 11 - FUNDING AND RESOURCES:

**FUND1**	
**The 'population nutrition' budget, as a proportion of total health spending and/or in relation to the diet-related NCD burden sufficiently contributes to reducing diet-related NCD's.**<br><br>**(This indicator isn't being included in the online rating process)**	
**International best practice examples (benchmarks)**	**New Zealand:** The total funding for population nutrition was estimated at about $67 million or 0.6% of the health budget during 2008/09 Healthy Eating Healthy Action period. Dietary risk factors account for 11.4% of health loss in New Zealand.<br><br>**Thailand:** According to the most recent report on health expenditure in 2012 the government greatly increased budget spent on policies and actions related to nutrition (excluding food, hygiene and drinking water control). Total expenditure on health related to nutrition specifically from local governments was 29,434.5 million Baht (7.57% of total health expenditure from public funding agencies), which was ten times over the budget spending on nutrition in 2011. Dietary risk factors account for more than 10% of health loss in Thailand.

**FUND2**	
**Government funded research is targeted for improving food environments, reducing obesity, NCDs and their related inequalities**	
**International best practice examples (benchmarks)**	**Australia:** The National Health and Medical Research Council (NHMRC) Act requires the CEO to identify major national health issues likely to arise. The National Health Priority Areas (NHPAs) articulate priorities for research and investment and have been designated by Australian governments as key targets because of their contribution to the burden of disease in Australia. For the 2015-16 Corporate Plan, obesity, diabetes and cardiovascular health are three of these NHPAs.<br><br>**Thailand:** The National Research Council funded more research projects on obesity and diet-related chronic diseases (such as diabetes, cardiovascular diseases and hypertension) in 2014, accountable for almost six times over the research funding in 2013 (from 6,875,028 baht in 2013 to 37,872,416 baht in 2014)<br><br>**Ireland:** The Food Institutional Research Measure (FIRM) is funded by the Department of Agriculture, Food and the Marine and is the primary national funding mechanism for food research in higher education institutions and other public research institutes. Beneficiaries are required to widely disseminate the results of their research. FIRM aims to develop public good technologies that will underpin a competitive, innovative and sustainable food manufacturing and

	marketing sector. The programme is creating a base of knowledge and expertise in generic technologies that will support a modern, consumer-focused industry and build Ireland's capacity for R&D (Marine, 2017). The Health Research Board (HRB) is a statutory agency under the aegis of the Department of Health. It's the lead agency in Ireland responsible for supporting and funding health research, information and evidence, which aims to improve people's health and to enhance healthcare delivery.

**FUND3**	
**There is a statutory health promotion agency in place that includes an objective to improve population nutrition with a secure funding stream**	
**International best practice examples (benchmarks)**	**Australia:** The Victorian Health Promotion Foundation (VicHealth) was the world's first health promotion foundation, established by the Victorian Parliament as part of the Tobacco Act of 1987 (for the first 10 years through a hypothecated tobacco tax) through which the objectives of VicHealth are stipulated. VicHealth continues to maintain bipartisan support.<br><br>**Germany:** The **Federal Centre for Health Education** and the Federal Center for Nutrition disseminate guidelines and health promotion strategies to the general public and stakeholders and multipliers<br><br>**The Netherlands:** The Dutch Nutrition Center (https://www.voedingscentrum.nl/nl/service/over-ons.aspx) is 100% funded by the government and offers consumers and professionals scientific and independent information about a healthy, safe and sustainable food choice. The famous 'Wheel of Five' Guidelines is one of their products.

DOMAIN 12 - PLATFORMS FOR INTERACTION

<table>
<tr><th colspan="2">PLAT1</th></tr>
<tr><td colspan="2">There are robust coordination mechanisms across departments and levels of government (national, state and local) to ensure policy coherence, alignment, and integration of food, obesity and diet-related NCD prevention policies across governments.</td></tr>
<tr><td>International best practice examples (benchmarks)</td><td>Finland: The Finnish National Nutrition Council is an inter-governmental expert body under the Ministry of Agriculture and Forestry with advisory, coordinating and monitoring functions. It is composed of representatives elected for three-year terms from government authorities dealing with nutrition, food safety, health promotion, catering, food industry, trade and agriculture (World Cancer Research Fund, 2016a).

Malta: Based on the Healthy Lifestyle Promotion and Care of NCDs Act (2016), Malta established an inter-ministerial Advisory Council on Healthy Lifestyles in August 2016 to advise the Minister of Health on any matter related to healthy lifestyles. In particular, the Advisory Council advises on a life course approach to physical activity and nutrition, and on policies, action plans and regulations intended to reduce the occurrence of NCDs. The prime minister appoints the chair and the secretary of the Advisory Council, while the ministers of education, health, finance, social policy, sports, local government, and home affairs appoint one member each (World Cancer Research Fund, 2016a).

Australia: There are several forums and committees for the purpose of strengthening food regulation with representation from New Zealand and Health Ministers from Australian States and Territories, the Australian Government, as well as other Ministers from related portfolios (e.g. Primary Industries). Where relevant, there is also representation from the Australian Local Government Association.

Ireland: The Department of Health, through 'A Healthy weight for Ireland', Obesity Policy and Action Plan 2016 - 2025, will provide leadership, engage and co-ordinate multi-sectorial action and implement best practice in the governance of the OPAP. The department of health and safefood are taking action to establish a multi stakeholder partnership to share knowledge and initiative on healthy weight initiatives (Department of Health, 2016a)</td></tr>
</table>

PLAT2	
**There are formal platforms (with clearly defined mandates, roles and structures) for regular interactions between government and the commercial food sector on the implementation of healthy food policies and other related strategies**	
**International best practice examples (benchmarks)**	**UK:** The UK 'Responsibility Deal' was a UK government initiative to bring together food companies and NGOs to take steps (through voluntary pledges) to address NCDs. It was chaired by the Secretary of State for Health and included senior representatives from the business community (as well as NGOs, public health organisations and local government). A number of other subgroups were responsible for driving specific programs relevant to the commercial food sector.<br><br>**Norway:** The letter of intent (Memorandum of Understanding, MoU) for facilitating a healthier diet in the population is a signed agreement between the Norwegian health authorities and food industry (food and trade organizations, food and beverage manufacturers, food retailers and food service industry) in a *Partnership for a healthier diet.* The MoU was signed in 2016 and lasts until 2021. The agreement contains specific quantitative goals related to reducing the intake of salt, added sugar and saturated fat, and increasing the intake of fruits and berries, vegetables, whole grain foods, fish and seafood in the population. The Partnership is organized in a Coordination group with representatives from the main partners including the health authorities. The Coordination group reports to the Minister's food industry group (lead by the Minister for the Elderly and Public Health) that ensures dialogue and political focus on the areas of action. The Coordination group is assisted by a Secretariat organized by the Directorate of Health. A Reference group of scientists within nutrition, food technology, consumer behaviour, psychology and marketing provide expert advice to the coordination group.

PLAT3	
**There are formal platforms (with clearly defined mandates, roles and structures) for regular interactions between government and civil society on the development, implementation and evaluation of healthy food policies and other related strategies.**	
**International best practice examples (benchmarks)**	**Brazil:** The National Council of Food and Nutrition Security (CONSEA) is a formal advisory platform made up of civil society (2/3) and government reps (1/3). It is a participatory instrument for designing, suggesting, implementing and evaluating food and nutritional security policy. Through CONSEA, civil society has been able to influence policy directions more directly. CONSEA supported Congress to pass a bill obliging local governments to buy at least 30% of the food destined for school meals from small-scale farmers.

PLAT4	
**The governments work with a system-based approach with (local and national) organisations/partners/groups to improve the healthiness of food environments at a national level.**	
**International best practice examples (benchmarks)**	**New Zealand:** Healthy Families NZ is a large-scale initiative that brings community leadership together in a united effort for better health. It aims to improve people's health where they live, learn, work and play, in order to prevent chronic disease. Led by the Ministry of Health, the initiative will focus on ten locations in New Zealand in the first instance. It has the potential to impact the lives of over a million New Zealanders. The Government has allocated $40 million over four years to support Healthy Families NZ.<br><br>**Australia:** Healthy together Victoria in Australia focuses on addressing the underlying causes of poor health in children's settings, workplaces and communities by encouraging healthy eating and physical activity and reducing smoking and harmful alcohol use. Healthy Together Victoria incorporates policies and strategies to support good health across Victoria, as well as locally-led Healthy Together Communities. The initiative was originally jointly funded by the State Government of Victoria and the Australian Government through the National Partnership Agreement on Preventive Health (Government of South Australia, 2016). It is unclear at this stage whether funding for Healthy Together Victoria will continue or not.

DOMAIN 13 - HEALTH IN ALL POLICIES

**HIAP1**	
**There are processes in place to ensure that population nutrition, health outcomes and reducing health inequalities or health impacts in vulnerable populations are considered and prioritised in the development of all government policies relating to food**	
**International best practice examples (benchmarks)**	**Slovenia:** A Health Impact Assessment was undertaken in Slovenia to assess the health effects of agricultural policy at national level policy analysis, rapid appraisal workshops with stakeholders from a range of backgrounds, review of research evidence relevant to the agricultural policy, analysis of Slovenian data for key health-related indicators, a report on the findings to a key cross-government group and evaluation.<br><br>**Ireland: Step 9** of the ' A Healthy Weight for Ireland', Obesity Policy and Action Plan 2016-2025 aims to, allocate resources according to need, in particular to those population groups most in need of support in the prevention and management of obesity, with particular emphasis on families and children during the first 1,000 days of life. The priority actions to commence in first year were to, assess the needs of vulnerable groups as the basis of allocation of resources for preventative and treatment services for children and adults (Department of Health, 2016b). The Healthy Ireland fund was established in 2017 with an allocation of €5 million and with additional allocations of €5 million in 2018 and 2019. The first round of the Fund was distributed through Local Community Development Committees, Children and Young Person's Services Committees and statutory organisations (Oireachtas, 2019).

**HIAP2**	
**There are processes e.g. Health Impact Assessment's (HIAs) to assess and consider health impacts during the development of other non-food policies.**	
**International best practice examples (benchmarks)**	**South Australia:** Established in 2007, the implementation of Health in All Policies (HiAP) in South Australia has been supported by a high-level mandate from central government, an overarching framework which is supportive of a diverse program of work, a commitment to work collaboratively and in partnership across agencies, and a strong evaluation process. The government has established a dedicated HiAP team within South Australia Health to build workforce capacity and support Health Lens Analysis projects. Since 2007, the South Australian HiAP approach has evolved to remain relevant in a changing context. However, the purpose and core principles of the approach remain unchanged. There have been five phases to the work of HiAP in South Australia between 2007 and 2016: 1) Prove concept and practice emerges (2007-2008), 2) Establish and apply methodology (2008-2009), 3) Consolidate and grow (2009-2013), 4) Adapt and review (2014) and 5) Strengthen and systematize (2015-2016).

Bibliography

ADMINISTRATION, F. A. D. 2019. FDA Nutrition Innovation Strategy. .

ADVERTISING STANDARDS AUTHORITY. 2018. *Food: HFSS Media Placement* [Online]. Available: https://www.asa.org.uk/advice-online/food-hfss-media-placement.html [Accessed].

BAE, S. G., J.Y. KIM, K.Y. KIM, S.W. PARK, J. BAE, AND W.K. LEE, 2012. Changes in dietary behavior among adolescents and their association with government nutrition policies in Korea, 2005-2009. J Prev Med Public Health, 45(1): p. 47-59.

BIRO, A. 2015. Did the junk food tax make the Hungarians eat healthier? Food Policy. 54: p. 107-115.

BROADCASTING AUTHORITY OF IRELAND 2013. BAI Children's Commercial Communications Code.

BROADCASTING AUTHORITY OF IRELAND. 2019. *Codes and Standards* [Online]. Available: https://www.bai.ie/en/codes-standards/#al-block-5 [Accessed].

CENTRES FOR DISEASE CONTROL 2016. National Health and Nutrition Examination Survey. Available from: http://www.cdc.gov/nchs/nhanes.htm.

CHANTAL, J. A. H., S., 2017. Development of a new front-of-pack nutrition label in France: the five colour Nutri-Score.

CITIZENS INFORMATION 2014. Freedom of information. [online] available: https://www.citizensinformation.ie/en/government_in_ireland/national_government/standards_and_accountability/freedom_of_information.html.

COLCHERO, M. A., B.M. POPKIN, J.A. RIVERA, AND S.W. NG, 2016. Beverage purchases from stores in Mexico under the excise tax on sugar sweetened beverages: observational study. BMJ 352: p. h6704.

COLRUYT GROUP. 2018. *What is the Nutri-Score? [online] available: https://nutriscore.colruytgroup.com/colruytgroup/en/about-nutri-score/* [Online]. [Accessed].

COMMONWEALTH OF AUSTRALIA 2016. Health Star Rating System. Available from: http://healthstarrating.gov.au/internet/healthstarrating/publishing.nsf/content/home.

CONSEA Building up the National Policy and System for Food and Nutrition Security: the Brazilian experience. Available from: https://www.fao.org.br/download/Seguranca_Alimentar_Ingles.pdf.

CORREA, T., FIERRO, C., REYES, M., DILLMAN CARPENTIER, F. R., TAILLIE, L. S. & CORVALAN, C. 2019. "Responses to the Chilean law of food labeling and advertising: exploring knowledge, perceptions and behaviors of mothers of young children". *International Journal of Behavioral Nutrition and Physical Activity,* 16**,** 21.

CORVALÁN C, R. M., GARMENDIA ML, UAUY R, 2018. Structural responses to the obesity and non-communicable diseases epidemic: Update on the Chilean law of food labelling and advertising. Obesity Reviews. 2018;1–8. https://

doi.org/10.1111/obr.12802.

DEPARMENT OF HEALTH 2013. Guide to creating a front of pack (FoP) nutrition label for pre-packed products sold through retail outlets. Available from: https://www.gov.uk/government/uploads/system/uploads/attachment_data/file/300886/2902158_FoP_Nutrition_2014.pdf.

DEPARTMENT OF HEALTH 2013. Healthy Ireland - a framework for improved health and wellbeing 2013 - 2025. Dublin: Department of Health.

DEPARTMENT OF HEALTH. 2014. *National salth reduction initiative corporate achievements 2009-2014.* [Online]. Available: https://www1.nyc.gov/assets/doh/downloads/pdf/cardio/nsri-corporate-commitments.pdf [Accessed].

DEPARTMENT OF HEALTH 2016a. A Healthy Weight for Ireland. Obesity Policy and Action Plan: 2016-2025.

DEPARTMENT OF HEALTH 2016b. A Healthy Weight for Ireland: Obesity Policy and Action Plan 2016 – 2025.
DEPARTMENT OF HEALTH 2019a. Healthy Ireland Fund 2019- 2021. Expression of Interest Guidelines. [online] Accessed on https://drive.google.com/file/d/1rqVbr_YILdi12GqeGNgPnILPaEbrCBKB/view.
DEPARTMENT OF HEALTH 2019b. Taoiseach and Ministers launch Healthy Ireland campaign. [online] Accessed on https://www.gov.ie/en/news/20cff4-taoiseach-and-health-ministers-to-launch-2019-healthy-ireland-campai/.
DEPARTMENT OF HEALTH AND MENTAL HYGIENE Food Preparation and Food Establishments (Article 81, Section 49) - Sodium Warning. Available from: http://rules.cityofnewyork.us/content/food-preparation-and-food-establishments-article-81-section-49-sodium-warning-0.
DOWNS, S., BLOEM, M AND GRAZIOSE, MM., 2017. Salt and the city: a preliminary examination of New York City's sodium warning labels. .
FOOD SAFETY AUTHORITY OF IRELAND 2011. Scientific Recommendations for Healthy Eating Guidelines in Ireland [Online]. Dublin: Food Safety Authority of Ireland.
FOOD STANDARDS AUSTRALIA NEW ZEALAND 2013. Stakeholder Engagement Strategy 2013-2016. FSANZ New Zealand: Wellington.
FUNDO NACIONAL DE DESINVOLVIMENTO DA EDUCACAO 2016. Sobre o PNAE. Available from: http://www.fnde.gov.br/programas/alimentacao-escolar.
GERMAN NUTRITION SOCIETY. *Nutrition Reports. [online] available: https://www.dge.de/en/nutrition-reports/* [Online]. [Accessed].
GLOBAL AGRICULTURAL INFORMATION NETWORK 2018. Update on Chilean Advertising of Packaged Foods and Beverages. [online] available: https://apps.fas.usda.gov/newgainapi/api/report/downloadreportbyfilename?filename=Update%20on%20Chilean%20Advertising%20of%20Packaged%20Foods%20and%20Beverages%20_Santiago_Chile_2-14-2018.pdf.
GOVERNMENT OF CANADA 2016. Symposium on Sodium Reduction in Foods Meeting Report. Available from: https://www.canada.ca/en/services/health/symposium-sodium-reduction-foods-meeting-report.html.
GOVERNMENT OF SOUTH AUSTRALIA 2016. Health in All Policies: The South Australian Approach. Available from: http://www.sahealth.sa.gov.au/wps/wcm/connect/public+content/sa+health+internet/health+reform/health+in+all+policies.
GREATER LONDON AUTHORITY 2019. TfL junk food ads ban will tackle child obesity. [online] available: https://www.london.gov.uk/what-we-do/business-and-economy/food/tfl-junk-food-ads-ban-will-tackle-child-obesity.
HEALTH AND SOCIAL CARE INFORMATION CENTRE 2016. National Child Measurement Programme. Available from: http://www.hscic.gov.uk/ncmp.
HEALTH COUNCIL OF THE NETHERLANDS 2015. Dutch dietary guidelines 2015. [online] available: file:///C:/Users/charlottegriffin/Downloads/advisory-report-dutch-dietary-guidelines-2015.pdf.
HEALTH RESEARCH BOARD 2016. Research Evidence Action. HRB Strategy 2016-2020.
HEALTH SERVICE EXECUTIVE 2019. HSE Vending Policy
HEALTHY EATING ADVISORY SERVICE 2017. Improving wellbeing through healthy eating.
HEALTHY IRELAND 2013-2025. A framework for improved health and wellbeing 2013-2025.
HEALTHY IRELAND 2016. Healthy Food for Life. Revised healthy eating guidelines and Food Pyramid rationale. .
HEALTHY IRELAND 2017. Nutrition Standards for School Meals
HELSEDIREKTORATET 2016. Partnership for a healthier diet. [online] available: https://www.helsedirektoratet.no/english/partnership-for-a-healthier-diet.

HOFMAN, K. J. & TOLLMAN, S. M. 2013. Population health in South Africa: a view from the salt mines. *The Lancet Global Health,* 1**,** e66-e67.

KELLY, P. M., A. DAVIES, A.J. GREIG, AND K.K. LEE, 2016. Obesity Prevention in a City State: Lessons from New York City during the Bloomberg Administration. Front Public Health, 4: p. 60.

KENT, M. P., DUBOIS, L. & WANLESS, A. J. I. J. O. P. O. 2011. Food marketing on children's television in two different policy environments. 6**,** e433-441.

LEDERER, A., C.J. CURTIS, L.D. SILVER, AND S.Y. ANGELL, 2014. Toward a healthier city: nutrition standards for New York City government. Am J Prev Med 46(4): p. 423-8.

LOCK, K., M. GABRIJELCIC-BLENKUS, M. MARTUZZI, P. OTOREPEC, P. WALLACE, C. DORA, J.M. ZAKOTNIC, 2003. Health impact assessment of agriculture and food policies: lessons learnt from the Republic of Slovenia. Bull World Health Organ, 81(6): p. 391-8.

MARINE, D. O. A. F. A. 2017. *Food Institutional Research Measure* [Online]. Available: https://www.agriculture.gov.ie/research/competitivenationalprogrammes/foodinstitutionalresearchmeasurefirm/ [Accessed].

MASSRI, C., SUTHERLAND, S., KÄLLESTÅL, C. & PEÑA, S. J. A. J. O. P. H. 2019. Impact of the Food-Labeling and Advertising Law Banning Competitive Food and Beverages in Chilean Public Schools, 2014–2016. 109**,** 1249-1254.

MATBRANSJENS FAGLIGE UTVALG 2019. Guidelines for marketing food and drink aimed at children. [online] available: http://www.mfu.as/85197-Retningslinjer-for-markedsforing-oppdatert-1-10-2019.

MCAVOY, H., BERGIN, D., KIERNAN, R., KEATING, T., 2018. Consultation on the development of a Healthy Workplaces Framework for Ireland: A report prepared for the Department of Health. Dublin: Institute of Public Health in Ireland.

MFU. 2016. *Code for marketing of food and drink aimed at children updated 1/9-2016* [Online]. Available: http://www.mfu.as/44552-Code-for-marketing-of-food-and-drink-aimed-at-children-updated-1-9-2016 [Accessed].

MINISTRY OF EDUCATION, N. F. F. E. D. 2016. Resolution No 26 of 17 June 2013 (Ministério da Educação, Fundo Nacional de Desenvolvimento da Educação, Resolução No 26 de 17 de junho de 2013). Ministry of Education: Brazil.

MINISTRY OF FOOD AND DRUG SAFETY 2017. The Special Act on the Safety Management of Children's Dietary Life. Available from: https://www.mfds.go.kr/eng/index.do?nMenuCode=66.

MINISTRY OF HEALTH BRAZIL 2011. Health Surveillance Secretariat: Health situation analysis department. Strategic action plan to tackle noncommunicable diseases in Brazil 2011-2022. Ministry of Health: Brazil.

MINISTRY OF HEALTH BRAZIL 2014. Dietary guidelines for the Brazilian population. Available from: http://www.foodpolitics.com/wp-content/uploads/Brazilian-Dietary-Guidelines-2014.pdf.

MINISTRY OF HEALTH NEW ZEALAND 2016. Healthy Families NZ. Available from: http://www.health.govt.nz/our-work/preventative-health-wellness/healthy-families-nz.

MINISTRY OF HEALTH SOUTH AFRICA 2013. Strategic Plan for the Prevention and Control of Non-Communicable Diseases 2013-17. Available from: http://www.hsrc.ac.za/uploads/pageContent/3893/NCDs%20STRAT%20PLAN%20%20CONTENT%208%20april%20proof.pdf.

MINISTRY OF PUBLIC HEALTH, W. A. S. Food Database. [online] available: https://www.levensmiddelendatabank.nl/Security/Login.aspx?ReturnUrl=%2f.

MONTEIRO, C. A., G. CANNON, J.C. MOUBARAC, A.P. MARTINS, C.A. MARTINS, J. GARZILLO, P.C. JAIME, 2015. Dietary guidelines to nourish humanity and the planet in the twenty-first century. A blueprint from Brazil. Public Health Nutr, 18(13): p. 2311-22.

MORLEY, B., SCULLY, M., MARTIN, J., NIVEN, P., DIXON, H. & WAKEFIELD, M. 2013. What types of nutrition menu labelling lead consumers to select less energy-dense fast food? An experimental study. *Appetite,* 67**,** 8-15.

NATIONAL AGREEMENT TO IMPROVE PRODUCT COMPOSITION 2017. Available from: http://www.akkoordverbeteringproductsamenstelling.nl/dsresource?type=pdf&disposition=inline&objectid=rivmp:303682&versionid=&subobjectname=.

NATIONAL INSTITUTE FOR HEALTH AND WELFARE 2019. EATING TOGETHER -food recommendations for families with children.

NATIONAL INSTITUTE FOR PUBLIC HEALTH AND THE ENVIRONMENT MINISTRY OF HEALTH, W. A. S. 2011. Overview of food consumption surveys. [online] available: https://www.rivm.nl/voedselconsumptiepeiling/overzicht-voedselconsumptiepeilingen.

NATIONAL NUTRITION COUNCIL 2017. Eating and learning together – recommendations for school meals.

NATIONAL NUTRITION COUNCIL 2018. HEALTH AND JOY FROM FOOD - meal recommendations for early childhood education and care.

NATIONAL NUTRITION SURVEILLANCE CENTRE Childhood Obesity Surveillance Initiative (COSI). [online] available: http://www.ucd.ie/nnsc/research/ourresearch/childhoodobesitysurveillanceinitiativecosi/.

NETHERLANDS, G. O. T. 2019. The National Prevention Agreement.

NETHERLANDS NUTRITION CENTRE 2017. Guidelines for Healthier Canteens Fact sheet. [online] available: https://mobiel.voedingscentrum.nl/Assets/Uploads/voedingscentrum/Documents/Professionals/Pers/Factsheets/English/Fact%20sheet%20Guidelines%20for%20Healthier%20Canteens.pdf.

NEW YORK CITY HEALTH 2017 National Salt Reduction Initiative (NSRI). Available from: https://www1.nyc.gov/site/doh/health/health-topics/national-salt-reduction-initiative.page.

NG, S. W., RIVERA, J. A., POPKIN, B. M. & COLCHERO, M. A. J. P. H. N. 2019. Did high sugar-sweetened beverage purchasers respond differently to the excise tax on sugar-sweetened beverages in Mexico? 22**,** 750-756.

NORWEGIAN MINISTRY OF LOCAL GOVERNMENT AND MODERNISATION 2019. Fourth Norwegian Action Plan Open Government Partnership (OGP) 2019 - 2021.

NUTRITION RESOURCE CENTRE 2017. Ontario's Healthy Menu Choices Act In Effect. [online] available https://opha.on.ca/Nutrition-Resource-Centre/News/NRC-News/2017/Ontario%E2%80%99s-Healthy-Menu-Choices-Act-In-Effect.aspx.

NYC HEALTH DEPARTMENT 2019. National Salt and Sugar Reduction Initiative (NSSRI). .

OFFICE OF THE FEDERAL REGISTER 2013. Food Labeling: Nutrition Labeling of Standard Menu Items in Restaurants and Similar Retail Food Establishments. Available from: https://www.federalregister.gov/regulations/0910-AG57/food-labeling-nutrition-labeling-of-standard-menu-items-in-restaurants-and-similar-retail-food-estab.

OIREACHTAS 2019. Obesity Strategy Dáil Éireann Debate, Tuesday - 12 March 2019. [online] Accessed https://www.oireachtas.ie/en/debates/question/2019-03-12/275/.

PARLIMENT UK 2017. Financial Statement. [online] available on https://hansard.parliament.uk/Commons/2017-03-08/debates/9128238B-4A1C-4EEA-A6F0-6FEB0B5024D8/FinancialStatement#contribution-7B30FFC0-4B7A-4D63-8075-3C7F83EDC5.

PETERS, S., DUNFORD, E., WARE, L., HARRIS, T., WALKER, A., WICKS, M., VAN ZYL, T., SWANEPOEL, B., CHARLTON, K. & WOODWARD, M. J. N. 2017. The sodium content of processed foods in South Africa during the introduction of mandatory sodium limits. 9**,** 404.

POBAL 2016. Healthy Ireland Fund. [online] Accessed on https://www.pobal.ie/programmes/healthy-ireland-fund/.

PUBLIC HEALTH ENGLAND 2017. Sugar Reduction: Achieving the 20% A technical report outlining progress to date, guidelines for industry, 2015 baseline levels in key foods and next steps. .

PUBLIC HEALTH ENGLAND 2018. Sugar Reduction Programme. Progress made by industry in the first year.

PUBLIC HEALTH ENGLAND 2019. Sugar reduction: Report on progress between 2015 and 2018. September 2019.
RATHBONE GREENBANK INVESTMENTS 2019. The UK sugar tax: one year on. [online] available: https://www.rathbonegreenbank.com/insight/uk-sugar-tax-one-year.
ROBERT KOCH INSTITUT. *The AdiMon Indicator System. [online] available: https://www.rki.de/EN/Content/Health_Monitoring/HealthSurveys/AdiMon/AdiMon_inhalt.html* [Online]. [Accessed].
RØYNSTRAND, S. 2017. *Spiser mosjonister og supermosjonister sunt?: En kartlegging av kostholdet til mannlige utholdenhetsmosjonister og supermosjonister.* Universitetet i Agder; University of Agder.
SAUDI FOOD & DRUF AUTHORITY 2018. SFDA launches Healthy Food Regulation Strategy. [online] available: https://www.sfda.gov.sa/en/food/news/Pages/f11-9-2018a1.aspx.
STANDARDS IN PUBLIC OFFICE COMMISSION 2019. 2018 Code of Conduct for persons carrying on lobbying acitivities. [online] Accessed on https://www.lobbying.ie/media/6119/code-of-conduct-english-final-version-for-web.pdf.
STATE SERVICES COMMISSION Best Practice Guidelines for Departments Responsible for Regulatory Processes with Significant Commercial Implications. Available from: http://www.ssc.govt.nz/resources/2814/all-pages.
THE CENTRAL GOVERNMENT FOR THE NETHERLANDS 2014. Agreed improvement product composition salt, saturated fat, sugar. [online] available: https://www.rijksoverheid.nl/documenten/convenanten/2014/01/23/akkoord-verbetering-productsamenstelling-zout-verzadigd-vet-suiker.
THE CHIP GROUP 2016. The Chip Group: Helping you make better chips. Available from: http://blog.thechipgroup.co.nz/.
THE DUTCH INSITITUTE OF PUBLIC HEALTH AND ENVIRONMENT 2017. Agreed improvement product composition salt, saturated fat, sugar. [online] available: https://www.rijksoverheid.nl/documenten/convenanten/2014/01/23/akkoord-verbetering-productsamenstelling-zout-verzadigd-vet-suiker.
THE ORGANISATION FOR ECONOMIC CO-OPERATION AND DEVELOPMENT 2019. OECD Reviews of Public Health: Chile: A Healthier Tomorrow, OECD Publishing, Paris.

https://doi.org/10.1787/9789264309593-en.
US NATIONAL INSTITUTES OF HEALTH 2016. Time-Sensitive Obesity Policy and Program Evaluation (R01). Available from: http://grants.nih.gov/grants/guide/pa-files/PAR-12-257.html.
VEERMAN, J. L. A. L. J. C. 2013 Removing the GST exemption for fresh fruits and vegetables could cost lives. Med J Aust, 199(8): p. 534-5.
WORLD CANCER RESEARCH FUND 2016a. NOURISHING Framework - Harness food supply chain. Available from: http://www.wcrf.org/int/policy/nourishing-framework/harness-food-supply-chain.
WORLD CANCER RESEARCH FUND 2016b. NOURISHING Framework - Improving the food supply. Available from: http://www.wcrf.org/int/policy/nourishing-framework/improve-food-supply.
WORLD CANCER RESEARCH FUND 2016c. NOURISHING Framework - Nutrition labels. Available from: http://www.wcrf.org/int/policy/nourishing-framework/nutrition-labels.
WORLD CANCER RESEARCH FUND 2016d. NOURISHING Framework - Offer healthy foods. Available from: http://www.wcrf.org/int/policy/nourishing-framework/offer-healthy-foods.
WORLD CANCER RESEARCH FUND 2016e. NOURISHING Framework - Restrict food marketing. Available from: http://www.wcrf.org/int/policy/nourishing-framework/restrict-food-marketing.
WORLD CANCER RESEARCH FUND 2016f. NOURISHING Framework - Use economic tools. Available from: http://www.wcrf.org/int/policy/nourishing-framework/use-economic-tools.

WORLD CANCER RESEARCH FUND 2016 NOURISHING Framework - Set retail environment incentives. Available from: http://www.wcrf.org/int/policy/nourishing-framework/set-retail-environment-incentives.
WORLD CANCER RESEARCH FUND 2018. NOURISHING framework. Use economic tools to address food affordability and purchase incentives. [online] available on https://www.wcrf.org/sites/default/files/Use-economic-tools.pdf.
WORLD HEALTH ORGANISATION 2017. France becomes one of the first countries in Region to recommend colour-coded front-of-pack nutrition labelling system.
WORLD PUBLIC HEALTH NUTRITION ASSOCIATION UPDATE TEAM 2014. Brazilian dietary guidelines. At last! Guidelines based on food and meals!. Available from: http://wphna.org/wp-content/uploads/2015/01/WN-2014-05-12-1050-1051-Update-Guia.pdf.
